# Subglacial Lake Vostok (Antarctica) Accretion Ice Contains a Diverse Set of Sequences from Aquatic, Marine and Sediment-Inhabiting Bacteria and Eukarya

**DOI:** 10.1371/journal.pone.0067221

**Published:** 2013-07-03

**Authors:** Yury M. Shtarkman, Zeynep A. Koçer, Robyn Edgar, Ram S. Veerapaneni, Tom D’Elia, Paul F. Morris, Scott O. Rogers

**Affiliations:** Department of Biological Sciences, Bowling Green State University, Bowling Green, Ohio, United States of America; The Roslin Institute, University of Edinburgh, United Kingdom

## Abstract

Lake Vostok, the 7^th^ largest (by volume) and 4^th^ deepest lake on Earth, is covered by more than 3,700 m of ice, making it the largest subglacial lake known. The combination of cold, heat (from possible hydrothermal activity), pressure (from the overriding glacier), limited nutrients and complete darkness presents extreme challenges to life. Here, we report metagenomic/metatranscriptomic sequence analyses from four accretion ice sections from the Vostok 5G ice core. Two sections accreted in the vicinity of an embayment on the southwestern end of the lake, and the other two represented part of the southern main basin. We obtained 3,507 unique gene sequences from concentrates of 500 ml of 0.22 µm-filtered accretion ice meltwater. Taxonomic classifications (to genus and/or species) were possible for 1,623 of the sequences. Species determinations in combination with mRNA gene sequence results allowed deduction of the metabolic pathways represented in the accretion ice and, by extension, in the lake. Approximately 94% of the sequences were from Bacteria and 6% were from Eukarya. Only two sequences were from Archaea. In general, the taxa were similar to organisms previously described from lakes, brackish water, marine environments, soil, glaciers, ice, lake sediments, deep-sea sediments, deep-sea thermal vents, animals and plants. Sequences from aerobic, anaerobic, psychrophilic, thermophilic, halophilic, alkaliphilic, acidophilic, desiccation-resistant, autotrophic and heterotrophic organisms were present, including a number from multicellular eukaryotes.

## Introduction

Lake Vostok is the largest of nearly 400 subglacial lakes that have been found in Antarctica [1]–[5], at least some of which are connected by subglacial rivers and streams [6]. As the overriding 4 km-thick glacier moves across Lake Vostok at a rate of 3 m yr^−1^, lake water freezes (i.e., accretes) to the bottom of the glacier creating a lake-water record that is a linear and temporal representation of the surface contents of the lake [7], [8]. At the Vostok 5G ice core drill site on the far side of the lake (relative to the glacier entry site), the meteoric ice is 3538 m thick, and the accretion ice is more than 231 m thick [9]. At that location, the glacier has passed over an embayment, near a peninsula/ridge, and then over part of the southern main lake basin [2], [8]. Ice that accreted in the vicinity of the embayment has relatively high concentrations of ions, organic carbon, biomass and mineral inclusions (termed “type I” – or type 1 - accretion ice) [8], [10], [11]. Over the majority of the lake, the accretion ice contains far fewer mineral inclusions, as well as lower concentrations of ions, organic carbon, and biomass [8], [10], [11]. This relatively clear ice is known as “type II” (or type 2) accretion ice.

Several studies have described the biotic and abiotic components from Lake Vostok accretion ice core sections [8], [12]–[19]. Mean cell concentrations in the accretion ice ranged from <1 to several hundred cells ml^−1^. In most studies, the highest concentrations of cells and highest sequence diversities were reported in accretion ice from within or near the embayment in the southwest corner of the lake near the entry point of the glacier. Additionally, we and others demonstrated that the concentrations of total cells and viable cells were higher in the accretion ice than in the meteoric ice above [12], [14]–[16]. In the same studies, we cultivated, sequenced (rDNA regions) and identified 18 unique isolates of Bacteria and 31 unique isolates of Fungi from the same Lake Vostok accretion ice core sections. All were phylogenetically closest to species from aquatic, lake/ocean sediment, cold, polar and/or deep-sea environments. The compilation of results suggests that organisms are living and reproducing in Lake Vostok. For the present metagenomic/metatranscriptomic study, we examined two ice core sections that accreted in the vicinity of the embayment (at depths 3563 and 3585 m), and two ice core sections that accreted over the southern main lake basin (at 3606 and 3621 m). The results of this research provide a detailed view of the potential life in Lake Vostok.

## Materials and Methods

The ice core sections were selected from the USGS NICL (United States Geological Survey, National Ice Core Laboratory, Denver, CO), and were shipped frozen to our laboratory. Sample V5 consisted of Vostok 5G core sections at 3563 and 3585 m (type I ice), corresponding to ice that accreted in the vicinity of the embayment. Sample V6 included core sections 3606 and 3621 m (type II ice), corresponding to ice that accreted over a portion of the southern main basin of Lake Vostok. Each core section was surface sterilized using a method that had been previously developed, tested, described and utilized [14], [15], [20], [21]. Briefly, quartered ice core sections, 6–16 cm in length (total volume approximately 125 ml), were immersed in a 5.25% sodium hypochlorite solution (pre-chilled to 4°C for at least 2 h) for 10 s followed by three rinses with 800 ml of sterile water (4°C, 18.2 M*Ω*, <1 ppb total organic carbon, autoclaved). The sections were then melted in sterile funnels and meltwater was collected in 50 ml aliquots. Each aliquot comprised a “shell” of meltwater corresponding to the outer portion, and sequentially more interior portions of the ice core section. The meltwater was then frozen at −20°C. A total of 250 ml of meltwater was used for each sample (125 ml from each ice core section). The meltwater samples were filtered sequentially through 1.2, 0.45 and 0.22 µm Durapore filters (Millipore, Billerica, MA). The filtered meltwater was subjected to ultracentrifugation at 100,000×g (aseptically) for 16 hours to pellet cells and nucleic acids. The filtered meltwater contained small cells, cell debris, viruses and biomolecules (including RNA and DNA) from single-celled and multicellular organisms. Two control samples (purified water, 18.2 MΩ, <1 ppb total organic carbon; and the same water, autoclaved and subjected to concentration by ultracentrifugation) also were processed using the same protocols. The V5, V6, and control samples were ultracentrifuged on different days to lessen potential cross-contamination. Pellets were rehydrated in 50 µl of sterile 0.1X TE (1 mM Tris [pH 7.5], 0.1 mM EDTA). Nucleic acid extraction was performed using MinElute Virus Spin Kits (QIAGEN, Valencia, CA) according to the manufacturer’s instructions and eluted in 150 µl AVE buffer (with 0.04% sodium azide). The eluted nucleic acids were allowed to precipitate overnight at −20°C with 0.5 M NaCl in 80% ethanol. They were then pelleted by centrifugation at 16,000×g for 15 min, washed with cold 80% ethanol and centrifuged at 16,000×g for 5 min. They were dried under vacuum, and were resuspended in 15 µl 0.1X TE. All glassware, tubes, and piptet tips were treated with RNase Away (Life Technologies, Grand Island, NY) and autoclaved prior to use. Solutions and reagents were autoclaved (except those purchased as reaction mixes).

### cDNA Synthesis

DNA copies of the RNA were produced using a cDNA kit (Invitrogen SuperScript® Choice System, Invitrogen, Grand Island, NY, USA) according to the manufacturer’s instructions. Briefly, random hexamer primers were added to 10 µl of each nucleic acid sample, separately. They were mixed by pipetting and incubated at 70°C for 10 min, followed by chilling on ice. The first strand synthesis reaction consisted of the RNA and hexamer primers (above) in 50 mM Tris-HCl [pH 8.3], 75 mM KCl, 3 mM MgCl_2_, 10 mM DTT (dithiothreitol), 500 µM of each dNTP (dATP, dCTP, dGTP, and dTTP), in a 20 µl volume. After mixing, 200 U of SuperScript® II RT (reverse transcriptase) was added and mixed. This was incubated at 37°C for 1 h, and then placed on ice. The second strand synthesis reaction consisted of the first strand synthesis reaction in the following solution: 25 mM Tris-HCl [pH 7.5], 100 mM KCl, 5 mM MgCl_2_, 10 mM (NH_4_)_2_SO_4_, 0.15 mM ß-NAD^+^, 250 µM each dNTP, 10 U DNA ligase, 4 U DNA pol I, 2 U RNase H and 1.2 mM DTT, in a total volume of 150 µl. The solution was mixed and incubated at 16°C for 2 h, then placed on ice. Next, 10 U of T4 DNA polymerase was added, and the reaction mix was incubated at 16°C for 5 min, and then was stopped by the addition of EDTA to a final concentration of 30 mM. Then, 150 µl of chloroform/isoamyl alcohol (24∶1) was added and the contents of the tubes were mixed vigorously to form an emulsion. After 2 min of centrifugation at 16,000×g, the aqueous layer was transferred into a new tube and the nucleic acids were precipitated with 0.5 M NaCl and cold 80% ethanol. The nucleic acids were precipitated at −20°C for 5 min, followed by centrifugation (at 16,000×g) for 20 min. The supernatant was decanted and the pellets were dried under vacuum for 15 min. Each contained both DNA (representing the metagenomic portion) and cDNA (representing the metatranscriptomic portion). Both were used to assure a sufficient mass of nucleic acids for pyrosequencing.

### Adapter Ligation and Chromatography

Pellets from the previous step were rehydrated in 18 µl of DEPC-treated water. Then, *Eco*RI (*Not*I) adapters were ligated to each end of the cDNA and DNA in the sample in order to provide defined ends for subsequent PCR amplification. The reaction mix consisted of: 66 mM Tris-HCl [pH 7.6], 10 mM MgCl_2_, 1 mM ATP, 14 mM DTT, 100 pmols *Eco*RI (*Not* I) adapters (AATTCGCGGCCGCGTCGAC, dsDNA), and 0.5 U of T4 DNA ligase, in a total volume of 50 µl. The mixture was incubated at 16°C for 16 h. DNA ligase was inactivated by heating at 70°C for 10 min. Each of the samples was then fractionated by column chromatography using Sephacryl S-500 HR with TEN buffer (10 mM Tris-HCl [pH 7.5], 0.1 mM EDTA, 25 mM NaCl), collecting twenty-four 35 µl fractions. A 5 µl aliquot of each was amplified by PCR using using a GeneAmp® PCR Reagent Kit with AmpliTaq® DNA Polymerase (Applied Biosystems, Carlsbad, CA, USA). Each reaction mixture contained: 10 mM Tris-HCl [pH 8.3], 50 mM KCl, 1.5 mM MgCl_2_, 0.001% (w/v) gelatin, 200 µM each dNTP, 1 U Ampli*Taq* DNA polymerase and 50 pmols *Eco*RI (*Not*I) adapter primers (AATTCGCGGCCGCGCTCGAC), each in 25 µl total volume. The thermal cycling program was: 94°C for 4 min; then 40 cycles of 94°C for 1 min, 55°C for 2 min, 72°C for 2 min; followed by an incubation for 10 min at 72°C. A 1 µl aliquot of each was subjected to 1% agarose gel electrophoresis at 5 V/cm in TBE (89 mM tris-base, 89 mM borate, 2 mM EDTA [pH 8.0]), containing 0.5 µg/ml ethidium bromide, and visualized by UV irradiation. Fractions that excluded small (<200 bp) and large (>2.0 kb) fragments were pooled, and were precipitated with NaCl and ethanol, washed, and dried (as above). Each was rehydrated in 35 µl of 0.1X TE.

### Addition of 454 A and B Sequences by PCR Amplification

Each of the pooled amplified samples was then reamplified using sequences that contained *Eco*RI/*Not*I sequences on their 3′ ends and 454-specific primer sequences on their 5′ ends, one primer with 454 sequence A (underlined): CGTATCGCCTCCCTCGCGCCA TCAGAATTCGCGGCCGCGTCGAC; and the other with 454 sequence B (underlined): CTATGCGCCTTGCCAGCCCGC TCAGAATTCGCGGCCGCGTCGAC). The thermal cycling program was: 94°C for 4 min; then 40 cycles of 94°C for 1 min, 55°C for 3 min, 72°C for 3 min; followed by an incubation for 10 min at 72°C. All PCR products were cleaned with a PCR purification kit (QIAGEN, Valencia, CA). The amplicons were quantified on agarose gels (as above) to calculate concentrations, based on comparisons to plasmid pGEM4Z (Promega, Madison, WI) standards on the same gel. After adjusting concentrations to approximately 1 µg/µl, 20 µg of each was sent to Roche Life Sciences 454 Technologies (Roche, Branford, CT) for sequencing using a 454 GS Junior System.

### Sequence Analysis

The sequences were extracted from the data file and organized using Python (Python Software Foundation) on the Ohio Super Computer (OSC, Columbus, OH, USA). Sequences were deposited in the GenBank nucleotide database at the National Center for Biotechnology Information (NCBI; accession numbers: JQ997163 - JQ997235; JQ997237 - JQ997322; JQ997324 - JQ997402; JQ997404 - JQ997547; JQ997549 - JQ998298; JQ998300 - JQ998745; JQ998747 -JQ999505; JQ999568 - JQ999624; JQ999909 - JQ999910; JQ997196 - JQ997198; JQ997274; JQ997284; JQ997285; JQ997287; JQ997308; JQ997309; JQ997361; JQ997374; JQ997375; JQ997378; JQ997384; JQ997393; JQ997394; JQ997443; JQ997448; JQ997457; JQ997460; JQ997469; JQ997487 - JQ997497; JQ997541; JQ997613; JQ997623; JQ997624; JQ997638; JQ997639; JQ997651; JQ997695; JQ997698; JQ997801; JQ997804; JQ997847; JQ998421; JQ998746; JQ999303; JQ999327; JQ999330; JQ999348; JQ999360; JQ999361; JJQ999365 - JQ999369; JQ999371; JQ999492; JQ999493; JQ999635 - JQ999829; JQ999837 - JQ999897; JQ999899; JQ999901 - JQ999905; JQ999507 - JQ999509; JQ999512; JQ999515; JQ999518 - JQ999521; JQ999523 - JQ999526; JQ999529 - JQ999530; JQ999533; JQ999538; JQ999540; JQ999545; JQ999549; JQ999552; JQ999554; JQ999556 - JQ999564; JQ999567; JQ999629; JQ999631; JQ999830; JQ999831; JQ999833 - JQ999835). The 454 reads were assembled using MIRA 3.0.5 (Whole Genome Shotgun and EST Sequence Assembler) [22] (Assembled sequences and reads are available upon request). Batch Mega-BLAST searches (e-value cuttoffs of 10^−10^) were performed to determine taxonomic and gene identities, retrieving the top 10 similar sequences that could be aligned over at least 100 nucleotides (nt). The top BLASTN hit that specified a genus and/or species name from the 10 retrieved was used to determine taxonomic classification. The sequences were divided into four categories: V5 rRNA genes, V6 rRNA genes, V5 mRNA genes and V6 mRNA genes (Tables S1, S2, S3, S4, S5, S6, S7, S8, S9, S10, S11, S12, S13). They were categorized according to the percent identity values in BLAST searches to known species, isolates and sequences. One set contained sequences that exhibited ≥97% identities (over at least 100 nt of continuous sequence with sequences in the NCBI database). Another set contained a subset of sequences exhibited ≥99% identities. A third set contained all of the sequences, regardless of percent identities. Each sequence was then categorized (according to the characteristics of the species indicated by the BLAST results) by temperature ranges, growth conditions, metabolic functions and ecological niches. Overall taxonomic proportions at the Domain, Kingdom and Phylum levels were determined using MG-RAST [23], Galaxy [24], and from the genus and species designations from the BLAST results. The mRNA sequences were used to determine metabolic function (using KAAS-KEGG [25]), as well as to determine or confirm taxa, where possible.

Sequences that were closest to possible vector sequences, repetitive elements, transposons and prophage were removed from the V5 and V6 data sets, to eliminate misidentification due to horizontal gene transfer events. Sequences and species that were similar to those found in the water controls were removed from the V5 and V6 data sets. These are listed in Tables S14 and S15. The sequence complexity was lower in the controls. Approximately 80% of the sequences in the control samples were from five species (*Propionibacterium acnes*, *E. coli*, *Homo sapiens*, *Psuedomonas fluorescens* and *Bos taurus*). Some of these may have originated in the PCR, or other, reagents (explained in Discussion).

## Results

**Figure 1:**
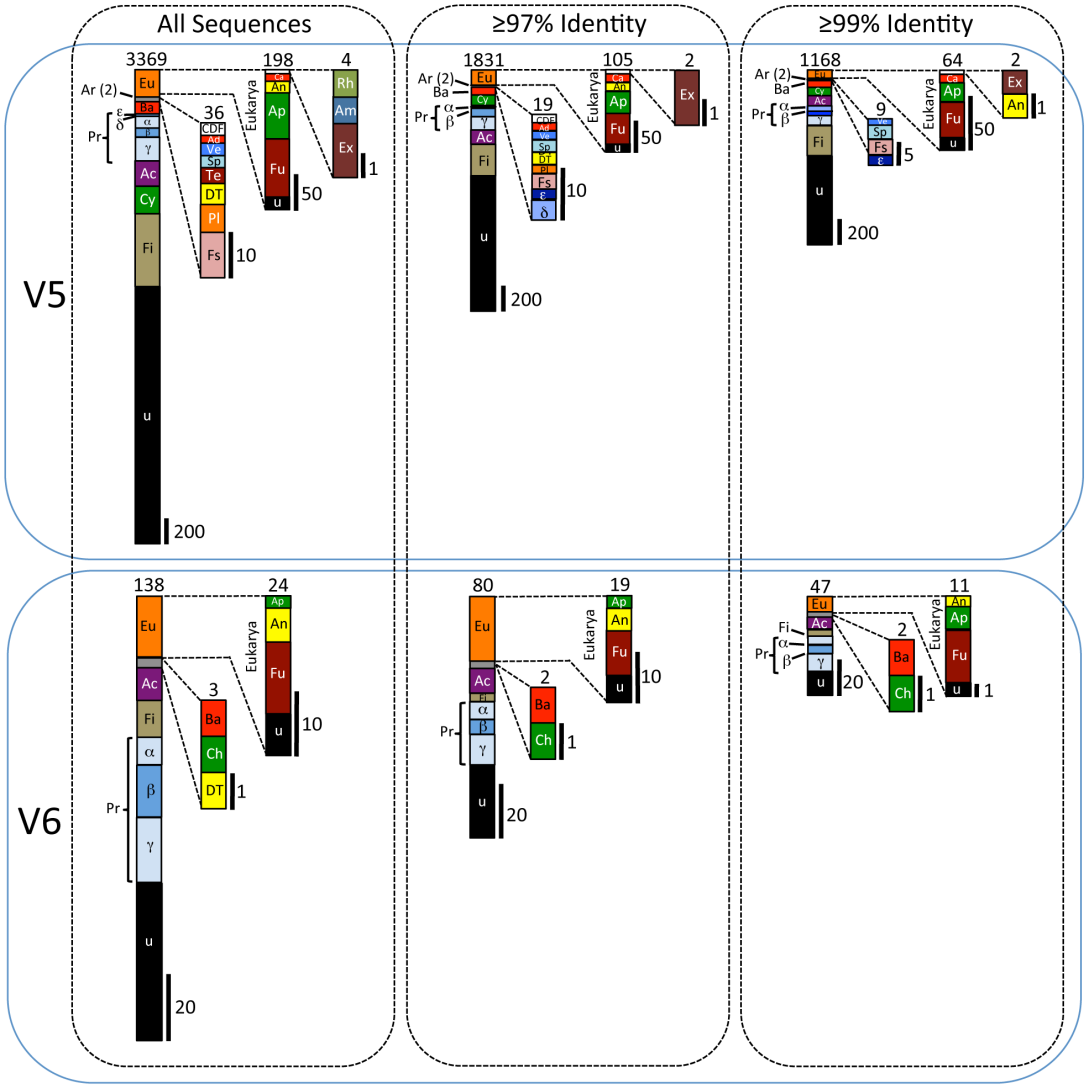
Summary of broad taxonomic proportions based on the metagenomic and metatranscriptomic data. The first column presents the proportion of unique sequences from all sequences in the entire data set, regardless of percent identity to sequences deposited in the NCBI nucleotide database. The upper row represents data from V5, and the lower row represents data from V6. The totals are represented in the bar graph on the left in each box, with numerical totals at the top. There were 3,507 unique sequences (3,369+138) in the entire data set, including 3,169 from Bacteria, 2 from Archaea and 198 from Eukarya in V5; and 114 Bacteria and 24 from Eukarya in V6. The middle column includes sequences that have identities between 97 and 100% with sequences in the NCBI database. There were 1,911 unique sequences, including 1,724 from Bacteria, 2 from Archaea and 105 from Eukarya in V5; and 61 from Bacteria and 19 from Eukarya in V6. The final column includes sequences that have identities between 99 and 100% with sequences in the NCBI database. There were 1,102 Bacteria, 2 Archaea and 64 Eukarya in V5; and 36 Bacteria and 11 Eukarya in V6. Scales (in number of sequences) are at the bottom right of each bar graph. Abbreviations: Ac = Actinobacteria; Ad = Acidobacteria; Am = Amoebozoa; An = Animalia; Ap = Archaeplastida; Greek alpha = Alphaproteobacteria; Ar = Archaea; Ba = Bacteroidetes; Greek beta = Betaproteobacteria; Ca = Chromalveolata; CDF = Chlorobi/Deferribacteres/Fibrobacteres; Ch = Chloroflexi; Cy = Cyanobacteria; Greek delta = Deltaproteobacteria; DT = Deinococcus/Thermus; Greek εpsilon = Epsilonproteobacteria; Eu = Eukarya; Ex = Excavata; Fi = Firmicutes; Fs = Fusobacteria; Fu = Fungi; Greek gamma = Gammaproteobacteria; Pl = Planctomyces; Pr = Proteobacteria; Rh = Rhizaria; Sp = Spirochaetes; Te = Tenericutes; u = uncultured/unidentified; Ve = Verrucomicrobia.

**Figure 2:**
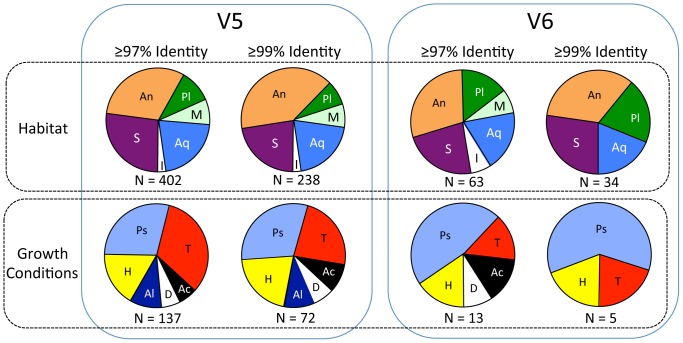
Summary of proportions of sequences in V5 (left) and V6 (right) categorized by habitat (upper row) and growth conditions (lower row) based on species with highest sequence identities. Each pie chart is comprised of sequences that were either ≥97% identity or ≥99% identity, and also could be classified by habitat (above) or growth conditions (below). Habitat abbreviations: An = animal associated (most are also found in soils and/or water); Aq = aquatic; I = ice, glaciers and/or polar; M = marine; Pl = plant associated (most are also found in soils and water); S = soils or sediments. Growth conditions abbreviations: Ac = acidophilic or acid tolerant; Al = alkaliphilic or alkali tolerant; D = desiccation resistant; H = halophilic or halotolerant; Ps = psychrophilic or psychrotolerant; T = thermophilic or thermotolerant. Number of sequences (N) used for each is indicated below each pie chart.

A total of 36,754,464 bp of sequence data was obtained from sample V5 that included 94,728 high quality 454 sequence reads, with a mean length of 388 bp. For the V6 sample, a total of 1,170,900 bp of sequence data was obtained that included 5,204 high quality reads, with a mean length of 225 bp. The lower quantity of sequence data for V6 might have resulted from lower nucleic acid concentrations (as we repored previously, [14], [15]), as well as higher degrees of degradation. Overall, approximately 15% of the sequences were unique, while the remaining 85% were additional copies from the unique set of sequences. A total of 3,369 unique sequences were derived from V5, of which 1,543 could be taxonomically classified (Figure 1; Tables S1, S2, S3, S4, S5, S6), and 138 unique assembled sequences were derived from V6, of which 80 could be taxonomically classified (Figure 1; Tables S7, S8, S9, S10). Approximately 94% of the unique sequences in V5 and 83% in V6 were from Bacteria. Only two unique Archaea sequences were found (both in V5), and they were most similar to methanotrophs from cold deep-ocean sediments. The remainder were Eukarya (4% in V5 and 17% in V6), including more than 150 unique sequences from multicellular organisms, most of which were Fungi. In general, the taxa were similar to organisms specific to lakes, brackish water, marine environments, soil, lake sediments, deep-sea sediments, deep-sea thermal vents, animals and plants (Figure 2). Sequences from autotrophs and heterotrophs were present.

### Bacteria and Archaea

Overall, the number of unique sequences identified as bacterial in the V5 sample was 3,169 (Figure 1). This included 1,724 (54% of the total) that matched NCBI sequences with ≥97% sequence identity, and 1,102 (35% of the total) that matched with ≥99% identity. A large proportion of the sequences (1,820 overall) were closest matches to sequences from uncultured and unidentified bacteria. Many of the sequence hits were from other environmental metagenomic studies. Of the taxa that could be identified, most were members of the phyla Firmicutes, Proteobacteria, Cyanobacteria, Actinobacteria and Bacteroides (Figure 1). This was true at all percent identity cut-off levels. Within the Proteobacteria, the data set included highest identities to sequences from members of the Alphaproteobacteria, Betaproteobacteria, Deltaproteobacteria, Epsilonproteobacteria and Gammaproteobacteria. The phyla proportions and the total number of sequences differed in the V6 sample (118 total, 61 at ≥97% identity and 36 at ≥99% identity; Figure 1). No sequences with high identities to Cyanobacteria, Deltaproteobacteria and Epsilonproteobacteria were found in the V6 sample. Only two sequences from Archaea were found, and both were in V5. They were closest to sequences found in cold deep-ocean sediments.

### Eukaryotes

While only about 6% of the unique sequences were eukaryotic (198 from V5 and 24 from V6), a variety of taxonomic groups were represented (Figure 1, all sequences). At the ≥97% identity level, the numbers decreased to 105 for V5, and 19 for V6. At the ≥99% identity level, they further decreased to 64 for V5, and 11 for V6. The majority were Fungi (81 sequences in V5 and 18 in V6, for all sequences; 50 and 14 in V5 and V6, respectively, at the ≥97% identity level; 34 and 7 in V5 and V6, respectively, at the ≥99% level), including one rRNA SSU sequence that was 99% similar to a marine fungus sequence that had been recovered from a deep-sea thermal vent [26]. Several sequences from members of the Animalia were found, including 14 sequences from arthropods (9 in V5 and 5 in V6). However, at the ≥97% identity level there were 5 sequences closest to those from arthropods, and at the ≥99% identity there were only 2. Some of these were closest matches to sequences from predatory or parasitic species, including taxa closest to members of *Daphnia* (planktonic crustaceans; 98% identity), Entomobryidae (slender springtails, some of which are aquatic; 89–98% identity) and other species (<90% sequence identity). Additionally, V5 contained sequences from one unidentified bilaterian (92% identity), a rotifer (closest to *Adineta* sp., a hardy, cosmopolitan, freshwater species; 98% identity), a tardigrade (closest to *Milnesium* sp., a hardy, predatory, cosmopolitan, freshwater species; 93% identity), a mollusk (most similar to *Nutricola tantilla*, a small marine bivalve [maximum diameter of 9 mm] that lives in sediments to about 120 m water depth; 100% identity) and a cniderian (related to *Nematostella* sp., a small sea anemone; 78% identity to a hypothetical protein).

Sequences closest to an uncultured lobster gut bacterium (93% identity), *Verminephrobacter* sp. (an annelid nephridia symbiont; 92% identity), *Renibacter salmonarium* (a salmonid fish pathogen; 98% identity), *Carnobacterium mobile* (associated with fish and shrimp; 95% identity), *Clostridium perfringens* (from fish intestines; 99% identity) and rainbow trout intestinal bacterium T1 (93% identity) all were found in the V6 accretion ice sample. Additionally, sequences closest to *Mobilicoccus pelagius* (a fish intestinal bacterium; 99% identity), *Macrococcus* sp. (associated with marine bivalve larvae; 97% identity), *Pseudomonas xanthomarina* (found in sea squirts; 99% identity), *Curvibacter* sp. (symbiont of *Hydra magnipapillata*; 99% identity), *Mycobacterium marinum* (associated with fish; 99% identity), *Rhodotorula lambellibrachiae* (a basidiomycete that grows in marine tubeworms; 99% identity), *Botrydiopsis constricta* (a heterokont that grows on Antarctic seaweed; 99% identity), and an uncultured sponge symbiont (98% identity) were present in the V5 sample. All of these species depend on intimate associations (symbiotic or parasitic) with their eukaryotic hosts, which are crustaceans, annelids, fish, and other animals. Additional indications of animals in the lake came from the presence of several sequences from members of the Enterobacteriaceae, which were present in both the V5 and V6 samples. These included sequences of several strains/species of *E. coli*, *Erwinia*, *Klebsiella*, *Salmonella*, and *Shigella* (identities ranged from 85–100%), all of which are found in the digestive systems of fish and other aquatic and marine animals. In addition, sequences closest to Fusobacteria that are parasitic on animals (87–99% identities), Gammaproteobacteria that are animal symbionts (≥97% identity), and Tenericutes that are arthropod symbionts and pathogens (identities ranged from 88–91%) were found in the V5 sample.

Sequences from 15 single-celled eukaryotic species were present in V5. These included sequences from members of Excavata (closest to *Trypanosoma cruzi*, 100% identity), Rhizaria (closest to *Paulinella* sp., a freshwater phototroph, 94% identity), Amoebozoa (closest to *Naegleria gruberei*, 98% identity; and *Nolandella* sp., marine, 88% identity) and Chromalveolata (12 unique sequences, including two Ciliophora [*Sterkiella histriomuscorum*, 93% identity; and *Uroleptus pisces*, 99% identity], three bacillariophytes [*Hantschia* sp., 95% identity; and two *Stephanodiscus* spp., 99% and 100% identities], three heterokonts [*Aphanomyces euteiches*, 98% identity; *Botrydiopsis constricta*, 99% identity; and *Halosiphon tomentosus*, 97% identity], a cryptophyte [*Cryptomonas paramecium*, 100% identity] and a member of Perkinsea [obligate parasite of mollusks, 100% identity]). Seventy Archaeplastida sequences were found in V5 and V6 (82–100% identity), of which 16 in V5 and 2 in V6 were at the ≥99% identity level.

### Habitats and Growth Conditions

The V5 sample contained sequences that matched (≥97% and ≥99% identity) those from organisms originating from a variety of habitats, including soil, sediment, aquatic, marine, animal-associated, plant-associated, ice, snow and glaciers (Figure 2). It should be noted that many of the species that have been described as animal-associated and plant-associated have also been found in soil, water and other environments. The proportions of sequences from aquatic species are nearly constant in both samples, while the proportions from marine species are reduced in the V6 sample. The proportion of sequences closest to known psychrophilic and psychrotolerant species was 31% in V5 and 60% in V6 (both at the ≥99% identity level; Figure 2). Within the Gammaproteobacteria alone, there were 33 unique sequences closest to various *Psychrobacter* species (90–100% identities), all known psychrophiles. Also present were psychrophilic or psychrotolerant members of Actinobacteria, Alphaproteobacteria, Archaea, Archaeplastida, Bacteroidetes, Betaproteobacteria, Firmicutes, Chromalveolata and Opisthokonta (both Animalia and Fungi). The proportion of sequences closest to those from thermophiles was approximately one-third in V5 (Figure 2), but was lower in V6, comprising from 13–15% of the sequences (≥97 and ≥99% identity). There were at least 35 sequences that were closest to those from thermophilic organisms (≥99% identity). While most were found in the V5 ice, three were found in the V6 ice. Sequences closest to alkaliphilic/alkalitolerant and acidophilic/acidotolerant taxa were found only in V5 (Figure 2).

The proportions of sequences closest to those from halophilic/halotolerant taxa were approximately equal in V5 and V6 (13–20%, ≥97% identity; Figure 2). Several sequences from marine organisms and halophiles also were present in the data set. *Jeotgalicoccus halotolerans* (98% identity), *Nesterenkonia halotolerans* (93% identity), and other halophilic bacteria (91–100% identities) were found in V5 accretion ice. Additionally, a number of sequences in V5 and V6 were most similar to marine organisms, including marine bacteria (90–100% identities), a marine hydrothermal vent fungus (99% identity), a sea anemone (78% identity) and a marine mollusk (100% identity).

### Metabolic Classification

**Figure 3:**
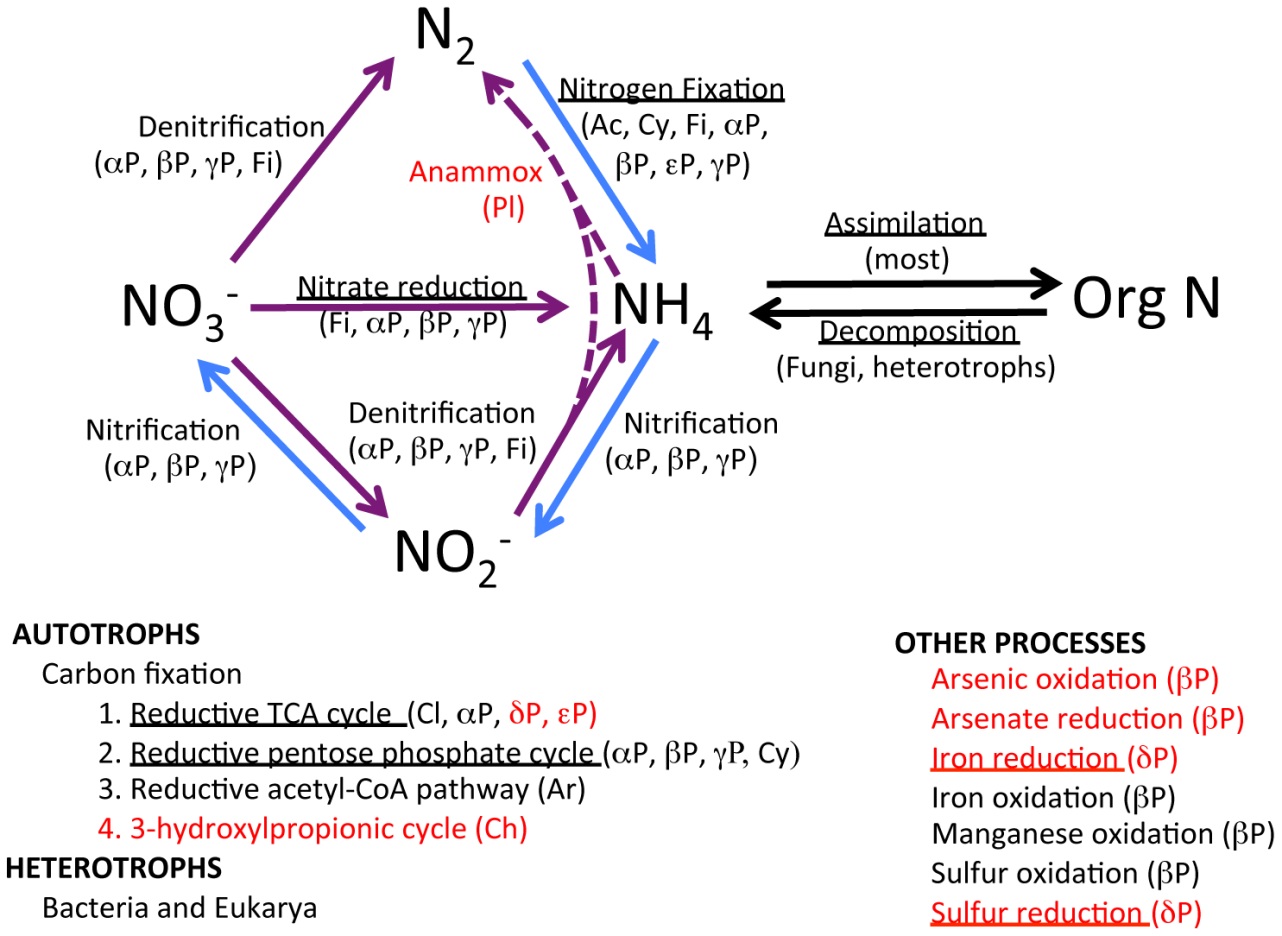
Summary of steps in nitrogen metabolism (above) indicated from the metagenomic/metatranscriptomic sequence identities, as well as types of carbon fixation (lower left) and other functions (lower right) indicated by the sequence data. Each of the pathways was indicated by species determinations that were represented in the metagenomic and metatranscriptomic data sets. Processes also supported by mRNA gene sequences encoding some of the enzymes in the pathways (Tables S11, S12, S13) are underlined. Phyla that include the species identified are provided (in parentheses). Blue arrows represent process that occur under aerobic conditions, while purple arrows indicate anaerobic processes. Carbon fixation pathways are listed below, with taxonomic affinities for each. A large number of sequences closest to those from heterotrophic bacteria and eukaryotes were found in the accretion ice. Other notable metabolic types are listed at the lower right. Abbreviations: Greek alpha = Alphaproteobacteria, Greek beta = Betaproteobacteria, Greek delta = Deltaproteobacteria, Greek epsilon = Epsilonproteobacteria, Greek gamma = Gammaproteobacteria, Ac = Actinobacteria, Ar = Archaea; Cl = Chlorobi; Ch = Chloroflexi, Cy = Cyanobacteria, Fi = Firmicutes, Pl = Planctomycetes. Pathways and taxa in black font denote sequences that exhibited sequence identities between 97 and 100% to sequences in the NCBI nucleotide database. Red font indicates support for sequence identities less than 97%. Examples of species and strains that accomplish each of the pathways are as follows [Species names and accession numbers (in parentheses) for sequences that were of highest identity (≥97% identity, except for *Kuenenia stuttgartienssis* and an uncultured *Nitrosomonas* sp., which exhibited 90% identity to the query sequence) the metagenomic/metatranscriptomic query sequences are presented.]: **Nitrogen fixation** – *Anabena azoica* (Cy; (GI21388238), *Bradyrhizobium* sp. ORS 278 (Gαμμαπροτεοβαχτερια, GI146189981), *Bradyrhizobium* sp. BTAi1 (Alphαπροτεοβαχτερια, GI146403799), *Campylobacter concisus* (Epsilonπροτεοβαχτερια, GI290759912), *Corynebacterium duram* (Ac, GI290759824), *Frankia alni* (Ac, GI111147037), *Geobacillus kaustophilus* (Fi, GI134290402), *Halomonas* sp. GS 1-2 (Gαμμαπροτεοβαχτερια, GI285027202), *Herbaspirillum* sp. B601 (Betαπροτεοβαχτερια, GI62183809), *Leptolyngbya boryana* (Cy, GI46409901), *Mesorhizobium loti* (Alphαπροτεοβαχτερια, GI29725918), *Nocardioides* sp. Cr7-14 (Ac, GI293629578), *Nostoc muscorum* (Cy, GI29124940), *Nostoc punctiforme* (Cy, GI186463002), *Phicicola gilvus* (Ac, GI111146878), *Phormidium autumnale* (Cy, GI166997748), *Rhodobacter changlensis* (Alphαπροτεοβαχτερια, GI125656032), *Synechococcus* sp. C9 (Cy, GI90186509); **Nitrification** – *Bradyrhizobium* sp. BTAi1 (Alphαπροτεοβαχτερια, GI146403799), *Denitrobacter* sp. BBTR53 (Betαπροτεοβαχτερια, GI85002019), *Herbaspirillum* sp. B601 (Betαπροτεοβαχτερια, GI62183809), uncultured *Nitrosomonas* sp. (Alphαπροτεοβαχτερια, GI223036385); **Denitrification** – *Bacillus cereus* (Fi, GI269994025), *Brevudomonas* sp. V3M6 (Alphαπροτεοβαχτερια, GI295809779), *Caulobacter* sp. can1 (Alphαπροτεοβαχτερια, GI288908581), *Geobacillus kaustophilus* (Fi, GI134290402), *Paracoccus* sp. YT0095 (Alphαπροτεοβαχτερια, GI158392748), *Pseudomonas xanthamarina* (Gαμμαπροτεοβαχτερια, GI254621816), *Psychrobacter maritimus* (Gαμμαπροτεοβαχτερια, GI240129723), uncultured Commomonadaceae sp. (Betαπροτεοβαχτερια, GI184189965); **Nitrate reduction** – *Bacillus cereus* (Fi, GI294999187), *Delftia acidovorans* (Betαπροτεοβαχτερια, GI213536827), *Paracoccus yeei* (Alphαπροτεοβαχτερια, GI206581410), uncultured *Citrobacter* sp. (Gαμμαπροτεοβαχτερια, GI257073647), **Anammox** – *Keunenia stuttgartiensis* (Pl; GI91199943). Support from mRNA gene sequences for specific processes is presented in Table S13. Pathways with rRNA and mRNA gene sequence support are underlined.

**Figure 4:**
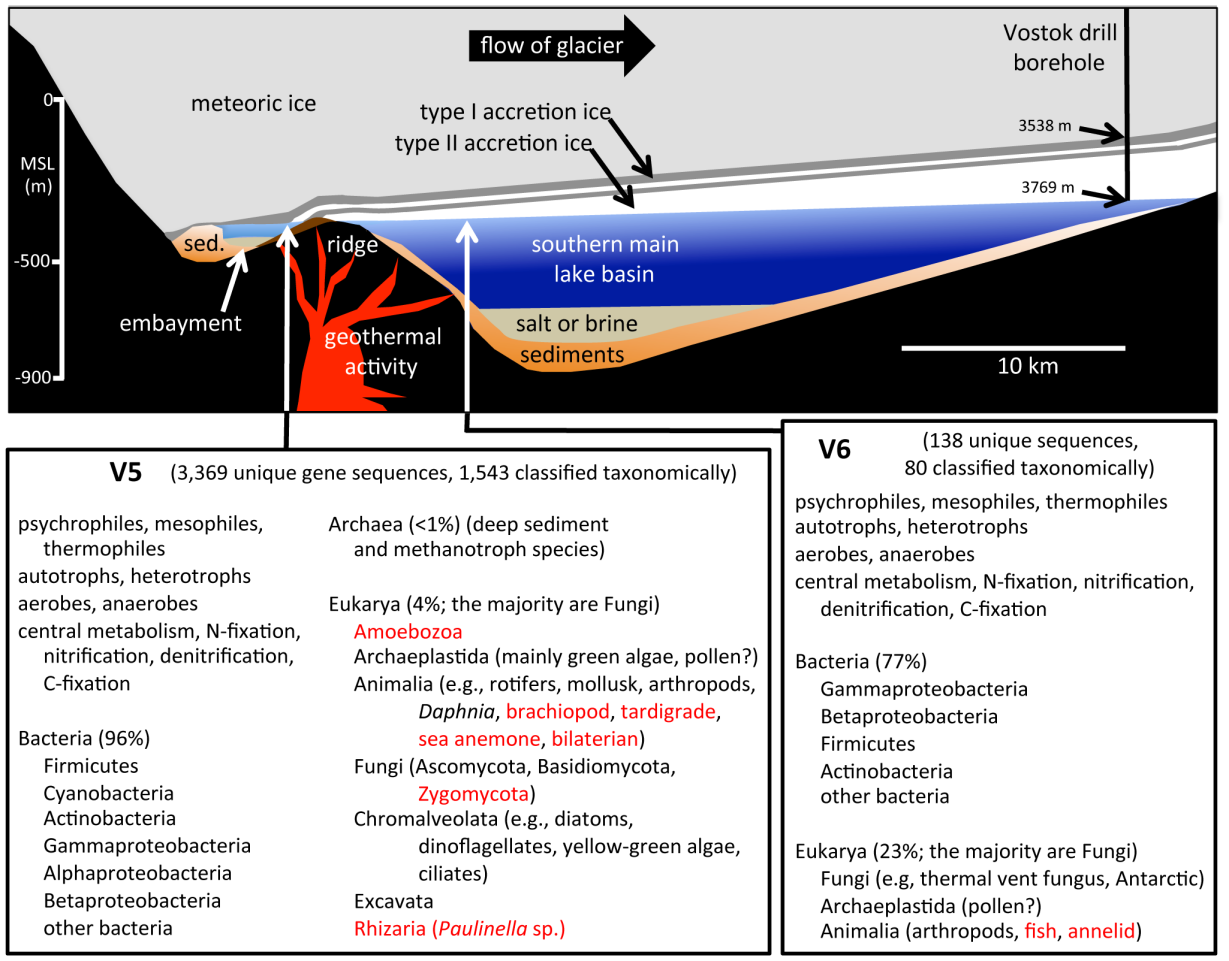
Schematic cross-section of Lake Vostok (above), drawn to scale (based on a radar study of Lake Vostok along the glacial flow line to the ice core drill site [**2**]) and metagenomic/metatranscriptomic summary (below). The overlying glacier (meteoric ice – light gray) is 3538 m thick at the Vostok drill site (right). At that depth, the ice is estimated to be approximately 1 to 2 million years old [53]. Organisms and biological molecules entrapped in the meteoric ice are deposited in the lake due to breakage and melting of the ice as it flows into the lake (left) [2], [8]. The transit time for the glacier to move across the lake is approximately 15,000 to 20,000 years [2], [8], [12]. As the glacier moves over the lake, water at its surface freezes (accretes) onto the bottom of the glacier. The uppermost regions of the accretion ice represent lake water from the vicinity of the embayment followed by ice accreting near a ridge (or peninsula), and then ice accreting over the southern main lake basin. Accretion ice closest to the bottom of the meteoric ice (3538–3539 m at the drill site) is approximately 10,000 years old, while ice closest to the lake surface (3769 m at the drill site) has accreted recently. The microbes in this study originated from core sections that represent water from the vicinity of the embayment (V5, approximate location indicated by arrow) and a section of the southern main lake basin (V6, approximate location indicated by arrow). Locations of the possible hydrothermal source (red), sediment depths (orange), and extent of saltwater layers (tan) are hypothetical. Type I ice is indicated in dark gray, while type II ice is white. Lower portions of the figure summarize the types of organisms and metabolic functions indicated by sequences found in each of the samples, based on the metagenomic/metatranscriptomic analyses (complete data set used). Red font indicates organisms whose sequence identities were <97% and/or were deduced from sequence identification of organisms normally associated with those organisms.

Determinations of metabolic processes were based on species determinations from rRNA sequences at identities ≥97%, and from high identities to mRNA sequences (Figure 3; Tables S11, S12, S13; Figures S1, S2, S3, S4). The central metabolic pathways (e.g. glycolysis, ATP synthesis, TCA cycle, nucleic acid synthesis and amino acid synthesis) were well represented in the data sets. Sequences that were closest to species involved in many phases of the nitrogen cycle and carbon fixation pathways were found (Figures 3 and 4). In addition, mRNA sequences for many of the enzymes in these processes were found (Figure 3; Tables S11, S12, S13; Figures S1, S2, S3, S4, S5). This was true whether the entire data set was used or the subsets at the ≥97% or ≥99% identity levels were used. Many sequences closest to many types of nitrogen fixing bacteria were found, including many sequences closest to species of *Azospirillum, Azotobacter*, *Bacillus, Burkholderia,* Cyanobacteria, *Frankia*, *Klebsiella, Rhizobium*, *Rhodobacter, Rhodopseudomonas* and *Sinorhizobium* (Figure 3; Tables S1, S2, S3, S4, S5, S6, S7, S8, S9, S10). Sequences of nitrifying bacteria included members of the genera *Methylococcus, Nitrobacter, Nitrococcus, Nitrosococcus* and *Nitrosomonas* (Figure 3.; Tables S1, S2, S3, S4, S5, S6, S7, S8, S9, S10). Sequences found from other genera that are important in the nitrogen cycle were *Alkaligenes, Bacillus*, *Clostridium*, *Micrococcus*, *Paracoccus*, *Proteus*, *Pseudomonas*, *Streptomyces* and *Thiobacter*. Sequences closest to those from planctomycetes (91–100% identities), including marine species capable of anammox metabolic processes, were present (Figures 3 and 4; Tables S1, S2, S3, S4, S5, S6, S7, S8, S9, S10).

At least three forms of carbon fixation were indicated (Figure 3; Tables S11, S12, S13; Figures S1, S2, S3, S4, S5). Most of the sequences were closest to the metagenomic/metatranscriptomic sequences utilize either the reductive TCA (rTCA) cycle, the reductive pentose phosphate cycle (rPP; Calvin-Benson) or the reductive acetyl-CoA (rACA) pathway. The most common (based on number of unique sequences; 77% overall; 79% at ≥97% identity; 81% at ≥99% identity) was the rPP cycle (in Alphaproteobacteria, Archaeplastida, Betaproteobacteria, Chromalveolates, Cyanobacteria, and Gammaproteobacteria). The second most common was the rTCA cycle (in Alphaproteobacteria, Bacterioidetes, Deltaproteobacteria, and Epsilonproteobacteria), and the third most common was the rACA pathway (in the two Archaea; <1% at all levels of identity) [27]. However, mRNA gene sequences for this last pathway were not found in searches of the metagenome/metatranscriptome data set. One sequence that was closest to a species of Chloroflexi was found. Some members of this taxon fix carbon via the 3-hydroxypropionic cycle, although some also use the rPP cycle [27].

A large number and diversity of sequences closest to sequences from phototrophs were present in the accretion ice, including 181 cyanobacterial (80 and 53 at the ≥97% and ≥99% identity levels, respectively), 11 algal (6 and 3 at the ≥97% and ≥99% identity levels, respectively), 11 chromalveolate (8 at the ≥99% identity level), and other sequences (primarily in V5). Sequences for many of the genes involved in the light reactions of photosynthesis in cyanobacteria were found in the V5 sample. Also, sequences for light-independent protochlorophyllide reductase and oxidase, phycocyanobilin oxidoreductase, a phycoerythrin subunit and several genes involved in carotenoid biosynthesis were found.

Two sequences from the V5 were moderately similar to those from arsenic oxidizing bacteria (*Thiomonas* spp., 81–95% identities; and *Herminiimonas arsenicoxydans,* 90% identity; Figure 3). One of the major sources of arsenic on Earth is from volcanic emissions. Both species can oxidize arsenic, but can also oxidize sulfur and fix carbon from CO_2_. A number of sequences closest to those from sulfur oxidizing bacteria, as well as from thermophilic sulfur reducing bacteria, were found in V5 (Figure 2).

## Discussion

Although Lake Vostok is oligotrophic, based on the metagenomic and metatranscriptomic results presented here, it is far from sterile. Overall, 3,507 unique sequences were found in the accretion ice, presumably representing the same number of unique species or strains (Figures 1 and 4). At higher stringency, there were 1,215 unique sequences that were closest to known sequences in the NCBI nucleotide database at identity levels of ≥99% (Figure 1). Over 96% (1,168) of these were found in the V5 (3563 m +3585 m) type I accretion ice samples from the vicinity of the shallow embayment on the southwest end of the lake. Of these, 95% (1,121 at ≥99% identity) were Bacteria, 5% were Eukarya (64), and 2 were Archaea. In contrast, only 138 unique sequences were obtained from the deeper V6 (3606 m +3621 m) type II accretion ice sample, which accreted over the southern main basin. At higher stringency (≥99% identity), there were 47 unique sequences, of which 77% (36) were Bacteria and the remainder (11) were Eukarya (Figure 1), the majority of which were Fungi. The reduced number of sequences was expected, based on previously reported cell counts, cultivation and sequencing from the same ice core sections [14], [15]. Firmicutes, Actinobacteria, Cyanobacteria and Proteobacteria were the most numerous (38–39%, combined), while sequences from Ascomycota and Basidiomycota were among the most numerous eukaryotic sequences (38–50%, combined), which is also consistent with our previous reports [14], [15].

The metagenomic/metatranscriptomic sequences suggest that a complex environment might exist in Lake Vostok. Sequences indicating organisms from aquatic, marine, sediment and icy environments were present in the accretion ice (Figure 2). In addition, another major proportion of the sequences was from organisms that are symbionts (commensals, mutualists, and pathogens) of animals and/or plants. Many of these have also been isolated from soil and water samples, and often they live opportunistically on other organisms. From 15 to 20% of the sequences in each sample are closest to sequences from aquatic organisms, while approximately 7% of the sequences are closest to sequences from marine organisms. The presence of sequences from marine organisms might be explained by the presence of saltwater, brackish water or brine layers in the lake. Previous studies have reported relatively high concentrations of Mg^2+^, Na^+^, Cl^−^ and SO_4_^2−^ in ice core sections adjacent to those used for the V5 sample (3563 and 3585 m) in this research [10]–[12]. The molecular signatures from marine species suggest a possible connection to the surrounding oceans sometime in the past for Lake Vostok, which currently lies entirely below sea level (Figure 4) [2], [28]. The levels of these ions are greatly reduced in ice core sections adjacent to those used for the V6 sample (3606 and 3621 m), and no sequences similar to marine organisms at ≥99% identity were found in the V6 sample (although there were a few at the ≥97% identity level).

Only a small number of sequences appear to originate from organisms that inhabit ice, snow and glaciers. However, psychrophiles and psychrotolerant species are poorly represented in most sequence databases, including the GenBank/NCBI database. A total of 31 sequences in the V5 sample, and three in the V6 sample, were closest to (≥99% identity) those from psychrophiles and psychrotolerant organisms (Figure 2, lower row). This would be expected of a lake that probably is close to the freezing point near its surface. A large number of sequences were closest to those from organisms found in soils and sediments, suggesting that water from depth might be reaching the surface of the lake. However, this supposition must remain tentative until the lake is explored directly.

Many sequences were closest (at ≥99% identity) to those from thermophilic and thermotolerant organisms. Some studies have postulated the presence of hydrothermal activity in Lake Vostok [4], [12]. A total of 35 sequences similar to those from known thermophilic/thermotolerant bacterial species at highest stringency (≥99% identity), and 51 using lower stringency (≥97% identity), were found. Only 3 of them were from the V6 sample. The number of thermophilic species supports the suggestion that hydrothermal activity might exist in Lake Vostok. The lake lies in a graben within a rift valley that began to form more than 35 million years ago [29], prior to ice formation over the lake. Volcanic activity and other geothermal features are usually associated with rift valleys. Because the sequences that are closest to those from known thermophilic and thermotolerant species are almost all from the V5 sample, thermal areas in the lake appear to be closer to the southwestern corner of the lake, in the vicinity of the embayment. Two sequences indicated the presence of arsenic oxidizing bacteria (*Thiomonas* sp. and *Herminiimonas arsenicoxydans*), which also metabolize sulfur. Arsenic is commonly present in volcanic emissions, also suggesting that thermal features may exist in the lake. Importantly, the hydrothermal vents could provide sources of energy and nutrients vital for organisms living in the lake. Two of the sequences in V6 were closest to species of *Thiobacillus*, which is within the same family (Hydrogenophilaceae) as a sequence from a thermophile (*Hydrogenophilus thermoluteus*) that was previously reported from the 3607 m section of the Vostok 5G ice core [30], [31]. The two samples were within a meter of one another in the ice cores, and therefore, they may have accreted from lake water only a few meters apart, and temporally spaced by a few years.

A set of sequences was closest to those from species and genes that carry out various parts of the nitrogen cycle (Figure 3; Tables S1, S2, S3, S4, S5, S6, S7, S8, S9, S10, S11, S12, S13), including nitrogen fixation (Actinobacteria, Cyanobacteria, Betaproteobacteria and Gammaproteobacteria), nitrification (Alphaproteobacteria and Betaproteobacteria), denitrification (Gammaproteobacteria), nitrate reduction (Betaproteobacteria and Gammaproteobacteria) anammox (planctomycetes), assimilation (most) and decomposition (fungi and other heterotrophs). Nitrogen gas is delivered to the lake by release of atmospheric gases as the glacial ice melts into the lake. Therefore, the supply of nitrogen to the lake probably is adequate for life in the lake. At least three modes of carbon fixation were inferred (Figure 3), based on species and mRNA gene sequences. The most common mode of carbon fixation (based on the number of species represented by unique sequences) was the reductive pentose phosphate cycle (rPP; i.e., Calvin-Benson cycle) [27]. Sequences representing the presence of members of the Alphaproteobacteria, Betaproteobacteria, Gammaproteobactera and Cyanobacteria that use this mode of carbon fixation were present in the ice core samples. However, the cyanobacteria probably are functioning as heterotrophs, because no light reaches the lake. The second most common was the reductive TCA (tricarboxylic acid) pathway, present in members of Chlorobi, Alphaproteobacteria, Deltaproteobacteria and Epsilonproteobacteria [27]. Some of the Deltaproteobacteria and Epsilonproteobacteria sequences exhibited less than 97% identity to known sequences, but the Chlorobi and Alphaproteobacteria sequences exhibited identities ≥97% to known sequences. This cycle is common in organisms living near hydrothermal vents, but many psychrophilic and mesophilic organisms also fix carbon using this cycle. The rTCA cycle also produces precursors for nucleic acid and aromatic amino acid syntheses. A third type of carbon fixation, the reductive acetyl-CoA pathway, is used by Archaea. While sequences matching those from two deep-sediment Archaea were found in V5, gene sequences for enzymes of this pathway were not found among the metagenomic/metatranscriptomic sequences, and therefore, it is questionable whether it exists in the lake. All three carbon fixation pathways use CO_2_ as the carbon source, and use NADH (or NADPH) as the electron donor, but the rTCA cycle and rACA pathway also can utilize ferredoxin pairs as electron donors. The rPP cycle requires 3.5 times as much ATP per fixed carbon as the rTCA cycle, and over 7 times as much as the rACA pathway [27]. A fourth mode of carbon fixation, the 3-hydroxypropionic cycle is used by members of Chloroflexi, one member of which was among the sequences (99% identity to an uncultured Chloroflexi). However, no gene sequences specific to this pathway were found. Some members of this phylum also use the rPP cycle, and therefore it is unclear which mode of carbon fixation is used by this species [27].

Many sequences from multicellular organisms were found (Figures 2 and 4), including 51 in V5 and 8 in V6 (≥99% identity level). Fungal sequences were the most frequently found (34 in V5 and 7 in V6). However, sequences from a diversity of taxonomic groups were present, including sequences closest to bivalves, arthropods and rotifers (Figures 2 and 4). A large number of bacterial sequences from animal commensals, mutualists and pathogens were present, including those associated with annelids, sea anemones, brachiopods, tardigrades and fish. While it is impossible at this time to conclude that these animals live in Lake Vostok, there also were dozens of sequences from species of animal-associated bacteria (e.g., many sequences that closely match those from members of the Enterobacteriaceae and several from members of the Fusobacteria). Additionally, there were a few sequences that were closest to species of animals (e.g., *Daphnia* sp. [planktonic crustacean; 98% identity], *Adineta* sp., [a rotifer, which is a hardy, cosmopolitan, freshwater species; 98% identity], and *Nutricola tantilla* [a marine bivalve; 100% identity]). This leads to the tentative conclusion that at least some complex animals might be present in the lake.

Dozens of sequences closest to those from members of the Archaeplastida (Chlorophyta, Rhodophyta and Streptophyta) were found in the accretion ice. These could either originate from the lake or might have been deposited from the glacial ice. Some plants buried in permafrost can remain viable for more than 30,000 years [32], and viable bacteria and fungi have been cultivated from ice and permafrost specimens, some of which were millions of years old [14], [15], [20], [33], [34]. Some nucleic acids can be detected and sequenced more than 100 million years after the organisms containing them have perished [14], [15], [20]. Therefore, it is possible that some of the DNAs that were detected were from long-dead organisms, possibly having been deposited prior to the lake being isolated by ice cover, or being delivered by the overriding glacial ice. However, RNA is less stable than DNA, and therefore, it would not be expected to remain intact for millions of years unless the organisms are metabolically active. The RNA that was sequenced in this study likely was from living organisms present in the accretion ice.

A large diverse set of organisms survive or thrive in extreme environments. Many species of bacteria, as well as a number of unicellular and multicellular eukaryotes (including opisthokonts) have been found inhabiting deep-sea hydrothermal vents [35]–[42], exposed to heat, cold, chemical gradients and high pressures [43]–[49]. Therefore, finding a large number of sequences from these taxa in Lake Vostok is expected. Sterility is decidedly unexpected. Previously, we microscopically observed and cultivated Bacteria and Fungi from some of the same Lake Vostok accretion ice core sections [14], [15]. They were identified by sequencing rRNA gene loci, followed by phylogenetic analyses. The Bacteria and Fungi from our cultivation studies comprised a subset of those found in our metagenomic data set, further confirming the presence of these organisms (Table S16). Sequences that indicated the presence of annelids, crustaceans, mollusks, sea anemones and fish also are consistent with conditions in the lake. Annelids have been found living near hydrothermal vents, as well as in deep-marine sediments [38]. Sea anemones have been found in deep-ocean locations, as well as near underwater volcanoes [39]. Species of fish, crustaceans and mollusks that live near hydrothermal vents and in deep-sea environments have also been described [40]–[42], [49], and a number of bacterial sequences were closest to taxa that are common symbionts and commensals of animal digestive and excretory organs. While some were specific to fish and mollusks, others have been found in a variety of animals, suggesting the possibility that other Animalia species might exist in the lake.

Over 35 million years ago, Lake Vostok was open to the atmosphere and was surrounded by a forested ecosystem [50], [51]. At that time, the lake (which might have been a marine bay [28]) probably contained a complex network of organisms. As recently as 15 million years ago, portions of the lake were ice-free at least part of the time [50], [51]. During these times, organisms were likely being deposited in the lake through atmospheric transport (i.e., wind and precipitation). Thus, during its history, there were many opportunities for organisms to enter and populate the lake. While the current conditions are different than earlier in its history, the lake seems to have maintained a surprisingly diverse community of organisms. These organisms may have slowly adapted to the changing conditions in Lake Vostok during the past 15–35 million years as the lake converted from a terrestrial system to a subglacial system.

### Cell Concentrations and Contamination Considerations

The issue of the potential for external contamination in ice is important, because of the low concentrations of cells that have been previously reported, and because there have been suggestions that the lake is sterile or nearly so [13]. In our research, all ice core sections were treated to assure the elimination of external contaminating organisms and nucleic acids [20], [21]. The ice core sections each were treated with 5.25% sodium hypochlorite (Clorox) for 10 seconds, then washed with an excess of autoclaved reverse osmosis (Nanopure) water prior to melting. This assured removal of external contaminating organisms and nucleic acids. After melting in a sterile hood, the meltwater was subjected to ultracentrifugation and processing for sequence determination (details in methods section). Our results indicate that the majority of sequences that were determined from the V5 and V6 samples likely originated from the accretion ice (and therefore from the lake). BLAST E-value cutoffs of 10^−10^ (for rRNA and mRNA sequences) were used to assure high stringency in sequence similarity searches. The same stringency was used to search through the sequences that resulted from the water controls. These controls yielded lower numbers of unique sequences. Approximately 100 species were indicated from the sequences in the controls. More than 59% of the sequences were from one species/strain, *Propionibacterium acnes* KPA1717202, common on human skin. Over 80% of the sequences represented just 5 species: *P. acnes*, *E. coli* K12, *Homo sapiens*, *Pseudomonas fluorescens* and *Bos taurus*. The remainder were generally single occurrences of common species, including some that might originate in the PCR and/or cDNA reagents. Sequences similar to these at the species level were removed from the V5 and V6 data sets prior to evaluation of the results (Tables S14 and S15). Because of the high number of PCR amplification cycles, contaminant sequences were expected. When nucleic acid template concentrations are low, or the templates are damaged, often additional cycles are needed to amplify the desired sequences. However, minor contaminants in the samples, introduced in the laboratory, or originating with the reagents used to manipulate the nucleic acids cannot be avoided completely, but can only be minimized [52]. Specifically, contaminating sequences from humans have been amplified from PCR reagents. Sequences from bovines, swine and chickens have been amplified by PCR reactions containing gelatin (which is produced from animal byproducts). Gelatin was a component of the PCR reagents used in this study, and therefore it is likely that the *Bos taurus* sequences, and possibly others, originated from the reaction components.

Notwithstanding the concerns above, from a practical standpoint, it is difficult to imagine how the majority of the sequences determined in this study were the result of contamination, given the number, diversity and physiological variety of the organisms indicated by the sequences that remained after subtraction of those from the controls. The number of unique rRNA gene sequences in the accretion ice core sections was from 138 (in V6) to 3,369 (in V5). In previous studies of accretion ice core sections, ice from 3563 to 3590 m (i.e., in the vicinity of the embayment) yielded higher cell counts, more cultured isolates and more sequence diversity than ice from 3606 to 3621 m (i.e., within the main lake basin) [14], [15]. The accretion ice sequences presented here from V5 and V6 yielded high identity BLAST matches to a wide range of organisms, including: thermophiles, psychrophiles, mesophiles, psychrotolerant species, themotolerant species, peizophiles, peizotolerant species, heterokonts, opisthokonts, amoebae, ciliated protozoans, excavates, rhizaria, mollusks, a deep sediment bilaterian, fish-associated bacteria, annelid symbionts, crustacean symbionts, arsenic oxidizers, sulfur oxidizers, sulfur reducers, deep sediment organisms, lake/ocean sediment microbes, aquatic microbes, limestone associated organisms, *Deinococcus*, tardigrades, rotifers, diatoms, dinoflagellates, polar organisms (mainly from Antarctica), yellow-green algae, green algae, red algae, a deep sediment Archaea, a methanotrophic Archaea, a diverse group of fungi, nitrogen fixing organisms, carbon fixing organisms, aerobes and anaerobes. Together, these are consistent with life in a deep, cold subglacial lake environment that also includes hydrothermal activity. The metabolic pathways that were deduced from the sequence data are consistent with a large oligotrophic lake ecosystem that includes melting ice, aerobic, anaerobic, hydrothermal, freshwater and saline zones.

The taxonomic classifications based on the metagenomic/metatranscriptomic sequences were compared with the taxonomic and phylogenetic determinations from our previous culture and sequencing results [14], [15]. Identical or highly similar sequences were found in all cases, primarily matching at the genus and/or species levels (Table S16). Also, as mentioned previously, one thermophile in V6 was similar to the *H. thermoluteus* that was previously reported [31], [31] from an ice core section that is less than one meter away, translating into adjacent regions of the lake surface separated by less than 200 m of lake water, and by less than a decade or two in time.

These metagenomic/metatranscriptomic results are consistent with results from other reports on Lake Vostok ice [8]–[19], [28]. From our previous studies, the cell concentrations for core sections corresponding to those in V5 and V6 ranged from <1 to 35 cells ml^−1^ (based on fluorescent microscopy of concentrated 10 ml aliquots). The core sections corresponding to V6 had lower cell counts compared to those corresponding to V5. The mean values were from 2.33 to 12.33 cells ml^−1^. Ranges from other studies have been from <1 to several hundred cells ml^−1^ [13], [20], [21], [30], [33], [34], [45]. These concentrations correspond well with the results of this metagenomic/metatranscriptomic study.

From the metagenomic/metatranscriptomic data, we calculated that there exist at least 14 unique sequences ml^−1^ in V5 ice meltwater (for the 250 ml sample), and at least 0.7 unique sequences ml^−1^ in V6 meltwater (for the 250 ml sample). By extension, there are at least the same number of organisms in the Lake Vostok surface water. Although these are low values for an aquatic sample, the metagenomic/metatranscriptomic sequence data are consistent with previously reported cell and sequence concentrations for adjacent accretion ice core sections. All indications are that Lake Vostok is oligotrophic, but that it contains a diverse assemblage of organisms, including complex multicellular eukaryotes, most of which are in the vicinity of the embayment.

### Conclusions

Lake Vostok accretion ice contains nucleic acids from a diverse set of organisms, including sequences from anaerobic, aerobic, psychrophilic, thermophilic, halophilic, alkaliphilic, acidophilic, aquatic, marine and sediment-inhabiting species. The list of taxa consists of approximately 94% Bacteria and 6% Eukarya, including over 100 sequences from multicellular Eukarya. The sequences represent members of species that participate in many phases of the nitrogen cycle, as well as those that fix, utilize and recycle carbon. The higher concentrations of microbes in accretion ice compared to the overriding meteoric ice, and the presence of RNA, suggest that viable organisms exist in the lake water. Therefore, Lake Vostok might contain a complex web of organisms, zones and habitats that have developed over the tens of millions of years of its existence.

## Supporting Information

Figure S1 **Global map of metabolic pathways represented in the data set, based on results from KAAS KEGG analyses.** Color key for the metabolic pathways are indicated at the lower right. Solid black lines indicate pathways within a metabolic process. Dashed red lines indicate connections among the pathways. Dashed black lines indicate pathways not represented in the sequence database.
(TIF)

Figure S2 **Tricarboxylic Acid (TCA) cycle, and adjoining metabolic processes found in the sequence data set.** The TCA cycle in most organisms proceeds in a clockwise direction, in an oxidative process. However, many genes and organisms found in the data set operate the TCA cycle in the reverse direction (counterclockwise, termed the rTCA cycle) in a reductive process to fix CO_2_. In the oxidative direction, NADH and ATP are produced, which can be used in other metabolic processes. In the reverse direction, NADH and ATP were required to fix CO_2_ into organic compounds. Line colors and styles are as in Figure S1.
(TIF)

Figure S3 **Details of connections between the TCA cycle (from** **Figure 2** **) and oxidative phosphorylation, based on sequences found in the data set.** Line colors and styles are as in Figure S1.
(TIF)

Figure S4 **Reductive pentose phosphate (rPP) cycle and adjoining metabolic processes found in the data set.** Many microbes fix CO_2_ using this pathway, including cyanobacteria and chloroplasts. Line colors and styles are as in Figure S1.
(TIF)

Figure S5 **Additional reactions and pathways represented in the sequence data base.** Line colors and styles are as in Figure S1.
(TIF)

Table S1 **Small subunit rRNA gene sequences of Bacteria and Eukarya from V5.** [“n” indicates information not specified in the NCBI GenBank database.].
(PDF)

Table S2 **Large subunit rRNA gene sequences of Bacteria and Eukarya from V5.** [“n” indicates information not specified in the NCBI GenBank database.].
(PDF)

Table S3 **Ribosomal RNA gene sequences less than 200 nt in length (could not be submitted to NCBI) from V5.** [“n” indicates information not specified in the NCBI GenBank database.].
(PDF)

Table S4 **Bacteria mRNA (and other non-rRNA) gene sequences from V5.** [“n” indicates information not specified in the NCBI GenBank database.].
(PDF)

Table S5 **Eukarya mRNA (and other non-rRNA) gene sequences from V5.** [“n” indicates information not specified in the NCBI GenBank database.].
(PDF)

Table S6 **Sequences from Archaea and Viruses in V5.** [“n” indicates information not specified in the NCBI GenBank database.].
(PDF)

Table S7 **Small subunit rRNA gene sequences of Bacteria and Eukarya from V6.** [“n” indicates information not specified in the NCBI GenBank database.].
(PDF)

Table S8 **Large subunit rRNA gene sequences of Bacteria and Eukarya from V6.** [“n” indicates information not specified in the NCBI GenBank database.].
(PDF)

Table S9 **Ribosomal RNA gene sequences less than 200 nt in length (could not be submitted to NCBI) from V6.** [“n” indicates information not specified in the NCBI GenBank database.].
(PDF)

Table S10 **Bacteria and Eukarya mRNA (and other non-rRNA) gene sequences from V6.** [“n” indicates information not specified in the NCBI GenBank database.].
(PDF)

Table S11 **Blastn and Blastx results from analysis of V5 sequences on the KAAS KEGG site** [**25**] **.** The searches were for highly similar sequences (megablast); Max. target sequences = 100; Expected threshold = 1e-10 (unless no results were found, then 0); filter low complexity regions and translated nucleotide search over Reference sequence protein database; Matrix - BLOSUM62; Scoring parameters (existence 11; extension 1); filter low complexity regions. [“n” indicates information not specified in the NCBI GenBank database.].
(PDF)

Table S12 **Blastn and Blastx results from analysis of V6 sequences on the KAAS KEGG site** [**25**] **.** The searches were for highly similar sequences (megablast); Max. target sequences = 100; Expected threshold = 1e-10 (unless no results were found, then 0); filter low complexity regions and translated nucleotide search over Reference sequence protein database; Matrix - BLOSUM62; Scoring parameters (existence 11; extension 1); filter low complexity regions. [“n” indicates information not specified in the NCBI GenBank database.].
(PDF)

Table S13 **Gene sequences found that support processes in** **Figure 3** **.**
(PDF)

Table S14 **Sequences removed from the V5 data set that were identical or similar to sequence from controls.** [“n” indicates information not specified in the NCBI GenBank database.].
(PDF)

Table S15 **Sequences removed from the V6 data set that were identical or similar to sequence from controls.** [“n” indicates information not specified in the NCBI GenBank database.].
(PDF)

Table S16 **Comparison of metagenomic/metatranscriptomic sequence results with previous cultivation and sequencing results [refs. 14 and 15].**
(PDF)

## References

[1] Kapista A, Ridley JF, Robin GDeQ, Siegert MJ, Zotikov I (1996) Large deep freshwater lake beneath the ice of central Antarctica. Nature 381: 684–686.

[2] MacGregor JA, Matsuoka K, Studinger M (2009) Radar detection of accreted ice over Lake Vostok, Antarctica. Earth Planet Sci Lett 282: 222–233.

[3] Masalov VN, Lukin VV, Shermetiev AN, Popov SV (2001) Geophysical investigation of the subglacial Lake Vostok in Eastern Antarctica. Doklady Earth Sci 379A: 734–738.

[4] Studinger M, Karner GD, Bell RE, Levin V, Raymond CA, et al (2003) Geophysical models for the tectonic framework of the Lake Vostok region East Antarctica. Earth Planet Sci Lett 216: 663–677.

[5] Wright A, Siegert MJ (2011) The identification and physiographical setting of Antarctic subglacial lakes: An update based on recent discoveries. Geophysical Monograph Ser 192: 9–26.

[6] Wingham D, Siegert M, Shepherd A, Muir AS (2006) Rapid discharge connects Antarctic subglacial lakes. Nature 440: 1033–1036. 16625193 10.1038/nature04660

[7] Jouzel J, Petit JR, Souchez R, Barkov NI, Lipenkov VY, et al (1999) More than 200 meters of lake ice above subglacial Lake Vostok, Antarctica. Science 286: 2138–2141. 10591641 10.1126/science.286.5447.2138

[8] Bell R, Studinger M, Tikku A, Castello JD (2005) Comparative biological analyses of accretion ice from subglacial Lake Vostok. In: Castello JD, Rogers SO, editors. Life in Ancient Ice. Princeton, NJ: Princeton University Press. 251–267.

[9] Gramling C (2012) A tiny window opens into Lake Vostok, while a vast continent awaits. Science 335: 788–789. 22344422 10.1126/science.335.6070.788

[10] Siegert MJ, Ellis-Evans JC, Tranter M, Mayer C, Petit J, et al (2001) Physical, chemical and biological processes in Lake Vostok and other Antarctic subglacial lakes. Nature 414: 603–609. 11740551 10.1038/414603a

[11] Siegert MJ, Tranter M, Ellis-Evans JC, Priscu JC, Lyons WB (2003) The hydrochemistry of Lake Vostok and the potential for life in Antarctic subglacial lakes. Hydrol Process 17: 795–814.

[12] Christner BC, Royston-Bishop G, Foreman CM, Arnold BR, Tranter M, et al (2006) Limnological conditions in subglacial Lake Vostok, Antarctica. Limnol Oceanogr 51: 2485–2501.

[13] Bulat SA, Alekhina IA, Lipenkov VYa, Lukin VV, Marie D, et al (2009) Cell concentrations of microorganisms in glacial and lake ice of the Vostok ice core, East Antarctica. Microbiol 78: 808–810.

[14] D’Elia T, Veerapaneni R, Rogers SO (2008) Isolation of microbes from Lake Vostok accretion ice. Appl Environ Microbiol 74: 4962–4965. 18552196 10.1128/AEM.02501-07 PMC2519340

[15] D’Elia T, Veerapaneni R, Theraisnathan V, Rogers SO (2009) Isolation of fungi from Lake Vostok accretion ice. Mycologia 101: 751–763. 19927741 10.3852/08-184

[16] Abyzov SS, Poglazova MN, Mitskevich JN, Ivanov MV (2005) Common features of microorganisms in ancient layers of the Antarctic ice sheet. In: Castello JD, Rogers SO, editors. Life in Ancient Ice. Princeton, NJ: Princeton University Press. 240–250.

[17] Christner BC, Mosley-Thompson E, Thompson LG, Reeve JN (2001) Isolation of bacteria and 16S rDNAs from Lake Vostok accretion ice. Environm Micrbiol 3: 570–577. 10.1046/j.1462-2920.2001.00226.x 11683867

[18] Karl DM, Bird DF, Björkman K, Houlihan T, Shakelford R, et al (1999) Microorganisms in the accreted ice of Lake Vostok, Antarctica. Science. 286: 2144–2147. 10.1126/science.286.5447.2144 10591643

[19] Priscu JC, Adams EE, Lyons WB, Voytek MA, Mogk DW, et al (1999) Geomicrobiology of subglacial ice above Lake Vostok, Antarctica. Science. 286: 2141–2144. 10.1126/science.286.5447.2141 10591642

[20] Rogers SO, Ma LJ, Zhao Y, Catranis CM, Starmer WT, et al. (2005) Recommendations for elimination of contaminants and authentication of isolates in ancient ice cores. In: Castello JD, Rogers SO, editors. Life in Ancient Ice. Princeton, NJ: Princeton University Press. 5–21.

[21] Rogers SO, Theraisnathan V, Ma LJ, Zhao Y, Zhang G, et al (2004) Comparisons of protocols to decontaminate environmental ice samples for biological and molecular examinations. Appl Environ Microbiol 70: 2540–2544. 15066857 10.1128/AEM.70.4.2540-2544.2004 PMC383132

[22] Chevreux B, Wetter T, Suhai S (1999) Genome Sequence Assembly Using Trace Signals and Additional Sequence Information. Computer Science and Biology: Proc German Conf Bioinform 99: 45–56 http://mira-assembler.sourceforge.net/.

[23] Meyer F, Paarmann D, D’Souza M, Olson R, Glass EM, et al (2008) The Metagenomics RAST server–A public resource for the automatic phylogenetic and functional analysis of metagenomes. BMC Bioinformatics 9: 386. 18803844 10.1186/1471-2105-9-386 PMC2563014

[24] Goecks J, Nekrutenko A, Taylor J (2011) The Galaxy Team. Galaxy: A comprehensive approach for supporting accessible, reproducible, and transparent computational research in the life sciences. Genome Biol 11: R86. 10.1186/gb-2010-11-8-r86 PMC2945788 20738864

[25] Moriya Y, Itoh M, Okuda S, Yoshizawa AC, Kanehisa K (2007) KAAS: An automatic genome annotation and pathway reconstruction server.; Bioinformatic Center, Institute for Chemical Research, Kyoto University, Gokasho, Uji, Kyoto 611–0011, Japan. Nucleic Acids Res 35: W182W185. 10.1093/nar/gkm321 PMC1933193 17526522

[26] Burgaud G, Le Calvez T, Arzur TD, Vandenkoornhuyse P, Barbier G (2009) Diversity of culturable marine filamentous fungi from deep-sea hydrothermal vents. Environm Microbiol 11: 1588–1600. 10.1111/j.1462-2920.2009.01886.x 19239486

[27] Bar-Even A, Noor E, Milo R (2012) A survey of carbon fixation pathways through a quantitative lens. J Exper Bot 63: 2325–2342. 22200662 10.1093/jxb/err417

[28] Rogers SO, Shtarkman YM, Koçer ZA, Edgar R, Veerapaneni R, et al. (2013) Ecology of subglacial Lake Vostok (Antarctica) based on metagenomic/metatranscriptomic analyses of accretion ice. Biology 2: 629–650. 10.3390/biology2020629 PMC3960894 24832801

[29] Ferracciolli F, Finn CA, Jordan TA, Bell RE, Anderson LM, et al (2011) East Antarctic rifting triggers uplift of the Gamburtsev Mountains. Nature 479: 388–392. 22094700 10.1038/nature10566

[30] Bulat SA, Alekhina IA, Blot M, Petit JR, Waggenbach D, et al (2004) DNA signature of thermophilic bacteria from the aged accretion ice of Lake Vostok, Antarctica: implications for searching for life in extreme icy environments. Int J Astobiol 1: 1–12.

[31] Lavire C, Normand P, Alekhina I, Bulat S, Prieur D, et al (2006) Presence of *Hydrogenophilus thermoluteolus* DNA in accretion ice in the subglacial Lake Vostok, Antarctica, assessed using *rrs*, *cbb* and *hox* . Environm Microbiol 8: 2106–2114. 10.1111/j.1462-2920.2006.01087.x 17107552

[32] Yashina S, Gubin S, Maksimovich S, Yashina A, Gakhova E, et al (2012) Regeneration of whole fertile plants from 30,000-y-old fruit tissue buried in Siberian permafrost. Proc Natl Acad Sci USA Published online before print February 21, 2012, doi:10.1073/pnas.1118386109 10.1073/pnas.1118386109 PMC3309767 22355102

[33] Bidle KD, SangHoon L, Marchant DR, Falkowski PG, et al (2007) Fossil genes and microbes in the oldest ice on Earth. Proc Natl Acad Sci USA 104: 13455–13460. 17686983 10.1073/pnas.0702196104 PMC1941643

[34] Ivanushkina NE, Kochkina GA, Ozerskaya SM (2005) Fungi in ancient permafrost sediments of the Arctic and Antarctic regions. In: Castello JD, Rogers SO, editors. Life in Ancient Ice. Princeton, NJ: Princeton University Press. 127–139.

[35] Tunnicliffe V (1991) The biology of hydrothermal vents: Ecology and evolution. Oceanorg Marine Biol Ann Rev 29: 319–407.

[36] Bell E (2012) Life at Extremes: Environments, Organisms and Strategies for Survival. Cambridge, MA: CABI. 576p.

[37] Beatty JT, Overmann J, Lince MT, Manske AK, Lang AS, et al (2005) An obligately photosynthetic bacterial anaerobe from a deep-sea hydrothermal vent. Proc Natl Acad Sci USA 102: 9306–9310. 15967984 10.1073/pnas.0503674102 PMC1166624

[38] Gaill F, Mann K, Wiedemann H, Engel J, Timpl R (1995) Structural comparison of cuticle and interstitial collagens from annelids living in shallow sea-water and at deep-sea hydrothermal vents. J Molec Biol 246: 284–294. 7869380 10.1006/jmbi.1994.0084

[39] Tarasov VG, Gebruk AV, Mironov AN, Moskalev LI (2005) Deep-sea and shallow-water hydrothermal vent communities: Two different phenomena? Chemical Geol 224: 5–39.

[40] Tunnicliffe V, McArthur AG, McHugh D (1998) A biogeographical perspective of the deep-sea hydrothermal vent fauna. Adv Marine Biol 34: 353–442.

[41] Shank TM, Black MB, Halanych KM, Lutz RA, Vrijenhoek RC (1999) Miocene radiation of deep-sea hydrothermal vent shrimp (Caridea: Bresiliidae): Evidence from mitochiondrial cytochrome oxidase subunit I. Molec Phylogen Evol. 13: 244–254. 10.1006/mpev.1999.0642 10603254

[42] Vrijenhoek RC (1997) Gene flow and genetic diversity in naturally fragmented metapolulations of deep-sea thermal vent animals. J Hered 88: 285–293. 9262010 10.1093/oxfordjournals.jhered.a023106

[43] Klok CJ (2010) Biological glass: a strategy to survive desiccation and heat. J Exp Biol 213: iv.

[44] Sømme L, Meier T (1995) Cold tolerance in Tardigrada from Dronning Maud Land, Antarctica. Polar Biol 15: 221–224.

[45] Vishnivetskaya TA, Erokhina LG, Spirina EV, Shatilovich AV, Vorobyova EA, et al. (2005) Viable phototrophs: cyanobacteria and green algae from the permafrost darkness, In: Castello JD, Rogers SO, editors. Life in Ancient Ice. Princeton, NJ: Princeton University Press. 140–158.

[46] Nagahama T (2006) Yeast Biodiversity in Freshwater, Marine and Deep-Sea Environments, In: Rosa C, Péter G, editors. Biodiversity and Ecophysiology of Yeasts. Berlin: Springer. 214–262.

[47] Ferrer M, Werner J, Chernikova TN, Barjiela R, Fernández L, et al (2011) Unveiling microbial life in the new deep-sea hypersaline Lake *Thetis*. Part II: a metagenomic study. Environm Microbiol 14: 268–281. 10.1111/j.1462-2920.2011.02634.x 22040283

[48] Toth DJ, Lerman A (1975) Stratified lake and ocean brines: salt movement and time limits of existence. Limnol Oceanog 20: 715–728.

[49] Daniel M, Cohen DM, Richard H, Rosenblatt RH, Moser HG (1990) Biology and description of a bythitid fish from deep-sea thermal vents in the tropical eastern Pacific. Deep Sea Res 37: 267–283.

[50] Young DA, Wright AP, Roberts JL, Warner RC, Young NW, et al (2011) A dynamic early East Antarctic Ice Sheet suggested by ice-covered fjord landscapes. Nature 474: 72–75. 21637255 10.1038/nature10114

[51] Zachos J, Pagani M, Sloan L, Thomas E, Billups K (2001) Trends, Rhythms, and Aberrations in Global Climate 65 Ma to Present. Science 292: 686–693. 10.1126/science.1059412 11326091

[52] Champlot S, Berthelot C, Pruvost M, Bennett EA, Grange T, et al (2010) An Efficient Multistrategy DNA Decontamination Procedure of PCR Reagents for Hypersensitive PCR Applications. PLoS ONE 5(9): e13042 doi:10.1371/journal.pone.0013042 20927390 10.1371/journal.pone.0013042 PMC2946917

[53] Salamatin AN, Tsyganova E, Lipenkov V, Petit JR (2004) Vostok (Antarctica) ice-core time-scale from datings of different origins. Annals Glaciol 39: 283–292.

