# Supplementary figures and images for: Subglacial Lake Vostok (Antarctica) Accretion Ice Contains a Diverse Set of Sequences from Aquatic, Marine and Sediment-Inhabiting Bacteria and Eukarya
Source: PLoS One. 2013 Jul 3;8(7):e67221. doi: 10.1371/journal.pone.0067221 (PMC3700977; doi:10.1371/journal.pone.0067221)

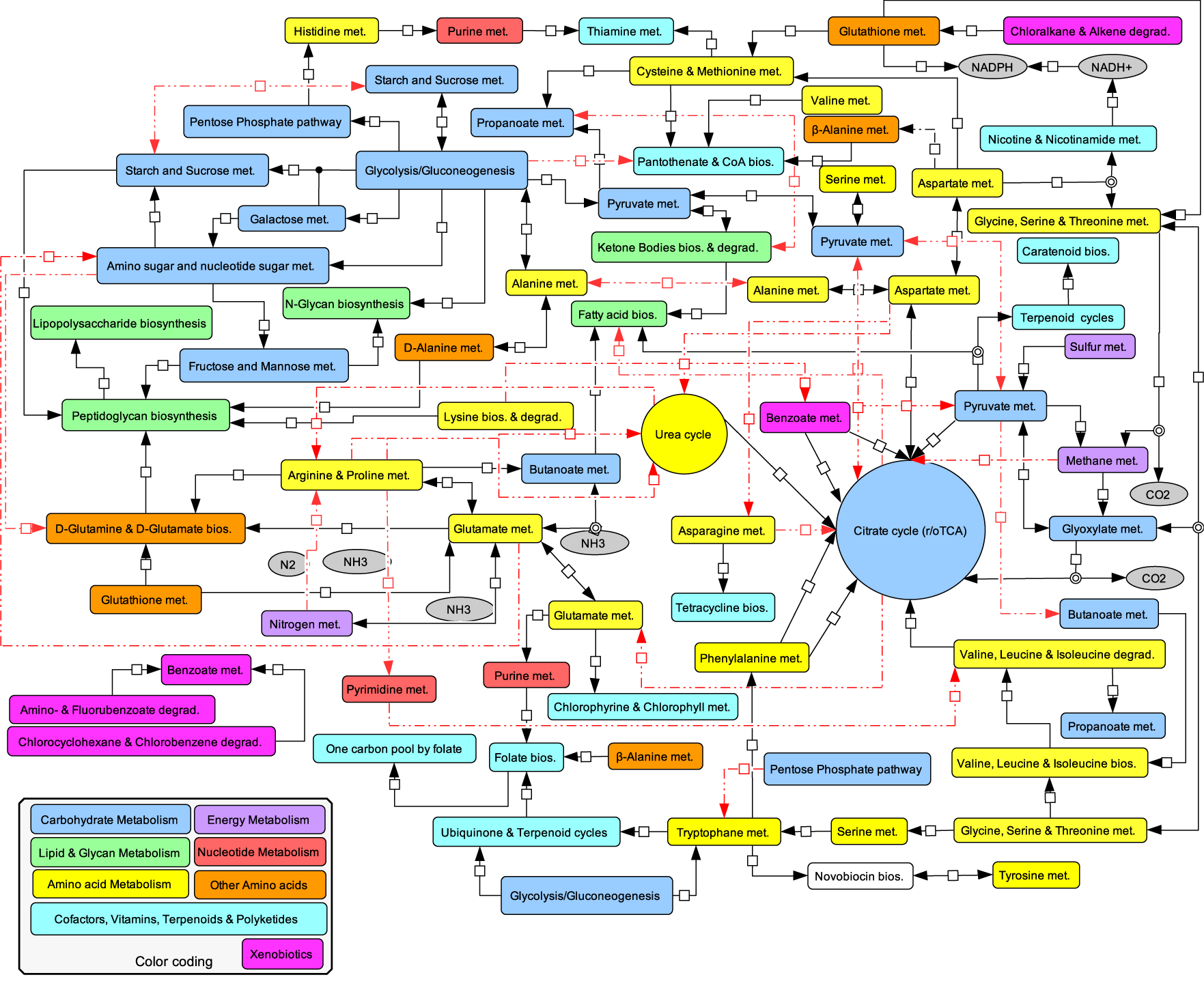

Supplement: Figure S1 — Global map of metabolic pathways represented in the data set, based on results from KAAS KEGG analyses. Color key for the metabolic pathways are indicated at the lower right. Solid black lines indicate pathways within a metabolic process. Dashed red lines indicate connections among the pathways. Dashed black lines indicate pathways not represented in the sequence database. (TIF) [file pone.0067221.s001.tif]

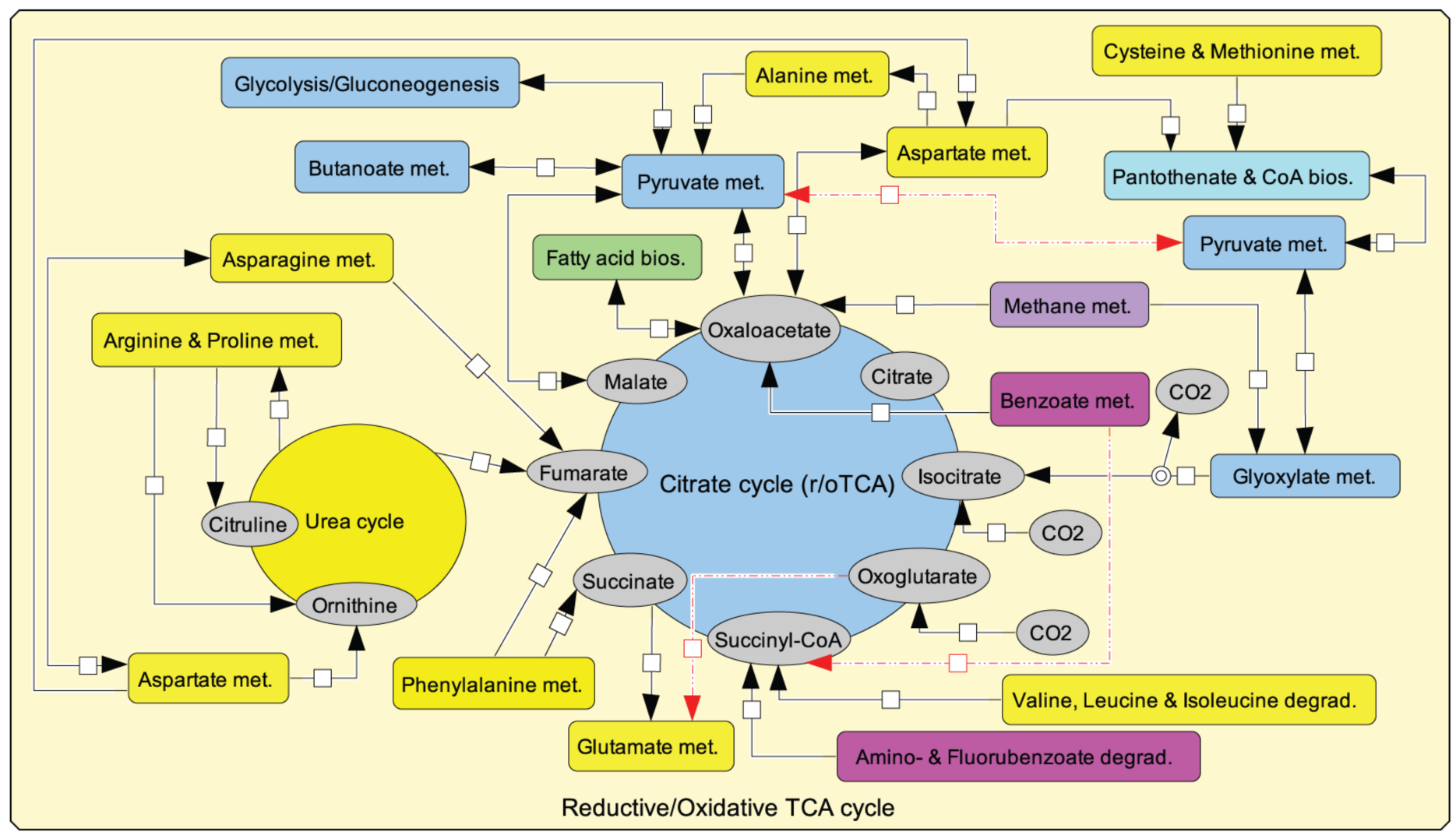

Supplement: Figure S2 — Tricarboxylic Acid (TCA) cycle, and adjoining metabolic processes found in the sequence data set. The TCA cycle in most organisms proceeds in a clockwise direction, in an oxidative process. However, many genes and organisms found in the data set operate the TCA cycle in the reverse direction (counterclockwise, termed the rTCA cycle) in a reductive process to fix CO2. In the oxidative direction, NADH and ATP are produced, which can be used in other metabolic processes. In the reverse direction, NADH and ATP were required to fix CO2 into organic compounds. Line colors and styles are as in Figure S1. (TIF) [file pone.0067221.s002.tif]

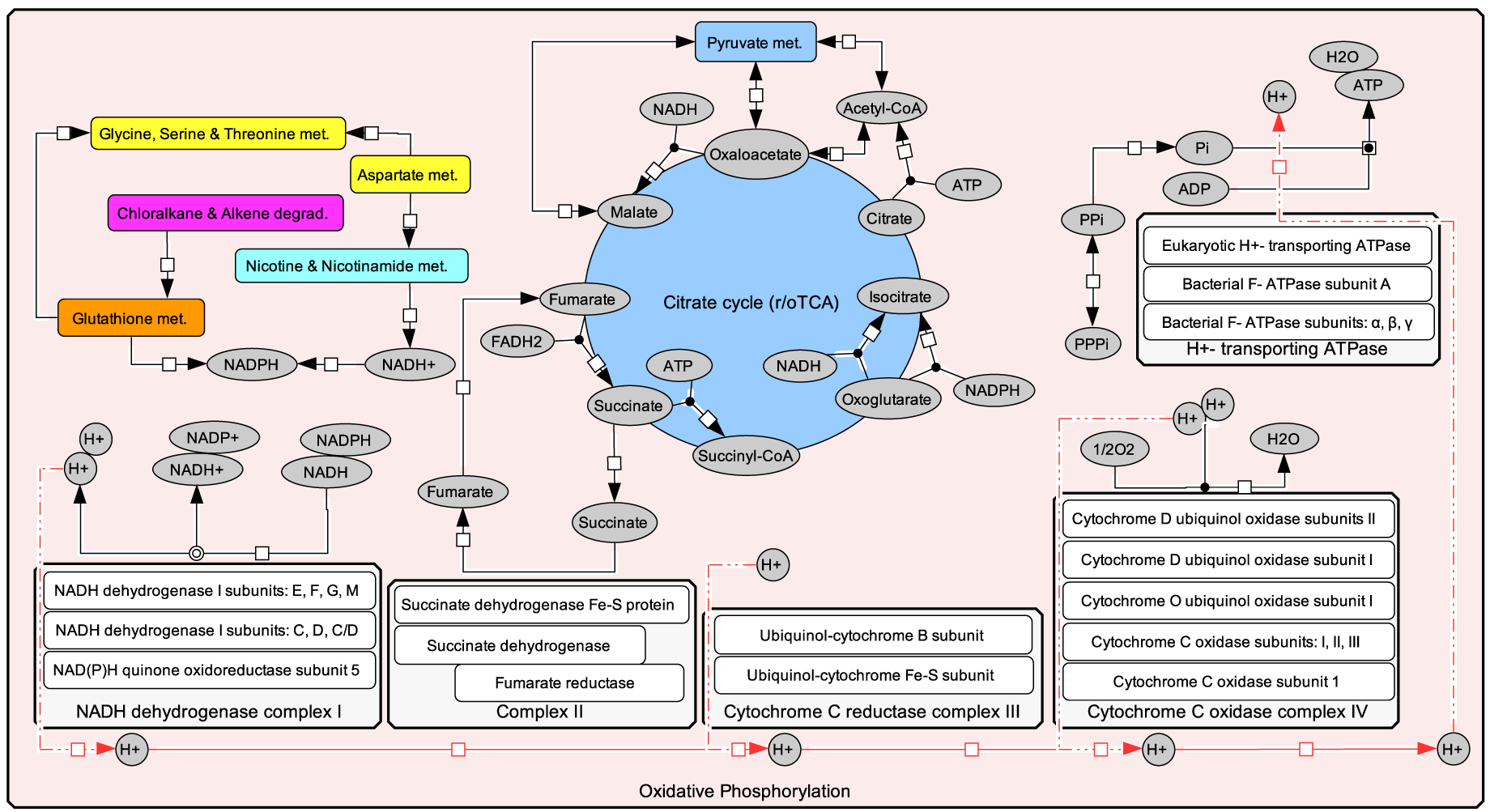

Supplement: Figure S3 — Details of connections between the TCA cycle (from Figure 2 ) and oxidative phosphorylation, based on sequences found in the data set. Line colors and styles are as in Figure S1. (TIF) [file pone.0067221.s003.tif]

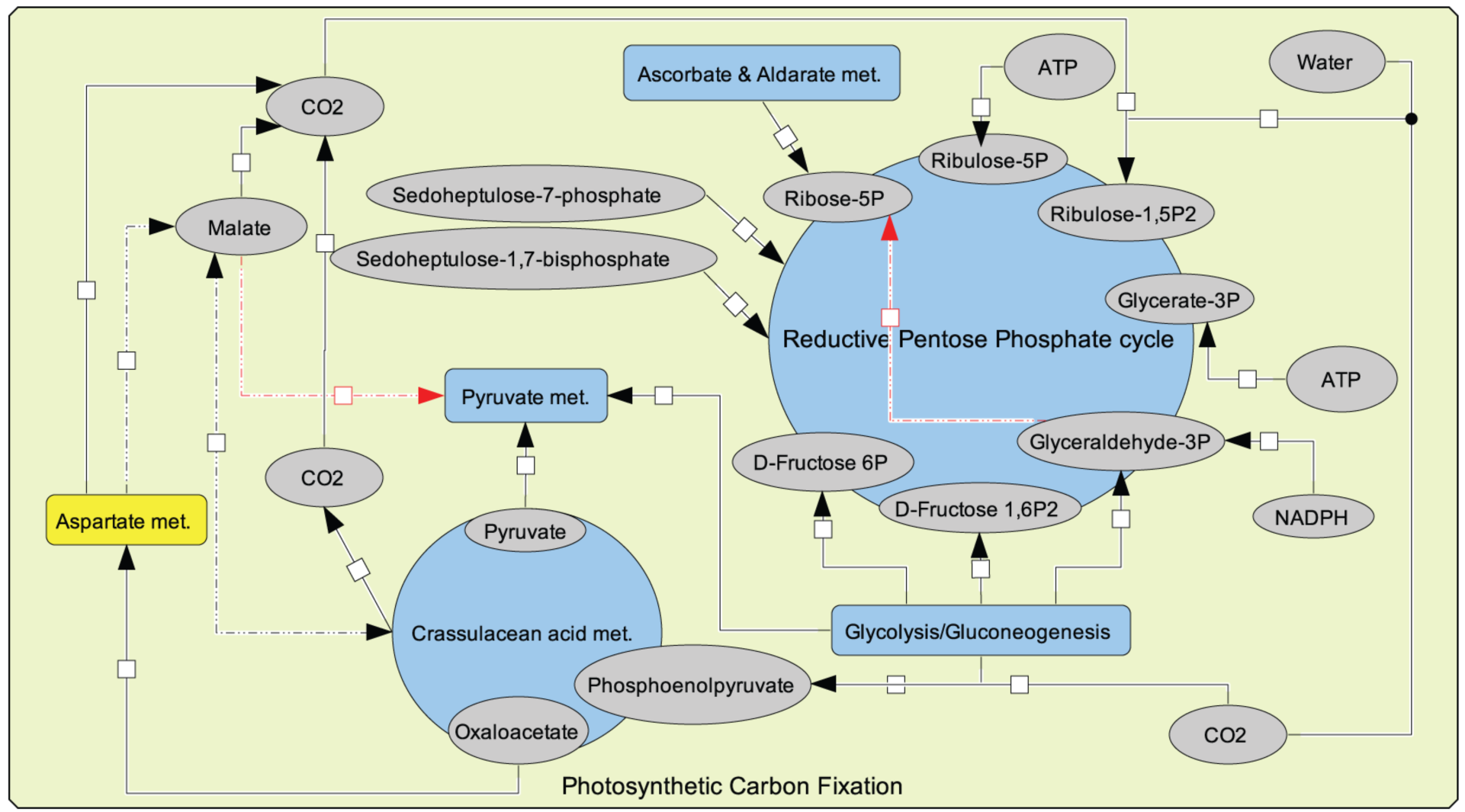

Supplement: Figure S4 — Reductive pentose phosphate (rPP) cycle and adjoining metabolic processes found in the data set. Many microbes fix CO2 using this pathway, including cyanobacteria and chloroplasts. Line colors and styles are as in Figure S1. (TIF) [file pone.0067221.s004.tif]

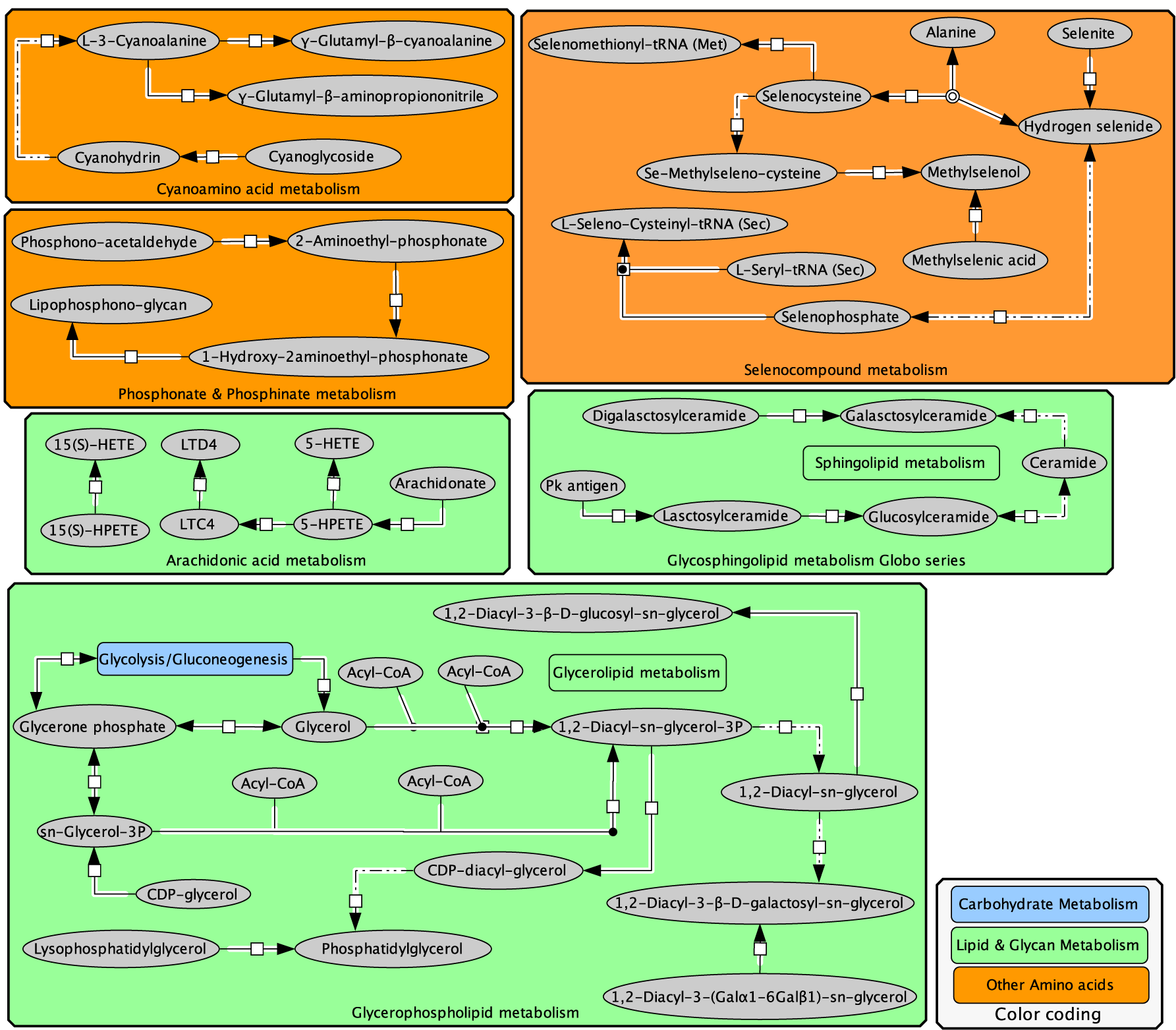

Supplement: Figure S5 — Additional reactions and pathways represented in the sequence data base. Line colors and styles are as in Figure S1. (TIF) [file pone.0067221.s005.tif]
