# Supplementary material for: Subglacial Lake Vostok (Antarctica) Accretion Ice Contains a Diverse Set of Sequences from Aquatic, Marine and Sediment-Inhabiting Bacteria and Eukarya
Source: PLoS One. 2013 Jul 3;8(7):e67221. doi: 10.1371/journal.pone.0067221 (PMC3700977; doi:10.1371/journal.pone.0067221)
Supplement: Table S1 — Small subunit rRNA gene sequences of Bacteria and Eukarya from V5. [“n” indicates information not specified in the NCBI GenBank database.]. (PDF) [file pone.0067221.s006.pdf]

Table S1. Small subunit rRNA gene sequences of Bacteria and Eukarya from V5. ["n" indicates information not specified in the NCBI GenBank database.]

Accession number	Q length	Q start	Q end	e-value	%-ident	%-sim	GI number	Domain	Phylum	Family	Genus / Species
JQ997163	424	2	424	0	96%	96%	185178423	Bacteria	Acidobacteria	n	uncultured Acidobacteriales bacterium
JQ997164	329	16	293	3E-133	98%	98%	290759818	Bacteria	Actinobacteria	Actinomycetaceae	Actinomyces georgiae
JQ997166	490	18	458	0	93%	93%	12641589	Bacteria	Actinobacteria	Actinomycetaceae	Actinomyces odontolyticus
JQ997165	320	17	275	2E-110	95%	95%	290759817	Bacteria	Actinobacteria	Actinomycetaceae	Actinomyces odontolyticus
JQ997170	575	4	574	0	100%	100%	288558711	Bacteria	Actinobacteria	Actinomycetaceae	Actinomyces oris
JQ997167	334	5	290	4E-147	100%	100%	290759821	Bacteria	Actinobacteria	Actinomycetaceae	Actinomyces oris
JQ997168	434	17	369	0	100%	100%	290759822	Bacteria	Actinobacteria	Actinomycetaceae	Actinomyces oris
JQ997169	520	4	309	2E-147	98%	98%	290759823	Bacteria	Actinobacteria	Actinomycetaceae	Actinomyces oris
JQ997171	554	5	194	9E-57	88%	88%	12641598	Bacteria	Actinobacteria	Actinomycetaceae	Actinomyces sp.
JQ997172	533	1	530	0	100%	100%	10946537	Bacteria	Actinobacteria	Actinomycetaceae	Actinomyces sp. oral clone EP011
JQ997173	550	10	538	0	99%	99%	33860321	Bacteria	Actinobacteria	Actinomycetaceae	Actinomyces sp. oral clone IO077
JQ997174	567	18	566	0	99%	99%	9837443	Bacteria	Actinobacteria	Actinomycetaceae	Actinomyces sp. oral strain B27SC
JQ997175	507	16	429	0	99%	99%	285797117	Bacteria	Actinobacteria	Actinomycetaceae	Actinomyces sp. oral taxon 169
JQ997177	540	23	532	0	100%	100%	284451154	Bacteria	Actinobacteria	Actinomycetaceae	Actinomyces sp. oral taxon 171
JQ997176	504	15	365	5E-178	99%	99%	285797225	Bacteria	Actinobacteria	Actinomycetaceae	Actinomyces sp. oral taxon 171
JQ997178	408	3	305	1E-138	97%	97%	285797322	Bacteria	Actinobacteria	Actinomycetaceae	Actinomyces sp. oral taxon 175
JQ997180	415	5	374	0	100%	100%	285797410	Bacteria	Actinobacteria	Actinomycetaceae	Actinomyces sp. oral taxon 177
JQ997179	307	5	244	3E-119	100%	100%	285797411	Bacteria	Actinobacteria	Actinomycetaceae	Actinomyces sp. oral taxon 177
JQ997181	568	1	565	0	99%	99%	284451155	Bacteria	Actinobacteria	Actinomycetaceae	Actinomyces sp. oral taxon 178
JQ997183	409	18	317	3E-149	99%	99%	285802785	Bacteria	Actinobacteria	Actinomycetaceae	Actinomyces sp. oral taxon 448
JQ997182	261	5	211	1E-101	100%	100%	285802883	Bacteria	Actinobacteria	Actinomycetaceae	Actinomyces sp. oral taxon 448
JQ997184	267	4	211	4E-102	100%	100%	285201485	Bacteria	Actinobacteria	Actinomycetaceae	Actinomyces sp. oral taxon B78
JQ997185	585	17	580	0	93%	93%	290759814	Bacteria	Actinobacteria	Actinomycetaceae	Actinomyces sp. TeJ5
JQ997188	472	5	427	0	99%	99%	12641602	Bacteria	Actinobacteria	Actinomycetaceae	Actinomyces viscosus
JQ997187	414	13	382	0	99%	99%	281485357	Bacteria	Actinobacteria	Actinomycetaceae	Actinomyces viscosus
JQ997186	365	5	264	6E-131	100%	100%	290759807	Bacteria	Actinobacteria	Actinomycetaceae	Actinomyces viscosus
JQ997189	556	23	548	0	97%	97%	162846486	Bacteria	Actinobacteria	Actinomycetaceae	uncultured Actinomyces sp.
JQ997190	270	4	212	3E-98	98%	98%	290759824	Bacteria	Actinobacteria	Corynebacteriaceae	Corynebacterium durum
JQ997191	544	5	542	0	99%	99%	282598458	Bacteria	Actinobacteria	Corynebacteriaceae	Corynebacterium sp. NML00-0156
JQ997192	337	5	274	3E-138	100%	100%	282598461	Bacteria	Actinobacteria	Corynebacteriaceae	Corynebacterium sp. NML09-0341
JQ997193	441	17	349	2E-156	97%	97%	9837446	Bacteria	Actinobacteria	Dermabacteraceae	Dermabacter sp. oral strain B46KS
JQ997194	463	4	414	0	96%	96%	291290506	Bacteria	Actinobacteria	Dermatophilaceae	Dermatophilus chelonae
JQ997195	537	4	534	0	99%	99%	291290503	Bacteria	Actinobacteria	Dermatophilaceae	Mobilicoccus pelagius
JQ997199	289	4	222	2E-104	99%	99%	255926777	Bacteria	Actinobacteria	Geodermatophilaceae	uncultured Blastococcus sp.
JQ997200	491	20	437	0	98%	98%	194680069	Bacteria	Actinobacteria	Intrasporangiaceae	Arsenicicoccus bolidensis
JQ997201	567	19	563	0	95%	95%	258678886	Bacteria	Actinobacteria	Intrasporangiaceae	Arsenicicoccus piscis
JQ997202	479	3	420	0	97%	97%	254692454	Bacteria	Actinobacteria	Intrasporangiaceae	Janibacter anophelis
JQ997203	304	18	238	2E-100	97%	97%	294992068	Bacteria	Actinobacteria	Intrasporangiaceae	Janibacter sp. MJ436
JQ997205	550	18	548	0	99%	99%	282934985	Bacteria	Actinobacteria	Intrasporangiaceae	Janibacter sp. RC5-101
JQ997206	548	18	509	0	99%	99%	197114172	Bacteria	Actinobacteria	Intrasporangiaceae	Janibacter terrae
JQ997207	420	20	373	4E-158	96%	96%	198404150	Bacteria	Actinobacteria	Intrasporangiaceae	Terracoccus sp. WPCB166
JQ997208	410	4	361	9E-175	98%	98%	169643232	Bacteria	Actinobacteria	Microbacteriaceae	Agrococcus sp. 1038/2
JQ997211	393	17	353	1E-142	95%	95%	270282501	Bacteria	Actinobacteria	Microbacteriaceae	Frigoribacterium sp. 19
JQ997212	525	18	518	0	97%	97%	37812156	Bacteria	Actinobacteria	Microbacteriaceae	Frigoribacterium sp. GWS-SE-H243
JQ997213	530	18	466	0	99%	99%	134105934	Bacteria	Actinobacteria	Microbacteriaceae	Leifsonia kribbensis
JQ997214	528	15	508	0	99%	99%	134105935	Bacteria	Actinobacteria	Microbacteriaceae	Leifsonia sp. MSL 07
JQ997215	536	17	533	0	98%	98%	283979980	Bacteria	Actinobacteria	Microbacteriaceae	Microbacteriaceae bacterium MIDF13
JQ997216	404	5	350	1E-153	95%	95%	284156650	Bacteria	Actinobacteria	Microbacteriaceae	Microbacterium sp. JDM-3-08
JQ997217	534	17	532	0	99%	99%	289594400	Bacteria	Actinobacteria	Microbacteriaceae	Microbacterium sp. KT 820
JQ997218	280	17	248	4E-107	97%	97%	254682001	Bacteria	Actinobacteria	Microbacteriaceae	Microbacterium sp. THWCSN36
JQ997219	514	17	462	0	100%	100%	111146878	Bacteria	Actinobacteria	Microbacteriaceae	Phycicola gilvus
JQ997220	533	5	473	0	99%	99%	117644155	Bacteria	Actinobacteria	Microbacteriaceae	Subtercola frigoramans
JQ997221	331	24	283	2E-106	94%	94%	195971977	Bacteria	Actinobacteria	Microbacteriaceae	uncultured Cryobacterium sp.
JQ997222	341	19	236	8E-65	89%	89%	219898423	Bacteria	Actinobacteria	Microbacteriaceae	uncultured Leifsonia sp.
JQ997223	406	17	372	0	100%	100%	270341314	Bacteria	Actinobacteria	Micrococcaceae	Arthrobacter flavus
JQ997224	414	16	341	2E-167	100%	100%	292596387	Bacteria	Actinobacteria	Micrococcaceae	Arthrobacter sp. 01-Au-006/3

JQ997225	423	4	73	7E-27	100%	100%	292596389	Bacteria	Actinobacteria	Micrococcaceae	Arthrobacter sp. 01-Je-001
JQ997226	237	5	193	7E-79	96%	96%	292596388	Bacteria	Actinobacteria	Micrococcaceae	Arthrobacter sp. 01-St-006-Luft
JQ997227	387	18	124	1E-23	88%	88%	107593744	Bacteria	Actinobacteria	Micrococcaceae	Arthrobacter sp. AE05102002_1
JQ997228	497	17	450	9E-161	91%	91%	239703779	Bacteria	Actinobacteria	Micrococcaceae	Arthrobacter sp. AMV8
JQ997229	424	3	379	0	99%	99%	145293728	Bacteria	Actinobacteria	Micrococcaceae	Arthrobacter sp. g7
JQ997230	350	12	311	5E-152	99%	99%	145293734	Bacteria	Actinobacteria	Micrococcaceae	Arthrobacter sp. h42
JQ997231	542	4	538	0	99%	99%	158562718	Bacteria	Actinobacteria	Micrococcaceae	Arthrobacter sp. NP2
JQ997232	537	5	531	0	99%	99%	158562707	Bacteria	Actinobacteria	Micrococcaceae	Arthrobacter sp. NP3
JQ997233	301	17	254	9E-119	100%	100%	228007468	Bacteria	Actinobacteria	Micrococcaceae	Arthrobacter sp. SH-43B
JQ997234	251	17	183	5E-81	100%	100%	289598546	Bacteria	Actinobacteria	Micrococcaceae	Kocuria palustris
JQ997235	296	4	251	4E-77	90%	90%	37785787	Bacteria	Actinobacteria	Micrococcaceae	Kocuria rosea
JQ997237	475	5	413	0	100%	100%	295809753	Bacteria	Actinobacteria	Micrococcaceae	Kocuria sp. DNG32
JQ999505	545	3	533	0	97%	97%	60544888	Bacteria	Actinobacteria	Micrococcaceae	Kocuria sp. T213BO3
JQ997238	520	6	461	0	97%	97%	272760971	Bacteria	Actinobacteria	Micrococcaceae	Micrococcaceae bacterium IVw_V
JQ997239	542	20	538	0	99%	99%	260100813	Bacteria	Actinobacteria	Micrococcaceae	Micrococcaceae bacterium M1S6-12
JQ997240	305	19	260	2E-115	98%	98%	154243273	Bacteria	Actinobacteria	Micrococcaceae	Micrococcaceae bacterium NASA2-30
JQ997251	549	5	520	0	95%	95%	209943871	Bacteria	Actinobacteria	Micrococcaceae	Micrococcus sp. 3S3
JQ997252	314	8	224	3E-104	99%	99%	158702950	Bacteria	Actinobacteria	Micrococcaceae	Micrococcus sp. 66H20-1
JQ997253	309	5	268	7E-125	98%	98%	219809684	Bacteria	Actinobacteria	Micrococcaceae	Micrococcus sp. BQAB-06d
JQ997254	557	5	555	0	99%	99%	219809025	Bacteria	Actinobacteria	Micrococcaceae	Micrococcus sp. BQN1N-03d
JQ997255	563	18	561	0	95%	95%	241897494	Bacteria	Actinobacteria	Micrococcaceae	Micrococcus sp. C-09
JQ997256	453	25	410	0	97%	97%	215983448	Bacteria	Actinobacteria	Micrococcaceae	Micrococcus sp. CCGE3063
JQ997257	559	19	522	0	99%	99%	240129659	Bacteria	Actinobacteria	Micrococcaceae	Micrococcus sp. CTDB2
JQ997258	277	4	226	4E-112	100%	100%	116119394	Bacteria	Actinobacteria	Micrococcaceae	Micrococcus sp. DY-1
JQ997259	259	82	201	3E-53	99%	99%	294992046	Bacteria	Actinobacteria	Micrococcaceae	Micrococcus sp. MJ314
JQ997260	545	5	543	0	99%	99%	294992048	Bacteria	Actinobacteria	Micrococcaceae	Micrococcus sp. MJ425
JQ997261	371	25	340	7E-111	90%	90%	294992049	Bacteria	Actinobacteria	Micrococcaceae	Micrococcus sp. MJ524
JQ997262	566	5	560	0	97%	97%	187319412	Bacteria	Actinobacteria	Micrococcaceae	Micrococcus sp. MOLA 73
JQ997263	531	18	254	2E-98	95%	95%	154243246	Bacteria	Actinobacteria	Micrococcaceae	Micrococcus sp. NASA2-3
JQ997264	554	5	554	0	99%	99%	157703991	Bacteria	Actinobacteria	Micrococcaceae	Micrococcus sp. SY-13
JQ997265	380	18	328	9E-125	93%	93%	265678768	Bacteria	Actinobacteria	Micrococcaceae	Nesterenkonia halotolerans
JQ997266	551	5	547	0	99%	99%	265678815	Bacteria	Actinobacteria	Micrococcaceae	Nesterenkonia lutea
JQ997267	537	18	536	0	98%	98%	283486727	Bacteria	Actinobacteria	Micrococcaceae	Nesterenkonia sandarakina
JQ997268	542	5	530	0	95%	95%	256592563	Bacteria	Actinobacteria	Micrococcaceae	Nesterenkonia sp. 110-7
JQ997269	543	4	538	0	91%	91%	256592564	Bacteria	Actinobacteria	Micrococcaceae	Nesterenkonia sp. 110-8
JQ997270	537	24	522	0	93%	93%	283486721	Bacteria	Actinobacteria	Micrococcaceae	Nesterenkonia sp. 2019
JQ997272	407	19	314	1E-133	96%	96%	219878171	Bacteria	Actinobacteria	Micrococcaceae	Rothia nasimurium
JQ997273	417	5	302	1E-153	100%	100%	295393241	Bacteria	Actinobacteria	Micrococcaceae	uncultured Rothia sp.
JQ997275	285	5	232	6E-115	100%	100%	260871474	Bacteria	Actinobacteria	Mycobacteriaceae	Mycobacterium sp. GN-10803
JQ997276	263	18	170	3E-73	100%	100%	62736086	Bacteria	Actinobacteria	Mycobacteriaceae	uncultured Mycobacterium sp.
JQ997277	548	19	548	0	99%	99%	146166675	Bacteria	Actinobacteria	n	Micrococcineae bacterium 4_C16_66
JQ997278	410	17	375	0	99%	99%	269113439	Bacteria	Actinobacteria	n	uncultured Actinomycetales bacterium
JQ997279	575	5	235	2E-107	98%	98%	11127808	Bacteria	Actinobacteria	n	uncultured sheep mite bacterium Llangefni 35
JQ997280	542	17	536	0	92%	92%	111146975	Bacteria	Actinobacteria	Nocardioidaceae	Marmoricola aequoreus
JQ997281	335	18	277	1E-132	100%	100%	215983420	Bacteria	Actinobacteria	Nocardioidaceae	Nocardioides sp. CCGE2239
JQ997282	455	18	318	3E-150	99%	99%	293629578	Bacteria	Actinobacteria	Nocardioidaceae	Nocardioides sp. Cr7-14
JQ997283	457	5	407	0	98%	98%	291464959	Bacteria	Actinobacteria	Nocardioidaceae	uncultured Nocardioides sp.
JQ997285	561	5	555	0	97%	97%	8574099	Bacteria	Actinobacteria	Streptomycetaceae	Streptomyces rimosus
JQ997286	461	3	55	8E-12	94%	94%	295702212	Bacteria	Actinobacteria	Streptomycetaceae	Streptomyces sp. 175_2010_
JQ997288	498	17	376	3E-175	98%	98%	219816071	Bacteria	Actinobacteria	Yaniellaceae	Yaniella soli
JQ997289	550	57	545	0	92%	92%	284930174	Bacteria	Actinobacteria	Bifidobacteriaceae	Bifidobacterium pullorum
JQ997290	473	5	419	8E-171	94%	94%	284930176	Bacteria	Actinobacteria	Bifidobacteriaceae	Bifidobacterium saeculare
JQ997292	404	18	357	5E-177	100%	100%	239924942	Bacteria	Actinobacteria	Bifidobacteriaceae	Parascardovia denticolens
JQ997291	303	5	230	9E-114	100%	100%	285178409	Bacteria	Actinobacteria	Bifidobacteriaceae	Parascardovia denticolens
JQ997293	547	46	546	0	98%	98%	295147946	Bacteria	Actinobacteria	Coriobacteriaceae	Atopobium parvulum
JQ997294	370	18	283	3E-134	100%	100%	34329838	Bacteria	Actinobacteria	n	actinobacterium iEI7
JQ997306	590	18	537	0	94%	94%	87042306	Bacteria	Actinobacteria	n	uncultured actinobacterium
JQ997296	257	70	225	1E-71	99%	99%	105990462	Bacteria	Actinobacteria	n	uncultured actinobacterium
JQ997304	573	234	566	4E-165	99%	99%	154186968	Bacteria	Actinobacteria	n	uncultured actinobacterium
JQ997300	474	11	415	0	99%	99%	154199122	Bacteria	Actinobacteria	n	uncultured actinobacterium

JQ997301	516	5	480	0	100%	100%	218533798	Bacteria	Actinobacteria	n	uncultured actinobacterium
JQ997302	538	43	422	0	98%	98%	220682447	Bacteria	Actinobacteria	n	uncultured actinobacterium
JQ997303	559	5	493	3E-171	90%	90%	226918723	Bacteria	Actinobacteria	n	uncultured actinobacterium
JQ997299	392	5	359	2E-175	98%	98%	237637651	Bacteria	Actinobacteria	n	uncultured actinobacterium
JQ997305	574	19	487	0	97%	97%	237637665	Bacteria	Actinobacteria	n	uncultured actinobacterium
JQ997298	277	4	221	2E-109	100%	100%	284387472	Bacteria	Actinobacteria	n	uncultured actinobacterium
JQ997295	251	5	144	5E-66	100%	100%	290565057	Bacteria	Actinobacteria	n	uncultured actinobacterium
JQ997297	266	18	174	7E-55	92%	92%	290794332	Bacteria	Actinobacteria	n	uncultured actinobacterium
JQ998479	465	18	340	5E-148	97%	97%	237934379	Bacteria	Actinobacteria	n	uncultured bacterium
JQ997307	527	18	464	0	98%	98%	159159332	Bacteria	Bacteroidetes	Bacteroidaceae	Bacteroides coprocola
JQ997314	394	5	349	8E-180	100%	100%	175940971	Bacteria	Bacteroidetes	Bacteroidaceae	uncultured Bacteroides sp.
JQ997312	315	4	271	2E-130	99%	99%	208689585	Bacteria	Bacteroidetes	Bacteroidaceae	uncultured Bacteroides sp.
JQ997310	284	18	252	4E-92	93%	93%	208690692	Bacteria	Bacteroidetes	Bacteroidaceae	uncultured Bacteroides sp.
JQ997317	455	2	398	8E-176	95%	95%	208690861	Bacteria	Bacteroidetes	Bacteroidaceae	uncultured Bacteroides sp.
JQ997318	464	18	411	1E-178	96%	96%	281324569	Bacteria	Bacteroidetes	Bacteroidaceae	uncultured Bacteroides sp.
JQ997319	576	5	564	0	92%	92%	281324578	Bacteria	Bacteroidetes	Bacteroidaceae	uncultured Bacteroides sp.
JQ997315	401	4	357	2E-151	94%	94%	281324586	Bacteria	Bacteroidetes	Bacteroidaceae	uncultured Bacteroides sp.
JQ997311	312	25	225	3E-93	98%	98%	281324587	Bacteria	Bacteroidetes	Bacteroidaceae	uncultured Bacteroides sp.
JQ997316	404	2	349	2E-156	96%	96%	281324588	Bacteria	Bacteroidetes	Bacteroidaceae	uncultured Bacteroides sp.
JQ997313	379	5	310	1E-143	97%	97%	281324591	Bacteria	Bacteroidetes	Bacteroidaceae	uncultured Bacteroides sp.
JQ997324	564	24	525	0	99%	99%	154193781	Bacteria	Bacteroidetes	n	uncultured Bacteroidales bacterium
JQ997322	557	5	531	0	99%	99%	154193791	Bacteria	Bacteroidetes	n	uncultured Bacteroidales bacterium
JQ997320	439	5	359	1E-163	96%	96%	217337425	Bacteria	Bacteroidetes	n	uncultured Bacteroidales bacterium
JQ997321	498	23	340	5E-163	100%	100%	261265014	Bacteria	Bacteroidetes	n	uncultured Bacteroidales bacterium
JQ997325	574	22	327	2E-67	83%	83%	159159377	Bacteria	Bacteroidetes	Porphyromonadaceae	Parabacteroides goldsteinii
JQ997327	474	23	427	6E-147	90%	90%	86371916	Bacteria	Bacteroidetes	Porphyromonadaceae	uncultured Porphyromonas sp.
JQ997326	276	9	114	2E-45	99%	99%	294613743	Bacteria	Bacteroidetes	Porphyromonadaceae	uncultured Porphyromonas sp.
JQ997328	329	18	297	2E-125	96%	96%	215273719	Bacteria	Bacteroidetes	Prevotellaceae	Paraprevotella xylaniphila
JQ997329	556	18	552	0	99%	99%	284451151	Bacteria	Bacteroidetes	Prevotellaceae	Prevotella denticola
JQ997330	372	5	335	3E-109	89%	89%	189406708	Bacteria	Bacteroidetes	Prevotellaceae	Prevotella falsenii
JQ997333	550	20	505	0	97%	97%	32492917	Bacteria	Bacteroidetes	Prevotellaceae	Prevotella melaninogenica
JQ997332	437	18	386	0	100%	100%	290759845	Bacteria	Bacteroidetes	Prevotellaceae	Prevotella melaninogenica
JQ997331	269	4	216	5E-96	97%	97%	290759846	Bacteria	Bacteroidetes	Prevotellaceae	Prevotella melaninogenica
JQ997334	294	18	276	4E-132	100%	100%	213399744	Bacteria	Bacteroidetes	Prevotellaceae	Prevotella sp. 8400706
JQ997335	421	18	382	7E-176	98%	98%	14161353	Bacteria	Bacteroidetes	Prevotellaceae	uncultured Prevotella sp.
JQ997336	461	4	424	0	99%	99%	253683837	Bacteria	Bacteroidetes	Prevotellaceae	uncultured Prevotella sp.
JQ997337	538	18	537	0	99%	99%	290759830	Bacteria	Bacteroidetes	Flavobacteriaceae	Capnocytophaga granulosa
JQ997338	316	18	268	1E-127	100%	100%	283443628	Bacteria	Bacteroidetes	Flavobacteriaceae	Flavobacterium johnsoniae
JQ997339	352	17	298	4E-133	98%	98%	157170669	Bacteria	Bacteroidetes	Flavobacteriaceae	Flavobacterium sp. P-131
JQ999022	552	17	310	5E-119	94%	94%	237931356	Bacteria	Bacteroidetes	n	uncultured bacterium
JQ997350	521	16	465	0	99%	99%	60266503	Bacteria	Bacteroidetes	n	uncultured Bacteroidetes bacterium
JQ997356	577	5	571	0	97%	97%	118772949	Bacteria	Bacteroidetes	n	uncultured Bacteroidetes bacterium
JQ997349	515	18	469	0	98%	98%	118772961	Bacteria	Bacteroidetes	n	uncultured Bacteroidetes bacterium
JQ997340	261	162	223	1E-22	100%	100%	126131306	Bacteria	Bacteroidetes	n	uncultured Bacteroidetes bacterium
JQ997353	546	17	542	0	97%	97%	151936618	Bacteria	Bacteroidetes	n	uncultured Bacteroidetes bacterium
JQ997348	500	24	438	0	100%	100%	197360255	Bacteria	Bacteroidetes	n	uncultured Bacteroidetes bacterium
JQ997351	538	18	537	2E-163	87%	87%	222079937	Bacteria	Bacteroidetes	n	uncultured Bacteroidetes bacterium
JQ997346	370	17	260	1E-123	100%	100%	239619880	Bacteria	Bacteroidetes	n	uncultured Bacteroidetes bacterium
JQ997352	544	18	510	0	98%	98%	291329669	Bacteria	Bacteroidetes	n	uncultured Bacteroidetes bacterium
JQ997344	364	5	306	6E-156	100%	100%	291329690	Bacteria	Bacteroidetes	n	uncultured Bacteroidetes bacterium
JQ997342	317	18	169	1E-72	100%	100%	291329981	Bacteria	Bacteroidetes	n	uncultured Bacteroidetes bacterium
JQ997354	546	5	510	0	100%	100%	291330802	Bacteria	Bacteroidetes	n	uncultured Bacteroidetes bacterium
JQ997355	565	23	565	0	99%	99%	291330808	Bacteria	Bacteroidetes	n	uncultured Bacteroidetes bacterium
JQ997343	362	4	312	8E-155	99%	99%	291330883	Bacteria	Bacteroidetes	n	uncultured Bacteroidetes bacterium
JQ997341	277	3	232	5E-116	100%	100%	291330884	Bacteria	Bacteroidetes	n	uncultured Bacteroidetes bacterium
JQ997347	437	5	380	0	99%	99%	291332865	Bacteria	Bacteroidetes	n	uncultured Bacteroidetes bacterium
JQ997345	369	24	304	4E-118	94%	94%	291332904	Bacteria	Bacteroidetes	n	uncultured Bacteroidetes bacterium
JQ999501	547	18	507	0	98%	98%	219961820	Bacteria	Bacteroidetes	n	uncultured Bacteroidetes bacterium
JQ997357	239	5	195	4E-71	93%	93%	246367044	Bacteria	Bacteroidetes	Sphingobacteriaceae	Pedobacter sp. BZ42
JQ997358	545	17	539	0	96%	96%	211908640	Bacteria	Bacteroidetes	Sphingobacteriaceae	Pedobacter sp. L2b-1

JQ997359	496	4	358	2E-171	98%	98%	225382587	Bacteria	Bacteroidetes	Sphingobacteriaceae	Sphingobacterium shayense
JQ997360	373	17	327	3E-154	99%	99%	203289069	Bacteria	Bacteroidetes	Sphingobacteriaceae	Sphingobacterium sp. MOL-1
JQ997362	256	15	224	3E-103	100%	100%	295149401	Bacteria	Cyanobacteria	n	uncultured Cyanobacterium sp.
JQ997363	332	5	282	1E-118	95%	95%	148299079	Bacteria	Cyanobacteria	n	cyanobacterium OSC
JQ997478	202	1	202	8E-59	88%	88%	372197969	Bacteria	Cyanobacteria	n	n
JQ997364	402	10	359	1E-157	96%	96%	34808714	Bacteria	Cyanobacteria	n	uncultured Antarctic cyanobacterium
JQ997367	759	5	136	1E-46	94%	94%	46948073	Bacteria	Cyanobacteria	n	uncultured Antarctic cyanobacterium
JQ997366	546	18	543	0	94%	94%	46948088	Bacteria	Cyanobacteria	n	uncultured Antarctic cyanobacterium
JQ997365	460	5	299	1E-114	93%	93%	220683477	Bacteria	Cyanobacteria	n	uncultured Antarctic cyanobacterium
JQ997368	283	18	253	1E-101	96%	96%	15212605	Bacteria	Cyanobacteria	n	uncultured cyanobacterium
JQ997380	466	5	383	6E-177	97%	97%	76096831	Bacteria	Cyanobacteria	n	uncultured cyanobacterium
JQ997377	401	18	323	1E-152	99%	99%	93359876	Bacteria	Cyanobacteria	n	uncultured cyanobacterium
JQ997376	384	27	315	1E-113	93%	93%	105990288	Bacteria	Cyanobacteria	n	uncultured cyanobacterium
JQ997379	434	13	386	2E-177	97%	97%	105990324	Bacteria	Cyanobacteria	n	uncultured cyanobacterium
JQ997383	504	17	456	0	95%	95%	129563748	Bacteria	Cyanobacteria	n	uncultured cyanobacterium
JQ997386	535	18	529	0	92%	92%	129563749	Bacteria	Cyanobacteria	n	uncultured cyanobacterium
JQ997387	536	5	535	0	92%	92%	146141933	Bacteria	Cyanobacteria	n	uncultured cyanobacterium
JQ997372	339	7	282	8E-125	96%	96%	146141966	Bacteria	Cyanobacteria	n	uncultured cyanobacterium
JQ997381	477	69	442	4E-164	95%	95%	149350798	Bacteria	Cyanobacteria	n	uncultured cyanobacterium
JQ997389	547	15	543	0	91%	91%	162289063	Bacteria	Cyanobacteria	n	uncultured cyanobacterium
JQ997391	556	5	525	0	95%	95%	192337841	Bacteria	Cyanobacteria	n	uncultured cyanobacterium
JQ997373	354	18	189	2E-60	92%	92%	192804239	Bacteria	Cyanobacteria	n	uncultured cyanobacterium
JQ997382	480	5	350	4E-159	97%	97%	213053960	Bacteria	Cyanobacteria	n	uncultured cyanobacterium
JQ997370	318	2	273	6E-101	92%	92%	219883478	Bacteria	Cyanobacteria	n	uncultured cyanobacterium
JQ997378	412	259	367	2E-31	92%	92%	227072228	Bacteria	Cyanobacteria	n	uncultured cyanobacterium
JQ997390	551	17	531	0	95%	95%	227072230	Bacteria	Cyanobacteria	n	uncultured cyanobacterium
JQ997385	517	18	240	8E-52	85%	85%	229562671	Bacteria	Cyanobacteria	n	uncultured cyanobacterium
JQ997369	290	5	195	2E-79	95%	95%	229563859	Bacteria	Cyanobacteria	n	uncultured cyanobacterium
JQ997392	556	4	554	0	97%	97%	238632326	Bacteria	Cyanobacteria	n	uncultured cyanobacterium
JQ997395	678	336	641	8E-63	83%	83%	261290524	Bacteria	Cyanobacteria	n	uncultured cyanobacterium
JQ997388	540	1	533	0	92%	92%	282765201	Bacteria	Cyanobacteria	n	uncultured cyanobacterium
JQ997371	328	228	255	0.001	100%	100%	285015409	Bacteria	Cyanobacteria	n	uncultured cyanobacterium
JQ997397	325	5	294	3E-129	96%	96%	18182396	Bacteria	Cyanobacteria	n	uncultured soil crust cyanobacterium
JQ997402	577	212	489	8E-102	92%	92%	18182408	Bacteria	Cyanobacteria	n	uncultured soil crust cyanobacterium
JQ997398	327	5	236	5E-112	99%	99%	18182418	Bacteria	Cyanobacteria	n	uncultured soil crust cyanobacterium
JQ997404	690	18	222	4E-76	93%	93%	18182460	Bacteria	Cyanobacteria	n	uncultured soil crust cyanobacterium
JQ997396	317	5	280	2E-126	97%	97%	18182464	Bacteria	Cyanobacteria	n	uncultured soil crust cyanobacterium
JQ997401	392	3	276	5E-127	97%	97%	18182489	Bacteria	Cyanobacteria	n	uncultured soil crust cyanobacterium
JQ997399	329	22	284	3E-134	100%	100%	18182490	Bacteria	Cyanobacteria	n	uncultured soil crust cyanobacterium
JQ997400	389	18	358	6E-176	100%	100%	18182491	Bacteria	Cyanobacteria	n	uncultured soil crust cyanobacterium
JQ997405	445	5	412	0	97%	97%	21388238	Bacteria	Cyanobacteria	Nostocaceae	Anabaena azotica
JQ997406	357	18	326	4E-88	87%	87%	19343359	Bacteria	Cyanobacteria	Nostocaceae	Nodularia spumigena
JQ997407	533	17	528	0	99%	99%	291360379	Bacteria	Cyanobacteria	Nostocaceae	Nostoc flagelliforme
JQ997408	308	13	262	4E-122	99%	99%	29124940	Bacteria	Cyanobacteria	Nostocaceae	Nostoc muscorum
JQ997409	514	17	514	0	99%	99%	82470879	Bacteria	Cyanobacteria	Nostocaceae	Nostoc sp. _Mollenhauer 1:1-115_
JQ997410	297	18	266	7E-100	93%	93%	124108938	Bacteria	Cyanobacteria	Nostocaceae	Nostoc sp. _Pannaria durietzii cyanobiont_ 1 NZ
JQ997411	549	4	543	0	95%	95%	82470910	Bacteria	Cyanobacteria	Nostocaceae	Nostoc sp. PCC 7423
JQ997412	263	5	168	2E-79	100%	100%	154361765	Bacteria	Cyanobacteria	Nostocaceae	Nostoc sp. SKJF2
JQ997413	707	23	75	3E-13	96%	96%	225922035	Bacteria	Cyanobacteria	Nostocaceae	uncultured Nostoc sp.
JQ997414	403	5	357	0	100%	100%	82697090	Bacteria	Cyanobacteria	n	Leptolyngbya sp. 0BB32S02
JQ997415	328	17	44	0.001	100%	100%	172050761	Bacteria	Cyanobacteria	n	Lyngbya birgei
JQ997416	561	3	554	0	95%	95%	12004672	Bacteria	Cyanobacteria	n	Microcoleus acremanii
JQ997417	497	17	456	0	99%	99%	149364155	Bacteria	Cyanobacteria	n	Microcoleus sp. HTT-U-KK5
JQ997418	416	5	343	2E-176	100%	100%	149364160	Bacteria	Cyanobacteria	n	Microcoleus sp. SAG 2212
JQ997419	538	5	505	0	91%	91%	19879913	Bacteria	Cyanobacteria	n	Microcoleus steenstrupii
JQ997453	442	3	234	6E-117	100%	100%	33327320	Bacteria	Cyanobacteria	n	Oscillatoria amoena
JQ997454	532	5	475	0	94%	94%	291603789	Bacteria	Cyanobacteria	n	Oscillatoria margaritifera
JQ997455	540	2	452	0	99%	99%	89242016	Bacteria	Cyanobacteria	n	Oscillatoria prolifera
JQ997456	489	15	377	6E-172	97%	97%	161723071	Bacteria	Cyanobacteria	n	Oscillatoria sp. 195-A20
JQ997457	479	24	445	0	96%	96%	222876471	Bacteria	Cyanobacteria	n	Oscillatoria sp. 327/2

JQ997458	238	2	184	7E-79	96%	96%	15428333	Bacteria	Cyanobacteria	n	Oscillatoria sp. Ant-G16
JQ997459	285	15	205	2E-94	100%	100%	23978201	Bacteria	Cyanobacteria	n	Oscillatoria sp. PCC 7112
JQ997460	558	17	525	0	98%	98%	281308415	Bacteria	Cyanobacteria	n	Oscillatoriales cyanobacterium 2Dp86E
JQ997461	351	18	317	8E-155	100%	100%	37782175	Bacteria	Cyanobacteria	n	Oscillatoriales cyanobacterium IL-1.4
JQ997462	517	7	478	1E-178	91%	91%	149364150	Bacteria	Cyanobacteria	n	Phormidiaceae cyanobacterium CPER-KK1
JQ997469	453	17	334	4E-34	77%	77%	124491646	Bacteria	Cyanobacteria	n	Phormidium autumnale
JQ997474	564	5	558	0	99%	99%	166997748	Bacteria	Cyanobacteria	n	Phormidium autumnale
JQ997476	585	5	583	0	92%	92%	166997749	Bacteria	Cyanobacteria	n	Phormidium autumnale
JQ997472	525	15	481	0	95%	95%	167508106	Bacteria	Cyanobacteria	n	Phormidium autumnale
JQ997475	571	10	568	0	97%	97%	167508107	Bacteria	Cyanobacteria	n	Phormidium autumnale
JQ997468	447	5	402	2E-177	95%	95%	167508108	Bacteria	Cyanobacteria	n	Phormidium autumnale
JQ997466	354	5	303	2E-135	96%	96%	167508118	Bacteria	Cyanobacteria	n	Phormidium autumnale
JQ997467	378	22	344	1E-158	98%	98%	167508119	Bacteria	Cyanobacteria	n	Phormidium autumnale
JQ997473	555	18	553	0	97%	97%	167508120	Bacteria	Cyanobacteria	n	Phormidium autumnale
JQ997471	490	18	434	1E-169	93%	93%	258547383	Bacteria	Cyanobacteria	n	Phormidium autumnale
JQ997470	478	18	429	1E-154	91%	91%	258547389	Bacteria	Cyanobacteria	n	Phormidium autumnale
JQ997465	328	16	236	6E-91	95%	95%	289976368	Bacteria	Cyanobacteria	n	Phormidium autumnale
JQ997477	317	31	285	2E-106	95%	95%	158452035	Bacteria	Cyanobacteria	n	Phormidium corium
JQ997479	548	1	545	0	93%	93%	149166808	Bacteria	Cyanobacteria	n	Phormidium sp. KU003
JQ997480	269	5	223	3E-108	100%	100%	1668785	Bacteria	Cyanobacteria	n	Phormidium sp. NIVA-CYA 203
JQ997481	527	18	515	0	95%	95%	167508121	Bacteria	Cyanobacteria	n	Phormidium subfuscum
JQ997482	245	7	113	1E-37	94%	94%	284159158	Bacteria	Cyanobacteria	n	uncultured Hydrocoleum sp.
JQ997483	443	29	397	3E-165	95%	95%	225382331	Bacteria	Cyanobacteria	n	uncultured Oscillatoriales cyanobacterium
JQ997484	551	4	546	0	100%	100%	225382352	Bacteria	Cyanobacteria	n	uncultured Oscillatoriales cyanobacterium
JQ997485	565	5	554	0	88%	88%	225382365	Bacteria	Cyanobacteria	n	uncultured Oscillatoriales cyanobacterium
JQ997486	246	18	214	4E-86	96%	96%	220683485	Bacteria	Cyanobacteria	n	Wilmottia murrayi
JQ997489	357	3	280	2E-125	96%	96%	225696175	Bacteria	Cyanobacteria	n	uncultured Chroococcidiopsis sp.
JQ997497	571	18	565	0	92%	92%	225696186	Bacteria	Cyanobacteria	n	uncultured Chroococcidiopsis sp.
JQ997487	307	15	118	1E-17	84%	84%	225696195	Bacteria	Cyanobacteria	n	uncultured Chroococcidiopsis sp.
JQ997494	535	5	402	3E-156	92%	92%	225696229	Bacteria	Cyanobacteria	n	uncultured Chroococcidiopsis sp.
JQ997493	519	14	482	1E-115	84%	84%	225696231	Bacteria	Cyanobacteria	n	uncultured Chroococcidiopsis sp.
JQ997491	386	19	279	3E-89	90%	90%	225696240	Bacteria	Cyanobacteria	n	uncultured Chroococcidiopsis sp.
JQ997488	331	4	286	4E-127	96%	96%	225696241	Bacteria	Cyanobacteria	n	uncultured Chroococcidiopsis sp.
JQ997492	427	1	106	9E-31	91%	91%	225696253	Bacteria	Cyanobacteria	n	uncultured Chroococcidiopsis sp.
JQ997495	546	5	546	0	93%	93%	225696255	Bacteria	Cyanobacteria	n	uncultured Chroococcidiopsis sp.
JQ997490	377	5	92	6E-32	97%	97%	225696261	Bacteria	Cyanobacteria	n	uncultured Chroococcidiopsis sp.
JQ997496	552	5	542	0	94%	94%	225696263	Bacteria	Cyanobacteria	n	uncultured Chroococcidiopsis sp.
JQ997498	531	19	467	0	97%	97%	3093968	Bacteria	Deferribacteres	Deferribacteraceae	Mucispirillum schaedleri
JQ997499	495	25	419	0	97%	97%	37665094	Bacteria	Deinococcus-Thermus	Deinococcaceae	Deinococcus marmoris
JQ997500	332	18	128	9E-25	87%	87%	214091025	Bacteria	Firmicutes	Bacillaceae	Anoxybacillus sp. F81
JQ997532	347	1	347	1E-173	99%	99%	15042017	Bacteria	Firmicutes	Bacillaceae	Bacillus
JQ997502	568	7	541	0	98%	98%	254826546	Bacteria	Firmicutes	Bacillaceae	Bacillus agaradhaerens
JQ997501	546	5	397	0	99%	99%	283486716	Bacteria	Firmicutes	Bacillaceae	Bacillus agaradhaerens
JQ997504	535	19	532	0	100%	100%	269994025	Bacteria	Firmicutes	Bacillaceae	Bacillus cereus
JQ997503	524	5	521	0	99%	99%	270297792	Bacteria	Firmicutes	Bacillaceae	Bacillus cereus
JQ997505	543	5	541	0	99%	99%	294999187	Bacteria	Firmicutes	Bacillaceae	Bacillus cereus
JQ997506	341	23	290	6E-131	99%	99%	294769167	Bacteria	Firmicutes	Bacillaceae	Bacillus circulans
JQ997507	457	18	381	0	99%	99%	223959348	Bacteria	Firmicutes	Bacillaceae	Bacillus cohnii
JQ997508	534	17	498	0	99%	99%	94962022	Bacteria	Firmicutes	Bacillaceae	Bacillus decisifrondis
JQ997509	463	15	393	0	99%	99%	209552631	Bacteria	Firmicutes	Bacillaceae	Bacillus halmapalus
JQ997511	535	18	522	0	97%	97%	13899044	Bacteria	Firmicutes	Bacillaceae	Bacillus horikoshii
JQ997510	248	20	216	4E-81	95%	95%	239812465	Bacteria	Firmicutes	Bacillaceae	Bacillus horikoshii
JQ997514	572	4	572	0	96%	96%	254675492	Bacteria	Firmicutes	Bacillaceae	Bacillus horikoshii
JQ997512	538	5	533	0	97%	97%	262410334	Bacteria	Firmicutes	Bacillaceae	Bacillus horikoshii
JQ997513	556	11	554	0	89%	89%	264667843	Bacteria	Firmicutes	Bacillaceae	Bacillus horikoshii
JQ997515	581	18	576	0	94%	94%	264667850	Bacteria	Firmicutes	Bacillaceae	Bacillus horikoshii
JQ997516	551	12	547	0	92%	92%	47847537	Bacteria	Firmicutes	Bacillaceae	Bacillus horti
JQ997518	356	5	305	2E-90	87%	87%	134290370	Bacteria	Firmicutes	Bacillaceae	Bacillus megaterium
JQ997517	270	3	54	4E-17	100%	100%	295790202	Bacteria	Firmicutes	Bacillaceae	Bacillus megaterium
JQ997519	431	21	323	1E-148	99%	99%	78038860	Bacteria	Firmicutes	Bacillaceae	Bacillus sp. 7327

JQ997520	556	16	527	0	92%	92%	148357814	Bacteria	Firmicutes	Bacillaceae	Bacillus sp. 8SB
JQ997521	539	4	538	0	91%	91%	56417368	Bacteria	Firmicutes	Bacillaceae	Bacillus sp. BA299
JQ997522	278	5	231	1E-112	100%	100%	289065368	Bacteria	Firmicutes	Bacillaceae	Bacillus sp. CCBAU 05776
JQ997523	540	5	534	0	99%	99%	24415973	Bacteria	Firmicutes	Bacillaceae	Bacillus sp. CPB 7
JQ997524	523	19	523	0	97%	97%	225031498	Bacteria	Firmicutes	Bacillaceae	Bacillus sp. E-163
JQ997525	692	2	253	2E-103	94%	94%	195548081	Bacteria	Firmicutes	Bacillaceae	Bacillus sp. EK-1
JQ997526	379	17	334	2E-161	99%	99%	222350117	Bacteria	Firmicutes	Bacillaceae	Bacillus sp. F2-1
JQ997527	552	19	548	0	97%	97%	281487256	Bacteria	Firmicutes	Bacillaceae	Bacillus sp. H3B7
JQ997528	257	5	203	8E-99	100%	100%	295815415	Bacteria	Firmicutes	Bacillaceae	Bacillus sp. I_B8
JQ997529	377	5	344	1E-148	95%	95%	193795485	Bacteria	Firmicutes	Bacillaceae	Bacillus sp. ISO_02_Chiprana
JQ997530	473	5	242	2E-87	93%	93%	116089632	Bacteria	Firmicutes	Bacillaceae	Bacillus sp. m3-13
JQ997531	541	5	540	0	99%	99%	257751815	Bacteria	Firmicutes	Bacillaceae	Bacillus sp. MB63
JQ997533	551	5	523	0	96%	96%	158562722	Bacteria	Firmicutes	Bacillaceae	Bacillus sp. NP16
JQ997534	337	4	305	5E-132	95%	95%	291196850	Bacteria	Firmicutes	Bacillaceae	Bacillus sp. OU-A7
JQ997535	408	18	273	5E-73	88%	88%	289186779	Bacteria	Firmicutes	Bacillaceae	Bacillus sp. QT14
JQ997536	647	24	205	6E-34	84%	84%	21541801	Bacteria	Firmicutes	Bacillaceae	Bacillus sp. RiMSX30
JQ997537	531	13	495	0	95%	95%	189307029	Bacteria	Firmicutes	Bacillaceae	Bacillus sp. SL177
JQ997538	569	26	565	0	94%	94%	223048105	Bacteria	Firmicutes	Bacillaceae	Bacillus sp. T2830
JQ997539	307	3	276	7E-120	96%	96%	295322911	Bacteria	Firmicutes	Bacillaceae	Bacillus sp. T47_2010_
JQ997540	553	16	553	0	99%	99%	86279623	Bacteria	Firmicutes	Bacillaceae	Bacillus sp. YIM DKMY117-2
JQ997541	538	16	485	0	93%	93%	143425	Bacteria	Firmicutes	Bacillaceae	Bacillus subtilis
JQ997542	542	17	539	0	93%	93%	223972589	Bacteria	Firmicutes	Bacillaceae	Bacillus trypoxylicola
JQ997543	585	22	516	0	94%	94%	283486718	Bacteria	Firmicutes	Bacillaceae	Marinococcus sp. 2009
JQ997544	526	5	523	0	98%	98%	283486730	Bacteria	Firmicutes	Bacillaceae	Marinococcus sp. 2046
JQ997545	556	34	554	0	99%	99%	45934548	Bacteria	Firmicutes	Bacillaceae	Marinococcus sp. GSP32
JQ997550	508	4	474	7E-152	88%	88%	157644545	Bacteria	Firmicutes	Bacillaceae	uncultured Bacillus sp.
JQ997552	560	18	555	0	96%	96%	189514178	Bacteria	Firmicutes	Bacillaceae	uncultured Bacillus sp.
JQ997551	546	17	543	0	94%	94%	195971876	Bacteria	Firmicutes	Bacillaceae	uncultured Bacillus sp.
JQ997547	445	17	400	2E-167	95%	95%	238836028	Bacteria	Firmicutes	Bacillaceae	uncultured Bacillus sp.
JQ997549	490	17	211	3E-96	100%	100%	282765781	Bacteria	Firmicutes	Bacillaceae	uncultured Bacillus sp.
JQ997546	401	5	291	7E-146	100%	100%	284428514	Bacteria	Firmicutes	Bacillaceae	uncultured Bacillus sp.
JQ997553	391	5	358	9E-140	92%	92%	291289395	Bacteria	Firmicutes	n	Alkalilactibacillus ikkense
JQ997554	296	24	264	2E-105	96%	96%	220897617	Bacteria	Firmicutes	n	uncultured Bacillales bacterium
JQ997555	412	5	380	1E-168	95%	95%	219857497	Bacteria	Firmicutes	Paenibacillaceae	Paenibacillus granivorans
JQ997556	537	18	536	0	95%	95%	154818670	Bacteria	Firmicutes	Paenibacillaceae	Saccharibacillus kuerlensis
JQ997557	384	18	327	1E-133	95%	95%	254972685	Bacteria	Firmicutes	Paenibacillaceae	uncultured Paenibacillus sp.
JQ997558	394	20	360	3E-174	99%	99%	293633232	Bacteria	Firmicutes	Planococcaceae	Planococcus maitriensis
JQ997559	546	3	540	0	95%	95%	292386047	Bacteria	Firmicutes	Planococcaceae	Planococcus maritimus
JQ997560	337	31	292	1E-128	99%	99%	262410342	Bacteria	Firmicutes	Planococcaceae	Planococcus psychrotoleratus
JQ997561	311	4	281	1E-87	90%	90%	24431214	Bacteria	Firmicutes	Planococcaceae	Planococcus sp. 1-1
JQ997562	524	5	413	0	96%	96%	78033696	Bacteria	Firmicutes	Planococcaceae	Planococcus sp. 3059
JQ997563	521	5	454	3E-161	90%	90%	219944562	Bacteria	Firmicutes	Planococcaceae	Planococcus sp. B-2
JQ997564	547	5	532	0	99%	99%	255689464	Bacteria	Firmicutes	Planococcaceae	Planococcus sp. BSw21500
JQ997565	495	17	387	3E-160	95%	95%	291293772	Bacteria	Firmicutes	Planococcaceae	Planococcus sp. enrichment culture clone B2-1
JQ997566	544	18	544	0	99%	99%	291419708	Bacteria	Firmicutes	Planococcaceae	Planococcus sp. JDN
JQ997567	602	18	576	0	89%	89%	270048079	Bacteria	Firmicutes	Planococcaceae	Planococcus sp. ljh-25
JQ997568	570	17	546	0	94%	94%	54303743	Bacteria	Firmicutes	Planococcaceae	Planococcus sp. NPO-JL-69
JQ997569	348	5	302	1E-128	95%	95%	289629721	Bacteria	Firmicutes	Planococcaceae	Planococcus sp. S118
JQ997570	552	5	552	0	99%	99%	89257980	Bacteria	Firmicutes	Planococcaceae	Planococcus sp. TSBY-25
JQ997571	547	1	522	0	91%	91%	209981399	Bacteria	Firmicutes	Planococcaceae	Planococcus sp. Zao-A
JQ997572	491	12	435	0	99%	99%	240248421	Bacteria	Firmicutes	Planococcaceae	Planomicrobium koreense
JQ997573	288	5	244	1E-97	94%	94%	262410321	Bacteria	Firmicutes	Planococcaceae	Planomicrobium okeanokoites
JQ997574	558	5	558	0	98%	98%	260100820	Bacteria	Firmicutes	Planococcaceae	Planomicrobium psychrophilum
JQ997575	573	21	569	0	95%	95%	256274955	Bacteria	Firmicutes	Planococcaceae	Planomicrobium sp. G-5
JQ997576	565	17	509	0	99%	99%	209917046	Bacteria	Firmicutes	Planococcaceae	Planomicrobium sp. ISL-41
JQ997577	295	23	239	1E-102	99%	99%	294992062	Bacteria	Firmicutes	Planococcaceae	Planomicrobium sp. MJ426
JQ997578	559	18	527	0	99%	99%	198250506	Bacteria	Firmicutes	Planococcaceae	Planomicrobium sp. RCML-41
JQ997579	437	4	361	0	99%	99%	270282461	Bacteria	Firmicutes	Planococcaceae	Sporosarcina sp. 4-76
JQ997580	251	5	206	2E-100	100%	100%	257043974	Bacteria	Firmicutes	Planococcaceae	Sporosarcina sp. LI4
JQ997581	571	24	567	0	96%	96%	154757245	Bacteria	Firmicutes	Planococcaceae	uncultured Jeotgalibacillus sp.

JQ997582	433	5	372	2E-147	93%	93%	255976610	Bacteria	Firmicutes	Planococcaceae	uncultured Planococcaceae bacterium
JQ997583	440	17	282	1E-68	86%	86%	260874923	Bacteria	Firmicutes	Planococcaceae	uncultured Planococcaceae bacterium
JQ997588	572	18	569	0	96%	96%	154757011	Bacteria	Firmicutes	Planococcaceae	uncultured Planococcus sp.
JQ997585	456	5	410	0	98%	98%	161702580	Bacteria	Firmicutes	Planococcaceae	uncultured Planococcus sp.
JQ997587	538	4	538	0	100%	100%	187711733	Bacteria	Firmicutes	Planococcaceae	uncultured Planococcus sp.
JQ997586	466	4	411	0	100%	100%	292485796	Bacteria	Firmicutes	Planococcaceae	uncultured Planococcus sp.
JQ997584	377	17	328	2E-146	97%	97%	292485817	Bacteria	Firmicutes	Planococcaceae	uncultured Planococcus sp.
JQ997590	550	5	533	0	96%	96%	161702576	Bacteria	Firmicutes	Planococcaceae	uncultured Planomicrobium sp.
JQ997589	482	26	436	1E-159	93%	93%	161702610	Bacteria	Firmicutes	Planococcaceae	uncultured Planomicrobium sp.
JQ997591	348	24	261	6E-96	94%	94%	74052526	Bacteria	Firmicutes	Sporolactobacillaceae	Sinobaca qinghaiensis
JQ997592	573	23	518	0	98%	98%	219846053	Bacteria	Firmicutes	Staphylococcaceae	Jeotgalicoccus halotolerans
JQ997593	561	21	528	0	96%	96%	290783602	Bacteria	Firmicutes	Staphylococcaceae	Jeotgalicoccus nanhaiensis
JQ997594	561	29	558	0	92%	92%	219846054	Bacteria	Firmicutes	Staphylococcaceae	Jeotgalicoccus psychrophilus
JQ997595	557	21	539	0	91%	91%	219373967	Bacteria	Firmicutes	Staphylococcaceae	Jeotgalicoccus sp. YD2-57
JQ997596	547	5	543	0	99%	99%	86279588	Bacteria	Firmicutes	Staphylococcaceae	Jeotgalicoccus sp. YIM KMY9-1
JQ997597	536	19	514	0	96%	96%	21586494	Bacteria	Firmicutes	Staphylococcaceae	Macrococcus brunensis
JQ997598	374	26	326	1E-127	95%	95%	294769193	Bacteria	Firmicutes	Staphylococcaceae	Macrococcus caseolyticus
JQ997599	751	5	291	2E-134	97%	97%	283858007	Bacteria	Firmicutes	Staphylococcaceae	Macrococcus sp. AMGM1
JQ997600	563	14	366	2E-162	96%	96%	194368442	Bacteria	Firmicutes	Staphylococcaceae	Salinicoccus sp. B-WPyS1
JQ997601	487	18	416	0	96%	96%	269313994	Bacteria	Firmicutes	Staphylococcaceae	Staphylococcus arlettae
JQ997603	347	5	275	1E-132	99%	99%	219809027	Bacteria	Firmicutes	Staphylococcaceae	Staphylococcus sp. BQN1P-02d
JQ997604	553	18	546	0	99%	99%	295443962	Bacteria	Firmicutes	Staphylococcaceae	Staphylococcus sp. NCCP-163
JQ997605	316	5	271	2E-131	99%	99%	242027442	Bacteria	Firmicutes	Staphylococcaceae	Staphylococcus sp. NII-116
JQ997612	614	5	86	4E-31	99%	99%	75753540	Bacteria	Firmicutes	Staphylococcaceae	uncultured Staphylococcus sp.
JQ997609	503	15	449	0	97%	97%	238835780	Bacteria	Firmicutes	Staphylococcaceae	uncultured Staphylococcus sp.
JQ997608	471	166	410	2E-122	100%	100%	238835868	Bacteria	Firmicutes	Staphylococcaceae	uncultured Staphylococcus sp.
JQ997610	540	5	533	0	95%	95%	238836019	Bacteria	Firmicutes	Staphylococcaceae	uncultured Staphylococcus sp.
JQ997611	545	18	541	0	96%	96%	238836032	Bacteria	Firmicutes	Staphylococcaceae	uncultured Staphylococcus sp.
JQ997607	349	21	314	4E-128	96%	96%	238836130	Bacteria	Firmicutes	Staphylococcaceae	uncultured Staphylococcus sp.
JQ997606	272	18	190	2E-84	100%	100%	259120992	Bacteria	Firmicutes	Staphylococcaceae	uncultured Staphylococcus sp.
JQ997613	707	30	134	3E-37	95%	95%	20385618	Bacteria	Firmicutes	Carnobacteriaceae	Carnobacterium mobile
JQ997614	500	18	441	0	100%	100%	257167997	Bacteria	Firmicutes	Carnobacteriaceae	Carnobacterium sp. 12266/2009
JQ997615	295	14	250	7E-120	100%	100%	49617305	Bacteria	Firmicutes	Carnobacteriaceae	Carnobacterium sp. BM-8
JQ997616	514	24	399	0	99%	99%	284022001	Bacteria	Firmicutes	Carnobacteriaceae	Trichococcus sp. EX-07
JQ997617	283	16	224	2E-89	96%	96%	225031786	Bacteria	Firmicutes	Carnobacteriaceae	uncultured Alkalibacterium sp.
JQ997619	551	5	477	0	99%	99%	292485813	Bacteria	Firmicutes	Carnobacteriaceae	uncultured Carnobacterium sp.
JQ997618	249	17	217	4E-96	99%	99%	295147519	Bacteria	Firmicutes	Carnobacteriaceae	uncultured Carnobacterium sp.
JQ997620	483	19	387	2E-88	84%	84%	223048104	Bacteria	Firmicutes	Enterococcaceae	Vagococcus sp. T4130
JQ997621	508	5	475	0	96%	96%	294438969	Bacteria	Firmicutes	Lactobacillaceae	Lactobacillus acidophilus
JQ997622	520	17	518	0	94%	94%	265679044	Bacteria	Firmicutes	Lactobacillaceae	Lactobacillus amylolyticus
JQ997625	343	5	195	5E-67	91%	91%	226377546	Bacteria	Firmicutes	Lactobacillaceae	Lactobacillus casei
JQ997627	482	5	434	0	100%	100%	292673285	Bacteria	Firmicutes	Lactobacillaceae	Lactobacillus curvatus
JQ997634	520	18	475	0	100%	100%	121581899	Bacteria	Firmicutes	Lactobacillaceae	Lactobacillus delbrueckii
JQ997629	383	5	344	5E-177	100%	100%	163954906	Bacteria	Firmicutes	Lactobacillaceae	Lactobacillus delbrueckii
JQ997631	494	24	492	0	93%	93%	237512278	Bacteria	Firmicutes	Lactobacillaceae	Lactobacillus delbrueckii
JQ997635	529	5	527	0	100%	100%	237512288	Bacteria	Firmicutes	Lactobacillaceae	Lactobacillus delbrueckii
JQ997628	350	5	306	5E-152	99%	99%	292673284	Bacteria	Firmicutes	Lactobacillaceae	Lactobacillus delbrueckii
JQ997632	513	5	510	0	100%	100%	292673288	Bacteria	Firmicutes	Lactobacillaceae	Lactobacillus delbrueckii
JQ997633	518	18	515	0	99%	99%	294438970	Bacteria	Firmicutes	Lactobacillaceae	Lactobacillus delbrueckii
JQ997630	480	18	438	0	99%	99%	294938080	Bacteria	Firmicutes	Lactobacillaceae	Lactobacillus delbrueckii
JQ997636	536	17	530	0	96%	96%	218775050	Bacteria	Firmicutes	Lactobacillaceae	Lactobacillus equicursoris
JQ997637	491	23	432	0	100%	100%	295149327	Bacteria	Firmicutes	Lactobacillaceae	Lactobacillus fermentum
JQ997652	425	5	392	1E-133	91%	91%	254305415	Bacteria	Firmicutes	Lactobacillaceae	Lactobacillus paracasei
JQ997664	557	18	534	0	96%	96%	37496510	Bacteria	Firmicutes	Lactobacillaceae	Lactobacillus rhamnosus
JQ997653	248	4	205	7E-99	100%	100%	57864919	Bacteria	Firmicutes	Lactobacillaceae	Lactobacillus rhamnosus
JQ997656	487	5	425	0	96%	96%	127905849	Bacteria	Firmicutes	Lactobacillaceae	Lactobacillus rhamnosus
JQ997661	542	20	497	1E-174	90%	90%	285201574	Bacteria	Firmicutes	Lactobacillaceae	Lactobacillus rhamnosus
JQ997657	502	18	442	2E-167	92%	92%	285201674	Bacteria	Firmicutes	Lactobacillaceae	Lactobacillus rhamnosus
JQ997654	359	4	309	8E-140	96%	96%	285201707	Bacteria	Firmicutes	Lactobacillaceae	Lactobacillus rhamnosus
JQ997655	407	18	297	2E-126	96%	96%	285201711	Bacteria	Firmicutes	Lactobacillaceae	Lactobacillus rhamnosus

JQ997662	553	5	505	0	99%	99%	285201754	Bacteria	Firmicutes	Lactobacillaceae	Lactobacillus rhamnosus
JQ997658	538	4	492	0	100%	100%	288812699	Bacteria	Firmicutes	Lactobacillaceae	Lactobacillus rhamnosus
JQ997659	538	23	534	0	100%	100%	290760129	Bacteria	Firmicutes	Lactobacillaceae	Lactobacillus rhamnosus
JQ997660	539	5	539	0	93%	93%	290784161	Bacteria	Firmicutes	Lactobacillaceae	Lactobacillus rhamnosus
JQ997663	555	171	555	0	98%	98%	294714412	Bacteria	Firmicutes	Lactobacillaceae	Lactobacillus rhamnosus
JQ997665	284	4	240	6E-120	100%	100%	288225761	Bacteria	Firmicutes	Lactobacillaceae	Lactobacillus salivarius
JQ997666	541	5	515	0	94%	94%	292385841	Bacteria	Firmicutes	Lactobacillaceae	Lactobacillus salivarius
JQ997667	436	5	404	7E-166	94%	94%	15408520	Bacteria	Firmicutes	Lactobacillaceae	Lactobacillus sp. B5406
JQ997668	542	18	437	0	98%	98%	55418398	Bacteria	Firmicutes	Lactobacillaceae	Lactobacillus sp. BCRC16000
JQ997669	399	3	190	2E-92	100%	100%	285171057	Bacteria	Firmicutes	Lactobacillaceae	Lactobacillus sp. oral taxon 461
JQ997670	470	18	397	0	99%	99%	285802972	Bacteria	Firmicutes	Lactobacillaceae	Lactobacillus sp. oral taxon 461
JQ997671	371	5	324	2E-150	98%	98%	38327309	Bacteria	Firmicutes	Lactobacillaceae	Lactobacillus sp. RA2062
JQ997672	542	18	542	0	92%	92%	38373968	Bacteria	Firmicutes	Lactobacillaceae	Lactobacillus sp. rennanqilfy2
JQ997673	493	5	447	0	94%	94%	295394120	Bacteria	Firmicutes	Lactobacillaceae	Lactobacillus sp. TAB-26
JQ997674	272	5	228	1E-87	93%	93%	188572057	Bacteria	Firmicutes	Lactobacillaceae	Lactobacillus vaginalis
JQ997675	525	154	474	1E-80	85%	85%	151384635	Bacteria	Firmicutes	Lactobacillaceae	uncultured Lactobacillus sp.
JQ997676	532	18	528	0	94%	94%	295646945	Bacteria	Firmicutes	Lactobacillaceae	uncultured Lactobacillus sp.
JQ997678	296	4	239	2E-119	100%	100%	21591599	Bacteria	Firmicutes	Streptococcaceae	Lactococcus lactis
JQ997677	276	4	227	5E-111	100%	100%	124244807	Bacteria	Firmicutes	Streptococcaceae	Lactococcus lactis
JQ997682	534	18	499	0	99%	99%	206600409	Bacteria	Firmicutes	Streptococcaceae	Lactococcus lactis
JQ997683	540	16	537	0	99%	99%	225029381	Bacteria	Firmicutes	Streptococcaceae	Lactococcus lactis
JQ997680	400	8	237	2E-77	91%	91%	225029671	Bacteria	Firmicutes	Streptococcaceae	Lactococcus lactis
JQ997681	508	18	428	0	100%	100%	254971972	Bacteria	Firmicutes	Streptococcaceae	Lactococcus lactis
JQ997679	313	4	271	4E-137	100%	100%	285803105	Bacteria	Firmicutes	Streptococcaceae	Lactococcus lactis
JQ997684	541	17	537	0	100%	100%	294828947	Bacteria	Firmicutes	Streptococcaceae	Lactococcus lactis
JQ997685	544	5	526	0	100%	100%	295815580	Bacteria	Firmicutes	Streptococcaceae	Lactococcus lactis
JQ997696	286	1	286	5E-147	100%	100%	11991762	Bacteria	Firmicutes	Streptococcaceae	Streptococcus mutans
JQ997686	342	5	293	1E-88	88%	88%	285177464	Bacteria	Firmicutes	Streptococcaceae	Streptococcus constellatus
JQ997691	555	18	550	0	97%	97%	55163273	Bacteria	Firmicutes	Streptococcaceae	Streptococcus cristatus
JQ997690	543	5	477	0	100%	100%	284451152	Bacteria	Firmicutes	Streptococcaceae	Streptococcus cristatus
JQ997689	441	4	289	3E-145	100%	100%	285177736	Bacteria	Firmicutes	Streptococcaceae	Streptococcus cristatus
JQ997688	375	18	354	2E-170	99%	99%	285178071	Bacteria	Firmicutes	Streptococcaceae	Streptococcus cristatus
JQ997687	283	4	221	1E-107	100%	100%	285178094	Bacteria	Firmicutes	Streptococcaceae	Streptococcus cristatus
JQ997692	556	3	551	0	100%	100%	295002588	Bacteria	Firmicutes	Streptococcaceae	Streptococcus cristatus
JQ997693	529	5	256	3E-121	98%	98%	295002589	Bacteria	Firmicutes	Streptococcaceae	Streptococcus gordonii
JQ997697	547	19	547	0	100%	100%	295002592	Bacteria	Firmicutes	Streptococcaceae	Streptococcus mutans
JQ997699	525	18	480	0	98%	98%	290759894	Bacteria	Firmicutes	Streptococcaceae	Streptococcus oralis
JQ997700	531	17	527	0	98%	98%	295002593	Bacteria	Firmicutes	Streptococcaceae	Streptococcus oralis
JQ997701	484	24	432	0	98%	98%	285801983	Bacteria	Firmicutes	Streptococcaceae	Streptococcus parasanguinis
JQ997702	491	5	453	0	98%	98%	285802105	Bacteria	Firmicutes	Streptococcaceae	Streptococcus parasanguinis
JQ997703	530	22	524	0	100%	100%	290759892	Bacteria	Firmicutes	Streptococcaceae	Streptococcus parasanguinis
JQ997704	526	17	470	0	100%	100%	290759899	Bacteria	Firmicutes	Streptococcaceae	Streptococcus pneumoniae
JQ997707	300	18	260	1E-121	100%	100%	24474984	Bacteria	Firmicutes	Streptococcaceae	Streptococcus salivarius
JQ997713	538	21	535	0	99%	99%	171191150	Bacteria	Firmicutes	Streptococcaceae	Streptococcus salivarius
JQ997714	539	15	537	0	99%	99%	208657445	Bacteria	Firmicutes	Streptococcaceae	Streptococcus salivarius
JQ997711	389	18	344	4E-168	100%	100%	284176962	Bacteria	Firmicutes	Streptococcaceae	Streptococcus salivarius
JQ997708	325	3	176	8E-85	100%	100%	285194533	Bacteria	Firmicutes	Streptococcaceae	Streptococcus salivarius
JQ997715	550	5	545	0	98%	98%	285194543	Bacteria	Firmicutes	Streptococcaceae	Streptococcus salivarius
JQ997705	275	18	244	1E-112	100%	100%	285194556	Bacteria	Firmicutes	Streptococcaceae	Streptococcus salivarius
JQ997709	326	3	269	3E-128	98%	98%	285194587	Bacteria	Firmicutes	Streptococcaceae	Streptococcus salivarius
JQ997706	284	51	251	9E-84	95%	95%	285194597	Bacteria	Firmicutes	Streptococcaceae	Streptococcus salivarius
JQ997712	401	17	351	5E-88	86%	86%	285194615	Bacteria	Firmicutes	Streptococcaceae	Streptococcus salivarius
JQ997710	337	18	266	8E-105	95%	95%	285194695	Bacteria	Firmicutes	Streptococcaceae	Streptococcus salivarius
JQ997716	565	18	563	0	94%	94%	295002596	Bacteria	Firmicutes	Streptococcaceae	Streptococcus salivarius
JQ997719	439	5	120	2E-28	89%	89%	11526815	Bacteria	Firmicutes	Streptococcaceae	Streptococcus sp. ES11
JQ997721	434	15	317	5E-133	95%	95%	285203360	Bacteria	Firmicutes	Streptococcaceae	Streptococcus sp. oral taxon C65
JQ997725	538	18	421	0	96%	96%	285203400	Bacteria	Firmicutes	Streptococcaceae	Streptococcus sp. oral taxon C65
JQ997724	506	2	418	0	97%	97%	285203424	Bacteria	Firmicutes	Streptococcaceae	Streptococcus sp. oral taxon C65
JQ997722	472	4	423	8E-141	89%	89%	285203520	Bacteria	Firmicutes	Streptococcaceae	Streptococcus sp. oral taxon C65
JQ997720	322	4	270	6E-126	98%	98%	285203550	Bacteria	Firmicutes	Streptococcaceae	Streptococcus sp. oral taxon C65

JQ997726	564	5	502	6E-123	85%	85%	285203608	Bacteria	Firmicutes	Streptococcaceae	Streptococcus sp. oral taxon C65
JQ997723	501	4	447	0	96%	96%	285203671	Bacteria	Firmicutes	Streptococcaceae	Streptococcus sp. oral taxon C65
JQ997727	333	21	288	2E-130	99%	99%	285206259	Bacteria	Firmicutes	Streptococcaceae	Streptococcus sp. oral taxon G59
JQ997740	513	5	440	0	99%	99%	28274377	Bacteria	Firmicutes	Streptococcaceae	Streptococcus vestibularis
JQ997739	337	4	271	5E-137	100%	100%	223470134	Bacteria	Firmicutes	Streptococcaceae	Streptococcus vestibularis
JQ997738	286	2	247	1E-121	99%	99%	285159297	Bacteria	Firmicutes	Streptococcaceae	Streptococcus vestibularis
JQ997741	530	5	402	0	100%	100%	285159329	Bacteria	Firmicutes	Streptococcaceae	Streptococcus vestibularis
JQ999504	340	23	280	1E-127	99%	99%	269911992	Bacteria	Firmicutes	Streptococcaceae	uncultured Streptococcaceae bacterium
JQ997757	548	17	542	0	97%	97%	15593129	Bacteria	Firmicutes	Streptococcaceae	uncultured Streptococcus sp.
JQ997753	540	5	539	0	99%	99%	15593133	Bacteria	Firmicutes	Streptococcaceae	uncultured Streptococcus sp.
JQ997746	400	23	351	3E-159	98%	98%	60501121	Bacteria	Firmicutes	Streptococcaceae	uncultured Streptococcus sp.
JQ997754	544	61	539	0	100%	100%	60501133	Bacteria	Firmicutes	Streptococcaceae	uncultured Streptococcus sp.
JQ997743	337	5	243	4E-103	96%	96%	60501533	Bacteria	Firmicutes	Streptococcaceae	uncultured Streptococcus sp.
JQ997758	550	18	518	0	100%	100%	60501679	Bacteria	Firmicutes	Streptococcaceae	uncultured Streptococcus sp.
JQ997759	551	4	548	0	99%	99%	60501751	Bacteria	Firmicutes	Streptococcaceae	uncultured Streptococcus sp.
JQ997742	241	5	187	9E-88	99%	99%	77819579	Bacteria	Firmicutes	Streptococcaceae	uncultured Streptococcus sp.
JQ997749	482	5	410	0	98%	98%	85813098	Bacteria	Firmicutes	Streptococcaceae	uncultured Streptococcus sp.
JQ997747	410	17	354	4E-168	99%	99%	110613571	Bacteria	Firmicutes	Streptococcaceae	uncultured Streptococcus sp.
JQ997761	560	9	560	0	88%	88%	164453546	Bacteria	Firmicutes	Streptococcaceae	uncultured Streptococcus sp.
JQ997756	547	18	509	0	98%	98%	171467463	Bacteria	Firmicutes	Streptococcaceae	uncultured Streptococcus sp.
JQ997748	414	5	349	2E-161	97%	97%	189305804	Bacteria	Firmicutes	Streptococcaceae	uncultured Streptococcus sp.
JQ997745	379	4	328	1E-168	100%	100%	189305923	Bacteria	Firmicutes	Streptococcaceae	uncultured Streptococcus sp.
JQ997744	341	5	294	3E-139	98%	98%	238914954	Bacteria	Firmicutes	Streptococcaceae	uncultured Streptococcus sp.
JQ997752	539	17	502	0	100%	100%	254972501	Bacteria	Firmicutes	Streptococcaceae	uncultured Streptococcus sp.
JQ997750	532	18	530	0	100%	100%	259221069	Bacteria	Firmicutes	Streptococcaceae	uncultured Streptococcus sp.
JQ997751	534	16	351	2E-157	97%	97%	281332796	Bacteria	Firmicutes	Streptococcaceae	uncultured Streptococcus sp.
JQ997760	554	4	548	0	96%	96%	295646938	Bacteria	Firmicutes	Streptococcaceae	uncultured Streptococcus sp.
JQ997755	544	18	544	0	99%	99%	295646981	Bacteria	Firmicutes	Streptococcaceae	uncultured Streptococcus sp.
JQ997770	495	19	439	6E-177	94%	94%	148717062	Bacteria	Firmicutes	n	uncultured Bacilli bacterium
JQ997775	545	370	487	1E-49	98%	98%	154185054	Bacteria	Firmicutes	n	uncultured Bacilli bacterium
JQ997768	473	15	404	0	98%	98%	154186406	Bacteria	Firmicutes	n	uncultured Bacilli bacterium
JQ997762	283	5	251	8E-124	100%	100%	154186415	Bacteria	Firmicutes	n	uncultured Bacilli bacterium
JQ997763	310	7	277	2E-110	94%	94%	154187391	Bacteria	Firmicutes	n	uncultured Bacilli bacterium
JQ997769	478	62	397	5E-153	96%	96%	154187409	Bacteria	Firmicutes	n	uncultured Bacilli bacterium
JQ997767	468	4	407	0	97%	97%	154187417	Bacteria	Firmicutes	n	uncultured Bacilli bacterium
JQ997771	532	5	531	0	97%	97%	154188162	Bacteria	Firmicutes	n	uncultured Bacilli bacterium
JQ997766	437	6	391	6E-152	93%	93%	154188901	Bacteria	Firmicutes	n	uncultured Bacilli bacterium
JQ997774	542	5	538	0	100%	100%	154189837	Bacteria	Firmicutes	n	uncultured Bacilli bacterium
JQ997772	540	15	531	2E-172	88%	88%	154191244	Bacteria	Firmicutes	n	uncultured Bacilli bacterium
JQ997777	547	5	547	0	99%	99%	154193192	Bacteria	Firmicutes	n	uncultured Bacilli bacterium
JQ997764	403	5	352	9E-180	100%	100%	154193211	Bacteria	Firmicutes	n	uncultured Bacilli bacterium
JQ997773	540	18	487	0	94%	94%	154195887	Bacteria	Firmicutes	n	uncultured Bacilli bacterium
JQ997765	432	34	369	4E-158	97%	97%	154198794	Bacteria	Firmicutes	n	uncultured Bacilli bacterium
JQ997776	546	18	540	0	98%	98%	154198888	Bacteria	Firmicutes	n	uncultured Bacilli bacterium
JQ997778	385	18	287	2E-136	100%	100%	171336058	Bacteria	Firmicutes	Clostridiaceae	Butyricicoccus pullicaecorum
JQ997779	527	15	497	0	95%	95%	166063935	Bacteria	Firmicutes	Clostridiaceae	Clostridiaceae bacterium SK082
JQ997780	583	18	399	1E-159	94%	94%	265678940	Bacteria	Firmicutes	Clostridiaceae	Clostridium nexile
JQ997781	338	17	306	3E-104	91%	91%	254841672	Bacteria	Firmicutes	Clostridiaceae	Clostridium perfringens
JQ997783	533	19	470	0	100%	100%	283945409	Bacteria	Firmicutes	Clostridiaceae	Clostridium perfringens
JQ997782	383	13	328	2E-156	99%	99%	294799804	Bacteria	Firmicutes	Clostridiaceae	Clostridium perfringens
JQ997784	348	3	260	4E-113	96%	96%	47558861	Bacteria	Firmicutes	Clostridiaceae	Clostridium saccharolyticum
JQ997785	465	5	431	0	96%	96%	269854738	Bacteria	Firmicutes	Clostridiaceae	Clostridium sp. 4-2a
JQ997786	650	4	76	2E-28	100%	100%	238769132	Bacteria	Firmicutes	Clostridiaceae	Clostridium sp. F-02
JQ997787	258	1	217	1E-106	100%	100%	295646952	Bacteria	Firmicutes	Clostridiaceae	uncultured Clostridium sp.
JQ997788	433	24	285	1E-133	100%	100%	91093763	Bacteria	Firmicutes	Eubacteriaceae	Eubacterium tenue
JQ997789	491	16	421	2E-162	93%	93%	285162551	Bacteria	Firmicutes	Lachnospiraceae	Lachnospiraceae bacterium oral taxon 107
JQ997790	299	18	256	2E-115	99%	99%	30908820	Bacteria	Firmicutes	Lachnospiraceae	Lachnospiraceae genomosp. C1
JQ997791	322	18	278	9E-119	97%	97%	110555124	Bacteria	Firmicutes	Lachnospiraceae	Robinsoniella peoriensis
JQ997793	484	4	412	0	98%	98%	154190788	Bacteria	Firmicutes	Lachnospiraceae	uncultured Lachnospiraceae bacterium
JQ997795	547	29	434	3E-165	93%	93%	154191505	Bacteria	Firmicutes	Lachnospiraceae	uncultured Lachnospiraceae bacterium

JQ997794	527	6	508	0	95%	95%	154194307	Bacteria	Firmicutes	Lachnospiraceae	uncultured Lachnospiraceae bacterium
JQ997792	332	4	254	1E-122	99%	99%	253683955	Bacteria	Firmicutes	Lachnospiraceae	uncultured Lachnospiraceae bacterium
JQ997798	422	25	368	7E-156	96%	96%	262223663	Bacteria	Firmicutes	n	Flavonifractor plautii
JQ997796	248	16	216	6E-100	100%	100%	294799805	Bacteria	Firmicutes	n	Flavonifractor plautii
JQ997797	313	18	265	2E-121	99%	99%	294799812	Bacteria	Firmicutes	n	Flavonifractor plautii
JQ997799	380	18	348	1E-157	98%	98%	186915004	Bacteria	Firmicutes	n	uncultured Clostridiales bacterium
JQ997800	552	5	548	0	98%	98%	215981601	Bacteria	Firmicutes	n	uncultured Clostridiales bacterium
JQ997802	471	15	415	2E-171	94%	94%	293509177	Bacteria	Firmicutes	Ruminococcaceae	Ruminococcus sp. 316498/08
JQ997803	575	3	96	2E-38	99%	99%	85542635	Bacteria	Firmicutes	n	uncultured Clostridia bacterium
JQ997805	462	4	417	0	99%	99%	295315546	Bacteria	Firmicutes	Erysipelotrichaceae	Eubacterium cylindroides
JQ997806	543	17	508	0	97%	97%	119371525	Bacteria	Firmicutes	n	Firmicutes bacterium BL80
JQ997807	556	18	444	0	95%	95%	119371526	Bacteria	Firmicutes	n	Firmicutes bacterium EG14
JQ997868	472	22	315	1E-144	99%	99%	20975393	Bacteria	Firmicutes	n	Firmicutes str. C29
JQ998191	364	5	306	4E-118	92%	92%	237970917	Bacteria	Firmicutes	n	uncultured bacterium
JQ998874	542	24	541	0	92%	92%	237987786	Bacteria	Firmicutes	n	uncultured bacterium
JQ997826	515	5	463	0	97%	97%	118135996	Bacteria	Firmicutes	n	uncultured Firmicutes bacterium
JQ997821	422	17	378	6E-132	91%	91%	151936484	Bacteria	Firmicutes	n	uncultured Firmicutes bacterium
JQ997822	428	24	363	2E-146	95%	95%	156121565	Bacteria	Firmicutes	n	uncultured Firmicutes bacterium
JQ997823	430	5	325	4E-158	98%	98%	197131304	Bacteria	Firmicutes	n	uncultured Firmicutes bacterium
JQ997835	573	20	550	0	91%	91%	217038553	Bacteria	Firmicutes	n	uncultured Firmicutes bacterium
JQ997830	538	6	536	0	93%	93%	239835509	Bacteria	Firmicutes	n	uncultured Firmicutes bacterium
JQ997833	545	18	509	0	99%	99%	260072815	Bacteria	Firmicutes	n	uncultured Firmicutes bacterium
JQ997831	538	5	239	3E-116	100%	100%	290565065	Bacteria	Firmicutes	n	uncultured Firmicutes bacterium
JQ997813	327	1	280	2E-95	90%	90%	290565070	Bacteria	Firmicutes	n	uncultured Firmicutes bacterium
JQ997815	369	18	323	4E-158	100%	100%	291328355	Bacteria	Firmicutes	n	uncultured Firmicutes bacterium
JQ997816	371	18	331	1E-162	100%	100%	291329218	Bacteria	Firmicutes	n	uncultured Firmicutes bacterium
JQ997812	326	130	294	4E-68	96%	96%	291329291	Bacteria	Firmicutes	n	uncultured Firmicutes bacterium
JQ997819	401	18	343	6E-127	93%	93%	291329413	Bacteria	Firmicutes	n	uncultured Firmicutes bacterium
JQ997808	274	5	212	8E-104	100%	100%	291329502	Bacteria	Firmicutes	n	uncultured Firmicutes bacterium
JQ997834	547	10	542	0	96%	96%	291329543	Bacteria	Firmicutes	n	uncultured Firmicutes bacterium
JQ997824	464	18	413	0	100%	100%	291329590	Bacteria	Firmicutes	n	uncultured Firmicutes bacterium
JQ997829	537	5	537	0	97%	97%	291329777	Bacteria	Firmicutes	n	uncultured Firmicutes bacterium
JQ997814	341	7	265	8E-130	100%	100%	291329778	Bacteria	Firmicutes	n	uncultured Firmicutes bacterium
JQ997817	384	18	337	3E-164	100%	100%	291329796	Bacteria	Firmicutes	n	uncultured Firmicutes bacterium
JQ997825	481	23	305	7E-142	99%	99%	291330079	Bacteria	Firmicutes	n	uncultured Firmicutes bacterium
JQ997832	538	17	534	0	100%	100%	291330349	Bacteria	Firmicutes	n	uncultured Firmicutes bacterium
JQ997818	400	5	357	0	100%	100%	291330933	Bacteria	Firmicutes	n	uncultured Firmicutes bacterium
JQ997810	293	5	248	2E-120	99%	99%	291331108	Bacteria	Firmicutes	n	uncultured Firmicutes bacterium
JQ997809	286	18	182	7E-80	100%	100%	291331129	Bacteria	Firmicutes	n	uncultured Firmicutes bacterium
JQ997811	323	5	283	3E-143	100%	100%	291331435	Bacteria	Firmicutes	n	uncultured Firmicutes bacterium
JQ997827	524	14	506	0	92%	92%	291331838	Bacteria	Firmicutes	n	uncultured Firmicutes bacterium
JQ997820	416	116	371	1E-128	100%	100%	291332873	Bacteria	Firmicutes	n	uncultured Firmicutes bacterium
JQ997828	531	14	177	8E-62	95%	95%	291332981	Bacteria	Firmicutes	n	uncultured Firmicutes bacterium
JQ997836	420	5	372	4E-153	94%	94%	55418240	Bacteria	Firmicutes	n	uncultured low G+C Gram-positive bacterium
JQ997837	514	4	465	0	92%	92%	257480629	Bacteria	Firmicutes	Veillonellaceae	Selenomonas sputigena
JQ997840	546	16	404	0	100%	100%	285166114	Bacteria	Firmicutes	Veillonellaceae	Selenomonas sputigena
JQ997839	543	5	444	0	99%	99%	285166457	Bacteria	Firmicutes	Veillonellaceae	Selenomonas sputigena
JQ997838	528	18	477	0	98%	98%	285166459	Bacteria	Firmicutes	Veillonellaceae	Selenomonas sputigena
JQ997841	433	5	374	0	99%	99%	162846335	Bacteria	Firmicutes	Veillonellaceae	uncultured Selenomonas sp.
JQ997842	531	29	529	0	98%	98%	209781609	Bacteria	Firmicutes	Veillonellaceae	uncultured Veillonella sp.
JQ997843	412	15	357	3E-164	98%	98%	290759910	Bacteria	Firmicutes	Veillonellaceae	Veillonella dispar
JQ997844	522	5	494	0	97%	97%	291220264	Bacteria	Firmicutes	Veillonellaceae	Veillonella parvula
JQ997845	414	3	365	1E-128	91%	91%	295315569	Bacteria	Firmicutes	Veillonellaceae	Veillonella sp. oral clone 13-17
JQ997846	549	5	544	0	99%	99%	62910916	Bacteria	Firmicutes	Veillonellaceae	Veillonella sp. oral clone VeillG4
JQ997849	534	153	533	0	99%	99%	253684045	Bacteria	Fusobacteria	Fusobacteriaceae	uncultured Leptotrichia sp.
JQ997848	526	4	522	0	94%	94%	269979883	Bacteria	Fusobacteria	Fusobacteriaceae	uncultured Leptotrichia sp.
JQ997850	450	119	418	1E-149	99%	99%	265678963	Bacteria	Fusobacteria	n	Clostridium rectum
JQ997851	430	18	358	1E-148	95%	95%	198387333	Bacteria	n	n	bacterium 071021-ONK-SLIME-CHAB2
JQ997852	353	258	315	3E-20	100%	100%	295639957	Bacteria	n	n	bacterium EK-I90
JQ997853	511	9	57	7E-13	98%	98%	294883925	Bacteria	n	n	bacterium enrichment culture clone heteroA1_4W

JQ997854	336	24	285	1E-133	100%	100%	294883955	Bacteria	n	n	bacterium enrichment culture clone heteroA75_4W
JQ997855	539	4	248	4E-115	98%	98%	294883970	Bacteria	n	n	bacterium enrichment culture clone heteroB99_4W
JQ997856	301	86	210	2E-40	92%	92%	291419661	Bacteria	n	n	bacterium enrichment culture clone NAP-24
JQ997857	542	5	527	0	97%	97%	291419658	Bacteria	n	n	bacterium enrichment culture clone NAP-40
JQ997858	404	9	366	9E-120	89%	89%	289188106	Bacteria	n	n	bacterium enrichment culture clone SRC_DSC19
JQ997859	511	18	455	0	98%	98%	256665427	Bacteria	n	n	bacterium F3_2009_
JQ997860	578	22	523	0	92%	92%	66932766	Bacteria	n	n	bacterium ic1311
JQ997861	565	42	542	0	98%	98%	66932769	Bacteria	n	n	bacterium ic1337
JQ997862	541	5	540	0	99%	99%	254547235	Bacteria	n	n	bacterium Mlm3
JQ997863	313	25	281	6E-106	94%	94%	219660860	Bacteria	n	n	bacterium N159B.200
JQ997864	491	5	427	5E-178	94%	94%	219660887	Bacteria	n	n	bacterium N159G.614
JQ997865	537	19	467	0	97%	97%	39652444	Bacteria	n	n	bacterium PE03-7A27
JQ997866	485	5	239	3E-115	99%	99%	56790880	Bacteria	n	n	bacterium SN12-19
JQ997867	738	12	302	4E-141	98%	98%	37789225	Bacteria	n	n	extreme arid zone bacterium HX-IE13
JQ997869	332	18	265	3E-124	100%	100%	290782562	Bacteria	n	n	halophilic bacterium NAHalo1
JQ997870	537	23	532	0	100%	100%	258618269	Bacteria	n	n	intestinal bacterium CPA-20A
JQ997871	325	49	294	1E-112	97%	97%	209865500	Bacteria	n	n	iron-reducing bacterium enrichment culture clone HN31
JQ997872	639	5	364	9E-162	96%	96%	224796376	Bacteria	n	n	swine fecal bacterium RF2B-Pec19
JQ997873	543	18	540	0	99%	99%	26225064	Bacteria	n	n	swine manure bacterium RT-3A
JQ998078	324	18	294	3E-129	97%	97%	2117330	Bacteria	n	n	uncultured bacterium
JQ999214	698	9	267	3E-122	98%	98%	3901210	Bacteria	n	n	uncultured bacterium
JQ998451	456	4	413	0	95%	95%	14289547	Bacteria	n	n	uncultured bacterium
JQ999183	582	168	442	9E-77	88%	88%	14916017	Bacteria	n	n	uncultured bacterium
JQ998187	364	92	331	2E-106	96%	96%	18141101	Bacteria	n	n	uncultured bacterium
JQ998575	497	5	440	0	94%	94%	18644181	Bacteria	n	n	uncultured bacterium
JQ997900	250	5	195	2E-89	98%	98%	18644258	Bacteria	n	n	uncultured bacterium
JQ998694	526	5	521	0	99%	99%	18644580	Bacteria	n	n	uncultured bacterium
JQ998361	419	12	350	6E-152	96%	96%	19170730	Bacteria	n	n	uncultured bacterium
JQ997979	288	2	257	7E-95	92%	92%	19170737	Bacteria	n	n	uncultured bacterium
JQ999187	595	5	368	2E-178	98%	98%	19908568	Bacteria	n	n	uncultured bacterium
JQ999210	680	5	256	2E-118	98%	98%	21213945	Bacteria	n	n	uncultured bacterium
JQ998841	540	23	435	9E-161	92%	92%	22296508	Bacteria	n	n	uncultured bacterium
JQ998081	325	18	280	1E-87	90%	90%	25188103	Bacteria	n	n	uncultured bacterium
JQ998033	309	20	264	6E-106	96%	96%	32187181	Bacteria	n	n	uncultured bacterium
JQ997914	258	3	222	6E-95	96%	96%	38455463	Bacteria	n	n	uncultured bacterium
JQ998000	298	24	248	7E-105	98%	98%	40806477	Bacteria	n	n	uncultured bacterium
JQ997935	268	51	224	7E-55	90%	90%	45738714	Bacteria	n	n	uncultured bacterium
JQ998421	445	4	357	1E-144	94%	94%	50059448	Bacteria	n	n	uncultured bacterium
JQ998238	380	5	320	1E-163	100%	100%	50080902	Bacteria	n	n	uncultured bacterium
JQ998603	506	24	461	0	97%	97%	50404665	Bacteria	n	n	uncultured bacterium
JQ998196	366	5	259	7E-121	98%	98%	50982376	Bacteria	n	n	uncultured bacterium
JQ999042	555	18	508	0	93%	93%	54695040	Bacteria	n	n	uncultured bacterium
JQ998859	542	23	536	0	98%	98%	54695044	Bacteria	n	n	uncultured bacterium
JQ999145	567	18	564	0	89%	89%	55845936	Bacteria	n	n	uncultured bacterium
JQ998366	421	20	371	3E-180	99%	99%	56044224	Bacteria	n	n	uncultured bacterium
JQ998212	370	5	330	1E-147	96%	96%	57434350	Bacteria	n	n	uncultured bacterium
JQ998097	332	18	279	1E-128	99%	99%	60327442	Bacteria	n	n	uncultured bacterium
JQ998446	454	131	410	1E-129	97%	97%	60657399	Bacteria	n	n	uncultured bacterium
JQ997942	270	4	214	5E-96	97%	97%	60657422	Bacteria	n	n	uncultured bacterium
JQ999002	551	18	550	0	97%	97%	61620047	Bacteria	n	n	uncultured bacterium
JQ998533	484	5	434	3E-170	92%	92%	61620155	Bacteria	n	n	uncultured bacterium
JQ998608	507	18	443	0	97%	97%	62753119	Bacteria	n	n	uncultured bacterium
JQ999082	558	18	553	0	99%	99%	62753204	Bacteria	n	n	uncultured bacterium
JQ999139	566	5	560	0	99%	99%	62755107	Bacteria	n	n	uncultured bacterium
JQ998816	538	17	505	0	95%	95%	66736353	Bacteria	n	n	uncultured bacterium
JQ999043	555	17	553	0	96%	96%	66736372	Bacteria	n	n	uncultured bacterium
JQ998221	375	5	330	4E-118	92%	92%	66736401	Bacteria	n	n	uncultured bacterium
JQ998651	518	149	511	9E-161	95%	95%	66878800	Bacteria	n	n	uncultured bacterium
JQ998761	534	5	521	0	100%	100%	66878873	Bacteria	n	n	uncultured bacterium
JQ998960	548	11	543	0	93%	93%	70959309	Bacteria	n	n	uncultured bacterium

JQ998087	328	18	281	2E-131	99%	99%	70959311	Bacteria	n	n		uncultured bacterium
JQ998842	540	23	495	0	94%	94%	70959336	Bacteria	n	n		uncultured bacterium
JQ998875	543	5	540	0	95%	95%	70959358	Bacteria	n	n		uncultured bacterium
JQ998646	517	18	473	0	96%	96%	71089293	Bacteria	n	n		uncultured bacterium
JQ998429	448	5	381	4E-144	92%	92%	71739182	Bacteria	n	n		uncultured bacterium
JQ998860	542	5	532	0	95%	95%	73536369	Bacteria	n	n		uncultured bacterium
JQ998391	432	4	328	2E-147	97%	97%	74038672	Bacteria	n	n		uncultured bacterium
JQ998207	369	6	317	2E-111	91%	91%	74038684	Bacteria	n	n		uncultured bacterium
JQ997899	249	11	169	1E-76	100%	100%	75993096	Bacteria	n	n		uncultured bacterium
JQ998422	445	9	378	2E-176	97%	97%	77379338	Bacteria	n	n		uncultured bacterium
JQ997984	292	5	260	9E-119	97%	97%	78058230	Bacteria	n	n		uncultured bacterium
JQ997997	297	16	260	5E-111	97%	97%	81238650	Bacteria	n	n		uncultured bacterium
JQ998433	449	18	392	0	99%	99%	82400218	Bacteria	n	n		uncultured bacterium
JQ998183	363	5	331	2E-146	96%	96%	82400224	Bacteria	n	n		uncultured bacterium
JQ998248	382	7	333	4E-143	95%	95%	82400229	Bacteria	n	n		uncultured bacterium
JQ998395	434	17	311	2E-122	94%	94%	82548202	Bacteria	n	n		uncultured bacterium
JQ998239	380	24	348	1E-128	93%	93%	83940142	Bacteria	n	n		uncultured bacterium
JQ998664	521	5	498	0	96%	96%	84315971	Bacteria	n	n		uncultured bacterium
JQ998801	537	7	487	0	92%	92%	85001804	Bacteria	n	n		uncultured bacterium
JQ998004	299	4	254	3E-94	92%	92%	85001806	Bacteria	n	n		uncultured bacterium
JQ998762	534	2	533	0	99%	99%	85062530	Bacteria	n	n		uncultured bacterium
JQ998109	338	5	308	4E-152	99%	99%	85682833	Bacteria	n	n		uncultured bacterium
JQ998583	501	5	436	0	95%	95%	85718236	Bacteria	n	n		uncultured bacterium
JQ998973	549	5	548	0	95%	95%	85718244	Bacteria	n	n		uncultured bacterium
JQ998111	339	18	288	5E-137	100%	100%	87042319	Bacteria	n	n		uncultured bacterium
JQ998827	539	18	536	0	98%	98%	90297218	Bacteria	n	n		uncultured bacterium
JQ999070	557	1	544	0	92%	92%	90822834	Bacteria	n	n		uncultured bacterium
JQ999207	664	5	351	4E-170	98%	98%	90904047	Bacteria	n	n		uncultured bacterium
JQ999106	560	5	488	0	97%	97%	92087243	Bacteria	n	n		uncultured bacterium
JQ998447	454	5	404	0	99%	99%	92087329	Bacteria	n	n		uncultured bacterium
JQ998376	426	88	363	8E-136	99%	99%	92087349	Bacteria	n	n		uncultured bacterium
JQ999083	558	5	558	0	95%	95%	92087371	Bacteria	n	n		uncultured bacterium
JQ998679	524	24	522	0	96%	96%	92087379	Bacteria	n	n		uncultured bacterium
JQ998069	323	18	286	6E-121	96%	96%	92087412	Bacteria	n	n		uncultured bacterium
JQ999060	556	18	550	0	99%	99%	92087442	Bacteria	n	n		uncultured bacterium
JQ998680	524	16	522	0	98%	98%	92087477	Bacteria	n	n		uncultured bacterium
JQ998208	370	5	325	1E-148	97%	97%	92087504	Bacteria	n	n		uncultured bacterium
JQ998545	487	5	450	0	99%	99%	92087513	Bacteria	n	n		uncultured bacterium
JQ998665	521	18	486	0	99%	99%	92087807	Bacteria	n	n		uncultured bacterium
JQ998623	510	18	446	0	100%	100%	98975473	Bacteria	n	n		uncultured bacterium
JQ999195	609	107	523	3E-72	81%	81%	99643491	Bacteria	n	n		uncultured bacterium
JQ998848	541	19	531	0	94%	94%	102415912	Bacteria	n	n		uncultured bacterium
JQ998719	529	18	523	0	94%	94%	102415948	Bacteria	n	n		uncultured bacterium
JQ998355	417	18	367	5E-177	99%	99%	108946447	Bacteria	n	n		uncultured bacterium
JQ998177	361	18	305	4E-148	100%	100%	108946553	Bacteria	n	n		uncultured bacterium
JQ997965	282	4	224	5E-111	100%	100%	109141309	Bacteria	n	n		uncultured bacterium
JQ998828	539	4	535	0	96%	96%	109143113	Bacteria	n	n		uncultured bacterium
JQ998829	539	18	534	0	91%	91%	109143656	Bacteria	n	n		uncultured bacterium
JQ998202	368	3	325	1E-167	100%	100%	109144240	Bacteria	n	n		uncultured bacterium
JQ998489	468	3	435	0	100%	100%	109144608	Bacteria	n	n		uncultured bacterium
JQ998050	316	5	268	7E-135	100%	100%	109144972	Bacteria	n	n		uncultured bacterium
JQ998326	407	5	374	6E-142	92%	92%	109145027	Bacteria	n	n		uncultured bacterium
JQ998913	545	5	528	0	98%	98%	109145060	Bacteria	n	n		uncultured bacterium
JQ999084	558	19	558	0	100%	100%	109145272	Bacteria	n	n		uncultured bacterium
JQ998334	410	18	378	0	100%	100%	109145452	Bacteria	n	n		uncultured bacterium
JQ998472	463	5	430	0	100%	100%	109145521	Bacteria	n	n		uncultured bacterium
JQ998104	336	18	268	1E-127	100%	100%	109145993	Bacteria	n	n		uncultured bacterium
JQ998327	407	5	301	4E-153	100%	100%	109146142	Bacteria	n	n		uncultured bacterium
JQ998861	542	5	513	0	100%	100%	109146225	Bacteria	n	n		uncultured bacterium
JQ998894	544	263	542	2E-138	99%	99%	109146231	Bacteria	n	n		uncultured bacterium

JQ998476	464	6	401	0	99%	99%	109146267	Bacteria	n	n	uncultured bacterium
JQ998222	375	4	340	2E-175	100%	100%	109146295	Bacteria	n	n	uncultured bacterium
JQ998802	537	18	491	0	100%	100%	109146793	Bacteria	n	n	uncultured bacterium
JQ999044	555	43	553	0	100%	100%	109146801	Bacteria	n	n	uncultured bacterium
JQ998107	337	81	298	3E-109	100%	100%	109147422	Bacteria	n	n	uncultured bacterium
JQ998961	548	11	548	0	99%	99%	109147514	Bacteria	n	n	uncultured bacterium
JQ998522	480	40	434	0	100%	100%	109147647	Bacteria	n	n	uncultured bacterium
JQ998803	537	5	517	9E-171	89%	89%	109147713	Bacteria	n	n	uncultured bacterium
JQ997906	253	18	223	4E-101	100%	100%	109147753	Bacteria	n	n	uncultured bacterium
JQ998079	325	33	292	3E-129	99%	99%	109147859	Bacteria	n	n	uncultured bacterium
JQ998502	475	5	121	5E-34	91%	91%	110188400	Bacteria	n	n	uncultured bacterium
JQ998137	349	5	312	3E-144	97%	97%	110434048	Bacteria	n	n	uncultured bacterium
JQ999085	558	1	553	0	98%	98%	110434224	Bacteria	n	n	uncultured bacterium
JQ998876	543	5	458	0	96%	96%	110434340	Bacteria	n	n	uncultured bacterium
JQ997923	263	5	228	2E-94	95%	95%	110434402	Bacteria	n	n	uncultured bacterium
JQ998928	546	23	538	0	95%	95%	110434494	Bacteria	n	n	uncultured bacterium
JQ998425	446	18	388	1E-169	96%	96%	110434584	Bacteria	n	n	uncultured bacterium
JQ998944	547	18	542	0	97%	97%	110434604	Bacteria	n	n	uncultured bacterium
JQ998405	438	5	364	1E-129	90%	90%	110434708	Bacteria	n	n	uncultured bacterium
JQ998184	363	5	297	1E-112	92%	92%	110435398	Bacteria	n	n	uncultured bacterium
JQ998152	352	5	300	1E-152	100%	100%	110435586	Bacteria	n	n	uncultured bacterium
JQ998070	323	5	237	6E-111	98%	98%	110435722	Bacteria	n	n	uncultured bacterium
JQ998204	369	18	296	9E-120	95%	95%	110436204	Bacteria	n	n	uncultured bacterium
JQ998720	529	19	463	0	96%	96%	110436291	Bacteria	n	n	uncultured bacterium
JQ998217	373	5	330	7E-126	92%	92%	110437056	Bacteria	n	n	uncultured bacterium
JQ999199	617	17	503	0	92%	92%	110438165	Bacteria	n	n	uncultured bacterium
JQ997924	263	23	215	2E-90	98%	98%	110438192	Bacteria	n	n	uncultured bacterium
JQ998763	534	7	527	0	89%	89%	110438225	Bacteria	n	n	uncultured bacterium
JQ998037	311	15	269	3E-83	89%	89%	110438326	Bacteria	n	n	uncultured bacterium
JQ998038	311	12	275	7E-115	95%	95%	110438379	Bacteria	n	n	uncultured bacterium
JQ998205	369	23	317	2E-121	94%	94%	110438381	Bacteria	n	n	uncultured bacterium
JQ998551	490	14	295	1E-124	96%	96%	110438438	Bacteria	n	n	uncultured bacterium
JQ998316	405	5	360	0	100%	100%	110438638	Bacteria	n	n	uncultured bacterium
JQ998467	461	72	413	1E-149	95%	95%	110438856	Bacteria	n	n	uncultured bacterium
JQ997947	272	18	228	2E-95	97%	97%	110439795	Bacteria	n	n	uncultured bacterium
JQ998688	525	5	478	0	94%	94%	110439901	Bacteria	n	n	uncultured bacterium
JQ998295	398	5	366	9E-145	93%	93%	110440019	Bacteria	n	n	uncultured bacterium
JQ999112	561	18	550	0	94%	94%	110440098	Bacteria	n	n	uncultured bacterium
JQ998275	392	18	347	2E-166	99%	99%	110440160	Bacteria	n	n	uncultured bacterium
JQ998473	463	16	413	1E-163	93%	93%	110440247	Bacteria	n	n	uncultured bacterium
JQ998601	505	18	468	0	96%	96%	110440335	Bacteria	n	n	uncultured bacterium
JQ998673	523	18	523	0	95%	95%	110440402	Bacteria	n	n	uncultured bacterium
JQ998359	419	18	364	7E-161	97%	97%	110440797	Bacteria	n	n	uncultured bacterium
JQ998661	520	18	470	4E-169	91%	91%	110440804	Bacteria	n	n	uncultured bacterium
JQ997976	287	5	223	7E-110	100%	100%	110441643	Bacteria	n	n	uncultured bacterium
JQ998143	350	5	308	1E-123	93%	93%	110442289	Bacteria	n	n	uncultured bacterium
JQ998558	493	18	446	0	97%	97%	110443809	Bacteria	n	n	uncultured bacterium
JQ999173	576	18	569	0	96%	96%	110444022	Bacteria	n	n	uncultured bacterium
JQ998468	461	24	403	3E-170	96%	96%	110444135	Bacteria	n	n	uncultured bacterium
JQ998945	547	22	544	0	99%	99%	110444775	Bacteria	n	n	uncultured bacterium
JQ998576	497	21	440	0	96%	96%	110444791	Bacteria	n	n	uncultured bacterium
JQ998849	541	1	527	0	95%	95%	110444825	Bacteria	n	n	uncultured bacterium
JQ998509	477	4	416	0	100%	100%	110445101	Bacteria	n	n	uncultured bacterium
JQ997888	244	18	200	1E-71	94%	94%	110445140	Bacteria	n	n	uncultured bacterium
JQ998298	399	18	357	1E-148	95%	95%	110445294	Bacteria	n	n	uncultured bacterium
JQ998946	547	5	543	0	91%	91%	110445618	Bacteria	n	n	uncultured bacterium
JQ997939	269	14	232	2E-90	95%	95%	110447114	Bacteria	n	n	uncultured bacterium
JQ998516	479	16	447	0	94%	94%	110447803	Bacteria	n	n	uncultured bacterium
JQ998564	494	18	437	0	94%	94%	110448781	Bacteria	n	n	uncultured bacterium
JQ997940	269	5	209	4E-82	94%	94%	110449223	Bacteria	n	n	uncultured bacterium

JQ998830	539	19	535	0	94%	94%	110449534	Bacteria	n	n	uncultured bacterium
JQ998993	550	18	529	0	93%	93%	110449796	Bacteria	n	n	uncultured bacterium
JQ998469	461	24	390	6E-157	94%	94%	110450561	Bacteria	n	n	uncultured bacterium
JQ998843	540	7	537	0	90%	90%	110450644	Bacteria	n	n	uncultured bacterium
JQ998974	549	5	538	0	96%	96%	110450647	Bacteria	n	n	uncultured bacterium
JQ998460	458	4	424	3E-170	93%	93%	110450683	Bacteria	n	n	uncultured bacterium
JQ998652	518	26	476	0	98%	98%	110450754	Bacteria	n	n	uncultured bacterium
JQ998130	347	4	301	6E-126	94%	94%	110451179	Bacteria	n	n	uncultured bacterium
JQ998831	539	17	507	0	92%	92%	110832477	Bacteria	n	n	uncultured bacterium
JQ998779	535	17	490	0	99%	99%	110832479	Bacteria	n	n	uncultured bacterium
JQ999137	565	20	562	0	96%	96%	110832499	Bacteria	n	n	uncultured bacterium
JQ997886	242	5	219	9E-103	99%	99%	110832569	Bacteria	n	n	uncultured bacterium
JQ998462	459	17	325	2E-147	98%	98%	112819327	Bacteria	n	n	uncultured bacterium
JQ997909	255	1	194	5E-96	100%	100%	113870203	Bacteria	n	n	uncultured bacterium
JQ998877	543	14	540	0	100%	100%	113870743	Bacteria	n	n	uncultured bacterium
JQ998947	547	5	541	0	95%	95%	116876085	Bacteria	n	n	uncultured bacterium
JQ998080	325	1	274	3E-119	96%	96%	116876101	Bacteria	n	n	uncultured bacterium
JQ997938	268	1	230	5E-86	92%	92%	117969615	Bacteria	n	n	uncultured bacterium
JQ998862	542	18	538	0	95%	95%	118406565	Bacteria	n	n	uncultured bacterium
JQ998709	528	74	525	7E-167	91%	91%	119436067	Bacteria	n	n	uncultured bacterium
JQ999045	555	16	549	0	91%	91%	119436142	Bacteria	n	n	uncultured bacterium
JQ998356	417	24	375	1E-128	91%	91%	119436366	Bacteria	n	n	uncultured bacterium
JQ998863	542	5	538	0	92%	92%	119437478	Bacteria	n	n	uncultured bacterium
JQ999046	555	5	551	0	93%	93%	119437735	Bacteria	n	n	uncultured bacterium
JQ998523	480	4	426	0	98%	98%	124303494	Bacteria	n	n	uncultured bacterium
JQ999146	567	5	565	0	100%	100%	124303511	Bacteria	n	n	uncultured bacterium
JQ998406	438	5	378	2E-167	95%	95%	125660699	Bacteria	n	n	uncultured bacterium
JQ999057	555	18	520	0	98%	98%	125715968	Bacteria	n	n	uncultured bacterium
JQ997980	289	18	243	1E-92	94%	94%	125742383	Bacteria	n	n	uncultured bacterium
JQ999061	556	3	552	0	99%	99%	126111464	Bacteria	n	n	uncultured bacterium
JQ998131	347	25	295	2E-115	95%	95%	126113031	Bacteria	n	n	uncultured bacterium
JQ998895	544	3	541	0	100%	100%	126113813	Bacteria	n	n	uncultured bacterium
JQ998483	467	18	438	0	97%	97%	126113939	Bacteria	n	n	uncultured bacterium
JQ999138	565	18	565	0	97%	97%	126114009	Bacteria	n	n	uncultured bacterium
JQ998218	373	93	327	2E-105	97%	97%	126114126	Bacteria	n	n	uncultured bacterium
JQ998764	534	17	532	0	98%	98%	126115468	Bacteria	n	n	uncultured bacterium
JQ998994	550	4	540	0	99%	99%	126115792	Bacteria	n	n	uncultured bacterium
JQ998962	548	1	528	0	92%	92%	126362330	Bacteria	n	n	uncultured bacterium
JQ998765	534	5	529	0	96%	96%	126674407	Bacteria	n	n	uncultured bacterium
JQ998463	459	112	405	8E-146	99%	99%	134021526	Bacteria	n	n	uncultured bacterium
JQ999113	561	18	548	0	97%	97%	134140995	Bacteria	n	n	uncultured bacterium
JQ998025	307	73	262	4E-92	99%	99%	138239976	Bacteria	n	n	uncultured bacterium
JQ998056	318	10	162	8E-70	99%	99%	138240043	Bacteria	n	n	uncultured bacterium
JQ997896	248	5	211	2E-99	99%	99%	138240346	Bacteria	n	n	uncultured bacterium
JQ998701	527	18	513	0	96%	96%	138240383	Bacteria	n	n	uncultured bacterium
JQ998172	359	4	324	2E-166	100%	100%	140326239	Bacteria	n	n	uncultured bacterium
JQ998185	363	5	330	1E-153	97%	97%	145284746	Bacteria	n	n	uncultured bacterium
JQ998039	311	17	259	6E-116	98%	98%	145285090	Bacteria	n	n	uncultured bacterium
JQ999071	557	34	555	0	98%	98%	145286222	Bacteria	n	n	uncultured bacterium
JQ998873	542	5	538	0	100%	100%	145582288	Bacteria	n	n	uncultured bacterium
JQ999105	559	17	532	0	97%	97%	145582336	Bacteria	n	n	uncultured bacterium
JQ998579	497	24	447	3E-145	89%	89%	145583005	Bacteria	n	n	uncultured bacterium
JQ998959	547	17	466	0	99%	99%	145583249	Bacteria	n	n	uncultured bacterium
JQ998195	365	23	314	4E-83	88%	88%	145584302	Bacteria	n	n	uncultured bacterium
JQ998541	485	3	418	6E-177	94%	94%	145584307	Bacteria	n	n	uncultured bacterium
JQ997901	250	5	205	3E-83	95%	95%	146165027	Bacteria	n	n	uncultured bacterium
JQ999072	557	5	551	0	99%	99%	146575767	Bacteria	n	n	uncultured bacterium
JQ999163	573	18	566	0	93%	93%	148248671	Bacteria	n	n	uncultured bacterium
JQ998347	414	20	368	7E-166	97%	97%	148472131	Bacteria	n	n	uncultured bacterium
JQ998100	334	5	302	1E-123	94%	94%	148730991	Bacteria	n	n	uncultured bacterium

JQ998243	381	62	335	4E-133	99%	99%	148731710	Bacteria	n	n	uncultured bacterium
JQ999073	557	5	554	0	99%	99%	148828955	Bacteria	n	n	uncultured bacterium
JQ998695	526	18	523	0	95%	95%	149350589	Bacteria	n	n	uncultured bacterium
JQ999179	579	17	559	0	92%	92%	152926424	Bacteria	n	n	uncultured bacterium
JQ998736	531	20	450	0	94%	94%	152926426	Bacteria	n	n	uncultured bacterium
JQ998721	529	18	523	0	99%	99%	155968453	Bacteria	n	n	uncultured bacterium
JQ998817	538	5	538	0	97%	97%	156505941	Bacteria	n	n	uncultured bacterium
JQ998790	535	16	527	0	98%	98%	156522978	Bacteria	n	n	uncultured bacterium
JQ997910	255	4	188	6E-80	96%	96%	157498485	Bacteria	n	n	uncultured bacterium
JQ999023	553	6	533	0	89%	89%	157499269	Bacteria	n	n	uncultured bacterium
JQ998053	317	5	263	4E-132	100%	100%	157500126	Bacteria	n	n	uncultured bacterium
JQ998088	328	5	251	2E-110	96%	96%	157649100	Bacteria	n	n	uncultured bacterium
JQ999030	553	5	514	0	92%	92%	157690469	Bacteria	n	n	uncultured bacterium
JQ998538	484	5	436	0	96%	96%	157690565	Bacteria	n	n	uncultured bacterium
JQ998975	549	18	531	0	98%	98%	157740581	Bacteria	n	n	uncultured bacterium
JQ998573	496	5	442	0	95%	95%	157926675	Bacteria	n	n	uncultured bacterium
JQ997876	228	5	165	2E-60	94%	94%	157926821	Bacteria	n	n	uncultured bacterium
JQ998127	346	19	318	8E-155	100%	100%	157927262	Bacteria	n	n	uncultured bacterium
JQ998174	360	16	85	4E-23	97%	97%	157927557	Bacteria	n	n	uncultured bacterium
JQ997933	267	164	222	5E-21	100%	100%	157927616	Bacteria	n	n	uncultured bacterium
JQ998427	446	33	400	5E-103	86%	86%	158148402	Bacteria	n	n	uncultured bacterium
JQ998367	422	14	391	2E-176	97%	97%	158148421	Bacteria	n	n	uncultured bacterium
JQ998430	448	15	359	5E-163	97%	97%	158442466	Bacteria	n	n	uncultured bacterium
JQ998201	367	24	324	2E-135	96%	96%	159885105	Bacteria	n	n	uncultured bacterium
JQ998464	459	17	377	0	99%	99%	160280259	Bacteria	n	n	uncultured bacterium
JQ998687	524	5	472	0	94%	94%	160332417	Bacteria	n	n	uncultured bacterium
JQ997892	246	5	200	3E-97	100%	100%	160714403	Bacteria	n	n	uncultured bacterium
JQ998737	531	5	387	1E-154	93%	93%	160922872	Bacteria	n	n	uncultured bacterium
JQ998271	390	5	354	4E-64	82%	82%	160922944	Bacteria	n	n	uncultured bacterium
JQ998609	507	5	444	0	98%	98%	160922945	Bacteria	n	n	uncultured bacterium
JQ999160	572	17	545	0	98%	98%	161876636	Bacteria	n	n	uncultured bacterium
JQ998386	431	19	386	3E-150	93%	93%	162950760	Bacteria	n	n	uncultured bacterium
JQ998272	390	17	305	9E-145	99%	99%	164460317	Bacteria	n	n	uncultured bacterium
JQ998110	338	5	306	3E-139	97%	97%	164509981	Bacteria	n	n	uncultured bacterium
JQ998635	515	16	73	3E-16	97%	97%	164521760	Bacteria	n	n	uncultured bacterium
JQ998584	501	136	435	3E-150	99%	99%	164523483	Bacteria	n	n	uncultured bacterium
JQ998896	544	52	527	0	96%	96%	164609939	Bacteria	n	n	uncultured bacterium
JQ998165	357	5	315	6E-151	98%	98%	164610216	Bacteria	n	n	uncultured bacterium
JQ998878	543	11	533	0	94%	94%	165968461	Bacteria	n	n	uncultured bacterium
JQ998963	548	4	544	0	99%	99%	166202170	Bacteria	n	n	uncultured bacterium
JQ998596	504	18	442	0	100%	100%	168997732	Bacteria	n	n	uncultured bacterium
JQ998491	469	5	424	0	95%	95%	168997829	Bacteria	n	n	uncultured bacterium
JQ998092	329	5	297	6E-146	99%	99%	169127868	Bacteria	n	n	uncultured bacterium
JQ999097	559	5	533	0	93%	93%	169129792	Bacteria	n	n	uncultured bacterium
JQ998181	362	5	326	4E-148	97%	97%	169130297	Bacteria	n	n	uncultured bacterium
JQ998546	487	4	441	1E-154	90%	90%	169130400	Bacteria	n	n	uncultured bacterium
JQ998392	433	8	381	7E-156	94%	94%	169131607	Bacteria	n	n	uncultured bacterium
JQ999086	558	18	555	0	100%	100%	169132460	Bacteria	n	n	uncultured bacterium
JQ998610	507	18	470	0	97%	97%	169132729	Bacteria	n	n	uncultured bacterium
JQ998702	527	15	516	0	93%	93%	169132832	Bacteria	n	n	uncultured bacterium
JQ998034	309	3	266	3E-119	97%	97%	169132904	Bacteria	n	n	uncultured bacterium
JQ998223	375	5	311	5E-152	99%	99%	169133475	Bacteria	n	n	uncultured bacterium
JQ998436	450	200	413	6E-107	100%	100%	169134448	Bacteria	n	n	uncultured bacterium
JQ998428	447	5	384	5E-173	96%	96%	169144791	Bacteria	n	n	uncultured bacterium
JQ997949	273	46	221	2E-79	98%	98%	169265423	Bacteria	n	n	uncultured bacterium
JQ998662	520	4	478	0	91%	91%	169266227	Bacteria	n	n	uncultured bacterium
JQ998879	543	5	519	0	97%	97%	169267043	Bacteria	n	n	uncultured bacterium
JQ998995	550	61	547	2E-177	90%	90%	169268927	Bacteria	n	n	uncultured bacterium
JQ997919	261	18	122	1E-16	84%	84%	169269241	Bacteria	n	n	uncultured bacterium
JQ998071	323	24	271	4E-117	98%	98%	169270538	Bacteria	n	n	uncultured bacterium

JQ999003	551	20	542	0	94%	94%	169273928	Bacteria	n	n	uncultured bacterium
JQ998703	527	2	490	5E-178	90%	90%	169273933	Bacteria	n	n	uncultured bacterium
JQ998383	429	21	386	0	98%	98%	169273949	Bacteria	n	n	uncultured bacterium
JQ999114	561	27	561	0	98%	98%	169273981	Bacteria	n	n	uncultured bacterium
JQ998559	493	5	434	0	94%	94%	169274045	Bacteria	n	n	uncultured bacterium
JQ998897	544	18	540	0	97%	97%	169274573	Bacteria	n	n	uncultured bacterium
JQ999134	564	5	559	0	99%	99%	169274878	Bacteria	n	n	uncultured bacterium
JQ998722	529	16	519	5E-178	90%	90%	169275081	Bacteria	n	n	uncultured bacterium
JQ998780	535	15	432	0	96%	96%	169275203	Bacteria	n	n	uncultured bacterium
JQ998319	406	1	343	5E-167	98%	98%	169275222	Bacteria	n	n	uncultured bacterium
JQ998471	462	16	425	0	98%	98%	169275300	Bacteria	n	n	uncultured bacterium
JQ999196	612	18	602	0	90%	90%	169275355	Bacteria	n	n	uncultured bacterium
JQ998898	544	4	535	0	95%	95%	169275415	Bacteria	n	n	uncultured bacterium
JQ998128	346	5	296	1E-132	97%	97%	169275467	Bacteria	n	n	uncultured bacterium
JQ999125	563	26	510	0	94%	94%	169275696	Bacteria	n	n	uncultured bacterium
JQ998118	341	24	310	1E-78	87%	87%	169275865	Bacteria	n	n	uncultured bacterium
JQ998880	543	5	540	0	94%	94%	169276279	Bacteria	n	n	uncultured bacterium
JQ998738	531	5	501	0	95%	95%	169276317	Bacteria	n	n	uncultured bacterium
JQ999909	532	243	528	2E-138	98%	98%	169277275	Bacteria	n	n	uncultured bacterium
JQ998095	331	16	299	2E-121	95%	95%	169278085	Bacteria	n	n	uncultured bacterium
JQ998470	461	18	402	1E-159	94%	94%	169278764	Bacteria	n	n	uncultured bacterium
JQ999216	730	5	237	4E-51	84%	84%	169278944	Bacteria	n	n	uncultured bacterium
JQ998266	388	87	352	7E-116	96%	96%	169280166	Bacteria	n	n	uncultured bacterium
JQ999062	556	12	551	0	90%	90%	169280905	Bacteria	n	n	uncultured bacterium
JQ997911	255	7	223	4E-97	97%	97%	169280992	Bacteria	n	n	uncultured bacterium
JQ998490	468	18	419	0	99%	99%	169281198	Bacteria	n	n	uncultured bacterium
JQ998257	385	17	348	1E-74	84%	84%	169281437	Bacteria	n	n	uncultured bacterium
JQ998006	300	5	247	1E-121	100%	100%	169281727	Bacteria	n	n	uncultured bacterium
JQ998517	479	17	376	4E-164	96%	96%	169281966	Bacteria	n	n	uncultured bacterium
JQ998340	412	17	298	2E-131	97%	97%	169282044	Bacteria	n	n	uncultured bacterium
JQ999126	563	6	534	0	91%	91%	169282669	Bacteria	n	n	uncultured bacterium
JQ998948	547	17	544	0	96%	96%	169283420	Bacteria	n	n	uncultured bacterium
JQ998351	416	16	348	2E-166	99%	99%	169283464	Bacteria	n	n	uncultured bacterium
JQ998527	482	5	445	5E-143	88%	88%	169283814	Bacteria	n	n	uncultured bacterium
JQ999087	558	16	529	0	95%	95%	169283893	Bacteria	n	n	uncultured bacterium
JQ998227	377	5	336	3E-139	94%	94%	169289574	Bacteria	n	n	uncultured bacterium
JQ998273	390	5	337	9E-130	92%	92%	169290095	Bacteria	n	n	uncultured bacterium
JQ998914	545	15	543	0	90%	90%	169290564	Bacteria	n	n	uncultured bacterium
JQ998102	335	5	282	6E-121	95%	95%	169290664	Bacteria	n	n	uncultured bacterium
JQ998539	485	18	434	0	95%	95%	169290715	Bacteria	n	n	uncultured bacterium
JQ998320	406	15	376	0	99%	99%	169291056	Bacteria	n	n	uncultured bacterium
JQ998437	450	18	426	0	97%	97%	169291284	Bacteria	n	n	uncultured bacterium
JQ998138	349	18	283	4E-113	95%	95%	169797979	Bacteria	n	n	uncultured bacterium
JQ999032	554	17	553	0	99%	99%	170652676	Bacteria	n	n	uncultured bacterium
JQ998804	537	4	528	0	97%	97%	170652677	Bacteria	n	n	uncultured bacterium
JQ998084	327	18	296	3E-113	94%	94%	171262582	Bacteria	n	n	uncultured bacterium
JQ997903	252	30	215	3E-83	97%	97%	171262599	Bacteria	n	n	uncultured bacterium
JQ998704	527	5	470	0	94%	94%	171467457	Bacteria	n	n	uncultured bacterium
JQ998147	351	6	212	9E-70	91%	91%	187424332	Bacteria	n	n	uncultured bacterium
JQ998625	511	4	395	0	98%	98%	187438950	Bacteria	n	n	uncultured bacterium
JQ998317	405	18	318	1E-153	100%	100%	187736691	Bacteria	n	n	uncultured bacterium
JQ998480	466	18	405	0	99%	99%	187963663	Bacteria	n	n	uncultured bacterium
JQ999140	566	20	546	0	93%	93%	187964336	Bacteria	n	n	uncultured bacterium
JQ998766	534	5	475	0	96%	96%	187965718	Bacteria	n	n	uncultured bacterium
JQ998554	491	14	363	3E-180	100%	100%	187967718	Bacteria	n	n	uncultured bacterium
JQ998035	310	18	276	7E-120	97%	97%	187968607	Bacteria	n	n	uncultured bacterium
JQ997934	267	18	198	8E-79	96%	96%	187968619	Bacteria	n	n	uncultured bacterium
JQ998234	379	5	322	8E-165	100%	100%	188530112	Bacteria	n	n	uncultured bacterium
JQ998276	392	24	347	7E-131	93%	93%	189309776	Bacteria	n	n	uncultured bacterium
JQ997993	296	5	263	9E-119	97%	97%	190364260	Bacteria	n	n	uncultured bacterium

JQ998007	300	15	253	3E-94	93%	93%	190705415	Bacteria	n	n	uncultured bacterium
JQ998134	348	5	316	2E-131	94%	94%	190707473	Bacteria	n	n	uncultured bacterium
JQ998616	508	5	432	4E-159	91%	91%	192966107	Bacteria	n	n	uncultured bacterium
JQ999088	558	16	552	3E-161	87%	87%	192966414	Bacteria	n	n	uncultured bacterium
JQ998950	547	5	542	0	98%	98%	192967750	Bacteria	n	n	uncultured bacterium
JQ998899	544	21	529	0	97%	97%	192968449	Bacteria	n	n	uncultured bacterium
JQ998767	534	23	503	0	100%	100%	192970868	Bacteria	n	n	uncultured bacterium
JQ998418	444	15	377	1E-164	96%	96%	192972691	Bacteria	n	n	uncultured bacterium
JQ998074	324	6	231	3E-84	92%	92%	192974165	Bacteria	n	n	uncultured bacterium
JQ998577	497	22	227	9E-101	100%	100%	192975291	Bacteria	n	n	uncultured bacterium
JQ998412	440	5	329	1E-138	95%	95%	192975946	Bacteria	n	n	uncultured bacterium
JQ999089	558	22	487	0	92%	92%	192975965	Bacteria	n	n	uncultured bacterium
JQ998915	545	18	541	0	91%	91%	192976013	Bacteria	n	n	uncultured bacterium
JQ998313	404	23	108	5E-28	95%	95%	192976059	Bacteria	n	n	uncultured bacterium
JQ998832	539	54	497	7E-172	92%	92%	192976124	Bacteria	n	n	uncultured bacterium
JQ998368	423	102	315	3E-100	98%	98%	192976149	Bacteria	n	n	uncultured bacterium
JQ998754	533	5	485	0	93%	93%	192976156	Bacteria	n	n	uncultured bacterium
JQ998162	356	4	324	3E-119	91%	91%	192976166	Bacteria	n	n	uncultured bacterium
JQ998108	337	18	302	3E-143	99%	99%	192976207	Bacteria	n	n	uncultured bacterium
JQ998377	427	5	357	3E-154	95%	95%	192976228	Bacteria	n	n	uncultured bacterium
JQ998647	517	8	445	3E-151	90%	90%	192976293	Bacteria	n	n	uncultured bacterium
JQ997981	289	18	246	5E-106	97%	97%	192976431	Bacteria	n	n	uncultured bacterium
JQ998755	533	24	530	0	95%	95%	192976582	Bacteria	n	n	uncultured bacterium
JQ998929	546	5	519	0	94%	94%	192976655	Bacteria	n	n	uncultured bacterium
JQ997982	290	9	239	3E-108	98%	98%	192976674	Bacteria	n	n	uncultured bacterium
JQ998739	531	15	526	0	99%	99%	192976754	Bacteria	n	n	uncultured bacterium
JQ999024	553	18	437	0	96%	96%	192976789	Bacteria	n	n	uncultured bacterium
JQ998674	523	15	523	0	97%	97%	192976806	Bacteria	n	n	uncultured bacterium
JQ998976	549	15	546	0	100%	100%	192976819	Bacteria	n	n	uncultured bacterium
JQ998534	484	18	444	0	94%	94%	192977859	Bacteria	n	n	uncultured bacterium
JQ998360	419	18	376	0	99%	99%	192978967	Bacteria	n	n	uncultured bacterium
JQ998314	404	1	347	9E-160	96%	96%	192978977	Bacteria	n	n	uncultured bacterium
JQ999033	554	18	548	0	96%	96%	192979708	Bacteria	n	n	uncultured bacterium
JQ998114	340	23	294	4E-128	98%	98%	192979745	Bacteria	n	n	uncultured bacterium
JQ998619	509	18	487	0	92%	92%	192979771	Bacteria	n	n	uncultured bacterium
JQ998964	548	5	544	0	91%	91%	192979804	Bacteria	n	n	uncultured bacterium
JQ999176	578	5	534	0	97%	97%	192979942	Bacteria	n	n	uncultured bacterium
JQ998792	536	18	470	0	96%	96%	192979948	Bacteria	n	n	uncultured bacterium
JQ998371	424	5	375	3E-169	96%	96%	192979964	Bacteria	n	n	uncultured bacterium
JQ999063	556	4	535	0	95%	95%	192979974	Bacteria	n	n	uncultured bacterium
JQ998793	536	18	532	2E-157	87%	87%	192979984	Bacteria	n	n	uncultured bacterium
JQ998175	360	19	309	1E-147	100%	100%	192980004	Bacteria	n	n	uncultured bacterium
JQ998139	349	23	293	2E-115	95%	95%	192980549	Bacteria	n	n	uncultured bacterium
JQ997881	240	28	196	6E-60	92%	92%	192980604	Bacteria	n	n	uncultured bacterium
JQ998727	530	5	492	0	97%	97%	192980815	Bacteria	n	n	uncultured bacterium
JQ998781	535	1	524	0	94%	94%	192980843	Bacteria	n	n	uncultured bacterium
JQ998407	438	9	383	2E-176	97%	97%	192980848	Bacteria	n	n	uncultured bacterium
JQ998157	353	5	322	1E-143	96%	96%	192980858	Bacteria	n	n	uncultured bacterium
JQ998085	327	17	296	1E-113	94%	94%	192980871	Bacteria	n	n	uncultured bacterium
JQ998115	340	5	256	4E-108	95%	95%	192980926	Bacteria	n	n	uncultured bacterium
JQ997944	271	23	239	4E-77	91%	91%	192981069	Bacteria	n	n	uncultured bacterium
JQ998597	504	18	446	9E-131	87%	87%	192981149	Bacteria	n	n	uncultured bacterium
JQ998666	521	15	515	0	94%	94%	192981198	Bacteria	n	n	uncultured bacterium
JQ998178	361	3	243	3E-114	98%	98%	192981346	Bacteria	n	n	uncultured bacterium
JQ998481	466	5	385	3E-160	94%	94%	192983964	Bacteria	n	n	uncultured bacterium
JQ998794	536	17	496	0	99%	99%	192983994	Bacteria	n	n	uncultured bacterium
JQ998747	532	4	473	0	94%	94%	192984056	Bacteria	n	n	uncultured bacterium
JQ997893	246	18	201	3E-77	96%	96%	192984107	Bacteria	n	n	uncultured bacterium
JQ998818	538	18	480	0	97%	97%	192984128	Bacteria	n	n	uncultured bacterium
JQ998549	488	5	427	0	96%	96%	192984156	Bacteria	n	n	uncultured bacterium

JQ999047	555	24	545	0	98%	98%	192984161	Bacteria	n	n	uncultured bacterium
JQ998833	539	17	536	0	99%	99%	192984206	Bacteria	n	n	uncultured bacterium
JQ998626	511	17	472	3E-175	91%	91%	192984284	Bacteria	n	n	uncultured bacterium
JQ998179	361	1	296	3E-139	97%	97%	192984388	Bacteria	n	n	uncultured bacterium
JQ998086	327	5	280	2E-136	99%	99%	192984568	Bacteria	n	n	uncultured bacterium
JQ999157	571	4	473	0	95%	95%	192984599	Bacteria	n	n	uncultured bacterium
JQ998008	300	22	268	9E-109	96%	96%	192984616	Bacteria	n	n	uncultured bacterium
JQ998352	416	17	367	0	100%	100%	192984640	Bacteria	n	n	uncultured bacterium
JQ998518	479	18	412	0	99%	99%	192984646	Bacteria	n	n	uncultured bacterium
JQ998413	440	5	406	4E-168	94%	94%	192984658	Bacteria	n	n	uncultured bacterium
JQ998040	311	5	255	2E-115	97%	97%	192984672	Bacteria	n	n	uncultured bacterium
JQ998036	310	5	265	3E-133	100%	100%	192984677	Bacteria	n	n	uncultured bacterium
JQ998611	507	42	466	1E-168	92%	92%	192984692	Bacteria	n	n	uncultured bacterium
JQ998452	456	7	422	4E-164	92%	92%	192984702	Bacteria	n	n	uncultured bacterium
JQ998267	388	5	344	2E-165	98%	98%	192984705	Bacteria	n	n	uncultured bacterium
JQ998321	406	24	374	3E-130	91%	91%	192984743	Bacteria	n	n	uncultured bacterium
JQ998410	439	5	396	3E-174	95%	95%	192984840	Bacteria	n	n	uncultured bacterium
JQ998434	449	18	403	8E-146	91%	91%	192985210	Bacteria	n	n	uncultured bacterium
JQ998417	443	4	390	3E-90	84%	84%	192985646	Bacteria	n	n	uncultured bacterium
JQ999004	551	4	549	0	97%	97%	192988880	Bacteria	n	n	uncultured bacterium
JQ999064	556	3	511	0	92%	92%	192989027	Bacteria	n	n	uncultured bacterium
JQ997897	248	19	204	6E-85	98%	98%	192989092	Bacteria	n	n	uncultured bacterium
JQ998667	522	24	474	0	92%	92%	192989236	Bacteria	n	n	uncultured bacterium
JQ998850	541	4	504	0	93%	93%	192989243	Bacteria	n	n	uncultured bacterium
JQ998805	537	5	537	0	91%	91%	192989244	Bacteria	n	n	uncultured bacterium
JQ998728	530	5	490	0	92%	92%	192989281	Bacteria	n	n	uncultured bacterium
JQ998659	519	5	472	0	98%	98%	192989317	Bacteria	n	n	uncultured bacterium
JQ998484	467	18	351	1E-173	100%	100%	192989331	Bacteria	n	n	uncultured bacterium
JQ998063	320	8	288	1E-122	95%	95%	192989685	Bacteria	n	n	uncultured bacterium
JQ998500	474	5	416	0	97%	97%	192989734	Bacteria	n	n	uncultured bacterium
JQ998041	312	5	226	3E-108	99%	99%	193849299	Bacteria	n	n	uncultured bacterium
JQ998213	371	24	327	2E-115	92%	92%	194136692	Bacteria	n	n	uncultured bacterium
JQ998017	304	18	272	3E-123	98%	98%	194137835	Bacteria	n	n	uncultured bacterium
JQ998641	516	5	484	0	100%	100%	194138815	Bacteria	n	n	uncultured bacterium
JQ997958	277	5	237	1E-97	95%	95%	194139439	Bacteria	n	n	uncultured bacterium
JQ998881	543	18	540	0	91%	91%	194139558	Bacteria	n	n	uncultured bacterium
JQ998585	501	17	456	0	99%	99%	194139664	Bacteria	n	n	uncultured bacterium
JQ998627	511	13	458	0	99%	99%	194139828	Bacteria	n	n	uncultured bacterium
JQ998681	524	5	521	0	99%	99%	194139970	Bacteria	n	n	uncultured bacterium
JQ999025	553	18	550	0	99%	99%	194140001	Bacteria	n	n	uncultured bacterium
JQ998441	451	18	404	3E-175	96%	96%	194140055	Bacteria	n	n	uncultured bacterium
JQ998166	358	5	313	2E-141	96%	96%	194140159	Bacteria	n	n	uncultured bacterium
JQ998353	416	18	370	3E-179	99%	99%	194140237	Bacteria	n	n	uncultured bacterium
JQ997975	286	14	241	7E-115	100%	100%	194140255	Bacteria	n	n	uncultured bacterium
JQ997953	275	17	231	1E-107	100%	100%	194140257	Bacteria	n	n	uncultured bacterium
JQ998336	411	18	364	0	100%	100%	194293700	Bacteria	n	n	uncultured bacterium
JQ998132	347	5	296	1E-143	99%	99%	194592166	Bacteria	n	n	uncultured bacterium
JQ998018	304	5	259	7E-130	100%	100%	194597963	Bacteria	n	n	uncultured bacterium
JQ998209	370	18	319	3E-154	100%	100%	194718448	Bacteria	n	n	uncultured bacterium
JQ998328	407	5	386	1E-114	88%	88%	194719015	Bacteria	n	n	uncultured bacterium
JQ997986	293	3	258	3E-73	87%	87%	195542932	Bacteria	n	n	uncultured bacterium
JQ999192	604	18	283	7E-103	93%	93%	197108620	Bacteria	n	n	uncultured bacterium
JQ997956	276	16	208	2E-95	100%	100%	197258193	Bacteria	n	n	uncultured bacterium
JQ999014	552	18	512	0	92%	92%	197342931	Bacteria	n	n	uncultured bacterium
JQ999115	561	17	368	3E-180	99%	99%	197342941	Bacteria	n	n	uncultured bacterium
JQ998064	320	17	273	1E-127	99%	99%	197342951	Bacteria	n	n	uncultured bacterium
JQ997950	273	100	241	4E-57	96%	96%	197342960	Bacteria	n	n	uncultured bacterium
JQ998348	415	16	354	1E-163	98%	98%	197342998	Bacteria	n	n	uncultured bacterium
JQ998335	410	5	356	3E-135	92%	92%	197343098	Bacteria	n	n	uncultured bacterium
JQ999005	551	15	484	0	95%	95%	197345311	Bacteria	n	n	uncultured bacterium

JQ998448	454	18	409	2E-167	94%	94%	197346844	Bacteria	n	n	uncultured bacterium
JQ998485	467	5	418	0	95%	95%	197346995	Bacteria	n	n	uncultured bacterium
JQ998026	307	5	273	1E-137	100%	100%	197347462	Bacteria	n	n	uncultured bacterium
JQ998261	386	18	291	5E-137	99%	99%	197349532	Bacteria	n	n	uncultured bacterium
JQ998851	541	5	455	0	97%	97%	197350011	Bacteria	n	n	uncultured bacterium
JQ998296	398	7	365	1E-152	94%	94%	197350154	Bacteria	n	n	uncultured bacterium
JQ997925	263	5	214	6E-65	89%	89%	197350162	Bacteria	n	n	uncultured bacterium
JQ998235	379	5	347	2E-130	91%	91%	197350304	Bacteria	n	n	uncultured bacterium
JQ998379	428	5	383	0	98%	98%	197350861	Bacteria	n	n	uncultured bacterium
JQ998144	350	5	296	8E-125	95%	95%	197351132	Bacteria	n	n	uncultured bacterium
JQ998145	350	5	297	6E-136	97%	97%	197351240	Bacteria	n	n	uncultured bacterium
JQ998806	537	18	534	0	93%	93%	197351291	Bacteria	n	n	uncultured bacterium
JQ998580	500	35	447	1E-169	93%	93%	197351616	Bacteria	n	n	uncultured bacterium
JQ998019	304	5	270	2E-134	100%	100%	197352517	Bacteria	n	n	uncultured bacterium
JQ998996	550	29	516	0	98%	98%	197352847	Bacteria	n	n	uncultured bacterium
JQ998586	501	23	358	5E-153	96%	96%	197353381	Bacteria	n	n	uncultured bacterium
JQ997887	242	5	211	1E-96	98%	98%	197355895	Bacteria	n	n	uncultured bacterium
JQ998363	420	17	355	4E-148	95%	95%	197357969	Bacteria	n	n	uncultured bacterium
JQ998400	435	19	395	8E-151	93%	93%	197358234	Bacteria	n	n	uncultured bacterium
JQ998390	432	14	373	0	99%	99%	197358911	Bacteria	n	n	uncultured bacterium
JQ998900	544	4	500	0	94%	94%	197365599	Bacteria	n	n	uncultured bacterium
JQ998864	542	4	541	0	94%	94%	197365624	Bacteria	n	n	uncultured bacterium
JQ998453	456	71	397	5E-168	100%	100%	197724312	Bacteria	n	n	uncultured bacterium
JQ997991	295	18	260	2E-85	92%	92%	198386168	Bacteria	n	n	uncultured bacterium
JQ998408	438	18	381	0	100%	100%	198386169	Bacteria	n	n	uncultured bacterium
JQ998882	543	4	540	0	95%	95%	198403335	Bacteria	n	n	uncultured bacterium
JQ998182	362	5	326	2E-165	100%	100%	198427020	Bacteria	n	n	uncultured bacterium
JQ998514	478	4	404	0	100%	100%	198447831	Bacteria	n	n	uncultured bacterium
JQ997987	293	4	240	7E-120	100%	100%	199582799	Bacteria	n	n	uncultured bacterium
JQ998768	534	18	533	0	92%	92%	202073332	Bacteria	n	n	uncultured bacterium
JQ998158	353	18	307	5E-147	100%	100%	206583457	Bacteria	n	n	uncultured bacterium
JQ998883	543	18	543	0	99%	99%	207083801	Bacteria	n	n	uncultured bacterium
JQ998380	428	5	210	9E-51	86%	86%	207299102	Bacteria	n	n	uncultured bacterium
JQ997889	244	23	214	3E-93	99%	99%	209165384	Bacteria	n	n	uncultured bacterium
JQ997874	215	5	103	1E-36	96%	96%	209171353	Bacteria	n	n	uncultured bacterium
JQ998930	546	18	544	0	89%	89%	209365353	Bacteria	n	n	uncultured bacterium
JQ999208	669	18	358	2E-164	98%	98%	209915822	Bacteria	n	n	uncultured bacterium
JQ998449	455	16	374	4E-174	98%	98%	209915834	Bacteria	n	n	uncultured bacterium
JQ998255	384	5	338	8E-160	98%	98%	209915940	Bacteria	n	n	uncultured bacterium
JQ998769	534	18	446	0	94%	94%	209915946	Bacteria	n	n	uncultured bacterium
JQ997989	294	3	150	2E-70	100%	100%	209973735	Bacteria	n	n	uncultured bacterium
JQ998901	544	5	488	0	92%	92%	209973762	Bacteria	n	n	uncultured bacterium
JQ998528	482	23	436	0	97%	97%	209973787	Bacteria	n	n	uncultured bacterium
JQ998252	383	4	255	4E-123	99%	99%	209973825	Bacteria	n	n	uncultured bacterium
JQ998965	548	18	539	0	98%	98%	209973833	Bacteria	n	n	uncultured bacterium
JQ998159	353	27	284	2E-96	92%	92%	209973849	Bacteria	n	n	uncultured bacterium
JQ999147	567	17	529	0	97%	97%	209973893	Bacteria	n	n	uncultured bacterium
JQ998135	348	18	260	2E-115	98%	98%	209973941	Bacteria	n	n	uncultured bacterium
JQ998163	356	5	304	8E-150	99%	99%	209974024	Bacteria	n	n	uncultured bacterium
JQ998258	385	5	343	4E-173	99%	99%	209974036	Bacteria	n	n	uncultured bacterium
JQ997917	260	18	74	7E-20	100%	100%	212293829	Bacteria	n	n	uncultured bacterium
JQ998931	546	18	517	0	98%	98%	212725698	Bacteria	n	n	uncultured bacterium
JQ997974	285	5	226	2E-110	100%	100%	213519754	Bacteria	n	n	uncultured bacterium
JQ998498	473	5	413	0	99%	99%	214017338	Bacteria	n	n	uncultured bacterium
JQ998884	543	24	266	6E-123	100%	100%	214018279	Bacteria	n	n	uncultured bacterium
JQ998602	505	18	372	0	100%	100%	214018628	Bacteria	n	n	uncultured bacterium
JQ999090	558	5	547	0	97%	97%	214019092	Bacteria	n	n	uncultured bacterium
JQ997904	252	21	204	6E-65	92%	92%	214019655	Bacteria	n	n	uncultured bacterium
JQ998210	370	12	325	3E-159	99%	99%	214021245	Bacteria	n	n	uncultured bacterium
JQ998098	333	5	272	3E-114	95%	95%	214023606	Bacteria	n	n	uncultured bacterium

JQ997880	238	5	195	9E-93	99%	99%	214024174	Bacteria	n	n	uncultured bacterium
JQ997926	264	5	232	1E-111	99%	99%	214025032	Bacteria	n	n	uncultured bacterium
JQ998387	431	51	387	1E-168	99%	99%	214025113	Bacteria	n	n	uncultured bacterium
JQ998629	513	18	458	0	100%	100%	214025124	Bacteria	n	n	uncultured bacterium
JQ998535	484	5	435	0	95%	95%	214025498	Bacteria	n	n	uncultured bacterium
JQ998594	503	57	456	3E-110	87%	87%	214026621	Bacteria	n	n	uncultured bacterium
JQ997878	233	5	219	4E-106	100%	100%	215267990	Bacteria	n	n	uncultured bacterium
JQ998778	534	18	529	0	98%	98%	215268007	Bacteria	n	n	uncultured bacterium
JQ998440	450	5	350	3E-170	98%	98%	215268914	Bacteria	n	n	uncultured bacterium
JQ998550	489	5	425	0	100%	100%	215268983	Bacteria	n	n	uncultured bacterium
JQ999058	555	17	542	0	93%	93%	215269445	Bacteria	n	n	uncultured bacterium
JQ998358	418	5	107	1E-43	99%	99%	215269470	Bacteria	n	n	uncultured bacterium
JQ998826	538	15	533	3E-175	88%	88%	215269500	Bacteria	n	n	uncultured bacterium
JQ998645	516	23	461	0	99%	99%	215269508	Bacteria	n	n	uncultured bacterium
JQ998814	537	5	535	0	100%	100%	215269603	Bacteria	n	n	uncultured bacterium
JQ998658	518	18	514	0	100%	100%	215269622	Bacteria	n	n	uncultured bacterium
JQ998350	415	5	378	2E-151	93%	93%	215269784	Bacteria	n	n	uncultured bacterium
JQ998013	301	15	257	3E-118	99%	99%	215270156	Bacteria	n	n	uncultured bacterium
JQ998892	543	10	543	0	98%	98%	215270289	Bacteria	n	n	uncultured bacterium
JQ998190	364	5	313	6E-126	94%	94%	215270482	Bacteria	n	n	uncultured bacterium
JQ998226	376	5	317	1E-158	99%	99%	215271112	Bacteria	n	n	uncultured bacterium
JQ998593	502	15	401	0	98%	98%	215271225	Bacteria	n	n	uncultured bacterium
JQ998142	349	18	315	2E-150	99%	99%	215271342	Bacteria	n	n	uncultured bacterium
JQ998478	465	5	414	0	100%	100%	215271352	Bacteria	n	n	uncultured bacterium
JQ998997	550	18	322	1E-114	92%	92%	215480383	Bacteria	n	n	uncultured bacterium
JQ998362	419	5	362	5E-177	98%	98%	217272751	Bacteria	n	n	uncultured bacterium
JQ998620	509	429	495	2E-23	99%	99%	217323685	Bacteria	n	n	uncultured bacterium
JQ998604	506	18	460	0	99%	99%	217416996	Bacteria	n	n	uncultured bacterium
JQ999177	578	18	573	0	92%	92%	217417011	Bacteria	n	n	uncultured bacterium
JQ998547	487	50	450	4E-149	91%	91%	217417036	Bacteria	n	n	uncultured bacterium
JQ998902	544	18	540	0	95%	95%	217417113	Bacteria	n	n	uncultured bacterium
JQ998277	392	3	334	7E-171	100%	100%	217417131	Bacteria	n	n	uncultured bacterium
JQ998998	550	3	539	0	92%	92%	217417141	Bacteria	n	n	uncultured bacterium
JQ998492	469	4	419	2E-152	91%	91%	217417149	Bacteria	n	n	uncultured bacterium
JQ997928	265	4	248	1E-117	98%	98%	217417286	Bacteria	n	n	uncultured bacterium
JQ998807	537	5	536	0	97%	97%	217417293	Bacteria	n	n	uncultured bacterium
JQ998315	404	5	337	4E-163	98%	98%	217417294	Bacteria	n	n	uncultured bacterium
JQ998136	348	18	292	6E-106	92%	92%	217417308	Bacteria	n	n	uncultured bacterium
JQ998009	300	13	267	3E-78	88%	88%	217417319	Bacteria	n	n	uncultured bacterium
JQ997961	280	5	230	2E-110	99%	99%	217417401	Bacteria	n	n	uncultured bacterium
JQ998553	490	8	418	0	96%	96%	218100559	Bacteria	n	n	uncultured bacterium
JQ998858	541	51	540	0	98%	98%	218411140	Bacteria	n	n	uncultured bacterium
JQ998770	534	17	518	0	96%	96%	218686562	Bacteria	n	n	uncultured bacterium
JQ998048	315	20	270	1E-117	98%	98%	219893613	Bacteria	n	n	uncultured bacterium
JQ999001	550	18	546	0	97%	97%	219906426	Bacteria	n	n	uncultured bacterium
JQ998341	412	18	88	4E-24	97%	97%	220937850	Bacteria	n	n	uncultured bacterium
JQ999059	555	18	550	0	99%	99%	220980864	Bacteria	n	n	uncultured bacterium
JQ998852	541	6	500	0	94%	94%	222090156	Bacteria	n	n	uncultured bacterium
JQ997898	248	23	215	2E-70	92%	92%	222090357	Bacteria	n	n	uncultured bacterium
JQ998020	304	15	253	3E-114	98%	98%	222101776	Bacteria	n	n	uncultured bacterium
JQ998146	350	17	305	8E-120	94%	94%	222101803	Bacteria	n	n	uncultured bacterium
JQ998815	537	3	532	0	98%	98%	222427076	Bacteria	n	n	uncultured bacterium
JQ998256	384	17	347	2E-155	97%	97%	222432210	Bacteria	n	n	uncultured bacterium
JQ997929	265	18	198	4E-77	96%	96%	223675333	Bacteria	n	n	uncultured bacterium
JQ998240	380	33	323	9E-140	98%	98%	223675605	Bacteria	n	n	uncultured bacterium
JQ998224	375	24	330	5E-142	97%	97%	223676588	Bacteria	n	n	uncultured bacterium
JQ999074	557	3	556	0	97%	97%	223676678	Bacteria	n	n	uncultured bacterium
JQ998401	435	5	372	3E-179	98%	98%	223676782	Bacteria	n	n	uncultured bacterium
JQ998705	527	24	481	0	98%	98%	223677097	Bacteria	n	n	uncultured bacterium
JQ998834	539	5	539	0	96%	96%	223677414	Bacteria	n	n	uncultured bacterium

JQ998259	385	15	257	1E-98	94%	94%	223677752	Bacteria	n	n	uncultured bacterium
JQ998782	535	5	531	0	95%	95%	223677840	Bacteria	n	n	uncultured bacterium
JQ999048	555	5	552	0	99%	99%	223678213	Bacteria	n	n	uncultured bacterium
JQ998278	392	5	326	5E-157	98%	98%	223678327	Bacteria	n	n	uncultured bacterium
JQ998337	411	4	235	1E-33	80%	80%	223678817	Bacteria	n	n	uncultured bacterium
JQ998306	402	23	369	4E-163	97%	97%	223678949	Bacteria	n	n	uncultured bacterium
JQ998660	519	18	519	0	100%	100%	223678952	Bacteria	n	n	uncultured bacterium
JQ998419	444	5	375	0	99%	99%	223679056	Bacteria	n	n	uncultured bacterium
JQ998403	437	18	392	0	100%	100%	223679058	Bacteria	n	n	uncultured bacterium
JQ999217	762	5	281	2E-129	97%	97%	223679096	Bacteria	n	n	uncultured bacterium
JQ998706	527	17	526	0	100%	100%	223679113	Bacteria	n	n	uncultured bacterium
JQ998653	518	181	516	1E-169	99%	99%	223679141	Bacteria	n	n	uncultured bacterium
JQ998555	491	5	435	0	96%	96%	223679337	Bacteria	n	n	uncultured bacterium
JQ998060	319	18	276	6E-116	96%	96%	223679587	Bacteria	n	n	uncultured bacterium
JQ998414	441	4	105	2E-27	90%	90%	223679739	Bacteria	n	n	uncultured bacterium
JQ998292	397	18	357	2E-136	92%	92%	223679771	Bacteria	n	n	uncultured bacterium
JQ998819	538	5	535	0	97%	97%	223679788	Bacteria	n	n	uncultured bacterium
JQ998795	536	20	532	0	97%	97%	223679898	Bacteria	n	n	uncultured bacterium
JQ997957	276	3	229	1E-112	100%	100%	223679919	Bacteria	n	n	uncultured bacterium
JQ998253	383	18	298	3E-144	100%	100%	223680075	Bacteria	n	n	uncultured bacterium
JQ999161	572	6	572	0	89%	89%	223680296	Bacteria	n	n	uncultured bacterium
JQ998228	377	18	321	2E-150	99%	99%	223680400	Bacteria	n	n	uncultured bacterium
JQ999006	551	18	547	0	99%	99%	223680555	Bacteria	n	n	uncultured bacterium
JQ998966	548	4	545	0	100%	100%	223680624	Bacteria	n	n	uncultured bacterium
JQ999107	560	5	473	0	95%	95%	223680699	Bacteria	n	n	uncultured bacterium
JQ997907	254	5	208	6E-100	100%	100%	223680762	Bacteria	n	n	uncultured bacterium
JQ999015	552	20	496	0	94%	94%	223680902	Bacteria	n	n	uncultured bacterium
JQ998510	477	12	406	0	99%	99%	223680920	Bacteria	n	n	uncultured bacterium
JQ998588	502	23	454	0	98%	98%	223680969	Bacteria	n	n	uncultured bacterium
JQ998199	367	5	320	1E-158	99%	99%	223680981	Bacteria	n	n	uncultured bacterium
JQ999121	562	16	558	0	100%	100%	223681376	Bacteria	n	n	uncultured bacterium
JQ998042	312	25	277	6E-106	95%	95%	223681393	Bacteria	n	n	uncultured bacterium
JQ998438	450	5	384	6E-157	94%	94%	223681492	Bacteria	n	n	uncultured bacterium
JQ998723	529	5	529	0	98%	98%	223681557	Bacteria	n	n	uncultured bacterium
JQ998345	414	18	369	1E-163	97%	97%	223681668	Bacteria	n	n	uncultured bacterium
JQ998569	495	18	176	1E-55	93%	93%	223681735	Bacteria	n	n	uncultured bacterium
JQ998507	476	2	304	8E-151	99%	99%	223681892	Bacteria	n	n	uncultured bacterium
JQ998123	343	31	298	5E-137	100%	100%	223682235	Bacteria	n	n	uncultured bacterium
JQ999049	555	18	551	0	98%	98%	223682240	Bacteria	n	n	uncultured bacterium
JQ997960	279	5	227	2E-110	100%	100%	223682363	Bacteria	n	n	uncultured bacterium
JQ998338	411	5	378	0	99%	99%	223682414	Bacteria	n	n	uncultured bacterium
JQ998642	516	13	457	0	99%	99%	223682419	Bacteria	n	n	uncultured bacterium
JQ998560	493	5	108	8E-37	95%	95%	223682843	Bacteria	n	n	uncultured bacterium
JQ998444	453	97	413	8E-156	98%	98%	223683254	Bacteria	n	n	uncultured bacterium
JQ998140	349	18	304	1E-147	100%	100%	223683434	Bacteria	n	n	uncultured bacterium
JQ999127	563	4	560	0	98%	98%	223683523	Bacteria	n	n	uncultured bacterium
JQ997915	258	4	209	5E-101	100%	100%	223683690	Bacteria	n	n	uncultured bacterium
JQ998710	528	17	525	0	98%	98%	223684138	Bacteria	n	n	uncultured bacterium
JQ997945	271	3	222	2E-100	97%	97%	223685588	Bacteria	n	n	uncultured bacterium
JQ998342	412	18	359	5E-177	100%	100%	223685657	Bacteria	n	n	uncultured bacterium
JQ999034	554	17	550	0	93%	93%	223685961	Bacteria	n	n	uncultured bacterium
JQ998711	528	5	528	0	99%	99%	223686127	Bacteria	n	n	uncultured bacterium
JQ997913	256	5	222	5E-76	91%	91%	223686201	Bacteria	n	n	uncultured bacterium
JQ998668	522	19	522	0	98%	98%	223688814	Bacteria	n	n	uncultured bacterium
JQ997992	295	4	229	4E-112	100%	100%	223689241	Bacteria	n	n	uncultured bacterium
JQ998771	534	6	527	0	98%	98%	223689728	Bacteria	n	n	uncultured bacterium
JQ998045	315	3	226	1E-77	91%	91%	223689798	Bacteria	n	n	uncultured bacterium
JQ999116	561	71	547	0	98%	98%	223694958	Bacteria	n	n	uncultured bacterium
JQ998783	535	36	524	0	99%	99%	223695178	Bacteria	n	n	uncultured bacterium
JQ998442	451	18	411	8E-161	93%	93%	223695266	Bacteria	n	n	uncultured bacterium

JQ998031	308	15	263	7E-125	100%	100%	223695358	Bacteria	n	n	uncultured bacterium
JQ999050	555	18	552	0	96%	96%	223695555	Bacteria	n	n	uncultured bacterium
JQ998445	453	18	397	0	100%	100%	223695721	Bacteria	n	n	uncultured bacterium
JQ998561	493	5	436	0	95%	95%	223695747	Bacteria	n	n	uncultured bacterium
JQ998772	534	14	531	0	93%	93%	223695950	Bacteria	n	n	uncultured bacterium
JQ998977	549	18	526	0	93%	93%	223696074	Bacteria	n	n	uncultured bacterium
JQ998027	307	25	272	5E-111	96%	96%	223696182	Bacteria	n	n	uncultured bacterium
JQ998105	336	5	280	2E-141	100%	100%	223696209	Bacteria	n	n	uncultured bacterium
JQ999165	574	8	572	0	91%	91%	223696223	Bacteria	n	n	uncultured bacterium
JQ998420	444	5	380	1E-164	95%	95%	223696268	Bacteria	n	n	uncultured bacterium
JQ998307	402	18	351	1E-168	99%	99%	223696324	Bacteria	n	n	uncultured bacterium
JQ998978	549	5	544	0	96%	96%	223696346	Bacteria	n	n	uncultured bacterium
JQ998707	527	3	514	0	100%	100%	223696671	Bacteria	n	n	uncultured bacterium
JQ999051	555	18	554	0	98%	98%	223696683	Bacteria	n	n	uncultured bacterium
JQ998197	366	18	335	4E-163	100%	100%	223696698	Bacteria	n	n	uncultured bacterium
JQ999016	552	4	548	0	99%	99%	223696706	Bacteria	n	n	uncultured bacterium
JQ998932	546	24	540	0	91%	91%	223696744	Bacteria	n	n	uncultured bacterium
JQ999166	574	5	549	0	94%	94%	223696748	Bacteria	n	n	uncultured bacterium
JQ998225	376	18	332	4E-163	100%	100%	223696749	Bacteria	n	n	uncultured bacterium
JQ998241	380	18	327	2E-160	100%	100%	223696760	Bacteria	n	n	uncultured bacterium
JQ998244	381	18	327	1E-157	99%	99%	223696843	Bacteria	n	n	uncultured bacterium
JQ999211	681	20	115	4E-41	100%	100%	223696866	Bacteria	n	n	uncultured bacterium
JQ998129	346	5	281	5E-142	100%	100%	223954980	Bacteria	n	n	uncultured bacterium
JQ998784	535	5	531	0	91%	91%	223955431	Bacteria	n	n	uncultured bacterium
JQ998916	545	15	495	0	100%	100%	224549126	Bacteria	n	n	uncultured bacterium
JQ998748	532	18	478	0	100%	100%	224555168	Bacteria	n	n	uncultured bacterium
JQ999180	579	96	577	5E-154	88%	88%	224566517	Bacteria	n	n	uncultured bacterium
JQ998503	475	24	422	0	98%	98%	224569127	Bacteria	n	n	uncultured bacterium
JQ998917	545	16	524	0	100%	100%	224569165	Bacteria	n	n	uncultured bacterium
JQ998167	358	26	304	2E-126	96%	96%	224569174	Bacteria	n	n	uncultured bacterium
JQ998301	400	13	360	2E-141	93%	93%	224569195	Bacteria	n	n	uncultured bacterium
JQ998773	534	18	291	2E-138	100%	100%	224569580	Bacteria	n	n	uncultured bacterium
JQ999099	559	5	521	0	94%	94%	224569637	Bacteria	n	n	uncultured bacterium
JQ998676	523	16	468	0	100%	100%	224569653	Bacteria	n	n	uncultured bacterium
JQ998112	339	160	307	5E-67	99%	99%	224569711	Bacteria	n	n	uncultured bacterium
JQ998305	401	5	325	2E-166	100%	100%	224569824	Bacteria	n	n	uncultured bacterium
JQ998617	508	26	353	3E-170	100%	100%	224569839	Bacteria	n	n	uncultured bacterium
JQ998329	407	35	329	3E-150	100%	100%	224569899	Bacteria	n	n	uncultured bacterium
JQ998903	544	2	526	0	95%	95%	224569901	Bacteria	n	n	uncultured bacterium
JQ998308	402	5	303	3E-154	100%	100%	224569914	Bacteria	n	n	uncultured bacterium
JQ998587	501	17	444	0	98%	98%	224569941	Bacteria	n	n	uncultured bacterium
JQ998385	430	19	373	0	100%	100%	224569990	Bacteria	n	n	uncultured bacterium
JQ998219	373	19	341	6E-166	100%	100%	224570013	Bacteria	n	n	uncultured bacterium
JQ998885	543	5	534	0	99%	99%	224570029	Bacteria	n	n	uncultured bacterium
JQ999201	620	5	154	1E-40	88%	88%	224570038	Bacteria	n	n	uncultured bacterium
JQ998933	546	5	468	0	93%	93%	224570044	Bacteria	n	n	uncultured bacterium
JQ998835	539	21	538	0	93%	93%	224570046	Bacteria	n	n	uncultured bacterium
JQ999007	551	18	550	0	97%	97%	224570047	Bacteria	n	n	uncultured bacterium
JQ999218	762	5	181	7E-64	93%	93%	224570055	Bacteria	n	n	uncultured bacterium
JQ998712	528	17	528	0	99%	99%	224570061	Bacteria	n	n	uncultured bacterium
JQ998046	315	5	275	2E-135	99%	99%	224570079	Bacteria	n	n	uncultured bacterium
JQ998106	336	4	252	6E-116	98%	98%	224570085	Bacteria	n	n	uncultured bacterium
JQ998740	531	17	526	0	94%	94%	224570113	Bacteria	n	n	uncultured bacterium
JQ999065	556	3	554	0	98%	98%	224570119	Bacteria	n	n	uncultured bacterium
JQ999100	559	259	556	1E-149	99%	99%	224570132	Bacteria	n	n	uncultured bacterium
JQ998669	522	5	516	0	97%	97%	224570156	Bacteria	n	n	uncultured bacterium
JQ997990	294	5	261	5E-131	100%	100%	224570180	Bacteria	n	n	uncultured bacterium
JQ998774	534	5	530	0	100%	100%	224570188	Bacteria	n	n	uncultured bacterium
JQ998309	402	5	334	1E-163	99%	99%	224570202	Bacteria	n	n	uncultured bacterium
JQ998322	406	125	351	2E-102	97%	97%	224570223	Bacteria	n	n	uncultured bacterium

JQ997962	280	18	238	5E-111	100%	100%	224570225	Bacteria	n	n	uncultured bacterium
JQ998264	387	18	337	2E-125	93%	93%	224570227	Bacteria	n	n	uncultured bacterium
JQ998168	358	5	286	1E-137	99%	99%	224611899	Bacteria	n	n	uncultured bacterium
JQ998552	490	5	423	0	100%	100%	224712074	Bacteria	n	n	uncultured bacterium
JQ999008	551	21	545	0	98%	98%	224714764	Bacteria	n	n	uncultured bacterium
JQ998654	518	19	516	0	97%	97%	225302504	Bacteria	n	n	uncultured bacterium
JQ998612	507	5	445	0	100%	100%	225302521	Bacteria	n	n	uncultured bacterium
JQ998570	495	5	452	0	94%	94%	225302626	Bacteria	n	n	uncultured bacterium
JQ998904	544	21	532	0	99%	99%	225302678	Bacteria	n	n	uncultured bacterium
JQ998393	433	18	376	7E-161	95%	95%	225302713	Bacteria	n	n	uncultured bacterium
JQ998075	324	157	293	1E-48	94%	94%	225337162	Bacteria	n	n	uncultured bacterium
JQ997977	287	24	236	7E-105	100%	100%	225338363	Bacteria	n	n	uncultured bacterium
JQ997994	296	5	251	2E-110	96%	96%	225338711	Bacteria	n	n	uncultured bacterium
JQ997882	240	14	106	6E-30	94%	94%	225382292	Bacteria	n	n	uncultured bacterium
JQ998504	475	1	403	5E-168	94%	94%	225382550	Bacteria	n	n	uncultured bacterium
JQ998893	543	23	519	0	97%	97%	225618928	Bacteria	n	n	uncultured bacterium
JQ998820	538	15	529	0	94%	94%	225936324	Bacteria	n	n	uncultured bacterium
JQ998749	532	5	185	4E-70	94%	94%	226350595	Bacteria	n	n	uncultured bacterium
JQ998262	386	18	340	6E-166	100%	100%	226351268	Bacteria	n	n	uncultured bacterium
JQ998369	423	4	378	3E-150	93%	93%	226429024	Bacteria	n	n	uncultured bacterium
JQ998967	548	18	516	0	99%	99%	226430279	Bacteria	n	n	uncultured bacterium
JQ998431	448	4	392	1E-124	88%	88%	226447054	Bacteria	n	n	uncultured bacterium
JQ997998	297	18	57	2E-10	100%	100%	227437797	Bacteria	n	n	uncultured bacterium
JQ998198	366	18	321	5E-157	100%	100%	227937314	Bacteria	n	n	uncultured bacterium
JQ998153	352	21	199	1E-83	99%	99%	228480902	Bacteria	n	n	uncultured bacterium
JQ999172	575	5	272	4E-125	97%	97%	229428720	Bacteria	n	n	uncultured bacterium
JQ998346	414	5	381	3E-160	94%	94%	229428732	Bacteria	n	n	uncultured bacterium
JQ998865	542	5	540	0	96%	96%	229428738	Bacteria	n	n	uncultured bacterium
JQ998065	320	5	268	2E-126	98%	98%	229428747	Bacteria	n	n	uncultured bacterium
JQ997936	268	18	232	6E-100	98%	98%	229428758	Bacteria	n	n	uncultured bacterium
JQ999091	558	3	381	2E-123	89%	89%	229428778	Bacteria	n	n	uncultured bacterium
JQ998160	353	4	295	3E-144	99%	99%	229428780	Bacteria	n	n	uncultured bacterium
JQ998670	522	18	488	0	95%	95%	229428782	Bacteria	n	n	uncultured bacterium
JQ998632	514	20	513	0	97%	97%	229428785	Bacteria	n	n	uncultured bacterium
JQ998750	532	21	528	0	94%	94%	229428786	Bacteria	n	n	uncultured bacterium
JQ998016	303	18	253	1E-87	92%	92%	229428847	Bacteria	n	n	uncultured bacterium
JQ999035	554	279	514	5E-119	100%	100%	229428882	Bacteria	n	n	uncultured bacterium
JQ998249	382	5	339	7E-141	94%	94%	237687615	Bacteria	n	n	uncultured bacterium
JQ998302	400	19	350	1E-152	96%	96%	237687664	Bacteria	n	n	uncultured bacterium
JQ998493	469	17	423	0	97%	97%	237687668	Bacteria	n	n	uncultured bacterium
JQ998148	351	5	308	5E-147	98%	98%	237687669	Bacteria	n	n	uncultured bacterium
JQ998886	543	5	540	0	100%	100%	237774863	Bacteria	n	n	uncultured bacterium
JQ998791	535	4	483	0	96%	96%	237934376	Bacteria	n	n	uncultured bacterium
JQ998061	319	15	280	3E-114	95%	95%	238068056	Bacteria	n	n	uncultured bacterium
JQ998066	320	5	251	4E-117	98%	98%	238068457	Bacteria	n	n	uncultured bacterium
JQ998775	534	5	529	0	93%	93%	238068520	Bacteria	n	n	uncultured bacterium
JQ998057	318	18	270	7E-115	97%	97%	238068847	Bacteria	n	n	uncultured bacterium
JQ998729	530	230	530	2E-118	93%	93%	238068889	Bacteria	n	n	uncultured bacterium
JQ999206	659	5	91	2E-23	92%	92%	238068929	Bacteria	n	n	uncultured bacterium
JQ999203	630	17	163	2E-69	100%	100%	238254033	Bacteria	n	n	uncultured bacterium
JQ998648	517	2	477	0	100%	100%	238254594	Bacteria	n	n	uncultured bacterium
JQ998149	351	18	319	6E-136	96%	96%	238256387	Bacteria	n	n	uncultured bacterium
JQ998887	543	18	543	0	100%	100%	238258794	Bacteria	n	n	uncultured bacterium
JQ998388	431	5	375	3E-159	94%	94%	238260512	Bacteria	n	n	uncultured bacterium
JQ997966	283	10	233	2E-105	98%	98%	238260516	Bacteria	n	n	uncultured bacterium
JQ998979	549	18	544	0	100%	100%	238262069	Bacteria	n	n	uncultured bacterium
JQ999122	562	5	558	0	97%	97%	238262159	Bacteria	n	n	uncultured bacterium
JQ998980	549	283	544	6E-108	95%	95%	238262473	Bacteria	n	n	uncultured bacterium
JQ998519	479	26	397	0	98%	98%	238262864	Bacteria	n	n	uncultured bacterium
JQ998290	396	5	363	0	100%	100%	238262966	Bacteria	n	n	uncultured bacterium

JQ999117	561	4	556	0	99%	99%	238263260	Bacteria	n	n	uncultured bacterium
JQ998043	313	5	230	2E-100	96%	96%	238263285	Bacteria	n	n	uncultured bacterium
JQ998934	546	18	541	0	99%	99%	238263468	Bacteria	n	n	uncultured bacterium
JQ998982	549	5	537	0	99%	99%	238263771	Bacteria	n	n	uncultured bacterium
JQ998808	537	18	533	0	97%	97%	238263814	Bacteria	n	n	uncultured bacterium
JQ998951	547	24	544	0	97%	97%	238263848	Bacteria	n	n	uncultured bacterium
JQ998124	343	5	317	2E-155	99%	99%	238263874	Bacteria	n	n	uncultured bacterium
JQ998370	423	1	352	3E-169	97%	97%	238265232	Bacteria	n	n	uncultured bacterium
JQ998028	307	17	232	6E-106	100%	100%	238265477	Bacteria	n	n	uncultured bacterium
JQ998119	342	5	294	3E-149	100%	100%	238266272	Bacteria	n	n	uncultured bacterium
JQ997967	283	19	238	2E-100	97%	97%	238268651	Bacteria	n	n	uncultured bacterium
JQ998260	385	17	340	1E-162	99%	99%	238269172	Bacteria	n	n	uncultured bacterium
JQ998785	535	5	501	0	100%	100%	238269688	Bacteria	n	n	uncultured bacterium
JQ997968	283	3	245	3E-108	96%	96%	238270075	Bacteria	n	n	uncultured bacterium
JQ998311	403	4	281	6E-122	96%	96%	238270979	Bacteria	n	n	uncultured bacterium
JQ998193	365	18	138	1E-53	99%	99%	238271851	Bacteria	n	n	uncultured bacterium
JQ998054	317	18	280	9E-129	99%	99%	238273315	Bacteria	n	n	uncultured bacterium
JQ998776	534	5	493	0	98%	98%	238274455	Bacteria	n	n	uncultured bacterium
JQ998450	455	24	371	4E-179	100%	100%	238274745	Bacteria	n	n	uncultured bacterium
JQ998001	298	14	249	4E-117	100%	100%	238275219	Bacteria	n	n	uncultured bacterium
JQ998101	334	17	301	7E-140	99%	99%	238275250	Bacteria	n	n	uncultured bacterium
JQ998540	485	5	137	7E-62	100%	100%	238275715	Bacteria	n	n	uncultured bacterium
JQ997985	292	5	259	9E-109	95%	95%	238275802	Bacteria	n	n	uncultured bacterium
JQ998357	417	4	366	0	100%	100%	238276735	Bacteria	n	n	uncultured bacterium
JQ998531	483	5	328	1E-164	99%	99%	238276739	Bacteria	n	n	uncultured bacterium
JQ998844	540	24	537	0	98%	98%	238276766	Bacteria	n	n	uncultured bacterium
JQ998853	541	5	500	0	98%	98%	238276795	Bacteria	n	n	uncultured bacterium
JQ999128	563	18	561	0	100%	100%	238277365	Bacteria	n	n	uncultured bacterium
JQ999209	679	4	375	0	100%	100%	238277647	Bacteria	n	n	uncultured bacterium
JQ998524	480	17	386	8E-161	95%	95%	238277655	Bacteria	n	n	uncultured bacterium
JQ997951	273	5	231	2E-95	95%	95%	238277784	Bacteria	n	n	uncultured bacterium
JQ998002	298	5	241	4E-102	96%	96%	238278012	Bacteria	n	n	uncultured bacterium
JQ998396	434	18	393	1E-163	95%	95%	238278031	Bacteria	n	n	uncultured bacterium
JQ998378	427	17	385	9E-170	96%	96%	238278034	Bacteria	n	n	uncultured bacterium
JQ998643	516	3	422	0	96%	96%	238278039	Bacteria	n	n	uncultured bacterium
JQ998082	326	5	282	1E-142	100%	100%	238278081	Bacteria	n	n	uncultured bacterium
JQ998349	415	5	257	6E-122	98%	98%	238278110	Bacteria	n	n	uncultured bacterium
JQ998141	349	5	300	3E-149	99%	99%	238278174	Bacteria	n	n	uncultured bacterium
JQ998935	546	18	542	0	100%	100%	238279128	Bacteria	n	n	uncultured bacterium
JQ998671	522	5	481	0	99%	99%	238280160	Bacteria	n	n	uncultured bacterium
JQ998845	540	18	538	0	98%	98%	238280720	Bacteria	n	n	uncultured bacterium
JQ998263	386	21	342	2E-135	94%	94%	238281878	Bacteria	n	n	uncultured bacterium
JQ998854	541	7	538	0	99%	99%	238286296	Bacteria	n	n	uncultured bacterium
JQ997970	284	5	246	1E-107	96%	96%	238286743	Bacteria	n	n	uncultured bacterium
JQ998200	367	18	323	4E-158	100%	100%	238286788	Bacteria	n	n	uncultured bacterium
JQ998918	545	17	516	0	95%	95%	238286790	Bacteria	n	n	uncultured bacterium
JQ998229	377	28	326	1E-137	97%	97%	238286826	Bacteria	n	n	uncultured bacterium
JQ999026	553	18	551	0	100%	100%	238286863	Bacteria	n	n	uncultured bacterium
JQ997927	264	5	198	4E-92	99%	99%	238289810	Bacteria	n	n	uncultured bacterium
JQ998866	542	5	540	0	96%	96%	238292394	Bacteria	n	n	uncultured bacterium
JQ998630	513	3	457	0	100%	100%	238292646	Bacteria	n	n	uncultured bacterium
JQ998093	330	23	285	1E-127	98%	98%	238292712	Bacteria	n	n	uncultured bacterium
JQ997959	277	19	246	6E-110	99%	99%	238293955	Bacteria	n	n	uncultured bacterium
JQ999193	604	5	79	1E-20	93%	93%	238295305	Bacteria	n	n	uncultured bacterium
JQ998809	537	13	497	0	100%	100%	238295567	Bacteria	n	n	uncultured bacterium
JQ999118	561	114	554	0	94%	94%	238296060	Bacteria	n	n	uncultured bacterium
JQ999123	562	5	559	0	99%	99%	238296317	Bacteria	n	n	uncultured bacterium
JQ998397	434	17	389	1E-158	94%	94%	238297072	Bacteria	n	n	uncultured bacterium
JQ998291	396	18	325	3E-159	100%	100%	238297462	Bacteria	n	n	uncultured bacterium
JQ998613	507	4	461	0	100%	100%	238297600	Bacteria	n	n	uncultured bacterium

JQ998133	347	4	315	8E-160	100%	100%	238297688	Bacteria	n	n	uncultured bacterium
JQ998952	547	17	545	0	98%	98%	238299775	Bacteria	n	n	uncultured bacterium
JQ998274	390	144	339	3E-95	99%	99%	238299976	Bacteria	n	n	uncultured bacterium
JQ998936	546	5	542	0	98%	98%	238300686	Bacteria	n	n	uncultured bacterium
JQ998303	400	5	284	4E-123	96%	96%	238301381	Bacteria	n	n	uncultured bacterium
JQ998636	515	17	467	0	100%	100%	238301499	Bacteria	n	n	uncultured bacterium
JQ997995	296	18	249	4E-112	99%	99%	238301575	Bacteria	n	n	uncultured bacterium
JQ998983	549	17	536	0	94%	94%	238301699	Bacteria	n	n	uncultured bacterium
JQ998282	393	17	246	3E-84	92%	92%	238302245	Bacteria	n	n	uncultured bacterium
JQ998919	545	32	279	2E-122	99%	99%	238302248	Bacteria	n	n	uncultured bacterium
JQ998394	433	18	396	0	99%	99%	238302270	Bacteria	n	n	uncultured bacterium
JQ999017	552	5	552	0	95%	95%	238302336	Bacteria	n	n	uncultured bacterium
JQ998796	536	5	534	0	99%	99%	238302651	Bacteria	n	n	uncultured bacterium
JQ998741	531	4	527	0	100%	100%	238302818	Bacteria	n	n	uncultured bacterium
JQ998663	520	18	452	0	100%	100%	238302994	Bacteria	n	n	uncultured bacterium
JQ998937	546	5	543	0	95%	95%	238303284	Bacteria	n	n	uncultured bacterium
JQ998542	486	1	454	0	99%	99%	238303306	Bacteria	n	n	uncultured bacterium
JQ998477	465	23	378	0	99%	99%	238303310	Bacteria	n	n	uncultured bacterium
JQ998005	299	18	252	4E-102	96%	96%	238303622	Bacteria	n	n	uncultured bacterium
JQ998022	305	17	259	1E-112	98%	98%	238303655	Bacteria	n	n	uncultured bacterium
JQ998173	359	18	320	2E-156	100%	100%	238304075	Bacteria	n	n	uncultured bacterium
JQ999213	689	24	356	2E-128	93%	93%	238305275	Bacteria	n	n	uncultured bacterium
JQ999200	617	5	256	1E-85	91%	91%	238305904	Bacteria	n	n	uncultured bacterium
JQ998999	550	18	374	0	100%	100%	238306208	Bacteria	n	n	uncultured bacterium
JQ999092	558	18	552	0	94%	94%	238306931	Bacteria	n	n	uncultured bacterium
JQ998032	308	3	261	4E-132	100%	100%	238307378	Bacteria	n	n	uncultured bacterium
JQ998051	316	5	270	6E-121	97%	97%	238308732	Bacteria	n	n	uncultured bacterium
JQ998076	324	5	286	2E-101	91%	91%	238308912	Bacteria	n	n	uncultured bacterium
JQ999194	607	18	517	0	94%	94%	238309010	Bacteria	n	n	uncultured bacterium
JQ998344	413	17	386	0	99%	99%	238309257	Bacteria	n	n	uncultured bacterium
JQ998855	541	5	539	0	99%	99%	238309294	Bacteria	n	n	uncultured bacterium
JQ998562	493	19	305	9E-136	98%	98%	238309323	Bacteria	n	n	uncultured bacterium
JQ998230	378	3	329	8E-170	100%	100%	238309341	Bacteria	n	n	uncultured bacterium
JQ998548	487	17	449	6E-172	92%	92%	238309363	Bacteria	n	n	uncultured bacterium
JQ997890	244	5	193	3E-93	100%	100%	238309370	Bacteria	n	n	uncultured bacterium
JQ998920	545	18	545	0	99%	99%	238309389	Bacteria	n	n	uncultured bacterium
JQ998364	421	5	272	2E-126	98%	98%	238309416	Bacteria	n	n	uncultured bacterium
JQ998751	532	4	514	0	100%	100%	238309418	Bacteria	n	n	uncultured bacterium
JQ998867	542	15	535	0	98%	98%	238309460	Bacteria	n	n	uncultured bacterium
JQ998810	537	7	533	0	99%	99%	238309470	Bacteria	n	n	uncultured bacterium
JQ999066	556	5	551	0	94%	94%	238309471	Bacteria	n	n	uncultured bacterium
JQ997937	268	5	232	6E-110	99%	99%	238309482	Bacteria	n	n	uncultured bacterium
JQ998905	544	5	179	2E-62	92%	92%	238309502	Bacteria	n	n	uncultured bacterium
JQ998682	524	4	485	0	100%	100%	238309538	Bacteria	n	n	uncultured bacterium
JQ997891	245	5	213	9E-103	100%	100%	238309566	Bacteria	n	n	uncultured bacterium
JQ997946	271	5	224	2E-110	100%	100%	238309915	Bacteria	n	n	uncultured bacterium
JQ998633	514	18	510	0	99%	99%	238310085	Bacteria	n	n	uncultured bacterium
JQ998953	547	27	546	0	99%	99%	238310341	Bacteria	n	n	uncultured bacterium
JQ998689	525	18	525	0	99%	99%	238310578	Bacteria	n	n	uncultured bacterium
JQ998811	537	16	511	0	99%	99%	238311386	Bacteria	n	n	uncultured bacterium
JQ999036	554	5	548	0	97%	97%	238312124	Bacteria	n	n	uncultured bacterium
JQ999164	573	5	398	8E-107	85%	85%	238312201	Bacteria	n	n	uncultured bacterium
JQ998968	548	17	543	0	98%	98%	238312949	Bacteria	n	n	uncultured bacterium
JQ998724	529	4	477	0	99%	99%	238313108	Bacteria	n	n	uncultured bacterium
JQ998713	528	18	490	0	99%	99%	238313116	Bacteria	n	n	uncultured bacterium
JQ997877	232	1	198	2E-83	96%	96%	238313383	Bacteria	n	n	uncultured bacterium
JQ998888	543	17	538	0	98%	98%	238313396	Bacteria	n	n	uncultured bacterium
JQ998595	503	18	451	0	100%	100%	238313410	Bacteria	n	n	uncultured bacterium
JQ998868	542	5	475	0	100%	100%	238313425	Bacteria	n	n	uncultured bacterium
JQ998398	434	35	346	6E-127	94%	94%	238314296	Bacteria	n	n	uncultured bacterium

JQ998605	506	13	446	0	98%	98%	238314984	Bacteria	n	n	uncultured bacterium
JQ998984	549	5	548	0	99%	99%	238315154	Bacteria	n	n	uncultured bacterium
JQ998821	538	5	535	0	90%	90%	238315752	Bacteria	n	n	uncultured bacterium
JQ998023	305	5	261	5E-131	100%	100%	238316074	Bacteria	n	n	uncultured bacterium
JQ998499	473	17	415	7E-122	88%	88%	238316219	Bacteria	n	n	uncultured bacterium
JQ999018	552	18	547	0	98%	98%	238316220	Bacteria	n	n	uncultured bacterium
JQ999186	591	6	589	0	92%	92%	238317490	Bacteria	n	n	uncultured bacterium
JQ998288	395	24	346	3E-149	97%	97%	238318817	Bacteria	n	n	uncultured bacterium
JQ998283	393	14	360	3E-179	100%	100%	238320101	Bacteria	n	n	uncultured bacterium
JQ998154	352	5	299	6E-101	91%	91%	238320376	Bacteria	n	n	uncultured bacterium
JQ998426	446	4	420	0	100%	100%	238321524	Bacteria	n	n	uncultured bacterium
JQ999067	556	16	497	0	97%	97%	238321974	Bacteria	n	n	uncultured bacterium
JQ999219	865	17	197	2E-65	93%	93%	238321986	Bacteria	n	n	uncultured bacterium
JQ999152	569	83	564	0	100%	100%	238321993	Bacteria	n	n	uncultured bacterium
JQ998889	543	2	509	0	97%	97%	238322379	Bacteria	n	n	uncultured bacterium
JQ998696	526	5	459	0	95%	95%	238322714	Bacteria	n	n	uncultured bacterium
JQ999019	552	5	544	0	100%	100%	238323226	Bacteria	n	n	uncultured bacterium
JQ998284	394	5	358	0	100%	100%	238324411	Bacteria	n	n	uncultured bacterium
JQ998690	525	4	474	0	100%	100%	238324967	Bacteria	n	n	uncultured bacterium
JQ999000	550	18	546	0	99%	99%	238325192	Bacteria	n	n	uncultured bacterium
JQ999108	560	5	557	0	97%	97%	238325493	Bacteria	n	n	uncultured bacterium
JQ998631	513	12	373	2E-177	98%	98%	238326766	Bacteria	n	n	uncultured bacterium
JQ998697	526	18	446	0	94%	94%	238327054	Bacteria	n	n	uncultured bacterium
JQ998466	460	5	384	0	99%	99%	238327290	Bacteria	n	n	uncultured bacterium
JQ999052	555	5	550	0	97%	97%	238327333	Bacteria	n	n	uncultured bacterium
JQ998293	397	17	245	1E-113	100%	100%	238327796	Bacteria	n	n	uncultured bacterium
JQ997999	297	5	253	7E-110	96%	96%	238327953	Bacteria	n	n	uncultured bacterium
JQ998836	539	24	537	0	97%	97%	238329691	Bacteria	n	n	uncultured bacterium
JQ998113	339	18	274	4E-108	95%	95%	238329799	Bacteria	n	n	uncultured bacterium
JQ999197	614	18	608	0	93%	93%	238330195	Bacteria	n	n	uncultured bacterium
JQ997883	240	5	193	3E-93	100%	100%	238330556	Bacteria	n	n	uncultured bacterium
JQ998454	456	1	407	2E-176	95%	95%	238331284	Bacteria	n	n	uncultured bacterium
JQ998822	538	23	511	0	99%	99%	238333276	Bacteria	n	n	uncultured bacterium
JQ998206	369	18	323	2E-156	100%	100%	238333610	Bacteria	n	n	uncultured bacterium
JQ998458	457	5	408	0	96%	96%	238333962	Bacteria	n	n	uncultured bacterium
JQ999129	563	17	546	0	100%	100%	238333963	Bacteria	n	n	uncultured bacterium
JQ999053	555	5	553	0	99%	99%	238334040	Bacteria	n	n	uncultured bacterium
JQ999167	574	18	378	0	99%	99%	238334087	Bacteria	n	n	uncultured bacterium
JQ998265	387	18	347	4E-163	98%	98%	238334867	Bacteria	n	n	uncultured bacterium
JQ998077	324	5	279	2E-121	96%	96%	238335483	Bacteria	n	n	uncultured bacterium
JQ999101	559	17	492	0	100%	100%	238335992	Bacteria	n	n	uncultured bacterium
JQ998285	394	5	344	2E-175	100%	100%	238336141	Bacteria	n	n	uncultured bacterium
JQ997963	280	5	235	6E-115	100%	100%	238336150	Bacteria	n	n	uncultured bacterium
JQ998052	316	5	262	2E-96	92%	92%	238336615	Bacteria	n	n	uncultured bacterium
JQ999141	566	24	563	0	97%	97%	238337104	Bacteria	n	n	uncultured bacterium
JQ998236	379	5	332	2E-170	100%	100%	238337355	Bacteria	n	n	uncultured bacterium
JQ998012	301	28	235	4E-102	100%	100%	238337361	Bacteria	n	n	uncultured bacterium
JQ998921	545	5	540	0	99%	99%	238337387	Bacteria	n	n	uncultured bacterium
JQ999184	582	5	577	0	94%	94%	238337402	Bacteria	n	n	uncultured bacterium
JQ998938	546	5	270	1E-135	100%	100%	238337649	Bacteria	n	n	uncultured bacterium
JQ998589	502	5	451	0	99%	99%	238338805	Bacteria	n	n	uncultured bacterium
JQ999009	551	31	546	0	98%	98%	238340189	Bacteria	n	n	uncultured bacterium
JQ998089	328	5	296	2E-126	95%	95%	238340895	Bacteria	n	n	uncultured bacterium
JQ998330	408	18	375	1E-148	94%	94%	238341047	Bacteria	n	n	uncultured bacterium
JQ998120	342	18	297	2E-125	96%	96%	238341169	Bacteria	n	n	uncultured bacterium
JQ999124	562	18	415	0	99%	99%	238341208	Bacteria	n	n	uncultured bacterium
JQ998985	549	16	545	0	99%	99%	238341728	Bacteria	n	n	uncultured bacterium
JQ998969	548	5	546	0	99%	99%	238341804	Bacteria	n	n	uncultured bacterium
JQ998869	542	17	542	0	99%	99%	238342154	Bacteria	n	n	uncultured bacterium
JQ999130	563	5	562	0	98%	98%	238342263	Bacteria	n	n	uncultured bacterium

JQ998494	469	4	419	0	99%	99%	238342484	Bacteria	n	n	uncultured bacterium
JQ998649	517	12	472	0	92%	92%	238342531	Bacteria	n	n	uncultured bacterium
JQ998072	323	17	279	3E-134	100%	100%	238342541	Bacteria	n	n	uncultured bacterium
JQ998486	467	4	417	0	98%	98%	238342679	Bacteria	n	n	uncultured bacterium
JQ999093	558	18	554	0	97%	97%	238342752	Bacteria	n	n	uncultured bacterium
JQ998245	381	17	338	5E-167	100%	100%	238343654	Bacteria	n	n	uncultured bacterium
JQ998323	406	13	155	8E-66	99%	99%	238343750	Bacteria	n	n	uncultured bacterium
JQ999020	552	5	524	0	93%	93%	238344032	Bacteria	n	n	uncultured bacterium
JQ998014	302	5	270	3E-113	95%	95%	238344125	Bacteria	n	n	uncultured bacterium
JQ998939	546	5	541	0	96%	96%	238344396	Bacteria	n	n	uncultured bacterium
JQ998373	425	5	395	0	99%	99%	238344958	Bacteria	n	n	uncultured bacterium
JQ998044	314	17	284	2E-130	99%	99%	238345059	Bacteria	n	n	uncultured bacterium
JQ998155	352	5	304	8E-150	99%	99%	238346677	Bacteria	n	n	uncultured bacterium
JQ998188	364	5	280	6E-136	99%	99%	238346725	Bacteria	n	n	uncultured bacterium
JQ998574	496	5	435	0	99%	99%	238346876	Bacteria	n	n	uncultured bacterium
JQ998250	382	5	280	5E-132	98%	98%	238347038	Bacteria	n	n	uncultured bacterium
JQ999109	560	5	291	2E-147	100%	100%	238347095	Bacteria	n	n	uncultured bacterium
JQ998598	504	5	442	0	93%	93%	238347314	Bacteria	n	n	uncultured bacterium
JQ998906	544	5	526	0	99%	99%	238347468	Bacteria	n	n	uncultured bacterium
JQ998907	544	18	310	1E-100	91%	91%	238347897	Bacteria	n	n	uncultured bacterium
JQ998970	548	5	548	0	99%	99%	238348588	Bacteria	n	n	uncultured bacterium
JQ998374	425	25	382	0	99%	99%	238348791	Bacteria	n	n	uncultured bacterium
JQ999153	569	5	569	0	98%	98%	238348794	Bacteria	n	n	uncultured bacterium
JQ998176	360	5	304	4E-133	96%	96%	238348865	Bacteria	n	n	uncultured bacterium
JQ999054	555	18	553	0	97%	97%	238349220	Bacteria	n	n	uncultured bacterium
JQ999168	574	18	573	0	99%	99%	238349987	Bacteria	n	n	uncultured bacterium
JQ998094	330	132	289	6E-76	100%	100%	238349990	Bacteria	n	n	uncultured bacterium
JQ998714	528	330	493	3E-71	97%	97%	238350004	Bacteria	n	n	uncultured bacterium
JQ997971	284	4	222	1E-92	96%	96%	238350221	Bacteria	n	n	uncultured bacterium
JQ998021	304	83	273	1E-92	99%	99%	238350299	Bacteria	n	n	uncultured bacterium
JQ998846	540	18	538	0	98%	98%	238350584	Bacteria	n	n	uncultured bacterium
JQ998742	531	19	530	0	96%	96%	238351114	Bacteria	n	n	uncultured bacterium
JQ998954	547	5	368	0	100%	100%	238351604	Bacteria	n	n	uncultured bacterium
JQ997884	241	34	197	2E-64	95%	95%	238351640	Bacteria	n	n	uncultured bacterium
JQ999181	579	289	575	5E-139	98%	98%	238351692	Bacteria	n	n	uncultured bacterium
JQ998312	403	1	357	0	100%	100%	238352483	Bacteria	n	n	uncultured bacterium
JQ997954	275	5	219	2E-105	100%	100%	238352567	Bacteria	n	n	uncultured bacterium
JQ999155	570	5	570	0	96%	96%	238400367	Bacteria	n	n	uncultured bacterium
JQ997930	266	18	221	5E-81	94%	94%	238400424	Bacteria	n	n	uncultured bacterium
JQ998922	545	5	526	0	100%	100%	238401117	Bacteria	n	n	uncultured bacterium
JQ999075	557	17	555	0	99%	99%	238404569	Bacteria	n	n	uncultured bacterium
JQ998459	457	3	409	0	99%	99%	238404723	Bacteria	n	n	uncultured bacterium
JQ997918	260	5	212	1E-101	100%	100%	238406617	Bacteria	n	n	uncultured bacterium
JQ999148	567	5	566	0	99%	99%	238407074	Bacteria	n	n	uncultured bacterium
JQ998870	542	17	539	0	98%	98%	238412051	Bacteria	n	n	uncultured bacterium
JQ998572	495	5	440	0	95%	95%	238412351	Bacteria	n	n	uncultured bacterium
JQ998443	452	2	386	4E-134	90%	90%	238415068	Bacteria	n	n	uncultured bacterium
JQ998847	540	17	535	0	100%	100%	238415407	Bacteria	n	n	uncultured bacterium
JQ998618	508	3	427	0	100%	100%	238415774	Bacteria	n	n	uncultured bacterium
JQ998526	481	16	424	0	98%	98%	238415993	Bacteria	n	n	uncultured bacterium
JQ997983	290	18	245	1E-106	98%	98%	238416037	Bacteria	n	n	uncultured bacterium
JQ998231	378	3	232	4E-108	98%	98%	238416695	Bacteria	n	n	uncultured bacterium
JQ997996	296	17	265	7E-120	98%	98%	238417704	Bacteria	n	n	uncultured bacterium
JQ998743	531	4	459	0	95%	95%	238419619	Bacteria	n	n	uncultured bacterium
JQ998683	524	20	228	6E-98	98%	98%	238421978	Bacteria	n	n	uncultured bacterium
JQ998435	449	15	255	1E-114	98%	98%	238422186	Bacteria	n	n	uncultured bacterium
JQ999135	564	5	563	0	96%	96%	238422435	Bacteria	n	n	uncultured bacterium
JQ998986	549	1	537	0	96%	96%	238423057	Bacteria	n	n	uncultured bacterium
JQ999068	556	29	556	0	97%	97%	238423665	Bacteria	n	n	uncultured bacterium
JQ998268	388	17	357	7E-166	98%	98%	238423676	Bacteria	n	n	uncultured bacterium

JQ998090	328	5	273	3E-129	98%	98%	238426672	Bacteria	n	n	uncultured bacterium
JQ998715	528	18	473	0	100%	100%	238426679	Bacteria	n	n	uncultured bacterium
JQ999154	569	9	567	0	97%	97%	238426699	Bacteria	n	n	uncultured bacterium
JQ998103	335	16	303	2E-90	89%	89%	238426702	Bacteria	n	n	uncultured bacterium
JQ998987	549	5	549	0	100%	100%	238426783	Bacteria	n	n	uncultured bacterium
JQ998908	544	15	543	0	98%	98%	238426795	Bacteria	n	n	uncultured bacterium
JQ999110	560	3	559	0	99%	99%	238426815	Bacteria	n	n	uncultured bacterium
JQ999156	570	5	563	0	93%	93%	238426833	Bacteria	n	n	uncultured bacterium
JQ999037	554	5	551	0	98%	98%	238426835	Bacteria	n	n	uncultured bacterium
JQ999038	554	4	540	0	97%	97%	238426859	Bacteria	n	n	uncultured bacterium
JQ998563	493	4	433	0	95%	95%	238560560	Bacteria	n	n	uncultured bacterium
JQ998725	529	18	482	0	98%	98%	238560573	Bacteria	n	n	uncultured bacterium
JQ998375	425	5	296	8E-101	90%	90%	238770193	Bacteria	n	n	uncultured bacterium
JQ998621	509	5	300	2E-147	99%	99%	238774505	Bacteria	n	n	uncultured bacterium
JQ998475	463	5	436	0	97%	97%	238836153	Bacteria	n	n	uncultured bacterium
JQ998465	459	17	400	6E-122	88%	88%	238836154	Bacteria	n	n	uncultured bacterium
JQ998565	494	18	404	3E-180	97%	97%	238836158	Bacteria	n	n	uncultured bacterium
JQ999178	578	20	571	0	90%	90%	238836167	Bacteria	n	n	uncultured bacterium
JQ997964	281	4	229	2E-94	95%	95%	238836172	Bacteria	n	n	uncultured bacterium
JQ999131	563	393	556	2E-67	96%	96%	238914983	Bacteria	n	n	uncultured bacterium
JQ998121	342	18	294	5E-142	100%	100%	239837090	Bacteria	n	n	uncultured bacterium
JQ997931	266	18	153	8E-64	100%	100%	239837186	Bacteria	n	n	uncultured bacterium
JQ998286	394	1	288	2E-146	100%	100%	239837190	Bacteria	n	n	uncultured bacterium
JQ998024	306	5	243	3E-119	100%	100%	239837210	Bacteria	n	n	uncultured bacterium
JQ998655	518	5	464	0	95%	95%	239837238	Bacteria	n	n	uncultured bacterium
JQ997943	270	4	209	5E-101	100%	100%	239837254	Bacteria	n	n	uncultured bacterium
JQ998992	549	1	546	0	92%	92%	239913644	Bacteria	n	n	uncultured bacterium
JQ998389	431	17	91	1E-29	100%	100%	239923289	Bacteria	n	n	uncultured bacterium
JQ999174	577	23	577	0	90%	90%	240000256	Bacteria	n	n	uncultured bacterium
JQ998684	524	28	523	0	95%	95%	240000985	Bacteria	n	n	uncultured bacterium
JQ998691	525	4	525	0	93%	93%	240001010	Bacteria	n	n	uncultured bacterium
JQ999076	557	5	557	0	95%	95%	240001265	Bacteria	n	n	uncultured bacterium
JQ998251	382	7	229	1E-108	99%	99%	240001267	Bacteria	n	n	uncultured bacterium
JQ999055	555	6	548	0	95%	95%	240001303	Bacteria	n	n	uncultured bacterium
JQ998073	323	5	271	2E-131	99%	99%	240001638	Bacteria	n	n	uncultured bacterium
JQ998246	381	5	339	3E-159	97%	97%	240001755	Bacteria	n	n	uncultured bacterium
JQ998971	548	18	545	0	94%	94%	240001795	Bacteria	n	n	uncultured bacterium
JQ998923	545	5	541	3E-180	88%	88%	240001834	Bacteria	n	n	uncultured bacterium
JQ998940	546	5	539	0	96%	96%	240001842	Bacteria	n	n	uncultured bacterium
JQ998029	307	41	98	2E-20	100%	100%	240001855	Bacteria	n	n	uncultured bacterium
JQ998941	546	17	499	0	95%	95%	240002020	Bacteria	n	n	uncultured bacterium
JQ998432	448	9	412	2E-126	88%	88%	240002108	Bacteria	n	n	uncultured bacterium
JQ998279	392	15	360	2E-170	98%	98%	240126815	Bacteria	n	n	uncultured bacterium
JQ998590	502	18	435	0	99%	99%	240126870	Bacteria	n	n	uncultured bacterium
JQ998581	500	18	422	0	100%	100%	240126924	Bacteria	n	n	uncultured bacterium
JQ999094	558	24	549	0	98%	98%	240126929	Bacteria	n	n	uncultured bacterium
JQ999132	563	25	562	0	99%	99%	240126955	Bacteria	n	n	uncultured bacterium
JQ998300	399	5	233	2E-91	94%	94%	240127013	Bacteria	n	n	uncultured bacterium
JQ998339	411	11	197	3E-85	98%	98%	242247783	Bacteria	n	n	uncultured bacterium
JQ998214	371	17	327	3E-159	100%	100%	242758918	Bacteria	n	n	uncultured bacterium
JQ998384	429	4	304	1E-148	99%	99%	253769623	Bacteria	n	n	uncultured bacterium
JQ998730	530	20	527	0	94%	94%	253770307	Bacteria	n	n	uncultured bacterium
JQ998716	528	5	528	0	99%	99%	254210154	Bacteria	n	n	uncultured bacterium
JQ998415	441	18	380	2E-176	98%	98%	254210156	Bacteria	n	n	uncultured bacterium
JQ998606	506	8	506	0	94%	94%	254210165	Bacteria	n	n	uncultured bacterium
JQ998708	527	1	501	0	93%	93%	254210171	Bacteria	n	n	uncultured bacterium
JQ998786	535	18	535	0	97%	97%	254210176	Bacteria	n	n	uncultured bacterium
JQ998637	515	18	476	0	98%	98%	254210178	Bacteria	n	n	uncultured bacterium
JQ998423	445	5	374	1E-148	93%	93%	254210199	Bacteria	n	n	uncultured bacterium
JQ998634	514	5	512	0	99%	99%	254210200	Bacteria	n	n	uncultured bacterium

JQ997932	266	5	240	6E-110	97%	97%	254210211	Bacteria	n	n	uncultured bacterium
JQ997978	288	3	251	2E-115	98%	98%	254210213	Bacteria	n	n	uncultured bacterium
JQ998318	405	74	371	1E-133	96%	96%	254210221	Bacteria	n	n	uncultured bacterium
JQ998797	536	5	488	0	94%	94%	254210229	Bacteria	n	n	uncultured bacterium
JQ998837	539	8	526	0	97%	97%	254210230	Bacteria	n	n	uncultured bacterium
JQ998582	500	18	291	5E-133	99%	99%	254210233	Bacteria	n	n	uncultured bacterium
JQ998726	529	4	523	0	99%	99%	254210234	Bacteria	n	n	uncultured bacterium
JQ998756	533	2	530	0	99%	99%	254210245	Bacteria	n	n	uncultured bacterium
JQ998838	539	18	533	0	96%	96%	254210246	Bacteria	n	n	uncultured bacterium
JQ999010	551	18	547	0	98%	98%	254210252	Bacteria	n	n	uncultured bacterium
JQ998304	400	5	384	0	98%	98%	254210253	Bacteria	n	n	uncultured bacterium
JQ998685	524	5	487	0	97%	97%	254210256	Bacteria	n	n	uncultured bacterium
JQ999102	559	5	552	0	99%	99%	254210257	Bacteria	n	n	uncultured bacterium
JQ998955	547	5	540	0	97%	97%	254210258	Bacteria	n	n	uncultured bacterium
JQ998731	530	4	527	0	96%	96%	254210260	Bacteria	n	n	uncultured bacterium
JQ998180	361	5	329	8E-165	99%	99%	254210263	Bacteria	n	n	uncultured bacterium
JQ998216	372	5	323	1E-158	99%	99%	254210269	Bacteria	n	n	uncultured bacterium
JQ998566	494	5	436	0	98%	98%	254210270	Bacteria	n	n	uncultured bacterium
JQ998732	530	5	527	0	100%	100%	254210272	Bacteria	n	n	uncultured bacterium
JQ998823	538	15	532	0	94%	94%	254210274	Bacteria	n	n	uncultured bacterium
JQ998956	547	5	538	0	100%	100%	254210275	Bacteria	n	n	uncultured bacterium
JQ998717	528	17	486	0	99%	99%	254210279	Bacteria	n	n	uncultured bacterium
JQ998372	424	18	391	7E-161	94%	94%	254527898	Bacteria	n	n	uncultured bacterium
JQ997916	258	10	214	4E-77	93%	93%	254771243	Bacteria	n	n	uncultured bacterium
JQ998505	475	18	426	0	97%	97%	254771273	Bacteria	n	n	uncultured bacterium
JQ998677	523	5	485	0	98%	98%	254971490	Bacteria	n	n	uncultured bacterium
JQ998232	378	5	261	5E-122	98%	98%	255040912	Bacteria	n	n	uncultured bacterium
JQ998203	368	5	264	1E-132	100%	100%	255041408	Bacteria	n	n	uncultured bacterium
JQ998289	395	17	350	1E-173	100%	100%	255041571	Bacteria	n	n	uncultured bacterium
JQ998310	402	16	360	9E-180	100%	100%	255042010	Bacteria	n	n	uncultured bacterium
JQ998496	470	3	424	2E-166	93%	93%	255042296	Bacteria	n	n	uncultured bacterium
JQ998055	317	8	273	5E-136	100%	100%	255043198	Bacteria	n	n	uncultured bacterium
JQ998591	502	5	216	2E-82	93%	93%	255044557	Bacteria	n	n	uncultured bacterium
JQ998404	437	5	365	0	100%	100%	255045388	Bacteria	n	n	uncultured bacterium
JQ998116	340	5	287	2E-110	93%	93%	255045393	Bacteria	n	n	uncultured bacterium
JQ998638	515	5	439	0	97%	97%	255339762	Bacteria	n	n	uncultured bacterium
JQ999039	554	8	547	0	95%	95%	255339765	Bacteria	n	n	uncultured bacterium
JQ998599	504	17	463	2E-166	91%	91%	255689714	Bacteria	n	n	uncultured bacterium
JQ999021	552	3	552	0	99%	99%	255762955	Bacteria	n	n	uncultured bacterium
JQ998556	492	5	432	0	97%	97%	255976649	Bacteria	n	n	uncultured bacterium
JQ998237	379	18	326	7E-151	98%	98%	256352262	Bacteria	n	n	uncultured bacterium
JQ999028	553	5	553	0	95%	95%	256355274	Bacteria	n	n	uncultured bacterium
JQ998324	406	14	362	2E-180	100%	100%	256355277	Bacteria	n	n	uncultured bacterium
JQ998062	319	18	282	2E-106	94%	94%	256355286	Bacteria	n	n	uncultured bacterium
JQ999095	558	20	553	0	99%	99%	256592693	Bacteria	n	n	uncultured bacterium
JQ998381	428	26	373	3E-169	98%	98%	256592782	Bacteria	n	n	uncultured bacterium
JQ998068	322	9	77	5E-12	89%	89%	257130685	Bacteria	n	n	uncultured bacterium
JQ998164	356	15	309	1E-113	92%	92%	257130762	Bacteria	n	n	uncultured bacterium
JQ998010	300	5	260	4E-127	99%	99%	257131100	Bacteria	n	n	uncultured bacterium
JQ997885	241	37	199	1E-62	94%	94%	257131333	Bacteria	n	n	uncultured bacterium
JQ999212	681	5	157	2E-28	84%	84%	257131399	Bacteria	n	n	uncultured bacterium
JQ999077	557	17	548	0	99%	99%	257131579	Bacteria	n	n	uncultured bacterium
JQ998543	486	5	426	0	97%	97%	257131616	Bacteria	n	n	uncultured bacterium
JQ999056	555	18	512	0	94%	94%	257131817	Bacteria	n	n	uncultured bacterium
JQ998988	549	5	501	0	93%	93%	257132045	Bacteria	n	n	uncultured bacterium
JQ998942	546	17	500	0	95%	95%	257132142	Bacteria	n	n	uncultured bacterium
JQ999119	561	18	557	0	100%	100%	257132354	Bacteria	n	n	uncultured bacterium
JQ999189	601	1	70	2E-23	97%	97%	257132566	Bacteria	n	n	uncultured bacterium
JQ997879	234	34	199	7E-69	96%	96%	257143803	Bacteria	n	n	uncultured bacterium
JQ998411	439	5	383	0	99%	99%	257144096	Bacteria	n	n	uncultured bacterium

JQ998615	507	15	480	0	99%	99%	257144395	Bacteria	n	n	uncultured bacterium
JQ998607	506	18	419	0	100%	100%	257358096	Bacteria	n	n	uncultured bacterium
JQ999158	571	5	563	0	97%	97%	258547754	Bacteria	n	n	uncultured bacterium
JQ998520	479	18	433	0	99%	99%	258547879	Bacteria	n	n	uncultured bacterium
JQ999175	577	17	470	0	97%	97%	258547880	Bacteria	n	n	uncultured bacterium
JQ998798	536	19	531	0	90%	90%	258547923	Bacteria	n	n	uncultured bacterium
JQ998839	539	5	499	6E-158	88%	88%	258547969	Bacteria	n	n	uncultured bacterium
JQ998656	518	17	507	0	98%	98%	258547993	Bacteria	n	n	uncultured bacterium
JQ999111	560	18	555	0	99%	99%	258548105	Bacteria	n	n	uncultured bacterium
JQ998718	528	5	526	0	99%	99%	258548557	Bacteria	n	n	uncultured bacterium
JQ998628	511	17	480	0	100%	100%	258548759	Bacteria	n	n	uncultured bacterium
JQ998890	543	5	541	0	90%	90%	258548879	Bacteria	n	n	uncultured bacterium
JQ998247	381	3	335	2E-151	96%	96%	258550027	Bacteria	n	n	uncultured bacterium
JQ998924	545	5	543	0	100%	100%	258550101	Bacteria	n	n	uncultured bacterium
JQ998536	484	1	422	0	94%	94%	258550255	Bacteria	n	n	uncultured bacterium
JQ997972	284	18	240	1E-102	98%	98%	258550277	Bacteria	n	n	uncultured bacterium
JQ998030	307	5	254	2E-105	95%	95%	258550675	Bacteria	n	n	uncultured bacterium
JQ998058	318	13	268	2E-130	100%	100%	258551222	Bacteria	n	n	uncultured bacterium
JQ998644	516	15	515	0	93%	93%	259027961	Bacteria	n	n	uncultured bacterium
JQ998799	536	282	536	6E-123	98%	98%	259880027	Bacteria	n	n	uncultured bacterium
JQ999103	559	17	536	0	96%	96%	259880124	Bacteria	n	n	uncultured bacterium
JQ998269	388	5	353	5E-167	98%	98%	259880130	Bacteria	n	n	uncultured bacterium
JQ998824	538	25	535	0	97%	97%	259880134	Bacteria	n	n	uncultured bacterium
JQ999162	572	18	506	0	98%	98%	259880150	Bacteria	n	n	uncultured bacterium
JQ997912	255	18	219	8E-59	88%	88%	259880207	Bacteria	n	n	uncultured bacterium
JQ998399	434	18	386	0	98%	98%	259880216	Bacteria	n	n	uncultured bacterium
JQ999120	561	22	556	0	99%	99%	259880659	Bacteria	n	n	uncultured bacterium
JQ998698	526	65	496	0	94%	94%	260080805	Bacteria	n	n	uncultured bacterium
JQ998800	536	4	536	0	94%	94%	260103508	Bacteria	n	n	uncultured bacterium
JQ998787	535	18	529	0	98%	98%	260103512	Bacteria	n	n	uncultured bacterium
JQ998856	541	18	485	0	99%	99%	260103513	Bacteria	n	n	uncultured bacterium
JQ997875	219	5	161	5E-25	83%	83%	260103519	Bacteria	n	n	uncultured bacterium
JQ998487	467	43	402	7E-122	89%	89%	260103520	Bacteria	n	n	uncultured bacterium
JQ998567	494	5	411	0	99%	99%	260103525	Bacteria	n	n	uncultured bacterium
JQ998614	507	18	433	0	98%	98%	260103526	Bacteria	n	n	uncultured bacterium
JQ998989	549	19	498	0	95%	95%	260103537	Bacteria	n	n	uncultured bacterium
JQ998699	526	17	522	0	97%	97%	260451472	Bacteria	n	n	uncultured bacterium
JQ998757	533	18	529	0	100%	100%	260600086	Bacteria	n	n	uncultured bacterium
JQ998482	466	5	428	1E-158	91%	91%	260609733	Bacteria	n	n	uncultured bacterium
JQ998825	538	18	533	0	98%	98%	260609736	Bacteria	n	n	uncultured bacterium
JQ998891	543	17	540	0	93%	93%	260609741	Bacteria	n	n	uncultured bacterium
JQ998909	544	19	543	0	99%	99%	260609766	Bacteria	n	n	uncultured bacterium
JQ998752	532	18	530	0	98%	98%	260609798	Bacteria	n	n	uncultured bacterium
JQ997905	252	5	204	2E-99	100%	100%	260609860	Bacteria	n	n	uncultured bacterium
JQ998957	547	5	341	3E-170	99%	99%	260609871	Bacteria	n	n	uncultured bacterium
JQ998910	544	18	541	0	96%	96%	260609902	Bacteria	n	n	uncultured bacterium
JQ998233	378	5	331	2E-151	97%	97%	260609911	Bacteria	n	n	uncultured bacterium
JQ998211	370	4	335	4E-108	89%	89%	260609943	Bacteria	n	n	uncultured bacterium
JQ999142	566	19	541	0	94%	94%	260610053	Bacteria	n	n	uncultured bacterium
JQ999011	551	5	546	0	98%	98%	260610111	Bacteria	n	n	uncultured bacterium
JQ998280	392	18	151	2E-62	100%	100%	260610172	Bacteria	n	n	uncultured bacterium
JQ998911	544	15	542	0	97%	97%	260610180	Bacteria	n	n	uncultured bacterium
JQ998059	318	18	272	7E-130	100%	100%	260610209	Bacteria	n	n	uncultured bacterium
JQ998532	483	18	304	2E-147	100%	100%	260610223	Bacteria	n	n	uncultured bacterium
JQ999159	571	17	415	4E-150	92%	92%	260610275	Bacteria	n	n	uncultured bacterium
JQ998871	542	17	540	0	98%	98%	260610340	Bacteria	n	n	uncultured bacterium
JQ998354	416	24	364	3E-169	99%	99%	261261807	Bacteria	n	n	uncultured bacterium
JQ998812	537	17	533	0	100%	100%	261261894	Bacteria	n	n	uncultured bacterium
JQ998592	502	18	438	0	99%	99%	261262014	Bacteria	n	n	uncultured bacterium
JQ999143	566	23	531	0	100%	100%	261262551	Bacteria	n	n	uncultured bacterium

JQ999150	568	5	488	0	100%	100%	261262602	Bacteria	n	n	uncultured bacterium
JQ999096	558	24	540	0	97%	97%	261262638	Bacteria	n	n	uncultured bacterium
JQ998753	532	18	532	0	99%	99%	261262880	Bacteria	n	n	uncultured bacterium
JQ998624	510	39	452	0	96%	96%	261262931	Bacteria	n	n	uncultured bacterium
JQ998521	479	18	434	0	100%	100%	261262996	Bacteria	n	n	uncultured bacterium
JQ999012	551	4	547	0	91%	91%	261263024	Bacteria	n	n	uncultured bacterium
JQ998501	474	5	408	0	100%	100%	261499577	Bacteria	n	n	uncultured bacterium
JQ998461	458	20	406	2E-161	94%	94%	261748993	Bacteria	n	n	uncultured bacterium
JQ999204	639	5	286	4E-105	92%	92%	262213280	Bacteria	n	n	uncultured bacterium
JQ998150	351	70	322	3E-104	94%	94%	262344293	Bacteria	n	n	uncultured bacterium
JQ998126	343	18	301	6E-141	99%	99%	262399014	Bacteria	n	n	uncultured bacterium
JQ998067	321	17	277	2E-131	100%	100%	264666385	Bacteria	n	n	uncultured bacterium
JQ998416	441	16	49	0.0000007	100%	100%	269175391	Bacteria	n	n	uncultured bacterium
JQ999136	564	5	561	0	98%	98%	269855868	Bacteria	n	n	uncultured bacterium
JQ998508	476	3	322	7E-127	93%	93%	269971070	Bacteria	n	n	uncultured bacterium
JQ998530	482	5	432	2E-131	88%	88%	269971816	Bacteria	n	n	uncultured bacterium
JQ998047	315	5	245	1E-82	91%	91%	270094636	Bacteria	n	n	uncultured bacterium
JQ998497	472	18	87	1E-25	99%	99%	270097664	Bacteria	n	n	uncultured bacterium
JQ998622	509	18	464	0	99%	99%	274138243	Bacteria	n	n	uncultured bacterium
JQ998639	515	3	478	7E-132	86%	86%	281187385	Bacteria	n	n	uncultured bacterium
JQ998872	542	20	57	0.0000003	97%	97%	281187515	Bacteria	n	n	uncultured bacterium
JQ998011	300	21	251	3E-113	99%	99%	281308637	Bacteria	n	n	uncultured bacterium
JQ999104	559	4	559	0	88%	88%	281333182	Bacteria	n	n	uncultured bacterium
JQ998733	530	5	528	0	98%	98%	281413467	Bacteria	n	n	uncultured bacterium
JQ999078	557	16	549	0	93%	93%	281484434	Bacteria	n	n	uncultured bacterium
JQ998194	365	52	263	5E-97	98%	98%	281487069	Bacteria	n	n	uncultured bacterium
JQ998215	371	5	298	1E-87	88%	88%	281487151	Bacteria	n	n	uncultured bacterium
JQ998456	456	4	375	1E-133	91%	91%	281487241	Bacteria	n	n	uncultured bacterium
JQ998744	531	18	528	0	93%	93%	281488370	Bacteria	n	n	uncultured bacterium
JQ998091	328	18	125	8E-45	98%	98%	281488374	Bacteria	n	n	uncultured bacterium
JQ997921	262	5	210	2E-99	99%	99%	281488574	Bacteria	n	n	uncultured bacterium
JQ998488	467	3	69	2E-22	97%	97%	281489388	Bacteria	n	n	uncultured bacterium
JQ998294	397	2	341	1E-173	99%	99%	281489463	Bacteria	n	n	uncultured bacterium
JQ998003	298	5	106	1E-23	89%	89%	283130988	Bacteria	n	n	uncultured bacterium
JQ999079	557	18	542	0	94%	94%	283765019	Bacteria	n	n	uncultured bacterium
JQ998734	530	5	481	2E-173	90%	90%	283765052	Bacteria	n	n	uncultured bacterium
JQ998678	523	18	480	0	100%	100%	283776532	Bacteria	n	n	uncultured bacterium
JQ999069	556	24	554	0	97%	97%	284025684	Bacteria	n	n	uncultured bacterium
JQ998840	539	15	374	3E-180	99%	99%	284025703	Bacteria	n	n	uncultured bacterium
JQ999080	557	25	516	0	95%	95%	284158321	Bacteria	n	n	uncultured bacterium
JQ998331	408	5	369	3E-169	96%	96%	284158446	Bacteria	n	n	uncultured bacterium
JQ997988	293	18	251	1E-111	98%	98%	284158467	Bacteria	n	n	uncultured bacterium
JQ998958	547	21	546	0	99%	99%	284158493	Bacteria	n	n	uncultured bacterium
JQ998990	549	17	543	0	99%	99%	284158495	Bacteria	n	n	uncultured bacterium
JQ998220	374	5	302	1E-143	98%	98%	284466811	Bacteria	n	n	uncultured bacterium
JQ998242	380	5	301	5E-147	99%	99%	284944606	Bacteria	n	n	uncultured bacterium
JQ998777	534	18	500	0	93%	93%	285016444	Bacteria	n	n	uncultured bacterium
JQ999191	603	1	599	0	90%	90%	285960213	Bacteria	n	n	uncultured bacterium
JQ998365	421	17	354	2E-172	99%	99%	285960215	Bacteria	n	n	uncultured bacterium
JQ998672	522	5	516	0	96%	96%	285960268	Bacteria	n	n	uncultured bacterium
JQ999133	563	9	558	0	97%	97%	285960443	Bacteria	n	n	uncultured bacterium
JQ998912	544	8	388	0	99%	99%	285960644	Bacteria	n	n	uncultured bacterium
JQ998287	394	5	102	6E-37	97%	97%	285960840	Bacteria	n	n	uncultured bacterium
JQ998511	477	5	430	2E-156	91%	91%	285960899	Bacteria	n	n	uncultured bacterium
JQ998325	406	18	357	5E-177	100%	100%	288551167	Bacteria	n	n	uncultured bacterium
JQ999013	551	5	546	0	99%	99%	288551183	Bacteria	n	n	uncultured bacterium
JQ998700	526	5	221	2E-108	100%	100%	289185876	Bacteria	n	n	uncultured bacterium
JQ998083	326	19	262	4E-122	100%	100%	289186586	Bacteria	n	n	uncultured bacterium
JQ998122	342	17	287	1E-138	100%	100%	289429664	Bacteria	n	n	uncultured bacterium
JQ998333	409	21	359	4E-128	92%	92%	289576451	Bacteria	n	n	uncultured bacterium

JQ998270	389	5	331	8E-165	99%	99%	289594430	Bacteria	n	n	uncultured bacterium
JQ999081	557	5	557	0	93%	93%	289594438	Bacteria	n	n	uncultured bacterium
JQ998972	548	7	473	6E-108	82%	82%	290564637	Bacteria	n	n	uncultured bacterium
JQ998529	482	18	414	0	99%	99%	290586931	Bacteria	n	n	uncultured bacterium
JQ998424	445	10	381	4E-134	90%	90%	290599218	Bacteria	n	n	uncultured bacterium
JQ998409	438	111	145	0.0000002	100%	100%	290603331	Bacteria	n	n	uncultured bacterium
JQ998281	392	5	320	6E-87	87%	87%	290607302	Bacteria	n	n	uncultured bacterium
JQ999198	615	136	312	6E-44	87%	87%	290608049	Bacteria	n	n	uncultured bacterium
JQ997908	254	14	207	5E-96	100%	100%	290609113	Bacteria	n	n	uncultured bacterium
JQ998745	531	40	483	1E-179	93%	93%	290610030	Bacteria	n	n	uncultured bacterium
JQ998925	545	41	447	0	95%	95%	290610134	Bacteria	n	n	uncultured bacterium
JQ999221	961	5	45	2E-10	100%	100%	290615238	Bacteria	n	n	uncultured bacterium
JQ998686	524	69	475	0	96%	96%	290615266	Bacteria	n	n	uncultured bacterium
JQ998857	541	34	502	0	96%	96%	290616525	Bacteria	n	n	uncultured bacterium
JQ998537	484	3	430	0	100%	100%	290616865	Bacteria	n	n	uncultured bacterium
JQ998099	333	144	301	6E-71	98%	98%	290617111	Bacteria	n	n	uncultured bacterium
JQ998788	535	229	508	1E-140	99%	99%	290617937	Bacteria	n	n	uncultured bacterium
JQ999040	554	19	454	3E-175	92%	92%	290618217	Bacteria	n	n	uncultured bacterium
JQ998297	398	16	322	5E-127	94%	94%	290619377	Bacteria	n	n	uncultured bacterium
JQ998926	545	24	500	0	93%	93%	290619838	Bacteria	n	n	uncultured bacterium
JQ999029	553	29	514	0	94%	94%	290619849	Bacteria	n	n	uncultured bacterium
JQ998506	475	18	404	0	99%	99%	290619868	Bacteria	n	n	uncultured bacterium
JQ998151	351	5	296	1E-123	95%	95%	290619952	Bacteria	n	n	uncultured bacterium
JQ998578	497	11	440	1E-129	87%	87%	290620581	Bacteria	n	n	uncultured bacterium
JQ998169	358	24	293	4E-123	97%	97%	290621172	Bacteria	n	n	uncultured bacterium
JQ998657	518	109	457	7E-152	95%	95%	290621918	Bacteria	n	n	uncultured bacterium
JQ997973	284	18	240	4E-97	96%	96%	290621930	Bacteria	n	n	uncultured bacterium
JQ998170	358	5	274	4E-128	98%	98%	290624086	Bacteria	n	n	uncultured bacterium
JQ998254	383	20	339	7E-151	97%	97%	290624805	Bacteria	n	n	uncultured bacterium
JQ999220	901	18	92	2E-25	97%	97%	290626899	Bacteria	n	n	uncultured bacterium
JQ997969	283	18	181	9E-64	94%	94%	290628890	Bacteria	n	n	uncultured bacterium
JQ999170	574	17	335	2E-158	99%	99%	290629721	Bacteria	n	n	uncultured bacterium
JQ997894	246	18	214	1E-87	97%	97%	290770329	Bacteria	n	n	uncultured bacterium
JQ999205	655	1	68	6E-24	99%	99%	291060461	Bacteria	n	n	uncultured bacterium
JQ998171	358	18	243	2E-100	96%	96%	291060512	Bacteria	n	n	uncultured bacterium
JQ998692	525	17	482	0	100%	100%	291192718	Bacteria	n	n	uncultured bacterium
JQ997955	275	5	243	3E-88	92%	92%	291192723	Bacteria	n	n	uncultured bacterium
JQ999144	566	5	564	0	97%	97%	291192725	Bacteria	n	n	uncultured bacterium
JQ998927	545	7	542	0	99%	99%	291192726	Bacteria	n	n	uncultured bacterium
JQ998457	456	18	411	0	97%	97%	291192727	Bacteria	n	n	uncultured bacterium
JQ998758	533	18	497	0	94%	94%	291192731	Bacteria	n	n	uncultured bacterium
JQ998991	549	17	541	0	97%	97%	291192733	Bacteria	n	n	uncultured bacterium
JQ998813	537	18	531	0	99%	99%	291192736	Bacteria	n	n	uncultured bacterium
JQ999151	568	17	550	0	94%	94%	291192737	Bacteria	n	n	uncultured bacterium
JQ998759	533	17	522	0	98%	98%	291192739	Bacteria	n	n	uncultured bacterium
JQ999041	554	8	545	0	94%	94%	291192741	Bacteria	n	n	uncultured bacterium
JQ998332	408	9	361	2E-132	91%	91%	291192751	Bacteria	n	n	uncultured bacterium
JQ998402	436	5	405	3E-179	95%	95%	291247631	Bacteria	n	n	uncultured bacterium
JQ998117	340	18	298	1E-142	100%	100%	291251358	Bacteria	n	n	uncultured bacterium
JQ998640	515	5	179	4E-85	100%	100%	291277719	Bacteria	n	n	uncultured bacterium
JQ998544	486	5	132	4E-59	100%	100%	291277728	Bacteria	n	n	uncultured bacterium
JQ997952	274	2	229	1E-111	99%	99%	291507687	Bacteria	n	n	uncultured bacterium
JQ998600	504	17	298	4E-139	99%	99%	291507688	Bacteria	n	n	uncultured bacterium
JQ998161	353	4	311	2E-150	98%	98%	294478352	Bacteria	n	n	uncultured bacterium
JQ999188	597	17	593	0	92%	92%	294478540	Bacteria	n	n	uncultured bacterium
JQ997922	262	5	218	2E-70	90%	90%	294478555	Bacteria	n	n	uncultured bacterium
JQ999215	700	16	265	5E-35	80%	80%	294478594	Bacteria	n	n	uncultured bacterium
JQ998189	364	5	169	4E-63	94%	94%	294478603	Bacteria	n	n	uncultured bacterium
JQ998156	352	17	301	1E-128	96%	96%	294478693	Bacteria	n	n	uncultured bacterium
JQ997920	261	5	160	4E-67	97%	97%	294514723	Bacteria	n	n	uncultured bacterium

JQ998525	480	5	423	0	100%	100%	294514757	Bacteria	n	n	uncultured bacterium
JQ998760	533	28	421	4E-144	91%	91%	294652611	Bacteria	n	n	uncultured bacterium
JQ998512	477	10	370	3E-120	90%	90%	294663720	Bacteria	n	n	uncultured bacterium
JQ998343	412	25	367	5E-177	100%	100%	294719638	Bacteria	n	n	uncultured bacterium
JQ998186	363	5	289	1E-133	98%	98%	294998181	Bacteria	n	n	uncultured bacterium
JQ997902	250	5	147	1E-67	100%	100%	295005427	Bacteria	n	n	uncultured bacterium
JQ997895	246	18	102	4E-27	94%	94%	295027798	Bacteria	n	n	uncultured bacterium
JQ997941	269	19	223	4E-82	94%	94%	295029932	Bacteria	n	n	uncultured bacterium
JQ997948	272	3	220	5E-106	99%	99%	295322327	Bacteria	n	n	uncultured bacterium
JQ998125	343	5	299	8E-150	100%	100%	295394073	Bacteria	n	n	uncultured bacterium
JQ998735	530	22	487	0	93%	93%	295809979	Bacteria	n	n	uncultured bacterium
JQ998049	315	5	283	2E-96	90%	90%	295810133	Bacteria	n	n	uncultured bacterium
JQ998557	492	5	423	0	100%	100%	295810494	Bacteria	n	n	uncultured bacterium
JQ998192	364	5	300	6E-136	97%	97%	295810605	Bacteria	n	n	uncultured bacterium
JQ998568	494	5	437	0	100%	100%	295814828	Bacteria	n	n	uncultured bacterium
JQ999500	399	49	354	4E-153	99%	99%	62997524	Bacteria	n	n	uncultured bacterium
JQ999496	429	5	392	1E-158	93%	93%	219962362	Bacteria	n	n	uncultured bacterium
JQ999495	370	5	337	2E-151	96%	96%	223030862	Bacteria	n	n	uncultured bacterium
JQ999498	426	18	394	7E-176	97%	97%	223033601	Bacteria	n	n	uncultured bacterium
JQ999499	521	27	473	0	96%	96%	223034843	Bacteria	n	n	uncultured bacterium
JQ999497	564	18	527	0	98%	98%	226903461	Bacteria	n	n	uncultured bacterium
JQ998382	207	1	194	8E-94	99%	99%	219529249	Bacteria	n	n	uncultured bacterium
JQ998439	242	1	242	1E-112	98%	98%	225337346	Bacteria	n	n	uncultured bacterium
JQ998474	236	1	236	1E-107	97%	97%	322226064	Bacteria	n	n	uncultured bacterium
JQ998513	231	1	218	1E-101	98%	98%	169288388	Bacteria	n	n	uncultured bacterium
JQ998789	224	1	224	5E-111	99%	99%	377550288	Bacteria	n	n	uncultured bacterium
JQ998981	268	1	262	4E-108	95%	95%	322177659	Bacteria	n	n	uncultured bacterium
JQ999169	428	1	428	3E-160	91%	91%	322206429	Bacteria	n	n	uncultured bacterium
JQ999171	243	1	240	1E-57	86%	86%	325960808	Bacteria	n	n	uncultured bacterium
JQ999202	246	1	246	3E-98	94%	94%	296951465	Bacteria	n	n	uncultured bacterium
JQ999222	362	18	317	2E-151	99%	99%	105990434	Bacteria	n	n	uncultured candidate division WYO bacterium
JQ999223	446	24	381	3E-135	91%	91%	20975333	Bacteria	n	n	uncultured chicken cecal bacterium
JQ999224	332	24	287	7E-135	100%	100%	295018012	Bacteria	n	n	uncultured compost bacterium
JQ999231	856	5	90	2E-20	90%	90%	295018042	Bacteria	n	n	uncultured compost bacterium
JQ999225	471	20	420	0	100%	100%	295018051	Bacteria	n	n	uncultured compost bacterium
JQ999227	531	5	529	0	98%	98%	295018127	Bacteria	n	n	uncultured compost bacterium
JQ999230	541	155	530	0	98%	98%	295018129	Bacteria	n	n	uncultured compost bacterium
JQ999229	539	5	537	0	100%	100%	295018133	Bacteria	n	n	uncultured compost bacterium
JQ999228	535	18	517	0	99%	99%	295026878	Bacteria	n	n	uncultured compost bacterium
JQ999226	511	17	442	0	100%	100%	295027011	Bacteria	n	n	uncultured compost bacterium
JQ999233	528	18	513	0	96%	96%	34525928	Bacteria	n	n	uncultured Gram-positive bacterium
JQ999234	540	110	513	2E-143	90%	90%	56541536	Bacteria	n	n	uncultured Gram-positive bacterium
JQ999232	501	7	434	1E-159	91%	91%	253721751	Bacteria	n	n	uncultured Gram-positive bacterium
JQ999235	247	5	212	3E-102	100%	100%	270305386	Bacteria	n	n	uncultured marine bacterium
JQ999239	460	4	381	0	99%	99%	284467687	Bacteria	n	n	uncultured marine bacterium
JQ999236	300	16	268	4E-127	100%	100%	284467695	Bacteria	n	n	uncultured marine bacterium
JQ999237	398	17	346	9E-170	100%	100%	284467703	Bacteria	n	n	uncultured marine bacterium
JQ999238	432	5	385	0	99%	99%	284468021	Bacteria	n	n	uncultured marine bacterium
JQ999240	421	18	329	6E-152	98%	98%	150022096	Bacteria	n	n	uncultured rumen bacterium
JQ999242	484	26	169	4E-64	99%	99%	283981089	Bacteria	n	n	uncultured rumen bacterium
JQ999241	469	22	437	6E-177	94%	94%	283982045	Bacteria	n	n	uncultured rumen bacterium
JQ999245	382	24	332	4E-158	100%	100%	16517886	Bacteria	n	n	uncultured soil bacterium
JQ999247	474	16	402	7E-117	88%	88%	81022835	Bacteria	n	n	uncultured soil bacterium
JQ999250	543	5	538	0	92%	92%	83285238	Bacteria	n	n	uncultured soil bacterium
JQ999249	541	5	541	0	96%	96%	87243108	Bacteria	n	n	uncultured soil bacterium
JQ999244	331	4	289	7E-135	97%	97%	109391696	Bacteria	n	n	uncultured soil bacterium
JQ999246	452	18	412	0	100%	100%	194475432	Bacteria	n	n	uncultured soil bacterium
JQ999248	504	5	454	0	96%	96%	194475447	Bacteria	n	n	uncultured soil bacterium
JQ999252	555	1	326	7E-167	100%	100%	194475452	Bacteria	n	n	uncultured soil bacterium
JQ999243	329	17	293	4E-132	98%	98%	239737157	Bacteria	n	n	uncultured soil bacterium

JQ999251	554	18	535	0	99%	99%	260750894	Bacteria	n	n	uncultured soil bacterium
JQ999253	324	15	107	5E-37	98%	98%	6018249	Bacteria	n	n	uncultured sponge symbiont PAUC32f
JQ999254	352	95	315	7E-61	87%	87%	6453690	Bacteria	n	n	unidentified eubacterium clone BSV70
JQ999255	535	4	516	0	91%	91%	218533752	Bacteria	Planctomycetes	n	uncultured planctomycete
JQ999256	543	18	493	0	100%	100%	224027504	Bacteria	Proteobacteria (alpha)	Caulobacteraceae	Brevundimonas sp. AKB-2008-JO46
JQ999257	532	5	424	0	98%	98%	239056019	Bacteria	Proteobacteria (alpha)	Caulobacteraceae	Brevundimonas sp. MCS 35
JQ999258	525	19	471	0	99%	99%	295809779	Bacteria	Proteobacteria (alpha)	Caulobacteraceae	Brevundimonas sp. V3M6
JQ999259	367	16	329	5E-152	98%	98%	288908581	Bacteria	Proteobacteria (alpha)	Caulobacteraceae	Caulobacter sp. cau1
JQ999260	283	5	164	9E-69	97%	97%	254933869	Bacteria	Proteobacteria (alpha)	n	alpha proteobacterium EX129
JQ999261	555	16	550	0	99%	99%	197360272	Bacteria	Proteobacteria (alpha)	n	uncultured alpha proteobacterium
JQ998943	546	5	545	0	95%	95%	237973923	Bacteria	Proteobacteria (alpha)	n	uncultured bacterium
JQ999262	548	20	529	0	96%	96%	285026366	Bacteria	Proteobacteria (alpha)	Hyphomicrobiaceae	Hyphomicrobium vulgare
JQ999263	564	17	554	0	97%	97%	73622359	Bacteria	Proteobacteria (alpha)	Methylobacteriaceae	Methylobacterium sp. iRIV1
JQ999264	275	5	243	1E-117	99%	99%	186923312	Bacteria	Proteobacteria (alpha)	Methylobacteriaceae	uncultured Methylobacterium sp.
JQ999265	550	18	548	0	99%	99%	242963821	Bacteria	Proteobacteria (alpha)	Rhizobiaceae	uncultured Agrobacterium sp.
JQ999266	548	5	543	0	90%	90%	164598042	Bacteria	Proteobacteria (alpha)	Rhizobiaceae	uncultured Rhizobium sp.
JQ999267	538	24	331	7E-147	98%	98%	295651547	Bacteria	Proteobacteria (alpha)	Rhodobacteraceae	Paracoccus sp. HMD3141
JQ999268	313	10	267	4E-117	97%	97%	38425236	Bacteria	Proteobacteria (alpha)	Rhodobacteraceae	Paracoccus sp. J364
JQ999269	571	18	527	0	98%	98%	194395238	Bacteria	Proteobacteria (alpha)	Rhodobacteraceae	Paracoccus sp. JLT1284
JQ999270	529	16	528	0	100%	100%	237638489	Bacteria	Proteobacteria (alpha)	Rhodobacteraceae	Paracoccus sp. MC5-8
JQ999271	293	5	237	2E-115	100%	100%	294662650	Bacteria	Proteobacteria (alpha)	Rhodobacteraceae	Paracoccus sp. PS31_2010_
JQ999272	341	30	293	8E-135	100%	100%	289064903	Bacteria	Proteobacteria (alpha)	Rhodobacteraceae	Paracoccus sp. sptzw33
JQ999273	556	3	302	2E-138	97%	97%	139001937	Bacteria	Proteobacteria (alpha)	Rhodobacteraceae	Paracoccus sp. SSRW9-1
JQ999274	334	18	190	3E-79	98%	98%	158392748	Bacteria	Proteobacteria (alpha)	Rhodobacteraceae	Paracoccus sp. YT0095
JQ999275	386	18	353	1E-158	97%	97%	56266597	Bacteria	Proteobacteria (alpha)	Rhodobacteraceae	Paracoccus versutus
JQ999276	503	18	457	0	100%	100%	206581410	Bacteria	Proteobacteria (alpha)	Rhodobacteraceae	Paracoccus yeei
JQ999277	519	13	469	0	100%	100%	125656032	Bacteria	Proteobacteria (alpha)	Rhodobacteraceae	Rhodobacter changlensis
JQ999278	531	18	529	0	96%	96%	282934988	Bacteria	Proteobacteria (alpha)	Rhodobacteraceae	Rhodobacter sp. RC5-103
JQ999279	558	17	410	0	99%	99%	71844062	Bacteria	Proteobacteria (alpha)	Rhodobacteraceae	uncultured Amaricoccus sp.
JQ999280	558	16	530	0	93%	93%	50364319	Bacteria	Proteobacteria (alpha)	Rhodobacteraceae	uncultured Sulfitobacter sp.
JQ999281	352	9	286	1E-103	92%	92%	197359977	Bacteria	Proteobacteria (alpha)	Erythrobacteraceae	uncultured Porphyrobacter sp.
JQ999282	519	5	474	0	100%	100%	197114179	Bacteria	Proteobacteria (alpha)	Sphingomonadaceae	Sphingomonas dokdonensis
JQ999283	271	5	238	5E-116	100%	100%	294992056	Bacteria	Proteobacteria (alpha)	Sphingomonadaceae	Sphingomonas sp. MJ528
JQ999284	388	21	352	2E-135	94%	94%	284434472	Bacteria	Proteobacteria (alpha)	Sphingomonadaceae	Sphingomonas sp. NMC17
JQ999285	574	18	566	0	96%	96%	158935501	Bacteria	Proteobacteria (alpha)	Sphingomonadaceae	Sphingomonas sp. PA218
JQ999286	591	118	263	3E-26	84%	84%	186925070	Bacteria	Proteobacteria (alpha)	Sphingomonadaceae	uncultured Sphingomonadaceae bacterium
JQ999287	342	18	295	1E-132	98%	98%	192803975	Bacteria	Proteobacteria (alpha)	Sphingomonadaceae	uncultured Sphingomonas sp.
JQ999288	499	24	423	0	98%	98%	209420935	Bacteria	Proteobacteria (alpha)	Sphingomonadaceae	uncultured Sphingomonas sp.
JQ999290	552	3	453	3E-161	90%	90%	209421510	Bacteria	Proteobacteria (alpha)	Sphingomonadaceae	uncultured Sphingomonas sp.
JQ999289	531	136	525	0	99%	99%	209423311	Bacteria	Proteobacteria (alpha)	Sphingomonadaceae	uncultured Sphingomonas sp.
JQ999291	411	5	246	2E-111	98%	98%	44194288	Bacteria	Proteobacteria (beta)	Burkholderiaceae	Burkholderia sp.
JQ999292	562	18	558	0	97%	97%	118139431	Bacteria	Proteobacteria (beta)	Burkholderiaceae	Burkholderia sp. Brij35
JQ999293	556	5	357	1E-104	88%	88%	246771317	Bacteria	Proteobacteria (beta)	Burkholderiaceae	Burkholderia sp. LD-11
JQ999296	465	4	295	3E-145	99%	99%	254972620	Bacteria	Proteobacteria (beta)	Burkholderiaceae	uncultured Burkholderia sp.
JQ999295	430	5	190	1E-89	99%	99%	295656441	Bacteria	Proteobacteria (beta)	Burkholderiaceae	uncultured Burkholderia sp.
JQ999297	542	18	475	9E-161	91%	91%	168812099	Bacteria	Proteobacteria (beta)	Comamonadaceae	Acidovorax defluvii
JQ999298	456	1	261	2E-131	100%	100%	109659435	Bacteria	Proteobacteria (beta)	Comamonadaceae	Caldimonas hydrothermale
JQ999299	539	5	534	0	100%	100%	189047087	Bacteria	Proteobacteria (beta)	Comamonadaceae	Caldimonas manganoxidans
JQ999300	538	18	535	0	99%	99%	290350907	Bacteria	Proteobacteria (beta)	Comamonadaceae	Comamonadaceae bacterium Gu-R-8
JQ999302	532	18	532	0	98%	98%	5007060	Bacteria	Proteobacteria (beta)	Comamonadaceae	Delftia acidovorans
JQ999301	264	5	219	5E-91	95%	95%	15529695	Bacteria	Proteobacteria (beta)	Comamonadaceae	Delftia acidovorans
JQ999303	569	17	568	0	100%	100%	213536827	Bacteria	Proteobacteria (beta)	Comamonadaceae	Delftia acidovorans
JQ999304	345	69	301	6E-111	98%	98%	282892628	Bacteria	Proteobacteria (beta)	Comamonadaceae	Diaphorobacter sp. DNB7
JQ999305	252	4	206	4E-101	100%	100%	294828897	Bacteria	Proteobacteria (beta)	Comamonadaceae	uncultured Polaromonas sp.
JQ999306	502	15	468	0	96%	96%	153869451	Bacteria	Proteobacteria (beta)	Oxalobacteraceae	Herbaspirillum huttiense
JQ999307	412	25	366	2E-176	100%	100%	62183809	Bacteria	Proteobacteria (beta)	Oxalobacteraceae	Herbaspirillum sp. B601
JQ999308	546	117	521	1E-159	92%	92%	121488021	Bacteria	Proteobacteria (beta)	Sutterellaceae	Sutterella morbirenis
JQ999309	551	6	548	0	98%	98%	215981578	Bacteria	Proteobacteria (beta)	Sutterellaceae	uncultured Sutterella sp.
JQ999310	443	18	373	0	99%	99%	295656273	Bacteria	Proteobacteria (beta)	n	beta proteobacterium enrichment culture clone VNAB098
JQ999311	527	15	522	0	99%	99%	85002019	Bacteria	Proteobacteria (beta)	n	Denitrobacter sp. BBTR53

JQ999313	540	19	534	0	100%	100%	154192572	Bacteria	Proteobacteria (beta)	n	uncultured beta proteobacterium
JQ999312	426	5	367	6E-177	98%	98%	219880888	Bacteria	Proteobacteria (beta)	n	uncultured beta proteobacterium
JQ999314	526	18	524	0	99%	99%	290759918	Bacteria	Proteobacteria (beta)	Neisseriaceae	Neisseria flava
JQ999315	544	3	544	0	96%	96%	60500789	Bacteria	Proteobacteria (beta)	Neisseriaceae	uncultured Neisseria sp.
JQ999316	547	22	547	0	99%	99%	238915008	Bacteria	Proteobacteria (beta)	Neisseriaceae	uncultured Neisseria sp.
JQ999317	535	18	529	0	90%	90%	223036385	Bacteria	Proteobacteria (beta)	Nitrosomonadaceae	uncultured Nitrosomonas sp.
JQ999494	222	1	222	8E-104	98%	98%	34604519	Bacteria	Proteobacteria (delta)	Bacteriovoracaceae	Bacteriovorax
JQ999318	550	5	508	0	94%	94%	34604519	Bacteria	Proteobacteria (delta)	Bacteriovoracaceae	Bacteriovorax sp. EPC3
JQ999319	464	18	431	1E-144	89%	89%	284428338	Bacteria	Proteobacteria (delta)	Pelobacteraceae	uncultured Pelobacter sp.
JQ999320	556	18	548	0	98%	98%	61201821	Bacteria	Proteobacteria (delta)	n	uncultured Myxococcales bacterium
JQ998693	525	18	502	0	95%	95%	237948946	Bacteria	Proteobacteria (delta)	n	uncultured bacterium
JQ999321	647	4	333	5E-154	97%	97%	290759912	Bacteria	Proteobacteria (epsilon)	Campylobacteraceae	Campylobacter concisus
JQ999322	336	4	287	2E-131	97%	97%	41400321	Bacteria	Proteobacteria (epsilon)	Helicobacteraceae	uncultured Helicobacter sp.
JQ999324	394	24	346	2E-112	90%	90%	254772196	Bacteria	Proteobacteria (gamma)	Alteromonadaceae	Aestuariibacter sp. PaD1.07
JQ999325	334	8	261	2E-106	95%	95%	219846182	Bacteria	Proteobacteria (gamma)	Alteromonadaceae	Microbulbifer maritimus
JQ999326	463	18	390	0	98%	98%	239775314	Bacteria	Proteobacteria (gamma)	Pseudoalteromonadaceae	Pseudoalteromonas sp. Ld19
JQ999328	423	5	349	2E-147	95%	95%	257123155	Bacteria	Proteobacteria (gamma)	Chromatiaceae	Rheinheimera sp. HMD2012
JQ999329	244	18	202	4E-91	100%	100%	220936495	Bacteria	Proteobacteria (gamma)	Enterobacteriaceae	Klebsiella sp. C611
JQ999330	278	5	225	5E-111	100%	100%	33334429	Bacteria	Proteobacteria (gamma)	Enterobacteriaceae	primary endosymbiont of Sitophilus zeamais
JQ999331	244	6	182	3E-82	98%	98%	215981570	Bacteria	Proteobacteria (gamma)	Enterobacteriaceae	uncultured Shigella sp.
JQ999332	612	306	601	2E-84	87%	87%	194368434	Bacteria	Proteobacteria (gamma)	n	gamma proteobacterium B-WPhS5
JQ999333	597	12	61	1E-15	100%	100%	238623540	Bacteria	Proteobacteria (gamma)	n	gamma proteobacterium C48 UNDR-2009
JQ999334	386	5	258	3E-100	93%	93%	291195498	Bacteria	Proteobacteria (gamma)	n	gamma proteobacterium enrichment culture clone BF35-3
JQ999335	354	18	271	1E-122	98%	98%	291195511	Bacteria	Proteobacteria (gamma)	n	gamma proteobacterium enrichment culture clone JF9-3
JQ999343	510	18	463	0	100%	100%	22135588	Bacteria	Proteobacteria (gamma)	n	uncultured gamma proteobacterium
JQ999341	505	14	446	3E-165	91%	91%	90995220	Bacteria	Proteobacteria (gamma)	n	uncultured gamma proteobacterium
JQ999342	507	18	449	0	95%	95%	148615128	Bacteria	Proteobacteria (gamma)	n	uncultured gamma proteobacterium
JQ999344	524	5	521	0	96%	96%	148723984	Bacteria	Proteobacteria (gamma)	n	uncultured gamma proteobacterium
JQ999346	569	18	565	0	98%	98%	152003521	Bacteria	Proteobacteria (gamma)	n	uncultured gamma proteobacterium
JQ999340	478	18	425	5E-168	93%	93%	218533821	Bacteria	Proteobacteria (gamma)	n	uncultured gamma proteobacterium
JQ999347	721	1	65	3E-22	98%	98%	225031789	Bacteria	Proteobacteria (gamma)	n	uncultured gamma proteobacterium
JQ999337	320	5	276	6E-116	95%	95%	238953195	Bacteria	Proteobacteria (gamma)	n	uncultured gamma proteobacterium
JQ999338	383	18	363	4E-173	99%	99%	239835504	Bacteria	Proteobacteria (gamma)	n	uncultured gamma proteobacterium
JQ999345	526	5	474	0	100%	100%	263040682	Bacteria	Proteobacteria (gamma)	n	uncultured gamma proteobacterium
JQ999336	286	18	248	1E-116	100%	100%	295791645	Bacteria	Proteobacteria (gamma)	n	uncultured gamma proteobacterium
JQ999348	565	1	559	0	91%	91%	14334262	Bacteria	Proteobacteria (gamma)	n	uncultured gamma proteobacterium MB11B08
JQ999349	541	5	538	0	99%	99%	283486725	Bacteria	Proteobacteria (gamma)	Halomonadaceae	Halomonas sp. 2029
JQ999350	351	18	307	5E-132	96%	96%	283486726	Bacteria	Proteobacteria (gamma)	Halomonadaceae	Halomonas sp. 2034
JQ999351	272	18	232	1E-107	100%	100%	295393594	Bacteria	Proteobacteria (gamma)	Halomonadaceae	Halomonas sp. AS-11
JQ999352	335	5	164	2E-75	99%	99%	285027202	Bacteria	Proteobacteria (gamma)	Halomonadaceae	Halomonas sp. G5 1-2
JQ999353	527	89	523	5E-173	92%	92%	290457130	Bacteria	Proteobacteria (gamma)	Halomonadaceae	Halomonas sp. JW2.4a
JQ999354	252	5	197	1E-91	99%	99%	186702557	Bacteria	Proteobacteria (gamma)	Halomonadaceae	Halomonas sp. NY93B
JQ999355	307	16	258	2E-81	90%	90%	158933844	Bacteria	Proteobacteria (gamma)	Halomonadaceae	Halomonas sp. VB93
JQ999356	272	5	220	1E-106	100%	100%	32127599	Bacteria	Proteobacteria (gamma)	Halomonadaceae	uncultured Halomonas sp.
JQ999357	446	16	407	3E-179	96%	96%	52424026	Bacteria	Proteobacteria (gamma)	Halomonadaceae	uncultured Halomonas sp.
JQ999358	579	19	572	0	99%	99%	290759922	Bacteria	Proteobacteria (gamma)	Pasteurellaceae	Haemophilus haemolyticus
JQ999359	538	170	534	7E-167	96%	96%	290759923	Bacteria	Proteobacteria (gamma)	Pasteurellaceae	Haemophilus parahaemolyticus
JQ999362	360	18	315	6E-151	99%	99%	289186780	Bacteria	Proteobacteria (gamma)	Moraxellaceae	Acinetobacter sp. QT15
JQ999363	391	18	346	3E-164	99%	99%	228007453	Bacteria	Proteobacteria (gamma)	Moraxellaceae	Enhydrobacter sp. KB3-12
JQ999364	573	15	572	0	90%	90%	284434468	Bacteria	Proteobacteria (gamma)	Moraxellaceae	Enhydrobacter sp. NMC13
JQ999365	659	51	211	3E-52	91%	91%	158343584	Bacteria	Proteobacteria (gamma)	Moraxellaceae	Moraxella atlantae
JQ999366	562	17	561	1E-179	88%	88%	102620805	Bacteria	Proteobacteria (gamma)	Moraxellaceae	Moraxella bovis
JQ999367	584	18	555	0	98%	98%	98975329	Bacteria	Proteobacteria (gamma)	Moraxellaceae	Moraxella bovoculi
JQ999368	587	18	586	0	98%	98%	98975330	Bacteria	Proteobacteria (gamma)	Moraxellaceae	Moraxella bovoculi
JQ999369	561	18	555	0	94%	94%	213536823	Bacteria	Proteobacteria (gamma)	Moraxellaceae	Moraxella catarrhalis
JQ999370	262	18	186	2E-80	99%	99%	285192491	Bacteria	Proteobacteria (gamma)	Moraxellaceae	Moraxella osloensis
JQ999371	711	14	390	3E-147	92%	92%	102620806	Bacteria	Proteobacteria (gamma)	Moraxellaceae	Moraxella ovis
JQ999372	531	24	531	0	100%	100%	294992072	Bacteria	Proteobacteria (gamma)	Moraxellaceae	Moraxella sp. MJ616
JQ999373	382	14	335	3E-149	97%	97%	198404097	Bacteria	Proteobacteria (gamma)	Moraxellaceae	Moraxella sp. WPCB001
JQ999374	362	17	305	5E-147	100%	100%	164506999	Bacteria	Proteobacteria (gamma)	Moraxellaceae	Psychrobacter faecalis

JQ999375	535	42	534	0	100%	100%	38601962	Bacteria	Proteobacteria (gamma)	Moraxellaceae	Psychrobacter frigidicola
JQ999376	508	18	457	0	99%	99%	168812014	Bacteria	Proteobacteria (gamma)	Moraxellaceae	Psychrobacter maritimus
JQ999377	543	17	540	0	98%	98%	240129723	Bacteria	Proteobacteria (gamma)	Moraxellaceae	Psychrobacter maritimus
JQ999378	298	5	198	1E-27	80%	80%	6691640	Bacteria	Proteobacteria (gamma)	Moraxellaceae	Psychrobacter pacificensis
JQ999379	474	17	408	8E-166	94%	94%	85001939	Bacteria	Proteobacteria (gamma)	Moraxellaceae	Psychrobacter psychrophilus
JQ999381	531	7	465	0	97%	97%	121483518	Bacteria	Proteobacteria (gamma)	Moraxellaceae	Psychrobacter pulmonis
JQ999380	415	4	367	0	100%	100%	284813477	Bacteria	Proteobacteria (gamma)	Moraxellaceae	Psychrobacter pulmonis
JQ999382	537	5	415	0	99%	99%	259121295	Bacteria	Proteobacteria (gamma)	Moraxellaceae	Psychrobacter sp. Air226
JQ999383	531	5	531	0	94%	94%	82547996	Bacteria	Proteobacteria (gamma)	Moraxellaceae	Psychrobacter sp. B-3151
JQ999384	496	23	462	1E-178	93%	93%	116688012	Bacteria	Proteobacteria (gamma)	Moraxellaceae	Psychrobacter sp. B6
JQ999385	485	5	432	0	100%	100%	291072775	Bacteria	Proteobacteria (gamma)	Moraxellaceae	Psychrobacter sp. BSw20995
JQ999386	472	17	409	0	99%	99%	291072776	Bacteria	Proteobacteria (gamma)	Moraxellaceae	Psychrobacter sp. BSw21055
JQ999387	523	18	522	0	93%	93%	291072779	Bacteria	Proteobacteria (gamma)	Moraxellaceae	Psychrobacter sp. BSw21072
JQ999388	540	3	460	0	100%	100%	291293761	Bacteria	Proteobacteria (gamma)	Moraxellaceae	Psychrobacter sp. enrichment culture clone B1-3
JQ999389	548	22	548	0	96%	96%	291293763	Bacteria	Proteobacteria (gamma)	Moraxellaceae	Psychrobacter sp. enrichment culture clone B2-3
JQ999390	523	17	520	0	100%	100%	291293769	Bacteria	Proteobacteria (gamma)	Moraxellaceae	Psychrobacter sp. enrichment culture clone B5-2
JQ999391	406	19	358	2E-175	100%	100%	34525843	Bacteria	Proteobacteria (gamma)	Moraxellaceae	Psychrobacter sp. es9
JQ999392	361	10	310	1E-152	99%	99%	294997060	Bacteria	Proteobacteria (gamma)	Moraxellaceae	Psychrobacter sp. JT05
JQ999393	542	18	523	0	90%	90%	133740735	Bacteria	Proteobacteria (gamma)	Moraxellaceae	Psychrobacter sp. Nj-36
JQ999394	536	5	532	0	100%	100%	158562692	Bacteria	Proteobacteria (gamma)	Moraxellaceae	Psychrobacter sp. NP43
JQ999395	509	18	476	0	100%	100%	154103703	Bacteria	Proteobacteria (gamma)	Moraxellaceae	Psychrobacter sp. P11-B-9
JQ999396	312	2	249	3E-124	100%	100%	218203793	Bacteria	Proteobacteria (gamma)	Moraxellaceae	Psychrobacter sp. SCSWD17
JQ999397	442	3	302	2E-137	97%	97%	196050561	Bacteria	Proteobacteria (gamma)	Moraxellaceae	Psychrobacter sp. ThV-A
JQ999398	477	8	414	0	99%	99%	89257986	Bacteria	Proteobacteria (gamma)	Moraxellaceae	Psychrobacter sp. TSBY-37
JQ999399	301	18	268	5E-126	100%	100%	225055455	Bacteria	Proteobacteria (gamma)	Moraxellaceae	Psychrobacter sp. UST050418-045
JQ999400	555	5	540	0	95%	95%	209421415	Bacteria	Proteobacteria (gamma)	Moraxellaceae	uncultured Enhydrobacter sp.
JQ999401	419	4	364	7E-166	96%	96%	186926363	Bacteria	Proteobacteria (gamma)	Moraxellaceae	uncultured Moraxellaceae bacterium
JQ999402	320	36	161	2E-51	97%	97%	78354988	Bacteria	Proteobacteria (gamma)	Moraxellaceae	uncultured Psychrobacter sp.
JQ999403	468	2	360	1E-114	89%	89%	284010071	Bacteria	Proteobacteria (gamma)	Pseudomonadaceae	Pseudomonadaceae bacterium IZ2
JQ999406	270	157	239	2E-34	100%	100%	1907095	Bacteria	Proteobacteria (gamma)	Pseudomonadaceae	Pseudomonas asplenii
JQ999407	550	5	525	0	99%	99%	22217940	Bacteria	Proteobacteria (gamma)	Pseudomonadaceae	Pseudomonas cf. stutzeri V4.MO.16
JQ999409	526	18	505	0	93%	93%	293628582	Bacteria	Proteobacteria (gamma)	Pseudomonadaceae	Pseudomonas lutea
JQ999410	552	18	445	0	100%	100%	294999830	Bacteria	Proteobacteria (gamma)	Pseudomonadaceae	Pseudomonas panipatensis
JQ999418	463	5	404	7E-117	88%	88%	52139970	Bacteria	Proteobacteria (gamma)	Pseudomonadaceae	Pseudomonas sp. 14III/A01/008
JQ999419	538	17	510	0	100%	100%	195969270	Bacteria	Proteobacteria (gamma)	Pseudomonadaceae	Pseudomonas sp. 47
JQ999420	408	36	375	2E-157	96%	96%	15778356	Bacteria	Proteobacteria (gamma)	Pseudomonadaceae	Pseudomonas sp. 5.1
JQ999421	468	5	371	0	99%	99%	289655714	Bacteria	Proteobacteria (gamma)	Pseudomonadaceae	Pseudomonas sp. 64B-38
JQ999422	539	17	481	0	97%	97%	78038858	Bacteria	Proteobacteria (gamma)	Pseudomonadaceae	Pseudomonas sp. 7325
JQ999423	432	12	288	8E-136	99%	99%	28932767	Bacteria	Proteobacteria (gamma)	Pseudomonadaceae	Pseudomonas sp. A_wp02211
JQ999424	524	15	484	0	95%	95%	295027170	Bacteria	Proteobacteria (gamma)	Pseudomonadaceae	Pseudomonas sp. ADR45
JQ999425	503	20	431	7E-172	94%	94%	295027184	Bacteria	Proteobacteria (gamma)	Pseudomonadaceae	Pseudomonas sp. ADR62
JQ999426	509	4	460	0	100%	100%	195364246	Bacteria	Proteobacteria (gamma)	Pseudomonadaceae	Pseudomonas sp. B6_2008_
JQ999427	562	186	528	2E-142	94%	94%	116248064	Bacteria	Proteobacteria (gamma)	Pseudomonadaceae	Pseudomonas sp. BL4
JQ999428	306	18	267	4E-117	98%	98%	164419540	Bacteria	Proteobacteria (gamma)	Pseudomonadaceae	Pseudomonas sp. BSi20432
JQ999429	551	17	489	6E-163	90%	90%	239829379	Bacteria	Proteobacteria (gamma)	Pseudomonadaceae	Pseudomonas sp. c246
JQ999430	271	15	182	6E-80	99%	99%	151564445	Bacteria	Proteobacteria (gamma)	Pseudomonadaceae	Pseudomonas sp. EGU641
JQ999431	539	24	535	0	99%	99%	295345518	Bacteria	Proteobacteria (gamma)	Pseudomonadaceae	Pseudomonas sp. enrichment culture clone 13.1
JQ999432	373	5	325	2E-166	100%	100%	117551063	Bacteria	Proteobacteria (gamma)	Pseudomonadaceae	Pseudomonas sp. G1016
JQ999433	538	3	535	0	100%	100%	294861179	Bacteria	Proteobacteria (gamma)	Pseudomonadaceae	Pseudomonas sp. GN33-1
JQ999434	504	15	453	0	94%	94%	117582528	Bacteria	Proteobacteria (gamma)	Pseudomonadaceae	Pseudomonas sp. Lin 2-2
JQ999435	555	5	532	0	97%	97%	270048071	Bacteria	Proteobacteria (gamma)	Pseudomonadaceae	Pseudomonas sp. ljh-6
JQ999436	472	5	398	2E-176	96%	96%	241911410	Bacteria	Proteobacteria (gamma)	Pseudomonadaceae	Pseudomonas sp. PUT
JQ999437	541	7	449	1E-164	91%	91%	290751426	Bacteria	Proteobacteria (gamma)	Pseudomonadaceae	Pseudomonas sp. R-27204
JQ999438	520	18	480	0	100%	100%	152061209	Bacteria	Proteobacteria (gamma)	Pseudomonadaceae	Pseudomonas sp. SCT
JQ999439	350	5	303	1E-83	86%	86%	21327145	Bacteria	Proteobacteria (gamma)	Pseudomonadaceae	Pseudomonas sp. SoO1
JQ999440	556	32	555	0	97%	97%	283837557	Bacteria	Proteobacteria (gamma)	Pseudomonadaceae	Pseudomonas sp. StFLB155
JQ999441	528	15	514	0	99%	99%	284999744	Bacteria	Proteobacteria (gamma)	Pseudomonadaceae	Pseudomonas sp. SU19
JQ999442	563	18	560	0	100%	100%	158699333	Bacteria	Proteobacteria (gamma)	Pseudomonadaceae	Pseudomonas sp. SY6
JQ999443	307	4	269	5E-136	100%	100%	259090477	Bacteria	Proteobacteria (gamma)	Pseudomonadaceae	Pseudomonas sp. VS05_10
JQ999444	550	8	530	0	97%	97%	259090490	Bacteria	Proteobacteria (gamma)	Pseudomonadaceae	Pseudomonas sp. VS05_24

JQ999445	537	18	529	0	94%	94%	34525812	Bacteria	Proteobacteria (gamma)	Pseudomonadaceae	Pseudomonas sp. wp17
JQ999446	301	18	261	1E-121	100%	100%	295003953	Bacteria	Proteobacteria (gamma)	Pseudomonadaceae	Pseudomonas sp. WP6
JQ999447	504	17	467	0	93%	93%	213493549	Bacteria	Proteobacteria (gamma)	Pseudomonadaceae	Pseudomonas sp. WR8-43
JQ999448	464	5	408	4E-114	87%	87%	218158071	Bacteria	Proteobacteria (gamma)	Pseudomonadaceae	Pseudomonas sp. X31
JQ999449	446	23	423	0	100%	100%	226815632	Bacteria	Proteobacteria (gamma)	Pseudomonadaceae	Pseudomonas sp. ZR1-10
JQ999471	542	5	539	0	99%	99%	254621816	Bacteria	Proteobacteria (gamma)	Pseudomonadaceae	Pseudomonas xanthomarina
JQ999473	557	5	554	0	98%	98%	254587030	Bacteria	Proteobacteria (gamma)	Pseudomonadaceae	uncultured Pseudomonadaceae bacterium
JQ999472	541	5	340	6E-163	98%	98%	254587124	Bacteria	Proteobacteria (gamma)	Pseudomonadaceae	uncultured Pseudomonadaceae bacterium
JQ999478	452	39	401	2E-166	96%	96%	32346517	Bacteria	Proteobacteria (gamma)	Pseudomonadaceae	uncultured Pseudomonas sp.
JQ999475	351	5	305	5E-152	99%	99%	46253495	Bacteria	Proteobacteria (gamma)	Pseudomonadaceae	uncultured Pseudomonas sp.
JQ999477	431	18	386	0	100%	100%	151564597	Bacteria	Proteobacteria (gamma)	Pseudomonadaceae	uncultured Pseudomonas sp.
JQ999480	530	5	504	2E-177	90%	90%	154757022	Bacteria	Proteobacteria (gamma)	Pseudomonadaceae	uncultured Pseudomonas sp.
JQ999474	331	15	158	6E-66	99%	99%	209421463	Bacteria	Proteobacteria (gamma)	Pseudomonadaceae	uncultured Pseudomonas sp.
JQ999479	502	18	466	0	96%	96%	257072692	Bacteria	Proteobacteria (gamma)	Pseudomonadaceae	uncultured Pseudomonas sp.
JQ999476	411	5	377	0	100%	100%	283580095	Bacteria	Proteobacteria (gamma)	Pseudomonadaceae	uncultured Pseudomonas sp.
JQ999481	554	17	550	0	100%	100%	283979647	Bacteria	Proteobacteria (gamma)	Pseudomonadaceae	uncultured Pseudomonas sp.
JQ999503	439	18	174	3E-75	100%	100%	157367007	Bacteria	Proteobacteria (gamma)	Pseudomonadaceae	uncultured Pseudomonas sp.
JQ999482	451	17	376	5E-168	97%	97%	162317487	Bacteria	Proteobacteria (gamma)	Piscirickettsiaceae	uncultured Methylophaga sp.
JQ999483	496	5	447	0	100%	100%	295815419	Bacteria	Proteobacteria (gamma)	Xanthomonadaceae	Stenotrophomonas sp. I_B14
JQ999484	670	29	104	4E-26	97%	97%	254039381	Bacteria	Proteobacteria (gamma)	Xanthomonadaceae	uncultured Frateuria sp.
JQ999485	441	18	386	8E-156	94%	94%	290873614	Bacteria	Proteobacteria (gamma)	Xanthomonadaceae	uncultured Stenotrophomonas sp.
JQ999486	406	1	371	3E-125	89%	89%	295148942	Bacteria	Proteobacteria (gamma)	Xanthomonadaceae	Xanthomonas axonopodis
JQ999487	535	1	531	0	97%	97%	255339862	Bacteria	Proteobacteria	n	proteobacterium symbiont of Nilaparvata lugens
JQ999491	465	28	431	2E-172	94%	94%	89889088	Bacteria	Proteobacteria	n	uncultured proteobacterium
JQ999490	418	18	374	0	99%	99%	184190210	Bacteria	Proteobacteria	n	uncultured proteobacterium
JQ999488	282	7	223	8E-109	100%	100%	262409981	Bacteria	Proteobacteria	n	uncultured proteobacterium
JQ999489	371	5	326	6E-141	95%	95%	262410007	Bacteria	Proteobacteria	n	uncultured proteobacterium
JQ999492	284	146	197	0.00000001	91%	91%	111283638	Bacteria	Tenericutes	Acholeplasmataceae	Acholeplasma axanthum
JQ999605	564	15	559	4E-174	88%	88%	167859741	Eukaryota	Amoebozoa	Hartmannellidae	Nolandella sp. ATCC 50913
JQ999598	541	18	538	0	98%	98%	52082731	Eukaryota	Arthropoda	Entomobryidae	Entomobrya dorsosignata
JQ999599	532	180	523	3E-111	89%	89%	68144261	Eukaryota	Arthropoda	Entomobryidae	Sinella curviseta
JQ999600	313	80	250	5E-57	91%	91%	224831607	Eukaryota	Arthropoda	Mycetophilidae	Boletina plana
JQ999601	537	18	536	0	89%	89%	532978	Eukaryota	Arthropoda	Diaspididae	Aonidiella aurantii
JQ999568	445	13	312	8E-146	98%	98%	284159116	Eukaryota	Ascomycota	Mycosphaerellaceae	Mycosphaerellaceae sp. CPC 12304
JQ999575	532	5	208	7E-43	85%	85%	293630602	Eukaryota	Ascomycota	n	Dothideomycetes sp. TRN 153
JQ999570	572	5	572	0	99%	99%	169893764	Eukaryota	Ascomycota	Didymellaceae	Boeremia exigua
JQ999571	568	17	552	0	99%	99%	31415568	Eukaryota	Ascomycota	Didymellaceae	Peyronellaea glomerata
JQ999572	580	17	550	0	98%	98%	294987147	Eukaryota	Ascomycota	Didymellaceae	Phoma vasinfecta
JQ999573	302	5	166	2E-76	99%	99%	1888314	Eukaryota	Ascomycota	Leptosphaeriaceae	Leptosphaeria doliolum
JQ999574	502	17	440	0	100%	100%	288557599	Eukaryota	Ascomycota	Phaeosphaeriaceae	Phaeosphaeria sp. UZK
JQ999576	386	5	340	9E-150	96%	96%	225134682	Eukaryota	Ascomycota	Trichocomaceae	Byssochlamys spectabilis
JQ999578	591	18	589	0	98%	98%	198250453	Eukaryota	Ascomycota	Helotiaceae	Articulospora tetracladia
JQ999579	573	17	557	0	99%	99%	171673225	Eukaryota	Ascomycota	n	uncultured Ascomycota
JQ999910	437	166	390	8E-101	97%	97%	295419269	Eukaryota	Ascomycota	n	Candida orthopsilosis
JQ999580	330	3	280	6E-141	100%	100%	6537145	Eukaryota	Ascomycota	Plectosphaerellaceae	Verticillium dahliae
JQ999581	573	18	568	0	100%	100%	254028318	Eukaryota	Ascomycota	n	Nigrospora sp. SGSGf13
JQ999604	335	1	290	3E-144	99%	99%	98990721	Eukaryota	Bacillariophyta	Thalassiosiraceae	Stephanodiscus sp. FHTC11
JQ999582	567	24	564	0	99%	99%	117168475	Eukaryota	Basidiomycota	Corticiaceae	Sistotrema brinkmannii
JQ999583	446	17	399	0	99%	99%	109452380	Eukaryota	Basidiomycota	n	Rhodotorula lamellibrachiae
JQ999584	579	24	506	0	98%	98%	111283858	Eukaryota	Basidiomycota	n	Sakaguchia dacryoidea
JQ999585	551	49	532	0	95%	95%	254927417	Eukaryota	Basidiomycota	n	Leucosporidium sp. AY30
JQ999586	289	18	244	2E-94	95%	95%	225134685	Eukaryota	Basidiomycota	n	Rhodotorula glutinis
JQ999587	558	24	553	0	99%	99%	260279026	Eukaryota	Basidiomycota	n	Dioszegia rishiriensis
JQ999588	428	5	354	1E-173	99%	99%	124377860	Eukaryota	Basidiomycota	Tremellaceae	Cryptococcus neoformans
JQ999612	549	21	540	0	97%	97%	183206236	Eukaryota	Chlorophyta	Microsporaceae	Microspora stagnorum
JQ999610	544	18	544	0	99%	99%	5566332	Eukaryota	Ciliophora	Urostylidae	Uroleptus pisces
JQ999611	537	14	518	0	99%	99%	46019696	Eukaryota	Heterokontophyta	Botryidiopsidaceae	Botrydiopsis constricta
JQ999589	542	23	488	0	100%	100%	219563746	Eukaryota	n	n	fungal sp. M222
JQ999608	556	24	531	0	98%	98%	194031911	Eukaryota	n	n	uncultured eukaryote
JQ999609	556	18	62	3E-11	98%	98%	291258337	Eukaryota	n	n	uncultured eukaryote

JQ999606	385	5	335	2E-170	100%	100%	198444245	Eukaryota	n	n	uncultured eukaryote
JQ999607	581	14	578	0	98%	98%	198444246	Eukaryota	n	n	uncultured eukaryote
JQ999592	550	18	162	3E-36	88%	88%	151413658	Eukaryota	n	n	uncultured fungus
JQ999593	550	23	550	0	98%	98%	189418598	Eukaryota	n	n	uncultured fungus
JQ999590	330	5	285	3E-144	100%	100%	234195395	Eukaryota	n	n	uncultured fungus
JQ999594	581	3	558	0	99%	99%	262358083	Eukaryota	n	n	uncultured fungus
JQ999591	385	5	328	4E-168	100%	100%	289470189	Eukaryota	n	n	uncultured fungus
JQ999595	581	17	575	0	98%	98%	291551855	Eukaryota	n	n	uncultured fungus
JQ999596	339	5	278	2E-136	99%	99%	157955968	Eukaryota	n	n	uncultured marine fungus
JQ999597	538	14	525	0	95%	95%	291172885	Eukaryota	n	n	uncultured soil fungus
JQ999603	527	17	523	0	98%	98%	259129963	Eukaryota	Rotifera	Adinetidae	Adineta vaga
JQ999613	489	5	126	1E-54	99%	99%	161621688	Eukaryota	Streptophyta	Cupressaceae	Cupressus tonkinensis
JQ999614	537	17	537	0	97%	97%	170516205	Eukaryota	Streptophyta	Taxaceae	Taxus wallichiana
JQ999615	395	19	294	4E-59	82%	82%	6752453	Eukaryota	Streptophyta	Orchidaceae	Isotria verticillata
JQ999616	267	3	212	3E-103	100%	100%	45386022	Eukaryota	Streptophyta	Poaceae	Hordeum vulgare
JQ999617	339	5	273	3E-134	99%	99%	166084404	Eukaryota	Streptophyta	Caprifoliaceae	Caprifoliaceae environmental sample
JQ999618	325	5	277	4E-137	99%	99%	290782507	Eukaryota	Streptophyta	Fabaceae	Calpurnia aurea
JQ999619	419	23	369	3E-154	96%	96%	22033	Eukaryota	Streptophyta	Fabaceae	Vicia faba
JQ999621	540	6	530	0	98%	98%	6688957	Eukaryota	Streptophyta	Plantaginaceae	Plantago lanceolata
JQ999623	551	5	545	0	98%	98%	1777739	Eukaryota	Streptophyta	Ulmaceae	Zelkova serrata
JQ999624	544	24	529	0	95%	95%	7595575	Eukaryota	Streptophyta	Xanthoceraceae	Xanthoceras sorbifolium
