# Supplementary material for: Subglacial Lake Vostok (Antarctica) Accretion Ice Contains a Diverse Set of Sequences from Aquatic, Marine and Sediment-Inhabiting Bacteria and Eukarya
Source: PLoS One. 2013 Jul 3;8(7):e67221. doi: 10.1371/journal.pone.0067221 (PMC3700977; doi:10.1371/journal.pone.0067221)
Supplement: Table S2 — Large subunit rRNA gene sequences of Bacteria and Eukarya from V5. [“n” indicates information not specified in the NCBI GenBank database.]. (PDF) [file pone.0067221.s007.pdf]

Table S2. Large subunit rRNA gene sequences of Bacteria and Eukarya from V5. ["n" indicates information not specified in the NCBI GenBank database.]

Accession number	Q length	Q start	Q end	e-value	%-ident	%-sim	GI number	Domain	Phylum	Family	Genus / Species
JQ997197	529	30	519	3E-165	89%	89%	48728139	Bacteria	Actinobacteria	Frankiaceae	uncultured Frankia sp.
JQ997198	732	17	128	2E-35	93%	93%	48728167	Bacteria	Actinobacteria	Frankiaceae	uncultured Frankia sp.
JQ997196	521	26	506	4E-95	81%	81%	48728178	Bacteria	Actinobacteria	Frankiaceae	uncultured Frankia sp.
JQ997274	369	8	54	4E-14	100%	100%	289551862	Bacteria	Actinobacteria	Mycobacteriaceae	Mycobacterium abscessus
JQ999637	486	5	321	7E-62	82%	82%	269314044	Bacteria	Actinobacteria	Mycobacteriaceae	Mycobacterium immunogenum
JQ999638	554	17	509	0	92%	92%	44368	Bacteria	Actinobacteria	Mycobacteriaceae	Mycobacterium kansasii
JQ999639	552	18	455	0	93%	93%	196174916	Bacteria	Actinobacteria	Mycobacteriaceae	Mycobacterium shottsii
JQ997284	598	5	598	0	90%	90%	2414571	Bacteria	Actinobacteria	Propionibacteriaceae	Propionibacterium freudenreichii
JQ999640	567	14	560	8E-152	85%	85%	6714990	Bacteria	Actinobacteria	Thermomonosporaceae	Actinoallomurus spadix
JQ997287	501	8	306	4E-119	93%	93%	5901576	Bacteria	Actinobacteria	Thermomonosporaceae	Thermomonospora chromogena
JQ999641	332	26	295	8E-115	95%	95%	291045144	Bacteria	Actinobacteria	Bifidobacteriaceae	Bifidobacterium bifidum
JQ999642	349	19	255	5E-82	90%	90%	30313593	Bacteria	Bacteroidetes	Bacteroidaceae	Bacteroides caccae
JQ997308	588	20	582	0	90%	90%	213536826	Bacteria	Bacteroidetes	Bacteroidaceae	Bacteroides ovatus
JQ999643	565	19	563	0	89%	89%	30313596	Bacteria	Bacteroidetes	Bacteroidaceae	Bacteroides stercoris
JQ997309	589	17	587	0	88%	88%	213536825	Bacteria	Bacteroidetes	Bacteroidaceae	Bacteroides vulgatus
JQ999644	301	83	269	2E-55	88%	88%	56744982	Bacteria	Bacteroidetes	n	uncultured Bacteroidales bacterium
JQ999645	347	55	295	2E-90	92%	92%	56744983	Bacteria	Bacteroidetes	n	uncultured Bacteroidales bacterium
JQ999646	552	40	550	0	90%	90%	30313598	Bacteria	Bacteroidetes	Porphyromonadaceae	Parabacteroides merdae
JQ999829	266	24	226	1E-77	93%	93%	38374131	Bacteria	Bacteroidetes	Cytophagaceae	Flexibacter flexilis
JQ999647	666	129	385	3E-92	91%	91%	46409892	Bacteria	Cyanobacteria	n	Gloeobacter violaceus
JQ999648	500	65	449	2E-146	91%	91%	46409882	Bacteria	Cyanobacteria	n	Euhalothece sp. BAA001
JQ997361	360	18	327	9E-85	86%	86%	222138150	Bacteria	Cyanobacteria	n	Gloeocapsopsis crepidinum
JQ999649	291	20	259	7E-110	97%	97%	90186509	Bacteria	Cyanobacteria	n	Synechococcus sp. C9
JQ997393	625	18	621	0	90%	90%	225696243	Bacteria	Cyanobacteria	n	uncultured cyanobacterium
JQ997384	515	4	438	9E-176	93%	93%	225696245	Bacteria	Cyanobacteria	n	uncultured cyanobacterium
JQ997375	383	5	246	1E-88	93%	93%	227072227	Bacteria	Cyanobacteria	n	uncultured cyanobacterium
JQ997374	361	18	307	8E-135	97%	97%	256692872	Bacteria	Cyanobacteria	n	uncultured cyanobacterium
JQ999650	558	26	555	0	97%	97%	46409901	Bacteria	Cyanobacteria	n	Leptolyngbya boryana
JQ999651	581	15	530	0	97%	97%	46409896	Bacteria	Cyanobacteria	n	Leptolyngbya sp. PCC 7104
JQ997443	551	5	545	0	93%	93%	149364162	Bacteria	Cyanobacteria	n	Microcoleus vaginatus
JQ999652	588	2	583	0	96%	96%	46409893	Bacteria	Cyanobacteria	n	Oscillatoria sp. PCC 6506
JQ999653	516	5	474	1E-168	90%	90%	46409900	Bacteria	Cyanobacteria	n	Plectonema terebrans
JQ999654	356	53	154	3E-29	91%	91%	281376717	Bacteria	Firmicutes	Alicyclobacillaceae	Alicyclobacillus sp. Z27
JQ999745	223	4	209	2E-69	91%	91%	347582959	Bacteria	Firmicutes	Bacillaceae	Bacillus
JQ999655	442	10	327	1E-113	91%	91%	20126656	Bacteria	Firmicutes	Bacillaceae	Bacillus cereus
JQ999656	587	14	587	0	94%	94%	241896768	Bacteria	Firmicutes	Bacillaceae	Bacillus licheniformis
JQ999657	525	13	466	0	95%	95%	152211809	Bacteria	Firmicutes	Listeriaceae	Listeria grayi
JQ999658	548	50	548	8E-97	81%	81%	152211814	Bacteria	Firmicutes	Listeriaceae	Listeria innocua
JQ999659	540	4	539	2E-138	84%	84%	296465	Bacteria	Firmicutes	Listeriaceae	Listeria monocytogenes
JQ999660	571	6	561	0	89%	89%	296416	Bacteria	Firmicutes	Planococcaceae	Sporosarcina globispora
JQ999665	579	18	575	0	97%	97%	93463980	Bacteria	Firmicutes	Staphylococcaceae	Staphylococcus xylosus
JQ999666	506	5	450	2E-157	90%	90%	11342510	Bacteria	Firmicutes	Enterococcaceae	Enterococcus asini
JQ999667	564	5	518	0	94%	94%	11342519	Bacteria	Firmicutes	Enterococcaceae	Enterococcus casseliflavus
JQ999668	549	18	496	0	94%	94%	11342513	Bacteria	Firmicutes	Enterococcaceae	Enterococcus cecorum
JQ999669	550	8	541	0	94%	94%	11342514	Bacteria	Firmicutes	Enterococcaceae	Enterococcus columbae
JQ999670	577	5	574	0	93%	93%	11342515	Bacteria	Firmicutes	Enterococcaceae	Enterococcus dispar
JQ999671	435	18	379	2E-146	93%	93%	183673657	Bacteria	Firmicutes	Enterococcaceae	Enterococcus faecium
JQ999672	506	7	439	0	94%	94%	183673673	Bacteria	Firmicutes	Enterococcaceae	Enterococcus faecium
JQ999674	616	13	193	2E-33	83%	83%	11078579	Bacteria	Firmicutes	Enterococcaceae	Enterococcus gallinarum
JQ999673	564	1	561	0	90%	90%	11342520	Bacteria	Firmicutes	Enterococcaceae	Enterococcus gallinarum
JQ999675	563	41	561	7E-177	89%	89%	11342522	Bacteria	Firmicutes	Enterococcaceae	Enterococcus malodoratus
JQ999676	570	5	505	0	91%	91%	11342523	Bacteria	Firmicutes	Enterococcaceae	Enterococcus mundtii
JQ999677	361	139	329	1E-77	95%	95%	11342524	Bacteria	Firmicutes	Enterococcaceae	Enterococcus pseudoavium
JQ999678	552	18	551	0	95%	95%	11342525	Bacteria	Firmicutes	Enterococcaceae	Enterococcus raffinosus
JQ999679	462	5	425	6E-167	92%	92%	11342526	Bacteria	Firmicutes	Enterococcaceae	Enterococcus saccharolyticus

JQ999680	561	21	378	2E-138	92%	92%	11342528	Bacteria	Firmicutes	Enterococcaceae	Enterococcus sulfureus
JQ999681	380	19	285	9E-95	91%	91%	11342596	Bacteria	Firmicutes	Enterococcaceae	Melissococcus plutonius
JQ999682	447	153	398	1E-114	98%	98%	11342527	Bacteria	Firmicutes	Enterococcaceae	Tetragenococcus solitarius
JQ999683	243	80	219	8E-54	94%	94%	164664890	Bacteria	Firmicutes	Enterococcaceae	uncultured Enterococcus sp.
JQ999684	362	17	120	3E-39	96%	96%	164664896	Bacteria	Firmicutes	Enterococcaceae	uncultured Enterococcus sp.
JQ997623	582	17	538	0	99%	99%	50080123	Bacteria	Firmicutes	Lactobacillaceae	Lactobacillus animalis
JQ997624	539	18	277	8E-77	87%	87%	190360905	Bacteria	Firmicutes	Lactobacillaceae	Lactobacillus brevis
JQ997651	537	23	534	0	94%	94%	50080122	Bacteria	Firmicutes	Lactobacillaceae	Lactobacillus murinus
JQ999685	375	18	238	1E-107	99%	99%	167046809	Bacteria	Firmicutes	Lactobacillaceae	Pediococcus claussenii
JQ999686	557	18	490	8E-97	81%	81%	167046804	Bacteria	Firmicutes	Lactobacillaceae	Pediococcus parvulus
JQ999687	552	18	547	1E-109	81%	81%	167046808	Bacteria	Firmicutes	Lactobacillaceae	Pediococcus pentosaceus
JQ999688	386	24	337	5E-127	93%	93%	167047104	Bacteria	Firmicutes	Lactobacillaceae	Pediococcus stilesii
JQ999689	557	5	535	8E-152	85%	85%	45597358	Bacteria	Firmicutes	Streptococcaceae	Streptococcus canis
JQ999690	586	4	231	2E-83	91%	91%	2897684	Bacteria	Firmicutes	Streptococcaceae	Streptococcus constellatus
JQ999692	513	18	449	0	94%	94%	25396587	Bacteria	Firmicutes	Streptococcaceae	Streptococcus dysgalactiae
JQ999691	328	54	190	1E-58	98%	98%	281331096	Bacteria	Firmicutes	Streptococcaceae	Streptococcus dysgalactiae
JQ999693	545	5	539	0	91%	91%	281331108	Bacteria	Firmicutes	Streptococcaceae	Streptococcus dysgalactiae
JQ999694	527	5	341	2E-148	95%	95%	45597360	Bacteria	Firmicutes	Streptococcaceae	Streptococcus equi
JQ999695	546	18	505	0	98%	98%	45597357	Bacteria	Firmicutes	Streptococcaceae	Streptococcus equinus
JQ999696	570	5	558	0	93%	93%	45597361	Bacteria	Firmicutes	Streptococcaceae	Streptococcus equinus
JQ999697	499	2	467	0	93%	93%	45597366	Bacteria	Firmicutes	Streptococcaceae	Streptococcus hyointestinalis
JQ997695	526	201	486	7E-147	100%	100%	11991762	Bacteria	Firmicutes	Streptococcaceae	Streptococcus mutans
JQ997698	576	4	570	0	92%	92%	213536840	Bacteria	Firmicutes	Streptococcaceae	Streptococcus mutans
JQ999698	594	5	556	0	89%	89%	288522	Bacteria	Firmicutes	Streptococcaceae	Streptococcus oralis
JQ999699	434	16	395	7E-176	96%	96%	433515	Bacteria	Firmicutes	Streptococcaceae	Streptococcus parauberis
JQ999880	262	1	262	2E-101	93%	93%	160426828	Bacteria	Firmicutes	Clostridiaceae	Clostridium
JQ997801	291	5	225	5E-91	95%	95%	213536838	Bacteria	Firmicutes	Peptostreptococcaceae	Peptostreptococcus anaerobius
JQ997804	456	12	391	3E-125	89%	89%	3821805	Bacteria	Firmicutes	Erysipelotrichaceae	Erysipelothrix rhusiopathiae
JQ999702	554	59	550	0	90%	90%	288510	Bacteria	Firmicutes	Veillonellaceae	Pectinatus frisingensis
JQ997847	422	18	268	2E-122	99%	99%	213536832	Bacteria	Fusobacteria	Fusobacteriaceae	Fusobacterium necrophorum
JQ999703	562	9	308	1E-95	89%	89%	15028908	Bacteria	Fusobacteria	Fusobacteriaceae	Fusobacterium nucleatum
JQ999704	570	27	561	2E-152	86%	86%	15029008	Bacteria	Fusobacteria	Fusobacteriaceae	Ilyobacter polytropus
JQ999705	565	13	551	6E-163	87%	87%	15029012	Bacteria	Fusobacteria	Fusobacteriaceae	Propionigenium maris
JQ999706	321	4	270	3E-104	93%	93%	15029011	Bacteria	Fusobacteria	Fusobacteriaceae	Propionigenium modestum
JQ999716	304	18	273	9E-114	96%	96%	41350813	Bacteria	n	n	uncultured bacterium
JQ999721	327	24	283	6E-116	96%	96%	41350814	Bacteria	n	n	uncultured bacterium
JQ999734	385	18	337	7E-151	97%	97%	224814922	Bacteria	n	n	uncultured bacterium
JQ999730	365	5	295	3E-109	92%	92%	240002803	Bacteria	n	n	uncultured bacterium
JQ999777	552	17	433	0	96%	96%	291258506	Bacteria	n	n	uncultured bacterium
JQ999760	500	17	457	0	94%	94%	291258521	Bacteria	n	n	uncultured bacterium
JQ999736	398	11	346	4E-153	96%	96%	291258526	Bacteria	n	n	uncultured bacterium
JQ999772	544	17	487	0	92%	92%	291258532	Bacteria	n	n	uncultured bacterium
JQ999773	544	17	543	6E-138	84%	84%	291258599	Bacteria	n	n	uncultured bacterium
JQ999743	423	15	304	5E-93	88%	88%	291258613	Bacteria	n	n	uncultured bacterium
JQ999788	572	14	564	0	94%	94%	291258642	Bacteria	n	n	uncultured bacterium
JQ999793	583	19	515	0	91%	91%	291258644	Bacteria	n	n	uncultured bacterium
JQ999749	441	154	390	1E-89	92%	92%	291258653	Bacteria	n	n	uncultured bacterium
JQ999785	566	5	268	5E-104	93%	93%	291258669	Bacteria	n	n	uncultured bacterium
JQ999761	500	5	468	3E-150	88%	88%	291258684	Bacteria	n	n	uncultured bacterium
JQ999797	696	5	233	3E-87	93%	93%	291258703	Bacteria	n	n	uncultured bacterium
JQ999746	431	5	81	7E-22	94%	94%	291258731	Bacteria	n	n	uncultured bacterium
JQ999714	299	18	240	2E-105	98%	98%	291258817	Bacteria	n	n	uncultured bacterium
JQ999758	490	3	444	1E-153	89%	89%	291258864	Bacteria	n	n	uncultured bacterium
JQ999751	448	18	437	3E-175	94%	94%	291258875	Bacteria	n	n	uncultured bacterium
JQ999715	300	4	242	7E-100	95%	95%	291258896	Bacteria	n	n	uncultured bacterium
JQ999712	270	26	229	9E-44	83%	83%	291258991	Bacteria	n	n	uncultured bacterium
JQ999774	545	137	529	6E-158	93%	93%	291259015	Bacteria	n	n	uncultured bacterium
JQ999724	337	5	251	5E-112	97%	97%	291259090	Bacteria	n	n	uncultured bacterium
JQ999713	279	5	223	3E-63	88%	88%	291259092	Bacteria	n	n	uncultured bacterium
JQ999748	433	20	363	2E-157	96%	96%	291259094	Bacteria	n	n	uncultured bacterium

JQ999757	486	18	422	2E-151	91%	91%	291259109	Bacteria	n	n	uncultured bacterium
JQ999784	565	29	535	1E-165	88%	88%	291259178	Bacteria	n	n	uncultured bacterium
JQ999769	536	47	532	0	91%	91%	291259189	Bacteria	n	n	uncultured bacterium
JQ999770	536	193	474	1E-109	93%	93%	291259240	Bacteria	n	n	uncultured bacterium
JQ999764	518	24	461	0	94%	94%	291259266	Bacteria	n	n	uncultured bacterium
JQ999756	483	5	426	0	95%	95%	291259291	Bacteria	n	n	uncultured bacterium
JQ999709	266	5	216	2E-60	88%	88%	291259334	Bacteria	n	n	uncultured bacterium
JQ999741	421	5	376	7E-151	93%	93%	291259383	Bacteria	n	n	uncultured bacterium
JQ999794	583	82	580	0	93%	93%	291259408	Bacteria	n	n	uncultured bacterium
JQ999755	456	76	431	4E-84	83%	83%	291259424	Bacteria	n	n	uncultured bacterium
JQ999778	557	5	524	0	89%	89%	291259440	Bacteria	n	n	uncultured bacterium
JQ999720	318	18	260	8E-70	87%	87%	291259455	Bacteria	n	n	uncultured bacterium
JQ999795	614	29	564	1E-145	86%	86%	291259482	Bacteria	n	n	uncultured bacterium
JQ999754	455	149	358	3E-25	78%	78%	291259493	Bacteria	n	n	uncultured bacterium
JQ999725	342	5	258	5E-107	94%	94%	291259502	Bacteria	n	n	uncultured bacterium
JQ999780	559	63	313	9E-72	87%	87%	291259509	Bacteria	n	n	uncultured bacterium
JQ999738	404	28	148	1E-38	92%	92%	291259601	Bacteria	n	n	uncultured bacterium
JQ999753	452	175	411	2E-71	88%	88%	291259787	Bacteria	n	n	uncultured bacterium
JQ999787	569	429	551	3E-32	89%	89%	291259805	Bacteria	n	n	uncultured bacterium
JQ999781	559	21	525	0	98%	98%	291259832	Bacteria	n	n	uncultured bacterium
JQ999775	548	26	543	2E-177	89%	89%	291259953	Bacteria	n	n	uncultured bacterium
JQ999732	368	5	333	1E-132	93%	93%	291259969	Bacteria	n	n	uncultured bacterium
JQ999708	261	6	211	3E-63	89%	89%	291260077	Bacteria	n	n	uncultured bacterium
JQ999711	269	5	227	4E-77	91%	91%	291260092	Bacteria	n	n	uncultured bacterium
JQ999768	535	9	410	3E-155	92%	92%	291260192	Bacteria	n	n	uncultured bacterium
JQ999783	564	18	463	1E-169	91%	91%	291260193	Bacteria	n	n	uncultured bacterium
JQ999786	566	17	553	2E-163	87%	87%	291260258	Bacteria	n	n	uncultured bacterium
JQ999796	666	5	311	8E-93	88%	88%	291260323	Bacteria	n	n	uncultured bacterium
JQ999707	248	5	190	1E-71	94%	94%	291260330	Bacteria	n	n	uncultured bacterium
JQ999752	451	15	392	3E-175	96%	96%	291260331	Bacteria	n	n	uncultured bacterium
JQ999767	533	18	480	1E-144	87%	87%	291260367	Bacteria	n	n	uncultured bacterium
JQ999719	314	185	268	2E-31	98%	98%	291260384	Bacteria	n	n	uncultured bacterium
JQ999766	532	15	516	3E-146	86%	86%	291260462	Bacteria	n	n	uncultured bacterium
JQ999710	268	18	223	1E-97	99%	99%	291260506	Bacteria	n	n	uncultured bacterium
JQ999733	383	5	289	9E-115	93%	93%	291260539	Bacteria	n	n	uncultured bacterium
JQ999765	520	22	452	0	96%	96%	291260594	Bacteria	n	n	uncultured bacterium
JQ999717	306	4	258	1E-102	94%	94%	291260683	Bacteria	n	n	uncultured bacterium
JQ999737	399	5	341	7E-131	92%	92%	291260701	Bacteria	n	n	uncultured bacterium
JQ999729	362	18	261	2E-90	92%	92%	291260769	Bacteria	n	n	uncultured bacterium
JQ999740	413	5	368	4E-158	95%	95%	291260837	Bacteria	n	n	uncultured bacterium
JQ999779	558	5	382	1E-159	94%	94%	291260876	Bacteria	n	n	uncultured bacterium
JQ999718	312	5	253	2E-76	88%	88%	291260895	Bacteria	n	n	uncultured bacterium
JQ999762	501	17	431	2E-176	94%	94%	291260953	Bacteria	n	n	uncultured bacterium
JQ999791	576	28	533	3E-91	80%	80%	291261001	Bacteria	n	n	uncultured bacterium
JQ999735	388	24	322	1E-108	91%	91%	291261015	Bacteria	n	n	uncultured bacterium
JQ999744	426	5	282	5E-53	82%	82%	291261049	Bacteria	n	n	uncultured bacterium
JQ999782	562	20	537	0	92%	92%	291261180	Bacteria	n	n	uncultured bacterium
JQ999776	550	45	485	1E-149	89%	89%	291261284	Bacteria	n	n	uncultured bacterium
JQ999763	517	27	473	0	96%	96%	291261295	Bacteria	n	n	uncultured bacterium
JQ999731	365	29	238	7E-81	93%	93%	291261310	Bacteria	n	n	uncultured bacterium
JQ999750	441	18	404	1E-179	96%	96%	291261311	Bacteria	n	n	uncultured bacterium
JQ999726	345	5	296	8E-140	98%	98%	291261346	Bacteria	n	n	uncultured bacterium
JQ999792	576	16	507	1E-70	78%	78%	291261350	Bacteria	n	n	uncultured bacterium
JQ999771	537	18	436	0	95%	95%	291261377	Bacteria	n	n	uncultured bacterium
JQ999759	490	2	160	5E-59	94%	94%	291261380	Bacteria	n	n	uncultured bacterium
JQ999723	336	23	306	3E-104	91%	91%	291261400	Bacteria	n	n	uncultured bacterium
JQ999722	335	18	202	3E-79	96%	96%	291261412	Bacteria	n	n	uncultured bacterium
JQ999747	432	19	382	3E-175	98%	98%	291261484	Bacteria	n	n	uncultured bacterium
JQ999789	575	33	523	0	92%	92%	291261504	Bacteria	n	n	uncultured bacterium
JQ999790	575	25	537	0	96%	96%	291261577	Bacteria	n	n	uncultured bacterium

JQ999739	412	5	177	2E-82	99%	99%	291261679	Bacteria	n	n	uncultured bacterium
JQ999728	355	17	264	3E-104	95%	95%	291261696	Bacteria	n	n	uncultured bacterium
JQ999742	422	135	308	5E-33	83%	83%	291261700	Bacteria	n	n	uncultured bacterium
JQ999727	347	5	302	2E-140	97%	97%	291261750	Bacteria	n	n	uncultured bacterium
JQ999798	586	18	582	0	91%	91%	12583964	Bacteria	Planctomycetes	Planctomycetaceae	Pirellula staleyi
JQ999799	581	18	577	0	90%	90%	2244633	Bacteria	Proteobacteria (alpha)	Caulobacteraceae	Brevundimonas diminuta
JQ999800	338	5	291	4E-68	84%	84%	32328286	Bacteria	Proteobacteria (alpha)	n	unknown marine alpha proteobacterium JP57
JQ999801	557	5	554	0	98%	98%	29725918	Bacteria	Proteobacteria (alpha)	Phyllobacteriaceae	Mesorhizobium loti
JQ999802	585	4	584	2E-178	87%	87%	197734889	Bacteria	Proteobacteria (alpha)	Rhizobiaceae	Rhizobium gallicum
JQ999803	559	24	278	2E-87	90%	90%	138754396	Bacteria	Proteobacteria (alpha)	Rhizobiaceae	Rhizobium giardinii
JQ999804	577	16	569	0	91%	91%	138754393	Bacteria	Proteobacteria (alpha)	Rhizobiaceae	Sinorhizobium arboris
JQ999805	412	5	338	2E-137	94%	94%	2244670	Bacteria	Proteobacteria (alpha)	Rhodobacteraceae	Paracoccus denitrificans
JQ999806	413	24	362	2E-116	90%	90%	89277242	Bacteria	Proteobacteria (alpha)	n	Caedibacter caryophilus
JQ999807	526	5	137	4E-60	99%	99%	32328294	Bacteria	Proteobacteria (alpha)	Sphingomonadaceae	Sphingomonas sp. KT0216
JQ999809	445	4	368	2E-162	95%	95%	290796637	Bacteria	Proteobacteria (beta)	n	uncultured Burkholderiales bacterium
JQ999808	359	16	349	2E-160	98%	98%	290796638	Bacteria	Proteobacteria (beta)	n	uncultured Burkholderiales bacterium
JQ999810	240	96	206	1E-32	91%	91%	73533084	Bacteria	Proteobacteria (beta)	Oxalobacteraceae	Herbaspirillum autotrophicum
JQ999811	523	83	522	4E-174	92%	92%	73533083	Bacteria	Proteobacteria (beta)	Oxalobacteraceae	Herbaspirillum huttiense
JQ999812	535	18	524	0	96%	96%	50957144	Bacteria	Proteobacteria (epsilon)	Helicobacteraceae	Helicobacter cholecystus
JQ999323	584	18	540	0	90%	90%	213536822	Bacteria	Proteobacteria (gamma)	Succinivibrionaceae	Anaerobiospirillum succiniciproducens
JQ999327	311	19	268	2E-80	89%	89%	213536828	Bacteria	Proteobacteria (gamma)	Cardiobacteriaceae	Cardiobacterium hominis
JQ999813	485	6	420	3E-175	94%	94%	225182735	Bacteria	Proteobacteria (gamma)	Halomonadaceae	Halomonas axialensis
JQ999814	574	5	517	0	98%	98%	225182736	Bacteria	Proteobacteria (gamma)	Halomonadaceae	Halomonas boliviensis
JQ999815	555	18	551	0	95%	95%	225182737	Bacteria	Proteobacteria (gamma)	Halomonadaceae	Halomonas neptunia
JQ999816	560	13	558	0	90%	90%	168148844	Bacteria	Proteobacteria (gamma)	Halomonadaceae	Halomonas sulfidaeris
JQ999817	354	51	308	4E-53	83%	83%	17976821	Bacteria	Proteobacteria (gamma)	Halomonadaceae	Halomonas variabilis
JQ999818	522	18	472	2E-137	87%	87%	225735345	Bacteria	Proteobacteria (gamma)	Halomonadaceae	Salinicola halophilus
JQ999819	562	5	557	0	92%	92%	164451918	Bacteria	Proteobacteria (gamma)	Pasteurellaceae	Actinobacillus suis
JQ999820	574	21	559	0	95%	95%	2244630	Bacteria	Proteobacteria (gamma)	Moraxellaceae	Acinetobacter calcoaceticus
JQ999361	576	5	576	0	97%	97%	127463238	Bacteria	Proteobacteria (gamma)	Moraxellaceae	Acinetobacter calcoaceticus
JQ999822	310	5	272	5E-126	98%	98%	1913845	Bacteria	Proteobacteria (gamma)	Xanthomonadaceae	Xanthomonas fragariae
JQ999823	339	25	308	6E-146	100%	100%	288225748	Bacteria	Spirochaetes	Brachyspiraceae	Brachyspira pilosicoli
JQ999824	460	5	412	0	100%	100%	288225749	Bacteria	Spirochaetes	Brachyspiraceae	Brachyspira pilosicoli
JQ999825	468	181	404	2E-102	97%	97%	288225746	Bacteria	Spirochaetes	Brachyspiraceae	Brachyspira sp. PT.C
JQ999826	414	18	382	2E-67	81%	81%	294768449	Bacteria	Tenericutes	Acholeplasmataceae	Acholeplasma equifetale
JQ999493	559	2	458	4E-145	88%	88%	294660656	Bacteria	Tenericutes	Spiroplasmataceae	Spiroplasma diabroticae
JQ999827	458	5	359	2E-126	90%	90%	294768451	Bacteria	Tenericutes	Mycoplasmataceae	Mycoplasma feliminutum
JQ999828	465	23	390	4E-179	98%	98%	183579829	Bacteria	Verrucomicrobia	Verrucomicrobia subdivision 3	Pedosphaera parvula
JQ999877	538	18	519	0	99%	99%	148372021	Eukaryota	Arthropoda	Entomobryidae	Lepidocyrtus sp. Yan Gao 06126
JQ999878	542	25	541	0	97%	97%	145308394	Eukaryota	Arthropoda	Entomobryidae	Sinella curviseta
JQ999876	445	33	83	1E-09	92%	92%	59890651	Eukaryota	Arthropoda	Silphidae	Silpha obscura
JQ999875	258	18	214	1E-82	96%	96%	202070905	Eukaryota	Arthropoda	Trichoceridae	Trichocera brevicornis
JQ999837	553	5	550	0	100%	100%	284158823	Eukaryota	Ascomycota	Davidiellaceae	Davidiella tassiana
JQ999838	553	2	516	0	97%	97%	284158872	Eukaryota	Ascomycota	Mycosphaerellaceae	Passalora perplexa
JQ999839	413	136	362	2E-97	96%	96%	283827928	Eukaryota	Ascomycota	Mycosphaerellaceae	Verrucisporota daviesiae
JQ999862	348	5	301	8E-150	99%	99%	282160302	Eukaryota	Ascomycota	Didymellaceae	Phoma infossa
JQ999865	503	5	457	0	100%	100%	294987020	Eukaryota	Ascomycota	Didymellaceae	Phoma macrostoma
JQ999872	552	89	548	0	97%	97%	294987076	Eukaryota	Ascomycota	Didymellaceae	Phoma viburnicola
JQ999863	419	5	364	0	99%	99%	294987114	Eukaryota	Ascomycota	Didymellaceae	Stagonosporopsis rudbeckiae
JQ999861	268	4	215	5E-101	99%	99%	294987118	Eukaryota	Ascomycota	Didymellaceae	Stagonosporopsis valerianellae
JQ999874	868	17	128	1E-42	96%	96%	290790471	Eukaryota	Ascomycota	Lentitheciaceae	Lentitheciaceae sp. A369-1
JQ999866	522	24	457	0	100%	100%	284192847	Eukaryota	Ascomycota	Phaeosphaeriaceae	Phaeodothis winteri
JQ999841	561	24	559	0	100%	100%	154563033	Eukaryota	Ascomycota	Phaeosphaeriaceae	Phaeosphaeria avenaria
JQ999840	558	18	525	0	96%	96%	154563036	Eukaryota	Ascomycota	Phaeosphaeriaceae	Phaeosphaeria avenaria
JQ999842	569	5	554	0	97%	97%	159171560	Eukaryota	Ascomycota	Phaeosphaeriaceae	Phaeosphaeria avenaria
JQ999869	542	5	541	0	100%	100%	208879720	Eukaryota	Ascomycota	Phaeosphaeriaceae	Phaeosphaeria nodorum
JQ999843	437	24	384	0	99%	99%	290760004	Eukaryota	Ascomycota	Phaeosphaeriaceae	Phaeosphaeria spartinicola
JQ999873	555	24	555	0	96%	96%	289449251	Eukaryota	Ascomycota	Pleosporaceae	Alternaria tenuissima
JQ999855	521	233	457	6E-108	99%	99%	90577143	Eukaryota	Ascomycota	n	Candida ontarioensis
JQ999856	212	11	225	1E-55	86%	86%	90577143	Eukaryota	Ascomycota	n	mitosporic Saccharomycetales

JQ999858	564	18	560	0	99%	99%	156099766	Eukaryota	Ascomycota	Saccharomycetaceae	Cyberlindnera jadinii
JQ999879	557	270	548	3E-121	95%	95%	18698476	Eukaryota	Bacillariophyta	Bacillariaceae	Hantzschia amphioxys
JQ999844	336	18	305	4E-108	92%	92%	148358050	Eukaryota	Basidiomycota	Clavariaceae	Macrotyphula fistulosa
JQ999845	477	15	410	2E-166	94%	94%	148358060	Eukaryota	Basidiomycota	Cortinariaceae	Leucocortinarius bulbiger
JQ999846	339	13	294	2E-120	95%	95%	148358057	Eukaryota	Basidiomycota	Nidulariaceae	Cyathus striatus
JQ999847	455	21	396	1E-144	92%	92%	84873683	Eukaryota	Basidiomycota	Physalacriaceae	Armillaria hinnulea
JQ999848	507	29	271	2E-112	98%	98%	54695062	Eukaryota	Basidiomycota	Tulasnellaceae	uncultured Tulasnellaceae
JQ999860	268	16	210	9E-44	84%	84%	256859920	Eukaryota	Basidiomycota	Geastraceae	Geastrum sessile
JQ999871	551	4	541	2E-153	86%	86%	34915795	Eukaryota	Basidiomycota	Gloeophyllaceae	Gloeophyllum sepiarium
JQ999864	429	27	253	1E-68	88%	88%	34915791	Eukaryota	Basidiomycota	Coriolaceae	Donkioporia expansa
JQ999849	547	5	543	7E-162	86%	86%	77379307	Eukaryota	Basidiomycota	Ganodermataceae	Ganoderma lucidum
JQ999868	537	19	516	0	96%	96%	117164085	Eukaryota	Basidiomycota	Polyporaceae	Fibroporia vaillantii
JQ999850	554	18	538	0	95%	95%	110704320	Eukaryota	Basidiomycota	Malasseziaceae	Malassezia cf. restricta HN3127
JQ999851	545	20	542	0	98%	98%	52631074	Eukaryota	Basidiomycota	Malasseziaceae	Malassezia pachydermatis
JQ999867	536	20	536	0	90%	90%	256859939	Eukaryota	Basidiomycota	Tilletiaceae	Tilletia olida
JQ999852	346	17	300	4E-138	98%	98%	111283857	Eukaryota	Basidiomycota	n	Sakaguchia dacryoidea
JQ999853	546	17	546	0	100%	100%	224979500	Eukaryota	Basidiomycota	n	Cryptococcus sp. ATT123
JQ999859	468	4	385	2E-111	87%	87%	304472	Eukaryota	Basidiomycota	Tremellaceae	Cryptococcus gattii
JQ999889	383	21	334	7E-81	85%	85%	17028321	Eukaryota	Chlorophyta	n	Chlorosarcina brevispinosa
JQ999905	550	18	546	0	92%	92%	237648547	Eukaryota	Chlorophyta	Chlorellaceae	Chlorella variabilis
JQ999890	561	8	557	0	93%	93%	535783	Eukaryota	Chlorophyta	Chlorellaceae	Pseudochlorella pringsheimii
JQ999891	596	141	595	1E-145	89%	89%	12667510	Eukaryota	Chlorophyta	n	Trichosarcina mucosa
JQ999886	531	18	528	0	93%	93%	220966760	Eukaryota	Ciliophora	Oxytrichidae	Sterkiella histriomuscorum
JQ999881	416	5	221	1E-98	97%	97%	157411062	Eukaryota	Heterokontophyta	Halosiphonaceae	Halosiphon tomentosus
JQ999857	542	18	539	0	99%	99%	85700735	Eukaryota	n	n	uncultured compost fungus
JQ999887	575	18	553	0	90%	90%	291261822	Eukaryota	n	n	uncultured eukaryote
JQ999888	580	25	557	0	89%	89%	291261826	Eukaryota	n	n	uncultured eukaryote
JQ999883	348	18	311	3E-119	93%	93%	291263034	Eukaryota	n	n	uncultured eukaryote
JQ999885	465	24	408	4E-159	94%	94%	291263118	Eukaryota	n	n	uncultured eukaryote
JQ999884	401	86	357	3E-109	93%	93%	291263535	Eukaryota	n	n	uncultured eukaryote
JQ999882	281	4	233	1E-112	99%	99%	291263834	Eukaryota	n	n	uncultured eukaryote
JQ999854	528	26	525	0	92%	92%	239819331	Eukaryota	n	n	uncultured fungus
JQ999904	581	36	577	0	97%	97%	170516193	Eukaryota	Streptophyta	Taxaceae	Taxus wallichiana
JQ999892	392	5	113	1E-23	87%	87%	109138712	Eukaryota	Streptophyta	Ptilidiaceae	Ptilidium pulcherrimum
JQ999893	538	33	538	0	97%	97%	66969404	Eukaryota	Streptophyta	Tofieldiaceae	Tofieldia calyculata
JQ999894	547	106	523	2E-178	94%	94%	109138849	Eukaryota	Streptophyta	Amborellaceae	Amborella trichopoda
JQ999895	307	5	275	3E-118	96%	96%	109138856	Eukaryota	Streptophyta	Chloranthaceae	Hedyosmum arborescens
JQ999897	355	102	321	2E-100	97%	97%	22595025	Eukaryota	Streptophyta	Hydrangeaceae	Philadelphus lewisii
JQ999899	225	1	214	2E-94	96%	96%	57340766	Eukaryota	Streptophyta	Lecythidaceae	Napoleona
JQ999903	572	15	559	0	95%	95%	62902790	Eukaryota	Streptophyta	Fabaceae	Glycine max
JQ999902	549	18	491	0	98%	98%	19655	Eukaryota	Streptophyta	Fabaceae	Medicago sativa
JQ999901	542	5	349	1E-139	93%	93%	267850659	Eukaryota	Streptophyta	Fabaceae	Spatholobus suberectus
