# Supplementary material for: Subglacial Lake Vostok (Antarctica) Accretion Ice Contains a Diverse Set of Sequences from Aquatic, Marine and Sediment-Inhabiting Bacteria and Eukarya
Source: PLoS One. 2013 Jul 3;8(7):e67221. doi: 10.1371/journal.pone.0067221 (PMC3700977; doi:10.1371/journal.pone.0067221)
Supplement: Table S3 — Ribosomal RNA gene sequences less than 200 nt in length (could not be submitted to NCBI) from V5. [“n” indicates information not specified in the NCBI GenBank database.]. (PDF) [file pone.0067221.s008.pdf]

Table S3. Ribosomal RNA gene sequences less than 200 nt in length (could not be submitted to NCBI) from V5. ["n" indicates information not specified in the NCBI GenBank database.]

454 Sequence ID	Q length	Q start	Q end	e-value	%-ident	%-sim	GI number	Domain	Phylum	Family	Genus / Species
GKJWQY101AUK82	258	5	168	4E-77	99%	99%	269979854	Bacteria	Actinobacteria	Actinomycetaceae	uncultured Actinomyces sp.
GKJWQY101AC1PI	228	5	174	2E-79	99%	99%	293629580	Bacteria	Actinobacteria	Microbacteriaceae	Klugiella sp. Cr8-25
GKJWQY101AKK09	64	5	58	1E-15	96%	96%	295345516	Bacteria	Actinobacteria	Micrococcaceae	Arthrobacter sp. enrichment culture clone _ENR1_9.1
GKJWQY101ALS2O	204	23	158	6E-64	100%	100%	295809764	Bacteria	Actinobacteria	Micrococcaceae	Arthrobacter sp. V2M1
GKJWQY101ATYDL	575	217	434	4E-75	91%	91%	241995735	Bacteria	Actinobacteria	Micrococcaceae	Kocuria sp. 10-4DEP
GKJWQY101AL2DJ	225	17	168	9E-73	100%	100%	158551986	Bacteria	Actinobacteria	Micrococcaceae	Micrococcus sp. SAWW3
GKJWQY101AGL3S	97	23	83	5E-21	98%	98%	170674451	Bacteria	Actinobacteria	n	Micrococcineae bacterium HM06-11
GKJWQY101A0PVQ	184	17	139	9E-57	100%	100%	295410271	Bacteria	Actinobacteria	Nocardiaceae	Rhodococcus sp. WTZ-R2
GKJWQY101BB1KL	302	110	220	8E-40	95%	95%	289470718	Bacteria	Actinobacteria	Nocardioidaceae	Kribbella sp. PIP 158
GKJWQY101APNNM	180	17	159	7E-68	100%	100%	110238724	Bacteria	Actinobacteria	Propionibacteriaceae	Propionibacterium sp. SV442
GKJWQY101A02B3	147	50	103	7E-17	98%	98%	2226289	Bacteria	Actinobacteria	Pseudonocardiaceae	Thermobispora bispora
GKJWQY101AUK0Q	186	18	141	1E-55	99%	99%	270048059	Bacteria	Actinobacteria	Sanguibacteraceae	Sanguibacter sp. ljh-8
GKJWQY101BSJK8	127	5	57	3E-15	96%	96%	219846414	Bacteria	Actinobacteria	Thermomonosporaceae	Actinocorallia libanotica
GKJWQY101ALSG0	260	5	196	8E-89	98%	98%	284930165	Bacteria	Actinobacteria	Bifidobacteriaceae	Bifidobacterium gallinarum
GKJWQY101A70F2	221	5	190	2E-78	96%	96%	237930451	Bacteria	Actinobacteria	Bifidobacteriaceae	uncultured Bifidobacterium sp.
GKJWQY101AHOH5	180	4	127	2E-23	85%	85%	151384647	Bacteria	Actinobacteria	Coriobacteriaceae	uncultured Olsenella sp.
GKJWQY101BJRK5	197	11	152	1E-56	96%	96%	1418399	Bacteria	Actinobacteria	n	bacterium WE2
GKJWQY101AJU56	147	1	101	6E-38	96%	96%	24210873	Bacteria	Actinobacteria	n	uncultured actinobacterium
GKJWQY101BXZWQ	126	21	79	1E-19	98%	98%	157837222	Bacteria	Actinobacteria	n	uncultured actinobacterium
GKJWQY101BUECR	185	5	150	1E-60	96%	96%	293618134	Bacteria	Actinobacteria	n	uncultured actinobacterium
GKJWQY101A8CYD	240	43	178	1E-61	99%	99%	293618152	Bacteria	Actinobacteria	n	uncultured actinobacterium
GKJWQY101AOYOC	152	4	94	2E-32	96%	96%	48728183	Bacteria	Actinobacteria	Frankiaceae	uncultured Frankia sp.
GKJWQY101APUX2	555	16	549	3E-170	88%	88%	37545001	Bacteria	Actinobacteria	Corynebacteriaceae	Corynebacterium
GKJWQY101A4BGJ	176	21	103	3E-22	92%	92%	7208098	Bacteria	Actinobacteria	Thermomonosporaceae	Actinomadura echinospora
GKJWQY101ATSX1	563	139	555	0	96%	96%	208690660	Bacteria	Bacteroidetes	n	uncultured Bacteroidales bacterium
GKJWQY101BNJE7	147	4	85	2E-27	95%	95%	217337428	Bacteria	Bacteroidetes	n	uncultured Bacteroidales bacterium
GKJWQY101BW1AP	192	2	121	2E-53	99%	99%	217337429	Bacteria	Bacteroidetes	n	uncultured Bacteroidales bacterium
GKJWQY101A82OQ	179	5	122	2E-47	97%	97%	162849118	Bacteria	Bacteroidetes	Porphyromonadaceae	uncultured Porphyromonas sp.
GKJWQY101AAUNK	212	18	168	6E-69	99%	99%	186926082	Bacteria	Bacteroidetes	Flavobacteriaceae	uncultured Flavobacterium sp.
GKJWQY101AHUQL	210	5	158	3E-72	99%	99%	184132972	Bacteria	Bacteroidetes	Sphingobacteriaceae	Pedobacter steynii
GKJWQY101BAP0G	236	4	198	7E-89	97%	97%	284431811	Bacteria	Bacteroidetes	Sphingobacteriaceae	Sphingobacterium sp. 0-1
GKJWQY101BHQ5F	188	17	153	7E-48	93%	93%	154759415	Bacteria	Bacteroidetes	Flavobacteriaceae	Flavobacterium columnare
GKJWQY101AULNM	159	18	132	3E-46	97%	97%	154845376	Bacteria	Cyanobacteria	n	Gloeocapsa sp. CR_L16
GKJWQY101BSMW8	63	25	55	0.000003	100%	100%	119657303	Bacteria	Cyanobacteria	n	uncultured Microcystis sp.
GKJWQY101AY8U6	476	372	410	0.00001	95%	95%	124361561	Bacteria	Cyanobacteria	n	uncultured Thermosynechococcus sp.
GKJWQY101BYVWY	294	82	258	2E-71	95%	95%	46948081	Bacteria	Cyanobacteria	n	uncultured Antarctic cyanobacterium
GKJWQY101BPXL9	157	5	104	6E-43	99%	99%	67482522	Bacteria	Cyanobacteria	n	uncultured cyanobacterium
GKJWQY101A5GVE	188	21	156	3E-61	99%	99%	105990262	Bacteria	Cyanobacteria	n	uncultured cyanobacterium
GKJWQY101A349D	280	18	166	2E-64	97%	97%	105990476	Bacteria	Cyanobacteria	n	uncultured cyanobacterium
GKJWQY101AGISR	560	5	559	0	97%	97%	146429655	Bacteria	Cyanobacteria	n	uncultured cyanobacterium
GKJWQY101AJCIH	146	5	65	7E-17	95%	95%	156187252	Bacteria	Cyanobacteria	n	uncultured cyanobacterium
GKJWQY101ACXP4	94	1	80	1E-31	99%	99%	163962573	Bacteria	Cyanobacteria	n	uncultured cyanobacterium
GKJWQY101AV5XT	196	5	178	2E-83	99%	99%	189484695	Bacteria	Cyanobacteria	n	uncultured cyanobacterium
GKJWQY101A9ERV	113	18	47	0.00002	100%	100%	189484726	Bacteria	Cyanobacteria	n	uncultured cyanobacterium
GKJWQY101BVZ69	231	2	180	1E-76	97%	97%	213053918	Bacteria	Cyanobacteria	n	uncultured cyanobacterium
GKJWQY101AJE27	232	5	164	7E-74	99%	99%	213053926	Bacteria	Cyanobacteria	n	uncultured cyanobacterium
GKJWQY101BUG7V	198	2	155	3E-72	99%	99%	213053927	Bacteria	Cyanobacteria	n	uncultured cyanobacterium
GKJWQY101AGMF1	194	3	150	1E-70	100%	100%	213053932	Bacteria	Cyanobacteria	n	uncultured cyanobacterium
GKJWQY101BD8H5	190	5	146	3E-67	100%	100%	219883489	Bacteria	Cyanobacteria	n	uncultured cyanobacterium
GKJWQY101AR8AI	150	8	134	2E-57	99%	99%	225031951	Bacteria	Cyanobacteria	n	uncultured cyanobacterium
GKJWQY101A3SVV	213	17	169	1E-61	95%	95%	227072232	Bacteria	Cyanobacteria	n	uncultured cyanobacterium
GKJWQY101AT7RZ	160	9	127	2E-52	99%	99%	229563895	Bacteria	Cyanobacteria	n	uncultured cyanobacterium
GKJWQY101A5DQZ	136	17	106	1E-38	100%	100%	229563905	Bacteria	Cyanobacteria	n	uncultured cyanobacterium
GKJWQY101BHNRF	165	25	112	2E-37	100%	100%	253749514	Bacteria	Cyanobacteria	n	uncultured cyanobacterium

GKJWQY101BA64N	229	27	99	2E-24	96%	96%	257782341	Bacteria	Cyanobacteria	n	uncultured cyanobacterium
GKJWQY101BKQNB	294	25	131	2E-31	91%	91%	261290032	Bacteria	Cyanobacteria	n	uncultured cyanobacterium
GKJWQY101BM0KB	120	5	63	2E-21	100%	100%	285015322	Bacteria	Cyanobacteria	n	uncultured cyanobacterium
GKJWQY101BQSM2	47	1	46	6E-15	100%	100%	225696274	Bacteria	Cyanobacteria	n	Uncultured Chroococcidiopsis sp.
GKJWQY101AGMV1	233	5	185	7E-89	100%	100%	18182395	Bacteria	Cyanobacteria	n	uncultured soil crust cyanobacterium
GKJWQY101AR5CJ	134	3	87	8E-36	100%	100%	45643565	Bacteria	Cyanobacteria	Microchaetaceae	Petalonema sp. ANT.LG2.8
GKJWQY101ALPMM	141	4	79	1E-19	92%	92%	183673428	Bacteria	Cyanobacteria	Nostocaceae	Trichormus naviculoides
GKJWQY101AB8TO	202	7	148	3E-62	98%	98%	24850290	Bacteria	Cyanobacteria	Rivulariaceae	Calothrix sp. CCMEE 5085
GKJWQY101BPQ7H	169	3	123	1E-55	100%	100%	143635043	Bacteria	Cyanobacteria	Scytonemataceae	Brasilonema terrestre
GKJWQY101BEX4H	218	22	197	4E-81	98%	98%	167508113	Bacteria	Cyanobacteria	n	Geitlerinema sp. CCALA 138
GKJWQY101BA9JR	241	5	169	6E-75	98%	98%	262477864	Bacteria	Cyanobacteria	n	Leptolyngbya sp. 22.A
GKJWQY101BVSZA	179	23	110	2E-18	88%	88%	57996802	Bacteria	Cyanobacteria	n	Microcoleus steenstrupii
GKJWQY101BCKFY	192	5	160	2E-73	99%	99%	121308615	Bacteria	Cyanobacteria	n	Oscillatoria lutea
GKJWQY101ALVLN	199	1	143	4E-66	99%	99%	291603790	Bacteria	Cyanobacteria	n	Oscillatoria margaritifera
GKJWQY101BYF48	148	4	40	5E-09	100%	100%	226844847	Bacteria	Cyanobacteria	n	Oscillatoria sp. MMG-2
GKJWQY101BBGQI	149	8	132	3E-55	98%	98%	258547392	Bacteria	Cyanobacteria	n	Phormidium autumnale
GKJWQY101BE15A	212	60	161	5E-45	100%	100%	258547396	Bacteria	Cyanobacteria	n	Phormidium autumnale
GKJWQY101BAT30	217	1	145	1E-66	99%	99%	260595431	Bacteria	Cyanobacteria	n	Phormidium autumnale
GKJWQY101BI23D	490	265	459	1E-34	82%	82%	45643541	Bacteria	Cyanobacteria	n	Phormidium pseudopristleyi
GKJWQY101BQZC6	125	23	93	5E-28	100%	100%	157384116	Bacteria	Cyanobacteria	n	uncultured Planktothricoides sp.
GKJWQY101AY7ZD	107	5	50	0.000002	90%	90%	225696182	Bacteria	Cyanobacteria	n	uncultured Chroococcidiopsis sp.
GKJWQY101ARSH3	168	5	80	1E-30	100%	100%	225696183	Bacteria	Cyanobacteria	n	uncultured Chroococcidiopsis sp.
GKJWQY101A09D3	176	18	142	2E-47	95%	95%	225696254	Bacteria	Cyanobacteria	n	uncultured Chroococcidiopsis sp.
GKJWQY101A4KSE	138	49	108	7E-22	100%	100%	225696281	Bacteria	Cyanobacteria	n	uncultured Chroococcidiopsis sp.
GKJWQY101BTRUZ	140	17	80	9E-21	97%	97%	225696289	Bacteria	Cyanobacteria	n	uncultured Chroococcidiopsis sp.
GKJWQY101BUHI6	294	5	84	1E-32	100%	100%	225696290	Bacteria	Cyanobacteria	n	uncultured Chroococcidiopsis sp.
GKJWQY101AEUTS_2	40	5	40	2E-10	100%	100%	358680771	Bacteria	Cyanobacteria	Arthrospira	Arthrospira platensis
GKJWQY101AZK7G	178	1	137	2E-43	90%	90%	46409885	Bacteria	Cyanobacteria	Nostocaceae	Anabaena sp. PCC 9109
GKJWQY101A6FV9	248	5	197	3E-68	91%	91%	46409888	Bacteria	Cyanobacteria	Nostocaceae	Nodularia harveyana
GKJWQY101BA9Y4	154	18	123	2E-47	100%	100%	46409897	Bacteria	Cyanobacteria	n	Lyngbya aestuarii
GKJWQY101BDPUJ	291	5	241	5E-106	97%	97%	284055538	Bacteria	Deinococcus-Thermus	Deinococcaceae	Deinococcus radiodurans
GKJWQY101AXBQG	175	17	128	3E-41	96%	96%	145567431	Bacteria	Fibrobacteres	Fibrobacteraceae	uncultured Fibrobacter sp.
GKJWQY101BM1GN	71	6	55	3E-11	94%	94%	294337929	Bacteria	Firmicutes	Bacillaceae	Bacillus clausii
GKJWQY101ARL9J	221	5	189	2E-89	99%	99%	294337906	Bacteria	Firmicutes	Bacillaceae	Bacillus sp. 3LF 24T
GKJWQY101BWSJU	549	201	547	2E-173	99%	99%	15042017	Bacteria	Firmicutes	Bacillaceae	Bacillus sp. NCIB 12289
GKJWQY101BURPU	269	15	99	2E-35	100%	100%	134290402	Bacteria	Firmicutes	Bacillaceae	Geobacillus kaustophilus
GKJWQY101BPODQ	120	18	79	4E-23	100%	100%	283131629	Bacteria	Firmicutes	Bacillaceae	uncultured Amphibacillus sp.
GKJWQY101BQ5E9	450	86	414	3E-135	94%	94%	284428972	Bacteria	Firmicutes	Bacillaceae	uncultured Bacillus sp.
GKJWQY101BR4I0	145	2	100	2E-43	100%	100%	292485814	Bacteria	Firmicutes	Planococcaceae	uncultured Planococcus sp.
GKJWQY101AWLH0	224	81	143	1E-21	98%	98%	292485818	Bacteria	Firmicutes	Planococcaceae	uncultured Planomicrobium sp.
GKJWQY101B0MXN	222	5	190	2E-83	97%	97%	254952546	Bacteria	Firmicutes	Enterococcaceae	Enterococcus columbae
GKJWQY101AAXDV	137	11	93	2E-31	98%	98%	50080727	Bacteria	Firmicutes	Enterococcaceae	Tetragenococcus doogicus
GKJWQY101BDM0N	186	5	140	4E-60	99%	99%	189345361	Bacteria	Firmicutes	Lactobacillaceae	Lactobacillus delbrueckii
GKJWQY101BBYPN	167	5	114	1E-49	100%	100%	285201718	Bacteria	Firmicutes	Lactobacillaceae	Lactobacillus rhamnosus
GKJWQY101AAULG	351	18	112	3E-39	99%	99%	285802968	Bacteria	Firmicutes	Lactobacillaceae	Lactobacillus sp. oral taxon 461
GKJWQY101A74IZ	194	5	136	1E-61	100%	100%	159032974	Bacteria	Firmicutes	Streptococcaceae	Lactococcus lactis
GKJWQY101ARYR6	239	24	187	2E-79	100%	100%	295002587	Bacteria	Firmicutes	Streptococcaceae	Streptococcus australis
GKJWQY101ADMQF	130	30	88	1E-19	98%	98%	24474990	Bacteria	Firmicutes	Streptococcaceae	Streptococcus constellatus
GKJWQY101BRH69	166	5	134	1E-54	97%	97%	290759882	Bacteria	Firmicutes	Streptococcaceae	Streptococcus cristatus
GKJWQY101AD5O6	203	15	158	3E-62	97%	97%	285184215	Bacteria	Firmicutes	Streptococcaceae	Streptococcus intermedius
GKJWQY101BRELC	206	5	107	2E-39	96%	96%	110432033	Bacteria	Firmicutes	Streptococcaceae	Streptococcus mutans
GKJWQY101A0A4K	169	24	137	1E-45	97%	97%	285205054	Bacteria	Firmicutes	Streptococcaceae	Streptococcus sp. oral taxon E07
GKJWQY101BCLHF	176	3	128	2E-58	100%	100%	60501147	Bacteria	Firmicutes	Streptococcaceae	uncultured Streptococcus sp.
GKJWQY101BJ018	220	5	164	3E-77	100%	100%	295322329	Bacteria	Firmicutes	Streptococcaceae	uncultured Streptococcus sp.
GKJWQY101BYGNW	193	5	119	1E-50	99%	99%	154189328	Bacteria	Firmicutes	n	uncultured Bacilli bacterium
GKJWQY101BNMTI	183	5	114	7E-33	91%	91%	254841686	Bacteria	Firmicutes	Clostridiaceae	Clostridium perfringens
GKJWQY101BGC3W	113	23	82	5E-22	100%	100%	295656276	Bacteria	Firmicutes	n	Firmicutes bacterium enrichment culture clone VNBB003
GKJWQY101BPAK0	220	15	175	2E-59	93%	93%	118135758	Bacteria	Firmicutes	n	uncultured Firmicutes bacterium
GKJWQY101B1XT2	490	5	353	3E-180	100%	100%	146430648	Bacteria	Firmicutes	n	uncultured Firmicutes bacterium

GKJWQY101ADWDT	111	18	78	3E-19	97%	97%	291330751	Bacteria	Firmicutes	n	uncultured Firmicutes bacterium
GKJWQY101BAJPW	199	4	146	8E-68	100%	100%	291332909	Bacteria	Firmicutes	n	uncultured Firmicutes bacterium
GKJWQY101BQAJ9	118	18	87	2E-27	100%	100%	295656572	Bacteria	Firmicutes	n	uncultured Firmicutes bacterium
GKJWQY101ANJ0H	213	3	164	4E-71	98%	98%	27819245	Bacteria	Firmicutes	n	uncultured low G+C Gram-positive bacterium
GKJWQY101BGFER	163	4	155	3E-71	99%	99%	285802763	Bacteria	Firmicutes	Veillonellaceae	Selenomonas sp. oral taxon 442
GKJWQY101AKQLO	205	18	148	6E-54	96%	96%	285166090	Bacteria	Firmicutes	Veillonellaceae	Selenomonas sputigena
GKJWQY101ACH6F	209	18	153	3E-62	99%	99%	285166317	Bacteria	Firmicutes	Veillonellaceae	Selenomonas sputigena
GKJWQY101BZOS5	330	50	211	2E-51	90%	90%	295083276	Bacteria	Firmicutes	Bacillaceae	Bacillus licheniformis
GKJWQY101AY4WN_2	69	1	69	3E-17	92%	92%	373943374	Bacteria	Firmicutes	Clostridiaceae	Clostridium
GKJWQY101BCYFO_2	175	1	175	4E-52	89%	89%	345283481	Bacteria	Firmicutes	Lactobacillaceae	Lactobacillus
GKJWQY101BXNXM_2	209	1	209	1E-86	96%	96%	339277069	Bacteria	Firmicutes	Streptococcaceae	Streptococcus
GKJWQY101A4X2Z	215	5	184	1E-85	99%	99%	16508084	Bacteria	n	n	uncultured bacterium
GKJWQY101AVTAG	302	5	176	9E-84	100%	100%	19908565	Bacteria	n	n	uncultured bacterium
GKJWQY101A7KKU	167	5	105	2E-42	99%	99%	37654631	Bacteria	n	n	uncultured bacterium
GKJWQY101A6OXT	139	15	102	1E-24	91%	91%	54695037	Bacteria	n	n	uncultured bacterium
GKJWQY101AW45M	226	5	194	2E-64	91%	91%	63087409	Bacteria	n	n	uncultured bacterium
GKJWQY101B2Z7C	207	5	174	3E-72	96%	96%	68004476	Bacteria	n	n	uncultured bacterium
GKJWQY101BN5ON	301	109	219	2E-41	95%	95%	71089366	Bacteria	n	n	uncultured bacterium
GKJWQY101AQAPU	232	5	185	2E-44	86%	86%	71089410	Bacteria	n	n	uncultured bacterium
GKJWQY101AGG0L	404	193	371	2E-82	98%	98%	71739177	Bacteria	n	n	uncultured bacterium
GKJWQY101ALMD0	274	17	162	2E-69	100%	100%	74038724	Bacteria	n	n	uncultured bacterium
GKJWQY101BRO0X	211	5	63	5E-15	93%	93%	80978525	Bacteria	n	n	uncultured bacterium
GKJWQY101A114T	131	3	84	4E-34	100%	100%	109143142	Bacteria	n	n	uncultured bacterium
GKJWQY101B2BO0	186	6	138	1E-60	99%	99%	109145063	Bacteria	n	n	uncultured bacterium
GKJWQY101AD5PI	150	3	97	3E-41	100%	100%	109146140	Bacteria	n	n	uncultured bacterium
GKJWQY101A3O5F	275	100	259	1E-67	96%	96%	110435279	Bacteria	n	n	uncultured bacterium
GKJWQY101BK3NT	188	3	134	9E-62	100%	100%	110441026	Bacteria	n	n	uncultured bacterium
GKJWQY101A4T8R	171	15	113	2E-38	97%	97%	110442172	Bacteria	n	n	uncultured bacterium
GKJWQY101BZXHF	161	19	98	3E-31	99%	99%	110445958	Bacteria	n	n	uncultured bacterium
GKJWQY101ALSLZ	399	129	352	7E-111	100%	100%	110449562	Bacteria	n	n	uncultured bacterium
GKJWQY101AAHX7	170	62	142	9E-32	99%	99%	110450750	Bacteria	n	n	uncultured bacterium
GKJWQY101AVQKA	241	14	196	2E-54	89%	89%	117572568	Bacteria	n	n	uncultured bacterium
GKJWQY101BGPB7	172	7	170	5E-69	96%	96%	126115235	Bacteria	n	n	uncultured bacterium
GKJWQY101A4OEB	530	375	522	2E-37	87%	87%	126674327	Bacteria	n	n	uncultured bacterium
GKJWQY101AKX7M	176	5	126	7E-53	98%	98%	148249503	Bacteria	n	n	uncultured bacterium
GKJWQY101BYO28	149	24	51	0.0005	100%	100%	151610746	Bacteria	n	n	uncultured bacterium
GKJWQY101BL1PV	87	4	42	0.0000004	95%	95%	156522979	Bacteria	n	n	uncultured bacterium
GKJWQY101AVV30	252	5	177	1E-82	99%	99%	157926754	Bacteria	n	n	uncultured bacterium
GKJWQY101BIS50	108	5	51	4E-13	98%	98%	158148312	Bacteria	n	n	uncultured bacterium
GKJWQY101ACR1Y	111	17	79	5E-22	98%	98%	160922959	Bacteria	n	n	uncultured bacterium
GKJWQY101BHT31	160	3	129	1E-55	98%	98%	160922967	Bacteria	n	n	uncultured bacterium
GKJWQY101BDAHB	214	28	161	8E-63	100%	100%	160922973	Bacteria	n	n	uncultured bacterium
GKJWQY101B0MIJ	173	5	131	1E-55	98%	98%	161085546	Bacteria	n	n	uncultured bacterium
GKJWQY101ADTLC	352	18	113	4E-38	98%	98%	164460342	Bacteria	n	n	uncultured bacterium
GKJWQY101BV86J	228	5	169	5E-65	95%	95%	169130236	Bacteria	n	n	uncultured bacterium
GKJWQY101BMOCO	135	5	84	5E-33	100%	100%	169132834	Bacteria	n	n	uncultured bacterium
GKJWQY101AQHB3	251	24	193	5E-81	99%	99%	169266077	Bacteria	n	n	uncultured bacterium
GKJWQY101BDMF0	229	19	194	2E-45	87%	87%	169270486	Bacteria	n	n	uncultured bacterium
GKJWQY101A7UQ6	95	6	62	2E-14	93%	93%	169270865	Bacteria	n	n	uncultured bacterium
GKJWQY101A7X5W	190	4	125	3E-46	95%	95%	169276777	Bacteria	n	n	uncultured bacterium
GKJWQY101BJ2CQ	133	42	101	1E-18	97%	97%	169281343	Bacteria	n	n	uncultured bacterium
GKJWQY101BYP36	218	5	173	4E-66	94%	94%	169285764	Bacteria	n	n	uncultured bacterium
GKJWQY101A0A9F	202	24	177	1E-55	93%	93%	169286886	Bacteria	n	n	uncultured bacterium
GKJWQY101AYP07	478	200	417	2E-101	98%	98%	169288388	Bacteria	n	n	uncultured bacterium
GKJWQY101ACHNZ	216	5	147	8E-68	100%	100%	169908324	Bacteria	n	n	uncultured bacterium
GKJWQY101AVWFQ	148	5	92	9E-36	99%	99%	186703460	Bacteria	n	n	uncultured bacterium
GKJWQY101AWIA1	153	24	97	3E-26	97%	97%	187424100	Bacteria	n	n	uncultured bacterium
GKJWQY101BZBZK	255	16	94	8E-29	97%	97%	187472598	Bacteria	n	n	uncultured bacterium
GKJWQY101BO4CQ	188	2	132	4E-55	97%	97%	187967876	Bacteria	n	n	uncultured bacterium
GKJWQY101BQFTF	133	17	85	6E-27	100%	100%	189017027	Bacteria	n	n	uncultured bacterium

GKJWQY101AW4ZG	229	5	100	6E-40	99%	99%	189305490	Bacteria	n	n	uncultured bacterium
GKJWQY101BTFQ8	263	7	131	3E-53	98%	98%	190703341	Bacteria	n	n	uncultured bacterium
GKJWQY101AT5HI	193	18	149	1E-46	93%	93%	192787190	Bacteria	n	n	uncultured bacterium
GKJWQY101BYLLM	216	5	162	2E-74	99%	99%	192975671	Bacteria	n	n	uncultured bacterium
GKJWQY101APW1M	234	6	174	1E-76	98%	98%	192976646	Bacteria	n	n	uncultured bacterium
GKJWQY101BCCB7	96	18	65	2E-15	100%	100%	192979799	Bacteria	n	n	uncultured bacterium
GKJWQY101BLGRG	233	14	189	2E-74	96%	96%	192984058	Bacteria	n	n	uncultured bacterium
GKJWQY101A68XQ	161	2	113	1E-50	100%	100%	192985634	Bacteria	n	n	uncultured bacterium
GKJWQY101AYCI9	190	5	133	2E-54	98%	98%	192988614	Bacteria	n	n	uncultured bacterium
GKJWQY101A0EAO	184	4	142	7E-58	96%	96%	192989009	Bacteria	n	n	uncultured bacterium
GKJWQY101AEMB4	196	10	102	1E-30	94%	94%	192989690	Bacteria	n	n	uncultured bacterium
GKJWQY101BEIXQ	337	22	203	6E-86	99%	99%	194139977	Bacteria	n	n	uncultured bacterium
GKJWQY101AFAG6	220	42	165	1E-55	99%	99%	196050989	Bacteria	n	n	uncultured bacterium
GKJWQY101BAP6K	228	3	180	3E-87	100%	100%	197346063	Bacteria	n	n	uncultured bacterium
GKJWQY101AEOVS	227	3	147	7E-69	100%	100%	197346834	Bacteria	n	n	uncultured bacterium
GKJWQY101B2WLT	208	21	179	6E-69	97%	97%	197347839	Bacteria	n	n	uncultured bacterium
GKJWQY101AC3X6	193	5	125	2E-48	97%	97%	197350746	Bacteria	n	n	uncultured bacterium
GKJWQY101BEOTH	220	4	168	1E-56	92%	92%	197358937	Bacteria	n	n	uncultured bacterium
GKJWQY101BTB05	73	18	50	0.0000003	100%	100%	198403818	Bacteria	n	n	uncultured bacterium
GKJWQY101BP7BQ	394	168	334	5E-63	95%	95%	198403887	Bacteria	n	n	uncultured bacterium
GKJWQY101A3PZM	120	25	85	1E-14	93%	93%	206598970	Bacteria	n	n	uncultured bacterium
GKJWQY101BBE60	189	24	149	4E-55	98%	98%	210076514	Bacteria	n	n	uncultured bacterium
GKJWQY101AP30O	219	5	174	2E-79	99%	99%	214022832	Bacteria	n	n	uncultured bacterium
GKJWQY101ATCER	216	18	202	6E-89	99%	99%	214025732	Bacteria	n	n	uncultured bacterium
GKJWQY101AZILO	235	18	149	1E-61	100%	100%	215262245	Bacteria	n	n	uncultured bacterium
GKJWQY101ATF4E	246	18	147	2E-60	100%	100%	215269474	Bacteria	n	n	uncultured bacterium
GKJWQY101AISXB	219	16	174	5E-75	99%	99%	215270163	Bacteria	n	n	uncultured bacterium
GKJWQY101A1VO9	144	16	66	7E-17	100%	100%	215271334	Bacteria	n	n	uncultured bacterium
GKJWQY101AQV1K	224	17	180	9E-58	92%	92%	217323656	Bacteria	n	n	uncultured bacterium
GKJWQY101A2QDJ	112	4	68	4E-23	98%	98%	217417638	Bacteria	n	n	uncultured bacterium
GKJWQY101BZW8R	188	6	90	2E-29	96%	96%	218411184	Bacteria	n	n	uncultured bacterium
GKJWQY101BAP4Z	144	1	102	3E-45	100%	100%	220980230	Bacteria	n	n	uncultured bacterium
GKJWQY101AF6N4	160	17	133	2E-33	91%	91%	222101801	Bacteria	n	n	uncultured bacterium
GKJWQY101BU7MO	133	18	80	2E-21	98%	98%	223675948	Bacteria	n	n	uncultured bacterium
GKJWQY101AVNTG	205	5	152	6E-69	99%	99%	223676026	Bacteria	n	n	uncultured bacterium
GKJWQY101AARCP	213	90	178	9E-38	100%	100%	223676405	Bacteria	n	n	uncultured bacterium
GKJWQY101AZYPW	181	21	150	3E-47	94%	94%	223677000	Bacteria	n	n	uncultured bacterium
GKJWQY101ACQYN	229	5	185	7E-89	100%	100%	223677158	Bacteria	n	n	uncultured bacterium
GKJWQY101A1FP8	170	5	118	8E-52	100%	100%	223677225	Bacteria	n	n	uncultured bacterium
GKJWQY101AXZSR	623	341	586	7E-98	94%	94%	223679670	Bacteria	n	n	uncultured bacterium
GKJWQY101AG521	203	5	159	8E-73	99%	99%	223679768	Bacteria	n	n	uncultured bacterium
GKJWQY101BPNVT	86	4	41	6E-10	100%	100%	223680429	Bacteria	n	n	uncultured bacterium
GKJWQY101BPZTP	212	24	167	2E-64	99%	99%	223681084	Bacteria	n	n	uncultured bacterium
GKJWQY101BSD63	168	17	130	2E-32	91%	91%	223681760	Bacteria	n	n	uncultured bacterium
GKJWQY101AF98O	498	293	451	1E-65	96%	96%	223684843	Bacteria	n	n	uncultured bacterium
GKJWQY101AQ46D	201	18	102	1E-35	100%	100%	223689665	Bacteria	n	n	uncultured bacterium
GKJWQY101BSWK8	103	18	97	6E-26	95%	95%	223696363	Bacteria	n	n	uncultured bacterium
GKJWQY101BWN63	212	5	179	1E-85	100%	100%	223696670	Bacteria	n	n	uncultured bacterium
GKJWQY101BF2X2	209	4	156	2E-58	94%	94%	223696681	Bacteria	n	n	uncultured bacterium
GKJWQY101BJIPN	129	5	58	1E-18	100%	100%	223696712	Bacteria	n	n	uncultured bacterium
GKJWQY101BINK2	216	5	173	3E-82	100%	100%	223987560	Bacteria	n	n	uncultured bacterium
GKJWQY101B1686	179	5	136	9E-62	100%	100%	224569286	Bacteria	n	n	uncultured bacterium
GKJWQY101BBI23	224	5	186	2E-89	100%	100%	224569396	Bacteria	n	n	uncultured bacterium
GKJWQY101AIJSJ	280	18	170	2E-69	99%	99%	224569450	Bacteria	n	n	uncultured bacterium
GKJWQY101ACUTJ	176	1	144	3E-66	99%	99%	224570010	Bacteria	n	n	uncultured bacterium
GKJWQY101BMIOK	206	5	161	6E-74	99%	99%	225302498	Bacteria	n	n	uncultured bacterium
GKJWQY101A8GVM	110	4	65	4E-23	100%	100%	225332552	Bacteria	n	n	uncultured bacterium
GKJWQY101AJOGY	450	154	395	2E-112	98%	98%	225337346	Bacteria	n	n	uncultured bacterium
GKJWQY101A94MM	132	17	100	5E-33	99%	99%	225382275	Bacteria	n	n	uncultured bacterium
GKJWQY101A97N8	114	5	65	7E-21	98%	98%	226446886	Bacteria	n	n	uncultured bacterium

GKJWQY101AQQE2	184	69	137	4E-25	99%	99%	227937910	Bacteria	n	n		uncultured bacterium
GKJWQY101A47QD	214	24	169	8E-58	95%	95%	229428878	Bacteria	n	n		uncultured bacterium
GKJWQY101B08E3	181	16	149	1E-50	95%	95%	229428912	Bacteria	n	n		uncultured bacterium
GKJWQY101AJVH3	191	18	174	6E-74	99%	99%	237774919	Bacteria	n	n		uncultured bacterium
GKJWQY101BKWJX	102	2	52	5E-17	100%	100%	238274022	Bacteria	n	n		uncultured bacterium
GKJWQY101BB15S	206	5	152	6E-69	99%	99%	238275953	Bacteria	n	n		uncultured bacterium
GKJWQY101BPQIT	176	3	118	7E-53	100%	100%	238286775	Bacteria	n	n		uncultured bacterium
GKJWQY101AX4CO	219	24	192	3E-82	100%	100%	238296004	Bacteria	n	n		uncultured bacterium
GKJWQY101AFUHT	181	5	120	7E-53	100%	100%	238302943	Bacteria	n	n		uncultured bacterium
GKJWQY101B0FZ0	182	5	137	2E-62	100%	100%	238303545	Bacteria	n	n		uncultured bacterium
GKJWQY101ANDAP	251	24	190	6E-75	98%	98%	238306185	Bacteria	n	n		uncultured bacterium
GKJWQY101AG7ZP	222	3	172	4E-81	99%	99%	238306254	Bacteria	n	n		uncultured bacterium
GKJWQY101ARRBE	234	4	202	7E-99	100%	100%	238309466	Bacteria	n	n		uncultured bacterium
GKJWQY101BPA56	228	3	174	1E-81	99%	99%	238309474	Bacteria	n	n		uncultured bacterium
GKJWQY101BNTDY	263	5	202	1E-96	99%	99%	238310689	Bacteria	n	n		uncultured bacterium
GKJWQY101AELLV	368	5	120	2E-47	97%	97%	238313072	Bacteria	n	n		uncultured bacterium
GKJWQY101BME63	269	5	189	4E-82	97%	97%	238313611	Bacteria	n	n		uncultured bacterium
GKJWQY101BQWYM	200	1	140	2E-64	99%	99%	238315007	Bacteria	n	n		uncultured bacterium
GKJWQY101AFK5L	220	5	180	4E-86	100%	100%	238319366	Bacteria	n	n		uncultured bacterium
GKJWQY101BVCL6	201	23	156	1E-60	99%	99%	238324477	Bacteria	n	n		uncultured bacterium
GKJWQY101ABFSX	463	196	431	2E-107	97%	97%	238327948	Bacteria	n	n		uncultured bacterium
GKJWQY101B0QT6	171	5	89	4E-20	91%	91%	238331528	Bacteria	n	n		uncultured bacterium
GKJWQY101AL5T9	227	14	202	1E-86	98%	98%	238335486	Bacteria	n	n		uncultured bacterium
GKJWQY101BIA0Y	180	5	129	4E-55	98%	98%	238336486	Bacteria	n	n		uncultured bacterium
GKJWQY101BND1Y	186	4	154	3E-72	100%	100%	238340166	Bacteria	n	n		uncultured bacterium
GKJWQY101A9YVS	229	45	183	3E-62	99%	99%	238341273	Bacteria	n	n		uncultured bacterium
GKJWQY101BFJXK	114	3	84	3E-34	100%	100%	238347940	Bacteria	n	n		uncultured bacterium
GKJWQY101A9BQW	162	5	116	1E-50	100%	100%	238349878	Bacteria	n	n		uncultured bacterium
GKJWQY101AG5ZO	231	5	182	3E-48	88%	88%	238351695	Bacteria	n	n		uncultured bacterium
GKJWQY101A2NQY	210	5	164	6E-74	99%	99%	238352312	Bacteria	n	n		uncultured bacterium
GKJWQY101AELIY	202	10	103	6E-39	99%	99%	238352426	Bacteria	n	n		uncultured bacterium
GKJWQY101A6YZN	180	2	123	1E-54	99%	99%	238352568	Bacteria	n	n		uncultured bacterium
GKJWQY101BSXH9	574	147	574	4E-160	91%	91%	238415763	Bacteria	n	n		uncultured bacterium
GKJWQY101A6U0N	178	21	133	3E-51	100%	100%	238587419	Bacteria	n	n		uncultured bacterium
GKJWQY101BURBQ	202	4	157	5E-70	99%	99%	239837088	Bacteria	n	n		uncultured bacterium
GKJWQY101AUZ7K	232	18	185	5E-80	99%	99%	239837341	Bacteria	n	n		uncultured bacterium
GKJWQY101BFQ9T	171	2	113	1E-50	100%	100%	240001746	Bacteria	n	n		uncultured bacterium
GKJWQY101AZUYL	129	17	89	4E-29	100%	100%	247891912	Bacteria	n	n		uncultured bacterium
GKJWQY101B09JV	123	17	91	6E-27	97%	97%	253825750	Bacteria	n	n		uncultured bacterium
GKJWQY101BUXPV	140	18	109	1E-39	100%	100%	254547427	Bacteria	n	n		uncultured bacterium
GKJWQY101BE5CL	181	7	131	5E-49	96%	96%	254771233	Bacteria	n	n		uncultured bacterium
GKJWQY101BWU6P	603	36	63	0.002	100%	100%	254971702	Bacteria	n	n		uncultured bacterium
GKJWQY101BHUKI	229	5	184	5E-80	97%	97%	255043921	Bacteria	n	n		uncultured bacterium
GKJWQY101BRBK4	185	2	119	3E-52	99%	99%	255044082	Bacteria	n	n		uncultured bacterium
GKJWQY101A55O7	145	23	105	5E-33	99%	99%	255339758	Bacteria	n	n		uncultured bacterium
GKJWQY101AD8U0	274	3	185	6E-90	100%	100%	255339764	Bacteria	n	n		uncultured bacterium
GKJWQY101ANPVF	190	24	158	9E-57	97%	97%	256592689	Bacteria	n	n		uncultured bacterium
GKJWQY101BBIYS	87	18	60	2E-10	98%	98%	256681321	Bacteria	n	n		uncultured bacterium
GKJWQY101AYIQ0	173	5	122	1E-49	98%	98%	257131081	Bacteria	n	n		uncultured bacterium
GKJWQY101B1L3S	133	3	65	1E-14	92%	92%	258548056	Bacteria	n	n		uncultured bacterium
GKJWQY101AWV2U	233	5	201	9E-93	98%	98%	258550960	Bacteria	n	n		uncultured bacterium
GKJWQY101APOW0	55	2	43	2E-12	100%	100%	258551205	Bacteria	n	n		uncultured bacterium
GKJWQY101BX81T	688	5	457	0	96%	96%	258680101	Bacteria	n	n		uncultured bacterium
GKJWQY101AVW0Q	378	18	335	1E-147	97%	97%	258680407	Bacteria	n	n		uncultured bacterium
GKJWQY101AOP20	284	5	210	1E-92	97%	97%	258680950	Bacteria	n	n		uncultured bacterium
GKJWQY101AEFC7	538	5	476	0	93%	93%	258681941	Bacteria	n	n		uncultured bacterium
GKJWQY101ADZOY	346	17	312	6E-146	99%	99%	258681993	Bacteria	n	n		uncultured bacterium
GKJWQY101ACRBS	347	7	260	2E-115	97%	97%	258682090	Bacteria	n	n		uncultured bacterium
GKJWQY101BPZYR	222	22	188	4E-51	89%	89%	258682100	Bacteria	n	n		uncultured bacterium
GKJWQY101AI27W	451	8	418	6E-142	89%	89%	258682119	Bacteria	n	n		uncultured bacterium

GKJWQY101BXXJY	285	17	241	7E-110	99%	99%	258682645	Bacteria	n	n	uncultured bacterium
GKJWQY101A3VNN	207	5	159	8E-73	99%	99%	258682725	Bacteria	n	n	uncultured bacterium
GKJWQY101AJO34	386	6	339	1E-148	96%	96%	258683264	Bacteria	n	n	uncultured bacterium
GKJWQY101B089A	543	26	541	0	97%	97%	258684587	Bacteria	n	n	uncultured bacterium
GKJWQY101B1U3Z	549	19	543	0	98%	98%	258684800	Bacteria	n	n	uncultured bacterium
GKJWQY101BMENF	559	20	547	0	96%	96%	258684963	Bacteria	n	n	uncultured bacterium
GKJWQY101ADSPO	151	24	106	2E-27	95%	95%	258685315	Bacteria	n	n	uncultured bacterium
GKJWQY101AWUL7	137	5	91	4E-34	98%	98%	258686152	Bacteria	n	n	uncultured bacterium
GKJWQY101BF5YU	522	21	425	0	96%	96%	258687396	Bacteria	n	n	uncultured bacterium
GKJWQY101A4682	528	107	472	9E-171	97%	97%	258687549	Bacteria	n	n	uncultured bacterium
GKJWQY101AJVQS	546	5	447	0	98%	98%	258687641	Bacteria	n	n	uncultured bacterium
GKJWQY101BNW25	426	5	245	8E-61	87%	87%	258687840	Bacteria	n	n	uncultured bacterium
GKJWQY101ADWNC	328	17	282	1E-132	99%	99%	258687963	Bacteria	n	n	uncultured bacterium
GKJWQY101AC6T3	447	25	368	4E-164	97%	97%	258688401	Bacteria	n	n	uncultured bacterium
GKJWQY101BAGMC	219	6	152	5E-70	100%	100%	258688531	Bacteria	n	n	uncultured bacterium
GKJWQY101BYR0S	412	5	378	1E-167	95%	95%	258688670	Bacteria	n	n	uncultured bacterium
GKJWQY101AIZ1T	226	18	192	1E-75	97%	97%	258689065	Bacteria	n	n	uncultured bacterium
GKJWQY101APBNE	557	10	538	0	89%	89%	258689086	Bacteria	n	n	uncultured bacterium
GKJWQY101BKH69	253	24	220	2E-89	97%	97%	258689185	Bacteria	n	n	uncultured bacterium
GKJWQY101ADEAR	164	4	65	6E-23	100%	100%	259604289	Bacteria	n	n	uncultured bacterium
GKJWQY101BXWG8	197	5	150	2E-58	96%	96%	259880044	Bacteria	n	n	uncultured bacterium
GKJWQY101BEEWO	134	17	102	2E-36	100%	100%	259880137	Bacteria	n	n	uncultured bacterium
GKJWQY101ACH03	164	4	119	6E-53	100%	100%	260108166	Bacteria	n	n	uncultured bacterium
GKJWQY101A1RXA	132	1	87	3E-35	99%	99%	260609497	Bacteria	n	n	uncultured bacterium
GKJWQY101BLGDQ	231	18	160	9E-68	100%	100%	260609898	Bacteria	n	n	uncultured bacterium
GKJWQY101AERMJ	210	14	157	1E-66	99%	99%	260609992	Bacteria	n	n	uncultured bacterium
GKJWQY101BY15Y	137	5	70	2E-21	97%	97%	260667055	Bacteria	n	n	uncultured bacterium
GKJWQY101BPHVX	143	5	99	4E-34	96%	96%	260667085	Bacteria	n	n	uncultured bacterium
GKJWQY101BKT0U	137	5	125	4E-49	97%	97%	261261872	Bacteria	n	n	uncultured bacterium
GKJWQY101AQVUZ	224	5	167	1E-75	99%	99%	261261913	Bacteria	n	n	uncultured bacterium
GKJWQY101AQWBK	90	24	63	2E-09	98%	98%	262174158	Bacteria	n	n	uncultured bacterium
GKJWQY101AD88J	193	1	134	3E-61	99%	99%	262174241	Bacteria	n	n	uncultured bacterium
GKJWQY101BRBKI	141	4	98	1E-39	99%	99%	262528725	Bacteria	n	n	uncultured bacterium
GKJWQY101BFOCG	165	18	87	5E-24	97%	97%	269152240	Bacteria	n	n	uncultured bacterium
GKJWQY101ADP9P	317	7	160	8E-55	93%	93%	269162065	Bacteria	n	n	uncultured bacterium
GKJWQY101AA3KS	167	5	125	1E-55	100%	100%	269162927	Bacteria	n	n	uncultured bacterium
GKJWQY101A0UAE	86	1	79	4E-31	99%	99%	269855408	Bacteria	n	n	uncultured bacterium
GKJWQY101AVZ1L	428	182	375	1E-93	99%	99%	269973185	Bacteria	n	n	uncultured bacterium
GKJWQY101BV8M2	198	18	165	1E-70	100%	100%	270104615	Bacteria	n	n	uncultured bacterium
GKJWQY101A2DWK	155	5	102	6E-43	100%	100%	270267828	Bacteria	n	n	uncultured bacterium
GKJWQY101BOFMM	163	5	111	6E-48	100%	100%	281485327	Bacteria	n	n	uncultured bacterium
GKJWQY101A7BNU	123	5	80	7E-31	100%	100%	281488397	Bacteria	n	n	uncultured bacterium
GKJWQY101BLS2Q	356	5	201	8E-95	99%	99%	284158463	Bacteria	n	n	uncultured bacterium
GKJWQY101AU3MF	184	5	132	2E-59	100%	100%	284944629	Bacteria	n	n	uncultured bacterium
GKJWQY101A4RQ9	107	5	55	5E-17	100%	100%	285016583	Bacteria	n	n	uncultured bacterium
GKJWQY101ANHEG	160	9	57	8E-12	96%	96%	285960274	Bacteria	n	n	uncultured bacterium
GKJWQY101BVG3R	205	5	160	2E-63	96%	96%	285960440	Bacteria	n	n	uncultured bacterium
GKJWQY101ALVLH	206	22	156	2E-63	100%	100%	285960578	Bacteria	n	n	uncultured bacterium
GKJWQY101AIGIT	131	3	99	4E-39	98%	98%	288551175	Bacteria	n	n	uncultured bacterium
GKJWQY101AX0X2	121	22	75	1E-18	100%	100%	289185720	Bacteria	n	n	uncultured bacterium
GKJWQY101AX88J	191	6	152	6E-49	91%	91%	289185786	Bacteria	n	n	uncultured bacterium
GKJWQY101AOFRT	245	17	194	7E-84	99%	99%	289656603	Bacteria	n	n	uncultured bacterium
GKJWQY101AC3OO	104	4	48	1E-13	100%	100%	290564556	Bacteria	n	n	uncultured bacterium
GKJWQY101AO7X1	208	17	128	1E-41	96%	96%	290591144	Bacteria	n	n	uncultured bacterium
GKJWQY101BSD8Q	473	143	178	0.00001	97%	97%	290596837	Bacteria	n	n	uncultured bacterium
GKJWQY101AU3TD	88	19	56	6E-10	100%	100%	290604206	Bacteria	n	n	uncultured bacterium
GKJWQY101BRKP7	177	5	160	7E-53	92%	92%	290611134	Bacteria	n	n	uncultured bacterium
GKJWQY101B3CO0	211	5	163	4E-71	98%	98%	290618299	Bacteria	n	n	uncultured bacterium
GKJWQY101AWPF6	225	24	181	4E-66	96%	96%	290618303	Bacteria	n	n	uncultured bacterium
GKJWQY101BI53V	248	18	138	2E-55	100%	100%	290620788	Bacteria	n	n	uncultured bacterium

GKJWQY101BTFT8	225	67	141	4E-26	97%	97%	290621759	Bacteria	n	n	uncultured bacterium
GKJWQY101BER09	186	5	153	3E-71	100%	100%	290625301	Bacteria	n	n	uncultured bacterium
GKJWQY101A3SBZ	181	5	137	2E-62	100%	100%	290770388	Bacteria	n	n	uncultured bacterium
GKJWQY101BSAL8	98	4	66	9E-24	100%	100%	290783722	Bacteria	n	n	uncultured bacterium
GKJWQY101AR8CU	184	23	182	2E-77	100%	100%	291067147	Bacteria	n	n	uncultured bacterium
GKJWQY101A3CG5	230	5	182	2E-65	93%	93%	291192724	Bacteria	n	n	uncultured bacterium
GKJWQY101BYJU2	164	5	105	1E-39	97%	97%	291246515	Bacteria	n	n	uncultured bacterium
GKJWQY101ASA6U	149	18	100	1E-34	100%	100%	291249052	Bacteria	n	n	uncultured bacterium
GKJWQY101BF0DI	67	18	62	5E-09	93%	93%	291249353	Bacteria	n	n	uncultured bacterium
GKJWQY101BTIQM	177	1	132	3E-56	98%	98%	291252246	Bacteria	n	n	uncultured bacterium
GKJWQY101BAAER	185	18	86	1E-26	100%	100%	291510658	Bacteria	n	n	uncultured bacterium
GKJWQY101A02NJ	200	18	168	6E-69	99%	99%	295008960	Bacteria	n	n	uncultured bacterium
GKJWQY101AVMBQ	106	24	61	8E-10	100%	100%	295012247	Bacteria	n	n	uncultured bacterium
GKJWQY101A3CHU	218	17	187	2E-63	93%	93%	295013607	Bacteria	n	n	uncultured bacterium
GKJWQY101ADQPK	186	5	137	1E-60	99%	99%	295013630	Bacteria	n	n	uncultured bacterium
GKJWQY101BR4RJ	260	5	193	3E-93	100%	100%	295027769	Bacteria	n	n	uncultured bacterium
GKJWQY101AIPYT	186	5	137	2E-54	97%	97%	295045179	Bacteria	n	n	uncultured bacterium
GKJWQY101AQDVB	104	28	73	3E-14	100%	100%	295083163	Bacteria	n	n	uncultured bacterium
GKJWQY101BM4L2	95	5	45	1E-11	100%	100%	295638929	Bacteria	n	n	uncultured bacterium
GKJWQY101BH7TJ	266	60	235	5E-86	100%	100%	295810063	Bacteria	n	n	uncultured bacterium
GKJWQY101AWOPR	185	5	130	2E-58	100%	100%	295810065	Bacteria	n	n	uncultured bacterium
GKJWQY101BD5HX	204	5	156	2E-69	99%	99%	295810143	Bacteria	n	n	uncultured bacterium
GKJWQY101AL26W	207	19	150	2E-54	97%	97%	295810432	Bacteria	n	n	uncultured bacterium
GKJWQY101AU1LV	120	5	92	2E-37	100%	100%	295810565	Bacteria	n	n	uncultured bacterium
GKJWQY101BBPAI	146	5	95	4E-39	100%	100%	62997521	Bacteria	n	n	uncultured bacterium
GKJWQY101BI2RK	467	190	413	1E-104	98%	98%	223030240	Bacteria	n	n	uncultured bacterium
GKJWQY101BRKQJ	268	24	216	4E-92	99%	99%	223033480	Bacteria	n	n	uncultured bacterium
GKJWQY101BPZ30	248	27	203	3E-73	96%	96%	295016296	Bacteria	n	n	uncultured compost bacterium
GKJWQY101B2BOY	143	21	98	7E-32	100%	100%	16517864	Bacteria	n	n	uncultured soil bacterium
GKJWQY101BK6K3	245	13	194	2E-84	98%	98%	283444151	Bacteria	n	n	uncultured soil bacterium
GKJWQY101A6O01	167	1	167	8E-73	97%	97%	193084603	Bacteria	n	n	Uncultured bacterium
GKJWQY101ARH2A	177	6	125	2E-53	99%	99%	291258594	Bacteria	n	n	uncultured bacterium
GKJWQY101BHTXI	135	17	105	8E-36	99%	99%	291259108	Bacteria	n	n	uncultured bacterium
GKJWQY101B1CM3	234	9	121	4E-17	83%	83%	291259661	Bacteria	n	n	uncultured bacterium
GKJWQY101B0GC4	232	5	182	1E-80	98%	98%	291259901	Bacteria	n	n	uncultured bacterium
GKJWQY101BK2V0	220	5	126	4E-46	95%	95%	291260044	Bacteria	n	n	uncultured bacterium
GKJWQY101A3QAF	430	5	174	8E-71	96%	96%	291260145	Bacteria	n	n	uncultured bacterium
GKJWQY101A4I2H	200	23	145	1E-51	98%	98%	291260173	Bacteria	n	n	uncultured bacterium
GKJWQY101A52X5	151	5	72	2E-22	97%	97%	291260287	Bacteria	n	n	uncultured bacterium
GKJWQY101AC0EY	132	5	59	5E-13	93%	93%	291260377	Bacteria	n	n	uncultured bacterium
GKJWQY101BH0G8	159	5	101	2E-42	100%	100%	291260391	Bacteria	n	n	uncultured bacterium
GKJWQY101B1PKG	118	5	74	3E-19	93%	93%	291260525	Bacteria	n	n	uncultured bacterium
GKJWQY101BEX01	215	52	140	2E-24	91%	91%	291260593	Bacteria	n	n	uncultured bacterium
GKJWQY101A0SSA	243	67	180	5E-21	84%	84%	291260615	Bacteria	n	n	uncultured bacterium
GKJWQY101BNG1Z	167	5	115	2E-48	99%	99%	291260854	Bacteria	n	n	uncultured bacterium
GKJWQY101AZHPN	175	15	119	2E-18	84%	84%	291261156	Bacteria	n	n	uncultured bacterium
GKJWQY101AI0QB	126	17	91	1E-28	99%	99%	291261438	Bacteria	n	n	uncultured bacterium
GKJWQY101AX367	102	5	46	0.0000005	93%	93%	291261727	Bacteria	n	n	uncultured bacterium
GKJWQY101BAGJP	139	4	84	7E-32	99%	99%	291261757	Bacteria	n	n	uncultured bacterium
GKJWQY101AH42X	188	31	108	1E-16	89%	89%	12583967	Bacteria	Planctomycetes	Planctomycetaceae	planctomycete str. 139
GKJWQY101BQI14	154	51	119	8E-22	96%	96%	12583972	Bacteria	Planctomycetes	Planctomycetaceae	planctomycete str. 670
GKJWQY101A0ZU2	198	5	144	4E-66	100%	100%	285200728	Bacteria	Proteobacteria	n	alpha proteobacterium oral taxon A67
GKJWQY101A6E43	105	8	62	2E-15	96%	96%	35464139	Bacteria	Proteobacteria	n	uncultured alpha proteobacterium
GKJWQY101BQS3G	101	2	60	2E-21	100%	100%	55975756	Bacteria	Proteobacteria	n	uncultured alpha proteobacterium
GKJWQY101BHUK1	433	18	367	6E-167	97%	97%	146429045	Bacteria	Proteobacteria	n	uncultured alpha proteobacterium
GKJWQY101AYR9D	217	5	149	7E-69	100%	100%	295147952	Bacteria	Proteobacteria	Methylobacteriaceae	Methylobacterium fujisawaense
GKJWQY101BTLIZ	177	17	143	3E-51	96%	96%	289185500	Bacteria	Proteobacteria	Rhodobiaceae	Afifella marina
GKJWQY101AL2H8	139	4	82	9E-31	99%	99%	109140177	Bacteria	Proteobacteria	Alcaligenaceae	uncultured Alcaligenes sp.
GKJWQY101ANXLQ	190	5	119	3E-52	100%	100%	76665718	Bacteria	Proteobacteria	Burkholderiaceae	Burkholderia sp. STM1424
GKJWQY101AUNYR	226	5	180	7E-84	99%	99%	269113442	Bacteria	Proteobacteria	Burkholderiaceae	uncultured Burkholderia sp.

GKJWQY101AGFKW	130	5	77	4E-29	100%	100%	189305112	Bacteria	Proteobacteria	Burkholderiaceae	uncultured Ralstonia sp.
GKJWQY101BA25Y	151	4	110	6E-48	100%	100%	184189965	Bacteria	Proteobacteria	Comamonadaceae	uncultured Comamonadaceae bacterium
GKJWQY101A9UVV	302	18	154	2E-60	99%	99%	284793547	Bacteria	Proteobacteria	n	uncultured beta proteobacterium
GKJWQY101BME2Z	235	5	179	2E-85	100%	100%	112012332	Bacteria	Proteobacteria	Helicobacteraceae	Helicobacter sp. MIT 01-3238
GKJWQY101APNF5	164	56	130	3E-21	94%	94%	260986235	Bacteria	Proteobacteria	Enterobacteriaceae	Erwinia sp. AaMG18
GKJWQY101AG17Z	148	5	95	3E-35	98%	98%	156618579	Bacteria	Proteobacteria	n	gamma proteobacterium A11
GKJWQY101AX2T2	204	32	158	3E-57	99%	99%	291195506	Bacteria	Proteobacteria	n	gamma proteobacterium enrichment culture clone BP44-5
GKJWQY101AG5GL	101	4	62	3E-19	98%	98%	8547190	Bacteria	Proteobacteria	n	uncultured gamma proteobacterium KEppiB7
GKJWQY101AGSVT	182	18	129	3E-21	85%	85%	159576434	Bacteria	Proteobacteria	Halomonadaceae	Halomonas sp. S8-1
GKJWQY101APW63	205	16	169	6E-74	100%	100%	192757978	Bacteria	Proteobacteria	Moraxellaceae	Psychrobacter frigidicola
GKJWQY101BCXNO	231	5	175	2E-79	99%	99%	13276758	Bacteria	Proteobacteria	Moraxellaceae	Psychrobacter immobilis
GKJWQY101AGV77	240	5	136	5E-31	87%	87%	168812040	Bacteria	Proteobacteria	Moraxellaceae	Psychrobacter maritimus
GKJWQY101ATVI7	196	5	107	1E-45	100%	100%	222142510	Bacteria	Proteobacteria	Moraxellaceae	Psychrobacter sp. 24
GKJWQY101AO5SO	172	18	90	5E-29	100%	100%	46250582	Bacteria	Proteobacteria	Moraxellaceae	Psychrobacter sp. CH61
GKJWQY101BVGIC	225	73	176	4E-46	100%	100%	209422607	Bacteria	Proteobacteria	Moraxellaceae	uncultured Acinetobacter sp.
GKJWQY101BNT3R	127	5	77	4E-24	96%	96%	10719523	Bacteria	Proteobacteria	Moraxellaceae	uncultured Psychrobacter SIC.10360
GKJWQY101B0ZPN	207	62	146	2E-33	99%	99%	295345523	Bacteria	Proteobacteria	Pseudomonadaceae	Pseudomonas sp. enrichment culture clone 23.2
GKJWQY101AD6DO	175	21	123	1E-45	100%	100%	295651552	Bacteria	Proteobacteria	Pseudomonadaceae	Pseudomonas sp. HMD3178
GKJWQY101AJLCW	334	17	133	4E-53	100%	100%	295815425	Bacteria	Proteobacteria	Pseudomonadaceae	Pseudomonas sp. III_B28
GKJWQY101BPHU6	70	18	59	3E-12	100%	100%	164707704	Bacteria	Proteobacteria	Pseudomonadaceae	Pseudomonas sp. PGO22
GKJWQY101BQTSN	196	4	147	2E-68	100%	100%	259090496	Bacteria	Proteobacteria	Pseudomonadaceae	Pseudomonas sp. VS05_30
GKJWQY101BZ7PS	172	4	138	4E-60	99%	99%	129561834	Bacteria	Proteobacteria	Pseudomonadaceae	Pseudomonas sp. WW6
GKJWQY101AHQ2U	206	93	184	2E-29	94%	94%	283979678	Bacteria	Proteobacteria	Pseudomonadaceae	uncultured Pseudomonadaceae bacterium
GKJWQY101BGAKE	423	17	168	2E-72	100%	100%	163676404	Bacteria	Proteobacteria	Pseudomonadaceae	uncultured Pseudomonas sp.
GKJWQY101BJYWX	357	80	324	1E-122	100%	100%	238543855	Bacteria	Proteobacteria	Pseudomonadaceae	uncultured Pseudomonas sp.
GKJWQY101BZD8Z	89	1	44	3E-13	100%	100%	242124429	Bacteria	Proteobacteria	Pseudomonadaceae	uncultured Pseudomonas sp.
GKJWQY101AYZXN	265	25	197	2E-84	100%	100%	295687302	Bacteria	Proteobacteria	Pseudomonadaceae	uncultured Pseudomonas sp.
GKJWQY101BI5B7	133	5	75	5E-18	92%	92%	151936519	Bacteria	Proteobacteria	n	uncultured proteobacterium
GKJWQY101BHXF9	221	18	190	2E-79	98%	98%	154189153	Bacteria	Proteobacteria	n	uncultured proteobacterium
GKJWQY101BFJ8J	221	3	160	9E-48	90%	90%	197734890	Bacteria	Proteobacteria	Rhizobiaceae	Sinorhizobium meliloti
GKJWQY101B09E4_2	227	1	227	5E-30	74%	74%	345468266	Bacteria	Proteobacteria	Campylobacteraceae	Arcobacter
GKJWQY101AY5N9_2	141	1	141	2E-53	94%	94%	116651960	Bacteria	Proteobacteria	Burkholderiaceae	Burkholderia cepacia complex
GKJWQY101BV8TV	70	1	70	9E-28	100%	100%	384478111	Bacteria	Proteobacteria	Enterobacteriaceae	Providencia
GKJWQY101ASNWF	122	16	76	1E-18	97%	97%	9828144	Eukaryota	Ascomycota	Lecanoraceae	Lecanora intumescens
GKJWQY101A5TK8	127	19	64	4E-14	100%	100%	268633305	Eukaryota	Bacillariophyta	Thalassiosiraceae	Stephanodiscus sp. KHR001
GKJWQY101BTMVW	234	18	202	1E-71	94%	94%	290770772	Eukaryota	Chlorophyta	Pycnococcaceae	Nephroselmis astigmatica
GKJWQY101AGMQF	507	4	251	9E-106	95%	95%	290770792	Eukaryota	Chlorophyta	n	Pyramimonas parkeae
GKJWQY101ARIDL	299	4	233	1E-107	98%	98%	290770790	Eukaryota	Chlorophyta	n	Pyramimonas tetrarhynchus
GKJWQY101AQVXH	245	32	200	2E-70	96%	96%	290770770	Eukaryota	Chlorophyta	Pycnococcaceae	Pseudoscourfieldia marina
GKJWQY101A9ES5	143	21	89	3E-25	99%	99%	225545967	Eukaryota	Heterokontophyta	n	uncultured labyrinthulid
GKJWQY101BX53J	135	37	80	5E-13	100%	100%	159031144	Eukaryota	Mollusca	Veneridae	Nutricola tantilla
GKJWQY101ASWK5	233	85	154	4E-17	92%	92%	220942102	Eukaryota	n	n	Bilateria environmental sample
GKJWQY101A02BY	209	5	164	6E-74	99%	99%	222089870	Eukaryota	n	n	uncultured eukaryote
GKJWQY101AJBZL	123	16	71	2E-17	98%	98%	218684719	Eukaryota	n	n	uncultured fungus
GKJWQY101BEOOB	167	1	114	4E-50	99%	99%	268637040	Eukaryota	n	n	uncultured fungus
GKJWQY101A1CUV	546	5	518	0	93%	93%	56713123	Eukaryota	n	n	uncultured phototrophic eukaryote
GKJWQY101BQ79B	114	2	63	4E-23	100%	100%	223674440	Eukaryota	n	n	uncultured phototrophic eukaryote
GKJWQY101BANY8	110	18	58	2E-11	100%	100%	39547204	Eukaryota	n	n	uncultured rumen protozoa
GKJWQY101BVC5X	141	19	96	2E-26	96%	96%	291262345	Eukaryota	n	n	uncultured eukaryote
GKJWQY101B0QOY_2	113	1	113	1E-44	96%	96%	291261826	Eukaryota	n	n	Uncultured eukaryote
GKJWQY101BZIGB	493	212	425	4E-94	96%	96%	57340766	Eukaryota	Streptophyta	n	Napoleona sp. JS-2005
GKJWQY101AF93I	149	9	107	7E-42	99%	99%	284506657	Eukaryota	Streptophyta	n	Zygnematales sp. M3006
GKJWQY101AM47G	217	5	93	5E-20	89%	89%	284506685	Eukaryota	Streptophyta	Zygnemataceae	Spirogyra sp. M1843
GKJWQY101AL9IH	486	19	99	3E-21	91%	91%	284506686	Eukaryota	Streptophyta	Zygnemataceae	Zygnema sp. M-1156
GKJWQY101AWRLV	167	1	167	2E-78	99%	99%	367479280	Eukaryota	Streptophyta	Gentianales	Asclepias
GKJWQY101AQ6JO	415	69	228	6E-57	93%	93%	216963381	Eukaryota	Tardigrada	Milnesiidae	Milnesium tardigradum
GKJWQY101A9BVY	144	20	101	2E-32	99%	99%	227452751	Eukaryota	Zygomycota	Mucoraceae	Gongronella sp. xt-2009
