# Supplementary material for: Subglacial Lake Vostok (Antarctica) Accretion Ice Contains a Diverse Set of Sequences from Aquatic, Marine and Sediment-Inhabiting Bacteria and Eukarya
Source: PLoS One. 2013 Jul 3;8(7):e67221. doi: 10.1371/journal.pone.0067221 (PMC3700977; doi:10.1371/journal.pone.0067221)
Supplement: Table S4 — Bacteria mRNA (and other non-rRNA) gene sequences from V5. [“n” indicates information not specified in the NCBI GenBank database.]. (PDF) [file pone.0067221.s009.pdf]

Table S4. Bacteria mRNA (and other non-rRNA) gene sequences from V5. ["n" indicates information not specified in the NCBI GenBank database.]

454 Sequence ID	Q length	Q start	Q end	e-value	%-ident	%-sim	GI number	Domain	Phylum	Class / Order	Description
GKJWQY101AGGHX	545	5	506	0	97%	97%	31789410	Bacteria	Acidobacteria	n	Uncultured Acidobacteria bacterium clone 38c7 genomic sequence
GKJWQY101BLAHS	571	66	567	2E-172	89%	89%	117647674	Bacteria	Actinobacteria	Actinobacteria	Acidothermus cellulolyticus 11B strain 11B, complete genome
GKJWQY101BM4QM	251	3	199	2E-75	93%	93%	140843962	Bacteria	Actinobacteria	Actinobacteria	Corynebacterium glutamicum R DNA, complete genome
GKJWQY101BHEK2	433	5	376	0	98%	98%	111147037	Bacteria	Actinobacteria	Actinobacteria	Frankia alni str. ACN14A chromosome, complete sequence
GKJWQY101ALVRX	500	5	174	2E-82	100%	100%	119951388	Bacteria	Actinobacteria	Actinobacteria	Arthrobacter aurescens TC1 plasmid TC1, complete sequence
GKJWQY101B2Q7Z	448	36	408	3E-140	91%	91%	41019279	Bacteria	Actinobacteria	Actinobacteria	Micromonospora echinospora ATCC 15835 gentamicin biosynthesis gene cluster, complete sequence
GKJWQY101BJIYE	542	38	541	3E-180	90%	90%	145301903	Bacteria	Actinobacteria	Actinobacteria	Salinispora tropica CNB-440, complete genome
GKJWQY101BP0TD	550	202	550	1E-80	83%	83%	118168627	Bacteria	Actinobacteria	Actinobacteria	Mycobacterium smegmatis str. MC2 155, complete genome
GKJWQY101B2BHA	504	342	439	1E-39	98%	98%	76556220	Bacteria	Actinobacteria	Actinobacteria	Propionibacterium freudenreichii subsp. shermanii fba2 gene for fructose-bisphosphate aldolase class I
GKJWQY101AE43F	573	18	571	0	91%	91%	133909243	Bacteria	Actinobacteria	Actinobacteria	Saccharopolyspora erythraea NRRL2338 complete genome
GKJWQY101A68QX	413	315	358	0.0000002	93%	93%	24413764	Bacteria	Actinobacteria	Actinobacteria	Streptomyces coelicolor A3(2) complete genome; segment 9/29
GKJWQY101AH1E9	507	161	482	2E-83	85%	85%	260644157	Bacteria	Actinobacteria	Actinobacteria	Streptomyces scabiei 87.22 complete genome
GKJWQY101BPEIQ	456	17	410	0	96%	96%	118764602	Bacteria	Actinobacteria	Actinobacteria	Bifidobacterium adolescentis ATCC 15703 DNA, complete genome
GKJWQY101AFA5U	502	380	494	8E-27	87%	87%	295793053	Bacteria	Actinobacteria	Actinobacteria	Bifidobacterium animalis subsp. lactis V9, complete genome
GKJWQY101A4K6T	615	4	148	2E-29	85%	85%	291516109	Bacteria	Actinobacteria	Actinobacteria	Bifidobacterium longum longum F8 draft genome
GKJWQY101AAOCE	567	19	512	3E-136	85%	85%	257473675	Bacteria	Actinobacteria	Actinobacteria	Eggerthella lenta DSM 2243, complete genome
GKJWQY101B0JWH	576	227	572	4E-115	89%	89%	295105686	Bacteria	Actinobacteria	Actinobacteria	Gordonibacter pamelaeae 7-10-1-bT draft genome
GKJWQY101BKG7S	547	17	512	6E-148	86%	86%	108764099	Bacteria	Actinobacteria	Actinobacteria	Rubrobacter xylanophilus DSM 9941, complete genome
GKJWQY101A5PQZ	510	4	427	4E-124	86%	86%	256007408	Bacteria	Actinobacteria	Actinobacteria	Acidimicrobium ferrooxidans DSM 10331, complete genome
GKJWQY101A65QS	554	23	549	0	95%	95%	38200856	Bacteria	Actinobacteria	Actinobacteria	Corynebacterium diphtheriae gravis NCTC13129, complete genome; segment 7/8
GKJWQY101BB5IP	574	5	569	0	94%	94%	68262661	Bacteria	Actinobacteria	Actinobacteria	Corynebacterium jeikeium K411 complete genome
GKJWQY101BC0IC	557	16	556	3E-175	88%	88%	237757549	Bacteria	Actinobacteria	Actinobacteria	Corynebacterium kroppenstedtii DSM 44385, complete genome
GKJWQY101AQ51G	597	5	571	0	88%	88%	256687298	Bacteria	Actinobacteria	Actinobacteria	Kytococcus sedentarius DSM 20547, complete genome
GKJWQY101AV3CT	587	19	581	0	93%	93%	119947346	Bacteria	Actinobacteria	Actinobacteria	Arthrobacter aurescens TC1, complete genome
GKJWQY101ASGX9	573	17	503	2E-173	90%	90%	183579876	Bacteria	Actinobacteria	Actinobacteria	Kocuria rhizophila DC2201 DNA, complete genome
GKJWQY101A6R2G	571	5	570	0	94%	94%	162952245	Bacteria	Actinobacteria	Actinobacteria	Renibacterium salmoninarum ATCC 33209, complete genome
GKJWQY101BURAB	566	21	523	2E-92	80%	80%	119953846	Bacteria	Actinobacteria	Actinobacteria	Mycobacterium vanbaalenii PYR-1, complete genome
GKJWQY101BLJAK	582	5	398	1E-144	91%	91%	54013472	Bacteria	Actinobacteria	Actinobacteria	Nocardia farcinica IFM 10152 DNA, complete genome
GKJWQY101APUX9	573	5	569	4E-170	87%	87%	110816552	Bacteria	Actinobacteria	Actinobacteria	Rhodococcus jostii RHA1, complete genome
GKJWQY101A2KCQ	591	17	589	0	94%	94%	269095543	Bacteria	Actinobacteria	Actinobacteria	Sanguibacter keddieii DSM 10542, complete genome
GKJWQY101ANZOF	303	4	68	2E-15	92%	92%	212548595	Bacteria	Bacteroidetes	Bacteroidia	Candidatus Azobacteroides pseudotrichonymphae genomovar. CFP2 DNA, complete genome
GKJWQY101BUUW5	565	17	562	0	93%	93%	188593544	Bacteria	Bacteroidetes	Bacteroidia	Porphyromonas gingivalis ATCC 33277 DNA, complete genome
GKJWQY101BQQMF	393	18	342	4E-78	84%	84%	291513545	Bacteria	Bacteroidetes	Bacteroidia	Alistipes shahii WAL 8301 draft genome
GKJWQY101BGIIN	310	18	247	3E-89	93%	93%	254946573	Bacteria	Bacteroidetes	Cytophagia	Dyadobacter fermentans DSM 18053, complete genome
GKJWQY101B2ENG	246	18	200	4E-62	91%	91%	283814236	Bacteria	Bacteroidetes	Cytophagia	Spirosoma linguale DSM 74, complete genome
GKJWQY101BHBKI	544	18	518	0	96%	96%	294979899	Bacteria	Bacteroidetes	Flavobacteria	Zunongwangia profunda SM-A87, complete genome
GKJWQY101AJ2KZ	595	18	592	0	89%	89%	52214156	Bacteria	Bacteroidetes	Bacteroidia	Bacteroides fragilis YCH46 DNA, complete genome
GKJWQY101A4I0Z	576	19	574	0	88%	88%	60491031	Bacteria	Bacteroidetes	Bacteroidia	Bacteroides fragilis NCTC 9343, complete genome
GKJWQY101BS0NP	584	64	582	0	91%	91%	29342101	Bacteria	Bacteroidetes	Bacteroidia	Bacteroides thetaiotaomicron VPI-5482, complete genome
GKJWQY101AN552	561	4	559	0	88%	88%	149931032	Bacteria	Bacteroidetes	Bacteroidia	Bacteroides vulgatus ATCC 8482, complete genome
GKJWQY101AWOSS	589	4	587	0	87%	87%	149935098	Bacteria	Bacteroidetes	Bacteroidia	Parabacteroides distasonis ATCC 8503, complete genome
GKJWQY101AP6Y7	476	18	421	8E-156	92%	92%	34398108	Bacteria	Bacteroidetes	Bacteroidia	Porphyromonas gingivalis W83, complete genome
GKJWQY101BYSCO	548	7	537	7E-167	87%	87%	294471613	Bacteria	Bacteroidetes	Bacteroidia	Prevotella ruminicola 23, complete genome
GKJWQY101BJIPK	572	1	569	0	92%	92%	255340365	Bacteria	Bacteroidetes	Flavobacteria	Flavobacteriaceae bacterium 3519-10, complete genome
GKJWQY101BZXZT	586	18	571	0	94%	94%	146152184	Bacteria	Bacteroidetes	Flavobacteria	Flavobacterium johnsoniae UW101, complete genome
GKJWQY101BEOT5	555	20	329	8E-132	95%	95%	149770655	Bacteria	Bacteroidetes	Flavobacteria	Flavobacterium psychrophilum JIP02/86 complete genome
GKJWQY101ABFUA	587	17	582	0	89%	89%	255342900	Bacteria	Bacteroidetes	Sphingobacteria	Pedobacter heparinus DSM 2366, complete genome
GKJWQY101A91OF	548	15	506	0	91%	91%	295083795	Bacteria	Bacteroidetes	Bacteroidia	Bacteroides xylanisolvens XB1A draft genome
GKJWQY101A8VQ6	567	32	251	4E-45	83%	83%	269787736	Bacteria	Chloroflexi	Thermomicrobia	Sphaerobacter thermophilus DSM 20745 chromosome 2, complete sequence
GKJWQY101ASMWQ	163	29	136	1E-40	95%	95%	37508091	Bacteria	Cyanobacteria	Gloeobacteria	Gloeobacter violaceus PCC 7421 DNA, complete genome
GKJWQY101BB7VS	233	67	184	2E-40	93%	93%	171696371	Bacteria	Cyanobacteria	Chroococcales	Cyanothece sp. ATCC 51142 circular chromosome, complete sequence
GKJWQY101ADTE9	305	19	271	2E-90	91%	91%	218169741	Bacteria	Cyanobacteria	Chroococcales	Cyanothece sp. PCC 7424, complete genome
GKJWQY101AIKHU	277	4	217	4E-92	96%	96%	256588085	Bacteria	Cyanobacteria	Chroococcales	Cyanothece sp. PCC 8802, complete genome
GKJWQY101A4H47	553	333	381	4E-10	94%	94%	256592529	Bacteria	Cyanobacteria	Chroococcales	Cyanothece sp. PCC 8802 plasmid pP880203, complete sequence
GKJWQY101AXWLH	352	18	320	3E-104	90%	90%	146740642	Bacteria	Cyanobacteria	Chroococcales	Synechococcus rubescens SAG 3.81 partial ribosomal RNA operon, strain SAG 3.81
GKJWQY101BYVV6	360	16	277	7E-41	79%	79%	147849409	Bacteria	Cyanobacteria	Chroococcales	Synechococcus sp. RCC307 genomic DNA sequence
GKJWQY101AGPF8	340	16	279	4E-43	80%	80%	33633126	Bacteria	Cyanobacteria	Chroococcales	Synechococcus sp. WH8102 complete genome; segment 6/7
GKJWQY101AQPZB	526	5	383	2E-68	80%	80%	47118315	Bacteria	Cyanobacteria	Chroococcales	Thermosynechococcus elongatus BP-1 DNA, complete genome
GKJWQY101ACHK0	426	24	349	8E-61	81%	81%	242955773	Bacteria	Cyanobacteria	Nostocales	Fremyella diplosiphon Fd33 light-indpendent protochlorophyllide oxidoreductase subunit B (chlB) gene, complete cds
GKJWQY101BXWGA	568	157	184	0.002	100%	100%	111610610	Bacteria	Cyanobacteria	Nostocales	Fremyella diplosiphon Fd33 phycobilisome linker-core protein (apcC) and phycocyanobilin oxidoreductase (pcyA) genes, complete
GKJWQY101BY2F1	480	18	443	1E-124	86%	86%	47606667	Bacteria	Cyanobacteria	Nostocales	Fremyella diplosiphon TspO-like protein, hypothetical protein, NblA-1 (nblA), CpeB (cpeB), CpeA (cpeA), CpeY (cpeY), and CpeZ (cp
GKJWQY101A5ZEU	261	8	214	4E-92	97%	97%	186463002	Bacteria	Cyanobacteria	Nostocales	Nostoc punctiforme PCC 73102, complete genome
GKJWQY101APB60	447	5	401	1E-158	92%	92%	47118302	Bacteria	Cyanobacteria	Nostocales	Nostoc sp. PCC 7120 DNA, complete genome
GKJWQY101BC039	546	335	540	3E-17	76%	76%	17134864	Bacteria	Cyanobacteria	Nostocales	Nostoc sp. PCC 7120 plasmid pCC7120delta DNA, complete sequence
GKJWQY101ATFBR	558	5	556	0	90%	90%	146740643	Bacteria	Cyanobacteria	Oscillatoriales	Microcoleus chthonoplastes PCC 7420 partial ribosomal RNA operon, strain PCC 7420
GKJWQY101BRVHX	334	19	280	8E-85	89%	89%	146740638	Bacteria	Cyanobacteria	Prochlorales	Prochlorothrix hollandica SAG 10.89 partial ribosomal RNA operon, strain SAG 10.89
GKJWQY101BYL6X	559	4	543	0	90%	90%	158303474	Bacteria	Cyanobacteria	n	Acaryochloris marina MBIC11017, complete genome
GKJWQY101AWOW9	290	5	221	2E-80	92%	92%	284809060	Bacteria	Cyanobacteria	n	Cyanobacterium UCYN-A, complete genome

GKJWQY101BQ48U	481	5	412	1E-169	93%	93%	219862254	Bacteria	Cyanobacteria	n	Cyanothece sp. PCC 7425, complete genome
GKJWQY101BJ44C	613	18	609	0	88%	88%	56684969	Bacteria	Cyanobacteria	n	Synechococcus elongatus PCC 6301 DNA, complete genome
GKJWQY101BQPKI	556	19	546	2E-88	79%	79%	75699950	Bacteria	Cyanobacteria	n	Anabaena variabilis ATCC 29413, complete genome
GKJWQY101AKAL4	539	17	497	6E-158	88%	88%	110164990	Bacteria	Cyanobacteria	n	Trichodesmium erythraeum IMS101, complete genome
GKJWQY101AKQUQ	222	18	177	7E-74	99%	99%	146740644	Bacteria	Cyanobacteria	Oscillatoriales	Spirulina sp. PCC 6313 partial ribosomal RNA operon, strain PCC 6313
GKJWQY101AQDCE	479	17	414	0	98%	98%	290469363	Bacteria	Deinococcus-Thermus	Deinococci	Meiothermus ruber DSM 1279, complete genome
GKJWQY101A2UHA	548	5	238	6E-98	95%	95%	294979666	Bacteria	Deinococcus-Thermus	Deinococci	Chain A, Initiation Complex Of 70s Ribosome With Two Trnas And Mrna. This Entry 3i9e Contains 50s Ribosomal Subunit Of Molec
GKJWQY101AWMCZ	545	26	543	3E-156	86%	86%	226319394	Bacteria	Deinococcus-Thermus	Deinococci	Deinococcus deserti VCD115 plasmid 1, complete sequence
GKJWQY101AQZAT	611	79	551	4E-140	87%	87%	154350369	Bacteria	Firmicutes	Bacilli	Bacillus amyloliquefaciens FZB42, complete genome
GKJWQY101ACEUC	378	6	334	1E-103	88%	88%	225785631	Bacteria	Firmicutes	Bacilli	Bacillus cereus 03BB102, complete genome
GKJWQY101AA6R0	423	5	386	4E-163	94%	94%	218540569	Bacteria	Firmicutes	Bacilli	Bacillus cereus G9842, complete genome
GKJWQY101B2DJZ	416	18	364	6E-147	94%	94%	221237819	Bacteria	Firmicutes	Bacilli	Bacillus cereus Q1, complete genome
GKJWQY101ANNC9	607	25	557	4E-95	80%	80%	37903989	Bacteria	Firmicutes	Bacilli	Bacillus cereus strain BGSC 6A5 rrnB operon, complete sequence
GKJWQY101A6VHS	591	5	575	0	93%	93%	157679556	Bacteria	Firmicutes	Bacilli	Bacillus pumilus SAFR-032, complete genome
GKJWQY101BLJ1O	463	5	368	6E-157	95%	95%	226092535	Bacteria	Firmicutes	Bacilli	Brevibacillus brevis NBRC 100599 DNA, complete genome
GKJWQY101AHEDV	181	30	142	2E-43	96%	96%	111182492	Bacteria	Firmicutes	Bacilli	Enterococcus avium clone EPE8_4 genomic sequence
GKJWQY101BC6PS	383	13	79	3E-20	96%	96%	283481151	Bacteria	Firmicutes	Bacilli	Enterococcus faecium partial plasmid pVEF4, strain 399/F98/A4
GKJWQY101AMO9E	334	134	282	1E-58	95%	95%	111182490	Bacteria	Firmicutes	Bacilli	Enterococcus gallinarum clone EPE8_2 genomic sequence
GKJWQY101A9OD0	479	5	477	0	100%	100%	15146028	Bacteria	Firmicutes	Bacilli	Lactobacillus delbrueckii subsp. bulgaricus ISL5 transposase gene, complete cds
GKJWQY101ADA2E	517	5	472	0	99%	99%	15146030	Bacteria	Firmicutes	Bacilli	Lactobacillus delbrueckii subsp. lactis ISL6 transposase gene, complete cds
GKJWQY101BLMTA	376	5	330	7E-86	85%	85%	120400324	Bacteria	Firmicutes	Bacilli	Lactobacillus johnsonii strain ATCC 33200 exopolysaccharide biosynthesis gene cluster, complete sequence
GKJWQY101BI8YA	641	193	226	0.000001	100%	100%	195537732	Bacteria	Firmicutes	Bacilli	Lactobacillus plantarum L137 plasmid pLTK13 DNA, complete genome
GKJWQY101A2WZI	582	14	578	0	96%	96%	183223999	Bacteria	Firmicutes	Bacilli	Lactobacillus reuteri JCM 1112 DNA, complete genome
GKJWQY101AKANV	345	100	142	0.00003	91%	91%	116090851	Bacteria	Firmicutes	Bacilli	Oenococcus oeni PSU-1, complete genome
GKJWQY101ALDHC	261	17	182	8E-79	99%	99%	3582195	Bacteria	Firmicutes	Bacilli	Lactococcus lactis strain DPC3147 plasmid pMRC01, complete sequence
GKJWQY101A6FQL	507	18	504	0	99%	99%	116108977	Bacteria	Firmicutes	Bacilli	Lactococcus lactis subsp. cremoris SK11 plasmid 4, complete sequence
GKJWQY101AF275	567	23	362	6E-173	99%	99%	116106497	Bacteria	Firmicutes	Bacilli	Lactococcus lactis subsp. cremoris SK11, complete genome
GKJWQY101A5HHI	374	5	271	9E-105	93%	93%	23094784	Bacteria	Firmicutes	Bacilli	Streptococcus agalactiae NEM316 complete genome, segment 3
GKJWQY101A7KUH	203	5	159	4E-71	99%	99%	113120160	Bacteria	Firmicutes	Bacilli	Streptococcus macedonicus strain ACA-DC 198 putative relaxase gene, complete cds; macedocin gene cluster, complete sequence
GKJWQY101BXGNS	493	5	378	0	99%	99%	225724295	Bacteria	Firmicutes	Bacilli	Streptococcus pneumoniae P1031, complete genome
GKJWQY101AFNBN	518	18	511	1E-169	89%	89%	25307955	Bacteria	Firmicutes	Bacilli	Streptococcus pneumoniae R6, complete genome
GKJWQY101A2ND4	505	5	355	5E-143	93%	93%	168994879	Bacteria	Firmicutes	Bacilli	Streptococcus pneumoniae Hungary19A-6, complete genome
GKJWQY101BVGTG	546	4	543	0	97%	97%	182628304	Bacteria	Firmicutes	Bacilli	Streptococcus pneumoniae CGSP14, complete genome
GKJWQY101A2QNZ	477	17	422	0	98%	98%	209539788	Bacteria	Firmicutes	Bacilli	Streptococcus pyogenes NZ131, complete genome
GKJWQY101AIT3S	370	24	324	1E-93	88%	88%	21905618	Bacteria	Firmicutes	Bacilli	Streptococcus pyogenes MGAS315, complete genome
GKJWQY101BVSNC	236	18	199	9E-83	98%	98%	158139258	Bacteria	Firmicutes	Clostridia	Alkaliphilus oremlandii OhILAs, complete genome
GKJWQY101BIJGR	305	24	146	4E-48	96%	96%	149901357	Bacteria	Firmicutes	Clostridia	Clostridium beijerinckii NCIMB 8052, complete genome
GKJWQY101AVTLD	316	127	276	1E-67	99%	99%	295317476	Bacteria	Firmicutes	Clostridia	Clostridium botulinum F str. 230613, complete genome
GKJWQY101AR4X1	537	18	537	0	100%	100%	110673209	Bacteria	Firmicutes	Clostridia	Clostridium perfringens ATCC 13124, complete genome
GKJWQY101AX0O9	546	25	207	1E-74	95%	95%	295089810	Bacteria	Firmicutes	Clostridia	Clostridium saccharolyticum-like K10 draft genome
GKJWQY101APBWG	500	18	322	6E-118	93%	93%	223928079	Bacteria	Firmicutes	Clostridia	Clostridium sp. enrichment culture clone 7-14 genomic sequence
GKJWQY101BXM9C	573	12	555	1E-115	82%	82%	125712750	Bacteria	Firmicutes	Clostridia	Clostridium thermocellum ATCC 27405, complete genome
GKJWQY101B17VM	233	16	192	1E-86	100%	100%	256797400	Bacteria	Firmicutes	Clostridia	Anaerococcus prevotii DSM 20548, complete genome
GKJWQY101AF4EN	544	18	542	0	98%	98%	167830502	Bacteria	Firmicutes	Clostridia	Finegoldia magna ATCC 29328 DNA, complete genome
GKJWQY101AIZHR	472	23	409	3E-135	90%	90%	291556121	Bacteria	Firmicutes	Clostridia	Eubacterium siraeum V10Sc8a draft genome
GKJWQY101B2AHH	421	14	374	1E-138	92%	92%	291529795	Bacteria	Firmicutes	Clostridia	Eubacterium siraeum 70/3 draft genome
GKJWQY101BNDMO	405	57	351	1E-59	82%	82%	114336511	Bacteria	Firmicutes	Clostridia	Syntrophomonas wolfei subsp. wolfei str. Goettingen, complete genome
GKJWQY101BBVTI	484	5	441	0	99%	99%	229264291	Bacteria	Firmicutes	Bacilli	Bacillus anthracis str. A0248, complete genome
GKJWQY101AXQ8R	580	4	576	0	92%	92%	56908016	Bacteria	Firmicutes	Bacilli	Bacillus clausii KSM-K16 DNA, complete genome
GKJWQY101ASAUF	575	5	528	0	95%	95%	152022606	Bacteria	Firmicutes	Bacilli	Bacillus cereus subsp. cytotoxis NVH 391-98, complete genome
GKJWQY101BZHXT	548	5	548	0	93%	93%	47118318	Bacteria	Firmicutes	Bacilli	Bacillus halodurans C-125 DNA, complete genome
GKJWQY101BN6EO	577	18	573	0	90%	90%	145902672	Bacteria	Firmicutes	Bacilli	Bacillus licheniformis ATCC 14580, complete genome
GKJWQY101A440I	454	3	188	5E-83	97%	97%	294346812	Bacteria	Firmicutes	Bacilli	Bacillus megaterium QM B1551, complete genome
GKJWQY101A2M8L	595	5	588	0	93%	93%	294799901	Bacteria	Firmicutes	Bacilli	Bacillus megaterium DSM319, complete genome
GKJWQY101AM35J	538	5	537	0	96%	96%	225184640	Bacteria	Firmicutes	Bacilli	Bacillus subtilis subsp. subtilis str. 168 complete genome
GKJWQY101BMIJM	565	1	560	0	95%	95%	168990106	Bacteria	Firmicutes	Bacilli	Lysinibacillus sphaericus C3-41, complete genome
GKJWQY101BMULU	441	5	385	0	98%	98%	42632302	Bacteria	Firmicutes	Bacilli	Oceanobacillus iheyensis HTE831 DNA, complete genome
GKJWQY101BL46W	622	12	498	8E-167	89%	89%	289169617	Bacteria	Firmicutes	Bacilli	Listeria seeligeri serovar 1/2b str. SLCC3954 complete genome
GKJWQY101BIMJV	561	5	558	6E-168	86%	86%	171988566	Bacteria	Firmicutes	Bacilli	Exiguobacterium sibiricum 255-15, complete genome
GKJWQY101AZ6PA	461	109	412	7E-107	90%	90%	229467163	Bacteria	Firmicutes	Bacilli	Exiguobacterium sp. AT1b, complete genome
GKJWQY101AKUVY	246	18	204	2E-75	95%	95%	261280339	Bacteria	Firmicutes	Bacilli	Paenibacillus sp. Y412MC10, complete genome
GKJWQY101AL2EY	563	5	556	0	91%	91%	222119372	Bacteria	Firmicutes	Bacilli	Macrococcus caseolyticus JCSC5402 DNA, complete genome
GKJWQY101BOYUA	408	5	365	1E-178	98%	98%	222420101	Bacteria	Firmicutes	Bacilli	Staphylococcus carnosus subsp. carnosus TM300 complete genome
GKJWQY101AKXQT	538	18	531	0	97%	97%	68445725	Bacteria	Firmicutes	Bacilli	Staphylococcus haemolyticus JCSC1435 DNA, complete genome
GKJWQY101BWB1P	559	18	552	0	97%	97%	289178903	Bacteria	Firmicutes	Bacilli	Staphylococcus lugdunensis HKU09-01, complete genome
GKJWQY101AIN1W	569	5	564	0	94%	94%	72493824	Bacteria	Firmicutes	Bacilli	Staphylococcus saprophyticus subsp. saprophyticus ATCC 15305 DNA, complete genome
GKJWQY101A2H1I	561	29	521	6E-173	89%	89%	158967071	Bacteria	Firmicutes	Bacilli	Lactobacillus acidophilus NCFM, complete genome
GKJWQY101BFXEC	525	18	522	0	99%	99%	116103724	Bacteria	Firmicutes	Bacilli	Lactobacillus casei ATCC 334, complete genome
GKJWQY101BFH4F	563	60	395	2E-167	99%	99%	190711126	Bacteria	Firmicutes	Bacilli	Lactobacillus casei BL23 complete genome, strain BL23
GKJWQY101ACCFZ	548	22	545	0	99%	99%	103422338	Bacteria	Firmicutes	Bacilli	Lactobacillus delbrueckii subsp. bulgaricus ATCC 11842 complete genome
GKJWQY101AIPY1	586	18	585	0	98%	98%	116092543	Bacteria	Firmicutes	Bacilli	Lactobacillus delbrueckii subsp. bulgaricus ATCC BAA-365, complete genome
GKJWQY101BXUBT	538	1	516	0	93%	93%	262396937	Bacteria	Firmicutes	Bacilli	Lactobacillus johnsonii FI9785, complete genome

GKJWQY101AKKWT	451	18	307	7E-92	88%	88%	254044096	Bacteria	Firmicutes	Bacilli	Lactobacillus plantarum JDM1, complete genome
GKJWQY101A9FF7	586	5	583	0	97%	97%	259648365	Bacteria	Firmicutes	Bacilli	Lactobacillus rhamnosus ATCC 53103 DNA, complete genome
GKJWQY101APN55	556	5	554	4E-145	84%	84%	78609255	Bacteria	Firmicutes	Bacilli	Lactobacillus sakei strain 23K complete genome
GKJWQY101BF89U	570	5	564	0	99%	99%	90820184	Bacteria	Firmicutes	Bacilli	Lactobacillus salivarius UCC118, complete genome
GKJWQY101A6GDN	151	13	119	4E-39	95%	95%	295831662	Bacteria	Firmicutes	Bacilli	Leuconostoc kimchii IMSNU 11154, complete genome
GKJWQY101AZ303	492	16	431	0	96%	96%	13400022	Bacteria	Firmicutes	Bacilli	Lactococcus lactis subsp. lactis Il1403, complete genome
GKJWQY101AUKS3	528	3	525	0	99%	99%	124491690	Bacteria	Firmicutes	Bacilli	Lactococcus lactis subsp. cremoris MG1363, complete genome
GKJWQY101A3SPW	574	5	574	0	100%	100%	281374316	Bacteria	Firmicutes	Bacilli	Lactococcus lactis subsp. lactis KF147, complete genome
GKJWQY101AB8GR	544	5	516	0	93%	93%	22535226	Bacteria	Firmicutes	Bacilli	Streptococcus agalactiae 2603V/R, complete genome
GKJWQY101BZH5I	545	5	410	3E-170	94%	94%	225700893	Bacteria	Firmicutes	Bacilli	Streptococcus equi subsp. zooepidemicus H70, complete genome
GKJWQY101AH1ID	551	5	544	0	97%	97%	288730948	Bacteria	Firmicutes	Bacilli	Streptococcus gallolyticus UCN34 complete genome
GKJWQY101BI2WE	560	5	557	0	98%	98%	157074445	Bacteria	Firmicutes	Bacilli	Streptococcus gordonii str. Challis substr. CH1, complete genome
GKJWQY101AYWCN	564	5	559	0	97%	97%	254996425	Bacteria	Firmicutes	Bacilli	Streptococcus mutans NN2025 DNA, complete genome
GKJWQY101BOVEU	558	21	556	0	97%	97%	225726369	Bacteria	Firmicutes	Bacilli	Streptococcus pneumoniae Taiwan19F-14, complete genome
GKJWQY101A1CSL	395	4	362	1E-172	98%	98%	19913450	Bacteria	Firmicutes	Bacilli	Streptococcus pyogenes MGAS8232, complete genome
GKJWQY101BNM0N	430	107	383	6E-142	100%	100%	292557464	Bacteria	Firmicutes	Bacilli	Streptococcus suis GZ1, complete genome
GKJWQY101AYWMX	285	5	253	7E-105	95%	95%	222113012	Bacteria	Firmicutes	Bacilli	Streptococcus uberis 0140J complete genome
GKJWQY101AOC8C	305	17	260	2E-115	98%	98%	188497817	Bacteria	Firmicutes	Clostridia	Clostridium botulinum E3 str. Alaska E43, complete genome
GKJWQY101BE4ZG	596	18	590	0	95%	95%	110681940	Bacteria	Firmicutes	Clostridia	Clostridium perfringens SM101, complete genome
GKJWQY101BX0WB	552	1	504	1E-174	89%	89%	160426828	Bacteria	Firmicutes	Clostridia	Clostridium phytofermentans ISDg, complete genome
GKJWQY101AUKJZ	542	17	488	6E-173	90%	90%	238871336	Bacteria	Firmicutes	Clostridia	Eubacterium eligens ATCC 27750, complete genome
GKJWQY101BDY1Q	551	5	485	0	95%	95%	238874104	Bacteria	Firmicutes	Clostridia	Eubacterium rectale ATCC 33656, complete genome
GKJWQY101ARORW	494	18	447	4E-79	80%	80%	219536331	Bacteria	Firmicutes	Clostridia	Desulfitobacterium hafniense DCB-2, complete genome
GKJWQY101AHKOV	564	5	555	0	91%	91%	283951607	Bacteria	Firmicutes	Negativicutes	Acidaminococcus fermentans DSM 20731, complete genome
GKJWQY101BGGHX	576	17	568	0	99%	99%	269093698	Bacteria	Firmicutes	Negativicutes	Veillonella parvula DSM 2008, complete genome
GKJWQY101BUKAI	560	18	560	0	94%	94%	295112306	Bacteria	Firmicutes	Bacilli	Enterococcus sp. 7L76 draft genome
GKJWQY101BPUIN	555	18	555	0	100%	100%	257152781	Bacteria	Firmicutes	Bacilli	Lactobacillus rhamnosus Lc 705 plasmid sequence, strain Lc 705
GKJWQY101AW8QY	590	23	587	0	94%	94%	257149867	Bacteria	Firmicutes	Bacilli	Lactobacillus rhamnosus Lc 705 whole genome sequence, strain Lc 705
GKJWQY101ALG3R	326	18	259	5E-112	98%	98%	15487824	Bacteria	Firmicutes	Bacilli	Lactobacillus sakei rrnA operon and rrnB operon, partial sequence
GKJWQY101ATSIV	593	7	583	0	87%	87%	223928104	Bacteria	Firmicutes	Clostridia	Clostridium sp. enrichment culture clone 7-25 genomic sequence
GKJWQY101BNDWM	572	5	571	0	88%	88%	291523683	Bacteria	Firmicutes	Clostridia	Eubacterium rectale DSM 17629 draft genome
GKJWQY101A40TK	418	4	370	7E-141	92%	92%	291526582	Bacteria	Firmicutes	Clostridia	Eubacterium rectale M104/1 draft genome
GKJWQY101AO1P2	622	6	618	0	86%	86%	291520697	Bacteria	Firmicutes	Clostridia	Coprococcus catus GD/7 draft genome
GKJWQY101A44JY	561	7	541	0	94%	94%	295092884	Bacteria	Firmicutes	Clostridia	Coprococcus sp. ART55/1 draft genome
GKJWQY101BQCZD	544	9	543	0	92%	92%	295114602	Bacteria	Firmicutes	Clostridia	Clostridiales sp. SM4/1 draft genome
GKJWQY101BQTA7	480	18	427	0	95%	95%	291558333	Bacteria	Firmicutes	Clostridia	Clostridiales sp. SSC/2 draft genome
GKJWQY101AYGQY	563	24	559	2E-163	87%	87%	291541372	Bacteria	Firmicutes	Clostridia	Ruminococcus bromii L2-63 draft genome
GKJWQY101AKXDF	588	18	579	0	91%	91%	295107714	Bacteria	Firmicutes	Clostridia	Ruminococcus obeum A2-162 draft genome
GKJWQY101BCTZL	587	5	534	0	89%	89%	291543184	Bacteria	Firmicutes	Clostridia	Ruminococcus sp. 18P13 draft genome
GKJWQY101BBYCY	569	17	526	0	96%	96%	291545299	Bacteria	Firmicutes	Clostridia	Ruminococcus sp. SR1/5 draft genome
GKJWQY101BCX1D	600	15	598	0	90%	90%	291548560	Bacteria	Firmicutes	Clostridia	Ruminococcus torques L2-14 draft genome
GKJWQY101BSTMC	564	27	564	0	91%	91%	295098739	Bacteria	Firmicutes	Erysipelotrichi	Eubacterium cylindroides T2-87 draft genome
GKJWQY101BWHUV	584	5	572	0	89%	89%	291532143	Bacteria	Firmicutes	Negativicutes	Megamonas hypermegale ART12/1 draft genome
GKJWQY101AEIGL	570	18	559	0	91%	91%	20095250	Bacteria	Fusobacteria	Fusobacteria _class_	Fusobacterium nucleatum subsp. nucleatum ATCC 25586, complete genome
GKJWQY101BJ7Q1	563	17	560	0	94%	94%	257048753	Bacteria	Fusobacteria	Fusobacteria _class_	Leptotrichia buccalis DSM 1135, complete genome
GKJWQY101AANQS	765	8	284	2E-108	93%	93%	156773414	Bacteria	n	n	Uncultured bacterium clone LM0ACA6ZE09FM1 genomic sequence
GKJWQY101AP35G	369	18	305	3E-104	91%	91%	156774261	Bacteria	n	n	Uncultured bacterium clone HA0AAA18ZA07FM1 genomic sequence
GKJWQY101BYYCA	271	5	223	1E-106	99%	99%	156770305	Bacteria	n	n	Uncultured bacterium clone LM0ABA36ZC08RM1 genomic sequence
GKJWQY101BA89J	207	18	127	8E-43	96%	96%	156776473	Bacteria	n	n	Uncultured bacterium clone LM0ACA9ZB08RM1 genomic sequence
GKJWQY101BFWYL	285	18	240	2E-105	98%	98%	156769021	Bacteria	n	n	Uncultured bacterium clone LM0ABA7ZF10FM1 genomic sequence
GKJWQY101AR683	223	3	155	2E-69	99%	99%	156770979	Bacteria	n	n	Uncultured bacterium clone LM0ABA6ZE04RM1 genomic sequence
GKJWQY101AMU20	257	18	106	8E-24	91%	91%	156768878	Bacteria	n	n	Uncultured bacterium clone LM0ABA5ZG02FM1 genomic sequence
GKJWQY101A8WXN	563	18	515	0	91%	91%	156773397	Bacteria	n	n	Uncultured bacterium clone LM0ACA6ZD02FM1 genomic sequence
GKJWQY101B0JOO	229	65	198	9E-58	98%	98%	156773640	Bacteria	n	n	Uncultured bacterium clone LM0ACA9ZC05FM1 genomic sequence
GKJWQY101BM98Q	303	4	217	3E-78	92%	92%	156773646	Bacteria	n	n	Uncultured bacterium clone LM0ACA9ZC11FM1 genomic sequence
GKJWQY101BQAGF	532	4	471	3E-151	88%	88%	156770997	Bacteria	n	n	Uncultured bacterium clone LM0ABA6ZG02RM1 genomic sequence
GKJWQY101BTYHD	557	13	343	3E-96	87%	87%	156770867	Bacteria	n	n	Uncultured bacterium clone LM0ABA5ZA11RM1 genomic sequence
GKJWQY101A2KY0	541	18	484	2E-138	86%	86%	156775997	Bacteria	n	n	Uncultured bacterium clone LM0ACA3ZE01RM1 genomic sequence
GKJWQY101BXWSP	521	15	449	5E-94	82%	82%	156770975	Bacteria	n	n	Uncultured bacterium clone LM0ABA6ZD12RM1 genomic sequence
GKJWQY101A06LP	569	18	569	0	94%	94%	156768872	Bacteria	n	n	Uncultured bacterium clone LM0ABA5ZF07FM1 genomic sequence
GKJWQY101ACT2K	476	108	435	5E-98	87%	87%	156775471	Bacteria	n	n	Uncultured bacterium clone LM0ACA21ZE06RM1 genomic sequence
GKJWQY101A2BN5	538	10	424	2E-163	92%	92%	156768589	Bacteria	n	n	Uncultured bacterium clone LM0ABA40ZF05FM1 genomic sequence
GKJWQY101BL1SN	555	18	465	0	98%	98%	156772425	Bacteria	n	n	Uncultured bacterium clone LM0ACA2ZF10FM1 genomic sequence
GKJWQY101A24D1	418	3	362	6E-92	84%	84%	156773609	Bacteria	n	n	Uncultured bacterium clone LM0ACA8ZH09FM1 genomic sequence
GKJWQY101BNAXW	568	15	560	0	88%	88%	156774731	Bacteria	n	n	Uncultured bacterium clone LM0ACA1ZH04RM1 genomic sequence
GKJWQY101A2W2H	537	34	489	3E-161	90%	90%	156769665	Bacteria	n	n	Uncultured bacterium clone LM0ABA1ZF09RM1 genomic sequence
GKJWQY101A5D79	603	129	555	1E-160	91%	91%	156768004	Bacteria	n	n	Uncultured bacterium clone LM0ABA31ZH10FM1 genomic sequence
GKJWQY101BU6EL	555	14	548	0	93%	93%	156767585	Bacteria	n	n	Uncultured bacterium clone LM0ABA2ZC09FM1 genomic sequence
GKJWQY101A4300	358	5	306	1E-108	91%	91%	193083711	Bacteria	n	n	Uncultured bacterium AD12-C5 genomic sequence
GKJWQY101A91EV	205	20	160	5E-40	89%	89%	193084530	Bacteria	n	n	Uncultured bacterium ARCTIC38_F_05 genomic sequence
GKJWQY101ADWXG	416	27	347	7E-131	94%	94%	193084460	Bacteria	n	n	Uncultured bacterium ARCTIC47_D_06 genomic sequence
GKJWQY101AU0ML	283	7	239	4E-102	96%	96%	193084613	Bacteria	n	n	Uncultured bacterium HF0070_34E11 genomic sequence

GKJWQY101ANMYP	93	23	60	7E-10	100%	100%	193084646	Bacteria	n	n	Uncultured bacterium HF0200_39L23 genomic sequence
GKJWQY101AHOJC	335	1	273	1E-102	92%	92%	193084638	Bacteria	n	n	Uncultured bacterium HF4000_48A13 genomic sequence
GKJWQY101BI2JO	400	5	337	1E-104	88%	88%	193083728	Bacteria	n	n	Uncultured bacterium KM3-47-A6 genomic sequence
GKJWQY101ANG5G	545	17	544	0	89%	89%	62945638	Bacteria	n	n	Uncultured bacterium zdt-25h14 clone zdt-25h14, complete sequence
GKJWQY101A9FHD	225	5	141	2E-24	83%	83%	83595892	Bacteria	n	n	Uncultured marine bacterium Ant4E12, partial genomic sequence
GKJWQY101BLR88	506	5	501	0	97%	97%	146260146	Bacteria	n	n	Uncultured soil bacterium clone M53 similar to beta-lactamase genomic sequence
GKJWQY101AH7PE	431	9	388	3E-164	94%	94%	62860402	Bacteria	n	n	Uncultured bacterium zdt-44a23 clone zdt-44a23, complete sequence
GKJWQY101B18KD	574	14	492	8E-172	90%	90%	156777063	Bacteria	n	n	Uncultured bacterium clone HA0AAA17ZD02RM1 genomic sequence
GKJWQY101AM37K	544	86	508	8E-117	85%	85%	156769510	Bacteria	n	n	Uncultured bacterium clone HA0AAA7ZF01FM1 genomic sequence
GKJWQY101A17ME	572	18	570	0	90%	90%	156767597	Bacteria	n	n	Uncultured bacterium clone LM0ABA2ZD09FM1 genomic sequence
GKJWQY101AECX4	530	4	522	9E-176	88%	88%	156769699	Bacteria	n	n	Uncultured bacterium clone LM0ABA2ZE04RM1 genomic sequence
GKJWQY101BZ6F8	569	18	566	3E-176	88%	88%	156767618	Bacteria	n	n	Uncultured bacterium clone LM0ABA2ZG05FM1 genomic sequence
GKJWQY101A2QNX	544	7	423	6E-163	92%	92%	156767628	Bacteria	n	n	Uncultured bacterium clone LM0ABA2ZH11FM1 genomic sequence
GKJWQY101BM4AZ	443	18	341	2E-121	91%	91%	156768405	Bacteria	n	n	Uncultured bacterium clone LM0ABA39ZA06FM1 genomic sequence
GKJWQY101BNW14	408	20	353	6E-92	86%	86%	156770664	Bacteria	n	n	Uncultured bacterium clone LM0ABA40ZH07RM1 genomic sequence
GKJWQY101AXA5Z	445	53	386	8E-126	91%	91%	156770556	Bacteria	n	n	Uncultured bacterium clone LM0ABA4ZC09RM1 genomic sequence
GKJWQY101BWIJY	548	18	457	6E-118	85%	85%	156768500	Bacteria	n	n	Uncultured bacterium clone LM0ABA4ZD05FM1 genomic sequence
GKJWQY101AOB4M	528	193	432	6E-93	93%	93%	156770596	Bacteria	n	n	Uncultured bacterium clone LM0ABA4ZH01RM1 genomic sequence
GKJWQY101A8S8N	551	5	547	0	92%	92%	156770864	Bacteria	n	n	Uncultured bacterium clone LM0ABA5ZA06RM1 genomic sequence
GKJWQY101BSJWR	566	11	564	0	94%	94%	156768827	Bacteria	n	n	Uncultured bacterium clone LM0ABA5ZA12FM1 genomic sequence
GKJWQY101BVQQU	585	18	580	3E-166	86%	86%	156770902	Bacteria	n	n	Uncultured bacterium clone LM0ABA5ZE03RM1 genomic sequence
GKJWQY101BKAYD	576	4	571	0	93%	93%	156771060	Bacteria	n	n	Uncultured bacterium clone LM0ABA7ZF03RM1 genomic sequence
GKJWQY101BJH0Q	560	16	557	0	95%	95%	156769048	Bacteria	n	n	Uncultured bacterium clone LM0ABA8ZA06FM1 genomic sequence
GKJWQY101BD56T	570	62	565	0	95%	95%	156773064	Bacteria	n	n	Uncultured bacterium clone LM0ACA28ZD06FM1 genomic sequence
GKJWQY101BY5FD	536	3	531	0	92%	92%	156775970	Bacteria	n	n	Uncultured bacterium clone LM0ACA3ZB06RM1 genomic sequence
GKJWQY101AKOGB	575	18	572	2E-152	85%	85%	156776145	Bacteria	n	n	Uncultured bacterium clone LM0ACA5ZC04RM1 genomic sequence
GKJWQY101B1UIB	569	17	562	0	90%	90%	156776214	Bacteria	n	n	Uncultured bacterium clone LM0ACA6ZA05RM1 genomic sequence
GKJWQY101AXTWR	530	18	455	6E-128	86%	86%	156776216	Bacteria	n	n	Uncultured bacterium clone LM0ACA6ZA08RM1 genomic sequence
GKJWQY101AZQX2	535	18	529	0	92%	92%	156773440	Bacteria	n	n	Uncultured bacterium clone LM0ACA6ZH03FM1 genomic sequence
GKJWQY101BH4I5	552	2	550	7E-177	87%	87%	156773475	Bacteria	n	n	Uncultured bacterium clone LM0ACA7ZC10FM1 genomic sequence
GKJWQY101BYYVG	588	16	586	0	91%	91%	156773480	Bacteria	n	n	Uncultured bacterium clone LM0ACA7ZD04FM1 genomic sequence
GKJWQY101A407X	528	4	523	0	93%	93%	156776331	Bacteria	n	n	Uncultured bacterium clone LM0ACA7ZE02RM1 genomic sequence
GKJWQY101B1OHM	297	18	247	1E-112	99%	99%	193084596	Bacteria	n	n	Uncultured bacterium 2.6_D6 genomic sequence
GKJWQY101A41PE	569	7	567	0	89%	89%	193083707	Bacteria	n	n	Uncultured bacterium AD12-A11 genomic sequence
GKJWQY101BT4ZY	567	24	531	0	96%	96%	193083717	Bacteria	n	n	Uncultured bacterium AD84-H10 genomic sequence
GKJWQY101AWPDP	568	17	566	0	96%	96%	193084603	Bacteria	n	n	Uncultured bacterium HF0010_04H24 genomic sequence
GKJWQY101BKKTZ	579	21	575	0	89%	89%	193084608	Bacteria	n	n	Uncultured bacterium HF0010_10C01 genomic sequence
GKJWQY101BD5KR	585	18	585	0	99%	99%	193084619	Bacteria	n	n	Uncultured bacterium HF0500_12O04 genomic sequence
GKJWQY101BMECY	519	17	477	0	96%	96%	193084621	Bacteria	n	n	Uncultured bacterium HF0500_24B12 genomic sequence
GKJWQY101AH347	608	20	87	1E-20	96%	96%	193084601	Bacteria	n	n	Uncultured bacterium JM9_G5 genomic sequence
GKJWQY101BE4R0	362	215	242	0.001	100%	100%	91199943	Bacteria	Planctomycetes	Planctomycetacia	Kuenenia stuttgartiensis genome fragment KUST_E (5 of 5)
GKJWQY101BDY6H	568	20	550	1E-135	84%	84%	283436255	Bacteria	Planctomycetes	Planctomycetacia	Pirellula staleyi DSM 6068, complete genome
GKJWQY101AYMDQ	317	20	270	1E-102	94%	94%	67003493	Bacteria	Proteobacteria	Alphaproteobacteria	Brevundimonas sp. SD212 carotenoid biosynthesis gene cluster (orf1, crtW, crtY, crtI, crtB, orf6, orf7, crtE, idi, crtZ, crtG, orf12), cc
GKJWQY101A2HLQ	209	31	165	5E-55	96%	96%	240266805	Bacteria	Proteobacteria	Alphaproteobacteria	Bartonella grahamii as4aup, complete genome
GKJWQY101B0GZZ	540	171	203	0.000003	100%	100%	146189981	Bacteria	Proteobacteria	Alphaproteobacteria	Bradyrhizobium sp. ORS278,complete sequence
GKJWQY101BQDY9	536	18	476	0	92%	92%	219944660	Bacteria	Proteobacteria	Alphaproteobacteria	Methylobacterium nodulans ORS 2060, complete genome
GKJWQY101A4OMR	527	191	527	6E-118	90%	90%	111606883	Bacteria	Proteobacteria	Alphaproteobacteria	Aminobacter aminovorans partial gapA gene for glyceraldehyde-3-phosphate dehydrogenase, strain DSM 10368
GKJWQY101BV2AQ	541	123	216	2E-23	89%	89%	221739013	Bacteria	Proteobacteria	Alphaproteobacteria	Agrobacterium vitis S4 plasmid pAtS4e, complete sequence
GKJWQY101BU6BN	504	38	404	7E-112	87%	87%	86284380	Bacteria	Proteobacteria	Alphaproteobacteria	Rhizobium etli CFN 42 plasmid p42e, complete sequence
GKJWQY101AX6T2	494	18	391	1E-75	81%	81%	190694918	Bacteria	Proteobacteria	Alphaproteobacteria	Rhizobium etli CIAT 652, complete genome
GKJWQY101BERYH	547	24	479	6E-78	79%	79%	227339586	Bacteria	Proteobacteria	Alphaproteobacteria	Rhizobium sp. NGR234, complete genome
GKJWQY101BHVBO	505	5	505	1E-99	81%	81%	227337257	Bacteria	Proteobacteria	Alphaproteobacteria	Rhizobium sp. NGR234 plasmid pNGR234b, complete sequence
GKJWQY101BRU7O	505	21	489	1E-84	80%	80%	30407155	Bacteria	Proteobacteria	Alphaproteobacteria	Sinorhizobium meliloti 1021 complete chromosome
GKJWQY101BRQ7P	560	134	394	5E-64	85%	85%	25168258	Bacteria	Proteobacteria	Alphaproteobacteria	Sinorhizobium meliloti 1021 plasmid pSymA, complete sequence
GKJWQY101ARU2K	500	434	480	0.0000002	91%	91%	158328513	Bacteria	Proteobacteria	Alphaproteobacteria	Azorhizobium caulinodans ORS 571 DNA, complete genome
GKJWQY101AJPHZ	518	63	472	4E-50	77%	77%	119372524	Bacteria	Proteobacteria	Alphaproteobacteria	Paracoccus denitrificans PD1222 chromosome 1, complete sequence
GKJWQY101AGSJB	496	5	429	0	99%	99%	32263857	Bacteria	Proteobacteria	Alphaproteobacteria	Paracoccus sp. Ol18 plasmid pOL18 rrn operon, complete sequence
GKJWQY101BYMZF	548	17	68	4E-10	93%	93%	221161990	Bacteria	Proteobacteria	Alphaproteobacteria	Rhodobacter sphaeroides KD131 chromosome 2, complete sequence
GKJWQY101ATYDP	385	15	328	7E-136	95%	95%	56676665	Bacteria	Proteobacteria	Alphaproteobacteria	Ruegeria pomeroyi DSS-3, complete genome
GKJWQY101BSXCF	501	54	453	4E-134	89%	89%	42414566	Bacteria	Proteobacteria	Alphaproteobacteria	Acetobacter pasteurianus partial ITS1, strain CCM 3606
GKJWQY101AL6DG	481	5	436	3E-130	87%	87%	82943940	Bacteria	Proteobacteria	Alphaproteobacteria	Magnetospirillum magneticum AMB-1 DNA, complete genome
GKJWQY101ATZNT	193	44	158	1E-50	99%	99%	288926859	Bacteria	Proteobacteria	Alphaproteobacteria	Rhodospirillum centenum SW, complete genome
GKJWQY101BUXBD	461	372	459	4E-10	82%	82%	145322317	Bacteria	Proteobacteria	Alphaproteobacteria	Novosphingobium aromaticivorans DSM 12444 plasmid pNL2, complete sequence
GKJWQY101BRVHF	354	21	316	1E-142	98%	98%	188532098	Bacteria	Proteobacteria	Alphaproteobacteria	Sphingobium chungbukense rrnC operon, complete sequence
GKJWQY101A14H0	579	23	576	0	91%	91%	170658659	Bacteria	Proteobacteria	Alphaproteobacteria	Methylobacterium radiotolerans JCM 2831 plasmid pMRAD01, complete sequence
GKJWQY101AHOIZ	503	10	497	9E-176	90%	90%	47118328	Bacteria	Proteobacteria	Alphaproteobacteria	Mesorhizobium loti MAFF303099 DNA, complete genome
GKJWQY101BSA8U	613	19	610	0	91%	91%	119376152	Bacteria	Proteobacteria	Alphaproteobacteria	Paracoccus denitrificans PD1222 chromosome 2, complete sequence
GKJWQY101BM00X	551	21	550	0	91%	91%	83574254	Bacteria	Proteobacteria	Alphaproteobacteria	Rhodospirillum rubrum ATCC 11170, complete genome
GKJWQY101ASBL2	563	18	545	0	96%	96%	292676846	Bacteria	Proteobacteria	Alphaproteobacteria	Sphingobium japonicum UT26S DNA, chromosome 2, complete genome
GKJWQY101BATJM	570	18	563	0	97%	97%	98975575	Bacteria	Proteobacteria	Alphaproteobacteria	Sphingopyxis alaskensis RB2256, complete genome
GKJWQY101BQWCO	376	66	220	8E-11	76%	76%	115421100	Bacteria	Proteobacteria	Betaproteobacteria	Bordetella avium 197N complete genome
GKJWQY101ASXGJ	515	371	468	2E-17	85%	85%	38707361	Bacteria	Proteobacteria	Betaproteobacteria	Burkholderia anthina pANT1 plasmid ORF3 for hypothetical protein, strain BHS1

GKJWQY101BJCLG	511	155	511	0	100%	100%	13625778	Bacteria	Proteobacteria	Betaproteobacteria	Burkholderia cepacia acyl-homoserine lactone synthase BviI (bviI) and transcriptional activator BviR (bviR) genes, complete cds
GKJWQY101AL5GX	499	279	313	0.00001	97%	97%	126238441	Bacteria	Proteobacteria	Betaproteobacteria	Burkholderia mallei NCTC 10247 chromosome II, complete sequence
GKJWQY101BS6QI	486	17	453	0	95%	95%	237502667	Bacteria	Proteobacteria	Betaproteobacteria	Burkholderia pseudomallei MSHR346 chromosome I, complete sequence
GKJWQY101BN6XJ	105	5	67	5E-17	94%	94%	295438061	Bacteria	Proteobacteria	Betaproteobacteria	Burkholderia sp. CCGE1002 chromosome 2, complete sequence
GKJWQY101BCW7I	562	74	193	3E-16	81%	81%	295434944	Bacteria	Proteobacteria	Betaproteobacteria	Burkholderia sp. CCGE1002 chromosome 1, complete sequence
GKJWQY101BXETD	510	18	215	1E-39	82%	82%	83649860	Bacteria	Proteobacteria	Betaproteobacteria	Burkholderia thailandensis E264 chromosome II, complete sequence
GKJWQY101AHRMM	534	5	308	9E-32	77%	77%	83652219	Bacteria	Proteobacteria	Betaproteobacteria	Burkholderia thailandensis E264 chromosome I, complete sequence
GKJWQY101BS6LH	508	5	505	1E-144	85%	85%	91685338	Bacteria	Proteobacteria	Betaproteobacteria	Burkholderia xenovorans LB400 chromosome 1, complete sequence
GKJWQY101BQ51J	519	141	502	7E-18	73%	73%	171192370	Bacteria	Proteobacteria	Betaproteobacteria	Polynucleobacter necessarius subsp. necessarius STIR1, complete genome
GKJWQY101AK7G8	502	18	469	9E-106	83%	83%	206593202	Bacteria	Proteobacteria	Betaproteobacteria	Ralstonia solanacearum strain IPO1609 Genome Draft
GKJWQY101BIKNE	401	18	348	1E-153	97%	97%	120587178	Bacteria	Proteobacteria	Betaproteobacteria	Acidovorax citrulli AAC00-1, complete genome
GKJWQY101AJROG	413	25	376	1E-133	92%	92%	121551644	Bacteria	Proteobacteria	Betaproteobacteria	Verminephrobacter eiseniae EF01-2, complete genome
GKJWQY101B2KTE	243	4	129	8E-49	95%	95%	295794626	Bacteria	Proteobacteria	Betaproteobacteria	Thiomonas intermedia K12, complete genome
GKJWQY101AW7FS	510	61	278	2E-37	81%	81%	294338440	Bacteria	Proteobacteria	Betaproteobacteria	Thiomonas sp. str. 3As, chromosome, complete genome
GKJWQY101BKTPD	537	17	533	0	93%	93%	48428765	Bacteria	Proteobacteria	Betaproteobacteria	Collimonas fungivorans fosmid CFUFOS26, complete sequence
GKJWQY101A7HUP	554	10	508	0	93%	93%	34105712	Bacteria	Proteobacteria	Betaproteobacteria	Chromobacterium violaceum ATCC 12472, complete genome
GKJWQY101BOC0B	511	18	391	4E-179	98%	98%	66731897	Bacteria	Proteobacteria	Betaproteobacteria	Neisseria meningitidis MC58, complete genome
GKJWQY101AXXBZ	498	5	442	0	98%	98%	161594571	Bacteria	Proteobacteria	Betaproteobacteria	Neisseria meningitidis 053442, complete genome
GKJWQY101BYX8F	547	24	547	0	96%	96%	254667570	Bacteria	Proteobacteria	Betaproteobacteria	Neisseria meningitidis alpha14 complete genome
GKJWQY101A4LWM	503	18	500	0	92%	92%	77964193	Bacteria	Proteobacteria	Betaproteobacteria	Burkholderia sp. 383 chromosome 3, complete sequence
GKJWQY101B17TY	497	18	341	2E-151	97%	97%	77965403	Bacteria	Proteobacteria	Betaproteobacteria	Burkholderia sp. 383 chromosome 1, complete sequence
GKJWQY101AGP17	539	17	500	0	97%	97%	77968738	Bacteria	Proteobacteria	Betaproteobacteria	Burkholderia sp. 383, complete sequence
GKJWQY101ATCIB	517	18	514	0	96%	96%	295441430	Bacteria	Proteobacteria	Betaproteobacteria	Burkholderia sp. CCGE1002 plasmid pBC201, complete sequence
GKJWQY101AD239	350	18	201	2E-90	100%	100%	288237308	Bacteria	Proteobacteria	Betaproteobacteria	Cupriavidus metallidurans CH34 megaplasmid, complete sequence
GKJWQY101AG8TI	459	5	405	2E-102	84%	84%	30407127	Bacteria	Proteobacteria	Betaproteobacteria	Ralstonia solanacearum GMI1000 chromosome complete sequence
GKJWQY101AX6PV	517	19	513	3E-151	87%	87%	30407128	Bacteria	Proteobacteria	Betaproteobacteria	Ralstonia solanacearum GMI1000 megaplasmid complete sequence
GKJWQY101BLFSC	544	18	528	0	95%	95%	221728669	Bacteria	Proteobacteria	Betaproteobacteria	Acidovorax ebreus TPSY, complete genome
GKJWQY101AQ5RN	548	18	545	0	91%	91%	120604516	Bacteria	Proteobacteria	Betaproteobacteria	Acidovorax sp. JS42, complete genome
GKJWQY101AEFDT	566	24	563	0	96%	96%	91695138	Bacteria	Proteobacteria	Betaproteobacteria	Polaromonas sp. JS666, complete genome
GKJWQY101BLFW7	540	18	534	0	90%	90%	133737197	Bacteria	Proteobacteria	Betaproteobacteria	Herminiimonas arsenicoxydans chromosome, complete sequence
GKJWQY101AVDT3	547	51	545	1E-159	88%	88%	71845263	Bacteria	Proteobacteria	Betaproteobacteria	Dechloromonas aromatica RCB, complete genome
GKJWQY101B3I7Y	506	18	506	0	98%	98%	186660182	Bacteria	Proteobacteria	Betaproteobacteria	Burkholderia vietnamiensis strain CBMB40 nitrogenase molybdenum-iron protein beta chain-like (nifK) gene, partial sequence
GKJWQY101BISXY	575	18	571	0	99%	99%	260222220	Bacteria	Proteobacteria	Betaproteobacteria	Curvibacter putative symbiont of Hydra magnipapillata genomic scaffold HmaUn_WGA71069_1
GKJWQY101A2RQ4	553	5	551	1E-150	85%	85%	90823168	Bacteria	Proteobacteria	Deltaproteobacteria	Pelobacter carbinolicus DSM 2380, complete genome
GKJWQY101AVQQS	550	11	275	8E-112	95%	95%	262076673	Bacteria	Proteobacteria	Deltaproteobacteria	Haliangium ochraceum DSM 14365, complete genome
GKJWQY101A7KK6	487	18	272	9E-91	91%	91%	219952977	Bacteria	Proteobacteria	Deltaproteobacteria	Anaeromyxobacter dehalogenans 2CP-1, complete genome
GKJWQY101AN6A1	562	23	555	2E-133	84%	84%	82617838	Bacteria	Proteobacteria	Deltaproteobacteria	Uncultured delta proteobacterium DeepAnt-32C6 fosmid, complete sequence
GKJWQY101ATI2M	440	5	407	5E-103	84%	84%	78217452	Bacteria	Proteobacteria	Deltaproteobacteria	Desulfovibrio alaskensis G20, complete genome
GKJWQY101A2EXF	323	32	265	1E-72	88%	88%	34483186	Bacteria	Proteobacteria	Epsilonproteobacteria	Wolinella succinogenes, complete genome; segment 4/7
GKJWQY101AR8JF	379	18	332	1E-137	95%	95%	237499037	Bacteria	Proteobacteria	Gammaproteobacteria	Tolumonas auensis DSM 9187, complete genome
GKJWQY101ACR3I	481	18	416	5E-158	92%	92%	120322793	Bacteria	Proteobacteria	Gammaproteobacteria	Marinobacter aquaeolei VT8, complete genome
GKJWQY101AML8K	373	18	335	8E-135	94%	94%	167351963	Bacteria	Proteobacteria	Gammaproteobacteria	Shewanella halifaxensis HAW-EB4, complete genome
GKJWQY101A8SDV	547	23	460	0	98%	98%	117610791	Bacteria	Proteobacteria	Gammaproteobacteria	Shewanella sp. ANA-3, complete genome
GKJWQY101BJ0XY	460	5	399	6E-127	88%	88%	219994503	Bacteria	Proteobacteria	Gammaproteobacteria	Thioalkalivibrio sp. HL-EbGR7, complete genome
GKJWQY101BMVH7	413	12	299	2E-122	95%	95%	261835099	Bacteria	Proteobacteria	Gammaproteobacteria	Halothiobacillus neapolitanus c2, complete genome
GKJWQY101ADZWX	379	7	356	2E-115	89%	89%	145316543	Bacteria	Proteobacteria	Gammaproteobacteria	Enterobacter sp. 638, complete genome
GKJWQY101A5AS0	229	5	197	3E-77	94%	94%	291551905	Bacteria	Proteobacteria	Gammaproteobacteria	Erwinia amylovora CFBP1430 complete genome
GKJWQY101AF69O	551	4	548	0	97%	97%	291197582	Bacteria	Proteobacteria	Gammaproteobacteria	Erwinia amylovora ATCC 49946 chromosomal sequence
GKJWQY101AYZFU	278	24	176	2E-41	88%	88%	150953431	Bacteria	Proteobacteria	Gammaproteobacteria	Klebsiella pneumoniae subsp. pneumoniae MGH 78578, complete sequence
GKJWQY101AULKC	321	5	36	0.000007	100%	100%	295647398	Bacteria	Proteobacteria	Gammaproteobacteria	Legionella pneumophila 2300/99 Alcoy, complete genome
GKJWQY101AOYQY	489	4	456	0	98%	98%	53749768	Bacteria	Proteobacteria	Gammaproteobacteria	Legionella pneumophila str. Paris complete genome
GKJWQY101BJB7H	400	5	271	4E-103	93%	93%	256794767	Bacteria	Proteobacteria	Gammaproteobacteria	Kangiella koreensis DSM 16069, complete genome
GKJWQY101ASOB7	495	119	292	1E-29	82%	82%	83630956	Bacteria	Proteobacteria	Gammaproteobacteria	Hahella chejuensis KCTC 2396, complete genome
GKJWQY101A55T9	550	25	496	5E-149	87%	87%	261412053	Bacteria	Proteobacteria	Gammaproteobacteria	Aggregatibacter actinomycetemcomitans D11S-1, complete genome
GKJWQY101AQVXX	253	21	216	5E-61	89%	89%	247533203	Bacteria	Proteobacteria	Gammaproteobacteria	Aggregatibacter aphrophilus NJ8700, complete genome
GKJWQY101BJFYS	507	5	462	0	99%	99%	108733343	Bacteria	Proteobacteria	Gammaproteobacteria	Pseudomonas syringae pv. syringae strain UMAF0158 mangotoxin production gene cluster and rrn operon, partial sequence
GKJWQY101AZO3V	560	18	452	0	95%	95%	49529273	Bacteria	Proteobacteria	Gammaproteobacteria	Acinetobacter sp. ADP1 complete genome
GKJWQY101AX65L	589	5	588	0	93%	93%	71037566	Bacteria	Proteobacteria	Gammaproteobacteria	Psychrobacter arcticus 273-4, complete genome
GKJWQY101ALTT0	576	18	572	0	97%	97%	148570901	Bacteria	Proteobacteria	Gammaproteobacteria	Psychrobacter sp. PRwf-1, complete genome
GKJWQY101ASWG9	515	17	430	0	97%	97%	226717097	Bacteria	Proteobacteria	Gammaproteobacteria	Azotobacter vinelandii DJ, complete genome
GKJWQY101ACB0K	278	18	227	1E-77	93%	93%	190684944	Bacteria	Proteobacteria	Gammaproteobacteria	Cellvibrio japonicus Ueda107, complete genome
GKJWQY101ARN9L	358	17	310	4E-148	99%	99%	63253978	Bacteria	Proteobacteria	Gammaproteobacteria	Pseudomonas syringae pv. syringae B728a, complete genome
GKJWQY101A9KUB	568	5	556	0	91%	91%	283472039	Bacteria	Proteobacteria	Gammaproteobacteria	Xanthomonas albilineans str. GPE PC73, chromosome, complete genome
GKJWQY101AGMLE	576	20	556	1E-155	86%	86%	117607074	Bacteria	Proteobacteria	n	Magnetococcus sp. MC-1, complete genome
GKJWQY101ALGOA	548	18	548	0	90%	90%	226525288	Bacteria	Verrucomicrobia	n	Uncultured Verrucomicrobia bacterium clone 118 genomic sequence
GKJWQY101BWYXQ	338	8	275	5E-82	88%	88%	187424568	Bacteria	Verrucomicrobia	Verrucomicrobiae	Akkermansia muciniphila ATCC BAA-835, complete genome
