# Supplementary material for: Subglacial Lake Vostok (Antarctica) Accretion Ice Contains a Diverse Set of Sequences from Aquatic, Marine and Sediment-Inhabiting Bacteria and Eukarya
Source: PLoS One. 2013 Jul 3;8(7):e67221. doi: 10.1371/journal.pone.0067221 (PMC3700977; doi:10.1371/journal.pone.0067221)
Supplement: Table S5 — Eukarya mRNA (and other non-rRNA) gene sequences from V5. [“n” indicates information not specified in the NCBI GenBank database.]. (PDF) [file pone.0067221.s010.pdf]

Table S5. Eukarya mRNA (and other non-rRNA) gene sequences from V5. ["n" indicates information not specified in the NCBI GenBank database.]

454 Sequence ID	Q length	Q start	Q end	e-value	%-ident	%-sim	GI number	Domain	Phylum	Class / Order	Genus / Species	Description
GKJWQY101B20DL	80	1	80	3E-33	100%	100%	294935818	Eukaryota	Incertae sedis	Perkinsea	Perkinsus marinus	Perkinsus marinus ATCC 50983 hypothetical protein, mRNA
GKJWQY101AEUTS	128	1	128	6E-53	97%	97%	50657597	Eukaryota	Rhodophyta	Florideophyceae	Gracilaria tenuistipitata	Gracilaria tenuistipitata var. liui chloroplast, complete genome
GKJWQY101AV8WV	332	1	332	2E-161	98%	98%	294661761	Eukaryota	Arthropoda	Branchiopoda	Daphnia pulex	Daphnia pulex ncRNA, kairomone-inducible transcript
GKJWQY101ACXJK	411	1	411	0	95%	95%	195094333	Eukaryota	Arthropoda	Insecta	Drosophila grimshawi	Drosophila grimshawi GH23874 (Dgri\GH23874), mRNA
GKJWQY101BDI9Y	393	1	393	0	96%	96%	195102030	Eukaryota	Arthropoda	Insecta	Drosophila grimshawi	Drosophila grimshawi GH23709 (Dgri\GH23709), mRNA
GKJWQY101BCYFO	306	1	306	3E-141	97%	97%	295393587	Eukaryota	Arthropoda	Insecta	Euphydryas editha	Euphydryas editha clone euphy 50 microsatellite sequence
GKJWQY101B0QOY	266	1	266	3E-84	88%	88%	153902485	Eukaryota	Arthropoda	Insecta	Gryllus bimaculatus	Gryllus bimaculatus mRNA, GBcontig04373
GKJWQY101B09E4	370	1	112	1E-25	88%	88%	153919430	Eukaryota	Arthropoda	Insecta	Gryllus bimaculatus	Gryllus bimaculatus mRNA, GBcontig29766
GKJWQY101AKNUG	492	1	492	6E-104	82%	82%	67540319	Eukaryota	Ascomycota	Eurotiomycetes	Emericella nidulans	Aspergillus nidulans FGSC A4 elongation factor 2 partial mRNA
GKJWQY101BA6T8	556	1	556	0	95%	95%	242805358	Eukaryota	Ascomycota	Eurotiomycetes	Talaromyces stipitatus	Talaromyces stipitatus ATCC 10500 hypothetical protein, mRNA
GKJWQY101A5AN6	72	1	72	1E-18	92%	92%	50542891	Eukaryota	Ascomycota	Saccharomycetes	Yarrowia lipolytica	Yarrowia lipolytica YALI0A00352p (YALI0A00352g) mRNA, complete cds
GKJWQY101BK6ZF	47	1	47	1E-09	94%	94%	49343295	Eukaryota	Ascomycota	Saccharomycetes	Kluyveromyces lactis	Kluyveromyces lactis mitochondrion, complete genome
GKJWQY101ATCHW	494	1	494	0	99%	99%	238033210	Eukaryota	Ascomycota	Saccharomycetes	Pichia pastoris	Pichia pastoris GS115 chromosome 4, complete sequence
GKJWQY101AFQK1	28	1	28	0.000005	100%	100%	259145041	Eukaryota	Ascomycota	Saccharomycetes	Saccharomyces cerevisiae	Saccharomyces cerevisiae EC1118 chromosome IV, EC1118_1D0 genomic scaffold, whole genome shotgun sequence
GKJWQY101A475X	79	1	79	1E-32	100%	100%	259147625	Eukaryota	Ascomycota	Saccharomycetes	Saccharomyces cerevisiae	Saccharomyces cerevisiae EC1118 chromosome XI, EC1118_1K5 genomic scaffold, whole genome shotgun sequence
GKJWQY101A88RH	115	1	115	2E-47	97%	97%	259147931	Eukaryota	Ascomycota	Saccharomycetes	Saccharomyces cerevisiae	Saccharomyces cerevisiae EC1118 chromosome XII, EC1118_1L10 genomic scaffold, whole genome shotgun sequence
GKJWQY101AQT0M	135	1	135	4E-51	94%	94%	259149327	Eukaryota	Ascomycota	Saccharomycetes	Saccharomyces cerevisiae	Saccharomyces cerevisiae EC1118 chromosome XV, EC1118_1O4 genomic scaffold, whole genome shotgun sequence
GKJWQY101BK6ZF_2	140	1	140	2E-19	78%	78%	23630288	Eukaryota	Basidiomycota	Tremellomycetes	Cryptococcus neoformans	Cryptococcus neoformans var. grubii strain H99 mitochondrion, complete genome
GKJWQY101BK6ZF_3	151	1	151	5E-15	78%	78%	315465309	Eukaryota	Basidiomycota	Ustilaginomycetes	Sporisorium	Sporisorium reilianum SRZ2 mitochondrial DNA sequence
GKJWQY101ANHKA	513	1	513	0	91%	91%	71483398	Eukaryota	Basidiomycota	Microbotryales	Microbotryum violaceum	Microbotryum violaceum isolate Dsylvestris_9119 elongation factor 1alpha (EF1) gene, partial cds
GKJWQY101B0NNM	284	1	284	7E-116	94%	94%	171191502	Eukaryota	Cercozoa	Imbricatea	Paulinella chromatophora	Paulinella chromatophora chromatophore, complete genome
GKJWQY101BSD9R	103	1	100	2E-42	99%	99%	56159573	Eukaryota	Chlorophyta	Ulvophyceae	Pseudendoclonium akinetum	Pseudendoclonium akinetum chloroplast, complete genome
GKJWQY101A15DF	332	1	332	2E-47	78%	78%	156307499	Eukaryota	Cnidaria	Anthozoa	Nematostella vectensis	Nematostella vectensis predicted protein (NEMVEDRAFT_v1g225918) partial mRNA
GKJWQY101AR687	53	1	53	3E-15	96%	96%	253981836	Eukaryota	Cryptophyta	Cryptophyceae	Cryptomonas paramecium	Cryptomonas paramecium strain CCAP977/2a plastid, complete genome
GKJWQY101AY5N9	185	1	185	4E-91	100%	100%	166044520	Eukaryota	Heterokontophyta	Oomycota	Aphanomyces euteiches	Aphanomyces euteiches cDNA
GKJWQY101BRT6Z	395	1	395	0	98%	98%	166044537	Eukaryota	Heterokontophyta	Oomycota	Aphanomyces euteiches	Aphanomyces euteiches cDNA
GKJWQY101AJXX3	28	1	28	0.000005	100%	100%	336170404	Eukaryota	Kinetoplastida	Trypanosomatidae	Schizotrypanum	Trypanosoma cruzi clone IIe_16 SMUG S protein gene, partial cds
GKJWQY101A8PWD	362	1	362	2E-177	98%	98%	154347362	Eukaryota	Percolozoa	Heterolobosea	Naegleria gruberi	Naegleria gruberi extrachromosomal rDNA plasmid DNA, complete sequence
GKJWQY101ASKP6	96	1	96	1E-38	98%	98%	124012151	Eukaryota	Streptophyta	Chlorokybophyceae	Chlorokybus atmophyticus	Chlorokybus atmophyticus chloroplast, complete genome
GKJWQY101AZ7HH	105	1	105	2E-37	94%	94%	210148086	Eukaryota	Streptophyta	Cornales	Cornus kousa	Cornus kousa clone CK083 microsatellite sequence
GKJWQY101BH3Q0	218	1	218	7E-45	83%	83%	13445170	Eukaryota	Streptophyta	Liliopsida	Festuca arundinacea	Festuca arundinacea genomic FaA5 repeat element
GKJWQY101ABSK7	442	1	442	0	93%	93%	21779916	Eukaryota	Streptophyta	Liliopsida	Aegilops tauschii	Aegilops tauschii leucine-rich-like protein gene, partial cds; seed globulin (Glo-2) gene, complete cds; HMW-glutenin (Glu-Dty) gene, Glu-Dty-T2 allele, complete cds; and HMW-glutenin (HMW) gene, HMW-Dtx2 allele, complete cds
GKJWQY101BPH2R	480	1	480	2E-109	83%	83%	32307244	Eukaryota	Streptophyta	Liliopsida	Aegilops tauschii	Aegilops tauschii clone 4P6-2 tandem repeat sequence
GKJWQY101AJYHQ	243	1	243	3E-108	97%	97%	40849982	Eukaryota	Streptophyta	Liliopsida	Triticum turgidum	Triticum turgidum A genome HMW glutenin A gene locus, sequence
GKJWQY101AXHYK	81	1	81	2E-26	95%	95%	72256311	Eukaryota	Streptophyta	Liliopsida	Triticum aestivum	Triticum aestivum clone wmc(3d2) microsatellite sequence
GKJWQY101BH96M	334	1	334	6E-63	83%	83%	102567891	Eukaryota	Streptophyta	Liliopsida	Zea perennis	Zea perennis mitochondrion, complete genome
GKJWQY101AQJ6W	300	1	299	2E-113	92%	92%	115392331	Eukaryota	Streptophyta	Liliopsida	Triticum monococcum	Triticum monococcum subsp. aegilopoides clone BAC TbBAC5, complete sequence
GKJWQY101AQJ6W_2	181	1	181	5E-70	94%	94%	115392331	Eukaryota	Streptophyta	Liliopsida	Triticum monococcum	Triticum monococcum subsp. aegilopoides clone BAC TbBAC5, complete sequence
GKJWQY101BYWC5	492	1	492	0	91%	91%	124007144	Eukaryota	Streptophyta	Liliopsida	Triticum urartu	Triticum urartu clone BAC 41C8 genomic sequence
GKJWQY101ANTJW	285	1	285	6E-131	97%	97%	148372275	Eukaryota	Streptophyta	Liliopsida	Triticum monococcum	Triticum monococcum subsp. aegilopoides clone TbBAC30, complete sequence
GKJWQY101BYJS2	492	1	492	0	99%	99%	148910867	Eukaryota	Streptophyta	Liliopsida	Triticum turgidum	Triticum turgidum overlapping clones BAC 326E2 and BAC 354M17, complete sequence
GKJWQY101AQ547	348	1	348	3E-100	87%	87%	194131647	Eukaryota	Streptophyta	Liliopsida	Triticum turgidum	Triticum turgidum subsp. dicoccoides clones BAC 391M13 and BAC 1144M20 genomic sequence
GKJWQY101BU9TF	262	1	262	2E-106	94%	94%	194239068	Eukaryota	Streptophyta	Liliopsida	Triticum aestivum	Triticum aestivum 3B chromosome, clone BAC TA3B95C9
GKJWQY101BF6SX	432	1	432	0	100%	100%	209361311	Eukaryota	Streptophyta	Liliopsida	Coix lacryma-jobi	Coix lacryma-jobi chloroplast, complete genome
GKJWQY101AOCWM	154	1	154	2E-13	77%	77%	212007811	Eukaryota	Streptophyta	Liliopsida	Triticum aestivum	Triticum aestivum clone BAC 502E09, complete sequence
GKJWQY101BW1XB	383	1	383	0	99%	99%	255099160	Eukaryota	Streptophyta	Liliopsida	Dendrocalamus latiflorus	Dendrocalamus latiflorus chloroplast, complete genome
GKJWQY101AN3LX	127	1	127	2E-57	99%	99%	219819090	Eukaryota	Streptophyta	Apiales	Daucus carota	Daucus carota subsp. sativus clone BAC C034E06 genomic sequence
GKJWQY101BVC5O	578	1	578	2E-123	83%	83%	88656961	Eukaryota	Streptophyta	Asterales	Lactuca sativa	Lactuca sativa cultivar Salinas chloroplast, complete genome
GKJWQY101BNTYY	300	273	300	0.001	100%	100%	259526188	Eukaryota	Streptophyta	Asterales	Artemisia annua	TSA: Artemisia annua strain Artemis Contig17, mRNA sequence
GKJWQY101AZBHB	232	1	232	1E-107	98%	98%	194132090	Eukaryota	Streptophyta	Malvales	Gonystylus	Gonystylus bancanus chloroplast, partial genome
GKJWQY101AYF7P	275	1	275	3E-139	99%	99%	170522360	Eukaryota	Streptophyta	Brassicales	Carica papaya	Carica papaya mitochondrion, complete genome
GKJWQY101ADA3T	198	1	198	1E-96	99%	99%	62149314	Eukaryota	Streptophyta	Caryophyllales	Silene latifolia	Silene latifolia chloroplast rpoB, rpoC1, rpoC2, rps2, atpI, atpH, atpF, atpA, psbI, psbK, rps16, matK, psbA, rpl2, ycf2, ndhB, rps7 genes, complete cds
GKJWQY101APA8N	428	1	428	0	94%	94%	193788921	Eukaryota	Streptophyta	Fabales	Trifolium subterraneum	Trifolium subterraneum chloroplast, complete genome
GKJWQY101A4746	335	1	335	2E-152	96%	96%	293338622	Eukaryota	Streptophyta	Fabales	Lathyrus sativus	Lathyrus sativus cultivar Cicerchia Marchigiana chloroplast, complete genome
GKJWQY101ATFGY	514	1	514	0	93%	93%	210143279	Eukaryota	Streptophyta	Fabales	Glycine max	Glycine max cDNA, clone: GMFL01-21-C10
GKJWQY101BGGKO	394	1	394	1E-159	93%	93%	218135405	Eukaryota	Streptophyta	Fabales	Medicago truncatula	Medicago truncatula chromosome 4 clone mth2-135p20, complete sequence
GKJWQY101A1C94	345	1	345	4E-178	99%	99%	329124647	Eukaryota	Streptophyta	Solanales	Solanum	Solanum tuberosum isolate DM1-3-516-R44 chloroplast, complete genome
GKJWQY101BOE3Q	467	1	467	0	96%	96%	147776538	Eukaryota	Streptophyta	Vitales	Vitis vinifera	Vitis vinifera contig VV78X160272.3, whole genome shotgun sequence
GKJWQY101BYF2M	52	1	52	5E-13	94%	94%	147820696	Eukaryota	Streptophyta	Vitales	Vitis vinifera	Vitis vinifera contig VV78X065077.2, whole genome shotgun sequence
GKJWQY101BTYU0	537	1	537	0	99%	99%	239764707	Eukaryota	Streptophyta	Vitales	Vitis vinifera	Vitis vinifera strain PN40024 mitochondrion, partial genome
