# Supplementary material for: Subglacial Lake Vostok (Antarctica) Accretion Ice Contains a Diverse Set of Sequences from Aquatic, Marine and Sediment-Inhabiting Bacteria and Eukarya
Source: PLoS One. 2013 Jul 3;8(7):e67221. doi: 10.1371/journal.pone.0067221 (PMC3700977; doi:10.1371/journal.pone.0067221)
Supplement: Table S6 — Sequences from Archaea and Viruses in V5. [“n” indicates information not specified in the NCBI GenBank database.]. (PDF) [file pone.0067221.s011.pdf]

Table S6. Sequences from Archaea and Viruses V5. ["n" indicates information not specified in the NCBI GenBank database.]

454 Sequence ID	Q length	Q start	Q end	e-value	%-ident	%-sim	GI number	Kingdom	Order	Family	Genus
GKJWQY101A0CZO	303	20	258	1E-117	99%	99%	207366080	Archaea	n	n	n
GKJWQY101A62OT	539	33	61	0.0005	100%	100%	262527001	Archaea	n	n	n
GKJWQY101A8DGR	536	17	533	0	98%	98%	288872851	Viruses	n	Microviridae	Microvirus
GKJWQY101AB5CE	552	18	547	1E-84	79%	79%	91982906	Viruses	Caudovirales	Siphoviridae	n

Description
"Uncultured archaeon partial 16S rRNA gene, clone ODP204_30_Bac263"
"uncultured archaeon ANME-1, unordered contigs"
"Enterobacteria phage phiX174 isolate JACSK, complete genome"
"Propionibacterium phage PA6, complete genome"
