# Supplementary material for: Subglacial Lake Vostok (Antarctica) Accretion Ice Contains a Diverse Set of Sequences from Aquatic, Marine and Sediment-Inhabiting Bacteria and Eukarya
Source: PLoS One. 2013 Jul 3;8(7):e67221. doi: 10.1371/journal.pone.0067221 (PMC3700977; doi:10.1371/journal.pone.0067221)
Supplement: Table S7 — Small subunit rRNA gene sequences of Bacteria and Eukarya from V6. [“n” indicates information not specified in the NCBI GenBank database.]. (PDF) [file pone.0067221.s012.pdf]

Table S7. Small subunit rRNA gene sequences of Bacteria and Eukarya from V6. ["n" indicates information not specified in the NCBI GenBank database.]

Accession number	Q length	Q start	Q end	e-value	%-ident	%-sim	GI number	Domain	Phylum	Class	Genus / Species	Description
JQ999507	239	1	239	1E-110	97%	97%	38195134	Bacteria	Actinobacteria	Actinobacteria	uncultured actinobacterium	Uncultured actinobacterium clone H5Ba05 small subunit ribosomal RNA gene, partial sequence
JQ999509	312	1	312	1E-157	99%	99%	154184543	Bacteria	Firmicutes	Clostridia	uncultured Lachnospiraceae bacterium	Uncultured Lachnospiraceae bacterium clone MS167A1_G05 16S ribosomal RNA gene, partial sequence
JQ999560	228	1	228	8E-113	100%	100%	295443962	Bacteria	Firmicutes	Bacilli	Staphylococcus sp. NCCP-163	Staphylococcus sp. NCCP-163 gene for 16S rRNA, partial sequence
JQ999508	274	1	272	3E-118	93%	93%	295815580	Bacteria	Firmicutes	Bacilli	Lactococcus lactis	Lactococcus lactis subsp. lactis strain KLC02 16S ribosomal RNA gene, partial sequence
JQ999559	247	1	247	2E-120	99%	99%	294337929	Bacteria	Firmicutes	Bacilli	Bacillus clausii	Bacillus clausii partial 16S rRNA gene, strain 3LF 22P
JQ999534	593	3	514	0	98%	98%	110448355	Bacteria	n	n	uncultured bacterium	Uncultured bacterium clone RL239_aaj10c07 16S ribosomal RNA gene, partial sequence
JQ999535	666	1	666	0	94%	94%	237774881	Bacteria	n	n	uncultured bacterium	Uncultured bacterium clone MONS_SW1105_60 16S ribosomal RNA gene, partial sequence
JQ999516	245	1	245	3E-122	100%	100%	70959311	Bacteria	n	n	uncultured bacterium	Uncultured bacterium clone C5-55 16S ribosomal RNA gene, partial sequence
JQ999537	871	1	871	0	98%	98%	238303249	Bacteria	n	n	uncultured bacterium	Uncultured bacterium clone nbw1021c12c1 16S ribosomal RNA gene, partial sequence
JQ999531	445	28	445	0	99%	99%	223675496	Bacteria	n	n	uncultured bacterium	Uncultured bacterium clone LL143-5K7 16S ribosomal RNA gene, partial sequence
JQ999561	246	1	239	1E-91	93%	93%	257143813	Bacteria	n	n	uncultured bacterium	Uncultured bacterium partial 16S rRNA gene, clone 12_E02
JQ999521	284	50	284	1E-106	97%	97%	220682601	Bacteria	n	n	uncultured bacterium	Uncultured bacterium clone ECN343f06 16S ribosomal RNA gene, partial sequence
JQ999515	243	1	243	7E-119	99%	99%	292596344	Bacteria	n	n	uncultured bacterium	Uncultured bacterium clone P2-DW11-16 16S ribosomal RNA gene, partial sequence
JQ999524	313	1	313	1E-151	98%	98%	71089597	Bacteria	n	n	uncultured bacterium	Uncultured bacterium clone AKAU4172 16S ribosomal RNA gene, partial sequence
JQ999518	251	60	248	2E-85	97%	97%	169286658	Bacteria	n	n	uncultured bacterium	Uncultured bacterium clone RP_3aaa02a05 16S ribosomal RNA gene, partial sequence
JQ999525	339	1	302	7E-155	100%	100%	169283493	Bacteria	n	n	uncultured bacterium	Uncultured bacterium clone CE2_c10_2 16S ribosomal RNA gene, partial sequence
JQ999530	430	29	430	1E-144	90%	90%	238409922	Bacteria	n	n	uncultured bacterium	Uncultured bacterium clone nbu95a04c1 16S ribosomal RNA gene, partial sequence
JQ999562	286	1	286	6E-125	95%	95%	76057884	Bacteria	n	n	uncultured bacterium	Uncultured bacterium partial 16S rRNA gene, clone E6
JQ999523	311	1	311	2E-159	100%	100%	291507684	Bacteria	n	n	uncultured bacterium	Uncultured bacterium clone 5EU8137F07 16S ribosomal RNA gene, partial sequence
JQ999563	398	1	363	1E-158	95%	95%	295810008	Bacteria	n	n	uncultured bacterium	Uncultured bacterium partial 16S rRNA gene, clone 25-0-H9
JQ999529	416	1	416	0	96%	96%	290616790	Bacteria	n	n	uncultured bacterium	Uncultured bacterium clone HF4686 16S ribosomal RNA gene, partial sequence
JQ999519	261	1	261	7E-129	99%	99%	238341248	Bacteria	n	n	uncultured bacterium	Uncultured bacterium clone nbw525g02c1 16S ribosomal RNA gene, partial sequence
JQ999533	544	1	529	0	93%	93%	192966167	Bacteria	n	n	uncultured bacterium	Uncultured bacterium clone A1_147 16S small subunit ribosomal RNA gene, partial sequence
JQ999520	270	80	270	4E-67	92%	92%	291506987	Bacteria	n	n	uncultured bacterium	Uncultured bacterium clone 4EU938C09 16S ribosomal RNA gene, partial sequence
JQ999526	349	1	349	3E-178	99%	99%	261262250	Bacteria	n	n	uncultured bacterium	Uncultured bacterium clone S1-8 16S ribosomal RNA gene, partial sequence
JQ999512	239	1	239	1E-110	97%	97%	284158279	Bacteria	n	n	uncultured bacterium	Uncultured bacterium clone BS13_119 16S ribosomal RNA gene, partial sequence
JQ999528	387	1	381	0	98%	98%	192988657	Bacteria	n	n	uncultured bacterium	Uncultured bacterium clone RP_3aaa01d02 16S ribosomal RNA gene, partial sequence
JQ999536	720	1	720	0	92%	92%	82393901	Bacteria	n	n	uncultured bacterium	Uncultured bacterium clone BANW440 16S ribosomal RNA gene, partial sequence
JQ999564	516	1	516	0	98%	98%	257144483	Bacteria	n	n	uncultured bacterium	Uncultured bacterium partial 16S rRNA gene, clone 5_E02
JQ999527	355	4	355	5E-142	93%	93%	151548166	Bacteria	n	n	uncultured bacterium	Uncultured bacterium clone FW1023-071 16S ribosomal RNA gene, partial sequence
JQ999532	482	1	482	0	92%	92%	110440003	Bacteria	n	n	uncultured bacterium	Uncultured bacterium clone RL305aal88b09 16S ribosomal RNA gene, partial sequence
JQ999517	249	1	249	7E-119	98%	98%	217417015	Bacteria	n	n	uncultured bacterium	Uncultured bacterium clone A_D_02_42 16S ribosomal RNA gene, partial sequence
JQ999511	219	1	219	4E-96	96%	96%	240000804	Bacteria	n	n	uncultured bacterium	Uncultured bacterium clone SHZB684 16S ribosomal RNA gene, partial sequence
JQ999513	240	2	240	2E-99	95%	95%	109676490	Bacteria	n	n	uncultured bacterium	Uncultured bacterium isolate DGGE gel band A3-5 16S ribosomal RNA gene, partial sequence
JQ999510	212	1	212	6E-99	98%	98%	158998775	Bacteria	n	n	lobster gut bacterium ABHa3	Lobster gut bacterium ABHa3 16S ribosomal RNA gene, partial sequence
JQ999514	242	1	218	4E-106	99%	99%	295814818	Bacteria	n	n	uncultured bacterium	Uncultured bacterium clone PE2 MKM24 16S ribosomal RNA gene, partial sequence
JQ999522	284	1	284	2E-120	95%	95%	192979871	Bacteria	n	n	uncultured bacterium	Uncultured bacterium clone CE2_d01_2 16S ribosomal RNA gene, partial sequence
JQ999565	401	1	319	1E-163	100%	100%	224027508	Bacteria	Proteobacteria	Alphaproteobacteria	Brevundimonas sp. AKB-2008-KU11	Brevundimonas sp. AKB-2008-KU11 partial 16S rRNA gene, strain AKB-2008-KU11
JQ999539	226	1	226	1E-105	98%	98%	189306205	Bacteria	Proteobacteria	Alphaproteobacteria	uncultured Mycoplana sp.	Uncultured Mycoplana sp. clone 3P-3-2-G09 16S ribosomal RNA gene, partial sequence
JQ999538	233	3	233	1E-90	93%	93%	148615349	Bacteria	Proteobacteria	Alphaproteobacteria	uncultured alpha proteobacterium	Uncultured alpha proteobacterium clone 2030 16S ribosomal RNA gene, partial sequence
JQ999566	270	1	245	2E-119	99%	99%	134084827	Bacteria	Proteobacteria	Alphaproteobacteria	Subaequorebacter tamlense	Subaequorebacter tamlense partial 16S rRNA gene, strain type strain: HST3-12
JQ999542	515	3	515	0	91%	91%	213536827	Bacteria	Proteobacteria	Betaproteobacteria	Delftia acidovorans	Delftia acidovorans ATCC:9355 16S ribosomal RNA gene, partial sequence; 16S-23S ribosomal intergenic spacer, complete sequence; and 23S ribosomal RNA gene, partial sequence
JQ999541	655	2	655	0	96%	96%	255348346	Bacteria	Proteobacteria	Betaproteobacteria	Comamonas sp. BF-3	Comamonas sp. BF-3 16S ribosomal RNA gene, partial sequence
JQ999540	373	1	373	5E-172	95%	95%	295322914	Bacteria	Proteobacteria	Betaproteobacteria	Burkholderia cepacia	Burkholderia cepacia strain TS20 16S ribosomal RNA gene, partial sequence
JQ999567	397	10	397	7E-146	91%	91%	291482199	Bacteria	Proteobacteria	Betaproteobacteria	uncultured beta proteobacterium	Uncultured beta proteobacterium partial 16S rRNA gene, clone P1B1G30
JQ999544	450	8	450	0	96%	96%	149900449	Bacteria	Proteobacteria	Betaproteobacteria	Uncultured betaproteobacterium	Uncultured beta proteobacterium clone 1233 16S ribosomal RNA gene, partial sequence
JQ999543	234	1	234	2E-113	99%	99%	285200309	Bacteria	Proteobacteria	Betaproteobacteria	Herbaspirillum sp. oral taxon A32	Herbaspirillum sp. oral taxon A32 clone XZ008 16S ribosomal RNA gene, partial sequence
JQ999548	959	20	959	0	100%	100%	295394130	Bacteria	Proteobacteria	Gammaproteobacteria	Escherichia sp. enrichment culture clone	Escherichia sp. enrichment culture clone NBAR006 16S ribosomal RNA gene, partial sequence
JQ999545	634	1	634	0	97%	97%	255763066	Bacteria	Proteobacteria	Gammaproteobacteria	Rheinheimera sp. HMD2012	Rheinheimera sp. HMD2012 16S ribosomal RNA gene, partial sequence
JQ999554	314	1	314	5E-161	100%	100%	295687302	Bacteria	Proteobacteria	Gammaproteobacteria	uncultured Pseudomonas sp.	Uncultured Pseudomonas sp. clone 5068 16S ribosomal RNA gene, partial sequence
JQ999552	275	1	275	8E-134	98%	98%	162951385	Bacteria	Proteobacteria	Gammaproteobacteria	uncultured gamma proteobacterium	Uncultured gamma proteobacterium clone TSN40 16S ribosomal RNA gene, partial sequence
JQ999506	346	73	346	5E-137	99%	99%	269931076	Bacteria	Proteobacteria	Gammaproteobacteria	Escherichia coli	Uncultured Escherichia sp. clone 2d2131 16S ribosomal RNA gene, partial sequence
JQ999549	309	1	309	5E-156	99%	99%	269911743	Bacteria	Proteobacteria	Gammaproteobacteria	uncultured Enterobacteriaceae bacterium	Uncultured Enterobacteriaceae bacterium clone Cat003E_B05 small subunit ribosomal RNA gene, partial sequence
JQ999556	416	32	416	7E-161	94%	94%	57918745	Bacteria	Proteobacteria	Gammaproteobacteria	Vibrio sp. U32	Vibrio sp. U32 16S ribosomal RNA gene, partial sequence
JQ999551	756	1	756	0	99%	99%	294799818	Bacteria	Proteobacteria	Gammaproteobacteria	Shigella sp. 29_2010_	Shigella sp. 29(2010) 16S ribosomal RNA gene, partial sequence
JQ999550	454	16	454	1E-174	93%	93%	39546462	Bacteria	Proteobacteria	Gammaproteobacteria	rainbow trout intestinal bacterium T1	Rainbow trout intestinal bacterium T1 16S ribosomal RNA gene, complete sequence
JQ999546	229	1	229	3E-112	99%	99%	257073647	Bacteria	Proteobacteria	Gammaproteobacteria	uncultured Citrobacter sp.	Uncultured Citrobacter sp. clone F2apr.30 16S ribosomal RNA gene, partial sequence
JQ999547	228	1	228	1E-111	99%	99%	257074351	Bacteria	Proteobacteria	Gammaproteobacteria	uncultured Enterobacter sp.	Uncultured Enterobacter sp. clone F4apr.29 16S ribosomal RNA gene, partial sequence
JQ999557	279	1	279	3E-123	96%	96%	154194068	Bacteria	Proteobacteria	n	uncultured proteobacterium	Uncultured proteobacterium clone MS032A1_C06 16S ribosomal RNA gene, partial sequence
JQ999558	297	49	297	5E-116	98%	98%	154190433	Bacteria	Proteobacteria	n	uncultured proteobacterium	Uncultured proteobacterium clone MS075A1_H11 16S ribosomal RNA gene, partial sequence
JQ999625	338	1	338	7E-170	99%	99%	291482367	Eukaryota	Ascomycota	Dothideomycetes	Cladosporium cladosporioides	Cladosporium cladosporioides gene for 18S ribosomal RNA, partial sequence
JQ999627	701	1	676	0	92%	92%	27447881	Eukaryota	Ascomycota	n	Medeolaria farlowii	Medeolaria farlowii 18S ribosomal RNA gene, complete sequence
JQ999626	368	1	368	6E-176	98%	98%	219563700	Eukaryota	Ascomycota	Leotiomycetes	Cyathicula microspora	Cyathicula microspora isolate M267 18S small subunit ribosomal RNA gene, partial sequence
JQ999629	264	1	264	2E-125	98%	98%	156637429	Eukaryota	Ascomycota	Saccharomycetes	Millerozyma farinosa	Pichia farinosa strain CO-2 18S ribosomal RNA gene, partial sequence
JQ999633	840	1	840	0	95%	95%	283131270	Eukaryota	n	n	uncultured fungus	Uncultured fungus gene for 18S rRNA, partial sequence, clone: DHUP10
JQ999630	226	1	226	1E-111	100%	100%	157925543	Eukaryota	n	n	uncultured fungus	Uncultured fungus clone G913P35FL10.T0 18S ribosomal RNA gene, partial sequence
JQ999632	273	1	254	1E-126	99%	99%	256006248	n	n	n	uncultured organism	Uncultured organism clone BFXI412-106 16S ribosomal RNA gene, partial sequence
JQ999631	303	1	303	2E-154	100%	100%	290782478	Eukaryota	Streptophyta	n	Melicope cf. crassiramis SW-2006	Melicope cf. crassiramis SW-2006 isolate EE4_03B_MELCRA 18S ribosomal RNA gene, partial sequence
