# Supplementary material for: Subglacial Lake Vostok (Antarctica) Accretion Ice Contains a Diverse Set of Sequences from Aquatic, Marine and Sediment-Inhabiting Bacteria and Eukarya
Source: PLoS One. 2013 Jul 3;8(7):e67221. doi: 10.1371/journal.pone.0067221 (PMC3700977; doi:10.1371/journal.pone.0067221)
Supplement: Table S8 — Large subunit rRNA gene sequences of Bacteria and Eukarya from V6. [“n” indicates information not specified in the NCBI GenBank database.]. (PDF) [file pone.0067221.s013.pdf]

Table S8. Large subunit rRNA gene sequences of Bacteria and Eukarya from V6. ["n" indicates information not specified in the NCBI GenBank database.]

Accession number	Q length	Q start	Q end	e-value	%-ident	%-sim	GI number	Domain	Phylum	Class	Genus / Species	Description
JQ999835	250	1	250	2E-105	95%	95%	296416	Bacteria	Firmicutes	Bacilli	Sporosarcina globispora	B.globisporus gene for 23S rRNA
JQ999833	308	68	308	7E-75	86%	86%	291259210	Bacteria	n	n	uncultured bacterium	Uncultured bacterium clone F5K2Q4C04IEQ8G 23S ribosomal RNA gene, partial sequence
JQ999831	226	1	205	5E-90	97%	97%	291260098	Bacteria	n	n	uncultured bacterium	Uncultured bacterium clone F5K2Q4C04I8I6J 23S ribosomal RNA gene, partial sequence
JQ999830	222	1	222	2E-108	99%	99%	291258986	Bacteria	n	n	uncultured bacterium	Uncultured bacterium clone F5K2Q4C04JAJZN 23S ribosomal RNA gene, partial sequence
JQ999907	588	178	588	0	97%	97%	159171560	Eukaryota	Ascomycota	Dothideomycetes	Phaeosphaeria avenaria	Phaeosphaeria avenaria f. sp. avenaria strain 1919WRS large subunit ribosomal RNA gene, partial sequence
JQ999906	500	1	500	0	99%	99%	284158823	Eukaryota	Ascomycota	Dothideomycetes	Davidiella tassiana	Davidiella tassiana strain CBS 723.79 28S large subunit ribosomal RNA gene, partial sequence
JQ999628	749	4	749	0	94%	94%	291170394	Eukaryota	Ascomycota	n	Coniosporium apollinis	Coniosporium apollinis strain CBS 100218 large subunit ribosomal RNA gene, partial sequence
JQ999908	231	1	231	1E-106	97%	97%	38154526	Eukaryota	n	n	uncultured fungus	Uncultured fungus 28S ribosomal RNA gene, partial sequence
