# Supplementary material for: Subglacial Lake Vostok (Antarctica) Accretion Ice Contains a Diverse Set of Sequences from Aquatic, Marine and Sediment-Inhabiting Bacteria and Eukarya
Source: PLoS One. 2013 Jul 3;8(7):e67221. doi: 10.1371/journal.pone.0067221 (PMC3700977; doi:10.1371/journal.pone.0067221)
Supplement: Table S9 — Ribosomal RNA gene sequences less than 200 nt in length (could not be submitted to NCBI) from V6. [“n” indicates information not specified in the NCBI GenBank database.]. (PDF) [file pone.0067221.s014.pdf]

**Table S9. Ribosomal RNA gene sequences less than 200 nt in length (could not be submitted to NCBI) from V6. ["n" indicates information not specified in the NCBI GenBank database.]**

| Contig ID | Q length | Q start | Q end | e-value | %-ident | %-sim | GI number | Domain | Phyum | Class | Genus / Species | Description |
|---|---|---|---|---|---|---|---|---|---|---|---|---|
| VostokV6_rep_c226 | 179 | 1 | 179 | 1E-85 | 99% | 99% | 262063836 | Bacteria | Actinobacteria | Actinobacteria | uncultured Frankineae bacterium | Uncultured Frankineae bacterium clone PE19.42 16S ribosomal RNA gene, partial sequence |
| VostokV6_s130 | 192 | 1 | 124 | 1E-55 | 99% | 99% | 268032028 | Bacteria | Actinobacteria | Actinobacteria | Micrococcus sp. M-B-1 | Micrococcus sp. M-B-1 16S ribosomal RNA gene, partial sequence |
| VostokV6_rep_c96 | 197 | 1 | 156 | 1E-60 | 94% | 94% | 95117795 | Bacteria | Actinobacteria | Actinobacteria | Clavibacter michiganensis | Clavibacter michiganensis subsp. tessellarius 23S ribosomal RNA gene, partial sequence |
| VostokV6_s134 | 134 | 1 | 134 | 1E-59 | 99% | 99% | 196174919 | Bacteria | Actinobacteria | Actinobacteria | Mycobacterium marinum | Mycobacterium marinum strain R106 23S ribosomal RNA gene, partial sequence |
| VostokV6_s244 | 108 | 1 | 108 | 5E-47 | 99% | 99% | 290794322 | Bacteria | Chloroflexi | n | uncultured Chloroflexi bacterium | Uncultured Chloroflexi bacterium clone 34Jc03 16S ribosomal RNA gene, partial sequence |
| VostokV6_rep_c194 | 175 | 1 | 175 | 1E-85 | 100% | 100% | 9187671 | Bacteria | n | n | uncultured rape rhizosphere | Uncultured rape rhizosphere bacterium wr0033 partial 16S rRNA gene |
| VostokV6_rep_c199 | 200 | 23 | 200 | 8E-68 | 94% | 94% | 119352337 | Bacteria | n | n | uncultured bacterium | Uncultured bacterium clone R383 16S ribosomal RNA gene, partial sequence |
| VostokV6_rep_c274 | 203 | 1 | 203 | 6E-59 | 85% | 85% | 28848718 | Bacteria | n | n | uncultured bacterium | Uncultured bacterium clone SB8 16S ribosomal RNA gene, partial sequence |
| VostokV6_s184 | 137 | 1 | 137 | 1E-64 | 100% | 100% | 126402247 | Bacteria | n | n | uncultured bacterium | Uncultured bacterium clone PLA_021 16S ribosomal RNA gene, partial sequence |
| VostokV6_s243 | 78 | 1 | 78 | 3E-32 | 100% | 100% | 295687301 | Bacteria | n | n | uncultured bacterium | Uncultured bacterium clone 3351 16S ribosomal RNA gene, partial sequence |
| VostokV6_s265 | 188 | 1 | 188 | 3E-86 | 98% | 98% | 217416971 | Bacteria | n | n | uncultured bacterium | Uncultured bacterium clone A_D_01_47 16S ribosomal RNA gene, partial sequence |
| VostokV6_rep_c157 | 210 | 1 | 210 | 1E-96 | 97% | 97% | 291260192 | Bacteria | n | n | uncultured bacterium | Uncultured bacterium clone F5K2Q4C04H4RWE 23S ribosomal RNA gene, partial sequence |
| VostokV6_rep_c293 | 195 | 1 | 195 | 9E-92 | 98% | 98% | 291261679 | Bacteria | n | n | uncultured bacterium | Uncultured bacterium clone F5K2Q4C04IX9DT 23S ribosomal RNA gene, partial sequence |
| VostokV6_s95 | 99 | 1 | 76 | 6E-31 | 100% | 100% | 291260719 | Bacteria | n | n | uncultured bacterium | Uncultured bacterium clone F5K2Q4C04IL5DQ 23S ribosomal RNA gene, partial sequence |
| VostokV6_c165 | 203 | 1 | 203 | 2E-84 | 95% | 95% | 285159491 | Bacteria | Proteobacteria | Betaproteobacteria | Lautropia mirabilis | Lautropia mirabilis clone AQ137 16S ribosomal RNA gene, partial sequence |
| VostokV6_s253 | 156 | 1 | 156 | 7E-72 | 99% | 99% | 284810302 | Bacteria | Proteobacteria | Betaproteobacteria | Burkholderia sp. CV4.4.3R1 | Burkholderia sp. CV4.4.3R1 16S ribosomal RNA gene, partial sequence |
| VostokV6_s62 | 105 | 43 | 105 | 6E-21 | 97% | 97% | 238057381 | Bacteria | Proteobacteria | Betaproteobacteria | Uncultured Thiobacillus sp. | Uncultured Thiobacillus sp. clone REG_R2P1_F10 16S ribosomal RNA gene, partial sequence |
| VostokV6_rep_c148 | 163 | 1 | 163 | 4E-65 | 95% | 95% | 98975330 | Bacteria | Proteobacteria | Gammaproteobacteria | Moraxella bovoculi | Moraxella bovoculi strain 2471-2 16S ribosomal RNA gene, partial sequence; 16S-23S ribosomal RNA intergenic spacer, complete sequence; and 23S ribosomal RNA gene, partial sequence |
| VostokV6_s113 | 198 | 1 | 162 | 8E-53 | 90% | 90% | 1240033 | Bacteria | Proteobacteria | Gammaproteobacteria | Escherichia | Escherichia sp. gene for 16S rRNA |
| VostokV6_c270 | 148 | 1 | 148 | 9E-71 | 100% | 100% | 284159233 | Eukaryota | Ascomycota | Saccharomycetes | Ogataea thermomethanolica | Ogataea thermomethanolica strain ATCC MYA-4465 18S ribosomal RNA gene, complete sequence |
| VostokV6_c110 | 94 | 25 | 94 | 7E-25 | 97% | 97% | 166947935 | Eukaryota | Ascomycota | Saccharomycetes | Candida tropicalis | Candida tropicalis isolate YZ1 26S ribosomal RNA gene, partial sequence |
| VostokV6_c187 | 111 | 1 | 111 | 5E-42 | 94% | 94% | 158819348 | Eukaryota | Ascomycota | Saccharomycetes | Babjeviella inositovora | Pichia inositovora strain NRRL Y-12698 26S ribosomal RNA gene, partial sequence |
| VostokV6_c100 | 177 | 4 | 177 | 4E-85 | 100% | 100% | 234195550 | Eukaryota | Basidiomycota | n | uncultured Agaricomycotina | Uncultured Agaricomycotina clone S-90 18S ribosomal RNA gene, partial sequence |
| VostokV6_c223 | 77 | 1 | 77 | 1E-31 | 100% | 100% | 295393258 | Eukaryota | Basidiomycota | Tremellomycetes | Bullera taiwanensis | Bullera taiwanensis strain CK14 18S ribosomal RNA gene, partial sequence |
| VostokV6_rep_c131 | 185 | 1 | 184 | 1E-90 | 100% | 100% | 292660457 | Eukaryota | Basidiomycota | n | uncultured Basidiomycota | Uncultured Basidiomycota clone CR73P4H12 18S ribosomal RNA gene, partial sequence |
| VostokV6_c273 | 47 | 3 | 42 | 7E-10 | 98% | 98% | 256859920 | Eukaryota | Basidiomycota | Agaricomycetes | Geastrum sessile | Geastrum sessile voucher MW 367 28S ribosomal RNA gene, partial sequence |
| VostokV6_c233 | 218 | 35 | 218 | 5E-85 | 98% | 98% | 50845139 | Eukaryota | n | n | uncultured fungus | Uncultured fungus clone SBS2w20f 18S ribosomal RNA gene, partial sequence |
| VostokV6_rep_c141 | 114 | 1 | 114 | 5E-52 | 100% | 100% | 256373711 | Eukaryota | Streptophyta | n | Thalictrum simplex | Thalictrum simplex 18S ribosomal RNA gene, partial sequence |
