# Supplementary material for: Subglacial Lake Vostok (Antarctica) Accretion Ice Contains a Diverse Set of Sequences from Aquatic, Marine and Sediment-Inhabiting Bacteria and Eukarya
Source: PLoS One. 2013 Jul 3;8(7):e67221. doi: 10.1371/journal.pone.0067221 (PMC3700977; doi:10.1371/journal.pone.0067221)
Supplement: Table S10 — Bacteria and Eukarya mRNA (and other non-rRNA) gene sequences from V6. [“n” indicates information not specified in the NCBI GenBank database.]. (PDF) [file pone.0067221.s015.pdf]

Table S10. Bacteria and Eukarya mRNA (and other non-rRNA) gene sequences from V6. ["n" indicates information not specified in the NCBI GenBank database.]

Contig ID	Q length	Q start	Q end	e-value	%-ident	%-sim	GI number	Domain	Phyla	Class	Genus	Description
VostokV6_rep_c158	182	1	182	3.00E-61	91%	91%	237757549	Bacteria	Actinobacteria	Actinobacteria	Corynebacterium kroppenstedtii	Corynebacterium kroppenstedtii DSM 44385, complete genome
VostokV6_rep_c202	205	1	205	3.00E-92	97%	97%	119947346	Bacteria	Actinobacteria	Actinobacteria	Arthrobacter aurescens	Arthrobacter aurescens TC1, complete genome
VostokV6_rep_c217	321	1	321	9.00E-124	92%	92%	38200856	Bacteria	Actinobacteria	Actinobacteria	Corynebacterium diphtheriae	Corynebacterium diphtheriae gravis NCTC13129, complete genome; segment 7/8
VostokV6_rep_c246	175	4	175	2.00E-52	89%	89%	140843962	Bacteria	Actinobacteria	Actinobacteria	Corynebacterium glutamicum	Corynebacterium glutamicum R DNA, complete genome
VostokV6_s74	113	1	113	1.00E-47	98%	98%	162952245	Bacteria	Actinobacteria	Actinobacteria	Renibacterium salmoninarum	Renibacterium salmoninarum ATCC 33209, complete genome
VostokV6_c235	95	1	95	1.00E-41	100%	100%	254946573	Bacteria	Bacteroidetes	Cytophagia	Dyadobacter fermentans	Dyadobacter fermentans DSM 18053, complete genome
VostokV6_c112	258	5	258	6.00E-35	79%	79%	218766851	Bacteria	Deinococcus-Thermus	Deinococci	Thermus thermophilus	Chain A, Crystal Structure Of A Translation Termination Complex Formed With Release F
VostokV6_c161	147	1	142	2.00E-53	94%	94%	289178903	Bacteria	Firmicutes	Bacilli	Staphylococcus lugdunensis	Staphylococcus lugdunensis HKU09-01, complete genome
VostokV6_c212	189	1	189	3.00E-72	94%	94%	256797400	Bacteria	Firmicutes	Clostridia	Anaerococcus prevotii	Anaerococcus prevotii DSM 20548, complete genome
VostokV6_c256	587	31	587	0	95%	95%	295107714	Bacteria	Firmicutes	Clostridia	Ruminococcus obeum	Ruminococcus obeum A2-162 draft genome
VostokV6_c97	217	1	217	1.00E-81	93%	93%	262396937	Bacteria	Firmicutes	Bacilli	Lactobacillus johnsonii	Lactobacillus johnsonii FI9785, complete genome
VostokV6_rep_c149	186	1	186	9.00E-87	98%	98%	225726369	Bacteria	Firmicutes	Bacilli	Streptococcus pneumoniae	Streptococcus pneumoniae Taiwan19F-14, complete genome
VostokV6_rep_c204	316	53	316	7.00E-130	99%	99%	68445725	Bacteria	Firmicutes	Bacilli	Staphylococcus haemolyticus	Staphylococcus haemolyticus JCSC1435 DNA, complete genome
VostokV6_rep_c139	317	1	317	3.00E-119	92%	92%	257048753	Bacteria	Fusobacteria	Fusobacteria	Leptotrichia buccalis	Leptotrichia buccalis DSM 1135, complete genome
VostokV6_c185	164	1	164	1.00E-79	100%	100%	193084619	Bacteria	n	n	uncultured bacterium HF0500_12O04	Uncultured bacterium HF0500_12O04 genomic sequence
VostokV6_c248	245	3	209	1.00E-22	77%	77%	193084063	Bacteria	n	n	uncultured bacterium KM3-23-D4	Uncultured bacterium KM3-23-D4 genomic sequence
VostokV6_s291	200	1	200	2.00E-68	92%	92%	62860402	Bacteria	n	n	uncultured bacterium zdt-44a23	Uncultured bacterium zdt-44a23 clone zdt-44a23, complete sequence
VostokV6_rep_c123	498	48	498	0	94%	94%	193084637	Bacteria	n	n	uncultured bacterium HF4000_16C08	Uncultured bacterium HF4000_16C08 genomic sequence
VostokV6_c15	584	1	584	0	100%	100%	146403799	Bacteria	Proteobacteria	Alphaproteobacteria	Bradyrhizobium sp. BTAi1	Bradyrhizobium sp. BTAi1, complete genome
VostokV6_rep_c230	265	2	265	2.00E-114	96%	96%	188532098	Bacteria	Proteobacteria	Alphaproteobacteria	Sphingobium chungbukense	Sphingobium chungbukense rrnC operon, complete sequence
VostokV6_s272	222	2	222	3.00E-97	96%	96%	288914861	Bacteria	Proteobacteria	Alphaproteobacteria	Azospirillum sp. B510	Azospirillum sp. B510 plasmid pAB510e DNA, complete genome
VostokV6_c16	689	25	689	0	98%	98%	120591888	Bacteria	Proteobacteria	Betaproteobacteria	Polaromonas naphthalenivorans	Polaromonas naphthalenivorans CJ2, complete genome
VostokV6_c245	332	1	332	8.00E-90	86%	86%	160361034	Bacteria	Proteobacteria	Betaproteobacteria	Delftia acidovorans	Delftia acidovorans SPH-1, complete genome
VostokV6_c268	191	71	191	6.00E-14	80%	80%	294338440	Bacteria	Proteobacteria	Betaproteobacteria	Thiomonas sp. 3As	Thiomonas sp. str. 3As, chromosome, complete genome
VostokV6_c72	111	1	96	2.00E-25	90%	90%	121551644	Bacteria	Proteobacteria	Betaproteobacteria	Verminephrobacter eiseniae	Verminephrobacter eiseniae EF01-2, complete genome
VostokV6_rep_c124	358	1	358	2.00E-155	95%	95%	221728669	Bacteria	Proteobacteria	Betaproteobacteria	Acidovorax ebreus	Acidovorax ebreus TPSY, complete genome
VostokV6_rep_c77	251	1	251	2.00E-95	92%	92%	237502667	Bacteria	Proteobacteria	Betaproteobacteria	Burkholderia pseudomallei	Burkholderia pseudomallei MSHR346 chromosome I, complete sequence
VostokV6_c247	203	1	203	6.00E-94	98%	98%	145692985	Bacteria	Proteobacteria	Gammaproteobacteria	Pseudomonas aeruginosa	Pseudomonas aeruginosa strain NIH-1 tail fiber (prf15) and tail fiber assembly (prf16) ge
VostokV6_rep_c104	605	1	563	0	99%	99%	281599365	Bacteria	Proteobacteria	Gammaproteobacteria	Shigella flexneri	Shigella flexneri 2002017, complete genome
VostokV6_rep_c117	194	1	194	2.00E-73	93%	93%	161361677	Bacteria	Proteobacteria	Gammaproteobacteria	Salmonella enterica	Salmonella enterica subsp. enterica serovar Paratyphi B str. SPB7, complete genome
VostokV6_rep_c155	257	1	257	2.00E-125	99%	99%	291150583	Bacteria	Proteobacteria	Gammaproteobacteria	Pantoea ananatis	Pantoea ananatis LMG 20103, complete genome
VostokV6_rep_c200	325	1	325	9.00E-139	94%	94%	206564770	Bacteria	Proteobacteria	Gammaproteobacteria	Klebsiella pneumoniae	Klebsiella pneumoniae 342, complete genome
VostokV6_rep_c218	285	62	285	2.00E-85	93%	93%	291551905	Bacteria	Proteobacteria	Gammaproteobacteria	Erwinia amylovora	Erwinia amylovora CFBP1430 complete genome
VostokV6_s87	79	1	79	5.00E-30	98%	98%	253778933	Bacteria	Proteobacteria	Gammaproteobacteria	Photorhabdus asymbiotica	Photorhabdus asymbiotica ATCC43949 complete genome
VostokV6_rep_c171	564	28	536	0	99%	99%	294910885	Eukaryota	Arthropoda	Arachnida	Dermacentor variabilis	TSA: Dermacentor variabilis contig00102.Dvb, mRNA sequence
VostokV6_rep_c192	247	1	247	4.00E-121	98%	98%	294661761	Eukaryota	Arthropoda	Branchiopoda	Daphnia pulex	Daphnia pulex ncRNA, kairomone-inducible transcript
VostokV6_s122	220	1	220	5.00E-105	98%	98%	294922086	Eukaryota	Arthropoda	Arachnida	Dermacentor variabilis	TSA: Dermacentor variabilis contig01324.Dvc, mRNA sequence
VostokV6_s69	206	1	206	1.00E-91	96%	96%	294922085	Eukaryota	Arthropoda	Arachnida	Dermacentor variabilis	TSA: Dermacentor variabilis contig01323.Dvc, mRNA sequence
VostokV6_s73	107	1	65	5.00E-22	97%	97%	294910977	Eukaryota	Arthropoda	Arachnida	Dermacentor variabilis	TSA: Dermacentor variabilis contig00178.Dvb, mRNA sequence
