# Supplementary material for: Subglacial Lake Vostok (Antarctica) Accretion Ice Contains a Diverse Set of Sequences from Aquatic, Marine and Sediment-Inhabiting Bacteria and Eukarya
Source: PLoS One. 2013 Jul 3;8(7):e67221. doi: 10.1371/journal.pone.0067221 (PMC3700977; doi:10.1371/journal.pone.0067221)
Supplement: Table S11 — Blastn and Blastx results from analysis of V5 sequences on the KAAS KEGG site [25] . The searches were for highly similar sequences (megablast); Max. target sequences = 100; Expected threshold = 1e-10 (unless no results were found, then 0); filter low complexity regions and translated nucleotide search over Reference sequence protein database; Matrix - BLOSUM62; Scoring parameters (existence 11; extension 1); filter low complexity regions. [“n” indicates information not specified in the NCBI GenBank database.]. (PDF) [file pone.0067221.s016.pdf]

Table S11. Blastn and Blastx results from analysis of V5 sequences on the KAAS KEGG site [25]. The searches were for highly similar sequences (megablast); Max. target sequences = 100; Expected threshold = 1e-10 (unless no results were found, then 0); filter low complexity regions and translated nucleotide search over Reference sequence protein database; Matrix - BLOSUM62; Scoring parameters (existence 11; extension 1); filter low complexity regions. ["n" indicates information not specified in the NCBI GenBank database.]

454 Sequence ID	Orthology #	KAAS KEGG Enzyme name	Pathways Names	Q length	Q start	Q end	e-value	%-ident	GI number	Domain	Phyla	Class / Order	Description
GKJWQY101AMLFW	K09687	antibiotic transport system ATP-binding protein	ABC transporters	308	1	308	2.0E-62	81%	451902131	Bacteria	Actinobacteria	Actinobacteria	Corynebacterium halotolerans YIM 70093 = DSM 44683, complete genome, product = BC-type multidrug transporter ATPase
GKJWQY101AYS8L	K02006	cobalt/nickel transport system ATP-binding protein	ABC transporters	288	1	288	1.0E-129	96%	428238862	Bacteria	Cyanobacteria	n	Oscillatoria nigro-viridis PCC 7112, complete genome, product = cobalt ABC transporter, ATPase subunit
GKJWQY101BNHJH	K02010	iron(III) transport system ATP-binding protein [EC:3.6.3.30]	ABC transporters	513	1	513	0.0E+00	95%	428238862	Bacteria	Cyanobacteria	n	Oscillatoria nigro-viridis PCC 7112, complete genome, product = Fe(3+)-transporting ATPase
GKJWQY101BAA0P	K02045	sulfate transport system ATP-binding protein [EC:3.6.3.25]	ABC transporters	468	1	468	0.0E+00	96%	428238862	Bacteria	Cyanobacteria	n	Oscillatoria nigro-viridis PCC 7112, complete genome, product = sulfate ABC transporter, ATPase subunit
GKJWQY101AGPVU	K09690	lipopolysaccharide transport system permease protein	ABC transporters	463	1	463	0.0E+00	94%	428238862	Bacteria	Cyanobacteria	n	Oscillatoria nigro-viridis PCC 7112, complete genome, product = ABC-2 type transporter
GKJWQY101AD59C	K11070	spermidine/putrescine transport system permease protein	ABC transporters	414	1	414	0.0E+00	95%	428238862	Bacteria	Cyanobacteria	n	Oscillatoria nigro-viridis PCC 7112, complete genome, product = binding-protein-dependent transport systems inner membrane component
GKJWQY101AYDKO	K11085	ATP-binding cassette, subfamily B, bacterial MsbA [EC:3.6.3.-]	ABC transporters	290	1	290	2.0E-123	97%	428238862	Bacteria	Cyanobacteria	n	Oscillatoria nigro-viridis PCC 7112, complete genome, product = xenobiotic-transporting ATPase
GKJWQY101AIM5Y	K01995	branched-chain amino acid transport system ATP-binding protein	ABC transporters	584	1	584	0.0E+00	99%	296848933	Bacteria	Deinococcus-Thermus	Deinococci	Meiothermus silvanus DSM 9946, complete genome, product = ABC transporter related protein
GKJWQY101A0EHG	K02035	peptide/nickel transport system substrate-binding protein	ABC transporters	495	1	495	0.0E+00	99%	296848933	Bacteria	Deinococcus-Thermus	Deinococci	Meiothermus silvanus DSM 9946, complete genome, product = ABC-type dipeptide/oligopeptide/nickel transport system, permease component
GKJWQY101ASUCA	K10036	glutamine transport system substrate-binding protein	ABC transporters	311	1	311	8.0E-161	100%	355393429	Bacteria	Firmicutes	Bacilli	Lactobacillus rhamnosus ATCC 8530, complete genome, product = amino ABC transporter, permease, 3-TM region, His/Glu/Gln/Arg/opine family domain protein
GKJWQY101AMX6D	K10118	multiple sugar transport system permease protein	ABC transporters	457	1	457	0.0E+00	98%	355393429	Bacteria	Firmicutes	Bacilli	Lactobacillus rhamnosus ATCC 8530, complete genome, product = binding--dependent transport system inner membrane component family protein (<1..51) (48..>456)
GKJWQY101AP0B9	K10440	ribose transport system permease protein	ABC transporters	239	1	239	1.0E-70	87%	433663430	Bacteria	Proteobacteria	Alphaproteobacteria	Mesorhizobium australicum WSM2073, complete genome, product = permease component of ribose/xylose/arabinose/galactoside ABC-type transporters
GKJWQY101BDGCG	K10441	ribose transport system ATP-binding protein [EC:3.6.3.17]	ABC transporters	450	1	450	2.0E-118	84%	433663430	Bacteria	Proteobacteria	Alphaproteobacteria	Mesorhizobium australicum WSM2073, complete genome, product = ABC-type sugar transport system, ATPase component <1..252); ABC-type xylose transport system, permease component (249..>446)
GKJWQY101BVWN6	K11073	putrescine transport system substrate-binding protein	ABC transporters	489	1	489	0.0E+00	99%	387575654	Bacteria	Proteobacteria	Betaproteobacteria	Burkholderia sp. KJ006 chromosome 1, complete sequence, product = putrescine ABC transporter putrescine-binding protein PotF
GKJWQY101B17TY	K10228	sorbitol/mannitol transport system permease protein	ABC transporters	315	1	315	2.0E-146	97%	77965403	Bacteria	Proteobacteria	Betaproteobacteria	Burkholderia sp. 383 chromosome 1, complete sequence, product = mannitol ABC transporter membrane protein/sorbitol ABC transporter membrane protein
GKJWQY101B17TY_2	K10228	sorbitol/mannitol transport system permease protein	ABC transporters	161	1	161	7.0E-64	95%	77965403	Bacteria	Proteobacteria	Betaproteobacteria	Burkholderia sp. 383 chromosome 1, complete sequence, product = sorbitol ABC transporter membrane protein/mannitol ABC transporter membrane protein
GKJWQY101BU6BN	K01999	branched-chain amino acid transport system substrate-binding protein	ABC transporters	376	1	376	8.0E-112	87%	86284380	Bacteria	Proteobacteria	Alphaproteobacteria	Rhizobium etli CFN 42 plasmid p42e, complete sequence, product = probable branched-chain amino acid ABC transporter, substrate-binding protein
GKJWQY101BLVUA	K11710	manganese/zinc/iron transport system ATP-binding protein	ABC transporters	521	4	521	0.0E+00	94%	428238862	Bacteria	Cyanobacteria	n	Oscillatoria nigro-viridis PCC 7112, complete genome, product = phosphonate-transporting ATPase
GKJWQY101AZ1BP	K11952	bicarbonate transport system ATP-binding protein [EC:3.6.3.-]	ABC transporters	401	1	401	0.0E+00	97%	428238862	Bacteria	Cyanobacteria	n	Oscillatoria nigro-viridis PCC 7112, complete genome, product = nitrate ABC transporter, ATPase subunits C and D
GKJWQY101BA0N7	K11959	urea transport system substrate-binding protein	ABC transporters	457	1	457	0.0E+00	92%	428238862	Bacteria	Cyanobacteria	n	Oscillatoria nigro-viridis PCC 7112, complete genome, product = integral membrane sensor hybrid histidine kinase
GKJWQY101BB18D	K12368	dipeptide transport system substrate-binding protein	ABC transporters	471	1	471	0.0E+00	99%	387575654	Bacteria	Proteobacteria	Betaproteobacteria	Burkholderia sp. KJ006 chromosome 1, complete sequence, product = dipeptide-binding ABC transporter, periplasmic substrate-binding component
GKJWQY101ACKKD	K12369	dipeptide transport system permease protein	ABC transporters	474	1	474	0.0E+00	99%	387575654	Bacteria	Proteobacteria	Betaproteobacteria	Burkholderia sp. KJ006 chromosome 1, complete sequence, product = dipeptide transport system permease protein DppB (<1..211); dipeptide-binding ABC transporter, periplasmic substrate-binding component (331..>474)
GKJWQY101BC35N	K01533	Cu2+-exporting ATPase [EC:3.6.3.4]	Acting on acid anhydrides to catalyse transmembrane movement of substances	331	1	331	4.0E-164	99%	355393429	Bacteria	Firmicutes	Bacilli	Lactobacillus rhamnosus ATCC 8530, complete genome, product = copper-translocating P-type ATPase
GKJWQY101AY87X	K01265	methionyl aminopeptidase [EC:3.4.11.18]	Acting on peptide bonds (peptidases)	359	1	359	2.0E-147	93%	428238862	Bacteria	Cyanobacteria	n	Oscillatoria nigro-viridis PCC 7112, complete genome, product = methionine aminopeptidase, type I
GKJWQY101AVMJN	K01258	tripeptide aminopeptidase [EC:3.4.11.4]	Acting on peptide bonds (peptidases)	498	1	498	0.0E+00	99%	355393429	Bacteria	Firmicutes	Bacilli	Lactobacillus rhamnosus ATCC 8530, complete genome, product = peptidase T
GKJWQY101B0Z6C	K00676	ribosomal-protein-alanine N-acetyltransferase [EC:2.3.1.128]	Acyltransferases [Transfer groups other than aminoacyl groups]	340	1	340	3.0E-135	93%	428238862	Bacteria	Cyanobacteria	n	Oscillatoria nigro-viridis PCC 7112, complete genome, product = GCN5-related N-acetyltransferase
GKJWQY101A7UDN	K00684	leucyl/phenylalanyl-tRNA--protein transferase [EC:2.3.2.6]	Acyltransferases [tRNA modification factors]	423	1	423	0.0E+00	95%	428238862	Bacteria	Cyanobacteria	n	Oscillatoria nigro-viridis PCC 7112, complete genome, product = leucyl/phenylalanyl-tRNA--protein transferase (<1..283); hypothetical protein (330..>422)
GKJWQY101B20C7	K13821	proline dehydrogenase / delta 1-pyrroline-5-carboxylate dehydrogenase [EC:1.5.1.12 1.5.99.8]	Alanine, aspartate and glutamate metabolism	480	2	478	2.0E-93	95%	400288736	Bacteria	Proteobacteria	Gammaproteobacteria	bifunctional proline dehydrogenase/pyrroline-5-carboxylate dehydrogenase [Psychrobacter sp. PAMC 21119]
GKJWQY101AKE4Q	K01425	glutaminase [EC:3.5.1.2]	Alanine, aspartate and glutamate metabolism/Arginine and proline metabolism/D-Glutamine and D-glutamate metabolism/Nitrogen metabolism	407	1	407	1.0E-170	94%	428238862	Bacteria	Cyanobacteria	n	Oscillatoria nigro-viridis PCC 7112, complete genome, product = L-glutaminase

GKJWQY101AKLKY	K00812	aspartate aminotransferase [EC:2.6.1.1]	Alanine, aspartate and glutamate metabolism/Cysteine and methionine metabolism/Arginine and proline metabolism/Tyrosine metabolism/Phenylalanine metabolism/Phenylalanine, tyrosine and tryptophan biosynthesis/Novobiocin biosynthesis/Isoquinoline alkaloid biosynthesis/Tropane, piperidine and pyridine alkaloid biosynthesis	473	1	473	0.0E+00	98%	325124855	Bacteria	Firmicutes	Bacilli	Lactobacillus delbrueckii subsp. bulgaricus 2038, complete genome, product = aspartate aminotransferase
GKJWQY101B0F5K	K00278	L-aspartate oxidase [EC:1.4.3.16]	Alanine, aspartate and glutamate metabolism/Nicotinate and nicotinamide metabolism	481	1	481	0.0E+00	97%	428238862	Bacteria	Cyanobacteria	n	Oscillatoria nigro-viridis PCC 7112, complete genome, product = L-aspartate oxidase
GKJWQY101BLU93	K00259	alanine dehydrogenase [EC:1.4.1.1]	Alanine, aspartate and glutamate metabolism/Taurine and hypotaurine metabolism	465	1	465	0.0E+00	93%	428238862	Bacteria	Cyanobacteria	n	Oscillatoria nigro-viridis PCC 7112, complete genome, product = alanine dehydrogenase/pyridine nucleotide transhydrogenase
GKJWQY101BPBGL	K04042	bifunctional UDP-N-acetylglucosamine pyrophosphorylase / Glucosamine-1-phosphate N-acetyltransferase [EC:2.3.1.157 2.7.7.23]	Amino sugar and nucleotide sugar metabolism	407	1	407	0.0E+00	99%	110673209	Bacteria	Firmicutes	Clostridia	Clostridium perfringens ATCC 13124, complete genome, product = UDP-N-acetylglucosamine diphosphorylase/glucosamine-1-phosphate N-acetyltransferase
GKJWQY101AGM2N	K01443	N-acetylglucosamine-6-phosphate deacetylase [EC:3.5.1.25]	Amino sugar and nucleotide sugar metabolism	458	1	458	0.0E+00	99%	355393429	Bacteria	Firmicutes	Bacilli	Lactobacillus rhamnosus ATCC 8530, complete genome, product = N-acetylglucosamine-6-phosphate deacetylase
GKJWQY101AM94T	K01791	UDP-N-acetylglucosamine 2-epimerase [EC:5.1.3.14]	Amino sugar and nucleotide sugar metabolism	449	1	449	0.0E+00	96%	387578572	Bacteria	Proteobacteria	Betaproteobacteria	Burkholderia sp. KJ006 chromosome 2, complete sequence, product = UDP-N-acetylglucosamine 2-epimerase
GKJWQY101ARLO8	K01876	aspartyl-tRNA synthetase [EC:6.1.1.12]	Aminoacyl-tRNA biosynthesis	357	1	357	3.0E-161	96%	428238862	Bacteria	Cyanobacteria	n	Oscillatoria nigro-viridis PCC 7112, complete genome, product = aspartyl-tRNA synthetase
GKJWQY101BS0OD	K01887	arginyl-tRNA synthetase [EC:6.1.1.19]	Aminoacyl-tRNA biosynthesis	233	1	233	7.0E-76	89%	428238862	Bacteria	Cyanobacteria	n	Oscillatoria nigro-viridis PCC 7112, complete genome, product = arginyl-tRNA synthetase
GKJWQY101AXKT6	K01890	phenylalanyl-tRNA synthetase beta chain [EC:6.1.1.20]	Aminoacyl-tRNA biosynthesis	352	1	352	2.0E-148	94%	428238862	Bacteria	Cyanobacteria	n	Oscillatoria nigro-viridis PCC 7112, complete genome, product = phenylalanyl-tRNA synthetase beta subunit
GKJWQY101BY4M2	K01890	phenylalanyl-tRNA synthetase beta chain [EC:6.1.1.20]	Aminoacyl-tRNA biosynthesis	508	1	508	0.0E+00	95%	428238862	Bacteria	Cyanobacteria	n	Oscillatoria nigro-viridis PCC 7112, complete genome, product = phenylalanyl-tRNA synthetase beta subunit
GKJWQY101BC61Q	K01873	valyl-tRNA synthetase [EC:6.1.1.9]	Aminoacyl-tRNA biosynthesis	267	1	267	9.0E-135	99%	355393429	Bacteria	Firmicutes	Bacilli	Lactobacillus rhamnosus ATCC 8530, complete genome, product = valine--tRNA ligase
GKJWQY101B0MN7	K01879	glycyl-tRNA synthetase beta chain [EC:6.1.1.14]	Aminoacyl-tRNA biosynthesis	488	1	488	0.0E+00	99%	355393429	Bacteria	Firmicutes	Bacilli	Lactobacillus rhamnosus ATCC 8530, complete genome, product = glycine--tRNA ligase, beta subunit (<1..286); glycine--tRNA ligase, alpha subunit (288..>484)
GKJWQY101ABVRO	K01883	cysteinyl-tRNA synthetase [EC:6.1.1.16]	Aminoacyl-tRNA biosynthesis	442	1	442	0.0E+00	99%	355393429	Bacteria	Firmicutes	Bacilli	Lactobacillus rhamnosus ATCC 8530, complete genome, product = cysteine--tRNA ligase
GKJWQY101BCECM	K01866	tyrosyl-tRNA synthetase [EC:6.1.1.1]	Aminoacyl-tRNA biosynthesis	311	1	311	7.0E-37	76%	156768434	Bacteria	n	n	Uncultured bacterium clone LM0ABA39ZD07FM1 genomic sequence
GKJWQY101AATC2	K01874	methionyl-tRNA synthetase [EC:6.1.1.10]	Aminoacyl-tRNA biosynthesis	498	1	498	2.0E-139	85%	433663430	Bacteria	Proteobacteria	Alphaproteobacteria	Mesorhizobium australicum WSM2073, complete genome, product = methionyl-tRNA synthetase
GKJWQY101A4YI5	K04566	lysyl-tRNA synthetase, class I [EC:6.1.1.6]	Aminoacyl-tRNA biosynthesis	466	1	466	5.0E-115	83%	433663430	Bacteria	Proteobacteria	Alphaproteobacteria	Mesorhizobium australicum WSM2073, complete genome, product = lysyl-tRNA synthetase class I (K), archaeal and spirochete
GKJWQY101A5G4B	K04566	lysyl-tRNA synthetase, class I [EC:6.1.1.6]	Aminoacyl-tRNA biosynthesis	478	1	478	5.0E-120	83%	433663430	Bacteria	Proteobacteria	Alphaproteobacteria	Mesorhizobium australicum WSM2073, complete genome, product = lysyl-tRNA synthetase class I (K), archaeal and spirochete
GKJWQY101AVMQG	K04567	lysyl-tRNA synthetase, class II [EC:6.1.1.6]	Aminoacyl-tRNA biosynthesis	411	1	411	0.0E+00	99%	387575654	Bacteria	Proteobacteria	Betaproteobacteria	Burkholderia sp. KJ006 chromosome 1, complete sequence, product = lysyl-tRNA synthetase (class II)
GKJWQY101A1JG9	K00461	arachidonate 5-lipoxygenase [EC:1.13.11.34]	Arachidonic acid metabolism	485	1	485	3.0E-172	89%	428238862	Bacteria	Cyanobacteria	n	Oscillatoria nigro-viridis PCC 7112, complete genome, product = arachidonate 15-lipoxygenase
GKJWQY101BMOU7	K01259	proline iminopeptidase [EC:3.4.11.5]	Arginine and proline metabolism	367	1	367	0.0E+00	99%	355393429	Bacteria	Firmicutes	Bacilli	Lactobacillus rhamnosus ATCC 8530, complete genome, product = proline-specific peptidases family protein
GKJWQY101ALQO4	K01755	argininosuccinate lyase [EC:4.3.2.1]	Arginine and proline metabolism	441	1	441	0.0E+00	99%	387578572	Bacteria	Proteobacteria	Betaproteobacteria	Burkholderia sp. KJ006 chromosome 2, complete sequence, product = argininosuccinate lyase
GKJWQY101BHQ01	K01572	oxaloacetate decarboxylase, beta subunit [EC:4.1.1.3]	Arginine and proline metabolism/Pyruvate metabolism	242	1	242	5.0E-52	83%	149931032	Bacteria	Bacteroidetes	Bacteroidia	Bacteroides vulgatus ATCC 8482, complete genome, product = oxaloacetate decarboxylase beta chain
GKJWQY101A2NZC	K03476	L-ascorbate 6-phosphate lactonase [EC:3.1.1.-]	Ascorbate and aldarate metabolism	315	1	315	4.0E-159	99%	257149867	Bacteria	Firmicutes	Bacilli	Lactobacillus rhamnosus Lc 705 whole genome sequence, strain Lc705, product = L-ascorbate-6-phosphate lactonase
GKJWQY101BTC2H	K03204	type IV secretion system protein VirB9	Bacterial secretion system	483	1	483	2.0E-109	82%	474421396	Bacteria	Proteobacteria	Alphaproteobacteria	Mesorhizobium loti DNA, symbiosis island, strain: NZP2037, product = conjugal transfer protein, TrbG
GKJWQY101AFHLP	K03199	type IV secretion system protein VirB4	Bacterial secretion system	466	1	466	0.0E+00	93%	27817680	Bacteria	Proteobacteria	Betaproteobacteria	Ralstonia oxalatica transposon Tn4371, product = putative mating pair formation protein
GKJWQY101ASAUQ	K03076	preprotein translocase subunit SecY	Bacterial secretion system/Protein export	239	1	239	7.0E-56	84%	149770655	Bacteria	Bacteroidetes	Flavobacteriia	Flavobacterium psychrophilum JIP02/86 complete genome, product = preprotein translocase SecY subunit
GKJWQY101BIP1B	K03073	preprotein translocase subunit SecE	Bacterial secretion system/Protein export	350	1	350	3.0E-161	97%	428238862	Bacteria	Cyanobacteria	n	Oscillatoria nigro-viridis PCC 7112, complete genome, product = protein translocase subunit secE/sec61 gamma (<1..175); transcription antitermination protein nusG (172..>347)
GKJWQY101BCK7L	K01142	exodeoxyribonuclease III [EC:3.1.11.2]	Base excision repair	472	1	472	0.0E+00	98%	387575654	Bacteria	Proteobacteria	Betaproteobacteria	Burkholderia sp. KJ006 chromosome 1, complete sequence, product = exodeoxyribonuclease III
GKJWQY101BJH43	K10255	omega-6 fatty acid desaturase (delta-12 desaturase) [EC:1.14.19.-]	Biosynthesis of unsaturated fatty acids	486	1	486	0.0E+00	99%	387578572	Bacteria	Proteobacteria	Betaproteobacteria	Burkholderia sp. KJ006 chromosome 2, complete sequence, product = fatty acid desaturase
GKJWQY101BGYFQ	K00656	formate C-acetyltransferase [EC:2.3.1.54]	Butanoate metabolism	292	1	292	1.0E-133	97%	428238862	Bacteria	Cyanobacteria	n	Oscillatoria nigro-viridis PCC 7112, complete genome, product = formate acetyltransferase
GKJWQY101A3577	K01907	acetoacetyl-CoA synthetase [EC:6.2.1.16]	Butanoate metabolism	470	1	470	1.0E-150	87%	433663430	Bacteria	Proteobacteria	Alphaproteobacteria	Mesorhizobium australicum WSM2073, complete genome, product = acetoacetyl-CoA synthase
GKJWQY101AI2W0	K01907	acetoacetyl-CoA synthetase [EC:6.2.1.16]	Butanoate metabolism	479	1	479	3.0E-157	88%	433663430	Bacteria	Proteobacteria	Alphaproteobacteria	Mesorhizobium australicum WSM2073, complete genome, product = acetoacetyl-CoA synthase
GKJWQY101BJZAX	K07190	phosphorylase kinase alpha/beta subunit	Calcium signaling pathway	258	1	258	2.0E-126	99%	428238862	Bacteria	Cyanobacteria	n	Oscillatoria nigro-viridis PCC 7112, complete genome, product = phosphorylase kinase alphabeta
GKJWQY101AVJYK	K01537	Ca2+-transporting ATPase [EC:3.6.3.8]	Calcium signaling pathway	482	1	482	0.0E+00	100%	355393429	Bacteria	Firmicutes	Bacilli	Lactobacillus rhamnosus ATCC 8530, complete genome, product = HAD ATPase, P-type, IC family protein
GKJWQY101AU07B	K00855	phosphoribulokinase [EC:2.7.1.19]	Carbon fixation in photosynthetic organisms	501	1	501	0.0E+00	95%	428238862	Bacteria	Cyanobacteria	n	Oscillatoria nigro-viridis PCC 7112, complete genome, product = phosphoribulokinase

GKJWQY101BZ4H8	K03737	putative pyruvate-flavodoxin oxidoreductase [EC:1.2.7.-]	Carbon fixation pathways in prokaryotes	442	1	442	4.0E-140	87%	29342101	Bacteria	Bacteroidetes	Bacteroidia	Bacteroides thetaiotaomicron VPI-5482, complete genome, product = pyruvate-flavodoxin oxidoreductase
GKJWQY101A6S1N	K03737	putative pyruvate-flavodoxin oxidoreductase [EC:1.2.7.-]	Carbon fixation pathways in prokaryotes	390	1	390	1.0E-99	84%	295083795	Bacteria	Bacteroidetes	Bacteroidia	Bacteroides xylanisolvens XB1A draft genome, product = pyruvate:ferredoxin (flavodoxin) oxidoreductase, homodimeric
GKJWQY101AR4JZ	K01358	ATP-dependent Clp protease, protease subunit [EC:3.4.21.92]	Cell cycle - Caulobacter	484	1	484	0.0E+00	98%	428238862	Bacteria	Cyanobacteria	n	Oscillatoria nigro-viridis PCC 7112, complete genome, product = ATP-dependent Clp protease proteolytic subunit ClpP
GKJWQY101BDWER	K01338	ATP-dependent Lon protease [EC:3.4.21.53]	Cell cycle - Caulobacter	514	1	514	3.0E-162	87%	433663430	Bacteria	Proteobacteria	Alphaproteobacteria	Mesorhizobium australicum WSM2073, complete genome, product = ATP-dependent protease La
GKJWQY101BFHGL	K03544	ATP-dependent Clp protease ATP-binding subunit ClpX	Cell cycle - Caulobacter	461	1	461	0.0E+00	99%	387575654	Bacteria	Proteobacteria	Betaproteobacteria	Burkholderia sp. KJ006 chromosome 1, complete sequence, product = ATP-dependent Clp protease ATP-binding subunit ClpX
GKJWQY101BK9HC	K06666	glucose repression regulatory protein TUP1	Cell cycle - yeast	480	1	480	1.0E-41	63%	427716641	Bacteria	Cyanobacteria	n	WD40 repeat-containing protein [Calothrix sp. PCC 7507]
GKJWQY101AKTZ2	K00148	glutathione-independent formaldehyde dehydrogenase [EC:1.2.1.46]	Chloroalkane and chloroalkene degradation/Methane metabolism	506	1	506	0.0E+00	98%	133737197	Bacteria	Proteobacteria	Betaproteobacteria	Herminiimonas arsenicoxydans chromosome, complete sequence, product = glutathione-independent formaldehyde dehydrogenase (FDH) (FALDH)
GKJWQY101AVMAD	K00148	glutathione-independent formaldehyde dehydrogenase [EC:1.2.1.46]	Chloroalkane and chloroalkene degradation/Methane metabolism	483	1	483	0.0E+00	98%	133737197	Bacteria	Proteobacteria	Betaproteobacteria	Herminiimonas arsenicoxydans chromosome, complete sequence, product = glutathione-independent formaldehyde dehydrogenase (FDH) (FALDH)
GKJWQY101BLVZX	K00148	glutathione-independent formaldehyde dehydrogenase [EC:1.2.1.46]	Chloroalkane and chloroalkene degradation/Methane metabolism	427	1	427	0.0E+00	99%	133737197	Bacteria	Proteobacteria	Betaproteobacteria	Herminiimonas arsenicoxydans chromosome, complete sequence, product = glutathione-independent formaldehyde dehydrogenase (FDH) (FALDH)
GKJWQY101AO712_2	K01563	haloalkane dehalogenase	Chlorocyclohexane and chlorobenzene degradation/Chloroalkane and chloroalkene degradation	178	1	178	8.0E-69	94%	428238862	Bacteria	Cyanobacteria	n	Oscillatoria nigro-viridis PCC 7112, complete genome, product = alpha/beta hydrolase fold protein
GKJWQY101AJE3X	K01802	peptidylprolyl isomerase [EC:5.2.1.8]	cis-trans-Isomerases	480	1	480	0.0E+00	94%	428238862	Bacteria	Cyanobacteria	n	Oscillatoria nigro-viridis PCC 7112, complete genome, product = peptidyl-prolyl cis-trans isomerase cyclophilin type
GKJWQY101BQ51J	K01676	fumarate hydratase, class I [EC:4.2.1.2]	Citrate cycle (TCA cycle)/Carbon fixation pathways in prokaryotes	307	1	307	1.0E-14	72%	167596226	Bacteria	Proteobacteria	Gammaproteobacteria	Francisella philomiragia subsp. philomiragia ATCC 25017, complete genome, product = fumarate hydratase, class I
GKJWQY101ADVWE	K00031	isocitrate dehydrogenase [EC:1.1.1.42]	Citrate cycle (TCA cycle)/Glutathione metabolism/Carbon fixation pathways in prokaryotes/	507	15	507	2.0E-64	76%	72493824	Bacteria	Firmicutes	Bacilli	Staphylococcus saprophyticus subsp. saprophyticus ATCC 15305 DNA, complete genome, product = isocitrate dehydrogenase
GKJWQY101AM1FT	K00031	isocitrate dehydrogenase [EC:1.1.1.42]	Citrate cycle (TCA cycle)/Glutathione metabolism/Carbon fixation pathways in prokaryotes/	474	14	474	0.0E+00	99%	387575654	Bacteria	Proteobacteria	Betaproteobacteria	Burkholderia sp. KJ006 chromosome 1, complete sequence, product = isocitrate dehydrogenase
GKJWQY101A6F8L	K00658	2-oxoglutarate dehydrogenase E2 component (dihydrolipoamide succinyltransferase) [EC:2.3.1.61]	Citrate cycle (TCA cycle)/Lysine degradation	481	1	481	0.0E+00	91%	469772332	Bacteria	Proteobacteria	Betaproteobacteria	Ralstonia solanacearum FQY_4, complete genome, product = dihydrolipoamide succinyltransferase component (E2) of 2-oxoglutarate dehydrogenase complex(<1..325); dihydrolipoamide dehydrogenase of 2-oxoglutarate dehydrogenase (396..>479)
GKJWQY101AHUTJ	K00164	2-oxoglutarate dehydrogenase E1 component [EC:1.2.4.2]	Citrate cycle (TCA cycle)/Lysine degradation/Tryptophan metabolism	348	1	348	6.0E-108	87%	71037566	Bacteria	Proteobacteria	Gammaproteobacteria	Psychrobacter arcticus 273-4, complete genome, product = 2-oxoglutarate dehydrogenase E1 component
GKJWQY101BIWNZ	K00240	succinate dehydrogenase iron-sulfur protein [EC:1.3.99.1]	Citrate cycle (TCA cycle)/Oxidative phosphorylation	394	1	394	0.0E+00	96%	299070035	Bacteria	Proteobacteria	Betaproteobacteria	Ralstonia solanacearum CFBP2957 chromosome complete genome, product = succinate dehydrogenase, Fe-S protein
GKJWQY101A05N9	K00558	DNA (cytosine-5-)-methyltransferase [EC:2.1.1.37]	Cysteine and methionine metabolism	344	1	344	3.0E-141	94%	428238862	Bacteria	Cyanobacteria	n	Oscillatoria nigro-viridis PCC 7112, complete genome, product = DNA-cytosine methyltransferase
GKJWQY101A15DF	K06997	Possibly alanine racemase, N-terminal	D-Alanine metabolism	319	1	319	8.0E-47	78%	156307499	Eukaryota	Cnidaria	Anthozoa	Nematostella vectensis predicted protein (NEMVEDRAFT_v1g225918) partial mRNA (note: note = pyridoxal 5'-phosphate (PLP) binding site)
GKJWQY101BICX6	K01776	glutamate racemase [EC:5.1.1.3]	D-Glutamine and D-glutamate metabolism	465	1	465	0.0E+00	95%	428238862	Bacteria	Cyanobacteria	n	Oscillatoria nigro-viridis PCC 7112, complete genome, product = glutamate racemase
GKJWQY101AUR1Y	K01925	UDP-N-acetylmuramoylalanine--D-glutamate ligase [EC:6.3.2.9]	D-Glutamine and D-glutamate metabolism/Peptidoglycan biosynthesis	495	1	495	0.0E+00	99%	325124855	Bacteria	Firmicutes	Bacilli	Lactobacillus delbrueckii subsp. bulgaricus 2038, complete genome, product = UDP-N-acetylmuramoylalanine--D-glutamate ligase
GKJWQY101ALDHC	K03502	DNA polymerase V	DNA repair and recombination proteins	165	1	165	3.0E-78	99%	3582195	Bacteria	Firmicutes	Bacilli	Lactococcus lactis strain DPC3147 plasmid pMRC01, complete sequence (conserved hypothetical protein, ORFU)
GKJWQY101ADMQY	K01356	repressor LexA [EC:3.4.21.88]	DNA repair and recombination proteins	503	1	503	0.0E+00	99%	312279338	Bacteria	Firmicutes	Bacilli	Lactobacillus delbrueckii subsp. bulgaricus ND02, complete genome, product = LexA repressor
GKJWQY101BKUOE	K02314	replicative DNA helicase [EC:3.6.4.12]	DNA replication/Cell cycle - Caulobacter	433	1	433	0.0E+00	100%	355393429	Bacteria	Firmicutes	Bacilli	Lactobacillus rhamnosus ATCC 8530, complete genome, product = replicative DNA helicase
GKJWQY101AJLH0	K02314	replicative DNA helicase [EC:3.6.4.12]	DNA replication/Cell cycle - Caulobacter	337	1	337	3.0E-136	93%	300072131	Bacteria	Proteobacteria	Betaproteobacteria	Herbaspirillum seropedicae SmR1, complete genome, product = replicative DNA helicase protein
GKJWQY101APN55	K02469	DNA gyrase subunit A [EC:5.99.1.3]	DNA replication/DNA repair and recombination	518	1	518	1.0E-140	85%	78609255	Bacteria	Firmicutes	Bacilli	Lactobacillus sakei strain 23K complete genome, product = DNA gyrase, A subunit
GKJWQY101A1C2A	K03111	single-strand DNA-binding protein	DNA replication/Mismatch repair/Homologous recombination	437	1	437	0.0E+00	94%	428238862	Bacteria	Cyanobacteria	n	Oscillatoria nigro-viridis PCC 7112, complete genome, product = single-strand binding protein
GKJWQY101AESM7	K01422	E3.4.99.- (found through discontiguous megablast)	Endopeptidases of unknown catalytic mechanism	504	1	504	1.0E-42	70%	149935098	Bacteria	Bacteroidetes	Bacteroidia	Parabacteroides distasonis ATCC 8503, complete genome, product = putative zinc protease YmxG
GKJWQY101BQ1WY	K01147	exoribonuclease II [EC:3.1.13.1]	Exoribonucleases producing 5'-phosphomonoesters	381	1	381	5.0E-109	86%	300072131	Bacteria	Proteobacteria	Betaproteobacteria	Herbaspirillum seropedicae SmR1, complete genome, product = exoribonuclease R protein (function = K - Transcription)
GKJWQY101BPW6B	K00648	3-oxoacyl-[acyl-carrier-protein] synthase III [EC:2.3.1.180]	Fatty acid biosynthesis	243	1	243	7.0E-96	93%	145902672	Bacteria	Firmicutes	Bacilli	Bacillus licheniformis ATCC 14580, complete genome, product = beta-ketoacyl-acyl carrier protein synthase III
GKJWQY101BGY07	K02371	enoyl-[acyl carrier protein] reductase II [EC:1.3.1.-]	Fatty acid biosynthesis	335	1	335	2.0E-172	99%	355393429	Bacteria	Firmicutes	Bacilli	Lactobacillus rhamnosus ATCC 8530, complete genome, product = enoyl-(Acyl-carrier-protein) reductase (<1..253); acyl carrier protein 2 (276..>335)
GKJWQY101A3SQJ	K01716	3-hydroxydecanoyl-[acyl-carrier-protein] dehydratase [EC:4.2.1.60]	Fatty acid biosynthesis	258	1	258	1.0E-69	86%	365177649	Bacteria	Proteobacteria	Alphaproteobacteria	Sinorhizobium fredii HH103 main chromosome, complete sequence, product = 3-hydroxydecanoyl-(acyl carrier protein) dehydratase
GKJWQY101BM1CY	K00208	enoyl-[acyl-carrier protein] reductase I [EC:1.3.1.9]	Fatty acid biosynthesis/Biotin metabolism	478	1	478	2.00E-148	87%	433663430	Bacteria	Proteobacteria	Alphaproteobacteria	Mesorhizobium australicum WSM2073, complete genome, product = enoyl-(acyl-carrier-protein) reductase (NADH)
GKJWQY101BMNR7	K00208	enoyl-[acyl-carrier protein] reductase I [EC:1.3.1.9]	Fatty acid biosynthesis/Biotin metabolism	435	1	435	6.00E-129	86%	433663430	Bacteria	Proteobacteria	Alphaproteobacteria	Mesorhizobium australicum WSM2073, complete genome, product = enoyl-(acyl-carrier-protein) reductase (NADH)
GKJWQY101BVV9P	K02372	3R-hydroxymyristoyl ACP dehydrase [EC:4.2.1.-]	Fatty acid biosynthesis/Biotin metabolism	227	1	227	9.0E-30	78%	326411376	Bacteria	Proteobacteria	Alphaproteobacteria	Polymorphum gilvum SL003B-26A1, complete genome, product = 3R)-hydroxymyristoyl-[acyl-carrier-protein] dehydratase
GKJWQY101ALM6K	K00059	3-oxoacyl-[acyl-carrier protein] reductase [EC:1.1.1.100]	Fatty acid biosynthesis/Biotin metabolism/Biosynthesis of unsaturated fatty acids	464	1	464	8.0E-78	79%	186463002	Bacteria	Cyanobacteria	n	Nostoc punctiforme PCC 73102, complete genome, product = short-chain dehydrogenase/reductase SDR
GKJWQY101BFXP6	K00059	3-oxoacyl-[acyl-carrier protein] reductase [EC:1.1.1.100]	Fatty acid biosynthesis/Biotin metabolism/Biosynthesis of unsaturated fatty acids	490	1	490	4.0E-146	86%	428238862	Bacteria	Cyanobacteria	n	Oscillatoria nigro-viridis PCC 7112, complete genome, product = 3-oxoacyl-(acyl-carrier-protein) reductase

GKJWQY101AOPRD	K00059	3-oxoacyl-[acyl-carrier protein] reductase [EC:1.1.1.100]	Fatty acid biosynthesis/Biotin metabolism/Biosynthesis of unsaturated fatty acids	404	1	404	1.0E-90	82%	170937689	Bacteria	Proteobacteria	Betaproteobacteria	Cupriavidus taiwanensis str. LMG19424 chromosome 1, complete genome, product = putative dehydrogenase/reductase
GKJWQY101BA0SO	K07511	enoyl-CoA hydratase [EC:4.2.1.17]	Fatty acid elongation	430	1	430	0.0E+00	97%	387578572	Bacteria	Proteobacteria	Betaproteobacteria	Burkholderia sp. KJ006 chromosome 2, complete sequence, product = 3-hydroxybutyryl-CoA dehydratase
GKJWQY101BXDHE	K01897	long-chain acyl-CoA synthetase [EC:6.2.1.3]	Fatty acid metabolism/Peroxisome /PPAR signaling pathway	295	1	295	7.0E-22	74%	433663430	Bacteria	Proteobacteria	Alphaproteobacteria	Mesorhizobium australicum WSM2073, complete genome, product = acyl-CoA synthetase (AMP-forming)/AMP-acid ligase II
GKJWQY101AQNM9	K01897	long-chain acyl-CoA synthetase [EC:6.2.1.3]	Fatty acid metabolism/Peroxisome /PPAR signaling pathway	511	1	511	0.0E+00	99%	387578572	Bacteria	Proteobacteria	Betaproteobacteria	Burkholderia sp. KJ006 chromosome 2, complete sequence, product = long-chain-fatty-acid--CoA ligase
GKJWQY101BLPEJ	K01897	long-chain acyl-CoA synthetase [EC:6.2.1.3]	Fatty acid metabolism/Peroxisome /PPAR signaling pathway	522	1	522	0.0E+00	99%	387578572	Bacteria	Proteobacteria	Betaproteobacteria	Burkholderia sp. KJ006 chromosome 2, complete sequence, product = long-chain-fatty-acid--CoA ligase
GKJWQY101BIW4Y	K00754	[EC:2.4.1.-]	Fructose and mannose metabolism	484	1	484	0.0E+00	92%	428238862	Bacteria	Cyanobacteria	n	Oscillatoria nigro-viridis PCC 7112, complete genome, product = glycosyl transferase, WecB/TagA/CpsF family (teichoic acid biosynthesis)
GKJWQY101A090Z	K00971	mannose-1-phosphate guanylyltransferase [EC:2.7.7.22]	Fructose and mannose metabolism/Amino sugar and nucleotide sugar metabolism	409	1	409	0.0E+00	96%	428238862	Bacteria	Cyanobacteria	n	Oscillatoria nigro-viridis PCC 7112, complete genome, product = mannose-1-phosphate guanylyltransferase
GKJWQY101B1CPM	K00965	UDPglucose--hexose-1-phosphate uridylyltransferase [EC:2.7.7.12]	Galactose metabolism/Amino sugar and nucleotide sugar metabolism	392	1	392	0.0E+00	98%	355393429	Bacteria	Firmicutes	Bacilli	Lactobacillus rhamnosus ATCC 8530, complete genome, product = galactose-1-phosphate uridylyltransferase
GKJWQY101BS0KQ	K01193	beta-fructofuranosidase [EC:3.2.1.26]	Galactose metabolism/Starch and sucrose metabolism	346	1	346	2.0E-178	99%	443424428	Bacteria	Firmicutes	Bacilli	Staphylococcus warneri SG1, complete genome, product = sucrose-6-phosphate hydrolase (note= COG1621 Beta-fructosidases (levanase/invertase)
GKJWQY101B1CAN	K00257	[EC:1.3.99.-]	Geraniol degradation	467	1	467	0.0E+00	100%	387575654	Bacteria	Proteobacteria	Betaproteobacteria	Burkholderia sp. KJ006 chromosome 1, complete sequence, product = acyl-CoA dehydrogenase
GKJWQY101BV1WT	K00257	[EC:1.3.99.-]	Geraniol degradation	462	1	462	0.0E+00	100%	387575654	Bacteria	Proteobacteria	Betaproteobacteria	Burkholderia sp. KJ006 chromosome 1, complete sequence, product = acyl-CoA dehydrogenase
GKJWQY101BPRRL	K00257	[EC:1.3.99.-]	Geraniol degradation	479	1	479	1.0E-180	91%	206593202	Bacteria	Proteobacteria	Betaproteobacteria	Ralstonia solanacearum strain IPO1609 Genome Draft, product = acyl-coa dehydrogenase protein
GKJWQY101BYCTV	K01255	leucyl aminopeptidase [EC:3.4.11.1]	Glutathione metabolism	494	1	494	0.0E+00	92%	428238862	Bacteria	Cyanobacteria	n	Oscillatoria nigro-viridis PCC 7112, complete genome, product = leucyl aminopeptidase
GKJWQY101AK7G8	K00799	glutathione S-transferase [EC:2.5.1.18]	Glutathione metabolism/Metabolism of xenobiotics by cytochrome P450	452	1	452	3.0E-107	83%	299065054	Bacteria	Proteobacteria	Betaproteobacteria	Ralstonia solanacearum str. CMR15 chromosome, complete genome, product = glutathione S-transferase
GKJWQY101BP3UP	K00681	gamma-glutamyltranspeptidase [EC:2.3.2.2]	Glutathione metabolism/Taurine and hypotaurine metabolism/Cyanoamino acid metabolism/Arachidonic acid metabolism	192	1	192	4.0E-93	99%	145902672	Bacteria	Firmicutes	Bacilli	Bacillus licheniformis ATCC 14580, complete genome, product = peptidase T3, gamma-glutamyltranspeptidase
GKJWQY101AX7BR	K07407	alpha-galactosidase [EC:3.2.1.22]	Glycerolipid metabolism	386	1	386	0.0E+00	98%	257149867	Bacteria	Firmicutes	Bacilli	Lactobacillus rhamnosus Lc 705 whole genome sequence, strain Lc 705, product = alpha-galactosidase (GH36)
GKJWQY101A5XCC	K00005	glycerol dehydrogenase [EC:1.1.1.6]	Glycerolipid metabolism	547	14	547	0.0E+00	95%	428238862	Bacteria	Cyanobacteria	n	Oscillatoria nigro-viridis PCC 7112, complete genome, product = glycerol dehydrogenase
GKJWQY101BK048	K00057	glycerol-3-phosphate dehydrogenase (NAD(P)+) [EC:1.1.1.94]	Glycerophospholipid metabolism	326	1	326	4.0E-124	92%	428238862	Bacteria	Cyanobacteria	n	Oscillatoria nigro-viridis PCC 7112, complete genome, product = glycerol-3-phosphate dehydrogenase (NAD(P)+)
GKJWQY101BWIVS	K00872	homoserine kinase [EC:2.7.1.39]	Glycine, serine and threonine metabolism	483	1	483	0.0E+00	99%	355393429	Bacteria	Firmicutes	Bacilli	Lactobacillus rhamnosus ATCC 8530, complete genome, product = homoserine kinase
GKJWQY101A43VY	K06001	tryptophan synthase beta chain [EC:4.2.1.20]	Glycine, serine and threonine metabolism	439	1	439	0.0E+00	99%	355393429	Bacteria	Firmicutes	Bacilli	Lactobacillus rhamnosus ATCC 8530, complete genome, product = tryptophan synthase, beta subunit
GKJWQY101AY8NU	K00302	sarcosine oxidase, subunit alpha [EC:1.5.3.1]	Glycine, serine and threonine metabolism	490	1	490	5.0E-165	88%	317165637	Bacteria	Proteobacteria	Alphaproteobacteria	Mesorhizobium ciceri biovar biserrulae WSM1271, complete genome, product = sarcosine oxidase, alpha subunit family protein
GKJWQY101AMFXC	K00108	choline dehydrogenase [EC:1.1.99.1]	Glycine, serine and threonine metabolism	455	13	455	2.0E-124	85%	433663430	Bacteria	Proteobacteria	Alphaproteobacteria	Mesorhizobium australicum WSM2073, complete genome, product = choline dehydrogenase-like flavoprotein
GKJWQY101ASOB7	K00281	glycine dehydrogenase [EC:1.4.4.2]	Glycine, serine and threonine metabolism	174	1	174	6.0E-30	81%	83630956	Bacteria	Proteobacteria	Gammaproteobacteria	Hahella chejuensis KCTC 2396, complete genome, product = glycine dehydrogenase
GKJWQY101BBP73	K00058	D-3-phosphoglycerate dehydrogenase [EC:1.1.1.95]	Glycine, serine and threonine metabolism/ Methane metabolism	528	1	525	0.0E+00	96%	103422338	Bacteria	Firmicutes	Bacilli	Lactobacillus delbrueckii subsp. bulgaricus ATCC 11842 complete genome, product = Phosphoglycerate dehydrogenase (<1..413); Phosphoserine aminotransferase (432..>524)
GKJWQY101ATR7M	K00133	aspartate-semialdehyde dehydrogenase [EC:1.2.1.11]	Glycine, serine and threonine metabolism/Cysteine and methionine metabolism/Lysine biosynthesis	381	1	381	1.0E-124	88%	336024847	Bacteria	Proteobacteria	Alphaproteobacteria	Mesorhizobium opportunistum WSM2075, complete genome, product = aspartate-semialdehyde dehydrogenase
GKJWQY101A7G4R	K00134	glyceraldehyde 3-phosphate dehydrogenase [EC:1.2.1.12]	Glycolysis-Gluconeogenesis	518	1	517	0.0E+00	97%	413973243	Bacteria	Firmicutes	Bacilli	Lactococcus lactis subsp. cremoris UC509.9, complete genome, product = putative alkylphosphonate uptake protein (<1..42); glyceraldehyde 3-phosphate dehydrogenase (110..>516)
GKJWQY101BNEJ6	K00134	glyceraldehyde 3-phosphate dehydrogenase [EC:1.2.1.12]	Glycolysis-Gluconeogenesis	413	1	413	0.0E+00	99%	413973243	Bacteria	Firmicutes	Bacilli	Lactococcus lactis subsp. cremoris UC509.9, complete genome, product = glyceraldehyde 3-phosphate dehydrogenase (8..414)
GKJWQY101BTPQT	K00131	glyceraldehyde-3-phosphate dehydrogenase (NADP) [EC:1.2.1.9]	Glycolysis-Gluconeogenesis	471	1	471	0.0E+00	94%	406368402	Bacteria	Firmicutes	Bacilli	Streptococcus pneumoniae gamPNI0373, complete genome, product = glyceraldehyde-3-phosphate dehydrogenase (NADP+)
GKJWQY101ASDW8	K00134	glyceraldehyde 3-phosphate dehydrogenase [EC:1.2.1.12]	Glycolysis-Gluconeogenesis	458	1	458	7.0E-168	90%	111606883	Bacteria	Proteobacteria	Alphaproteobacteria	Aminobacter aminovorans partial gapA gene for glyceraldehyde-3-phosphate dehydrogenase, strain DSM 10368
GKJWQY101B0A0U	K00134	glyceraldehyde 3-phosphate dehydrogenase [EC:1.2.1.12]	Glycolysis-Gluconeogenesis	477	1	477	3.0E-172	90%	111606883	Bacteria	Proteobacteria	Alphaproteobacteria	Aminobacter aminovorans partial gapA gene for glyceraldehyde-3-phosphate dehydrogenase, strain DSM 10368
GKJWQY101A4OMR	K00134	glyceraldehyde 3-phosphate dehydrogenase [EC:1.2.1.12]	Glycolysis-Gluconeogenesis	480	1	480	4.0E-116	83%	343461404	Bacteria	Proteobacteria	Alphaproteobacteria	Ketogulonigenium vulgarum WSH-001, complete genome, product = glyceraldehyde-3-phosphate dehydrogenase B
GKJWQY101A4IEA	K00627	pyruvate dehydrogenase E2 component (dihydrolipoamide acetyltransferase) [EC:2.3.1.12]	Glycolysis-Gluconeogenesis/Citrate cycle (TCA cycle)/Pyruvate metabolism	433	1	433	0.0E+00	97%	387575654	Bacteria	Proteobacteria	Betaproteobacteria	Burkholderia sp. KJ006 chromosome 1, complete sequence, product = dihydrolipoamide acetyltransferase component of pyruvate dehydrogenase complex
GKJWQY101AYI7F	K00163	pyruvate dehydrogenase E1 component [EC:1.2.4.1]	Glycolysis-Gluconeogenesis/Citrate cycle (TCA cycle)/Pyruvate metabolism/Butanoate metabolism	479	1	479	0.0E+00	99%	387575654	Bacteria	Proteobacteria	Betaproteobacteria	Burkholderia sp. KJ006 chromosome 1, complete sequence, product = pyruvate dehydrogenase E1 component
GKJWQY101BVMGW	K00162	pyruvate dehydrogenase E1 component subunit beta [EC:1.2.4.1]	Glycolysis-Gluconeogenesis/Citrate cycle (TCA cycle)/Pyruvate metabolism/Butanoate metabolism/HIF-1 signaling pathway	318	1	318	4.00E-159	99%	392602377	Bacteria	Firmicutes	Bacilli	Streptococcus mutans GS-5, complete genome, product = branched-chain alpha-keto acid dehydrogenase subunit E2 (<1..105); pyruvate dehydrogenase E1 component beta subunit (118..>316)
GKJWQY101BGJYW	K01610	phosphoenolpyruvate carboxykinase (ATP) [EC:4.1.1.49]	Glycolysis-Gluconeogenesis/Citrate cycle (TCA cycle)/Pyruvate metabolism/Carbon fixation in photosynthetic organisms	510	1	510	8.0E-163	87%	301161079	Bacteria	Bacteroidetes	Bacteroidia	Bacteroides fragilis 638R genome, product = putative phosphoenolpyruvate carboxykinase
GKJWQY101A0Q2Y	K01689	enolase [EC:4.2.1.11]	Glycolysis-Gluconeogenesis/Methane metabolism/RNA degradation/HIF-1 signaling pathway	235	1	235	1.0E-118	100%	257149867	Bacteria	Firmicutes	Bacilli	Lactobacillus rhamnosus Lc 705 whole genome sequence, strain Lc705, product = enolase

GKJWQY101BGU7F	K00128	aldehyde dehydrogenase (NAD+) [EC:1.2.1.3]	Glycolysis-Gluconeogenesis/Pentose and glucuronate interconversions/Ascorbate and aldarate metabolism/Fatty acid metabolism/Valine, leucine and isoleucinedegradation/Lysine degradation/Arginine and proline metabolism/Histidine metabolism/Tryptophan metabolism/beta-Alanine metabolism/Glycerolipid metabolism/Pyruvate metabolism/Chloroalkane and chloroalkene degradation/Propanoate metabolism/Limonene and pinene degradation	338	1	338	9.0E-156	96%	428238862	Bacteria	Cyanobacteria	n	Oscillatoria nigro-viridis PCC 7112, complete genome, product = aldehyde dehydrogenase
GKJWQY101BV866	K00128	aldehyde dehydrogenase (NAD+) [EC:1.2.1.3]	Glycolysis-Gluconeogenesis/Pentose and glucuronate interconversions/Ascorbate and aldarate metabolism/Fatty acid metabolism/Valine, leucine and isoleucinedegradation/Lysine degradation/Arginine and proline metabolism/Histidine metabolism/Tryptophan metabolism/beta-Alanine metabolism/Glycerolipid metabolism/Pyruvate metabolism/Chloroalkane and chloroalkene degradation/Propanoate metabolism/Limonene and pinene degradation	245	3	245	4.0E-83	90%	428238862	Bacteria	Cyanobacteria	n	Oscillatoria nigro-viridis PCC 7112, complete genome, product = aldehyde dehydrogenase
GKJWQY101BHVBO	K00128	aldehyde dehydrogenase (NAD+) [EC:1.2.1.3]	Glycolysis-Gluconeogenesis/Pentose and glucuronate interconversions/Ascorbate and aldarate metabolism/Fatty acid metabolism/Valine, leucine and isoleucinedegradation/Lysine degradation/Arginine and proline metabolism/Histidine metabolism/Tryptophan metabolism/beta-Alanine metabolism/Glycerolipid metabolism/Pyruvate metabolism/Chloroalkane and chloroalkene degradation/Propanoate metabolism/Limonene and pinene degradation	495	1	495	9.0E-98	80%	227337257	Bacteria	Proteobacteria	Alphaproteobacteria	Sinorhizobium fredii NGR234 plasmid pNGR234b, complete sequence, product = aldehyde dehydrogenase
GKJWQY101BQ494	K01810	glucose-6-phosphate isomerase [EC:5.3.1.9]	Glycolysis-Gluconeogenesis/Pentose phosphate pathway/Starch and sucrose metabolism/Amino sugar and nucleotide sugar metabolism	357	1	357	1.0E-175	98%	428238862	Bacteria	Cyanobacteria	n	Oscillatoria nigro-viridis PCC 7112, complete genome, product = glucose 6-phosphate isomerase
GKJWQY101BAZHJ	K01895	acetyl-CoA synthetase [EC:6.2.1.1]	Glycolysis-Gluconeogenesis/Pyruvate metabolism/Propanoate metabolism/Carbon fixation pathways in prokaryotes/Methane metabolism	296	1	296	9.0E-91	88%	336024847	Bacteria	Proteobacteria	Alphaproteobacteria	Mesorhizobium opportunistum WSM2075, complete genome, product = acetate--CoA ligase
GKJWQY101BG1OI	K01895	acetyl-CoA synthetase [EC:6.2.1.1]	Glycolysis-Gluconeogenesis/Pyruvate metabolism/Propanoate metabolism/Carbon fixation pathways in prokaryotes/Methane metabolism	363	1	363	5.0E-19	73%	385869619	Bacteria	Proteobacteria	Gammaproteobacteria	Pectobacterium sp. SCC3193, complete genome, product = acetyl-coenzyme A synthetase
GKJWQY101ALV77	K01235	alpha-glucuronidase [EC:3.2.1.139]	Glycosidases[hydrolyse O- and S-glycosyl]	403	1	403	5.0E-20	72%	433303004	Bacteria	Bacteroidetes	Bacteroidia	Prevotella dentalis DSM 3688 chromosome 2, complete sequence, product = alpha-glucuronidase
GKJWQY101AFEH7	K01200	pullulanase [EC:3.2.1.41]	Glycosidases[hydrolyse O- and S-glycosyl]	566	1	566	0.0E+00	98%	296848933	Bacteria	Deinococcus-Thermus	Deinococci	Meiothermus silvanus DSM 9946, complete genome, product = alpha-1,6-glucosidase, pullulanase-type
GKJWQY101B1465	K01608	tartronate-semialdehyde synthase [EC:4.1.1.47]	Glyoxylate and dicarboxylate metabolism	287	1	287	8.0E-61	82%	327367349	Bacteria	Proteobacteria	Betaproteobacteria	Burkholderia gladioli BSR3 chromosome 1, complete sequence, product = glyoxylate carboligase
GKJWQY101AIKIJ	K11472	glycolate oxidase FAD binding subunit	Glyoxylate and dicarboxylate metabolism	391	1	391	4.0E-180	96%	428238862	Bacteria	Cyanobacteria	n	Oscillatoria nigro-viridis PCC 7112, complete genome, product = FAD linked oxidase domain protein
GKJWQY101BRYKR	K01715	3-hydroxybutyryl-CoA dehydratase [EC:4.2.1.55]	Glyoxylate and dicarboxylate metabolism/Butanoate metabolism/Carbon fixation pathways in prokaryotes	432	1	432	0.0E+00	99%	387578572	Bacteria	Proteobacteria	Betaproteobacteria	Burkholderia sp. KJ006 chromosome 2, complete sequence, product = 3-hydroxybutyryl-CoA dehydratase
GKJWQY101AH3TL	K00123	formate dehydrogenase, alpha subunit [EC:1.2.1.2]	Glyoxylate and dicarboxylate metabolism/Methane metabolism	348	1	348	2.0E-176	99%	387578572	Bacteria	Proteobacteria	Betaproteobacteria	Burkholderia sp. KJ006 chromosome 2, complete sequence, product = formate dehydrogenase O beta subunit (1..25); formate dehydrogenase O alpha subunit (36..349)
GKJWQY101AH3TL_2	K00123	formate dehydrogenase, alpha subunit [EC:1.2.1.2]	Glyoxylate and dicarboxylate metabolism/Methane metabolism	116	1	116	3.0E-46	97%	387578572	Bacteria	Proteobacteria	Betaproteobacteria	Burkholderia sp. KJ006 chromosome 2, complete sequence, product = formate dehydrogenase O alpha subunit
GKJWQY101AZSAO	K00124	formate dehydrogenase, beta subunit [EC:1.2.1.2]	Glyoxylate and dicarboxylate metabolism/Methane metabolism	267	2	267	9.0E-100	92%	334194119	Bacteria	Proteobacteria	Betaproteobacteria	Ralstonia solanacearum Po82, complete genome, product = formate dehydrogenase iron-sulfur subunit
GKJWQY101AIW11	K01915	glutamine synthetase [EC:6.3.1.2]	Glyoxylate and dicarboxylate metabolism/Nitrogen metabolism/Alanine, aspartate and glutamate metabolism/Arginine and proline metabolism/Two-component system	486	1	486	2.0E-149	87%	269095543	Bacteria	Actinobacteria	Actinobacteria	Sanguibacter keddieii DSM 10542, complete genome, product = L-glutamine synthetase
GKJWQY101BRVH2	K00013	histidinol dehydrogenase [EC:1.1.1.23]	Histidine metabolism	494	1	494	6.0E-164	89%	428238862	Bacteria	Cyanobacteria	n	Oscillatoria nigro-viridis PCC 7112, complete genome, product = histidinol dehydrogenase
GKJWQY101BLV08	K02500	cyclase [EC:4.1.3.-]	Histidine metabolism	499	1	499	0.0E+00	93%	428238862	Bacteria	Cyanobacteria	n	Oscillatoria nigro-viridis PCC 7112, complete genome, product = imidazole glycerol phosphate synthase subunit hisF
GKJWQY101AQTYB	K00765	ATP phosphoribosyltransferase [EC:2.4.2.17]	Histidine metabolism	352	1	352	3.0E-170	98%	355393429	Bacteria	Firmicutes	Bacilli	Lactobacillus rhamnosus ATCC 8530, complete genome, product = ATP phosphoribosyltransferase

GKJWQY101AD3KT	K02501	glutamine amidotransferase [EC:2.4.2.-]	Histidine metabolism	508	1	508	0.0E+00	99%	387575654	Bacteria	Proteobacteria	Betaproteobacteria	Burkholderia sp. KJ006 chromosome 1, complete sequence, product = imidazole glycerol phosphate synthase amidotransferase su...
GKJWQY101BZKZO	K00817	histidinol-phosphate aminotransferase [EC:2.6.1.9]	Histidine metabolism/Tyrosine metabolism/Phenylalanine metabolism/Phenylalanine, tyrosine and tryptophan biosynthesis/Novobiocin biosynthesis/Tropane, piperidine and pyridine alkaloid biosynthesis	481	1	481	0.0E+00	99%	355393429	Bacteria	Firmicutes	Bacilli	Lactobacillus rhamnosus ATCC 8530, complete genome, product = histidinol-phosphate transaminase
GKJWQY101B1I99	K03553	recombination protein RecA	Homologous recombination	343	1	343	2.0E-152	96%	428238862	Bacteria	Cyanobacteria	n	Oscillatoria nigro-viridis PCC 7112, complete genome, product = protein recA
GKJWQY101BWEV4	K04066	primosomal protein N' (replication factor Y) (superfamily II helicase) [EC:3.6.4.-]	Homologous recombination	473	1	473	0.0E+00	96%	428238862	Bacteria	Cyanobacteria	n	Oscillatoria nigro-viridis PCC 7112, complete genome, product = replication restart DNA helicase PriA
GKJWQY101BZIGR	K04066	primosomal protein N' (replication factor Y) (superfamily II helicase) [EC:3.6.4.-]	Homologous recombination	498	1	498	0.0E+00	94%	428238862	Bacteria	Cyanobacteria	n	Oscillatoria nigro-viridis PCC 7112, complete genome, product = replication restart DNA helicase PriA
GKJWQY101A6L2S	K03655	ATP-dependent DNA helicase RecG [EC:3.6.4.12]	Homologous recombination	344	1	344	2.0E-177	99%	413973243	Bacteria	Firmicutes	Bacilli	Lactococcus lactis subsp. cremoris UC509.9, complete genome, product = ATP-dependent DNA helicase RecG
GKJWQY101AM4OJ	K03336	3D-(3,5/4)-trihydroxycyclohexane-1,2-dione hydrolase [EC:3.7.1.-]	Inositol phosphate metabolism	201	1	201	2.0E-96	99%	355393429	Bacteria	Firmicutes	Bacilli	Lactobacillus rhamnosus ATCC 8530, complete genome, product = 3D-(3, 5/4)-trihydroxycyclohexane-1, 2-dione hydrolase
GKJWQY101BLMTA	K01854	UDP-galactopyranose mutase [EC:5.4.99.9]	Intramolecular transferases	326	1	326	1.0E-85	85%	120400324	Bacteria	Firmicutes	Bacilli	Lactobacillus johnsonii strain ATCC 33200 exopolysaccharide biosynthesis gene cluster, complete sequence, product = UDP-galactopyranose mutase
GKJWQY101BRHOO	K00677	UDP-N-acetylglucosamine acyltransferase [EC:2.3.1.129]	Lipopolysaccharide biosynthesis	293	1	293	3.0E-130	96%	428238862	Bacteria	Cyanobacteria	n	Oscillatoria nigro-viridis PCC 7112, complete genome, product = acyl-(acyl-carrier-protein)--UDP-N-acetylglucosamine O-acyltransferase
GKJWQY101B1OUF	K00748	lipid-A-disaccharide synthase [EC:2.4.1.182]	Lipopolysaccharide biosynthesis	413	1	413	0.0E+00	96%	428238862	Bacteria	Cyanobacteria	n	Oscillatoria nigro-viridis PCC 7112, complete genome, product = putative lipid-A-disaccharide synthase
GKJWQY101AZRJ4	K02535	UDP-3-O-[3-hydroxymyristoyl] N-acetylglucosamine deacetylase [EC:3.5.1.-]	Lipopolysaccharide biosynthesis	498	1	498	8.0E-138	85%	407894523	Bacteria	Proteobacteria	Betaproteobacteria	Acidovorax sp. KKS102, complete genome, product = UDP-3-O-[3-hydroxymyristoyl] N-acetylglucosamine deacetylase
GKJWQY101AUUBE	K02517	lipid A biosynthesis lauroyl acyltransferase [EC:2.3.1.-]	Lipopolysaccharide biosynthesis	312	1	312	6.0E-128	94%	387575654	Bacteria	Proteobacteria	Betaproteobacteria	Burkholderia sp. KJ006 chromosome 1, complete sequence, product = lipid A biosynthesis lauroyl acyltransferase
GKJWQY101A7HV4	K01929	UDP-N-acetylmuramoylalanyl-D-glutamyl-2,6-diaminopimelate--D-alanyl-D-alanine ligase [EC:6.3.2.10]	Lysine biosynthesis/Peptidoglycan biosynthesis	480	1	480	0.0E+00	99%	355393429	Bacteria	Firmicutes	Bacilli	Lactobacillus rhamnosus ATCC 8530, complete genome, product = UDP-N-acetylmuramoyl-tripeptide--D-alanyl-D-alanine ligase family protein
GKJWQY101BMITR	K01929	UDP-N-acetylmuramoylalanyl-D-glutamyl-2,6-diaminopimelate--D-alanyl-D-alanine ligase [EC:6.3.2.10]	Lysine biosynthesis/Peptidoglycan biosynthesis	479	1	479	0.0E+00	99%	355393429	Bacteria	Firmicutes	Bacilli	Lactobacillus rhamnosus ATCC 8530, complete genome, product = UDP-N-acetylmuramoyl-tripeptide--D-alanyl-D-alanine ligase family protein
GKJWQY101BHYEF	K01423	[EC:3.4.-.-]	Lysine degradation/Biotin metabolism	314	1	314	2.0E-136	95%	428238862	Bacteria	Cyanobacteria	n	Oscillatoria nigro-viridis PCC 7112, complete genome, product = processing peptidase
GKJWQY101AJPHZ	K08223	MFS transporter, FSR family, fosmidomycin resistance protein	Major Facilitator Superfamily (MFS) Drug transporters	410	1	410	5.0E-50	76%	119372524	Bacteria	Proteobacteria	Alphaproteobacteria	Paracoccus denitrificans PD1222 chromosome 1, complete sequence, product = major facilitator superfamily MFS_1
GKJWQY101BW6ZG	K01070	S-formylglutathione hydrolase [EC:3.1.2.12]	Methane metabolism	237	1	237	4.0E-98	95%	428238862	Bacteria	Cyanobacteria	n	Oscillatoria nigro-viridis PCC 7112, complete genome, product = S-formylglutathione hydrolase
GKJWQY101A44JV	K00598	trans-aconitate 2-methyltransferase [EC:2.1.1.144]	Methyltransferases [Transferring one-carbon groups]	477	1	477	0.0E+00	99%	187427012	Bacteria	Proteobacteria	Gammaproteobacteria	Shigella boydii CDC 3083-94, complete genome, product = trans-aconitate 2-methyltransferase
GKJWQY101AIZ3R	K03572	DNA mismatch repair protein MutL	Mismatch repair	490	1	490	0.0E+00	93%	428238862	Bacteria	Cyanobacteria	n	Oscillatoria nigro-viridis PCC 7112, complete genome, product = DNA mismatch repair protein MutL
GKJWQY101BYVK9	K03601	exodeoxyribonuclease VII large subunit [EC:3.1.11.6]	Mismatch repair	511	1	511	0.0E+00	96%	428238862	Bacteria	Cyanobacteria	n	Oscillatoria nigro-viridis PCC 7112, complete genome, product = exodeoxyribonuclease VII small subunit (<1..20) & VII large subunit(17..>511)
GKJWQY101A5EC7	K07496	putative transposase	n/a	517	1	517	0.0E+00	96%	428238862	Bacteria	Cyanobacteria	n	Oscillatoria nigro-viridis PCC 7112, complete genome, product = transposase, IS605 OrfB family
GKJWQY101AF275	K06929	Predicted CoA-binding protein	n/a	339	1	339	2.0E-172	99%	116106497	Bacteria	Firmicutes	Bacilli	Lactococcus lactis subsp. cremoris SK11, complete genome, product = predicted CoA-binding protein
GKJWQY101BOJM6	K07492	putative transposase	n/a	227	1	227	1.0E-112	100%	407894523	Bacteria	Proteobacteria	Betaproteobacteria	Acidovorax sp. KKS102, complete genome, product = IS4 family transposase (<1..213); hypothetical protein (210..>227)
GKJWQY101ASQTP	K00721	dolichol-phosphate mannosyltransferase [EC:2.4.1.83]	N-Glycan biosynthesis	418	1	418	0.0E+00	96%	428238862	Bacteria	Cyanobacteria	n	Oscillatoria nigro-viridis PCC 7112, complete genome, product = glycosyl transferase family 2
GKJWQY101AV5PN	K01950	NAD+ synthase (glutamine-hydrolysing) [EC:6.3.5.1]	Nicotinate and nicotinamide metabolism	235	1	235	2.0E-115	99%	430780086	Bacteria	Spirochaetes	Spirochaetes	Brachyspira pilosicoli P43/6/78, complete genome, product = NAD+ synthetase
GKJWQY101AIJQR	K00367	ferredoxin-nitrate reductase [EC:1.7.7.2]	Nitrogen metabolism	430	1	430	0.0E+00	97%	428238862	Bacteria	Cyanobacteria	n	Oscillatoria nigro-viridis PCC 7112, complete genome, product = assimilatory nitrate reductase (ferredoxin) precursor
GKJWQY101ACCFZ	K01953	asparagine synthase (glutamine-hydrolysing) [EC:6.3.5.4]	Nitrogen metabolism/Alanine, aspartate and glutamate metabolism	515	1	515	0.0E+00	99%	103422338	Bacteria	Firmicutes	Bacilli	Lactobacillus delbrueckii subsp. bulgaricus ATCC 11842 complete genome, product = asparagine synthase
GKJWQY101BN57R	K02050	sulfonate/nitrate/taurine transport system permease protein	NitT/TauT family transport system	458	1	458	6.0E-114	83%	469772332	Bacteria	Proteobacteria	Betaproteobacteria	Ralstonia solanacearum FQY_4, complete genome, product = ABC-type nitrate/sulfonate/bicarbonate transport system, ATPase component (<1..75); Alkanesulfonates transport system permease protein (99..>458)
GKJWQY101BFZXQ	K03723	transcription-repair coupling factor (superfamily II helicase) [EC:3.6.4.-]	Nucleotide excision repair	476	1	476	1.0E-100	81%	295083795	Bacteria	Bacteroidetes	Bacteroidia	Bacteroides xylanisolvens XB1A draft genome, product = genomic DNA
GKJWQY101AEXUL	K03703	excinuclease ABC subunit C	Nucleotide excision repair	461	1	461	1.0E-131	86%	336024847	Bacteria	Proteobacteria	Alphaproteobacteria	Mesorhizobium opportunistum WSM2075, complete genome, product = excinuclease ABC, C subunit
GKJWQY101AJ43P	K03702	excinuclease ABC subunit B	Nucleotide excision repair	476	1	476	0.0E+00	93%	133737197	Bacteria	Proteobacteria	Betaproteobacteria	Herminiimonas arsenicoxydans chromosome, complete sequence, product = UvrABC system protein B (Protein uvrB) (Excinuclease ABC subunit B)
GKJWQY101ACX1U	K02335	DNA polymerase I [EC:2.7.7.7]	Nucleotide excision repair/Base excision repair/Purine metabolism/Pyrimidine metabolism/DNA replication/Mismatch repair/Homologous recombination	421	1	421	0.0E+00	96%	325332286	Bacteria	Firmicutes	Bacilli	Lactobacillus acidophilus 30SC, complete genome, product = DNA polymerase I
GKJWQY101AHB7R	K03657	DNA helicase II / ATP-dependent DNA helicase PcrA [EC:3.6.4.12]	Nucleotide excision repair/Mismatch repair	533	1	533	0.0E+00	94%	428238862	Bacteria	Cyanobacteria	n	Oscillatoria nigro-viridis PCC 7112, complete genome, product = ATP-dependent DNA helicase PcrA
GKJWQY101A0KMK	K00986	RNA-directed DNA polymerase [EC:2.7.7.49]	Nucleotidyltransferases	516	1	516	0.0E+00	91%	428244862	Bacteria	Cyanobacteria	n	Oscillatoria nigro-viridis PCC 7112 plasmid pOSC7112.02, complete sequence, product = RNA-directed DNA polymerase (Reverse transcriptase)

GKJWQY101BNDE7	K13403	methylenetetrahydrofolate dehydrogenase(NAD+) / 5,10-methenyltetrahydrofolate cyclohydrolase [EC:3.5.4.9 1.5.1.15]	One carbon pool by folate	532	1	532	0.0E+00	94%	428238862	Bacteria	Cyanobacteria	n	Oscillatoria nigro-viridis PCC 7112, complete genome, product = bifunctional protein folD (<1..293); hypothetical protein (370..>535)
GKJWQY101AO712	K08045	adenylate cyclase 5 [EC:4.6.1.1]	Oocyte meiosis	244	1	244	1.0E-92	92%	428238862	Bacteria	Cyanobacteria	n	Oscillatoria nigro-viridis PCC 7112, complete genome, product = adenylate/guanylate cyclase with GAF and PAS/PAC sensors
GKJWQY101BVTHW	K01507	inorganic pyrophosphatase [EC:3.6.1.1]	Oxidative phosphorylation	234	1	234	1.0E-97	95%	428238862	Bacteria	Cyanobacteria	n	Oscillatoria nigro-viridis PCC 7112, complete genome, product = inorganic pyrophosphatase
GKJWQY101A6GJD	K01535	H+-transporting ATPase [EC:3.6.3.6]	Oxidative phosphorylation	290	1	290	6.0E-132	97%	428238862	Bacteria	Cyanobacteria	n	Oscillatoria nigro-viridis PCC 7112, complete genome, product = ATPase, P-type (transporting), HAD superfamily, subfamily IC
GKJWQY101A2N5U	K05577	NADH dehydrogenase I subunit 5 [EC:1.6.5.3]	Oxidative phosphorylation	251	1	251	1.0E-108	96%	428238862	Bacteria	Cyanobacteria	n	Oscillatoria nigro-viridis PCC 7112, complete genome, product = NADH dehydrogenase subunit L
GKJWQY101BK6YD	K03885	NADH dehydrogenase [EC:1.6.99.3]	Oxidative phosphorylation	330	1	330	1.0E-169	99%	133737197	Bacteria	Proteobacteria	Betaproteobacteria	Herminiimonas arsenicoxydans chromosome, complete sequence, product = NADH dehydrogenase
GKJWQY101B1IG8	K02298	cytochrome o ubiquinol oxidase subunit I [EC:1.10.3.-]	Oxidative phosphorylation	465	1	465	4.0E-175	91%	30407127	Bacteria	Proteobacteria	Betaproteobacteria	Ralstonia solanacearum GMI1000 chromosome complete sequence, product = probable transmembrane cytochrome o ubiquinol oxidase (subunitI oxidoreductase protein
GKJWQY101APEM7	K00342	NADH dehydrogenase I subunit M	Oxidative phosphorylation/Nitrogen metabolism	476	1	476	0.0E+00	92%	428238862	Bacteria	Cyanobacteria	n	Oscillatoria nigro-viridis PCC 7112, complete genome, product = proton-translocating NADH-quinone oxidoreductase, chain M
GKJWQY101BAGJL	K00334	NADH dehydrogenase I subunit E [EC:1.6.5.3]	Oxidative phosphorylation/Nitrogen metabolism	447	1	447	0.0E+00	98%	387575654	Bacteria	Proteobacteria	Betaproteobacteria	Burkholderia sp. KJ006 chromosome 1, complete sequence, product = NADH-ubiquinone oxidoreductase chain E
GKJWQY101BREYN	K00412	ubiquinol-cytochrome c reductase cytochrome b subunit [EC:1.10.2.2]	Oxidative phosphorylation/Nitrogen metabolism/Two-component system	509	1	509	0.0E+00	98%	77965403	Bacteria	Proteobacteria	Betaproteobacteria	Burkholderia sp. 383 chromosome 1, complete sequence, product = cytochrome b/b6-like protein(<1..392); cytochrome c1 (414..>509)
GKJWQY101BY1LH	K02115	F-type H+-transporting ATPase subunit gamma [EC:3.6.3.14]	Oxidative phosphorylation/Photosynthesis	465	1	465	0.0E+00	96%	428238862	Bacteria	Cyanobacteria	n	Oscillatoria nigro-viridis PCC 7112, complete genome, product = ATP synthase F1 subcomplex gamma subunit
GKJWQY101B2HJ8	K00425	cytochrome bd-I oxidase subunit I [EC:1.10.3.-]	Oxidative phosphorylation/Two-component system	471	1	471	0.0E+00	99%	387575654	Bacteria	Proteobacteria	Betaproteobacteria	Burkholderia sp. KJ006 chromosome 1, complete sequence, product = cytochrome d ubiquinol oxidase subunit I (<1..399) and subunit II (431..>471)
GKJWQY101BPM80	K00425	cytochrome bd-I oxidase subunit I [EC:1.10.3.-]	Oxidative phosphorylation/Two-component system	471	1	471	0.0E+00	99%	387575654	Bacteria	Proteobacteria	Betaproteobacteria	Burkholderia sp. KJ006 chromosome 1, complete sequence, product = cytochrome d ubiquinol oxidase subunit I (<1..397); cytochrome d ubiquinol oxidase subunit II (429..>469)
GKJWQY101BIGXG	K00184	molybdopterin oxidoreductase, iron-sulfur binding subunit [EC:1.2.7.-]	Oxidoreductases [iron-sulfur protein as acceptor]	357	1	357	1.0E-89	84%	255342900	Bacteria	Bacteroidetes	Sphingobacteriia	Pedobacter heparinus DSM 2366, complete genome, product = putative iron-sulfur binding oxidoreductase
GKJWQY101BIWTP	K00359	NADH oxidase [EC:1.6.-.-]	Oxidoreductases/Acting on NADH or NADPH	525	1	525	0.0E+00	99%	355393429	Bacteria	Firmicutes	Bacilli	Lactobacillus rhamnosus ATCC 8530, complete genome, product = NADH peroxidase
GKJWQY101BRRK3	K01118	FMN-dependent NADH-azoreductase [EC:1.7.-.-]	Oxidoreductases[Acting on nitrogenous compounds as donors]	503	1	503	6.0E-159	87%	299075079	Bacteria	Proteobacteria	Betaproteobacteria	Ralstonia solanacearum PSI07 megaplasmid mpPSI07, complete sequence, product = FMN-dependent NADH-azoreductase (FMN-dependent NADH-azo compound oxidoreductase) (Azo-dye reductase)
GKJWQY101BYRYA	K01918	pantoate--beta-alanine ligase [EC:6.3.2.1]	Pantothenate and CoA biosynthesis/beta-Alanine metabolism	412	1	412	8.0E-162	92%	428238862	Bacteria	Cyanobacteria	n	Oscillatoria nigro-viridis PCC 7112, complete genome, product = pantothenate synthetase
GKJWQY101AQW1B	K01467	beta-lactamase [EC:3.5.2.6]	Penicillin and cephalosporin biosynthesis/beta-Lactam resistance/Two-component system	448	1	448	0.0E+00	99%	407955691	Bacteria	Firmicutes	Bacilli	Bacillus subtilis BEST7613 DNA, complete genome, product = beta-lactamase
GKJWQY101BOVOO	K00012	UDPglucose 6-dehydrogenase [EC:1.1.1.22]	Pentose and glucuronate interconversions/Ascorbate and aldarate metabolism/Amino sugar and nucleotide sugar metabolism/ Starch and sucrose metabolism	290	12	290	5.0E-63	83%	363404872	Bacteria	Proteobacteria	Alphaproteobacteria	Brucella canis HSK A52141 chromosome 2, complete sequence, product = UDP glucose 6-dehydrogenase
GKJWQY101BH0SD	K00616	transaldolase [EC:2.2.1.2]	Pentose phosphate pathway	322	1	322	2.0E-141	95%	428238862	Bacteria	Cyanobacteria	n	Oscillatoria nigro-viridis PCC 7112, complete genome, product = transaldolase
GKJWQY101AV9UD	K00874	2-dehydro-3-deoxygluconokinase [EC:2.7.1.45]	Pentose phosphate pathway/Pentose and glucuronate interconversions	496	1	496	0.0E+00	99%	355393429	Bacteria	Firmicutes	Bacilli	Lactobacillus rhamnosus ATCC 8530, complete genome, product = 2-keto-3-deoxygluconate kinase
GKJWQY101AOSDF	K00948	ribose-phosphate pyrophosphokinase [EC:2.7.6.1]	Pentose phosphate pathway/Purine metabolism	396	1	396	0.0E+00	99%	355393429	Bacteria	Firmicutes	Bacilli	Lactobacillus rhamnosus ATCC 8530, complete genome, product = ribose-phosphate pyrophosphokinase
GKJWQY101A52UG	K03587	cell division protein FtsI (penicillin-binding protein 3) [EC:2.4.1.129]	Peptidoglycan biosynthesis	391	1	391	2.0E-134	89%	428238862	Bacteria	Cyanobacteria	n	Oscillatoria nigro-viridis PCC 7112, complete genome, product = penicillin-binding protein transpeptidase
GKJWQY101AIWNW	K03693	penicillin-binding protein	Peptidoglycan biosynthesis	414	1	414	0.0E+00	99%	355393429	Bacteria	Firmicutes	Bacilli	Lactobacillus rhamnosus ATCC 8530, complete genome, product = transglycosylase family protein
GKJWQY101AUBWF	K06153	undecaprenyl-diphosphatase [EC:3.6.1.27]	Peptidoglycan biosynthesis	469	1	469	0.0E+00	98%	257048753	Bacteria	Fusobacteria	Fusobacteriales	Leptotrichia buccalis DSM 1135, complete genome, product = undecaprenol kinase (PFAM: Bacitracin resistance protein BacA)
GKJWQY101AYAGY	K05712	3-(3-hydroxy-phenyl)propionate hydroxylase [EC:1.14.13.-]	Phenylalanine metabolism	476	1	476	0.0E+00	96%	387578572	Bacteria	Proteobacteria	Betaproteobacteria	Burkholderia sp. KJ006 chromosome 2, complete sequence, product = 3-(3-hydroxy-phenyl)propionate hydroxylase
GKJWQY101BTCIC	K01451	hippurate hydrolase [EC:3.5.1.32]	Phenylalanine metabolism	479	1	479	6.0E-154	87%	299076774	Bacteria	Proteobacteria	Betaproteobacteria	Ralstonia solanacearum str. PSI07 chromosome, complete genome, product = putative Hippurate hydrolase (hipO)
GKJWQY101A7AXB	K01735	3-dehydroquinate synthase [EC:4.2.3.4]	Phenylalanine, tyrosine and tryptophan biosynthesis	501	1	501	0.0E+00	96%	428238862	Bacteria	Cyanobacteria	n	Oscillatoria nigro-viridis PCC 7112, complete genome, product = 3-dehydroquinate synthase
GKJWQY101AP61F	K01090	protein phosphatase [EC:3.1.3.16]	Phosphoric-monoester hydrolases [serine-threonine]	417	1	417	0.0E+00	99%	312279338	Bacteria	Firmicutes	Bacilli	Lactobacillus delbrueckii subsp. bulgaricus ND02, complete genome, product = Serine/threonine protein phosphatase Stp1
GKJWQY101A6VDP	K02794	PTS system, mannose-specific IIB component [EC:2.7.1.69]	Phosphotransferase system (PTS)/Fructose and mannose metabolism/Amino sugar and nucleotide sugar metabolism	253	1	253	3.0E-119	98%	335369081	Bacteria	Firmicutes	Bacilli	Streptococcus parasanguinis ATCC 15912, complete genome, product = PTS system, mannose-specific IIB component
GKJWQY101BWBP4	K02786	PTS system, lactose-specific IIA component [EC:2.7.1.69]	Phosphotransferase system (PTS)/Galactose metabolism	472	1	472	0.0E+00	100%	355393429	Bacteria	Firmicutes	Bacilli	Lactobacillus rhamnosus ATCC 8530, complete genome, product = lactose-specific phosphotransferase enzyme IIA component
GKJWQY101BMIJH	K02787	PTS system, lactose-specific IIB component [EC:2.7.1.69]	Phosphotransferase system (PTS)/Galactose metabolism	315	1	315	5.0E-163	100%	406356677	Bacteria	Firmicutes	Bacilli	Lactobacillus casei W56 complete genome, product = PTS system lactose-specific EIICB component
GKJWQY101BVTHW_2	T02375	S-layer domain-containing protein	Photosynthesis	180	1	180	2.0E-74	96%	428238862	Bacteria	Cyanobacteria	n	Oscillatoria nigro-viridis PCC 7112, complete genome, product = S-layer domain-containing protein
GKJWQY101BQ2XO	K02634	apocytochrome f	Photosynthesis	506	1	506	0.0E+00	96%	428238862	Bacteria	Cyanobacteria	n	Oscillatoria nigro-viridis PCC 7112, complete genome, product = apocytochrome f
GKJWQY101BIZEW	K02689	photosystem I P700 chlorophyll a apoprotein A1	Photosynthesis	543	1	543	0.0E+00	96%	428238862	Bacteria	Cyanobacteria	n	Oscillatoria nigro-viridis PCC 7112, complete genome, product = photosystem I P700 chlorophyll a apoprotein A2 (<1..244); photosystem I P700 chlorophyll a apoprotein A1 (355..>541)

GKJWQY101AWFUY	K02690	photosystem I P700 chlorophyll a apoprotein A2	Photosynthesis	429	1	429	0.0E+00	97%	428238862	Bacteria	Cyanobacteria	n	Oscillatoria nigro-viridis PCC 7112, complete genome, product = photosystem I P700 chlorophyll a apoprotein A2
GKJWQY101BJE6N	K02699	photosystem I subunit XI	Photosynthesis	504	1	504	0.0E+00	95%	428238862	Bacteria	Cyanobacteria	n	Oscillatoria nigro-viridis PCC 7112, complete genome, product = photosystem I reaction centre subunit XI PsaL (<1..262); glycosyl transferase family 2 (376..>506)
GKJWQY101A5ZXB	K02703	photosystem II P680 reaction center D1 protein	Photosynthesis	336	1	336	7.0E-152	96%	428238862	Bacteria	Cyanobacteria	n	Oscillatoria nigro-viridis PCC 7112, complete genome, product = photosystem Q(B) protein (photosystem II PsbA/D1, reaction centre)
GKJWQY101A0MVM	K02705	photosystem II CP43 chlorophyll apoprotein	Photosynthesis	430	1	430	0.0E+00	97%	428238862	Bacteria	Cyanobacteria	n	Oscillatoria nigro-viridis PCC 7112, complete genome, product = photosystem II 44 kDa subunit reaction center protein
GKJWQY101BY2F1	K02285	phycocyanin beta chain	Photosynthesis - antenna proteins	442	1	442	0.0E+00	98%	428238862	Bacteria	Cyanobacteria	n	Oscillatoria nigro-viridis PCC 7112, complete genome, product = Phycocyanin
GKJWQY101BDPWW	K02358	elongation factor Tu	Plant-pathogen interaction	487	1	487	3.0E-147	86%	384071898	Bacteria	Firmicutes	Bacilli	Halobacillus halophilus DSM 2266 complete genome, product = translation elongation factor Tu
GKJWQY101BVGC1	K02358	elongation factor Tu	Plant-pathogen interaction	455	1	455	2.0E-128	85%	307830710	Bacteria	Firmicutes	Bacilli	Staphylococcus kloosii strain DSM 20676 elongation factor Tu (tuf) gene, partial cds
GKJWQY101BRE7O	K01790	dTDP-4-dehydrorhamnose 3,5-epimerase [EC:5.1.3.13]	Polyketide sugar unit biosynthesis/Streptomycin biosynthesis	132	1	132	1.3E+02	79%	451908558	Bacteria	Proteobacteria	Gammaproteobacteria	Salmonella enterica subsp. enterica serovar Javiana str. CFSAN001992, complete genome, product = dTDP-4-dehydrorhamnose 3,5-epimerase
GKJWQY101ASQXM_2	K02014	mechanosensitive ion channel	Pores ion channels	93	1	93	3.0E-20	87%	428238862	Bacteria	Cyanobacteria	n	Oscillatoria nigro-viridis PCC 7112, complete genome, product = MscS Mechanosensitive ion channel
GKJWQY101BLISX	K04744	LPS-assembly protein	Pores ion channels	515	1	515	0.0E+00	99%	387575654	Bacteria	Proteobacteria	Betaproteobacteria	Burkholderia sp. KJ006 chromosome 1, complete sequence, product = outer membrane protein Imp, required for envelope biogenesis/Organic solvent tolerance protein precursor
GKJWQY101AH1BL	K01599	uroporphyrinogen decarboxylase [EC:4.1.1.37]	Porphyrin and chlorophyll metabolism	494	1	494	0.0E+00	94%	428238862	Bacteria	Cyanobacteria	n	Oscillatoria nigro-viridis PCC 7112, complete genome, product = uroporphyrinogen decarboxylase
GKJWQY101AU6TO	K01845	glutamate-1-semialdehyde 2,1-aminomutase [EC:5.4.3.8]	Porphyrin and chlorophyll metabolism	392	1	392	2.0E-153	92%	428238862	Bacteria	Cyanobacteria	n	Oscillatoria nigro-viridis PCC 7112, complete genome, product = glutamate-1-semialdehyde 2,1-aminomutase
GKJWQY101BUDW0	K03403	magnesium chelatase subunit H [EC:6.6.1.1]	Porphyrin and chlorophyll metabolism	479	1	479	0.0E+00	98%	428238862	Bacteria	Cyanobacteria	n	Oscillatoria nigro-viridis PCC 7112, complete genome, product = cobaltochelatase CobN subunit (note: CobN/Magnesium Chelatase)
GKJWQY101AB54X	K06042	precorrin-8X methylmutase [EC:5.4.1.2]	Porphyrin and chlorophyll metabolism	321	1	321	8.0E-151	97%	428238862	Bacteria	Cyanobacteria	n	Oscillatoria nigro-viridis PCC 7112, complete genome, product = precorrin-8X methylmutase
GKJWQY101BVYRA	K00228	coproporphyrinogen III oxidase [EC:1.3.3.3]	Porphyrin and chlorophyll metabolism	339	1	339	3.0E-106	88%	336024847	Bacteria	Proteobacteria	Alphaproteobacteria	Mesorhizobium opportunistum WSM2075, complete genome, product = coproporphyrinogen III oxidase
GKJWQY101AQPZB	K04037	light-independent protochlorophyllide reductase subunit L [EC:1.18.-.-]	Porphyrin and chlorophyll metabolism	376	1	376	1.0E-176	97%	428238862	Bacteria	Cyanobacteria	n	Oscillatoria nigro-viridis PCC 7112, complete genome, product = ferredoxin protochlorophyllide reductase subunit L
GKJWQY101ACHK0	K04039	light-independent protochlorophyllide reductase subunit B [EC:1.18.-.-]	Porphyrin and chlorophyll metabolism	355	1	355	2.0E-167	96%	428238862	Bacteria	Cyanobacteria	n	Oscillatoria nigro-viridis PCC 7112, complete genome, product = ferredoxin protochlorophyllide reductase subunit B
GKJWQY101ASQXM	K00510	heme oxygenase [EC:1.14.99.3]	Porphyrin and chlorophyll metabolism/Mineral absorption	263	1	263	2.0E-122	97%	428238862	Bacteria	Cyanobacteria	n	Oscillatoria nigro-viridis PCC 7112, complete genome, product = heme oxygenase
GKJWQY101BRN0I	K07250	4-aminobutyrate aminotransferase / (S)-3-amino-2-methylpropionate transaminase [EC:2.6.1.22 2.6.1.19]	Propanoate metabolism	480	1	480	3.0E-57	76%	344313278	Bacteria	Actinobacteria	Actinobacteria	Streptomyces sp. SirexAA-E, complete genome, product = 4-aminobutyrate aminotransferase
GKJWQY101APEQF	K03101	signal peptidase II [EC:3.4.23.36]	Protein export	430	1	430	0.0E+00	97%	428238862	Bacteria	Cyanobacteria	n	Oscillatoria nigro-viridis PCC 7112, complete genome, product = lipoprotein signal peptidase
GKJWQY101BUW9I	K01524	exopolyphosphatase / guanosine-5'-triphosphate,3'-diphosphate pyrophosphatase [EC:3.6.1.40 3.6.1.11]	Purine metabolism	484	1	484	0.0E+00	95%	428238862	Bacteria	Cyanobacteria	n	Oscillatoria nigro-viridis PCC 7112, complete genome, product = Ppx/GppA phosphatase
GKJWQY101BSGTQ	K01952	phosphoribosylformylglycinamidine synthase [EC:6.3.5.3]	Purine metabolism	461	1	461	0.0E+00	96%	428238862	Bacteria	Cyanobacteria	n	Oscillatoria nigro-viridis PCC 7112, complete genome, product = phosphoribosylformylglycinamidine synthase subunit II
GKJWQY101ANN8E	K00088	IMP dehydrogenase [EC:1.1.1.205]	Purine metabolism	368	1	368	0.0E+00	99%	355393429	Bacteria	Firmicutes	Bacilli	Lactobacillus rhamnosus ATCC 8530, complete genome, product = inosine-5'-monophosphate dehydrogenase
GKJWQY101ASHPM	K01139	guanosine-3',5'-bis(diphosphate) 3'-pyrophosphohydrolase [EC:3.1.7.2]	Purine metabolism	481	1	481	0.0E+00	99%	387575654	Bacteria	Proteobacteria	Betaproteobacteria	Burkholderia sp. KJ006 chromosome 1, complete sequence, product = GTP pyrophosphokinase, (p)ppGpp synthetase II / Guanosine-3',5'-bis(diphosphate) 3'-pyrophosphohydrolase
GKJWQY101BS894	K01933	phosphoribosylformylglycinamidine cyclo-ligase [EC:6.3.3.1]	Purine metabolism	355	1	355	3.0E-180	99%	387575654	Bacteria	Proteobacteria	Betaproteobacteria	Burkholderia sp. KJ006 chromosome 1, complete sequence, product = phosphoribosylformylglycinamidine cyclo-ligase
GKJWQY101BI3EE	K01951	GMP synthase (glutamine-hydrolysing) [EC:6.3.5.2]	Purine metabolism/Drug metabolism - cytochrome P450	341	1	341	1.0E-154	96%	428238862	Bacteria	Cyanobacteria	n	Oscillatoria nigro-viridis PCC 7112, complete genome, product = GMP synthase (glutamine-hydrolyzing)
GKJWQY101A7UX8	K01951	GMP synthase (glutamine-hydrolysing) [EC:6.3.5.2]	Purine metabolism/Drug metabolism - cytochrome P450	469	1	469	4.0E-96	81%	110283346	Bacteria	Proteobacteria	Alphaproteobacteria	Chelativorans sp. BNC1, complete genome, product = GMP synthase (glutamine-hydrolyzing)
GKJWQY101AF62K	K01768	adenylate cyclase [EC:4.6.1.1]	Purine metabolism/Meiosis-yeast	488	1	488	0.0E+00	93%	428238862	Bacteria	Cyanobacteria	n	Oscillatoria nigro-viridis PCC 7112, complete genome, product = adenylate/guanylate cyclase with GAF sensor(s)
GKJWQY101BHHIQ	K00525	ribonucleoside-diphosphate reductase alpha chain [EC:1.17.4.1]	Purine metabolism/Pyrimidine metabolism	484	1	484	2.0E-108	82%	78609255	Bacteria	Firmicutes	Bacilli	Lactobacillus sakei strain 23K complete genome, product = ribonucleoside-diphosphate reductase, alpha chain
GKJWQY101AQ3AJ	K02340	DNA polymerase III subunit delta [EC:2.7.7.7]	Purine metabolism/Pyrimidine metabolism/DNA replication/Mismatch repair/Homologous recombination	434	1	434	3.0E-176	93%	428238862	Bacteria	Cyanobacteria	n	Oscillatoria nigro-viridis PCC 7112, complete genome, product = DNA polymerase III, delta subunit
GKJWQY101A1FIZ	K02343	DNA polymerase III subunit gamma/tau [EC:2.7.7.7]	Purine metabolism/Pyrimidine metabolism/DNA replication/Mismatch repair/Homologous recombination	436	1	436	0.0E+00	99%	355393429	Bacteria	Firmicutes	Bacilli	Lactobacillus rhamnosus ATCC 8530, complete genome, product = DNA polymerase III, subunit gamma and tau
GKJWQY101AW79B	K00962	polyribonucleotide nucleotidyltransferase [EC:2.7.7.8]	Purine metabolism/Pyrimidine metabolism/RNA degradation	502	1	502	9.0E-123	83%	319414919	Bacteria	Bacteroidetes	Bacteroidia	Bacteroides helcogenes P 36-108, complete genome, product = polyribonucleotide nucleotidyltransferase
GKJWQY101BZRZ2	K03046	DNA-directed RNA polymerase subunit beta' [EC:2.7.7.6]	Purine metabolism/Pyrimidine metabolism/RNA polymerase	430	1	430	0.0E+00	99%	387575654	Bacteria	Proteobacteria	Betaproteobacteria	Burkholderia sp. KJ006 chromosome 1, complete sequence, product = DNA-directed RNA polymerase beta' subunit
GKJWQY101BUUVY	K00226	dihydroorotate dehydrogenase (fumarate) [EC:1.3.98.1]	Pyrimidine metabolism	433	1	433	0.0E+00	94%	428238862	Bacteria	Cyanobacteria	n	Oscillatoria nigro-viridis PCC 7112, complete genome, product = dihydroorotate oxidase A
GKJWQY101ANJN3	K00756	pyrimidine-nucleoside phosphorylase [EC:2.4.2.2]	Pyrimidine metabolism	355	1	355	5.0E-173	98%	333956887	Bacteria	Firmicutes	Bacilli	Lactobacillus kefiranofaciens ZW3, complete genome, product = pyrimidine-nucleoside phosphorylase
GKJWQY101BOOIO	K00857	thymidine kinase [EC:2.7.1.21]	Pyrimidine metabolism	352	1	352	5.0E-168	98%	355393429	Bacteria	Firmicutes	Bacilli	Lactobacillus rhamnosus ATCC 8530, complete genome, product = thymidine kinase family protein
GKJWQY101A7UM0	K01494	dCTP deaminase [EC:3.5.4.13]	Pyrimidine metabolism	444	1	444	0.0E+00	99%	387575654	Bacteria	Proteobacteria	Betaproteobacteria	Burkholderia sp. KJ006 chromosome 1, complete sequence, product = arginine decarboxylase, ornithine decarboxylase, lysine decarboxylase (<1..70); deoxycytidine triphosphate deaminase (152..>444)
GKJWQY101A9VNV	K01955	carbamoyl-phosphate synthase large subunit [EC:6.3.5.5]	Pyrimidine metabolism/Alanine, aspartate and glutamate metabolism	469	1	469	7.0E-163	89%	71037566	Bacteria	Proteobacteria	Gammaproteobacteria	Psychrobacter arcticus 273-4, complete genome, product = carbamoyl-phosphate synthase large subunit

GKJWQY101BUBHU	K01485	cytosine deaminase [EC:3.5.4.1]	Pyrimidine metabolism/Arginine and proline metabolism	483	1	483	1.0E-150	87%	428238862	Bacteria	Cyanobacteria	n	Oscillatoria nigro-viridis PCC 7112, complete genome, product = cytosine deaminase
GKJWQY101BNDRC	K00560	thymidylate synthase [EC:2.1.1.45]	Pyrimidine metabolism/One carbon pool by folate	302	1	302	5.0E-148	99%	413973243	Bacteria	Firmicutes	Bacilli	Lactococcus lactis subsp. cremoris UC509.9, complete genome, product = thymidylate synthase
GKJWQY101AH75P	K00158	pyruvate oxidase [EC:1.2.3.3]	Pyruvate metabolism	464	1	464	0.0E+00	99%	257149867	Bacteria	Firmicutes	Bacilli	Lactobacillus rhamnosus Lc 705 whole genome sequence, strain Lc705, product = pyruvate oxidase
GKJWQY101BFXEC	K00027	malate dehydrogenase (oxaloacetate-decarboxylating) [EC:1.1.1.38]	Pyruvate metabolism/ Two-component system	495	14	495	0.0E+00	99%	116103724	Bacteria	Firmicutes	Bacilli	Lactobacillus casei ATCC 334, complete genome, product = malolactic enzyme
GKJWQY101A94GH	K01759	lactoylglutathione lyase [EC:4.4.1.5]	Pyruvate metabolism/MAPK signaling pathway-yeast	289	1	289	3.0E-115	93%	428238862	Bacteria	Cyanobacteria	n	Oscillatoria nigro-viridis PCC 7112, complete genome, product = lactoylglutathione lyase
GKJWQY101AZOWP	K01007	pyruvate, water dikinase [EC:2.7.9.2]	Pyruvate metabolism/Methane metabolism/Carbon fixation pathways in prokaryotes	482	1	482	0.0E+00	99%	387575654	Bacteria	Proteobacteria	Betaproteobacteria	Burkholderia sp. KJ006 chromosome 1, complete sequence, product = phosphoenolpyruvate synthase
GKJWQY101ARU6X_2	K01977	16S ribosomal RNA gene	Ribosome	179	1	179	2.0E-79	97%	209915940	Bacteria	n	n	Uncultured bacterium clone PBM_b9 16S ribosomal RNA gene, partial sequence
GKJWQY101BO1HI_2	K01980	23S ribosomal RNA	Ribosomes	271	29	271	2.0E-117	99%	428238862	Bacteria	Cyanobacteria	n	Oscillatoria nigro-viridis PCC 7112, complete genome, product = 23S rRNA gene
GKJWQY101BVVMM_2	K01980	23S ribosomal RNA	Ribosomes	249	8	248	2.0E-120	99%	428238862	Bacteria	Cyanobacteria	n	Oscillatoria nigro-viridis PCC 7112, complete genome, product = 23S rRNA gene
GKJWQY101BV866_2	K01980	23S ribosomal RNA	Ribosomes	171	1	171	3.0E-78	98%	428238862	Bacteria	Cyanobacteria	n	Oscillatoria nigro-viridis PCC 7112, complete genome, product = 23S rRNA gene
GKJWQY101A4443_2	K01980	23S ribosomal RNA	Ribosomes	334	1	334	2.0E-152	96%	428238862	Bacteria	Cyanobacteria	n	Oscillatoria nigro-viridis PCC 7112, complete genome, product = signal peptidase I (<1..241); exonuclease V subunit alpha (303..>444)
GKJWQY101BTYHD	K03703	23S ribosomal RNA	Ribosomes	331	1	331	3.0E-96	86%	156770867	Bacteria	n	n	Uncultured bacterium clone LM0ABA5ZA11RM1 genomic sequence
GKJWQY101BK048_2	K08311	putative (di)nucleoside polyphosphate hydrolase [EC:3.6.1.-]	RNA degradation	122	1	120	5.0E-39	93%	428238862	Bacteria	Cyanobacteria	n	Oscillatoria nigro-viridis PCC 7112, complete genome, product = NUDIX hydrolase (Nucleoside Diphosphate linked to X)
GKJWQY101ABJOM	K05592	ATP-dependent RNA helicase DeaD [EC:3.6.4.13]	RNA degradation	512	1	512	0.0E+00	96%	428238862	Bacteria	Cyanobacteria	n	Oscillatoria nigro-viridis PCC 7112, complete genome, product = DEAD/DEAH box helicase domain protein
GKJWQY101BRBEO	K05592	ATP-dependent RNA helicase DeaD [EC:3.6.4.13]	RNA degradation	517	1	517	0.0E+00	96%	428238862	Bacteria	Cyanobacteria	n	Oscillatoria nigro-viridis PCC 7112, complete genome, product = DEAD/DEAH box helicase domain protein
GKJWQY101AOWCF	K03654	ATP-dependent DNA helicase RecQ [EC:3.6.4.12]	RNA degradation	413	1	413	0.0E+00	93%	312279338	Bacteria	Firmicutes	Bacilli	Lactobacillus delbrueckii subsp. bulgaricus ND02, complete genome, product = ATP-dependent DNA helicase RecQ
GKJWQY101A17VU	K03628	transcription termination factor Rho	RNA degradation	411	1	411	0.0E+00	99%	387575654	Bacteria	Proteobacteria	Betaproteobacteria	Burkholderia sp. KJ006 chromosome 1, complete sequence, product = transcription termination factor Rho
GKJWQY101AWCAE	K12598	ATP-dependent RNA helicase DOB1 [EC:3.6.4.13]	RNA degradation [Proteasome]	411	1	411	0.0E+00	97%	428238862	Bacteria	Cyanobacteria	n	Oscillatoria nigro-viridis PCC 7112, complete genome, product = DSH domain protein (superfamily II RNA helicase)
GKJWQY101A7U37	K12614	ATP-dependent RNA helicase DDX6/DHH1 [EC:3.6.4.13]	RNA degradation [Proteasome]	475	1	475	0.0E+00	99%	428238862	Bacteria	Cyanobacteria	n	Oscillatoria nigro-viridis PCC 7112, complete genome, product = DEAD/DEAH box helicase domain protein
GKJWQY101BSNY0	K03231	elongation factor 1-alpha	RNA transport	510	1	510	0.0E+00	91%	71483398	Eukaryota	Basidiomycota	Microbotryomycetes	Microbotryum violaceum isolate Dsylvestris_9119 elongation factor 1alpha (EF1) gene, partial cds
GKJWQY101ASUL5	K00690	sucrose phosphorylase [EC:2.4.1.7]	Starch and sucrose metabolism	405	1	405	0.0E+00	96%	428238862	Bacteria	Cyanobacteria	n	Oscillatoria nigro-viridis PCC 7112, complete genome, product = trehalose synthase
GKJWQY101AFXGY	K05343	maltose alpha-D-glucosyltransferase [EC:5.4.99.16]	Starch and sucrose metabolism	394	1	394	0.0E+00	98%	428238862	Bacteria	Cyanobacteria	n	Oscillatoria nigro-viridis PCC 7112, complete genome, product = trehalose synthase (PRIAM: Maltose alpha-D-glucosyltransferase)
GKJWQY101BPKKT	K00703	starch synthase [EC:2.4.1.21]	Starch and sucrose metabolism	495	1	495	0.0E+00	95%	326682110	Bacteria	Firmicutes	Bacilli	Streptococcus oralis Uo5 complete genome sequence, product = glycogen synthase
GKJWQY101BG8L6	K01710	dTDP-glucose 4,6-dehydratase [EC:4.2.1.46]	Streptomycin biosynthesis/Polyketide sugar unit biosynthesis/Biosynthesis of vancomycin group antibiotics	502	1	502	3.0E-172	89%	428238862	Bacteria	Cyanobacteria	n	Oscillatoria nigro-viridis PCC 7112, complete genome, product = UDP-glucuronate decarboxylase (<1..263); nucleotide sugar dehydrogenase (384..>487)
GKJWQY101AFENF	K00381	sulfite reductase (NADPH) hemoprotein beta component [EC:1.8.1.2]	Sulfur metabolism	417	1	417	0.0E+00	99%	387575654	Bacteria	Proteobacteria	Betaproteobacteria	Burkholderia sp. KJ006 chromosome 1, complete sequence, product = Sulfite reductase [NADPH] hemoprotein beta-component
GKJWQY101BCE0I	K01738	cysteine synthase A [EC:2.5.1.47]	Sulfur metabolism/Cysteine and methionine metabolism	518	1	518	0.0E+00	99%	257152781	Bacteria	Firmicutes	Bacilli	Lactobacillus rhamnosus Lc 705 plasmid sequence, strain Lc 705, product = cystathionine beta-synthase
GKJWQY101BPUIN	K01738	cysteine synthase A [EC:2.5.1.47]	Sulfur metabolism/Cysteine and methionine metabolism	525	1	525	0.0E+00	99%	257152781	Bacteria	Firmicutes	Bacilli	Lactobacillus rhamnosus Lc 705 plasmid sequence, strain Lc 705
GKJWQY101BWI6R	K00566	tRNA-specific 2-thiouridylase [EC:2.8.1.-]	Sulfur relay system	526	1	526	0.0E+00	95%	428238862	Bacteria	Cyanobacteria	n	Oscillatoria nigro-viridis PCC 7112, complete genome, product = tRNA (5-methylaminomethyl-2-thiouridylate)-methyltransferase
GKJWQY101ACULM	K03639	molybdenum cofactor biosynthesis protein	Sulfur relay system; Folate biosynthesis	546	1	546	0.0E+00	96%	428238862	Bacteria	Cyanobacteria	n	Oscillatoria nigro-viridis PCC 7112, complete genome, product = cyclic pyranopterin monophosphate synthase subunit MoaA
GKJWQY101BFZWI	K00806	undecaprenyl diphosphate synthase [EC:2.5.1.31]	Terpenoid backbone biosynthesis	493	1	493	0.0E+00	98%	428238862	Bacteria	Cyanobacteria	n	Oscillatoria nigro-viridis PCC 7112, complete genome, product = undecaprenyl pyrophosphate synthetase
GKJWQY101BZA8P	K00805	heptaprenyl diphosphate synthase [EC:2.5.1.30]	Terpenoid backbone biosynthesis	256	1	256	2.0E-130	100%	355393429	Bacteria	Firmicutes	Bacilli	Lactobacillus rhamnosus ATCC 8530, complete genome, product = polyprenyl synthetase family protein
GKJWQY101BCEGP	K00919	4-diphosphocytidyl-2-C-methyl-D-erythritol kinase [EC:2.7.1.148]	Terpenoid backbone biosynthesis	408	1	408	0.0E+00	99%	387575654	Bacteria	Proteobacteria	Betaproteobacteria	Burkholderia sp. KJ006 chromosome 1, complete sequence, product = 4-diphosphocytidyl-2-C-methyl-D-erythritol kinase
GKJWQY101AKUTV	K03527	4-hydroxy-3-methylbut-2-enyl diphosphate reductase [EC:1.17.1.2]	Terpenoid backbone biosynthesis	478	1	478	0.0E+00	99%	387575654	Bacteria	Proteobacteria	Betaproteobacteria	Burkholderia sp. KJ006 chromosome 1, complete sequence, product = 4-hydroxy-3-methylbut-2-enyl diphosphate reductase (<1..263); FKBP-type peptidyl-prolyl cis-trans isomerase slpA (266..>479)
GKJWQY101ARLFL	K00788	thiamine-phosphate pyrophosphorylase [EC:2.5.1.3]	Thiamine metabolism	450	1	450	2.0E-178	92%	428238862	Bacteria	Cyanobacteria	n	Oscillatoria nigro-viridis PCC 7112, complete genome, product = thiamine-phosphate diphosphorylase
GKJWQY101BCW7I	K03147	thiamine biosynthesis protein ThiC	Thiamine metabolism	406	1	406	0.0E+00	95%	428238862	Bacteria	Cyanobacteria	n	Oscillatoria nigro-viridis PCC 7112, complete genome, product = hydroxymethylpyrimidine synthase (ThiC family)
GKJWQY101ASDW0	K00897	aminoglycoside 3'-phosphotransferase [EC:2.7.1.95]	Transferases [Phosphotransferases/ acceptor - alcohol group]	456	1	456	0.0E+00	99%	392495344	Bacteria	Bacteroidetes	Flavobacteriia	Riemerella anatipestifer strain W9 aminoglycoside-3'-O-phosphotransferase (aph) gene, partial cds
GKJWQY101BVJ36	K00936	E2.7.3.-	Transferases[Phosphotransferases/ acceptor - nitrogenous group]	433	1	433	4.0E-180	93%	428238862	Bacteria	Cyanobacteria	n	Oscillatoria nigro-viridis PCC 7112, complete genome, product = PAS/PAC sensor signal transduction histidine kinase
GKJWQY101AKNUG	K03234	elongation factor 2	Translation factors	480	1	480	1.0E-101	81%	67540319	Eukaryota	Ascomycota	Eurotiomycetes	Aspergillus nidulans FGSC A4 elongation factor 2 partial mRNA
GKJWQY101AH1E9	K13628	Fe-S cluster assembly protein	tRNA modification factors	332	1	332	2.0E-83	85%	260644157	Bacteria	Actinobacteria	Actinobacteria	Streptomyces scabiei 87.22 complete genome, product = putative iron-sulphur cluster biosynthesis protein
GKJWQY101AU6TO_2	K07769	Cyan7425_1370 Signal transduction histidine kinase	Two-component system	143	1	143	1.0E-21	82%	428238862	Bacteria	Cyanobacteria	n	Oscillatoria nigro-viridis PCC 7112, complete genome, product = 844 bp at 5' side: putative GAF sensor protein, 178 bp at 3' side: phosphoesterase RecJ domain protein
GKJWQY101BN289	K07778	two-component system, NarL family, sensor histidine kinase DesK [EC:2.7.13.3]	Two-component system	470	1	470	0.0E+00	93%	339291081	Bacteria	Firmicutes	Bacilli	Streptococcus salivarius 57.I, complete genome, product = signal transduction histidine kinase

GKJWQY101AG52Q	K07709	two-component system, NtrC family, sensor histidine kinase HydH [EC:2.7.13.3]	Two-component system	208	1	280	1.0E-68	90%	469772332	Bacteria	Proteobacteria	Betaproteobacteria	Ralstonia solanacearum FQY_4, complete genome, product = signal transduction histidine kinase regulating C4-dicarboxylate transport system"
GKJWQY101AX6T2	K00990	[protein-PII] uridylyltransferase [EC:2.7.7.59]	Two-component system	366	1	366	8.0E-73	81%	190694918	Bacteria	Proteobacteria	Alphaproteobacteria	Rhizobium etli CIAT 652, complete genome, product = [protein-PII] uridylyltransferase protein
GKJWQY101AHQ0Q	K13924	two-component system, chemotaxis family, CheB/CheR fusion protein [EC:3.1.1.61 2.1.1.80]	Two-component system	197	1	197	5.0E-71	92%	428238862	Bacteria	Cyanobacteria	n	Oscillatoria nigro-viridis PCC 7112, complete genome, product = signal transduction histidine kinase with CheB and CheR activity
GKJWQY101A0TE3	K02488	two-component system, cell cycle response regulator	Two-component system/Cell cycle - Caulobacter	447	1	447	0.0E+00	95%	428238862	Bacteria	Cyanobacteria	n	Oscillatoria nigro-viridis PCC 7112, complete genome, product = response regulator receiver modulated diguanylate cyclase
GKJWQY101BDHFA	K02313	chromosomal replication initiator protein	Two-component system/Cell cycle - Caulobacter	246	1	246	9.0E-125	100%	257149867	Bacteria	Firmicutes	Bacilli	Lactobacillus rhamnosus Lc 705 whole genome sequence, strain Lc 705 product = chromosomal replication initiator protein dnaA
GKJWQY101BVI18	K03407	two-component system, chemotaxis family, sensor kinase CheA [EC:2.7.13.3]	Two-component system; Bacterial chemotaxis	511	1	511	0.0E+00	95%	428238862	Bacteria	Cyanobacteria	n	Oscillatoria nigro-viridis PCC 7112, complete genome, product = CheA signal transduction histidine kinase
GKJWQY101A6MA3	K00680	[EC:2.3.1.-]	Tyrosine metabolism/Benzoate degradation/Aminobenzoate degradation/Ethylbenzene degradation/Limonene and pinene degradation	522	3	522	1.0E-111	81%	78609255	Bacteria	Firmicutes	Bacilli	Lactobacillus sakei strain 23K complete genome, product = putative N-Acetyltransferase, GNAT family (<1..420); hypothetical protein (483..>521)
GKJWQY101ADM9M	K02551	2-succinyl-5-enolpyruvyl-6-hydroxy-3-cyclohexene-1-carboxylate synthase [EC:2.2.1.9]	Ubiquinone and other terpenoid-quinone biosynthesis	346	1	346	6.0E-143	93%	428238862	Bacteria	Cyanobacteria	n	Oscillatoria nigro-viridis PCC 7112, complete genome, product = 2-succinyl-5-enolpyruvyl-6-hydroxy-3-cyclohexene-1-carboxylatesynthase
GKJWQY101AAEO3	K03183	ubiquinone/menaquinone biosynthesis methyltransferase [EC:2.1.1.- 2.1.1.163]	Ubiquinone and other terpenoid-quinone biosynthesis	402	1	402	9.0E-172	94%	428238862	Bacteria	Cyanobacteria	n	Oscillatoria nigro-viridis PCC 7112, complete genome, product = demethylmenaquinone methyltransferase
GKJWQY101AOVNZ	K00053	ketol-acid reductoisomerase [EC:1.1.1.86]	Valine, leucine and isoleucine biosynthesis/ Pantothenate and CoA biosynthesis	326	13	312	7.0E-67	83%	323467537	Bacteria	Actinobacteria	Actinobacteria	Arthrobacter phenanthrenivorans Sphe3, complete genome, product = ketol-acid reductoisomerase
GKJWQY101BVJE8	K00053	ketol-acid reductoisomerase [EC:1.1.1.86]	Valine, leucine and isoleucine biosynthesis/ Pantothenate and CoA biosynthesis	301	1	301	7.0E-67	83%	323467537	Bacteria	Actinobacteria	Actinobacteria	Arthrobacter phenanthrenivorans Sphe3, complete genome, product = ketol-acid reductoisomerase
GKJWQY101ASA5O	K00053	ketol-acid reductoisomerase [EC:1.1.1.86]	Valine, leucine and isoleucine biosynthesis/ Pantothenate and CoA biosynthesis	214	1	211	2.0E-25	78%	29342101	Bacteria	Bacteroidetes	Bacteroidia	Bacteroides thetaiotaomicron VPI-5482, complete genome, product = ketol-acid reductoisomerase
GKJWQY101AJPN3	K01652	acetolactate synthase I/II/III large subunit [EC:2.2.1.6]	Valine, leucine and isoleucine biosynthesis/Butanoate metabolism/C5-Branched dibasic acid metabolism/Pantothenate and CoA biosynthesis	498	1	498	0.0E+00	94%	428238862	Bacteria	Cyanobacteria	n	Oscillatoria nigro-viridis PCC 7112, complete genome, product = acetolactate synthase
GKJWQY101BF7DE	K01703	3-isopropylmalate/(R)-2-methylmalate dehydratase large subunit [EC:4.2.1.35 4.2.1.33]	Valine, leucine and isoleucine biosynthesis/C5-Branched dibasic acid metabolism	437	1	437	0.0E+00	94%	428238862	Bacteria	Cyanobacteria	n	Oscillatoria nigro-viridis PCC 7112, complete genome, product = homoaconitate hydratase family protein (3-isopropylmalate dehydratase, large subunit)
GKJWQY101APBSQ	K00020	3-hydroxyisobutyrate dehydrogenase [EC:1.1.1.31]	Valine, leucine and isoleucine degradation	444	20	444	0.0E+00	99%	257149867	Bacteria	Firmicutes	Bacilli	Lactobacillus rhamnosus Lc 705 whole genome sequence, strain Lc 705, product = 3-hydroxyisobutyrate dehydrogenase
GKJWQY101BTV1D	K00020	3-hydroxyisobutyrate dehydrogenase [EC:1.1.1.31]	Valine, leucine and isoleucine degradation	502	20	502	0.0E+00	100%	257149867	Bacteria	Firmicutes	Bacilli	Lactobacillus rhamnosus Lc 705 whole genome sequence, strain Lc 705, product = 3-hydroxyisobutyrate dehydrogenase
GKJWQY101AX6PV	K00140	methylmalonate-semialdehyde dehydrogenase [EC:1.2.1.27]	Valine, leucine and isoleucine degradation/beta-Alanine metabolism/Inositol phosphate metabolism/Propanoate metabolism	491	1	491	6.0E-154	87%	299068436	Bacteria	Proteobacteria	Betaproteobacteria	Ralstonia solanacearum str. CMR15 plasmid CMR15_mp, complete genome, product = methylmalonate-semialdehyde dehydrogenase
GKJWQY101BN3ES	K00826	branched-chain amino acid aminotransferase [EC:2.6.1.42]	Valine, leucine and isoleucine degradation/Valine, leucine and isoleucine biosynthesis/Pantothenate and CoA biosynthesis	158	1	158	9.0E-58	94%	428238862	Bacteria	Cyanobacteria	n	Oscillatoria nigro-viridis PCC 7112, complete genome, product = branched-chain amino acid aminotransferase
GKJWQY101A4443	K03087	RNA polymerase nonessential primary-like sigma factor	Vibrio cholerae pathogenic cycle	162	1	162	1.0E-76	99%	387575654	Bacteria	Proteobacteria	Betaproteobacteria	Burkholderia sp. KJ006 chromosome 1, complete sequence, product = RNA polymerase sigma factor RpoS
GKJWQY101BXMTR	K09686	antibiotic transport system permease protein	ABC transporters	377	3	377	2.0E-78	99%	471965147	Bacteria	Actinobacteria	Actinobacteria	putative ABC transporter, permease protein [Arthrobacter gangotriensis Lz1y]
GKJWQY101BPNKE	K02015	iron complex transport system permease protein	ABC transporters	486	485	3	7.0E-57	100%	189460866	Bacteria	Bacteroidetes	Bacteroidia	hypothetical protein BACCOP_01513 [Bacteroides coprocola DSM 17136] note = ABC-type enterobactin transport system, permease component
GKJWQY101BF26C	K09808	lipoprotein-releasing system permease protein	ABC transporters	387	386	3	6.0E-66	88%	224025277	Bacteria	Bacteroidetes	Bacteroidia	hypothetical protein BACCOPRO_02016 [Bacteroides coprophilus DSM 18228]
GKJWQY101ASBJ0	K06148	ATP-binding cassette, subfamily C, bacterial	ABC transporters	408	402	1	1.0E-69	88%	428210401	Bacteria	Cyanobacteria	n	bacteriocin-processing peptidase [Chroococcidiopsis thermalis PCC 7203] (note = ABC-type bacteriocin/lantibiotic exporters)
GKJWQY101AUUMV	K11069	spermidine/putrescine transport system substrate-binding protein	ABC transporters	414	3	413	4.0E-63	98%	428318681	Bacteria	Cyanobacteria	n	binding-protein-dependent transport systems inner membrane component [Oscillatoria nigro-viridis PCC 7112]
GKJWQY101AF6LQ	K02013	iron complex transport system ATP-binding protein [EC:3.6.3.34]	ABC transporters	411	4	411	5.0E-79	93%	323490869	Bacteria	Firmicutes	Bacilli	iron transport system ATP-binding protein [Planococcus donghaensis MPA1U2]
GKJWQY101AJY6W	K10017	histidine transport system ATP-binding protein [EC:3.6.3.21]	ABC transporters	236	235	8	9.0E-34	92%	358065153	Bacteria	Firmicutes	Clostridia	hypothetical protein HMPREF9473_03766 [Clostridium hathewayi WAL-18680] region_name = ABC_HisP_GlnQ_permeases
GKJWQY101BSAEK	K02006	cobalt/nickel transport system ATP-binding protein	ABC transporters	410	6	407	6.0E-50	78%	332654627	Bacteria	Firmicutes	Clostridia	putative ABC transporter ATP-binding protein [Ruminococcaceae bacterium D16]
GKJWQY101BKTPU	K11070	spermidine/putrescine transport system permease protein	ABC transporters	416	404	3	2.0E-53	89%	84514738	Bacteria	Proteobacteria	Alphaproteobacteria	spermidine/putrescine ABC transporter, permease protein [Loktanella vestfoldensis SKA53]
GKJWQY101ANA9N	K11004	ATP-binding cassette, subfamily B, bacterial HlyB/CyaB	ABC transporters	491	1	489	3.0E-74	95%	337270029	Bacteria	Proteobacteria	Alphaproteobacteria	type I secretion system ATPase [Mesorhizobium opportunistum WSM2075]
GKJWQY101AXQRA	K11074	putrescine transport system permease protein	ABC transporters	469	22	468	9.0E-43	71%	294676929	Bacteria	Proteobacteria	Alphaproteobacteria	polyamine ABC transporter permease PotC [Rhodobacter capsulatus SB 1003]
GKJWQY101BN6IV	K02041	phosphonate transport system ATP-binding protein	ABC transporters	210	209	3	2.0E-33	94%	390570583	Bacteria	Proteobacteria	Betaproteobacteria	ABC transporter [Burkholderia terrae BS001]
GKJWQY101BN6IV_2		polar amino acid ABC transporter inner membrane subunit	ABC transporters	189	187	2	1.0E-19	83%	390570584	Bacteria	Proteobacteria	Betaproteobacteria	polar amino acid ABC transporter inner membrane subunit [Burkholderia terrae BS001]
GKJWQY101APEXF	K02013	iron complex transport system ATP-binding protein [EC:3.6.3.34]	ABC transporters	258	2	256	3.0E-34	84%	400286648	Bacteria	Proteobacteria	Gammaproteobacteria	ABC transporter-like protein [Psychrobacter sp. PAMC 21119]
GKJWQY101BOU8W_2	K02046	sulfate transport system permease protein	ABC transporters	264	263	3	2.0E-51	100%	71065631	Bacteria	Proteobacteria	Gammaproteobacteria	ABC sulfate/thiosulfate transporter inner membrane protein CysT [Psychrobacter arcticus 273-4]

GKJWQY101BNQIG	K11632	bacitracin transport system permease protein	ABC transporters	340	12	338	5.0E-35	77%	471974841	Bacteria	Firmicutes	Bacilli	FtsX-like permease family protein [Bhargavaea cecembensis DSE10]
GKJWQY101ARCYY	K01551	arsenite-transporting ATPase [EC:3.6.3.16]	Acting on acid anhydrides	398	387	1	5.0E-58	87%	403071345	Bacteria	Firmicutes	Bacilli	Arsenical pump-driving ATPase [Oceanobacillus sp. Ndiop]
GKJWQY101B09E4	K01533	Cu2+-exporting ATPase [EC:3.6.3.4]	Acting on acid anhydrides to catalyse transmembrane movement of substances	208	208	2	2.0E-26	88%	76799990	Bacteria	Firmicutes	Bacilli	copper-translocating P-type ATPase, partial [Streptococcus agalactiae 18RS21]
GKJWQY101BNPQZ	K01284	peptidyl-dipeptidase Dcp [EC:3.4.15.5]	Acting on peptide bonds (peptidases)	382	379	14	4.0E-69	92%	237709863	Bacteria	Bacteroidetes	Bacteroidia	peptidyl-dipeptidase [Bacteroides sp. 9_1_42FAA]
GKJWQY101BPZWT	K09758	aspartate 4-decarboxylase [EC:4.1.1.12]	Alanine, aspartate and glutamate metabolism	373	372	1	2.0E-50	80%	385206715	Bacteria	Proteobacteria	Betaproteobacteria	aspartate 4-decarboxylase [Burkholderia sp. Ch1-1]
GKJWQY101ALPLG	K13821	proline dehydrogenase / delta 1-pyrroline-5-carboxylate dehydrogenase [EC:1.5.1.12 1.5.99.8]	Alanine, aspartate and glutamate metabolism	480	2	478	2.0E-93	95%	400288736	Bacteria	Proteobacteria	Gammaproteobacteria	bifunctional proline dehydrogenase/pyrroline-5-carboxylate dehydrogenase [Psychrobacter sp. PAMC 21119]
GKJWQY101AMLGF	K00262	glutamate dehydrogenase (NADP+) [EC:1.4.1.4]	Alanine, aspartate and glutamate metabolism/Arginine and proline metabolism/Nitrogen metabolism	310	309	13	7.0E-62	100%	189431537	Bacteria	Bacteroidetes	Bacteroidia	glutamate dehydrogenase, NAD-specific [Bacteroides coprocola DSM 17136]
GKJWQY101A4RPZ	K00830	alanine-glyoxylate transaminase / serine-glyoxylate transaminase / serine-pyruvate transaminase [EC:2.6.1.51 2.6.1.45 2.6.1.44]	Alanine, aspartate and glutamate metabolism/Glycine, serine and threonine metabolism/Glyoxylate and dicarboxylate metabolism/Methane metabolism	319	2	316	8.0E-60	92%	399543417	Bacteria	Proteobacteria	Gammaproteobacteria	Serine--glyoxylate aminotransferase [Marinobacter sp. BSs20148], alanine-glyoxylate aminotransferase (AGAT) family
GKJWQY101BPUSX	K00278	L-aspartate oxidase [EC:1.4.3.16]	Alanine, aspartate and glutamate metabolism/Nicotinate and nicotinamide metabolism	398	4	387	1.0E-82	96%	189431608	Bacteria	Bacteroidetes	Bacteroidia	L-aspartate oxidase [Bacteroides coprocola DSM 17136]
GKJWQY101BWE86	K00278	L-aspartate oxidase [EC:1.4.3.16]	Alanine, aspartate and glutamate metabolism/Nicotinate and nicotinamide metabolism	369	4	366	3.0E-78	98%	189431608	Bacteria	Bacteroidetes	Bacteroidia	L-aspartate oxidase [Bacteroides coprocola DSM 17136]
GKJWQY101BMOQ0	K00265	glutamate synthase (NADPH/NADH) large chain [EC:1.4.1.14 1.4.1.13]	Alanine, aspartate and glutamate metabolism/Nitrogen metabolism	496	458	3	9.0E-88	94%	283778000	Bacteria	Planctomycetes	Planctomycetia	glutamate synthase (NADH) [Pirellula staleyi DSM 6068]
GKJWQY101BXEDH_2	K00259	alanine dehydrogenase [EC:1.4.1.1]	Alanine, aspartate and glutamate metabolism/Taurine and hypotaurine metabolism	222	5	220	8.0E-33	88%	415884092	Bacteria	Firmicutes	Bacilli	alanine dehydrogenase [Bacillus methanolicus MGA3]
GKJWQY101BX3GV	K01654	N-acetylneuraminate synthase [EC:2.5.1.56]	Amino sugar and nucleotide sugar metabolism	373	21	371	2.0E-27	66%	163758286	Bacteria	Proteobacteria	Alphaproteobacteria	N-acetylneuraminic acid synthetase [Hoeflea phototrophica DFL-43]
GKJWQY101B2KLB	K00075	UDP-N-acetylmuramate dehydrogenase [EC:1.1.1.158]	Amino sugar and nucleotide sugar metabolism/Peptidoglycan biosynthesis	315	1	306	4.0E-55	93%	289551402	Bacteria	Firmicutes	Bacilli	UDP-N-acetylenolpyruvoylglucosamine reductase [Staphylococcus lugdunensis HKU09-01]
GKJWQY101A5QWZ	K01881	prolyl-tRNA synthetase [EC:6.1.1.15]	Aminoacyl-tRNA biosynthesis	362	2	352	2.0E-17	62%	260947818	Eukaryota	Ascomycota	Saccharomycotina	hypothetical protein CLUG_01665 [Clavispora lusitaniae ATCC 42720]
GKJWQY101AV49L	K01889	phenylalanyl-tRNA synthetase alpha chain [EC:6.1.1.20]	Aminoacyl-tRNA biosynthesis	161	5	160	6.0E-18	90%	150004775	Bacteria	Bacteroidetes	Bacteroidia	phenylalanyl-tRNA synthetase subunit alpha [Bacteroides vulgatus ATCC 8482]
GKJWQY101AV49L_2	K01889	phenylalanyl-tRNA synthetase alpha chain [EC:6.1.1.20]	Aminoacyl-tRNA biosynthesis	269	2	268	2.0E-40	82%	330996958	Bacteria	Bacteroidetes	Bacteroidia	phenylalanine--tRNA ligase, alpha subunit [Paraprevotella xylaniphila YIT 11841]
GKJWQY101AGV5Y	K01892	histidyl-tRNA synthetase [EC:6.1.1.21]	Aminoacyl-tRNA biosynthesis	474	474	1	2.0E-101	100%	357043085	Bacteria	Bacteroidetes	Bacteroidia	histidyl-tRNA synthetase [Prevotella histicola F0411]
GKJWQY101A8MT2	K01892	histidyl-tRNA synthetase [EC:6.1.1.21]	Aminoacyl-tRNA biosynthesis	497	3	497	2.0E-109	97%	357043085	Bacteria	Bacteroidetes	Bacteroidia	histidyl-tRNA synthetase [Prevotella histicola F0411]
GKJWQY101BM66A	K01892	histidyl-tRNA synthetase [EC:6.1.1.21]	Aminoacyl-tRNA biosynthesis	474	474	1	2.0E-101	100%	357043085	Bacteria	Bacteroidetes	Bacteroidia	histidyl-tRNA synthetase [Prevotella histicola F0411]
GKJWQY101BXGRA	K09461	anthraniloyl-CoA monooxygenase [EC:1.14.13.40]	Aminobenzoate degradation	242	240	1	1.0E-41	93%	440227685	Bacteria	Proteobacteria	Alphaproteobacteria	2-amninobenzoyl-CoA monooxygenase/reductase [Rhizobium tropici CIAT 899]
GKJWQY101BGCC7	K01173	endonuclease G, mitochondrial	Apoptosis	359	358	2	9.0E-45	75%	445497248	Bacteria	Proteobacteria	Betaproteobacteria	DNA/RNA non-specific endonuclease [Janthinobacterium sp. HH01]
GKJWQY101AD9QP	K10536	agmatine deiminase [EC:3.5.3.12]	Arginine and proline metabolism	463	461	3	3.0E-92	98%	281420760	Bacteria	Bacteroidetes	Bacteroidia	peptidylarginine deiminase-related protein [Prevotella copri DSM 18205]
GKJWQY101BCW87	K00611	ornithine carbamoyltransferase [EC:2.1.3.3]	Arginine and proline metabolism	236	1	225	4.0E-44	100%	428319688	Bacteria	Cyanobacteria	n	ornithine carbamoyltransferase [Oscillatoria nigro-viridis PCC 7112]
GKJWQY101AHUTZ	K01438	acetylornithine deacetylase [EC:3.5.1.16]	Arginine and proline metabolism	382	382	2	2.0E-64	86%	228983691	Bacteria	Firmicutes	Bacilli	Acetylornitine deacetylase [Bacillus thuringiensis serovar tochigiensis BGSC 4Y1]
GKJWQY101AEBTJ	K00619	amino-acid N-acetyltransferase [EC:2.3.1.1]	Arginine and proline metabolism	447	447	1	6.0E-48	73%	407712609	Bacteria	Proteobacteria	Betaproteobacteria	amino-acid N-acetyltransferase [Burkholderia phenoliruptrix BR3459a]
GKJWQY101BZEK4	K03079	L-ribulose-5-phosphate 3-epimerase [EC:5.1.3.22]	Ascorbate and aldarate metabolism	303	303	4	6.0E-50	86%	225386566	Bacteria	Firmicutes	Clostridia	hypothetical protein CLOSTASPAR_00313 [Clostridium asparagiforme DSM 15981] note = putative L-xylulose 5-phosphate 3-epimerase; reviewed
GKJWQY101BQJK1	K11003	hemolysin D	Bacterial secretion system	464	3	464	2.0E-72	84%	337270028	Bacteria	Proteobacteria	Alphaproteobacteria	HlyD family type I secretion membrane fusion protein [Mesorhizobium opportunistum WSM2075]
GKJWQY101A2NZL	K03076	preprotein translocase subunit SecY	Bacterial secretion system/Protein export	446	1	444	2.0E-42	78%	261367783	Bacteria	Firmicutes	Clostridia	preprotein translocase, SecY subunit [Subdoligranulum variabile DSM 15176]
GKJWQY101AOY6A	K07823	3-oxoadipyl-CoA thiolase [EC:2.3.1.174]	Benzoate degradation	185	168	1	2.0E-24	92%	471966247	Bacteria	Actinobacteria	Actinobacteria	beta-ketoadipyl CoA thiolase [Arthrobacter gangotriensis Lz1y]
GKJWQY101ASHYA	K00517	[EC:1.14.-.-]	Bisphenol degradation/Polycyclic aromatic hydrocarbon degradation/Aminobenzoate degradation	400	397	2	1.0E-50	78%	434407557	Bacteria	Cyanobacteria	n	cytochrome P450 [Cylindrospermum stagnale PCC 7417]
GKJWQY101ARU2K	K07516	3-hydroxyacyl-CoA dehydrogenase [EC:1.1.1.35]	Carbon fixation pathways in prokaryotes	425	1	396	5.0E-41	79%	359790820	Bacteria	Proteobacteria	Alphaproteobacteria	3-hydroxyacyl-CoA dehydrogenase NAD-binding protein [Mesorhizobium alhagi CCNWXJ12-2]
GKJWQY101BSTX6	K11749	regulator of sigma E protease [EC:3.4.24.-]	Cell cycle - Caulobacter	496	494	3	9.0E-82	87%	389815285	Bacteria	Firmicutes	Bacilli	zinc metalloprotease Lmo1318 [Planococcus antarcticus DSM 14505]
GKJWQY101AYPYY	K01681	aconitate hydratase 1 [EC:4.2.1.3]	Citrate cycle (TCA cycle)/Lysine degradation/Glyoxylate and dicarboxylate metabolism/Carbon fixation pathways in prokaryotes	393	392	3	3.0E-81	99%	389820589	Bacteria	Firmicutes	Bacilli	aconitate hydratase [Planococcus antarcticus DSM 14505]
GKJWQY101BF22D	K01243	S-adenosylhomocysteine/5'-methylthioadenosine nucleosidase [EC:3.2.2.9]	Cysteine and methionine metabolism	249	249	7	2.0E-29	81%	351728658	Bacteria	Proteobacteria	Betaproteobacteria	mta/sah nucleosidase [Acidovorax radicis N35]
GKJWQY101ABL6A	K05396	D-cysteine desulfhydrase [EC:4.4.1.15]	Cysteine and methionine metabolism	372	1	372	2.0E-28	64%	212703443	Bacteria	Proteobacteria	Deltaproteobacteria	hypothetical protein DESPIG_01487 [Desulfovibrio piger ATCC 29098] (note: D-cysteine desulfhydrase; Validated)
GKJWQY101BZOWH	K01921	D-alanine-D-alanine ligase [EC:6.3.2.4]	D-Alanine metabolism/Peptidoglycan biosynthesis	361	1	360	2.0E-43	80%	88854490	Bacteria	Actinobacteria	n	D-alanine--D-alanine ligase [marine actinobacterium PHSC20C1]
GKJWQY101ADGTJ	K00567	methylated-DNA-[protein]-cysteine S-methyltransferase [EC:2.1.1.63]	DNA repair and recombination [Single Strand Breaks Repair]	346	338	3	1.0E-25	66%	302387653	Bacteria	Firmicutes	Clostridia	methylated-DNA--protein-cysteine methyltransferase [Clostridium saccharolyticum WM1]
GKJWQY101BWY0I	K01669	deoxyribodipyrimidine photo-lyase [EC:4.1.99.3]	DNA repair and recombination proteins	139	2	124	9.0E-11	78%	410861788	Bacteria	Proteobacteria	Gammaproteobacteria	deoxyribodipyrimidine photo-lyase [Alteromonas macleodii AltDE1]
GKJWQY101BH9UZ	K02314	replicative DNA helicase [EC:3.6.4.12]	DNA replication/Cell cycle - Caulobacter	341	3	341	7.0E-35	74%	224477942	Bacteria	Firmicutes	Bacilli	Replicative DNA helicase [Staphylococcus carnosus subsp. carnosus TM300]
GKJWQY101BSHB9	K01153	type I restriction enzyme, R subunit [EC:3.1.21.3]	Endodeoxyribonucleases producing 5'-phosphomonoesters	399	394	2	3.0E-54	81%	404494602	Bacteria	Proteobacteria	Deltaproteobacteria	type I restriction-modification system, restriction subunit [Pelobacter carbinolicus DSM 2380]
GKJWQY101AF0JM	K01273	membrane dipeptidase [EC:3.4.13.19]	Family M19: membrane dipeptidase family	486	486	1	7.0E-98	94%	226944628	Bacteria	Proteobacteria	Gammaproteobacteria	peptidase M19 [Azotobacter vinelandii DJ]

GKJWQY101AJ8K7	K00249	acyl-CoA dehydrogenase [EC:1.3.99.3]	Fatty acid metabolism/Valine, leucine and isoleucine degradation/beta-Alanine metabolism/Propanoate metabolism/PPAR signaling pathway	399	3	398	6.00E-59	92%	146298219	Bacteria	Bacteroidetes	Flavobacteria	acyl-CoA dehydrogenase [Flavobacterium johnsoniae UW101]
GKJWQY101AWYJI	K00849	galactokinase [EC:2.7.1.6]	Galactose metabolism/Amino sugar and nucleotide sugar metabolism	414	2	409	2.0E-82	97%	325298068	Bacteria	Bacteroidetes	Bacteroidia	galactokinase [Bacteroides salanitronis DSM 18170]
GKJWQY101BXW5Y	K01190	beta-galactosidase [EC:3.2.1.23]	Galactose metabolism/Other glycan degradation/Sphingolipid metabolism	401	1	396	5.0E-38	71%	149280049	Bacteria	Bacteroidetes	Sphingobacteriia	beta-galactosidase [Pedobacter sp. BAL39]
GKJWQY101AGQIA	K00257	[EC:1.3.99.-]	Geraniol degradation	313	311	12	5.0E-52	93%	374595657	Bacteria	Bacteroidetes	Flavobacteria	acyl-CoA dehydrogenase domain-containing protein [Gillisia limnaea DSM 15749]
GKJWQY101BUN1T	K01919	glutamate--cysteine ligase [EC:6.3.2.2]	Glutathione metabolism	150	148	8	2.0E-23	100%	393719433	Bacteria	Proteobacteria	Alphaproteobacteria	glutamate--cysteine ligase [Sphingomonas echinoides ATCC 14820]
GKJWQY101BHN6U	K00681	gamma-glutamyltranspeptidase [EC:2.3.2.2]	Glutathione metabolism/Taurine and hypotaurine metabolism/Cyanoamino acid metabolism/Arachidonic acid metabolism	367	366	16	1.0E-57	89%	398310944	Bacteria	Firmicutes	Bacilli	gamma-glutamyltranspeptidase [Bacillus mojavensis RO-H-1]
GKJWQY101AJC8H	K00981	phosphatidate cytidylyltransferase [EC:2.7.7.41]	Glycerophospholipid metabolism/Phosphatidylinositol signaling system	154	1	144	5.0E-20	93%	323142324	Bacteria	Firmicutes	Negativicutes	phosphatidate cytidylyltransferase [Phascolarctobacterium succinatutens YIT 12067]
GKJWQY101AG8YC	K00281	glycine dehydrogenase [EC:1.4.4.2]	Glycine, serine and threonine metabolism	326	318	1	7.0E-58	93%	300770671	Bacteria	Bacteroidetes	Sphingobacteriia	glycine dehydrogenase (decarboxylating) [Sphingobacterium spiritivorum ATCC 33861]
GKJWQY101BFOCB	K00108	choline dehydrogenase [EC:1.1.99.1]	Glycine, serine and threonine metabolism	347	1	345	3.0E-60	90%	433773923	Bacteria	Proteobacteria	Alphaproteobacteria	choline dehydrogenase-like flavoprotein [Mesorhizobium australicum WSM2073]
GKJWQY101ASOB_2	K00281	glycine dehydrogenase [EC:1.4.4.2]	Glycine, serine and threonine metabolism	96	1	96	2.0E-09	93%	89093021	Bacteria	Proteobacteria	Gammaproteobacteria	glycine dehydrogenase [Neptuniibacter caesariensis]
GKJWQY101AVAMV	K00274	monoamine oxidase [EC:1.4.3.4]	Glycine, serine and threonine metabolism/Arginine and proline metabolism/Histidine metabolism/Tyrosine metabolism/Phenylalanine metabolism/Tryptophan metabolism/Isoquinoline alkaloid biosynthesis	300	2	295	9.0E-43	96%	400287901	Bacteria	Proteobacteria	Gammaproteobacteria	L-amino acid oxidase [Psychrobacter sp. PAMC 21119]
GKJWQY101A4KSO	K01181	endo-1,4-beta-xylanase [EC:3.2.1.8]	Glycosidases[hydrolyse O- and S-glycosyl]	379	3	275	6.0E-33	75%	393718930	Bacteria	Proteobacteria	Alphaproteobacteria	endo-1,4-beta-xylanase [Sphingomonas echinoides ATCC 14820]
GKJWQY101A3F2S	K01966	propionyl-CoA carboxylase beta chain [EC:6.4.1.3]	Glyoxylate and dicarboxylate metabolism/Propanoate metabolism/Carbon fixation pathways in prokaryotes/Valine, leucine and isoleucine degradation	340	338	3	6.0E-68	97%	332877849	Bacteria	Bacteroidetes	Flavobacteriia	carboxyl transferase domain protein [Capnocytophaga sp. oral taxon 329 str. F0087]
GKJWQY101BB17C	K02501	glutamine amidotransferase [EC:2.4.2.-]	Histidine metabolism	336	335	3	2.0E-44	76%	163758289	Bacteria	Proteobacteria	Alphaproteobacteria	imidazole glycerol phosphate synthase, glutamine amidotransferase subunit [Hoeflea phototrophica DFL-43]
GKJWQY101AZ7ML	K03551	holliday junction DNA helicase RuvB [EC:3.6.4.12]	Homologous recombination	336	325	2	2.0E-56	90%	83592425	Bacteria	Proteobacteria	Alphaproteobacteria	Holliday junction DNA helicase RuvB [Rhodospirillum rubrum ATCC 11170]
GKJWQY101BEK18	K00666	fatty-acyl-CoA synthase [EC:6.2.1.-]	Lipid biosynthesis [Ligases forming carbon-sulfur bonds]	385	381	10	4.0E-59	84%	359771954	Bacteria	Actinobacteria	Actinobacteria	putative fatty-acid--CoA ligase [Gordonia effusa NBRC 100432]
GKJWQY101AQ218	K00666	fatty-acyl-CoA synthase [EC:6.2.1.-]	Lipid biosynthesis [Ligases forming carbon-sulfur bonds]	388	2	338	2.0E-31	59%	73541092	Bacteria	Proteobacteria	Betaproteobacteria	long-chain-fatty-acid--CoA ligase [Ralstonia eutropha JMP134]
GKJWQY101A2KTL	K00674	2,3,4,5-tetrahydropyridine-2-carboxylate N-succinyltransferase [EC:2.3.1.117]	Lysine biosynthesis	234	230	6	1.0E-38	97%	302024578	Bacteria	Firmicutes	Bacilli	2,3,4,5-tetrahydropyridine-2,6-dicarboxylate N-acetyltransferase [Streptococcus suis 05HAS68]
GKJWQY101ADMIV	K01417	putative zinc metalloprotease [EC:3.4.24.-]	Metalloendopeptidases	235	2	223	9.0E-31	85%	154490034	Bacteria	Bacteroidetes	Bacteroidia	hypothetical protein PARMER_00263 [Parabacteroides merdae ATCC 43184]
GKJWQY101BJ19A	K01953	asparagine synthase (glutamine-hydrolysing) [EC:6.3.5.4]	Nitrogen metabolism/Alanine, aspartate and glutamate metabolism	420	1	420	3.0E-45	72%	403236697	Bacteria	Firmicutes	Bacilli	asparagine synthetase [Bacillus sp. 10403023]
GKJWQY101AHO0M	K00370	nitrate reductase 1, alpha subunit [EC:1.7.99.4]	Nitrogen metabolism/Two-component system	130	1	129	2.0E-13	83%	86137746	Bacteria	Proteobacteria	Alphaproteobacteria	respiratory nitrate reductase, alpha subunit [Roseobacter sp. MED193]
GKJWQY101AHO0M_2	K00370	nitrate reductase 1, alpha subunit [EC:1.7.99.4]	Nitrogen metabolism/Two-component system	304	7	294	1.0E-54	98%	262374579	Bacteria	Proteobacteria	Gammaproteobacteria	nitrate reductase, alpha subunit [Acinetobacter junii SH205]
GKJWQY101BS60U	K02051	sulfonate/nitrate/taurine transport system substrate-binding protein	NitT/TauT family transport system	356	3	356	1.0E-72	99%	404395813	Bacteria	Proteobacteria	Betaproteobacteria	hypothetical protein HMPREF0989_00609 [Ralstonia sp. 5_2_56FAA] note = ABC-type nitrate/sulfonate/bicarbonate transport systems, periplasmic components
GKJWQY101BP7PB	K00604	methionyl-tRNA formyltransferase [EC:2.1.2.9]	One carbon pool by folate/Aminoacyl-tRNA biosynthesis	265	209	3	2.0E-34	97%	400287034	Bacteria	Proteobacteria	Gammaproteobacteria	methionyl-tRNA formyltransferase [Psychrobacter sp. PAMC 21119]
GKJWQY101ANZNS	K01206	alpha-L-fucosidase [EC:3.2.1.51]	Other glycan degradation	417	2	415	1.0E-64	83%	374385611	Bacteria	Bacteroidetes	Bacteroidia	hypothetical protein HMPREF9449_01500 [Odoribacter laneus YIT 12061]
GKJWQY101BHU3U	K05903	NADH dehydrogenase (quinone) [EC:1.6.99.5]	Oxidative phosphorylation	444	444	1	1.0E-59	90%	261368112	Bacteria	Firmicutes	Clostridia	putative Ech hydrogenase, subunit EchA [Subdoligranulum variabile DSM 15176] (note = NADH-iquinone/plastoquinone (complex I))
GKJWQY101BQYQT	K00333	NADH dehydrogenase I subunit D [EC:1.6.5.3]	Oxidative phosphorylation/Nitrogen metabolism	252	245	6	9.0E-47	98%	149280567	Bacteria	Bacteroidetes	Sphingobacteriia	NADH dehydrogenase I chain D [Pedobacter sp. BAL39]
GKJWQY101AMELW	K00335	NADH dehydrogenase I subunit F [EC:1.6.5.3]	Oxidative phosphorylation/Nitrogen metabolism	290	7	282	1.0E-44	90%	253579944	Bacteria	Firmicutes	Clostridia	NADH dehydrogenase I subunit F [Ruminococcus sp. 5_1_39BFAA]
GKJWQY101AOGKN	K00412	ubiquinol-cytochrome c reductase cytochrome b subunit [EC:1.10.2.2]	Oxidative phosphorylation/Nitrogen metabolism/Two-component system	335	333	22	8.0E-38	96%	400288130	Bacteria	Proteobacteria	Gammaproteobacteria	cytochrome b/b6-like protein [Psychrobacter sp. PAMC 21119]
GKJWQY101A11NA	K02111	F-type H+-transporting ATPase subunit alpha [EC:3.6.3.14]	Oxidative phosphorylation/Photosynthesis	184	184	2	4.0E-34	100%	402569635	Bacteria	Proteobacteria	Betaproteobacteria	F0F1 ATP synthase subunit alpha [Burkholderia cepacia GG4]
GKJWQY101BN3QR	K00428	cytochrome c peroxidase [EC:1.11.1.5]	Oxidoreductases [Acting on a peroxide as acceptor]	513	512	9	7.0E-93	88%	265763882	Bacteria	Bacteroidetes	Bacteroidia	cytochrome-C peroxidase [Bacteroides sp. 2_1_16]
GKJWQY101ABF0Q	K00433	chloride peroxidase [EC:1.11.1.10]	Oxidoreductases [Acting on a peroxide as acceptor]	374	374	3	2.0E-81	99%	387903695	Bacteria	Proteobacteria	Betaproteobacteria	Non-heme chloroperoxidase [Burkholderia sp. KJ006]
GKJWQY101ACH7I	K00859	dephospho-CoA kinase [EC:2.7.1.24]	Pantothenate and CoA biosynthesis	345	1	333	1.0E-31	81%	308177711	Bacteria	Actinobacteria	Actinobacteridae	dephospho-CoA kinase [Arthrobacter arilaitensis Re117]
GKJWQY101AOU9D	K13038	phosphopantothenoylcysteine decarboxylase / phosphopantothenate--cysteine ligase [EC:6.3.2.5 4.1.1.36]	Pantothenate and CoA biosynthesis	329	5	319	3.0E-27	64%	403667677	Bacteria	Firmicutes	Bacilli	phosphopantothenoylcysteine synthetase/decarboxylase [Kurthia sp. JC8E]

GKJWQY101A5BE1	K01918	pantoate--beta-alanine ligase [EC:6.3.2.1]	Pantothenate and CoA biosynthesis/beta-Alanine metabolism	409	404	3	2.0E-90	100%	471964577	Bacteria	Actinobacteria	Actinobacteria	pantoate/beta-alanine ligase [Arthrobacter gangotriensis Lz1y]
GKJWQY101BDF2G	K01685	altronate hydrolase [EC:4.2.1.7]	Pentose and glucuronate interconversions	397	3	392	5.0E-34	67%	325300639	Bacteria	Bacteroidetes	Bacteroidia	altronate dehydratase [Bacteroides salanitronis DSM 18170]
GKJWQY101BVCND	K00854	xylulokinase [EC:2.7.1.17]	Pentose and glucuronate interconversions	520	519	4	2.0E-73	84%	332655340	Bacteria	Firmicutes	Clostridia	xylulokinase [Ruminococcaceae bacterium D16]
GKJWQY101BO1HI	K00012	UDPglucose 6-dehydrogenase [EC:1.1.1.22]	Pentose and glucuronate interconversions/Ascorbate and aldarate metabolism/Amino sugar and nucleotide sugar metabolism/ Starch and sucrose metabolism	256	9	254	5.0E-32	85%	298209197	Bacteria	Bacteroidetes	Flavobacteria	UDP-glucose 6-dehydrogenase [Croceibacter atlanticus HTCC2559]
GKJWQY101BVVMM	K00012	UDPglucose 6-dehydrogenase [EC:1.1.1.22]	Pentose and glucuronate interconversions/Ascorbate and aldarate metabolism/Amino sugar and nucleotide sugar metabolism/ Starch and sucrose metabolism	278	15	269	1.0E-47	96%	164657686	Eukaryote	Basidiomycota	Ustilaginomycotina	hypothetical protein MGL_2955 [Malassezia globosa CBS 7966]
GKJWQY101BRUMM	K05367	penicillin-binding protein 1C [EC:2.4.1.-]	Peptidoglycan biosynthesis	485	478	2	5.0E-66	74%	149276515	Bacteria	Bacteroidetes	Sphingobacteriia	putative penicillin-binding protein [Pedobacter sp. BAL39]
GKJWQY101BUDXS	K02563	UDP-N-acetylglucosamine--N-acetylmuramyl-(pentapeptide) pyrophosphoryl-undecaprenol N-acetylglucosamine transferase [EC:2.4.1.227]	Peptidoglycan biosynthesis/Cell cycle - Caulobacter	347	338	3	1.0E-41	77%	198274308	Bacteria	Bacteroidetes	Bacteroidia	hypothetical protein BACPLE_00452 [Bacteroides plebeius DSM 17135]
GKJWQY101AD9H9	K04564	superoxide dismutase, Fe-Mn family [EC:1.15.1.1]	Peroxisome	223	222	1	2.0E-21	74%	336114384	Bacteria	Firmicutes	Bacilli	Superoxide dismutase [Bacillus coagulans 2-6]
GKJWQY101AS6OK	K03781	catalase [EC:1.11.1.6]	Peroxisome	485	2	484	3.0E-81	85%	319893610	Bacteria	Firmicutes	Bacilli	catalase [Staphylococcus pseudintermedius HKU10-03]
GKJWQY101BVCXB	K00014	shikimate dehydrogenase [EC:1.1.1.25]	Phenylalanine, tyrosine and tryptophan biosynthesis	292	2	292	2.0E-34	77%	225866315	Bacteria	Firmicutes	Bacilli	shikimate 5-dehydrogenase [Bacillus cereus 03BB102]
GKJWQY101BN853	K04518	prephenate dehydratase [EC:4.2.1.51]	Phenylalanine, tyrosine and tryptophan biosynthesis	355	1	354	1.0E-68	95%	458759422	Bacteria	Firmicutes	Bacilli	Prephenate dehydratase [Planococcus halocryophilus Or1]
GKJWQY101BHNZ6	K05349	beta-glucosidase [EC:3.2.1.21]	Phenylpropanoid biosynthesis	405	1	405	9.0E-62	83%	317480750	Bacteria	Bacteroidetes	Bacteroidia	glycosyl hydrolase family 3 C terminal domain-containing protein [Bacteroides sp. 4_1_36]
GKJWQY101A9B69	K00067	dTDP-4-dehydrorhamnose reductase [EC:1.1.1.133]	Polyketide sugar unit biosynthesis/Streptomycin biosynthesis	457	5	457	5.0E-60	76%	15892379	Bacteria	Proteobacteria	Alphaproteobacteria	dTDP-4-dehydrorhamnose reductase. [Rickettsia conorii str. Malish 7]
GKJWQY101ARUMF	K00768	nicotinate-nucleotide--dimethylbenzimidazole phosphoribosyltransferase [EC:2.4.2.21]	Porphyrin and chlorophyll metabolism	250	240	4	5.0E-24	81%	325262503	Bacteria	Firmicutes	Clostridia	nicotinate-nucleotide--dimethylbenzimidazole phosphoribosyltransferase [Clostridium sp. D5]
GKJWQY101A3PTQ	K12670	oligosaccharyltransferase complex subunit beta	Protein processing in endoplasmic reticulum	383	382	2	7.0E-59	86%	164662188	Eukaryota	Basidiomycota	Ustilaginomycotina	hypothetical protein MGL_0809 [Malassezia globosa CBS 7966] note = oligosaccharyltransferase 48 kDa subunit beta; pfam03345
GKJWQY101BPKHW	K14018	phospholipase A-2-activating protein	Protein processing in endoplasmic reticulum	439	437	3	5.0E-48	72%	164657618	Eukaryota	Basidiomycota	Ustilaginomycotina	hypothetical protein MGL_2921 [Malassezia globosa CBS 7966]
GKJWQY101A5QPE	K01945	phosphoribosylamine--glycine ligase [EC:6.3.4.13]	Purine metabolism	546	526	17	1.0E-73	81%	308178295	Bacteria	Actinobacteria	Actinobacteria	phosphoribosylamine--glycine ligase [Arthrobacter arilaitensis Re117]
GKJWQY101BHOCV	K00951	GTP pyrophosphokinase [EC:2.7.6.5]	Purine metabolism	495	494	15	9.0E-87	90%	325298624	Bacteria	Bacteroidetes	Bacteroidia	(p)ppGpp synthetase I SpoT/RelA [Bacteroides salanitronis DSM 18170]
GKJWQY101BQAM3	K00073	ureidoglycolate dehydrogenase [EC:1.1.1.154]	Purine metabolism	468	2	457	6.0E-74	87%	458798528	Bacteria	Firmicutes	Bacilli	ureidoglycolate dehydrogenase [Bacillus sonorensis L12]
GKJWQY101BEEY9	K14977	ureidoglycine aminohydrolase [EC:3.5.3.-]	Purine metabolism	374	3	356	2.0E-69	90%	220914710	Bacteria	Proteobacteria	Alphaproteobacteria	hypothetical protein [Methylobacterium nodulans ORS 2060]
GKJWQY101ACXQT_2	K01939	adenylosuccinate synthase [EC:6.3.4.4]	Purine metabolism/Alanine, aspartate and glutamate metabolism	155	140	3	3.0E-06	65%	322437074	Bacteria	Acidobacteria	Acidobacteriales	adenylosuccinate synthetase [Granulicella tundricola MP5ACTX9]
GKJWQY101BNDWQ	K00764	amidophosphoribosyltransferase [EC:2.4.2.14]	Purine metabolism/Alanine, aspartate and glutamate metabolism	520	520	14	5.0E-102	95%	189461995	Bacteria	Bacteroidetes	Bacteroidia	hypothetical protein BACCOP_02664 [Bacteroides coprocola DSM17136] note = Glutamine phosphoribosylpyrophosphate amidotransferase
GKJWQY101BSHTT	K02428	nucleoside-triphosphate pyrophosphatase [EC:3.6.1.19]	Purine metabolism/Pyrimidine metabolism	250	1	249	4.0E-43	90%	389819483	Bacteria	Firmicutes	Bacilli	HAM1-like protein [Planococcus antarcticus DSM 14505] and pyrophosphate-releasing xanthosine/ inosine triphosphatase
GKJWQY101BROII	K02342	DNA polymerase III subunit epsilon [EC:2.7.7.7]	Purine metabolism/Pyrimidine metabolism/DNA replication/Mismatch repair/Homologous recombination	210	2	208	9.0E-19	73%	375146288	Bacteria	Bacteroidetes	Sphingobacteriia	DNA polymerase III subunit epsilon [Niastella koreensis GR20-10]
GKJWQY101B0W4V	K02065	putative ABC transport system ATP-binding protein	Putative ABC transport system	341	341	3	3.0E-49	74%	94498321	Bacteria	Proteobacteria	Alphaproteobacteria	ABC transporter, ATP-binding protein [Sphingomonas sp. SKA58]
GKJWQY101BECUD	K01493	dCMP deaminase [EC:3.5.4.12]	Pyrimidine metabolism	384	329	9	7.0E-62	92%	256826526	Bacteria	Actinobacteria	Coriobacteridae	deoxycytidylate deaminase [Cryptobacterium curtum DSM 15641]
GKJWQY101BRT8J	K01937	CTP synthase [EC:6.3.4.2]	Pyrimidine metabolism	306	1	249	2.0E-33	80%	308175443	Bacteria	Firmicutes	Bacilli	CTP synthetase [Bacillus amyloliquefaciens DSM 7]
GKJWQY101AOB8M	K02825	pyrimidine operon attenuation protein / uracil phosphoribosyltransferase [EC:2.4.2.9]	Pyrimidine metabolism	234	6	233	1.0E-20	73%	223936078	Bacteria	Verrucomicrobia	Verrucomicrobiae	Uracil phosphoribosyltransferase [Pedosphaera parvula Ellin514]
GKJWQY101BMA7B	K00609	aspartate carbamoyltransferase catalytic subunit [EC:2.1.3.2]	Pyrimidine metabolism/Alanine, aspartate and glutamate metabolism	182	178	2	6.0E-21	81%	375089143	Bacteria	Firmicutes	Bacilli	aspartate carbamoyltransferase [Dolosigranulum pigrum ATCC 51524]
GKJWQY101AATB5	K01362	E3.4.21.-	Serine endopeptidases	422	417	1	4.0E-80	97%	294795056	Bacteria	Firmicutes	Negativicutes	putative serine protease HtrA [Veillonella sp. 3_1_44]
GKJWQY101BFG0O	K01134	arylsulfatase A [EC:3.1.6.8]	Sphingolipid metabolism	202	201	1	2.0E-16	62%	149198675	Bacteria	Lentisphaerae	Lentisphaeria	arylsulphatase A [Lentisphaera araneosa HTCC2155]
GKJWQY101AXJYB	K00706	1,3-beta-glucan synthase [EC:2.4.1.34]	Starch and sucrose metabolism	397	384	16	3.0E-72	94%	164662831	Eukaryote	Basidiomycota	Exobasidiomycetes	hypothetical protein MGL_0312 [Malassezia globosa CBS 7966] note = 1,3-beta-glucan synthase component
GKJWQY101BPTLK	K00975	glucose-1-phosphate adenylyltransferase [EC:2.7.7.27]	Starch and sucrose metabolism/Amino sugar and nucleotide sugar metabolism	338	8	337	1.0E-51	87%	375145467	Bacteria	Bacteroidetes	Bacteroidia	glucose-1-phosphate adenylyltransferase [Niastella koreensis GR20-10]
GKJWQY101AZOQD	K01662	1-deoxy-D-xylulose-5-phosphate synthase [EC:2.2.1.7]	Thiamine metabolism/Terpenoid backbone biosynthesis	246	6	245	1.0E-22	77%	150004317	Bacteria	Bacteroidetes	Bacteroidia	1-deoxy-D-xylulose-5-phosphate synthase [Bacteroides vulgatus ATCC 8482]
GKJWQY101BNN0W	K00982	glutamate-ammonia-ligase adenylyltransferase [EC:2.7.7.42]	Transferases[Adenylyltransferase/ glutamate--ammonia-ligase]	419	419	3	6.0E-61	87%	88854956	Bacteria	Actinobacteria	n	glutamine synthetase adenylyltransferase [marine actinobacterium PHSC20C1]
GKJWQY101AL5GX	K07154	serine/threonine-protein kinase HipA [EC:2.7.11.1]	Transferring phosphorus-containing groups	406	405	1	2.0E-70	88%	357027749	Bacteria	Proteobacteria	Alphaproteobacteria	HipA N-terminal domain-containing protein [Mesorhizobium amorphae CCNWGS0123]
GKJWQY101BV8AW	K07668	two-component system, OmpR family, response regulator VicR	Two-component system	469	1	468	5.0E-104	97%	323487712	Bacteria	Firmicutes	Bacilli	response regulator [Planococcus donghaensis MPA1U2] region_name = REC (<1..49); region_name = trans_reg_C (67..>156)
GKJWQY101BRBAH	K07644	two-component system, OmpR family, heavy metal sensor histidine kinase CusS [EC:2.7.13.3]	Two-component system	418	413	3	8.0E-41	67%	78063616	Bacteria	Proteobacteria	Betaproteobacteria	heavy metal sensor signal transduction histidine kinase [Burkholderia sp. 383]
GKJWQY101B1X48	K07787	Cu(I)/Ag(I) efflux system membrane protein CusA	Two-component system	484	1	483	1.0E-100	97%	416909337	Bacteria	Proteobacteria	Betaproteobacteria	CzcA family heavy metal efflux protein, partial [Burkholderia sp. TJI49]
GKJWQY101BP6MS	K07798	Cu(I)/Ag(I) efflux system membrane protein CusB	Two-component system	384	378	1	1.0E-52	82%	413959210	Bacteria	Proteobacteria	Betaproteobacteria	copper efflux system membrane protein [Burkholderia sp. SJ98]

GKJWQY101BGPT5	K07636	two-component system, OmpR family, phosphate regulon sensor histidine kinase PhoR [EC:2.7.13.3]	Two-component system	386	1	384	3.0E-82	99%	309778701	Bacteria	Proteobacteria	Betaproteobacteria	sensor protein CzcS [Ralstonia sp. 5_7_47FAA]
GKJWQY101AOVFW	K07638	two-component system, OmpR family, osmolarity sensor histidine kinase EnvZ [EC:2.7.13.3]	Two-component system	244	243	1	5.0E-47	97%	400287352	Bacteria	Proteobacteria	Gammaproteobacteria	histidine kinase [Psychrobacter sp. PAMC 21119] note = osmolarity sensor protein
GKJWQY101BAQ3Y	K00680	[EC:2.3.1.-]	Tyrosine metabolism/Benzoate degradation/Aminobenzoate degradation/Ethylbenzene degradation/Limonene and pinene degradation	407	3	407	2.0E-91	99%	354806737	Bacteria	Firmicutes	Bacilli	acetyltransferase, GNAT family [Lactobacillus curvatus CRL 705]
GKJWQY101A4OKZ	K00097	4-hydroxythreonine-4-phosphate dehydrogenase [EC:1.1.1.262]	Vitamin B6 metabolism	234	6	233	8.0E-44	98%	352106048	Bacteria	Proteobacteria	Gammaproteobacteria	4-hydroxythreonine-4-phosphate dehydrogenase [Halomonas sp. HAL1]
GKJWQY101AYM89	K00791	tRNA dimethylallyltransferase [EC:2.5.1.75]	Zeatin biosynthesis	167	161	6	3.0E-19	90%	332876550	Bacteria	Bacteroidetes	Flavobacteriia	tRNA dimethylallyltransferase [Capnocytophaga sp. oral taxon 329 str. F0087]
GKJWQY101BQ2U_2	K01119	2',3'-cyclic-nucleotide 2'-phosphodiesterase [EC:3.1.4.16]	Purine metabolism/Pyrimidine metabolism	249	242	3	1.0E-35	90%	359790610	Bacteria	Proteobacteria	Alphaproteobacteria	bifunctional 2',3'-cyclic nucleotide 2'-phosphodiesterase/3'-nucleotidase periplasmic precursor protein [Mesorhizobium alhagi CCNWXJ12-2]
