# Supplementary material for: Subglacial Lake Vostok (Antarctica) Accretion Ice Contains a Diverse Set of Sequences from Aquatic, Marine and Sediment-Inhabiting Bacteria and Eukarya
Source: PLoS One. 2013 Jul 3;8(7):e67221. doi: 10.1371/journal.pone.0067221 (PMC3700977; doi:10.1371/journal.pone.0067221)
Supplement: Table S12 — Blastn and Blastx results from analysis of V6 sequences on the KAAS KEGG site [25] . The searches were for highly similar sequences (megablast); Max. target sequences = 100; Expected threshold = 1e-10 (unless no results were found, then 0); filter low complexity regions and translated nucleotide search over Reference sequence protein database; Matrix - BLOSUM62; Scoring parameters (existence 11; extension 1); filter low complexity regions. [“n” indicates information not specified in the NCBI GenBank database.]. (PDF) [file pone.0067221.s017.pdf]

Table S12. Blastn and Blastx results from analysis of V6 sequences on the KAAS KEGG site [25]. The searches were for highly similar sequences (megablast); Max. target sequences = 100; Expected threshold = 1e-10 (unless no results were found, then 0); filter low complexity regions and translated nucleotide search over Reference sequence protein database; Matrix - BLOSUM62; Scoring parameters (existence 11; extension 1); filter low complexity regions. ["n" indicates information not specified in the NCBI GenBank database.]

454 Sequence ID	Orthology #	KAAS KEGG Enzyme name	Pathways Names	Q length	Q start	Q end	e-value	%-ident	GI number	Domain	Phyla	Class / Order	Description
GJDB4OT01BSP4O	K01903	succinyl-CoA synthetase beta subunit [EC:6.2.1.5]	Citrate cycle (TCA cycle)/Propanoate metabolism/C5-Branched dibasic acid metabolism/Carbon fixation pathways in prokaryotes	176	1	174	1.00E-76	97%	299065054	Bacteria	Proteobacteria	Betaproteobacteria	Ralstonia solanacearum str. CMR15 chromosome, complete genome, product = succinate-CoA ligase (ADP-forming) beta subunit
GJDB4OT01BKJYT	K01154	type I restriction enzyme, S subunit [EC:3.1.21.3]	Endodeoxyribonucleases	122	1	122	3.00E-51	98%	407894523	Bacteria	Proteobacteria	Betaproteobacteria	Acidovorax sp. KKS102, complete genome, product = restriction modification system DNA specificity subunit
GJDB4OT01ARL6R	K00928	aspartate kinase [EC:2.7.2.4]	Glycine, serine and threonine metabolism/Cysteine and methionine metabolism/Lysine biosynthesis	280	1	280	4.00E-129	97%	387575654	Bacteria	Proteobacteria	Betaproteobacteria	Burkholderia sp. KJ006 chromosome 1, complete sequence, product = aspartokinase
GJDB4OT01A4BOH	K03933	chitin-binding protein	n/a	214	1	214	5.00E-82	93%	77964193	Bacteria	Proteobacteria	Betaproteobacteria	Burkholderia sp. 383 chromosome 3, complete sequence, product = chitin-binding protein
GJDB4OT01AZQW8	K06891	ATP-dependent Clp protease adaptor protein ClpS	n/a	106	1	106	2.00E-33	92%	334194119	Bacteria	Proteobacteria	Betaproteobacteria	Ralstonia solanacearum Po82, complete genome, product = atp-dependent clp protease adaptor protein clps
GJDB4OT01BVZ09	K07486	Transposase and inactivated derivatives	n/a	202	1	202	8.00E-31	79%	387580705	Bacteria	Proteobacteria	Betaproteobacteria	Burkholderia sp. KJ006 chromosome 3, complete sequence, product = transposase
GJDB4OT01BOSSW	K03702	excinuclease ABC subunit B	Nucleotide excision repair	252	1	252	3.00E-109	96%	133737197	Bacteria	Proteobacteria	Betaproteobacteria	Herminiimonas arsenicoxydans chromosome, complete sequence, product = UvrABC system protein B (Protein uvrB)(Excinuclease ABC subunit B)
GJDB4OT01BUG3G	K02275	cytochrome c oxidase subunit II [EC:1.9.3.1]	Oxidative phosphorylation	165	1	165	1.00E-69	97%	387575654	Bacteria	Proteobacteria	Betaproteobacteria	Burkholderia sp. KJ006 chromosome 1, complete sequence, product = cytochrome c oxidase polypeptide II
GJDB4OT01BUG3G_2	K02275	cytochrome c oxidase subunit II [EC:1.9.3.1]	Oxidative phosphorylation	106	1	106	2.00E-47	100%	387575654	Bacteria	Proteobacteria	Betaproteobacteria	Burkholderia sp. KJ006 chromosome 1, complete sequence, product = cytochrome c oxidase polypeptide II
GJDB4OT01AOX6C	K02298	cytochrome o ubiquinol oxidase subunit I [EC:1.10.3.-]	Oxidative phosphorylation	181	1	181	1.00E-83	98%	387575654	Bacteria	Proteobacteria	Betaproteobacteria	Burkholderia sp. KJ006 chromosome 1, complete sequence, product = cytochrome O ubiquinol oxidase subunit I
GJDB4OT01BBC3K	K00615	transketolase [EC:2.2.1.1]	Pentose phosphate pathway/Carbon fixation in photosynthetic organisms/Biosynthesis of ansamycins	238	1	238	1.00E-117	99%	356871503	Eukaryota	Ascomycota	Saccharomycetes	Pichia sorbitophila strain CBS 7064 chromosome J complete sequence, product = Piso0_002788 (Transketolase similar to Tkl2p)
GJDB4OT01B2XJX	K01485	cytosine deaminase [EC:3.5.4.1]	Pyrimidine metabolism/Arginine and proline metabolism	138	1	138	7.00E-45	91%	30407127	Bacteria	Proteobacteria	Betaproteobacteria	Ralstonia solanacearum GMI1000 chromosome complete sequence, product = probable cytosine deaminase (cytosine aminohydrolase) protein
GJDB4OT01BHOMH	K02892	large subunit ribosomal protein L23	Ribosome	271	1	271	2.00E-131	99%	387575654	Bacteria	Proteobacteria	Betaproteobacteria	Burkholderia sp. KJ006 chromosome 1, complete sequence, product = LSU ribosomal protein L4p (L1e) (<1..28); LSU ribosomal protein L23p (L23Ae) (25..>269)
GJDB4OT01AZ705	K03566	LysR family transcriptional regulator, glycine cleavage system transcriptional activator	Transcription factors	215	1	215	1.00E-77	92%	187713229	Bacteria	Proteobacteria	Betaproteobacteria	Burkholderia phytofirmans PsJN chromosome 1, complete sequence, product = transcriptional regulator, LysR family
GJDB4OT01AOVN9	K01533	Cu2+-exporting ATPase [EC:3.6.3.4]	Acting on acid anhydrides to catalyse transmembrane movement of substances	174	1	174	1.00E-16	79%	209517887	Bacteria	Proteobacteria	Betaproteobacteria	heavy metal translocating P-type ATPase [Burkholderia sp. H160]
GJDB4OT01AHOQ9	K03818	putative colanic acid biosynthesis acetyltransferase WcaF [EC:2.3.1.-]	Acyltransferases	228	226	2	5.00E-26	75%	311107671	Bacteria	Proteobacteria	Betaproteobacteria	colanic acid biosynthesis acetyltransferase WcaF [Achromobacter xylosoxidans A8]
GJDB4OT01A77FL	K01791	UDP-N-acetylglucosamine 2-epimerase [EC:5.1.3.14]	Amino sugar and nucleotide sugar metabolism	270	259	8	6.00E-32	77%	300313505	Bacteria	Proteobacteria	Betaproteobacteria	UDP-N-acetylglucosamine 2-epimerase [Herbaspirillum seropedicae SmR1]
GJDB4OT01ALI0S	K01919	glutamate--cysteine ligase [EC:6.3.2.2]	Glutathione metabolism	174	1	174	7.00E-19	81%	319764755	Bacteria	Proteobacteria	Betaproteobacteria	glutamate/cysteine ligase [Alicycliphilus denitrificans BC]
GJDB4OT01BVG95	K07008	Predicted glutamine amidotransferase	Histidine metabolism	190	1	189	4.00E-32	96%	351728124	Bacteria	Proteobacteria	Betaproteobacteria	glutamine amidotransferase class-II, partial [Acidovorax radicis N35]
GJDB4OT01A179C	K06942	Predicted GTPase, probable translation factor	n/a	122	121	2	2.00E-19	100%	223043362	Bacteria	Firmicutes	Bacilli	GTP-binding protein YchF [Staphylococcus capitis SK14]
GJDB4OT01AO7LF	K02259	cytochrome c oxidase assembly protein subunit 15	Oxidative phosphorylation/Porphyrin and chlorophyll metabolism/Two-component system	171	1	171	1.00E-23	91%	418245854	Bacteria	Actinobacteria	Actinobacteria	cytochrome c oxidase subunit XV assembly protein [Corynebacterium glutamicum ATCC 14067]
GJDB4OT01ACOWR	K08483	phosphotransferase system, enzyme I, PtsI [EC:2.7.3.9]	Phosphotransferase system	186	1	186	3.00E-31	95%	149926016	Bacteria	Proteobacteria	Betaproteobacteria	Phosphoenolpyruvate-protein phosphotransferase [Limnobacter sp. MED105]
GJDB4OT01BQTBQ	K07799	putative multidrug efflux transporter MdtA	Two-component system	259	3	254	1.00E-21	74%	417708018	Bacteria	Proteobacteria	Gammaproteobacteria	efflux transporter, RND family, MFP subunit [Shigella flexneri VA-6]
