# Supplementary material for: Subglacial Lake Vostok (Antarctica) Accretion Ice Contains a Diverse Set of Sequences from Aquatic, Marine and Sediment-Inhabiting Bacteria and Eukarya
Source: PLoS One. 2013 Jul 3;8(7):e67221. doi: 10.1371/journal.pone.0067221 (PMC3700977; doi:10.1371/journal.pone.0067221)
Supplement: Table S13 — Gene sequences found that support processes in Figure 3 . (PDF) [file pone.0067221.s018.pdf]

Table S13. Gene sequences found that support processes in Figure 3.

Nitrogen Metabolism

Most Similar Gene	Most Similar Taxon	GI Number	Percent Identity
asparagine synthase (glutamine-hydrolyzing)	*Bacillus* sp. 10403023	403236697	72%
ferredoxin-nitrate reductase	*Oscillatoria nigro-viridis* PCC 7112	428238862	97%
FMN-dependent NADH-azoreductase	*Lactobacillus rhamnosus* ATCC 8530	355393429	99%
glutamate dehydrogenase	*Bacteroides coprocola* DSM 17136	189431537	100%
NADH dehydrogenase I subunit E	*Burkholderia* sp. KJ006	387575654	98%
NADH dehydrogenase I subunit M	*Oscillatoria nigro-viridis* PCC 7112	428238862	92%
nitrate reductase 1, alpha subunit	*Acinetobacter junii* SH205	262374579	98%
nitrate reductase 1, alpha subunit	*Roseobacter* sp. MED193	86137746	83%
putative pyruvate-flavodoxin oxidoreductase	*Bacteroides thetaiotaomicron* VPI-5482	29342101	87%
ubiquinol-cytochrome c reductase cytochrome b subunit	*Burkholderia* sp. 383	77965403	98%

Carbon Fixation

Most Similar Gene	Most Similar Taxon	GI Number	Percent Identity
aconitate hydratase 1	*Planococcus antarcticus* DSM 14505	389820589	99%
3-hydroxyacyl-CoA dehydrogenase	*Mesorhizobium alhagi* CCNWXJ12-2	359790820	79%
isocitrate dehydrogenase	*Staphylococcus saprophyticus* subsp. *saprophyticus* ATCC 15305	72493824	76%
isocitrate dehydrogenase	*Burkholderia* sp. KJ006	387575654	99%
malate dehydrogenase	*Lactobacillus casei* ATCC 334	116103724	99%
2-oxoglutarate dehydrogenase E2 component	*Methylibium petroleiphilum* PM1	124267201	100%
phosphoenolpyruvate carboxykinase	*Bacteroides fragilis* 638R	301161079	87%
phosphoribulokinase	*Oscillatoria nigro-viridis* PCC 711	428238862	99%
putative pyruvate-flavodoxin oxidoreductase	*Bacteroides thetaiotaomicron* VPI-5482	29342101	87%
pyruvate dehydrogenase E2 component	*Burkholderia* sp. KJ006	387575654	97%
pyruvate dehydrogenase E1 component	*Burkholderia* sp. KJ006	387575654	99%
pyruvate, water dikinase	*Burkholderia* sp. KJ006	387575654	99%
succinyl-CoA synthetase alpha subunit	*Chitinophaga pinensis* DSM 2588	256420259	87%

Other Processes

Most Similar Gene	Most Similar Taxon	GI Number	Percent Identity
cysteine synthase A	*Lactobacillus rhamnosus* Lc 705	257152781	99%
MFS transporter, FSR family, fosmidomycin resistance protein	*Paracoccus denitrificans* PD1222	119372524	76%
molybdenum cofactor biosynthesis protein	*Oscillatoria nigro-viridis* PCC 7112	428238862	96%
sulfite reductase (NADPH) hemoprotein beta-component	*Burkholderia* sp. KJ00	387575654	99%

Transporters (important for the above processes)

Most Similar Gene	Most Similar Taxon	GI Number	Percent Identity
arsenite-transporting ATPase	*Oceanobacillus* sp. Ndiop	403071345	87%
ATP-binding cassette, subfamily B, bacterial MsbA	*Oscillatoria nigro-viridis* PCC 7112	428238862	97%
iron(III) transport system ATP-binding protein	*Oscillatoria nigro-viridis* PCC 7112	428238862	95%
putative ABC transport system ATP-binding protein	*Sphingomonas* sp. SKA58	94498321	74%
putrescine transport system substrate-binding protein	*Burkholderia* sp. KJ006	387575654	99%
mechanosensitive ion channel	*Oscillatoria nigro-viridis* PCC 7112	428238862	87%
spermidine/putrescine transport system permease protein	*Oscillatoria nigro-viridis* PCC 7112	428238862	95%
sulfate transport system ATP-binding protein	*Oscillatoria nigro-viridis* PCC 7112	428238862	96%
urea transport system substrate-binding protein	*Oscillatoria nigro-viridis* PCC 7112	428238862	92%
