# Supplementary material for: Subglacial Lake Vostok (Antarctica) Accretion Ice Contains a Diverse Set of Sequences from Aquatic, Marine and Sediment-Inhabiting Bacteria and Eukarya
Source: PLoS One. 2013 Jul 3;8(7):e67221. doi: 10.1371/journal.pone.0067221 (PMC3700977; doi:10.1371/journal.pone.0067221)
Supplement: Table S14 — Sequences removed from the V5 data set that were identical or similar to sequence from controls. [“n” indicates information not specified in the NCBI GenBank database.]. (PDF) [file pone.0067221.s019.pdf]

**Table S14. Sequences removed from the V5 data set that were identical or similar to sequence from controls. ["n" indicates information not specified in the NCBI GenBank database.]**

| Accession number / 454 sequence ID | Q length | Q start | Q end | e-value | %-ident | %-sim | GI number | Domain | Phylum | Family | Genus / Species |
|---|---|---|---|---|---|---|---|---|---|---|---|
| JQ997204 | 550 | 17 | 547 | 0 | 90% | 90% | 262527504 | Bacteria | Actinobacteria | Intrasporangiaceae | Janibacter sp. N2M |
| JQ997210 | 558 | 14 | 446 | 0 | 100% | 100% | 14252975 | Bacteria | Actinobacteria | Microbacteriaceae | Clavibacter michiganensis |
| JQ999635 | 554 | 18 | 508 | 0 | 95% | 95% | 95117795 | Bacteria | Actinobacteria | Microbacteriaceae | Clavibacter michiganensis |
| JQ997209 | 542 | 5 | 535 | 0 | 94% | 94% | 254728761 | Bacteria | Actinobacteria | Microbacteriaceae | Clavibacter michiganensis |
| GKJWQY101ABFJ2 | 584 | 18 | 583 | 0 | 95% | 95% | 147829108 | Bacteria | Actinobacteria | Microbacteriaceae | Clavibacter michiganensis |
| GKJWQY101BBWIZ | 602 | 7 | 594 | 0 | 92% | 92% | 169155030 | Bacteria | Actinobacteria | Microbacteriaceae | Clavibacter michiganensis |
| GKJWQY101BOGGY | 580 | 18 | 576 | 0 | 94% | 94% | 227452846 | Bacteria | Actinobacteria | Corynebacteriaceae | Corynebacterium aurimucosum |
| GKJWQY101BNKEW | 560 | 16 | 500 | 5E-164 | 89% | 89% | 47118314 | Bacteria | Actinobacteria | Corynebacteriaceae | Corynebacterium efficiens |
| GKJWQY101AS6C1 | 579 | 17 | 578 | 0 | 89% | 89% | 219857661 | Bacteria | Actinobacteria | Micrococcaceae | Arthrobacter chlorophenolicus |
| GKJWQY101AGV6J | 570 | 5 | 567 | 0 | 89% | 89% | 116608677 | Bacteria | Actinobacteria | Micrococcaceae | Arthrobacter sp. FB24 |
| JQ997264 | 554 | 5 | 554 | 0 | 99% | 99% | 157703991 | Bacteria | Actinobacteria | Micrococcaceae | Micrococcus sp. SY-13 |
| JQ997244 | 536 | 5 | 376 | 0 | 99% | 99% | 53986985 | Bacteria | Actinobacteria | Micrococcaceae | Micrococcus luteus |
| JQ997241 | 378 | 5 | 324 | 1E-158 | 99% | 99% | 108745470 | Bacteria | Actinobacteria | Micrococcaceae | Micrococcus luteus |
| JQ997249 | 561 | 5 | 558 | 0 | 91% | 91% | 121078653 | Bacteria | Actinobacteria | Micrococcaceae | Micrococcus luteus |
| JQ997246 | 552 | 15 | 532 | 0 | 95% | 95% | 209875195 | Bacteria | Actinobacteria | Micrococcaceae | Micrococcus luteus |
| JQ997247 | 558 | 156 | 491 | 5E-124 | 92% | 92% | 219523974 | Bacteria | Actinobacteria | Micrococcaceae | Micrococcus luteus |
| JQ997248 | 559 | 5 | 554 | 0 | 100% | 100% | 219809053 | Bacteria | Actinobacteria | Micrococcaceae | Micrococcus luteus |
| JQ997242 | 395 | 14 | 362 | 1E-178 | 99% | 99% | 255683811 | Bacteria | Actinobacteria | Micrococcaceae | Micrococcus luteus |
| JQ997250 | 581 | 18 | 562 | 0 | 92% | 92% | 284010036 | Bacteria | Actinobacteria | Micrococcaceae | Micrococcus luteus |
| JQ997243 | 533 | 45 | 531 | 0 | 95% | 95% | 291170385 | Bacteria | Actinobacteria | Micrococcaceae | Micrococcus luteus |
| JQ997245 | 549 | 18 | 548 | 0 | 97% | 97% | 295027175 | Bacteria | Actinobacteria | Micrococcaceae | Micrococcus luteus |
| GKJWQY101ANDPA | 579 | 7 | 578 | 0 | 96% | 96% | 239837778 | Bacteria | Actinobacteria | Micrococcaceae | Micrococcus luteus |
| GKJWQY101BB4KB | 552 | 2 | 534 | 0 | 92% | 92% | 283133067 | Bacteria | Actinobacteria | Micrococcaceae | Rothia mucilaginosa |
| JQ997271 | 545 | 21 | 542 | 0 | 100% | 100% | 290759827 | Bacteria | Actinobacteria | Micrococcaceae | Rothia mucilaginosa |
| GKJWQY101AYY7N | 514 | 17 | 463 | 1E-149 | 89% | 89% | 145213092 | Bacteria | Actinobacteria | Mycobacteriaceae | Mycobacterium gilvum |
| GKJWQY101AHIKR | 502 | 14 | 449 | 3E-81 | 80% | 80% | 126232413 | Bacteria | Actinobacteria | Mycobacteriaceae | Mycobacterium sp. JLS |
| GKJWQY101BW78O | 526 | 17 | 230 | 6E-83 | 93% | 93% | 258553496 | Bacteria | Actinobacteria | Nakamurellaceae | Nakamurella multipartita |
| GKJWQY101BNTM3 | 401 | 18 | 370 | 9E-145 | 93% | 93% | 255918463 | Bacteria | Actinobacteria | Actinosynnemataceae | Actinosynnema mirum |
| GKJWQY101B0WYX | 571 | 17 | 571 | 0 | 88% | 88% | 229564415 | Bacteria | Actinobacteria | Beutenbergiaceae | Beutenbergia cavernae |
| GKJWQY101BWYS7 | 570 | 16 | 565 | 0 | 94% | 94% | 256558041 | Bacteria | Actinobacteria | Dermabacteraceae | Brachybacterium faecium |
| GKJWQY101BID96 | 589 | 24 | 584 | 0 | 93% | 93% | 284061874 | Bacteria | Actinobacteria | Geodermatophilaceae | Geodermatophilus obscurus |
| GKJWQY101B0W1Z | 560 | 18 | 553 | 0 | 89% | 89% | 262083393 | Bacteria | Actinobacteria | Gordoniaceae | Gordonia bronchialis |
| GKJWQY101AS57B | 556 | 5 | 548 | 0 | 89% | 89% | 196121877 | Bacteria | Actinobacteria | Kineosporiaceae | Kineococcus radiotolerans |
| GKJWQY101BURT9 | 573 | 18 | 561 | 0 | 90% | 90% | 226237899 | Bacteria | Actinobacteria | Nocardiaceae | Rhodococcus opacus |
| GKJWQY101AL5D7 | 537 | 17 | 483 | 0 | 97% | 97% | 119534933 | Bacteria | Actinobacteria | Nocardiaceae | Nocardioides sp. JS614 |
| GKJWQY101BJOST | 547 | 8 | 546 | 0 | 98% | 98% | 50839098 | Bacteria | Actinobacteria | Propionibacteriaceae | Propionibacterium acnes |
| GKJWQY101BQS08 | 598 | 4 | 584 | 0 | 95% | 95% | 291375051 | Bacteria | Actinobacteria | Propionibacteriaceae | Propionibacterium acnes |
| GKJWQY101BYVMH | 599 | 5 | 363 | 1E-114 | 88% | 88% | 270504784 | Bacteria | Actinobacteria | Streptosporangiaceae | Streptosporangium roseum |
| GKJWQY101A0TH4 | 586 | 17 | 583 | 0 | 95% | 95% | 257472321 | Bacteria | Actinobacteria | Coriobacteriaceae | Atopobium parvulum |
| GKJWQY101A3ZEU | 584 | 18 | 577 | 1E-160 | 86% | 86% | 256033965 | Bacteria | Bacteroidetes | Chitinophagaceae | Chitinophaga pinensis |
| JQ997450 | 586 | 5 | 346 | 1E-164 | 98% | 98% | 13774313 | Bacteria | Cyanobacteria | Phormidiaceae | Microcoleus vaginatus |
| JQ997424 | 341 | 16 | 294 | 1E-127 | 97% | 97% | 13774314 | Bacteria | Cyanobacteria | Phormidiaceae | Microcoleus vaginatus |
| JQ997437 | 542 | 5 | 487 | 0 | 100% | 100% | 19070784 | Bacteria | Cyanobacteria | Phormidiaceae | Microcoleus vaginatus |
| JQ997442 | 549 | 18 | 543 | 0 | 93% | 93% | 19070787 | Bacteria | Cyanobacteria | Phormidiaceae | Microcoleus vaginatus |
| JQ997445 | 555 | 17 | 552 | 0 | 100% | 100% | 19070788 | Bacteria | Cyanobacteria | Phormidiaceae | Microcoleus vaginatus |
| JQ997444 | 553 | 1 | 500 | 0 | 96% | 96% | 19070791 | Bacteria | Cyanobacteria | Phormidiaceae | Microcoleus vaginatus |
| JQ997421 | 275 | 5 | 242 | 4E-87 | 92% | 92% | 19070794 | Bacteria | Cyanobacteria | Phormidiaceae | Microcoleus vaginatus |
| JQ997433 | 482 | 18 | 434 | 0 | 95% | 95% | 19070795 | Bacteria | Cyanobacteria | Phormidiaceae | Microcoleus vaginatus |
| JQ997425 | 361 | 19 | 292 | 1E-138 | 100% | 100% | 19070798 | Bacteria | Cyanobacteria | Phormidiaceae | Microcoleus vaginatus |
| JQ997438 | 542 | 12 | 539 | 0 | 94% | 94% | 19070799 | Bacteria | Cyanobacteria | Phormidiaceae | Microcoleus vaginatus |

| | | | | | | | | | | | |
|---|---|---|---|---|---|---|---|---|---|---|---|
| JQ997428 | 429 | 3 | 386 | 0 | 99% | 99% | 19070802 | Bacteria | Cyanobacteria | Phormidiaceae | Microcoleus vaginatus |
| JQ997431 | 445 | 18 | 410 | 0 | 99% | 99% | 19070804 | Bacteria | Cyanobacteria | Phormidiaceae | Microcoleus vaginatus |
| JQ997452 | 693 | 17 | 349 | 3E-152 | 97% | 97% | 19070806 | Bacteria | Cyanobacteria | Phormidiaceae | Microcoleus vaginatus |
| JQ997430 | 430 | 5 | 388 | 1E-124 | 89% | 89% | 19070807 | Bacteria | Cyanobacteria | Phormidiaceae | Microcoleus vaginatus |
| JQ997439 | 544 | 20 | 522 | 0 | 92% | 92% | 19070809 | Bacteria | Cyanobacteria | Phormidiaceae | Microcoleus vaginatus |
| JQ997432 | 464 | 18 | 417 | 0 | 96% | 96% | 19070810 | Bacteria | Cyanobacteria | Phormidiaceae | Microcoleus vaginatus |
| JQ997420 | 267 | 5 | 230 | 5E-96 | 95% | 95% | 19070813 | Bacteria | Cyanobacteria | Phormidiaceae | Microcoleus vaginatus |
| JQ997447 | 560 | 5 | 196 | 5E-89 | 98% | 98% | 19070814 | Bacteria | Cyanobacteria | Phormidiaceae | Microcoleus vaginatus |
| JQ997429 | 429 | 3 | 324 | 7E-146 | 96% | 96% | 19070815 | Bacteria | Cyanobacteria | Phormidiaceae | Microcoleus vaginatus |
| JQ997422 | 313 | 3 | 266 | 1E-132 | 100% | 100% | 19070816 | Bacteria | Cyanobacteria | Phormidiaceae | Microcoleus vaginatus |
| JQ997440 | 546 | 5 | 543 | 0 | 96% | 96% | 19070817 | Bacteria | Cyanobacteria | Phormidiaceae | Microcoleus vaginatus |
| JQ997423 | 330 | 18 | 306 | 2E-145 | 99% | 99% | 19070818 | Bacteria | Cyanobacteria | Phormidiaceae | Microcoleus vaginatus |
| JQ997427 | 413 | 7 | 364 | 2E-141 | 93% | 93% | 19070819 | Bacteria | Cyanobacteria | Phormidiaceae | Microcoleus vaginatus |
| JQ997441 | 548 | 5 | 543 | 0 | 94% | 94% | 19070820 | Bacteria | Cyanobacteria | Phormidiaceae | Microcoleus vaginatus |
| JQ997426 | 368 | 18 | 331 | 8E-160 | 99% | 99% | 57996791 | Bacteria | Cyanobacteria | Phormidiaceae | Microcoleus vaginatus |
| JQ997435 | 536 | 5 | 508 | 0 | 99% | 99% | 57996792 | Bacteria | Cyanobacteria | Phormidiaceae | Microcoleus vaginatus |
| JQ997451 | 610 | 24 | 398 | 2E-163 | 95% | 95% | 149364148 | Bacteria | Cyanobacteria | Phormidiaceae | Microcoleus vaginatus |
| JQ997446 | 556 | 5 | 553 | 0 | 98% | 98% | 149364157 | Bacteria | Cyanobacteria | Phormidiaceae | Microcoleus vaginatus |
| JQ997436 | 538 | 23 | 537 | 0 | 93% | 93% | 149364161 | Bacteria | Cyanobacteria | Phormidiaceae | Microcoleus vaginatus |
| JQ997448 | 560 | 5 | 560 | 0 | 94% | 94% | 149364212 | Bacteria | Cyanobacteria | Phormidiaceae | Microcoleus vaginatus |
| JQ997449 | 564 | 4 | 561 | 0 | 94% | 94% | 171474880 | Bacteria | Cyanobacteria | Phormidiaceae | Microcoleus vaginatus |
| JQ997434 | 524 | 5 | 385 | 0 | 98% | 98% | 260534371 | Bacteria | Cyanobacteria | Phormidiaceae | Microcoleus vaginatus |
| GKJWQY101AJSLD | 166 | 5 | 151 | 8E-67 | 99% | 99% | 19070792 | Bacteria | Cyanobacteria | Phormidiaceae | Microcoleus vaginatus |
| GKJWQY101ALAE0 | 352 | 84 | 244 | 2E-71 | 98% | 98% | 19070793 | Bacteria | Cyanobacteria | Phormidiaceae | Microcoleus vaginatus |
| GKJWQY101BAG81 | 118 | 3 | 106 | 2E-46 | 100% | 100% | 19070796 | Bacteria | Cyanobacteria | Phormidiaceae | Microcoleus vaginatus |
| GKJWQY101BPKV7 | 194 | 20 | 163 | 1E-66 | 99% | 99% | 19070797 | Bacteria | Cyanobacteria | Phormidiaceae | Microcoleus vaginatus |
| GKJWQY101BSRBZ | 199 | 18 | 169 | 4E-71 | 99% | 99% | 19070801 | Bacteria | Cyanobacteria | Phormidiaceae | Microcoleus vaginatus |
| GKJWQY101BYSFG | 120 | 14 | 88 | 1E-28 | 99% | 99% | 19070808 | Bacteria | Cyanobacteria | Phormidiaceae | Microcoleus vaginatus |
| GKJWQY101A7XVF | 188 | 5 | 144 | 3E-66 | 100% | 100% | 149364147 | Bacteria | Cyanobacteria | Phormidiaceae | Microcoleus vaginatus |
| JQ997463 | 552 | 21 | 549 | 0 | 93% | 93% | 167508130 | Bacteria | Cyanobacteria | Phormidiaceae | Phormidium amoenum |
| JQ998746 | 238 | 13 | 238 | 2E-105 | 98% | 98% | 46409893 | Bacteria | Cyanobacteria | Oscillatoriaceae | Oscillatoria |
| JQ997394 | 642 | 5 | 635 | 0 | 91% | 91% | 225696244 | Bacteria | Cyanobacteria | n | uncultured cyanobacterium |
| GKJWQY101A8PWD_2 | 97 | 1 | 97 | 1E-42 | 100% | 100% | 225696243 | Bacteria | Cyanobacteria | n | uncultured cyanobacterium |
| GKJWQY101A8PV9 | 556 | 5 | 555 | 0 | 91% | 91% | 212559329 | Bacteria | Firmicutes | Bacillaceae | Anoxybacillus flavithermus |
| GKJWQY101AEF0G | 232 | 5 | 135 | 4E-61 | 100% | 100% | 294987211 | Bacteria | Firmicutes | Bacillaceae | Anoxybacillus flavithermus |
| GKJWQY101A52X2 | 566 | 10 | 522 | 0 | 93% | 93% | 239805877 | Bacteria | Firmicutes | Bacillaceae | Geobacillus sp. WCH70 |
| GKJWQY101BMIJM | 565 | 1 | 560 | 0 | 95% | 95% | 168990106 | Bacteria | Firmicutes | Bacillaceae | Lysinibacillus sphaericus |
| GKJWQY101A1C8O | 551 | 23 | 498 | 0 | 92% | 92% | 291482254 | Bacteria | Firmicutes | Clostridiaceae | Clostridium difficile |
| GKJWQY101BEI1M | 547 | 24 | 521 | 0 | 93% | 93% | 291482099 | Bacteria | Firmicutes | Clostridiaceae | Clostridium difficile |
| GKJWQY101BG2WT | 586 | 5 | 580 | 0 | 92% | 92% | 291482100 | Bacteria | Firmicutes | Clostridiaceae | Clostridium difficile |
| GKJWQY101BAWHO | 552 | 4 | 531 | 0 | 91% | 91% | 291482251 | Bacteria | Firmicutes | Clostridiaceae | Clostridium difficile |
| GKJWQY101B14ZL | 587 | 5 | 585 | 0 | 90% | 90% | 291482250 | Bacteria | Firmicutes | Clostridiaceae | Clostridium difficile |
| GKJWQY101A7XRP | 573 | 18 | 570 | 0 | 92% | 92% | 115249003 | Bacteria | Firmicutes | Clostridiaceae | Clostridium difficile |
| GKJWQY101AQWZZ | 574 | 18 | 571 | 0 | 89% | 89% | 295102939 | Bacteria | Firmicutes | Clostridiaceae | Faecalibacterium prausnitzii |
| GKJWQY101AGCFL | 593 | 5 | 590 | 0 | 91% | 91% | 291537741 | Bacteria | Firmicutes | Lachnospiraceae | Roseburia intestinalis |
| GKJWQY101AKNEB | 590 | 18 | 104 | 7E-28 | 94% | 94% | 260066140 | Bacteria | Firmicutes | Staphylococcaceae | Staphylococcus aureus |
| GKJWQY101A4B8O | 578 | 5 | 565 | 0 | 88% | 88% | 149944932 | Bacteria | Firmicutes | Staphylococcaceae | Staphylococcus aureus |
| GKJWQY101BM94T | 530 | 5 | 527 | 0 | 89% | 89% | 47118312 | Bacteria | Firmicutes | Staphylococcaceae | Staphylococcus aureus |
| GKJWQY101BOSAK | 552 | 5 | 552 | 0 | 97% | 97% | 9664721 | Bacteria | Firmicutes | Staphylococcaceae | Staphylococcus epidermidis |
| GKJWQY101BO8W7 | 564 | 17 | 560 | 0 | 99% | 99% | 9664737 | Bacteria | Firmicutes | Staphylococcaceae | Staphylococcus epidermidis |
| GKJWQY101AO1AU | 556 | 18 | 534 | 0 | 99% | 99% | 9664799 | Bacteria | Firmicutes | Staphylococcaceae | Staphylococcus epidermidis |
| GKJWQY101A7HX3 | 540 | 18 | 538 | 3E-180 | 89% | 89% | 9623643 | Bacteria | Firmicutes | Staphylococcaceae | Staphylococcus epidermidis |
| GKJWQY101ALP91 | 553 | 18 | 553 | 0 | 100% | 100% | 9624258 | Bacteria | Firmicutes | Staphylococcaceae | Staphylococcus epidermidis |
| GKJWQY101BUHRL | 539 | 33 | 440 | 6E-168 | 93% | 93% | 27316888 | Bacteria | Firmicutes | Staphylococcaceae | Staphylococcus epidermidis |
| GKJWQY101BOC3N | 562 | 24 | 533 | 0 | 97% | 97% | 9664635 | Bacteria | Firmicutes | Staphylococcaceae | Staphylococcus epidermidis |

| | | | | | | | | | | | |
|---|---|---|---|---|---|---|---|---|---|---|---|
| GKJWQY101BML37 | 564 | 17 | 530 | 0 | 99% | 99% | 9664791 | Bacteria | Firmicutes | Staphylococcaceae | Staphylococcus epidermidis |
| JQ997602 | 218 | 5 | 149 | 1E-45 | 91% | 91% | 223016892 | Bacteria | Firmicutes | Staphylococcaceae | Staphylococcus epidermidis |
| JQ999663 | 489 | 3 | 350 | 5E-178 | 99% | 99% | 31044171 | Bacteria | Firmicutes | Staphylococcaceae | Staphylococcus epidermidis |
| JQ999664 | 507 | 18 | 431 | 0 | 100% | 100% | 31044172 | Bacteria | Firmicutes | Staphylococcaceae | Staphylococcus epidermidis |
| JQ999662 | 437 | 5 | 393 | 0 | 99% | 99% | 31044173 | Bacteria | Firmicutes | Staphylococcaceae | Staphylococcus epidermidis |
| JQ999661 | 344 | 5 | 267 | 4E-128 | 99% | 99% | 213688819 | Bacteria | Firmicutes | Staphylococcaceae | Staphylococcus epidermidis |
| GKJWQY101BLWUI | 529 | 18 | 474 | 0 | 97% | 97% | 295029968 | Bacteria | Firmicutes | Lactobacillaceae | Lactobacillus crispatus |
| JQ997626 | 290 | 17 | 199 | 3E-53 | 89% | 89% | 285198791 | Bacteria | Firmicutes | Lactobacillaceae | Lactobacillus crispatus |
| GKJWQY101BFEY2 | 607 | 18 | 605 | 0 | 99% | 99% | 160347623 | Bacteria | Firmicutes | Lactobacillaceae | Lactobacillus helveticus |
| GKJWQY101A9I15 | 579 | 5 | 571 | 0 | 95% | 95% | 111610219 | Bacteria | Firmicutes | Lactobacillaceae | Lactobacillus helveticus |
| GKJWQY101AMEIU | 538 | 18 | 536 | 0 | 95% | 95% | 112148580 | Bacteria | Firmicutes | Lactobacillaceae | Lactobacillus helveticus |
| GKJWQY101ANUFG | 478 | 5 | 472 | 0 | 100% | 100% | 13398532 | Bacteria | Firmicutes | Lactobacillaceae | Lactobacillus helveticus |
| GKJWQY101AWVPF | 572 | 18 | 569 | 0 | 94% | 94% | 157272205 | Bacteria | Firmicutes | Lactobacillaceae | Lactobacillus helveticus |
| GKJWQY101AJJAJ | 390 | 18 | 357 | 5E-177 | 100% | 100% | 111610285 | Bacteria | Firmicutes | Lactobacillaceae | Lactobacillus helveticus |
| GKJWQY101BYWHX | 641 | 17 | 355 | 4E-140 | 94% | 94% | 111610140 | Bacteria | Firmicutes | Lactobacillaceae | Lactobacillus helveticus |
| GKJWQY101ATCEK | 553 | 1 | 549 | 0 | 100% | 100% | 3282340 | Bacteria | Firmicutes | Lactobacillaceae | Lactobacillus helveticus |
| GKJWQY101B1CMN | 543 | 4 | 542 | 0 | 98% | 98% | 112148551 | Bacteria | Firmicutes | Lactobacillaceae | Lactobacillus helveticus |
| GKJWQY101A41CM | 532 | 18 | 505 | 0 | 94% | 94% | 157272234 | Bacteria | Firmicutes | Lactobacillaceae | Lactobacillus helveticus |
| GKJWQY101ACW54 | 527 | 4 | 524 | 0 | 100% | 100% | 111610264 | Bacteria | Firmicutes | Lactobacillaceae | Lactobacillus helveticus |
| GKJWQY101ARFS8 | 557 | 18 | 357 | 7E-167 | 98% | 98% | 133917173 | Bacteria | Firmicutes | Lactobacillaceae | Lactobacillus helveticus |
| GKJWQY101BSMTE | 558 | 18 | 554 | 0 | 96% | 96% | 111610178 | Bacteria | Firmicutes | Lactobacillaceae | Lactobacillus helveticus |
| GKJWQY101AE8IV | 527 | 18 | 523 | 0 | 99% | 99% | 157272211 | Bacteria | Firmicutes | Lactobacillaceae | Lactobacillus helveticus |
| GKJWQY101AGVG5 | 509 | 19 | 503 | 0 | 97% | 97% | 2687734 | Bacteria | Firmicutes | Lactobacillaceae | Lactobacillus helveticus |
| GKJWQY101AD5HW | 559 | 78 | 517 | 0 | 98% | 98% | 111610125 | Bacteria | Firmicutes | Lactobacillaceae | Lactobacillus helveticus |
| GKJWQY101A4URK | 517 | 18 | 516 | 0 | 97% | 97% | 157272233 | Bacteria | Firmicutes | Lactobacillaceae | Lactobacillus helveticus |
| GKJWQY101BJRWH | 560 | 16 | 553 | 0 | 98% | 98% | 157272216 | Bacteria | Firmicutes | Lactobacillaceae | Lactobacillus helveticus |
| GKJWQY101BJ0YC | 532 | 5 | 460 | 0 | 100% | 100% | 111610270 | Bacteria | Firmicutes | Lactobacillaceae | Lactobacillus helveticus |
| JQ997639 | 332 | 17 | 288 | 4E-132 | 99% | 99% | 6537242 | Bacteria | Firmicutes | Lactobacillaceae | Lactobacillus helveticus |
| JQ997638 | 246 | 28 | 198 | 3E-73 | 97% | 97% | 30060365 | Bacteria | Firmicutes | Lactobacillaceae | Lactobacillus helveticus |
| JQ997643 | 428 | 17 | 390 | 0 | 100% | 100% | 53766373 | Bacteria | Firmicutes | Lactobacillaceae | Lactobacillus helveticus |
| JQ997650 | 554 | 15 | 538 | 0 | 91% | 91% | 57231848 | Bacteria | Firmicutes | Lactobacillaceae | Lactobacillus helveticus |
| JQ997641 | 391 | 18 | 339 | 2E-165 | 100% | 100% | 77681103 | Bacteria | Firmicutes | Lactobacillaceae | Lactobacillus helveticus |
| JQ997648 | 533 | 5 | 531 | 0 | 99% | 99% | 121581962 | Bacteria | Firmicutes | Lactobacillaceae | Lactobacillus helveticus |
| JQ997642 | 407 | 16 | 346 | 5E-157 | 97% | 97% | 168208498 | Bacteria | Firmicutes | Lactobacillaceae | Lactobacillus helveticus |
| JQ997645 | 526 | 23 | 522 | 0 | 99% | 99% | 224924260 | Bacteria | Firmicutes | Lactobacillaceae | Lactobacillus helveticus |
| JQ997646 | 528 | 21 | 522 | 0 | 99% | 99% | 225029237 | Bacteria | Firmicutes | Lactobacillaceae | Lactobacillus helveticus |
| JQ997640 | 370 | 18 | 342 | 2E-150 | 97% | 97% | 226815166 | Bacteria | Firmicutes | Lactobacillaceae | Lactobacillus helveticus |
| JQ997649 | 535 | 18 | 531 | 0 | 100% | 100% | 239586136 | Bacteria | Firmicutes | Lactobacillaceae | Lactobacillus helveticus |
| JQ997647 | 532 | 17 | 529 | 0 | 99% | 99% | 270513917 | Bacteria | Firmicutes | Lactobacillaceae | Lactobacillus helveticus |
| JQ997644 | 446 | 17 | 407 | 0 | 100% | 100% | 290784156 | Bacteria | Firmicutes | Lactobacillaceae | Lactobacillus helveticus |
| GKJWQY101B1IA6 | 116 | 3 | 44 | 5E-12 | 100% | 100% | 285801734 | Bacteria | Firmicutes | Streptococcaceae | Streptococcus mitis |
| GKJWQY101AKWUI | 575 | 16 | 574 | 0 | 91% | 91% | 288906474 | Bacteria | Firmicutes | Streptococcaceae | Streptococcus mitis |
| JQ997694 | 532 | 18 | 527 | 0 | 98% | 98% | 262286142 | Bacteria | Firmicutes | Streptococcaceae | Streptococcus mitis |
| JQ997717 | 387 | 17 | 353 | 3E-99 | 88% | 88% | 285195837 | Bacteria | Firmicutes | Streptococcaceae | Streptococcus sanguinis |
| GKJWQY101AODH5 | 587 | 5 | 587 | 0 | 95% | 95% | 125496804 | Bacteria | Firmicutes | Streptococcaceae | Streptococcus sanguinis |
| JQ997718 | 740 | 18 | 370 | 0 | 100% | 100% | 295002597 | Bacteria | Firmicutes | Streptococcaceae | Streptococcus sanguinis |
| JQ999700 | 546 | 5 | 546 | 0 | 99% | 99% | 45597365 | Bacteria | Firmicutes | Streptococcaceae | Streptococcus sanguinis |
| GKJWQY101BCX33 | 567 | 18 | 560 | 0 | 99% | 99% | 24473733 | Bacteria | Firmicutes | Streptococcaceae | Streptococcus thermophilus |
| GKJWQY101BGY7W | 543 | 24 | 521 | 0 | 94% | 94% | 154424882 | Bacteria | Firmicutes | Streptococcaceae | Streptococcus thermophilus |
| GKJWQY101BXHH4 | 536 | 5 | 483 | 0 | 100% | 100% | 46019822 | Bacteria | Firmicutes | Streptococcaceae | Streptococcus thermophilus |
| GKJWQY101ANZOM | 524 | 18 | 517 | 0 | 96% | 96% | 221047219 | Bacteria | Firmicutes | Streptococcaceae | Streptococcus thermophilus |
| GKJWQY101AG25G | 267 | 3 | 204 | 2E-100 | 100% | 100% | 284080586 | Bacteria | Firmicutes | Streptococcaceae | Streptococcus thermophilus |
| GKJWQY101BB72C | 419 | 4 | 354 | 1E-173 | 98% | 98% | 90655828 | Bacteria | Firmicutes | Streptococcaceae | Streptococcus thermophilus |
| GKJWQY101AWY8E | 592 | 27 | 306 | 2E-69 | 84% | 84% | 15485427 | Bacteria | Firmicutes | Streptococcaceae | Streptococcus thermophilus |
| GKJWQY101AY5Z9 | 543 | 18 | 540 | 0 | 99% | 99% | 6708106 | Bacteria | Firmicutes | Streptococcaceae | Streptococcus thermophilus |

| | | | | | | | | | | | |
|---|---|---|---|---|---|---|---|---|---|---|---|
| GKJWQY101AXEED | 594 | 18 | 590 | 0 | 98% | 98% | 55737978 | Bacteria | Firmicutes | Streptococcaceae | Streptococcus thermophilus |
| GKJWQY101A4EQD | 585 | 5 | 579 | 0 | 97% | 97% | 55736088 | Bacteria | Firmicutes | Streptococcaceae | Streptococcus thermophilus |
| GKJWQY101BO375 | 620 | 18 | 609 | 0 | 92% | 92% | 116100249 | Bacteria | Firmicutes | Streptococcaceae | Streptococcus thermophilus |
| JQ999701 | 524 | 17 | 440 | 0 | 100% | 100% | 288525 | Bacteria | Firmicutes | Streptococcaceae | Streptococcus thermophilus |
| JQ997733 | 530 | 18 | 492 | 0 | 95% | 95% | 152002890 | Bacteria | Firmicutes | Streptococcaceae | Streptococcus thermophilus |
| JQ997732 | 508 | 16 | 455 | 0 | 99% | 99% | 157400510 | Bacteria | Firmicutes | Streptococcaceae | Streptococcus thermophilus |
| JQ997730 | 434 | 18 | 309 | 3E-150 | 100% | 100% | 162945243 | Bacteria | Firmicutes | Streptococcaceae | Streptococcus thermophilus |
| JQ997728 | 368 | 21 | 313 | 6E-151 | 100% | 100% | 187475307 | Bacteria | Firmicutes | Streptococcaceae | Streptococcus thermophilus |
| JQ997734 | 542 | 17 | 541 | 0 | 100% | 100% | 225029151 | Bacteria | Firmicutes | Streptococcaceae | Streptococcus thermophilus |
| JQ997736 | 549 | 5 | 520 | 0 | 99% | 99% | 253720698 | Bacteria | Firmicutes | Streptococcaceae | Streptococcus thermophilus |
| JQ997731 | 491 | 17 | 430 | 0 | 100% | 100% | 254305414 | Bacteria | Firmicutes | Streptococcaceae | Streptococcus thermophilus |
| JQ997737 | 551 | 4 | 547 | 0 | 100% | 100% | 268619092 | Bacteria | Firmicutes | Streptococcaceae | Streptococcus thermophilus |
| JQ997729 | 425 | 9 | 376 | 0 | 99% | 99% | 285803102 | Bacteria | Firmicutes | Streptococcaceae | Streptococcus thermophilus |
| JQ997735 | 545 | 4 | 545 | 0 | 100% | 100% | 292673279 | Bacteria | Firmicutes | Streptococcaceae | Streptococcus thermophilus |
| JQ999660 | 571 | 6 | 561 | 0 | 89% | 89% | 296416 | Bacteria | Firmicutes | Planococcaceae | Sporosarcina globispora |
| GKJWQY101B0W4V | 543 | 341 | 488 | 9E-12 | 77% | 77% | 90103542 | Bacteria | Proteobacteria | Alphaproteobacteria | Rhodopseudomonas palustris |
| JQ999360 | 560 | 19 | 554 | 0 | 97% | 97% | 67527215 | Bacteria | Proteobacteria | Moraxellaceae | Acinetobacter baumannii |
| GKJWQY101AXN46 | 232 | 59 | 183 | 9E-53 | 98% | 98% | 168192641 | Bacteria | Proteobacteria | Methylobacteriaceae | Methylobacterium sp. 4-46 |
| GKJWQY101A3454 | 419 | 55 | 374 | 8E-41 | 77% | 77% | 159140696 | Bacteria | Proteobacteria | Rhizobiaceae | Agrobacterium tumefaciens |
| GKJWQY101BEXW8 | 522 | 288 | 501 | 8E-27 | 78% | 78% | 221721649 | Bacteria | Proteobacteria | Rhizobiaceae | Agrobacterium tumefaciens |
| GKJWQY101A44CM | 474 | 6 | 420 | 2E-88 | 81% | 81% | 115259848 | Bacteria | Proteobacteria | Rhizobiaceae | Rhizobium leguminosarum |
| GKJWQY101AA9UB | 528 | 33 | 448 | 2E-83 | 81% | 81% | 115254414 | Bacteria | Proteobacteria | Rhizobiaceae | Rhizobium leguminosarum |
| GKJWQY101BSXFZ | 528 | 349 | 470 | 5E-24 | 85% | 85% | 209533368 | Bacteria | Proteobacteria | Rhizobiaceae | Rhizobium leguminosarum |
| GKJWQY101A1E9L | 514 | 6 | 509 | 2E-148 | 86% | 86% | 240856645 | Bacteria | Proteobacteria | Rhizobiaceae | Rhizobium leguminosarum |
| JQ999799 | 581 | 18 | 577 | 0 | 90% | 90% | 2244633 | Bacteria | Proteobacteria | Caulobacteraceae | Brevundimonas diminuta |
| GKJWQY101A9F0H | 544 | 336 | 471 | 7E-23 | 82% | 82% | 295429362 | Bacteria | Proteobacteria | Caulobacteraceae | Caulobacter segnis |
| GKJWQY101A9VAR | 532 | 208 | 508 | 9E-37 | 77% | 77% | 84785911 | Bacteria | Proteobacteria | Erythrobacteraceae | Erythrobacter litoralis |
| GKJWQY101B2TF4 | 563 | 1 | 515 | 0 | 95% | 95% | 148498119 | Bacteria | Proteobacteria | Sphingomonadaceae | Sphingomonas wittichii |
| GKJWQY101BOJM6 | 364 | 106 | 331 | 1E-83 | 92% | 92% | 163258032 | Bacteria | Proteobacteria | Alcaligenaceae | Bordetella petrii |
| GKJWQY101BQ54I | 256 | 18 | 211 | 1E-92 | 99% | 99% | 171994659 | Bacteria | Proteobacteria | Burkholderiaceae | Burkholderia ambifaria |
| GKJWQY101BYI13 | 513 | 26 | 510 | 8E-97 | 81% | 81% | 115283258 | Bacteria | Proteobacteria | Burkholderiaceae | Burkholderia ambifaria |
| GKJWQY101BEI1O | 536 | 18 | 534 | 0 | 95% | 95% | 171998010 | Bacteria | Proteobacteria | Burkholderiaceae | Burkholderia ambifaria |
| GKJWQY101BRVHV | 506 | 3 | 491 | 0 | 91% | 91% | 115280044 | Bacteria | Proteobacteria | Burkholderiaceae | Burkholderia ambifaria |
| GKJWQY101A440F | 528 | 9 | 525 | 1E-159 | 87% | 87% | 190714216 | Bacteria | Proteobacteria | Burkholderiaceae | Burkholderia cenocepacia |
| GKJWQY101BPELL | 514 | 42 | 514 | 0 | 97% | 97% | 116652879 | Bacteria | Proteobacteria | Burkholderiaceae | Burkholderia cenocepacia |
| GKJWQY101BZA3A | 566 | 5 | 562 | 0 | 99% | 99% | 169820555 | Bacteria | Proteobacteria | Burkholderiaceae | Burkholderia cenocepacia |
| GKJWQY101BWFJ6 | 528 | 156 | 524 | 4E-159 | 95% | 95% | 116649273 | Bacteria | Proteobacteria | Burkholderiaceae | Burkholderia cenocepacia |
| GKJWQY101ALL88 | 537 | 32 | 498 | 1E-154 | 89% | 89% | 190714214 | Bacteria | Proteobacteria | Burkholderiaceae | Burkholderia cenocepacia |
| GKJWQY101A2LFA | 523 | 18 | 523 | 0 | 92% | 92% | 169814598 | Bacteria | Proteobacteria | Burkholderiaceae | Burkholderia cenocepacia |
| GKJWQY101AO2I9 | 584 | 18 | 580 | 0 | 93% | 93% | 190714218 | Bacteria | Proteobacteria | Burkholderiaceae | Burkholderia cenocepacia |
| GKJWQY101BL5T8 | 548 | 24 | 545 | 0 | 98% | 98% | 169817759 | Bacteria | Proteobacteria | Burkholderiaceae | Burkholderia cenocepacia |
| GKJWQY101BP3WG | 502 | 5 | 499 | 0 | 100% | 100% | 190714220 | Bacteria | Proteobacteria | Burkholderiaceae | Burkholderia cenocepacia |
| GKJWQY101BB8D5 | 506 | 22 | 462 | 4E-99 | 82% | 82% | 189336000 | Bacteria | Proteobacteria | Burkholderiaceae | Burkholderia multivorans |
| GKJWQY101BJXXU | 557 | 260 | 555 | 2E-137 | 97% | 97% | 189338899 | Bacteria | Proteobacteria | Burkholderiaceae | Burkholderia multivorans |
| GKJWQY101A2NI2 | 512 | 214 | 474 | 1E-34 | 78% | 78% | 189332915 | Bacteria | Proteobacteria | Burkholderiaceae | Burkholderia multivorans |
| GKJWQY101AY842 | 526 | 5 | 519 | 0 | 98% | 98% | 189338131 | Bacteria | Proteobacteria | Burkholderiaceae | Burkholderia multivorans |
| JQ999294 | 563 | 18 | 556 | 0 | 94% | 94% | 290457127 | Bacteria | Proteobacteria | Burkholderiaceae | Burkholderia vietnamiensis |
| GKJWQY101A1LOF | 527 | 6 | 520 | 0 | 97% | 97% | 134134073 | Bacteria | Proteobacteria | Burkholderiaceae | Burkholderia vietnamiensis |
| GKJWQY101ACN2B | 554 | 9 | 552 | 0 | 99% | 99% | 134137285 | Bacteria | Proteobacteria | Burkholderiaceae | Burkholderia vietnamiensis |
| GKJWQY101A02CY | 512 | 5 | 511 | 0 | 99% | 99% | 134132180 | Bacteria | Proteobacteria | Burkholderiaceae | Burkholderia vietnamiensis |
| GKJWQY101B056W | 577 | 4 | 572 | 0 | 93% | 93% | 134135188 | Bacteria | Proteobacteria | Burkholderiaceae | Burkholderia vietnamiensis |
| GKJWQY101AEMEY | 513 | 16 | 513 | 0 | 96% | 96% | 28974940 | Bacteria | Proteobacteria | Ralstoniaceae | Ralstonia pickettii |
| GKJWQY101ARIRG | 539 | 39 | 414 | 3E-146 | 92% | 92% | 601939 | Bacteria | Proteobacteria | Ralstoniaceae | Ralstonia pickettii |
| GKJWQY101ALG7X | 519 | 7 | 518 | 0 | 92% | 92% | 240863652 | Bacteria | Proteobacteria | Ralstoniaceae | Ralstonia pickettii |
| GKJWQY101APKXX | 485 | 3 | 480 | 0 | 92% | 92% | 240867064 | Bacteria | Proteobacteria | Ralstoniaceae | Ralstonia pickettii |

| | | | | | | | | | | | |
|---|---|---|---|---|---|---|---|---|---|---|---|
| GKJWQY101BDQBV | 545 | 20 | 544 | 0 | 99% | 99% | 187724002 | Bacteria | Proteobacteria | Ralstoniaceae | Ralstonia pickettii |
| GKJWQY101AAZQK | 550 | 5 | 541 | 0 | 91% | 91% | 240868245 | Bacteria | Proteobacteria | Ralstoniaceae | Ralstonia pickettii |
| GKJWQY101A3210 | 489 | 17 | 431 | 0 | 96% | 96% | 124257968 | Bacteria | Proteobacteria | Comamonadaceae | Methylibium petroleiphilum |
| GKJWQY101BPNEG | 528 | 24 | 468 | 0 | 100% | 100% | 262206648 | Bacteria | Proteobacteria | Comamonadaceae | Comamonas testosteroni |
| GKJWQY101BJ06O | 538 | 17 | 534 | 0 | 98% | 98% | 239799596 | Bacteria | Proteobacteria | Comamonadaceae | Variovorax paradoxus |
| GKJWQY101BNV97 | 515 | 55 | 512 | 1E-178 | 92% | 92% | 170774137 | Bacteria | Proteobacteria | Comamonadaceae | Leptothrix cholodnii |
| GKJWQY101AP1J4 | 648 | 9 | 306 | 4E-61 | 84% | 84% | 190010013 | Bacteria | Proteobacteria | Xanthomonadaceae | Stenotrophomonas maltophilia |
| GKJWQY101AXBCM | 557 | 18 | 557 | 0 | 95% | 95% | 288887617 | Bacteria | Proteobacteria | Enterobacteriaceae | Klebsiella variicola |
| GKJWQY101BYDKG | 535 | 19 | 533 | 0 | 97% | 97% | 260447279 | Bacteria | Proteobacteria | Enterobacteriaceae | Escherichia coli |
| GKJWQY101BGYZ8 | 410 | 5 | 361 | 0 | 100% | 100% | 294489418 | Bacteria | Proteobacteria | Enterobacteriaceae | Escherichia coli |
| GKJWQY101AVWVM | 539 | 5 | 532 | 0 | 90% | 90% | 290760697 | Bacteria | Proteobacteria | Enterobacteriaceae | Escherichia coli |
| GKJWQY101BUK33 | 498 | 5 | 237 | 1E-79 | 90% | 90% | 295054830 | Bacteria | Proteobacteria | Enterobacteriaceae | Enterobacter cloacae |
| GKJWQY101BJ7OH | 517 | 18 | 516 | 0 | 100% | 100% | 295059951 | Bacteria | Proteobacteria | Enterobacteriaceae | Enterobacter cloacae |
| JQ999405 | 609 | 13 | 64 | 5E-15 | 98% | 98% | 215435094 | Bacteria | Proteobacteria | Pseudomonadaceae | Pseudomonas aeruginosa |
| GKJWQY101ADACY | 576 | 7 | 567 | 0 | 90% | 90% | 218768969 | Bacteria | Proteobacteria | Pseudomonadaceae | Pseudomonas aeruginosa |
| JQ999404 | 428 | 47 | 364 | 5E-133 | 94% | 94% | 285028782 | Bacteria | Proteobacteria | Pseudomonadaceae | Pseudomonas aeruginosa |
| GKJWQY101AGCOW | 420 | 5 | 240 | 3E-100 | 95% | 95% | 150958624 | Bacteria | Proteobacteria | Pseudomonadaceae | Pseudomonas aeruginosa |
| GKJWQY101A6JM9 | 537 | 23 | 508 | 0 | 99% | 99% | 95101722 | Bacteria | Proteobacteria | Pseudomonadaceae | Pseudomonas entomophila |
| GKJWQY101A34YN | 233 | 25 | 195 | 6E-65 | 93% | 93% | 7546742 | Bacteria | Proteobacteria | Pseudomonadaceae | Pseudomonas fluorescens |
| GKJWQY101BV9BO | 477 | 5 | 414 | 0 | 98% | 98% | 68342549 | Bacteria | Proteobacteria | Pseudomonadaceae | Pseudomonas fluorescens |
| GKJWQY101AYR98 | 500 | 18 | 492 | 2E-127 | 85% | 85% | 171705315 | Bacteria | Proteobacteria | Pseudomonadaceae | Pseudomonas fluorescens |
| GKJWQY101A1SM7 | 594 | 17 | 593 | 0 | 98% | 98% | 229359445 | Bacteria | Proteobacteria | Pseudomonadaceae | Pseudomonas fluorescens |
| GKJWQY101B1U2N | 566 | 6 | 562 | 0 | 97% | 97% | 253992019 | Bacteria | Proteobacteria | Pseudomonadaceae | Pseudomonas fluorescens |
| JQ999408 | 560 | 5 | 557 | 0 | 99% | 99% | 118026408 | Bacteria | Proteobacteria | Pseudomonadaceae | Pseudomonas fluorescens |
| GKJWQY101AZRPU | 177 | 5 | 130 | 2E-58 | 100% | 100% | 295646750 | Bacteria | Proteobacteria | Pseudomonadaceae | Pseudomonas fluorescens |
| GKJWQY101A1CE6 | 212 | 3 | 167 | 5E-80 | 100% | 100% | 295149356 | Bacteria | Proteobacteria | Pseudomonadaceae | Pseudomonas mendocina |
| GKJWQY101BF9AJ | 569 | 17 | 532 | 0 | 99% | 99% | 145573243 | Bacteria | Proteobacteria | Pseudomonadaceae | Pseudomonas mendocina |
| GKJWQY101AZLOG | 580 | 5 | 579 | 0 | 98% | 98% | 169757190 | Bacteria | Proteobacteria | Pseudomonadaceae | Pseudomonas putida |
| GKJWQY101BZK7N | 571 | 17 | 567 | 0 | 98% | 98% | 148509317 | Bacteria | Proteobacteria | Pseudomonadaceae | Pseudomonas putida |
| GKJWQY101BA5SM | 560 | 5 | 558 | 0 | 98% | 98% | 166857509 | Bacteria | Proteobacteria | Pseudomonadaceae | Pseudomonas putida |
| GKJWQY101BQFXA | 514 | 24 | 510 | 0 | 99% | 99% | 24987239 | Bacteria | Proteobacteria | Pseudomonadaceae | Pseudomonas putida |
| JQ999412 | 397 | 18 | 350 | 2E-171 | 100% | 100% | 4928221 | Bacteria | Proteobacteria | Pseudomonadaceae | Pseudomonas putida |
| JQ999416 | 540 | 18 | 539 | 0 | 100% | 100% | 183585700 | Bacteria | Proteobacteria | Pseudomonadaceae | Pseudomonas putida |
| JQ999413 | 398 | 18 | 363 | 2E-180 | 100% | 100% | 227433755 | Bacteria | Proteobacteria | Pseudomonadaceae | Pseudomonas putida |
| JQ999414 | 539 | 18 | 403 | 0 | 99% | 99% | 254621800 | Bacteria | Proteobacteria | Pseudomonadaceae | Pseudomonas putida |
| JQ999417 | 544 | 5 | 484 | 0 | 100% | 100% | 295083378 | Bacteria | Proteobacteria | Pseudomonadaceae | Pseudomonas putida |
| JQ999415 | 539 | 81 | 537 | 0 | 98% | 98% | 295646754 | Bacteria | Proteobacteria | Pseudomonadaceae | Pseudomonas putida |
| JQ999411 | 335 | 5 | 289 | 2E-146 | 100% | 100% | 295814491 | Bacteria | Proteobacteria | Pseudomonadaceae | Pseudomonas putida |
| GKJWQY101AY10W | 527 | 18 | 55 | 0.00004 | 95% | 95% | 13310118 | Bacteria | Proteobacteria | Pseudomonadaceae | Pseudomonas putida |
| GKJWQY101A3Z0H | 573 | 4 | 514 | 0 | 97% | 97% | 71553748 | Bacteria | Proteobacteria | Pseudomonadaceae | Pseudomonas savastanoi |
| GKJWQY101AU6HQ | 497 | 14 | 493 | 0 | 98% | 98% | 60115908 | Bacteria | Proteobacteria | Pseudomonadaceae | Pseudomonas stutzeri |
| GKJWQY101BQTGJ | 598 | 5 | 595 | 0 | 96% | 96% | 145568602 | Bacteria | Proteobacteria | Pseudomonadaceae | Pseudomonas stutzeri |
| GKJWQY101APOFH | 134 | 5 | 88 | 2E-32 | 98% | 98% | 1718243 | Bacteria | Proteobacteria | Pseudomonadaceae | Pseudomonas stutzeri |
| GKJWQY101BSMOV | 275 | 106 | 249 | 5E-61 | 97% | 97% | 227452753 | Bacteria | Proteobacteria | Pseudomonadaceae | Pseudomonas stutzeri |
| JQ999821 | 579 | 5 | 576 | 0 | 99% | 99% | 2244673 | Bacteria | Proteobacteria | Pseudomonadaceae | Pseudomonas stutzeri |
| JQ999452 | 325 | 5 | 226 | 1E-98 | 97% | 97% | 7321259 | Bacteria | Proteobacteria | Pseudomonadaceae | Pseudomonas stutzeri |
| JQ999451 | 284 | 17 | 179 | 2E-55 | 92% | 92% | 12832002 | Bacteria | Proteobacteria | Pseudomonadaceae | Pseudomonas stutzeri |
| JQ999453 | 342 | 5 | 302 | 4E-152 | 100% | 100% | 15282431 | Bacteria | Proteobacteria | Pseudomonadaceae | Pseudomonas stutzeri |
| JQ999450 | 264 | 5 | 216 | 1E-56 | 88% | 88% | 19338604 | Bacteria | Proteobacteria | Pseudomonadaceae | Pseudomonas stutzeri |
| JQ999463 | 539 | 5 | 536 | 0 | 99% | 99% | 22474444 | Bacteria | Proteobacteria | Pseudomonadaceae | Pseudomonas stutzeri |
| JQ999468 | 567 | 5 | 564 | 0 | 99% | 99% | 77456204 | Bacteria | Proteobacteria | Pseudomonadaceae | Pseudomonas stutzeri |
| JQ999470 | 575 | 5 | 575 | 0 | 99% | 99% | 86211364 | Bacteria | Proteobacteria | Pseudomonadaceae | Pseudomonas stutzeri |
| JQ999467 | 559 | 5 | 559 | 0 | 99% | 99% | 102231497 | Bacteria | Proteobacteria | Pseudomonadaceae | Pseudomonas stutzeri |
| JQ999461 | 510 | 5 | 288 | 4E-144 | 100% | 100% | 112820874 | Bacteria | Proteobacteria | Pseudomonadaceae | Pseudomonas stutzeri |
| JQ999459 | 476 | 15 | 431 | 0 | 99% | 99% | 194399053 | Bacteria | Proteobacteria | Pseudomonadaceae | Pseudomonas stutzeri |

| | | | | | | | | | | | |
|---|---|---|---|---|---|---|---|---|---|---|---|
| JQ999469 | 568 | 67 | 541 | 0 | 94% | 94% | 209981671 | Bacteria | Proteobacteria | Pseudomonadaceae | Pseudomonas stutzeri |
| JQ999464 | 544 | 16 | 529 | 0 | 100% | 100% | 256861793 | Bacteria | Proteobacteria | Pseudomonadaceae | Pseudomonas stutzeri |
| JQ999466 | 556 | 18 | 556 | 0 | 99% | 99% | 257043242 | Bacteria | Proteobacteria | Pseudomonadaceae | Pseudomonas stutzeri |
| JQ999458 | 433 | 5 | 371 | 3E-175 | 97% | 97% | 281191476 | Bacteria | Proteobacteria | Pseudomonadaceae | Pseudomonas stutzeri |
| JQ999462 | 513 | 142 | 439 | 3E-121 | 94% | 94% | 285206748 | Bacteria | Proteobacteria | Pseudomonadaceae | Pseudomonas stutzeri |
| JQ999456 | 395 | 18 | 360 | 5E-177 | 100% | 100% | 289547136 | Bacteria | Proteobacteria | Pseudomonadaceae | Pseudomonas stutzeri |
| JQ999455 | 382 | 18 | 203 | 4E-88 | 99% | 99% | 294662661 | Bacteria | Proteobacteria | Pseudomonadaceae | Pseudomonas stutzeri |
| JQ999454 | 355 | 18 | 307 | 1E-147 | 100% | 100% | 295810392 | Bacteria | Proteobacteria | Pseudomonadaceae | Pseudomonas stutzeri |
| JQ999460 | 498 | 4 | 443 | 0 | 98% | 98% | 295810394 | Bacteria | Proteobacteria | Pseudomonadaceae | Pseudomonas stutzeri |
| JQ999457 | 399 | 18 | 344 | 2E-161 | 98% | 98% | 295810395 | Bacteria | Proteobacteria | Pseudomonadaceae | Pseudomonas stutzeri |
| JQ999465 | 554 | 5 | 549 | 0 | 99% | 99% | 295810397 | Bacteria | Proteobacteria | Pseudomonadaceae | Pseudomonas stutzeri |
| GKJWQY101A8AMI | 547 | 20 | 307 | 6E-123 | 95% | 95% | 213985689 | Bacteria | Proteobacteria | Moraxellaceae | Acinetobacter baumannii |
| JQ999816 | 560 | 13 | 558 | 0 | 90% | 90% | 168148844 | Bacteria | Proteobacteria | Halomonadaceae | Halomonas sulfidaeris |
| JQ999822 | 310 | 5 | 272 | 5E-126 | 98% | 98% | 1913845 | Bacteria | Proteobacteria | Xanthomonadaceae | Xanthomonas fragariae |
| GKJWQY101BEX3C | 583 | 5 | 583 | 0 | 98% | 98% | 92392509 | Bacteria | Proteobacteria | Moraxellaceae | Psychrobacter cryohalolentis |
| JQ999502 | 324 | 5 | 284 | 2.00E-110 | 93% | 93% | 157367002 | Bacteria | Proteobacteria | Pseudomonadaceae | uncultured Pseudomonas sp. |
| JQ999339 | 437 | 18 | 391 | 3.00E-179 | 97% | 97% | 60266657 | Bacteria | Proteobacteria | n | uncultured gamma proteobacterium |
| GKJWQY101B2XIV | 586 | 5 | 580 | 0 | 91% | 91% | 156769729 | Bacteria | n | n | uncultured bacterium |
| GKJWQY101BLIPI | 556 | 2 | 555 | 0 | 95% | 95% | 156768841 | Bacteria | n | n | uncultured bacterium |
| JQ999031 | 554 | 20 | 554 | 0 | 92% | 92% | 92087350 | Bacteria | n | n | uncultured bacterium |
| JQ998949 | 547 | 24 | 544 | 0 | 96% | 96% | 189182700 | Bacteria | n | n | uncultured bacterium |
| JQ998015 | 303 | 18 | 259 | 1.00E-122 | 100% | 100% | 192787447 | Bacteria | n | n | uncultured bacterium |
| JQ999098 | 559 | 17 | 418 | 0 | 97% | 97% | 209170703 | Bacteria | n | n | uncultured bacterium |
| JQ998675 | 523 | 18 | 520 | 0 | 98% | 98% | 224569115 | Bacteria | n | n | uncultured bacterium |
| JQ998571 | 495 | 15 | 379 | 0 | 99% | 99% | 229429018 | Bacteria | n | n | uncultured bacterium |
| JQ999027 | 553 | 19 | 520 | 0 | 100% | 100% | 238330523 | Bacteria | n | n | uncultured bacterium |
| JQ998515 | 478 | 5 | 438 | 0 | 99% | 99% | 238415793 | Bacteria | n | n | uncultured bacterium |
| JQ998455 | 456 | 8 | 400 | 0 | 98% | 98% | 256355282 | Bacteria | n | n | uncultured bacterium |
| JQ999190 | 602 | 4 | 595 | 0 | 91% | 91% | 256592892 | Bacteria | n | n | uncultured bacterium |
| JQ999149 | 567 | 18 | 565 | 0 | 94% | 94% | 285960330 | Bacteria | n | n | uncultured bacterium |
| JQ999185 | 588 | 3 | 584 | 0 | 93% | 93% | 285960363 | Bacteria | n | n | uncultured bacterium |
| JQ998650 | 517 | 69 | 463 | 0 | 100% | 100% | 289185872 | Bacteria | n | n | uncultured bacterium |
| JQ999182 | 581 | 1 | 577 | 0 | 91% | 91% | 289656597 | Bacteria | n | n | uncultured bacterium |
| JQ998096 | 331 | 5 | 270 | 1.00E-127 | 98% | 98% | 291192742 | Bacteria | n | n | uncultured bacterium |
| JQ998495 | 469 | 5 | 419 | 0 | 96% | 96% | 291192757 | Bacteria | n | n | uncultured bacterium |
| JQ999774 | 545 | 137 | 529 | 6E-158 | 93% | 93% | 291259015 | Bacteria | n | n | uncultured bacterium |
| JQ999729 | 362 | 18 | 261 | 2E-90 | 92% | 92% | 291260769 | Bacteria | n | n | uncultured bacterium |
| JQ999740 | 413 | 5 | 368 | 4E-158 | 95% | 95% | 291260837 | Bacteria | n | n | uncultured bacterium |
| GKJWQY101BR4RJ | 260 | 5 | 193 | 3E-93 | 100% | 100% | 295027769 | Bacteria | n | n | uncultured bacterium |
| GKJWQY101AZEVC | 445 | 1 | 445 | 0 | 96% | 96% | 83281396 | Eukaryota | Arthropoda | Culicidae | Culex quinquefasciatus |
| GKJWQY101A08VK | 28 | 1 | 28 | 0.002 | 100% | 100% | 113193577 | Eukaryota | Arthropoda | Drosophilidae | Drosophila melanogaster |
| GKJWQY101BXQ6C | 491 | 1 | 491 | 0 | 95% | 95% | 172190 | Eukaryota | Ascomycota | Saccharomycetaceae | Saccharomyces cerevisiae |
| GKJWQY101AUREB | 124 | 23 | 53 | 0.000008 | 100% | 100% | 295393257 | Eukaryota | Ascomycota | Saccharomycetaceae | Saccharomyces cerevisiae |
| GKJWQY101BBYO0 | 43 | 1 | 43 | 2E-10 | 98% | 98% | 269944715 | Eukaryota | Ascomycota | Saccharomycetaceae | Saccharomyces cerevisiae |
| GKJWQY101BBSPQ | 105 | 1 | 105 | 1E-46 | 100% | 100% | 294929468 | Eukaryota | Ascomycota | Saccharomycetaceae | Saccharomyces cerevisiae |
| JQ999577 | 553 | 17 | 543 | 0 | 93% | 93% | 225134683 | Eukaryota | Ascomycota | Trichocomaceae | Penicillium chrysogenum |
| JQ999569 | 517 | 103 | 511 | 5.00E-173 | 94% | 94% | 283827965 | Eukaryota | Ascomycota | Teratosphaeriaceae | Teratosphaeria suttonii |
| GKJWQY101AFQZR | 257 | 1 | 257 | 5E-62 | 85% | 85% | 72256214 | Eukaryota | Basidiomycota | Ustilaginaceae | Ustilago maydis |
| GKJWQY101BSALF | 484 | 1 | 484 | 1E-56 | 76% | 76% | 71022004 | Eukaryota | Basidiomycota | Ustilaginaceae | Ustilago maydis |
| JQ999870 | 548 | 18 | 544 | 0 | 95% | 95% | 228547090 | Eukaryota | Basidiomycota | Polyporaceae | Lignosus rhinocerus |
| GKJWQY101BNMZJ | 534 | 18 | 124 | 1.00E-40 | 96% | 96% | 148888555 | Eukaryota | Chordata | Bovidae | Bos indicus |
| GKJWQY101BV8EI | 508 | 288 | 478 | 6.00E-73 | 93% | 93% | 1002428 | Eukaryota | Chordata | Bovidae | Bos taurus |
| GKJWQY101BURCN | 486 | 54 | 188 | 3.00E-46 | 93% | 93% | 32364476 | Eukaryota | Chordata | Bovidae | Bos taurus |
| GKJWQY101BF0CZ | 495 | 170 | 483 | 7.00E-107 | 90% | 90% | 36988715 | Eukaryota | Chordata | Bovidae | Bos taurus |
| GKJWQY101AVJ8U | 535 | 7 | 501 | 3.00E-161 | 88% | 88% | 50363274 | Eukaryota | Chordata | Bovidae | Bos taurus |

| | | | | | | | | | | | |
|---|---|---|---|---|---|---|---|---|---|---|---|
| GKJWQY101BSNZS | 474 | 204 | 452 | 1.00E-118 | 98% | 98% | 59858218 | Eukaryota | Chordata | Bovidae | Bos taurus |
| GKJWQY101AVMZK | 474 | 153 | 237 | 2.00E-28 | 95% | 95% | 67944510 | Eukaryota | Chordata | Bovidae | Bos taurus |
| GKJWQY101B05KZ | 538 | 5 | 85 | 7.00E-33 | 100% | 100% | 83638662 | Eukaryota | Chordata | Bovidae | Bos taurus |
| GKJWQY101AO1F2 | 531 | 5 | 528 | 0 | 91% | 91% | 119216318 | Eukaryota | Chordata | Bovidae | Bos taurus |
| GKJWQY101A8YVU | 538 | 61 | 192 | 7.00E-33 | 87% | 87% | 126010708 | Eukaryota | Chordata | Bovidae | Bos taurus |
| GKJWQY101AC3Q3 | 671 | 18 | 109 | 1.00E-26 | 92% | 92% | 126033171 | Eukaryota | Chordata | Bovidae | Bos taurus |
| GKJWQY101BG5W1 | 523 | 215 | 475 | 3.00E-91 | 91% | 91% | 148743975 | Eukaryota | Chordata | Bovidae | Bos taurus |
| GKJWQY101AM38L | 551 | 210 | 536 | 1.00E-125 | 92% | 92% | 151554659 | Eukaryota | Chordata | Bovidae | Bos taurus |
| GKJWQY101A2KRZ | 508 | 218 | 483 | 6.00E-53 | 82% | 82% | 151556917 | Eukaryota | Chordata | Bovidae | Bos taurus |
| GKJWQY101A35JD | 485 | 231 | 479 | 3.00E-81 | 89% | 89% | 154425576 | Eukaryota | Chordata | Bovidae | Bos taurus |
| GKJWQY101AO1F2 | 531 | 5 | 528 | 0 | 91% | 91% | 119216318 | Eukaryota | Chordata | Bovidae | Bos taurus |
| GKJWQY101A8YVU | 538 | 61 | 192 | 7.00E-33 | 87% | 87% | 126010708 | Eukaryota | Chordata | Bovidae | Bos taurus |
| GKJWQY101AC3Q3 | 671 | 18 | 109 | 1.00E-26 | 92% | 92% | 126033171 | Eukaryota | Chordata | Bovidae | Bos taurus |
| GKJWQY101BG5W1 | 523 | 215 | 475 | 3.00E-91 | 91% | 91% | 148743975 | Eukaryota | Chordata | Bovidae | Bos taurus |
| GKJWQY101AM38L | 551 | 210 | 536 | 1.00E-125 | 92% | 92% | 151554659 | Eukaryota | Chordata | Bovidae | Bos taurus |
| GKJWQY101A2KRZ | 508 | 218 | 483 | 6.00E-53 | 82% | 82% | 151556917 | Eukaryota | Chordata | Bovidae | Bos taurus |
| GKJWQY101A35JD | 485 | 231 | 479 | 3.00E-81 | 89% | 89% | 154425576 | Eukaryota | Chordata | Bovidae | Bos taurus |
| GKJWQY101BW7C1 | 558 | 44 | 152 | 9.00E-37 | 94% | 94% | 188485453 | Eukaryota | Chordata | Bovidae | Bos taurus |
| GKJWQY101AECTS | 487 | 197 | 416 | 1.00E-94 | 96% | 96% | 188485455 | Eukaryota | Chordata | Bovidae | Bos taurus |
| GKJWQY101A6Y0M | 586 | 514 | 564 | 4.00E-11 | 94% | 94% | 214010996 | Eukaryota | Chordata | Bovidae | Bos taurus |
| GKJWQY101BWLQC | 551 | 5 | 488 | 6.00E-168 | 90% | 90% | 270310991 | Eukaryota | Chordata | Bovidae | Bos taurus |
| GKJWQY101AV25T | 516 | 18 | 516 | 0 | 95% | 95% | 270310993 | Eukaryota | Chordata | Bovidae | Bos taurus |
| GKJWQY101BKUSG | 547 | 70 | 369 | 2.00E-82 | 87% | 87% | 211998866 | Eukaryota | Chordata | Bovidae | Bos taurus |
| GKJWQY101ABYCR | 519 | 179 | 449 | 2.00E-112 | 94% | 94% | 163565 | Eukaryota | Chordata | Bovidae | Bos taurus |
| GKJWQY101BL45R | 458 | 101 | 189 | 3.00E-16 | 86% | 86% | 3873616 | Eukaryota | Chordata | Bovidae | Bos taurus |
| GKJWQY101BDGMI | 512 | 18 | 509 | 0 | 96% | 96% | 13569587 | Eukaryota | Chordata | Bovidae | Bos taurus |
| GKJWQY101BMRAG | 546 | 40 | 439 | 0 | 100% | 100% | 14594798 | Eukaryota | Chordata | Bovidae | Bos taurus |
| GKJWQY101AA90W | 514 | 19 | 503 | 5.00E-143 | 86% | 86% | 21425595 | Eukaryota | Chordata | Bovidae | Bos taurus |
| GKJWQY101ARRLV | 257 | 27 | 212 | 1.00E-81 | 97% | 97% | 27227458 | Eukaryota | Chordata | Bovidae | Bos taurus |
| GKJWQY101BWE5U | 523 | 146 | 347 | 8.00E-57 | 87% | 87% | 29692104 | Eukaryota | Chordata | Bovidae | Bos taurus |
| GKJWQY101BYYPR | 520 | 152 | 356 | 2.00E-53 | 86% | 86% | 46850518 | Eukaryota | Chordata | Bovidae | Bos taurus |
| GKJWQY101AAK5N | 520 | 91 | 294 | 2.00E-62 | 89% | 89% | 52839263 | Eukaryota | Chordata | Bovidae | Bos taurus |
| GKJWQY101BXKWL | 518 | 19 | 516 | 0 | 91% | 91% | 56411964 | Eukaryota | Chordata | Bovidae | Bos taurus |
| GKJWQY101BCK3R | 513 | 17 | 512 | 0 | 94% | 94% | 63169154 | Eukaryota | Chordata | Bovidae | Bos taurus |
| GKJWQY101BCG59 | 533 | 16 | 532 | 0 | 95% | 95% | 66734170 | Eukaryota | Chordata | Bovidae | Bos taurus |
| GKJWQY101BKG3M | 533 | 20 | 530 | 4.00E-129 | 84% | 84% | 83286786 | Eukaryota | Chordata | Bovidae | Bos taurus |
| GKJWQY101BSEK5 | 618 | 139 | 166 | 0.002 | 100% | 100% | 129561996 | Eukaryota | Chordata | Bovidae | Bos taurus |
| GKJWQY101BQ1W1 | 560 | 10 | 507 | 1.00E-155 | 88% | 88% | 134244145 | Eukaryota | Chordata | Bovidae | Bos taurus |
| GKJWQY101BHOYS | 528 | 223 | 494 | 6.00E-133 | 99% | 99% | 31341883 | Eukaryota | Chordata | Bovidae | Bos taurus |
| GKJWQY101BFBG8 | 547 | 216 | 468 | 1.00E-109 | 96% | 96% | 31342962 | Eukaryota | Chordata | Bovidae | Bos taurus |
| GKJWQY101BBE6M | 231 | 5 | 183 | 9.00E-88 | 100% | 100% | 47564057 | Eukaryota | Chordata | Bovidae | Bos taurus |
| GKJWQY101BD9DT | 552 | 18 | 95 | 3.00E-31 | 100% | 100% | 74267625 | Eukaryota | Chordata | Bovidae | Bos taurus |
| GKJWQY101ACOHL | 558 | 135 | 274 | 2.00E-34 | 87% | 87% | 77735986 | Eukaryota | Chordata | Bovidae | Bos taurus |
| GKJWQY101AWRQM | 511 | 76 | 486 | 6.00E-48 | 76% | 76% | 94574056 | Eukaryota | Chordata | Bovidae | Bos taurus |
| GKJWQY101ALMP9 | 585 | 100 | 236 | 1.00E-60 | 99% | 99% | 115495638 | Eukaryota | Chordata | Bovidae | Bos taurus |
| GKJWQY101BZ030 | 560 | 20 | 533 | 0 | 98% | 98% | 115496399 | Eukaryota | Chordata | Bovidae | Bos taurus |
| GKJWQY101BW03O | 515 | 70 | 162 | 6.00E-38 | 99% | 99% | 115496837 | Eukaryota | Chordata | Bovidae | Bos taurus |
| GKJWQY101B2T6S | 554 | 481 | 549 | 9.00E-22 | 96% | 96% | 115497327 | Eukaryota | Chordata | Bovidae | Bos taurus |
| GKJWQY101BUUKG | 515 | 123 | 512 | 0 | 99% | 99% | 118150885 | Eukaryota | Chordata | Bovidae | Bos taurus |
| GKJWQY101AB70E | 532 | 43 | 147 | 5.00E-44 | 99% | 99% | 122692336 | Eukaryota | Chordata | Bovidae | Bos taurus |
| GKJWQY101BSN1F | 579 | 133 | 174 | 2.00E-09 | 98% | 98% | 125991941 | Eukaryota | Chordata | Bovidae | Bos taurus |
| GKJWQY101BBORT | 108 | 1 | 62 | 4.00E-23 | 100% | 100% | 148227015 | Eukaryota | Chordata | Bovidae | Bos taurus |
| GKJWQY101AHX0L | 436 | 20 | 194 | 7.00E-57 | 90% | 90% | 149642896 | Eukaryota | Chordata | Bovidae | Bos taurus |
| GKJWQY101BA3QT | 520 | 403 | 514 | 1.00E-30 | 91% | 91% | 154152074 | Eukaryota | Chordata | Bovidae | Bos taurus |
| GKJWQY101BRRAJ | 517 | 194 | 413 | 4.00E-55 | 85% | 85% | 156120486 | Eukaryota | Chordata | Bovidae | Bos taurus |

| | | | | | | | | | | | |
|---|---|---|---|---|---|---|---|---|---|---|---|
| GKJWQY101AZUNM | 522 | 5 | 198 | 6.00E-63 | 90% | 90% | 156120792 | Eukaryota | Chordata | Bovidae | Bos taurus |
| GKJWQY101A32BJ | 526 | 5 | 77 | 9.00E-27 | 99% | 99% | 156523067 | Eukaryota | Chordata | Bovidae | Bos taurus |
| GKJWQY101BIASS | 446 | 23 | 184 | 7.00E-47 | 88% | 88% | 157073991 | Eukaryota | Chordata | Bovidae | Bos taurus |
| GKJWQY101BWCRK | 599 | 18 | 55 | 6.00E-09 | 100% | 100% | 157074093 | Eukaryota | Chordata | Bovidae | Bos taurus |
| GKJWQY101APUR7 | 513 | 348 | 500 | 2.00E-33 | 85% | 85% | 157074095 | Eukaryota | Chordata | Bovidae | Bos taurus |
| GKJWQY101A8WC2 | 557 | 387 | 555 | 8.00E-82 | 100% | 100% | 157428077 | Eukaryota | Chordata | Bovidae | Bos taurus |
| GKJWQY101A3AP7 | 332 | 42 | 236 | 2.00E-90 | 98% | 98% | 158937292 | Eukaryota | Chordata | Bovidae | Bos taurus |
| GKJWQY101BNNNZ | 541 | 18 | 510 | 0 | 99% | 99% | 14336700 | Eukaryota | Chordata | Hominidae | Homo sapiens |
| GKJWQY101AI583 | 524 | 5 | 75 | 2.00E-22 | 96% | 96% | 262331521 | Eukaryota | Chordata | Hominidae | Homo sapiens |
| GKJWQY101BIW6Y | 573 | 5 | 570 | 0 | 96% | 96% | 197245396 | Eukaryota | Chordata | Hominidae | Homo sapiens |
| GKJWQY101ALYO2 | 519 | 18 | 516 | 0 | 98% | 98% | 224922786 | Eukaryota | Chordata | Hominidae | Homo sapiens |
| GKJWQY101A1CZ5 | 606 | 5 | 259 | 1.00E-119 | 98% | 98% | 163954924 | Eukaryota | Chordata | Hominidae | Homo sapiens |
| GKJWQY101AD8KV | 559 | 19 | 106 | 2.00E-18 | 88% | 88% | 193220939 | Eukaryota | Chordata | Hominidae | Homo sapiens |
| GKJWQY101BSGL8 | 510 | 20 | 358 | 6.00E-63 | 81% | 81% | 291290993 | Eukaryota | Chordata | Hominidae | Homo sapiens |
| GKJWQY101BSMSU | 558 | 18 | 438 | 0 | 98% | 98% | 194385695 | Eukaryota | Chordata | Hominidae | Homo sapiens |
| GKJWQY101BNWXC | 522 | 329 | 513 | 8.00E-32 | 82% | 82% | 255652918 | Eukaryota | Chordata | Hominidae | Homo sapiens |
| GKJWQY101AYL0B | 524 | 18 | 498 | 0 | 99% | 99% | 3289998 | Eukaryota | Chordata | Hominidae | Homo sapiens |
| GKJWQY101BZ34P | 542 | 24 | 534 | 0 | 95% | 95% | 124302213 | Eukaryota | Chordata | Hominidae | Homo sapiens |
| GKJWQY101BGPYK | 537 | 18 | 356 | 1.00E-84 | 84% | 84% | 30023945 | Eukaryota | Chordata | Hominidae | Homo sapiens |
| GKJWQY101AOCTV | 445 | 5 | 389 | 8.00E-131 | 89% | 89% | 134152716 | Eukaryota | Chordata | Hominidae | Homo sapiens |
| GKJWQY101BXZZB | 632 | 23 | 488 | 1.00E-159 | 89% | 89% | 195927052 | Eukaryota | Chordata | Hominidae | Homo sapiens |
| GKJWQY101AVDNO | 528 | 5 | 526 | 0 | 95% | 95% | 219520697 | Eukaryota | Chordata | Hominidae | Homo sapiens |
| GKJWQY101BITIR | 518 | 18 | 515 | 0 | 98% | 98% | 282396079 | Eukaryota | Chordata | Hominidae | Homo sapiens |
| GKJWQY101BQ8KU | 231 | 5 | 163 | 7.00E-64 | 95% | 95% | 237874182 | Eukaryota | Chordata | Hominidae | Homo sapiens |
| GKJWQY101BHE6J | 306 | 197 | 240 | 1.00E-07 | 93% | 93% | 219521881 | Eukaryota | Chordata | Hominidae | Homo sapiens |
| GKJWQY101A3FOT | 565 | 5 | 475 | 5.00E-64 | 77% | 77% | 261278320 | Eukaryota | Chordata | Hominidae | Homo sapiens |
| GKJWQY101ARFPP | 536 | 17 | 489 | 0 | 95% | 95% | 291084840 | Eukaryota | Chordata | Hominidae | Homo sapiens |
| GKJWQY101ACOO1 | 537 | 4 | 518 | 0 | 95% | 95% | 73697497 | Eukaryota | Chordata | Hominidae | Homo sapiens |
| GKJWQY101A09S8 | 500 | 18 | 433 | 0 | 97% | 97% | 254039630 | Eukaryota | Chordata | Hominidae | Homo sapiens |
| GKJWQY101AVI61 | 525 | 18 | 521 | 0 | 99% | 99% | 197333801 | Eukaryota | Chordata | Hominidae | Homo sapiens |
| GKJWQY101BJBYT | 555 | 18 | 545 | 1.00E-135 | 85% | 85% | 114306774 | Eukaryota | Chordata | Hominidae | Homo sapiens |
| GKJWQY101AT79U | 575 | 75 | 198 | 3.00E-11 | 79% | 79% | 237820692 | Eukaryota | Chordata | Hominidae | Homo sapiens |
| GKJWQY101BN89C | 516 | 18 | 512 | 0 | 97% | 97% | 150170720 | Eukaryota | Chordata | Hominidae | Homo sapiens |
| GKJWQY101B0JO4 | 588 | 17 | 588 | 0 | 98% | 98% | 167830470 | Eukaryota | Chordata | Hominidae | Homo sapiens |
| GKJWQY101BVZGM | 545 | 5 | 541 | 0 | 97% | 97% | 19718557 | Eukaryota | Chordata | Hominidae | Homo sapiens |
| GKJWQY101ATS4V | 522 | 18 | 469 | 0 | 99% | 99% | 47940445 | Eukaryota | Chordata | Hominidae | Homo sapiens |
| GKJWQY101AJ4OL | 558 | 192 | 556 | 0 | 98% | 98% | 62087205 | Eukaryota | Chordata | Hominidae | Homo sapiens |
| GKJWQY101ABSQH | 491 | 18 | 485 | 0 | 95% | 95% | 160948584 | Eukaryota | Chordata | Hominidae | Homo sapiens |
| GKJWQY101BUHAT | 214 | 5 | 121 | 3.00E-42 | 94% | 94% | 160948585 | Eukaryota | Chordata | Hominidae | Homo sapiens |
| GKJWQY101A2QDN | 617 | 18 | 600 | 0 | 89% | 89% | 270048017 | Eukaryota | Chordata | Hominidae | Homo sapiens |
| GKJWQY101BCNPF | 549 | 80 | 473 | 3.00E-126 | 88% | 88% | 293597499 | Eukaryota | Chordata | Hominidae | Homo sapiens |
| GKJWQY101AKRP1 | 595 | 7 | 589 | 0 | 94% | 94% | 224809264 | Eukaryota | Chordata | Hominidae | Homo sapiens |
| GKJWQY101BZNKX | 481 | 5 | 442 | 0 | 99% | 99% | 261823972 | Eukaryota | Chordata | Hominidae | Homo sapiens |
| GKJWQY101AR7W1 | 555 | 23 | 553 | 0 | 99% | 99% | 237874188 | Eukaryota | Chordata | Hominidae | Homo sapiens |
| GKJWQY101BTVAZ | 574 | 18 | 572 | 0 | 98% | 98% | 281182732 | Eukaryota | Chordata | Hominidae | Homo sapiens |
| GKJWQY101BSHC9 | 515 | 5 | 512 | 0 | 98% | 98% | 33341735 | Eukaryota | Chordata | Hominidae | Homo sapiens |
| GKJWQY101BJPFL | 363 | 7 | 222 | 5.00E-97 | 97% | 97% | 291190796 | Eukaryota | Chordata | Hominidae | Homo sapiens |
| GKJWQY101BERG1 | 149 | 20 | 86 | 4.00E-24 | 99% | 99% | 13625541 | Eukaryota | Chordata | Hominidae | Homo sapiens |
| GKJWQY101BXHYS | 569 | 135 | 533 | 3.00E-41 | 76% | 76% | 227330591 | Eukaryota | Chordata | Hominidae | Homo sapiens |
| GKJWQY101BOL5J | 512 | 16 | 506 | 0 | 98% | 98% | 293597503 | Eukaryota | Chordata | Hominidae | Homo sapiens |
| GKJWQY101BQ9JT | 502 | 18 | 496 | 0 | 98% | 98% | 157426899 | Eukaryota | Chordata | Hominidae | Homo sapiens |
| GKJWQY101BGJ0B | 82 | 18 | 75 | 1.00E-16 | 95% | 95% | 215983102 | Eukaryota | Chordata | Hominidae | Homo sapiens |
| GKJWQY101B17JJ | 558 | 18 | 552 | 0 | 99% | 99% | 226510210 | Eukaryota | Chordata | Hominidae | Homo sapiens |
| GKJWQY101AQCZO | 442 | 18 | 47 | 1.00E-04 | 100% | 100% | 261598996 | Eukaryota | Chordata | Hominidae | Homo sapiens |
| GKJWQY101BX9QE | 573 | 3 | 552 | 4.00E-85 | 78% | 78% | 386434 | Eukaryota | Chordata | Hominidae | Homo sapiens |

| | | | | | | | | | | | |
|---|---|---|---|---|---|---|---|---|---|---|---|
| GKJWQY101A2A1E | 473 | 224 | 252 | 5.00E-04 | 100% | 100% | 37537319 | Eukaryota | Chordata | Hominidae | Pan troglodytes |
| GKJWQY101ATLTF | 519 | 7 | 475 | 1.00E-115 | 84% | 84% | 37537322 | Eukaryota | Chordata | Hominidae | Pan troglodytes |
| GKJWQY101BSUBR | 551 | 31 | 432 | 5.00E-139 | 90% | 90% | 37537465 | Eukaryota | Chordata | Hominidae | Pan troglodytes |
| GKJWQY101AAQYP | 521 | 69 | 126 | 4.00E-10 | 90% | 90% | 172072623 | Eukaryota | Chordata | Muridae | Mus musculus |
| GKJWQY101AHH9C | 194 | 1 | 194 | 2E-40 | 83% | 83% | 45423887 | Eukaryota | Chordata | Phasianidae | Gallus gallus |
| GKJWQY101A1FPO | 439 | 1 | 439 | 2E-149 | 89% | 89% | 118085917 | Eukaryota | Chordata | Phasianidae | Gallus gallus |
| GKJWQY101BXNXM | 328 | 1 | 328 | 1E-124 | 92% | 92% | 154937557 | Eukaryota | Chordata | Phasianidae | Gallus gallus |
| JQ999602 | 295 | 5 | 245 | 9E-119 | 99% | 99% | 218047175 | Eukaryota | Chordata | Phasianidae | Gallus gallus |
| GKJWQY101A1FPO | 576 | 18 | 456 | 1.00E-149 | 89% | 89% | 118085917 | Eukaryota | Chordata | Phasianidae | Gallus gallus |
| JQ999622 | 517 | 24 | 468 | 0 | 100% | 100% | 155573953 | Eukaryota | Streptophyta | Euphorbiaceae | Euphorbia atoto |
| JQ999620 | 540 | 5 | 537 | 0 | 99% | 99% | 290782471 | Eukaryota | Streptophyta | Fagaceae | Quercus suber |
| JQ999896 | 558 | 5 | 557 | 0 | 98% | 98% | 37993790 | Eukaryota | Streptophyta | Fagaceae | Quercus suber |
| GKJWQY101BOE6X | 501 | 1 | 501 | 0 | 93% | 93% | 32994295 | Eukaryota | Streptophyta | Poaceae | Oryza sativa |
| GKJWQY101B08PD | 536 | 1 | 536 | 0 | 92% | 92% | 32995694 | Eukaryota | Streptophyta | Poaceae | Oryza sativa |
| GKJWQY101AI87M | 526 | 1 | 526 | 0 | 98% | 98% | 99651997 | Eukaryota | Streptophyta | Poaceae | Oryza sativa |
