# Supplementary material for: Subglacial Lake Vostok (Antarctica) Accretion Ice Contains a Diverse Set of Sequences from Aquatic, Marine and Sediment-Inhabiting Bacteria and Eukarya
Source: PLoS One. 2013 Jul 3;8(7):e67221. doi: 10.1371/journal.pone.0067221 (PMC3700977; doi:10.1371/journal.pone.0067221)
Supplement: Table S15 — Sequences removed from the V6 data set that were identical or similar to sequence from controls. [“n” indicates information not specified in the NCBI GenBank database.]. (PDF) [file pone.0067221.s020.pdf]

Table S15. Sequences removed from the V6 data set that were identical or similar to sequence from controls. ["n" indicates information not specified in the NCBI GenBank database.]

Accession number / Contig ID	Q length	Q start	Q end	e-value	%-ident	%-sim	GI number	Domain	Phylum	Family	Genus / Species
VostokV6_c1	2175	1	2175	0	100%	100%	291375051	Bacteria	Actinobacteria	Propionibacteriaceae	Propionibacterium acnes
VostokV6_c21	1016	1	1016	0	99%	99%	239837778	Bacteria	Actinobacteria	Micrococcaceae	Micrococcus luteus
VostokV6_rep_c162	209	12	209	2.00E-63	90%	90%	171850984	Bacteria	Actinobacteria	Corynebacteriaceae	Corynebacterium urealyticum
VostokV6_rep_c241	71	1	71	9.00E-27	99%	99%	227452846	Bacteria	Actinobacteria	Corynebacteriaceae	Corynebacterium aurimucosum
VostokV6_c269	191	1	191	1.00E-85	97%	97%	134265192	Bacteria	Firmicutes	Bacillaceae	Geobacillus thermodenitrificans
VostokV6_c263	154	1	154	2E-68	98%	98%	9664721	Bacteria	Firmicutes	Staphylococcaceae	Staphylococcus epidermidis
VostokV6_c4	896	1	896	0	99%	99%	9624251	Bacteria	Firmicutes	Staphylococcaceae	Staphylococcus epidermidis
VostokV6_rep_c178	168	1	168	8E-82	100%	100%	9664799	Bacteria	Firmicutes	Staphylococcaceae	Staphylococcus epidermidis
VostokV6_c66	57	1	57	4E-19	98%	98%	288906474	Bacteria	Firmicutes	Staphylococcaceae	Streptococcus mitis
JQ999835	250	1	250	2.00E-105	95%	95%	296416	Bacteria	Firmicutes	Planococcaceae	Sporosarcina globispora
JQ999836	1187	5	1175	0	97%	97%	2244633	Bacteria	Proteobacteria	Caulobacteraceae	Brevundimonas diminuta
VostokV6_c144	253	68	253	2E-80	96%	96%	115280044	Bacteria	Proteobacteria	Burkholderiaceae	Burkholderia ambifaria
VostokV6_c11	1091	23	1091	0	95%	95%	190714218	Bacteria	Proteobacteria	Burkholderiaceae	Burkholderia cenocepacia
VostokV6_c20	548	8	548	0	93%	93%	105891751	Bacteria	Proteobacteria	Burkholderiaceae	Burkholderia cenocepacia
VostokV6_c118	120	1	120	2E-55	100%	100%	189338131	Bacteria	Proteobacteria	Burkholderiaceae	Burkholderia multivorans
VostokV6_c209	101	1	101	7E-45	100%	100%	134134073	Bacteria	Proteobacteria	Burkholderiaceae	Burkholderia vietnamiensis
VostokV6_c255	153	1	153	3E-71	99%	99%	134137285	Bacteria	Proteobacteria	Burkholderiaceae	Burkholderia vietnamiensis
VostokV6_c262	138	1	138	3E-65	100%	100%	134135188	Bacteria	Proteobacteria	Burkholderiaceae	Burkholderia vietnamiensis
VostokV6_c247	203	1	203	6E-94	98%	98%	145692985	Bacteria	Proteobacteria	Pseudomonadaceae	Pseudomonas aeruginosa
JQ999555	554	28	554	0	100%	100%	292386075	Bacteria	Proteobacteria	Pseudomonadaceae	Pseudomonas putida
JQ999553	215	1	212	2E-104	100%	100%	116294371	Bacteria	Proteobacteria	Pseudomonadaceae	Pseudomonas putida
VostokV6_c98	102	1	102	1E-38	96%	96%	229359445	Bacteria	Proteobacteria	Pseudomonadaceae	Pseudomonas fluorescens
VostokV6_s215	125	1	125	6E-37	91%	91%	239829322	Bacteria	Proteobacteria	Enterobacteriaceae	Escherichia coli
VostokV6_c146	924	1	924	0	91%	91%	157065147	Bacteria	Proteobacteria	Enterobacteriaceae	Escherichia coli
VostokV6_c17	2018	56	2018	0	100%	100%	606010	Bacteria	Proteobacteria	Enterobacteriaceae	Escherichia coli
VostokV6_c33	1524	29	1524	0	99%	99%	218350208	Bacteria	Proteobacteria	Enterobacteriaceae	Escherichia coli
VostokV6_c34	1654	1	1421	0	95%	95%	5801827	Bacteria	Proteobacteria	Enterobacteriaceae	Escherichia coli
VostokV6_c7	712	1	712	0	100%	100%	284919779	Bacteria	Proteobacteria	Enterobacteriaceae	Escherichia coli
VostokV6_c70	113	42	113	1E-22	95%	95%	294489418	Bacteria	Proteobacteria	Enterobacteriaceae	Escherichia coli
VostokV6_rep_c125	151	1	85	4E-34	99%	99%	290760697	Bacteria	Proteobacteria	Enterobacteriaceae	Escherichia coli
VostokV6_rep_c136	351	58	351	4E-138	98%	98%	257762509	Bacteria	Proteobacteria	Enterobacteriaceae	Escherichia coli
VostokV6_rep_c168	556	34	556	0	99%	99%	238859724	Bacteria	Proteobacteria	Enterobacteriaceae	Escherichia coli
VostokV6_rep_c198	1034	1	1034	0	100%	100%	260447279	Bacteria	Proteobacteria	Enterobacteriaceae	Escherichia coli
VostokV6_rep_c43	708	1	637	0	98%	98%	218425442	Bacteria	Proteobacteria	Enterobacteriaceae	Escherichia coli
VostokV6_rep_c52	454	1	453	0	98%	98%	291220687	Bacteria	Proteobacteria	Enterobacteriaceae	Escherichia coli
VostokV6_s116	215	1	215	2E-104	99%	99%	281177210	Bacteria	Proteobacteria	Enterobacteriaceae	Escherichia coli
VostokV6_c264	137	4	64	1.00E-14	93%	93%	288887617	Bacteria	Proteobacteria	Enterobacteriaceae	Klebsiella variicola
JQ999834	323	6	323	4.00E-142	96%	96%	1913845	Bacteria	Proteobacteria	Xanthomonadaceae	Xanthomonas fragariae
VostokV6_s265	188	1	188	3E-86	98%	98%	217416971	Bacteria	n	n	uncultured bacterium
VostokV6_c167	43	6	43	0.000000007	97%	97%	285811954	Eukaryota	Ascomycota	Saccharomycetaceae	Saccharomyces cerevisiae
VostokV6_c257	278	1	278	2E-125	96%	96%	285813361	Eukaryota	Ascomycota	Saccharomycetaceae	Saccharomyces cerevisiae
VostokV6_c71	43	1	43	3E-13	100%	100%	285813870	Eukaryota	Ascomycota	Saccharomycetaceae	Saccharomyces cerevisiae
VostokV6_c120	67	1	46	3.00E-11	96%	96%	35978	Eukaryota	Chordata	Hominidae	Homo sapiens
VostokV6_c79	61	1	61	6.00E-23	100%	100%	18653449	Eukaryota	Chordata	Hominidae	Homo sapiens
VostokV6_rep_c49	165	33	165	5.00E-59	99%	99%	118636082	Eukaryota	Chordata	Hominidae	Homo sapiens
VostokV6_c163	141	1	141	3.00E-65	99%	99%	189027236	Eukaryota	Chordata	Hominidae	Homo sapiens
