# Supplementary material for: Subglacial Lake Vostok (Antarctica) Accretion Ice Contains a Diverse Set of Sequences from Aquatic, Marine and Sediment-Inhabiting Bacteria and Eukarya
Source: PLoS One. 2013 Jul 3;8(7):e67221. doi: 10.1371/journal.pone.0067221 (PMC3700977; doi:10.1371/journal.pone.0067221)
Supplement: Table S16 — Comparison of metagenomic/metatranscriptomic sequence results with previous cultivation and sequencing results [refs. 14 and 15]. (PDF) [file pone.0067221.s021.pdf]

Table S16. Comparison of metagenomic/metatranscriptomic sequence results with previous cultivation and sequencing results [refs. 14 and 15].

Ice Core Section	Taxon (metagenomic study)[a]	Taxon (cultivation study)[a]
3563 m (V5)	***Phoma* sp.**	***Phoma* sp.**
3582 m	Antarctic alphaproteobacteria	Antarctic alphaproteobacterium
	Carnobacterium sp.	*Carnobacterium* sp.
	**Davidiellaceae sp.**	***Cladosporium* sp.**
	***Cryptococcus* sp.**	***Cryptococcus* sp.**
	**Dothiodiomycetes sp.**	**Dothioraceae sp.**
	Acinitobacter sp., *Psychrobacter* sp.	*Frigoribacterum* sp.
	***Rhodotorula* sp.**	***Rhodotorula* sp.**
3585 m (V5)	*Carnobacterium* sp.	*Carnobacterium* sp.
	Kocuria sp.	*Kocuria* sp.
	***Penicillium* sp.**	***Penicillium* sp.**
3606 m (V6)	**Agaricamycotina sp.**	***Cystofilobasidium* sp.**
	Micrococcus sp.	*Micrococcus sp.*
	**uncultured basidiomycete**	***Rhodotorula* sp.**
3610 m	***Cladosporium* sp.**	***Cladosporium* sp.**

3613 m	**uncultured ascomycete**	***Aspergillus* sp.**
	***Cladosporium* sp.**	***Cladosporium* sp.**
3619 m	**Tremellomycete sp.**	***Cryptococcus* sp.**
3621 m (V6)	**uncultured basidiomycete**	***Pseudozyma* sp.**

[a]Bold font indicates fungi
